# Modular Two-Step Route to Sulfondiimidamides

**DOI:** 10.1021/jacs.2c04404

**Published:** 2022-06-22

**Authors:** Ze-Xin Zhang, Charles Bell, Mingyan Ding, Michael C. Willis

**Affiliations:** Department of Chemistry, Chemistry Research Laboratory, University of Oxford, Mansfield Road, Oxford OX1 3TA, U.K.

## Abstract

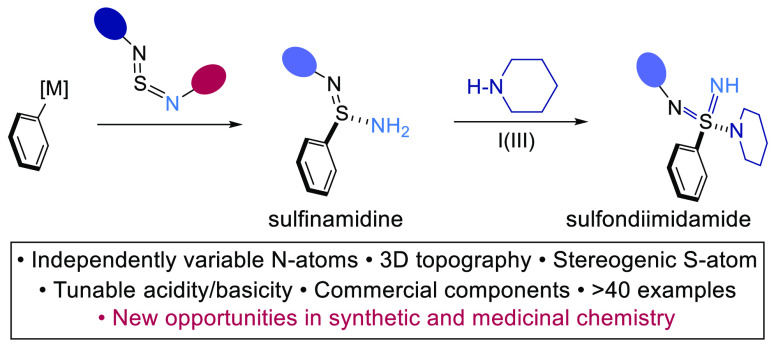

Sulfur functional groups are common motifs in bioactive molecules. Sulfonamides are most prevalent but related aza-derivatives, in which oxygen atoms are replaced by imidic nitrogens, such as sulfoximines and sulfonimidamides, are gaining attraction. Despite this activity, the double aza-variants of sulfonamides, termed sulfondiimidamides, are almost completely absent from the literature. The reason for this is poor synthetic accessibility. Although a recent synthesis has established sulfondiimidamides as viable motifs, the length of the route and the capricious nature of the key sulfondiimidoyl fluoride intermediates mean that direct application to discovery chemistry is challenging. Herein, we describe a two-step synthesis of sulfondiimidamides, exploiting a hypervalent iodine-mediated amination as the key step. The starting materials are organometallic reagents, an unsymmetrical sulfurdiimide, and amines. The method allowed >40 examples to be prepared, including derivatives of three sulfonamide-based drugs. The operational simplicity, broad scope, and concise nature make this route attractive for discovery chemistry applications.

## 1 Introduction

Sulfur functional groups, most prominently sulfonamides, have made a tremendous impact on pharmaceuticals,^1^ with the first “sulfa-drugs”, the sulfonamide-based antibiotics that preceded the penicillins, being developed 90 years ago.^2^ Nearly a century later, almost 10% of FDA-approved drugs feature a sulfur functional group.^3^ The last decade has seen the emergence of sulfonimidamides, the mono-aza variants of sulfonamides,^4^ as a valuable addition to the sulfur-based functionalities used in the design of medicinal agents,^5^ and molecules featuring this group have been developed across a broad range of disease areas.^6^

**Figure 1:**
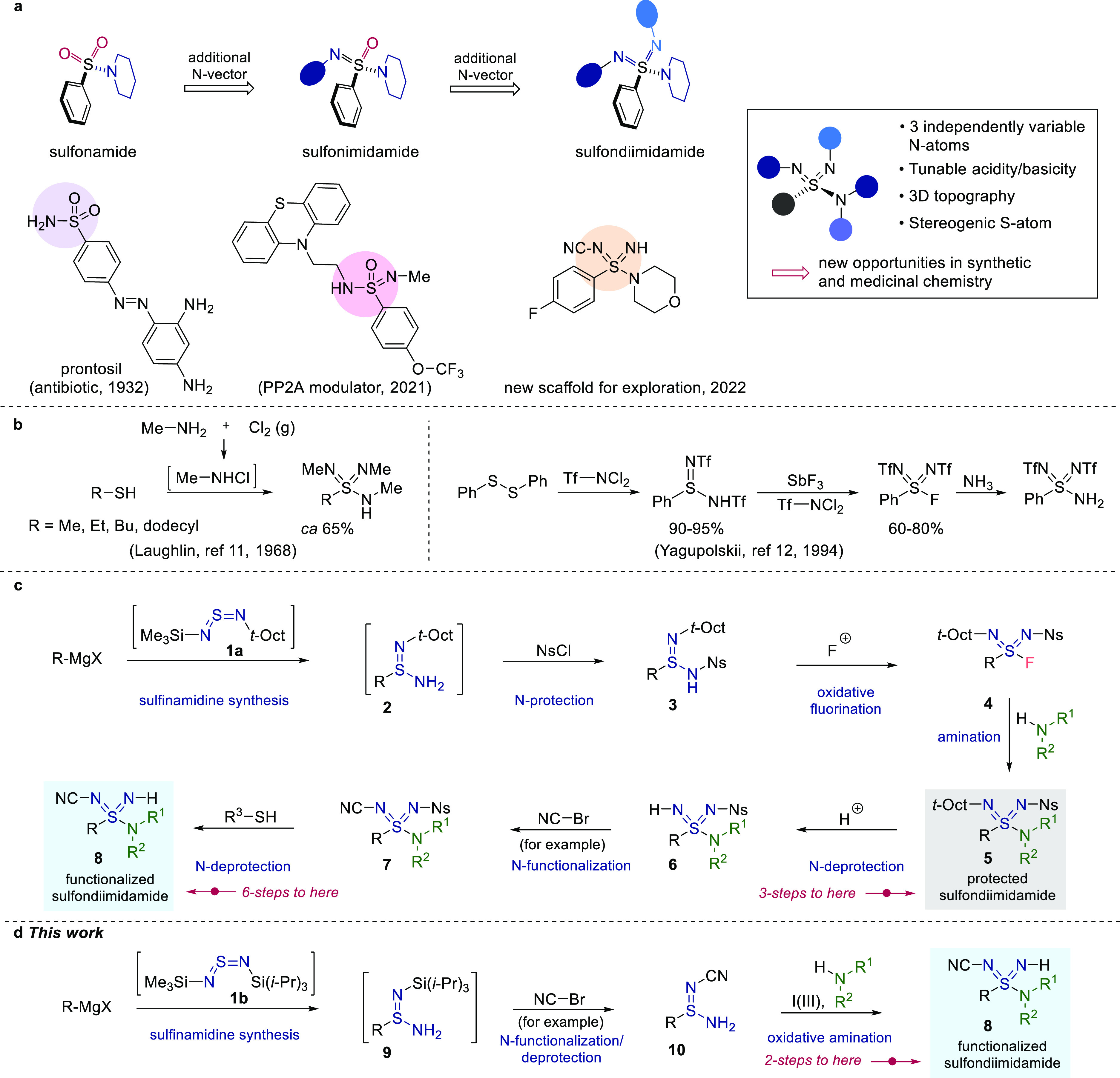
(a) Sulfonamides, sulfonimidamides, and sulfondiimidamides as functional groups in medicinal chemistry. (b) Synthesis of sulfondiimidamides from Laughlin and Yagupolskii. (c) Synthesis of sulfondiimidamides via sulfondiimidoyl fluorides 4. (d) *This work:* the synthesis of sulfondiimidamides exploiting I(III)-mediated amination.

Sulfondiimidamides, the double aza-variants of sulfonamides, provide an exciting platform for the design of new bioactive molecules.^7^ Building on many of the favorable attributes of sulfonamides, the addition of two imidic N-groups presents multiple opportunities to manipulate their properties (Figure 1a). For example, variation of the two N-substituents should allow for precise control of the acid/base nature, and the closely related H-bonding donor and acceptor capabilities, of these groups. Additionally, the two imidic N-substituents attached to a tetrahedral sulfur-center provide opportunities to modulate physiochemical properties,^8^ as well as additional vectors to explore regions of chemical space,^9^ which can be crucial to the fine-tuning of structure–activity relationships. When appropriately decorated, the tetrahedral sulfur center renders the molecules chiral, providing a further opportunity to engineer for selectivity with a biological binding site.^10^ The reason that these benefits have not been exploited by medicinal chemists is that until very recently,^7^ synthetic routes to sulfondiimidamides were essentially non-existent, as were data regarding the functionalization and stability of these intriguing molecules.

Laughlin reported the first synthesis of a sulfondiimidamide in 1968 with a route requiring treatment of an alkyl thiol with an excess of the hazardous reagent methylchloramine;^11^ a small number of fully methylated sulfondiimidamides were prepared in this way (Figure 1b). A later report from Yagupolskii, recently modified by List,^9b^ achieved the preparation of ditriflyl derivatives^12^ but again relied on the use of unattractive reagents (Figure 1b). Neither of these routes are amenable to discovery chemistry, in which the preparation of a diverse collection of molecules using simple, reliable procedures is needed. To address these shortcomings we recently reported a route to sulfondiimidamides starting from organometallic reagents and proceeding through key sulfondiimidoyl fluoride intermediates.^7^ The route is summarized in Figure 1c, and involves addition of organometallic reagents to an unsymmetrical sulfurdiimide (**1a**), followed by N-nosyl-protection, providing sulfinamidines **3**. Oxidative fluorination then delivers the key sulfondiimidoyl fluorides (**4**). Calcium triflimide-mediated amination of these fluorides forms the final S–N bond and achieves the fully protected sulfondiimidamide core (**5**).^13^ With the basic structure established, the two imidic substituents could be manipulated, commencing with removal of the N-*t*-octyl substituent to allow for installation of a range of terminal functional groups;^14^ an N–CN example is shown (**5** → **6** → **7**).^15^ Removal of the imidic Ns-substituent delivers the final sulfondiimidamide **8**. Using this approach we were able to prepare a diverse range of sulfondiimidamides with reasonable variation at all positions. We also established the general viability of sulfondiimidamides as a useful functional group in synthesis as we were able to achieve a variety of synthetic manipulations around the core structure, obtaining stable, isolable products.

Despite these successes, there remain some limitations that will limit the uptake of this chemistry. Most significant among these is the overall length of the sequence, with six steps needed before sulfondiimidamides substituted with attractive medicinal chemistry-like groups are achieved. In addition, the oxidative fluorination transformation was not compatible with many heterocyclic carbon-substituents, thus limiting the scope with regard to the range of C-nucleophiles that could be used. The same fluorination step is slow and can take several days to reach completion. In addition, conversion of the sulfondiimidoyl fluorides into the protected sulfondiimidamides is mediated by stoichiometric quantities of the costly Lewis acid Ca(NTf_2_)_2_ and is inefficient with electron-rich examples. Finally, strong acidic conditions are needed to remove the imidic *t*-octyl-protecting group. To deliver a synthesis of sulfondiimidamides that would be attractive to discovery chemists, we targeted a route that would deliver final products, that is, molecules already substituted with desirable terminal groups, in far fewer steps than our prior chemistry. We also wanted to expand the scope of carbon-substituents that could be employed and to avoid the use of strong acids and bases and high temperatures. In this article, we describe the successful realization of these goals and validate these claims by the preparation of diverse sulfondiimidamides, as well as sulfondiimidamide derivatives of three medicinal agents.

## 2 Results and Discussion

The preparation and onward reactivity of sulfondiimidoyl fluorides (**4**) were responsible for many of the challenges associated with our prior route to sulfondiimidamides. To avoid the issues associated with these intermediates, and to achieve a shorter overall sequence, we planned to generate and exploit a reactive sulfur(VI)-intermediate in situ. Taking inspiration from a recent synthesis of sulfonimidamides,^16^ we proposed an I(III)-mediated oxidative amination of suitably protected primary sulfinamidines as the key step in our new route (Figure 1d). Related I(III) chemistry has been described using tertiary sulfenamides and tertiary sulfinamides as substrates, leading to sulfonimidamides^17^ and sulfonimidates^18^ as products, depending on the reaction conditions employed. The use of sulfinamidines with I(III) reagents is unknown. To avoid steps associated with protecting group manipulations, we proposed installing the terminal N-substituents early in the reaction sequence and using the resultant N-functionalized sulfinamidines in the key I(III)-transformation. Finally, in order to achieve maximum functional group tolerance, we would exclude the *t*-octyl-decorated sulfurdiimide reagent in favor of an unsymmetrical *bis*(silyl)sulfurdiimide (**1b**).^16,19^ Together, these innovations should provide a shorter route that is amenable to greater diversification than the earlier chemistry. The concise nature of this proposed route is clear if we consider the preparation of functionalized sulfondiimidamide **8**, in which the imidic CN-substituent has been selected as a representative terminal functional group. Using our earlier synthetic route, six steps are needed to prepare sulfondiimidamide **8**; the proposed route would provide the same molecule in only two steps (compare Figure 1c,d).

### 2.1 Primary Sulfinamidine Synthesis

The substrates for the key I(III)-mediated amination are primary sulfinamidines **10**. These were readily prepared from the addition of organometallic reagents to sulfurdiimide **1b**, which was generated in situ from the commercially available sulfinylamine TIPS-NSO;^20^ N-functionalization and cleavage of the N-Si(*i*-Pr)_3_ substituent followed. The latter two transformations were carried out directly on the initial adducts **9** after an aqueous work-up, and the desired primary sulfinamidines (**10**) are the first intermediates that are isolated and purified (Table 1). A N-nosyl substituent was selected for initial investigation, which allowed for the scope of the carbon fragment to be explored; substituted aryl (**10a**–**c**), heteroaryl (**10d**–**h**), primary, secondary, and tertiary alkyl (**10i**–**l**), benzylic (**10m**), and alkenyl (**10n**) groups were all introduced. The aryl fragment of the COX-2 inhibitor celecoxib was also used, providing the corresponding sulfinamidine in good yield (**10o**). Alternatives to the nosyl group were readily incorporated, with cyano (**10p**), acyl (**10q**, **r**), carbamate (**10s**), and sulfonyl (**10t**, **u**) imidic N-substituents all smoothly prepared.

**Table 1:**
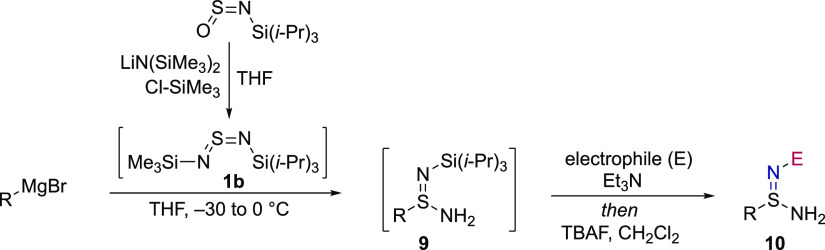

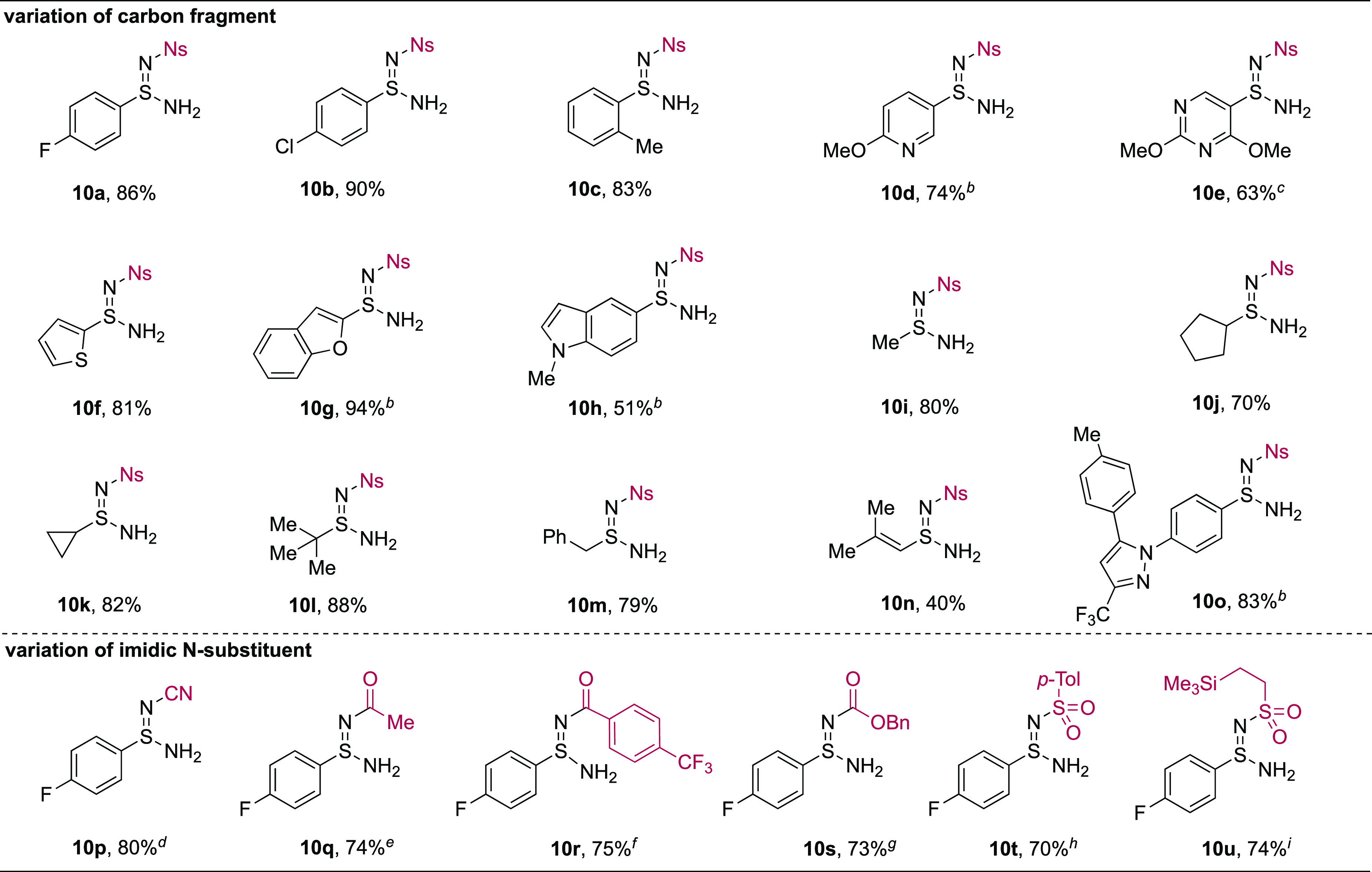
Synthesis of Primary Sulfinamidines 10 Using Sulfurdiimide Reagent **1b**^a^^b^^c^^d^^e^^f^^g^^h^^i^

a Reaction conditions: tri-isopropylsilyl sulfinylamine (1.0 equiv), LiHMDS (1.0 equiv), THF (0.5 M), −30 °C, 5 min, then 0 °C, 5 min, then TMSCl (1.0 equiv), 0 °C, 10 min, then RMgBr (1.2 equiv), 0 °C, 10 min. Aqueous work-up. Then Et_3_N (1.2 equiv), NsCl (1.0 equiv), CH_2_Cl_2_ (0.2 M), 0 °C, 20 min, then TBAF (1.1 equiv), 0 °C, 10 min. Isolated yields.

b Organolithium reagent employed.

c “Turbo Grignard” reagent (R-MgCl·LiCl) employed.

d Br-CN used.

e Ac_2_O used.

f 4-CF_3_-BzCl used.

g Cbz-Cl used.

h Ts-Cl used.

i Ses-Cl used.

### 2.2 Iodine(III)-Mediated Sulfondiimidamide Synthesis

Reaction conditions for the key oxidative amination were based on a related transformation to access sulfonimidamides.^16^ The optimized reaction conditions involved treating sulfinamidine **10a** with a slight excess of amine and using 1.5 equiv of commercial PhI(OAc)_2_ in the presence of NEt_3_, with toluene as the solvent. The reactions were performed at ambient temperature. Application of these conditions delivered N–H sulfondiimidamide **8a** in an excellent 93% yield (Table 2). This is the first report of hypervalent iodine oxidants being used with sulfinamidines, which confirms the compatibility of these useful oxidants with this aza-S(IV) functional group. Variations from these conditions are summarized in Table 2; notable is the tolerance for the alternative solvents CH_3_CN and CH_2_Cl_2_ (entries 9 and 10).

**Table 2:**
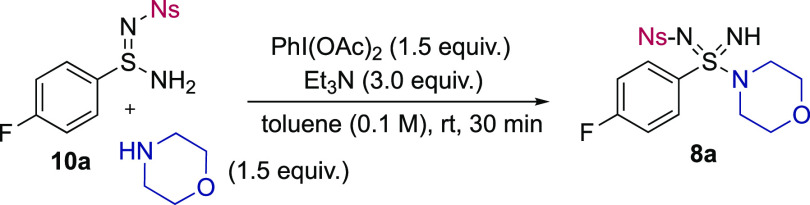
Reaction Conditions for the Conversion of Sulfinamidine **10a** into Sulfondiimidamide **8a**^a^

entry	variation from above	yield of **8a**
1	none	93%
2	no PhI(OAc)_2_	0%
3	1.0 equiv PhI(OAc)_2_	75%
4	no Et_3_N	76%
5	1.5 equiv Et_3_N	81%
6	1.0 equiv morpholine	65%
7	3.0 equiv morpholine	97%
8	DBU instead of Et_3_N	69%^b^
9	CH_3_CN instead of toluene	62%^b^
10	CH_2_Cl_2_ instead of toluene	88%^b^

a Reaction conditions: **10a** (1.0 equiv), PhI(OAc)_2_, base, morpholine, solvent (0.1 M), 30 min. Isolated yields.

b Reaction complete after 10 min.

We next applied these optimized conditions to the sulfinamidines prepared in Table 1. Morpholine was used as the amine component in this scoping study (Table 3). All of the sulfinamidines were smoothly converted to the corresponding N–H sulfondiimidamides in good to excellent yields. The range of alkyl (**8j**, **8l**, **8m**) and heteroaryl (**8e**–**8h**) derivatives prepared using this method surpasses what was possible using the previous route via sulfondiimidoyl fluorides.^7^ Amoxapine was used as the amine for the synthesis of alkenyl sulfondiimidamide **8n** due to purification issues with the morpholine derivative; **8n** is the first example of an alkenyl sulfondiimidamide to be reported. Sulfinamidines with varied N-substituents were then evaluated, and as can be seen, N-cyano (**8p**), acyl (**8q**, **r**), carbamate (**8s**), and sulfonyl (**8t**, **u**) derivatives were all converted into the corresponding N-functionalized sulfondiimidamides in excellent yields. N-cyano derivative **8p** was also prepared in our earlier report, which then required six steps; in the present study, the synthesis of sulfondiimidamide **8p** is achieved in only two steps from the starting Grignard reagent. The scope of the amine partners used in the reaction was then investigated. A wide range of cyclic secondary amines could be employed to give the corresponding sulfondiimidamides in high yields. For example, piperidines (**8v**), including examples featuring ketal (**8w**) and cyano-substituents (**8x**), were incorporated efficiently. Piperazine fragments are common in medicinal agents, and we include examples present in the pharmaceuticals buspirone (**8y**), amoxapine (**8z**), and perospirone and ziprasidone (**8aa**). Pyrrolidine (**8ab**, **ac**) and azepane (**8ad**) examples were also obtained with high yields. Sulfondiimidamide **8ac** was formed as a 1:1 mixture of diastereomers at sulfur; N-acylation allowed separation of the diastereomers and isolation of enantiomerically pure examples. Acyclic secondary amines such as *N*-benzylmethylamine (**8ae**), diethyl amine (**8af**), and diallyl amine (**8ag**) could also be included.

**Table 3:**
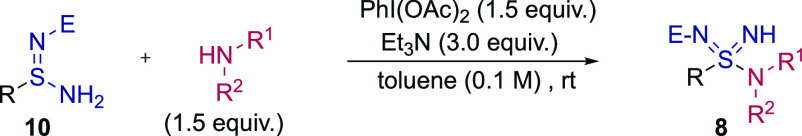

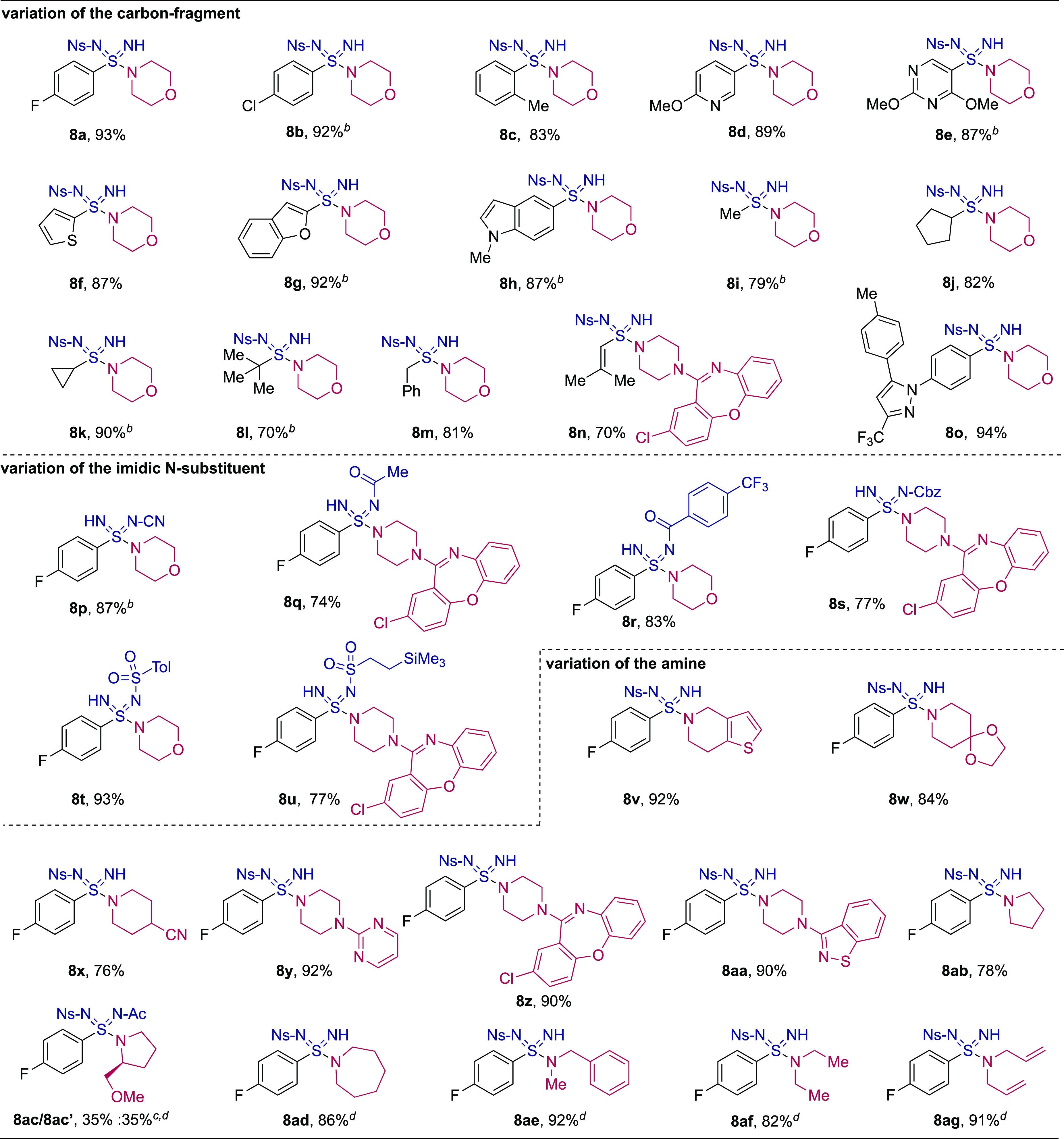
Synthesis of Sulfondiimidamides **8** Using Oxidative Amination^a^^b^^c^^d^

a Reaction conditions: sulfinamidine (1.0 equiv), PhI(OAc)_2_ (1.5 equiv), Et_3_N (3.0 equiv), amine (1.5 equiv), toluene (0.1 M). Isolated yields.

b CH_2_Cl_2_ (0.1 M) used in place of toluene.

c Followed by Ac_2_O (1.5 equiv), Et_3_N (2.0 equiv), DMAP (0.2 equiv), CH_2_Cl_2_ (0.2 M).

d Amine (3.0 equiv), toluene (0.2 M).

**Figure 2:**
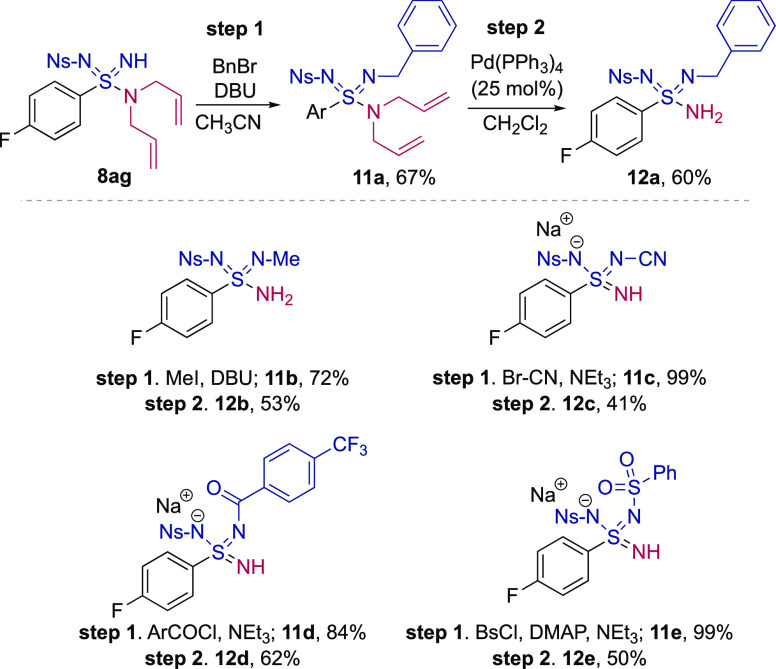
Synthesis of sulfondiimidamides via diallyl derivative **8ag**.

The I(III)-mediated amination was poorly effective for primary amines and for electron-poor nitrogen nucleophiles such as amides and anilines. Accordingly, we have developed a modified procedure that allows sulfondiimidamides derived from these types of nucleophiles to be prepared. Di-allyl N–Ns sulfondiimidamide **8ag** was reacted with benzyl bromide and DBU to form the N–Bn derivative **11a** (Figure 2). Then, removal of the two allyl groups from **11a** using catalytic Pd(0) in combination with barbituric acid^21^ provided the primary sulfondiimidamide **12a**. Sulfondiimidamide **12a** is the formal product of the I(III)-mediated amination using benzylamine. Using this approach, we prepared sulfondiimidamides formally derived from the addition of methylamine (**12b**), cyanamide (**12c**), a benzamide (**12d**), and a sulfonamide (**12e**); these are all nucleophiles incompatible with the I(III)-mediated amination. The derivatives with two strong electron-withdrawing substituents (**12c**-**e**) were isolated as their sodium salts following a basic work-up procedure.^22^

### 2.3 Applications

**Figure 3:**
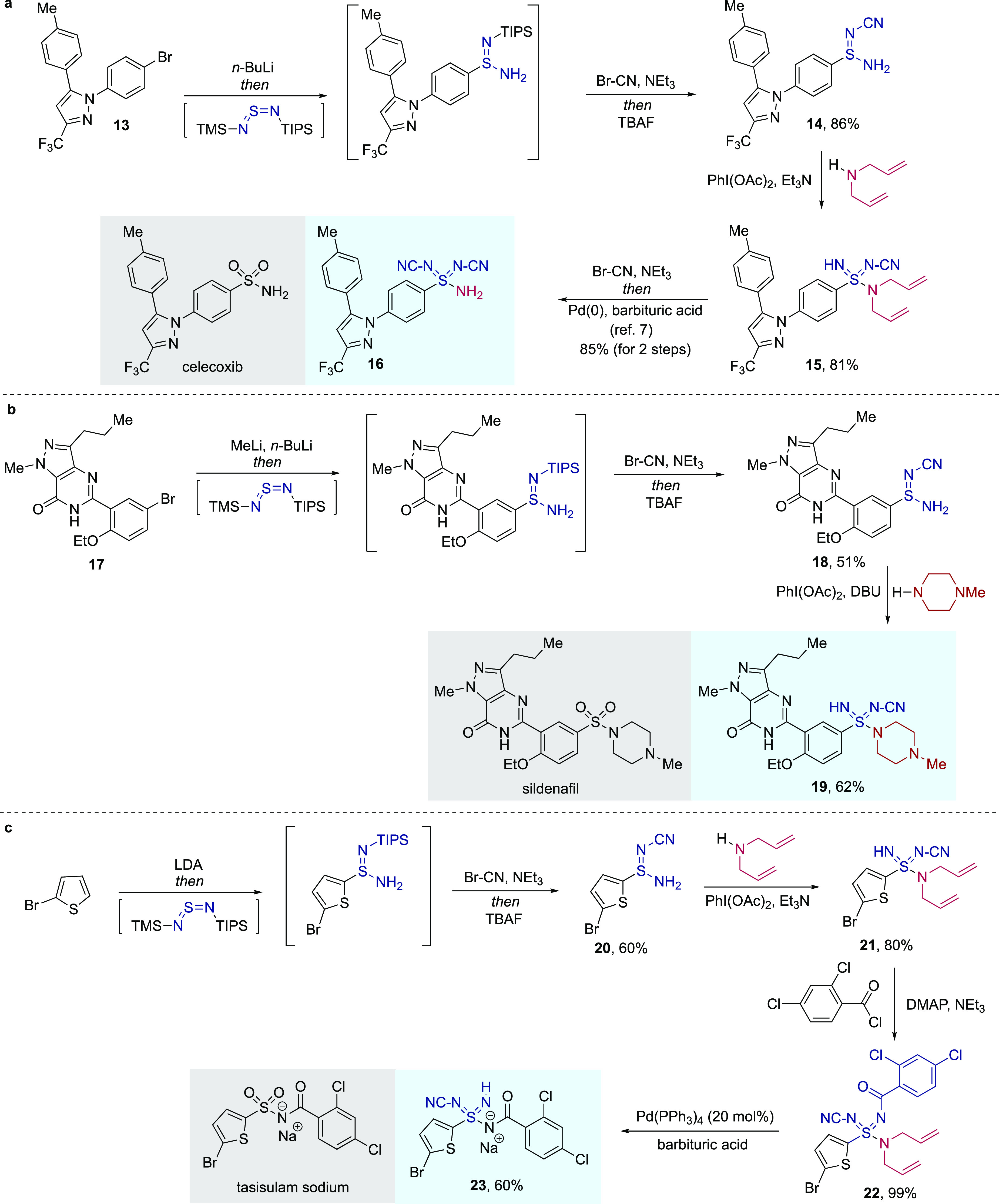
Synthesis of sulfondiimidamide derivatives of bioactive molecules. (a) Synthesis of celecoxib derivative **16**; (b) synthesis of sildenafil derivative **19**; and (c) synthesis of tasisulam sodium derivative **23**.

To highlight the key advantages of the newly developed method, in particular the short reaction sequences and excellent functional group tolerance of the chemistry, we have prepared sulfondiimidamide derivatives of three pharmaceuticals (Figure 3). In all three derivatives, we have chosen to install imidic N-cyano substituents. These are common imidic-N-substituents in various aza-sulfur functional groups,^15^ with their popularity stemming from their good metabolic stability,^23^ small size, and the presence of such a group in the marketed agrochemical Sulfoxaflor.^24^ Using our earlier sulfondiimidoyl fluoride chemistry, we had previously prepared the *bis*(N-CN)-celecoxib derivative **16** in an eight-step sequence.^7^ Here, we prepare sulfondiimidamide **16** using a four-step route starting from the aryl halide **13**. The key oxidative amination, employing primary sulfinamidine **14** and di-allylamine, delivers sulfondiimidamide **15** in 81% yield. Cyanation of the free imidic N–H followed by de-allylation (as reported previously) delivers the target structure (**16**) in an overall yield of 59%. The yield exploiting the earlier method, from the same starting material, was 24%. The second target structure prepared was the N–CN sulfondiimidamide derivative of sildenafil (**19**). Addition of in situ generated sulfurdiimide **1b** into the aryl lithium reagent derived from aryl halide **17**, followed by installation of a N–CN group and silyl deprotection, provided primary sulfinamidine **18** in 51% yield. Oxidative amination using *N*-methyl piperazine provided the target sulfondiimidamide **19** in 62% yield. The final example is a sulfondiimidamide derivative of the investigational melanoma treatment, tasisulam sodium.^25^ Starting from 2-bromothiophene, the primary sulfinamidine **20** was obtained in 60% yield. Oxidative amination using diallylamine, followed by N-benzoylation, delivered sulfondiimidamide **22**. Pd(0)-catalyzed de-allylation provided the target molecule, sulfondiimidamide sodium salt **23**.

An observation regarding the stability of sulfondiimidamides is that at least one electron-withdrawing N-substituent is needed to deliver stable products. In addition, we have measured the stability of sulfondiimidamides **8a**, **8p**, **11b**, and **16** in DMSO/buffer solutions at pH 1, 7, and 10; N–H derivatives **8a** and **8p** show slow degradation under all conditions, while fully substituted derivative **11b** and di-CN example **16** both showed excellent stability (see Supporting Information for more details).

## 3 Conclusions

In conclusion, we have demonstrated that sulfondiimidamides can be prepared in two steps, with pre-formed organometallic reagents, a sulfurdiimide reagent, and amines being the starting materials. An iodine(III)-mediated oxidative amination is the key operation and transforms primary sulfinamidines into N–H sulfondiimidamides in good yields. The reactions are broad in scope and encompass a variety of aryl, heteroaryl, alkenyl, and alkyl carbon fragments and a wide range of amines. Additionally, we demonstrate the suitability of the chemistry for the preparation of bioactive molecules by the synthesis of three sulfondiimidamide-derivatives of known medicinal agents. Taken together, we anticipate that these attributes will lend the developed method to applications in discovery chemistry.

## Supporting Information Available

Experimental procedures, spectral characterization, and additional data (PDF)
