# Supplementary material for: Modular Two-Step Route to Sulfondiimidamides
Source: J Am Chem Soc. 2022 Jun 22;144(26):11851–8. doi: 10.1021/jacs.2c04404 (PMC9264364; doi:10.1021/jacs.2c04404)
Supplement: Supplementary file 1 — ja2c04404_si_001.pdf [file ja2c04404_si_001.pdf]

A Modular Two-Step Route to Sulfondiimidamides

Ze-Xin Zhang, Charles Bell, Mingyan Ding, Michael C. Willis*

Department of Chemistry, Chemistry Research Laboratory, University of Oxford, Mansfield Road, Oxford OX1 3TA, United Kingdom.

michael.willis@chem.ox.ac.uk

Supplementary Information

Supplemental Information – Contents

1. Experimental S2

1.1. General considerations S2

1.2 Synthetic Procedures and Characterisation Data S4

1.2.1 General Procedure A for Sulfinamidine 10 Synthesis S4

1.2.2. General Procedure B for Sulfondiimidamide 8 Synthesis S24

1.2.3 The Synthesis of Sulfondiimidamide 12 S49

1.2.4 The Synthesis of a Sulfondiimidamide Analogue of Celecoxib S58

1.2.5 The Synthesis of a Sulfondiimidamide Analogue of Sildenafil S61

1.2.6 The Synthesis of a Sulfondiimidamide Analogue of Tasisulam Sodium S64

2. Aqueous Stability Study S69

3. References S71

4. NMR Spectra S72

1. Experimental

1.1. General considerations

Handling techniques: Reactions were performed under inert nitrogen atmosphere with anhydrous solvent unless otherwise stated. All glassware was oven-dried at >100 °C and allowed to cool to room temperature under a positive pressure of nitrogen. Reactions were monitored by TLC until deemed complete using aluminum backed silica plates. Plates were visualised under ultraviolet light (254 nm) and/or by staining with $KMnO_4$ solution. Cooling of reaction mixtures to 0 °C was achieved using an ice-water bath. Cooling of reaction mixtures to -78 °C was achieved using a dry ice-acetone bath. ‘Room temperature’ refers to an ambient temperature of 21 ± 2 °C.

Reagents: Reagents were purchased from Sigma Aldrich Chemical Co. Ltd., Alfa Aesar, Tokyo Chemical Industry UK or Fluorochem Ltd. and were used as supplied. Tri-*iso*propylsilyl sulfinylamine (TIPS-NSO) was prepared according to literature method.[1] Anhydrous solvents were purified by filtration through dried alumina columns using the University of Oxford internal solvent drying system (Innovative Technology Inc. PS-400-7) and sparged with nitrogen before use. All inert gases were sourced from the University of Oxford internal supplies and dried through $CaCl_2$ drying columns. Grignard reagents were titrated against salicylaldehyde phenylhydrazone.[2] Flash column chromatography was carried out using matrix 60 silica gel. ‘Petrol’ refers to the fraction of light petroleum ether boiling in the range 40-60 °C.

NMR Spectroscopy: ^{1}H-NMR spectra were obtained on a Bruker AVIII400 (400 MHz) spectrometer using the residual solvent as an internal standard. ^{13}C-NMR spectra were obtained on a Bruker AVIII400 (101 MHz) using the residual solvent as an internal standard. ^{19}F-NMR spectra were obtained on a Bruker AVIII400 (377 MHz) spectrometer. All reported ^{1}H and ^{13}C chemical shifts (δ_H, δ_C) are referenced to the residual signal of deuterated solvents ($CDCl_3$: δ_H = 7.26 ppm, δ_C = 77.16 ppm; $(CD_3)_2SO$: δ_H = 2.50 ppm, δ_C = 39.52 ppm; $(CD_3)_2CO$: δ_H = 2.05 ppm, δ_C = 206.26 ppm; CD_3CN: δ_H = 1.94 ppm, δ_C = 118.26 ppm; C_6D_6: δ_H = 7.16 ppm, δ_C = 128.06 ppm). ^{19}F chemical shifts (δ_F) are referenced externally to $CFCl_3$ (δ_F = 0.0 ppm). Chemical shifts (δ) are reported in parts per million (ppm) to the nearest 0.01 ppm for ^{1}H NMR, and 0.1 ppm for ^{13}C and ^{19}F NMR. Coupling constants (*J*) are reported in Hertz (Hz). Multiplicities are reported as followings: s (singlet), d (doublet), t (triplet), q (quartet), pent. (pentet), m (multiplet), br. (broad signal), app. (apparent).

Mass Spectroscopy: Low resolution ESI mass spectra were recorded on a Waters LCT Premier spectrometer. High resolution mass spectrometry measurements were recorded on a Bruker Daltronics MicroTOF (ESI) spectrometer or on a Micromass LCT (FI) spectrometer by the internal service at Chemistry Research Laboratory, University of Oxford. Samples for mass spectra were prepared as 1 mg/mL solution in MeOH (LRMS, HRMS-ESI) or submitted neat (HRMS-FI).

IR Spectroscopy: Infrared spectra were recorded as thin films on a Bruker Tensor 27 FT-IR spectrometer.

Melting point: Melting points were determined using a Stuart Scientific Melting Point Apparatus SMP1.

1.2 Synthetic Procedures and Characterisation Data

1.2.1 General Procedure A for Sulfinamidine 10 Synthesis

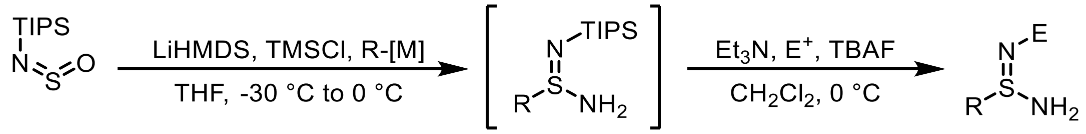

An oven-dried round-bottom flask containing TIPS-NSO (1.00 equiv.) was sealed and subjected to three N_2 evacuation/refill cycles before pre-sparged anhydrous THF (TIPS-NSO conc. 0.5 M) was added. The solution was cooled to -30 °C and then LiHMDS solution (1.00 M in THF, 1.00 equiv.) was added. The reaction was stirred at -30 °C for 5 min before being warmed to 0 °C and stirred for 5 min at the same temperature. TMSCl (1.00 equiv.) was then added and the reaction was stirred at 0 °C for 10 min. Subsequently, the corresponding organometallic reagent (1.20 equiv.) was added. The reaction was stirred at 0 °C for another 10 min before being diluted with ethyl acetate and quenched with sat. aq. tetrasodium EDTA solution. The aqueous layer was separated and extracted with ethyl acetate two times. The combined organic extracts were dried over anhydrous Na_2SO_4, filtered and concentrated under reduced pressure. This crude mixture was then dissolved in anhydrous CH_2Cl_2 (TIPS-NSO conc. 0.2 M) and cooled to 0 °C. Et_3N (1.20 equiv.) and electrophile (1.00 equiv.) were then added. The reaction was stirred at 0 °C for 20 min prior to the addition of TBAF solution (1.00 M in THF, 1.10 equiv.) at the same temperature. The reaction was stirred at 0 °C for 10 min until completion (judged by TLC). Then primary sulfinamidines **10** were isolated following different work-up procedures (**Work-up Procedure A1-A3**).

Work-up Procedure A1:

If the addition of TBAF solution to the reaction mixture resulted in precipitation of a solid, the solid was filtered and washed with a cold solution of ethyl acetate and Et_2O (1:2) to give analytically pure sulfinamidine **10**. Sat. aq. NaCl solution was added to the combined filtrate. The aqueous phase was extracted with ethyl acetate two times. The combined organic extracts were then dried over anhydrous Na_2SO_4, filtered and concentrated under reduced pressure. The resulting primary sulfinamidine **10** was then purified by flash column chromatography with the appropriate solvent system or recrystallization.

Work-up Procedure A2:

If the addition of TBAF solution to the reaction mixture did not result in the precipitation of a solid, the reaction was then diluted with ethyl acetate and quenched with sat. aq. NaCl solution. The aqueous phase was separated and extracted with ethyl acetate two times. The combined organic extracts were then dried over anhydrous Na_2SO_4, filtered and concentrated under reduced pressure to approximately $1/20^{th}$ of the original volume. At this point, a large amount of solid product precipitated out of solution, which was then separated by filtration and washed with cold Et_2O or CH_2Cl_2 to give analytically pure primary sulfinamidine **10**. The combined filtrates were then concentrated under reduced pressure and purified by flash column chromatography with the appropriate solvent system.

Work-up Procedure A3:

If the addition of TBAF solution to the reaction mixture did not result in the precipitation of a solid, the reaction was then diluted with ethyl acetate and quenched with sat. aq. NaCl solution. The aqueous phase was separated and extracted with ethyl acetate two times. The combined organic layers were then dried over anhydrous Na_2SO_4, filtered and concentrated under reduced pressure to give an oil. The primary sulfinamidine **10** was then purified by flash column chromatography with the appropriate solvent system.

Notes:

1. TIPS-protected primary sulfinamidines **9** are sensitive to moisture and heat, and may decompose slightly at room temperature. For this reason, during the work-up stage volatiles are removed using a rotary evaporator with the bath temperature set to 30 °C or lower. Additionally, crude TIPS-protected primary sulfinamidines **9** should immediately be used in the following step without further purification.
2. If the organometallic reagents were prepared at -78 °C, the *in-situ* generated sulfurdiimide reagent was added to the organometallic reagents at -78 °C. The reaction was then warmed to 0 °C and stirred at 0 °C for 10 min before being diluted with ethyl acetate and quenched with sat. aq. tetrasodium EDTA solution.

_N_-(Amino(4-fluorophenyl)-λ⁴-sulfaneylidene)-4-nitrobenzenesulfonamide (10a)

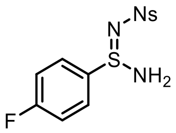

An oven-dried 100 mL round-bottom flask containing TIPS-NSO (1.10 g, 5.00 mmol, 1.00 equiv.) was sealed and subjected to three N_2 evacuation/refill cycles before pre-sparged anhydrous THF (10.0 mL) was added. The solution was cooled to -30 °C and then LiHMDS solution (5.00 mL, 1.00 M in THF, 5.00 mmol, 1.00 equiv.) was added. The reaction was stirred at -30 °C for 5 min before being warmed to 0 °C and stirred for 5 min at the same temperature. TMSCl (0.64 mL, 5.00 mmol, 1.00 equiv.) was then added and the reaction was stirred at 0 °C for 10 min. Subsequently, 4-fluorophenylmagnesium bromide solution (7.50 mL, 0.80 M in THF, 6.00 mmol, 1.20 equiv.) was added. The reaction was stirred at 0 °C for another 10 min before being diluted with ethyl acetate (80 mL) and quenched with sat. aq. tetrasodium EDTA solution (150 mL). The aqueous layer was separated and extracted with ethyl acetate (2 × 40 mL). The combined organic extracts were dried over anhydrous Na_2SO_4, filtered and concentrated under reduced pressure. This crude mixture was then dissolved in anhydrous CH_2Cl_2 (25.0 mL) in an oven-dried 100 mL round-bottom flask and cooled to 0 °C. Et_3N (0.84 mL, 6.00 mmol, 1.20 equiv.) and NsCl (1.11 g, 5.00 mmol, 1.00 equiv.) were then added. The reaction was stirred at 0 °C for 20 min prior to the addition of TBAF solution (5.50 mL, 1.00 M in THF, 5.50 mmol, 1.10 equiv.) at the same temperature. The reaction was stirred at 0 °C for 10 min until completion (judged by TLC). The reaction was then diluted with ethyl acetate (80 mL) and quenched with sat. aq. NaCl solution (150 mL). The aqueous phase was separated and extracted with ethyl acetate (2 × 40 mL). The combined organic extracts were then dried over anhydrous Na_2SO_4, filtered and concentrated under reduced pressure to approximately 1/20th of the original volume. At this point, a large amount of solid product precipitated out of solution, which was then separated by filtration and washed with cold Et_2O (10 mL) and CH_2Cl_2 (10 mL) to give analytically pure primary sulfinamidine **10a**. The combined filtrates were then concentrated under reduced pressure and purified by flash column chromatography (CH_2Cl_2/ethyl acetate, 3:1 to 1:1). Filtration and flash column chromatography afforded the sulfinamidine **10a** as a white solid (1.48 g, 4.31 mmol, 86%).

mp 145-147 °C;

R_f 0.35 (CH_2Cl_2/ethyl acetate, 3:1);

^{1}H NMR (400 MHz, $(CD_3)_2SO$): δ (ppm) = 8.34 (d, J = 8.8 Hz, 2H), 8.06 (d, J = 8.8 Hz, 2H), 7.83-7.77 (m, 2H), 7.49-7.43 (m, 2H), 7.12 (s, 2H);

^{13}C NMR (101 MHz, $(CD_3)_2SO$): δ (ppm) = 163.9 (d, J = 250.2 Hz), 150.5, 148.8, 134.8 (d, J = 2.8 Hz), 129.5 (d, J = 9.4 Hz), 127.3, 124.3, 116.6 (d, J = 22.9 Hz);

^{19}F NMR (377 MHz, $(CD_3)_2SO$): δ (ppm) = -108.9 (tt, J = 8.9, 5.1 Hz);

IR (ATR): $\tilde{\nu}$ (cm^{-1}) = 1521, 1487, 1353, 1276, 1230, 1139, 1085, 975, 835, 783, 730;

HRMS (ESI^+) calcd. for $C_{12}H_{11}FN_3O_4S_2^+$ $[M+H]^+$: 344.0170, found: 344.0171.

N-(Amino(4-chlorophenyl)-λ⁴-sulfaneylidene)-4-nitrobenzenesulfonamide (10b)

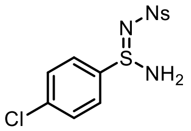

Prepared according to **General Procedure A** using TIPS-NSO (1.10 g, 5.00 mmol, 1.00 equiv.), THF (10.0 mL), LiHMDS solution (5.00 mL, 1.00 M in THF, 5.00 mmol, 1.00 equiv.), TMSCl (0.64 mL, 5.00 mmol, 1.00 equiv.), 4-chlorophenylmagnesium bromide solution (6.00 mL, 1.00 M in THF, 6.00 mmol, 1.20 equiv.), CH_2Cl_2 (25.0 mL), Et_3N (0.84 mL, 6.00 mmol, 1.20 equiv.), NsCl (1.11 g, 5.00 mmol, 1.00 equiv.) and TBAF solution (5.50 mL, 1.00 M in THF, 5.50 mmol, 1.10 equiv.).

The primary sulfinamidine **10b** was isolated and purified according to **Work-up Procedure A2**. Filtration and flash column chromatography (CH_2Cl_2/ethyl acetate, 3:1 to 1:1) afforded the desired product **10b** as a white solid (1.61 g, 4.48 mmol, 90%).

mp 174-176 °C;

R_f 0.47 (CH_2Cl_2/ethyl acetate, 2:1);

^{1}H NMR (400 MHz, $(CD_3)_2SO$): δ (ppm) = 8.34 (d, J = 8.9 Hz, 2H), 8.06 (d, J = 8.9 Hz, 2H), 7.75 (d, J = 9.0 Hz, 2H), 7.68 (d, J = 9.0 Hz, 2H), 7.16 (s, 2H);

^{13}C NMR (101 MHz, $(CD_3)_2SO$): δ (ppm) = 150.4, 148.8, 137.8, 136.8, 129.5, 128.7, 127.3, 124.3;

IR (ATR): $\tilde{\nu}$ (cm^{-1}) = 1520, 1475, 1393, 1350, 1271, 1149, 1080, 968, 832;

HRMS (ESI^+) calcd. for $C_{12}H_{11}ClN_3O_4S_2^+$ $[M+H]^+$: 359.9874, found: 359.9874.

N-(Amino(*o*-tolyl)-λ^4-sulfaneylidene)-4-nitrobenzenesulfonamide (10c)

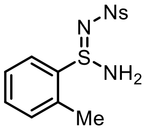

Prepared according to **General Procedure A** using TIPS-NSO (1.10 g, 5.00 mmol, 1.00 equiv.), THF (10.0 mL), LiHMDS solution (5.00 mL, 1.00 M in THF, 5.00 mmol, 1.00 equiv.), TMSCl (0.64 mL, 5.00 mmol, 1.00 equiv.), 2-methylphenylmagnesium bromide solution (7.69 mL, 0.78 M in THF, 6.00 mmol, 1.20 equiv.), CH_2Cl_2 (25.0 mL), Et_3N (0.84 mL, 6.00 mmol, 1.20 equiv.), NsCl (1.11 g, 5.00 mmol, 1.00 equiv.) and TBAF solution (5.50 mL, 1.00 M in THF, 5.50 mmol, 1.10 equiv.).

The primary sulfinamidine **10c** was isolated and purified according to **Work-up Procedure A2**. Filtration and flash column chromatography (CH_2Cl_2/ethyl acetate, 3:1 to 1:1) afforded the desired product **10c** as a white solid (1.41 g, 4.16 mmol, 83%).

mp 168-170 °C;

R*f* 0.46 (CH_2Cl_2/ethyl acetate, 2:1);

^{1}H NMR (400 MHz, $(CD_3)_2SO$): δ (ppm) = 8.31 (d, J = 8.9 Hz, 2H), 8.06-7.97 (m, 3H), 7.52-7.41 (m, 2H), 7.34 (ddd, J = 7.1, 1.9, 1.0 Hz, 1H), 6.90 (s, 2H), 2.39 (s, 3H);

^{13}C NMR (101 MHz, $(CD_3)_2SO$): δ (ppm) = 150.7, 148.7, 137.6, 135.9, 131.9, 131.3, 127.2, 127.0, 125.0, 124.2, 18.5;

IR (ATR): $\tilde{\nu}$ (cm^{-1}) = 1530, 1474, 1382, 1351, 1267, 1152, 1084, 967;

HRMS (ESI$^+$) calcd. for $C_{13}H_{14}N_3O_4S_2^+$ [M+H]$^+$: 340.0425, found: 340.0421.

N-(Amino(6-methoxypyridin-3-yl)-λ^4-sulfaneylidene)-4-nitrobenzenesulfonamide (10d)

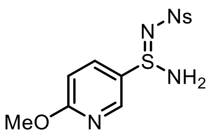

Preparation of organometallic reagent

(6-Methoxypyridin-3-yl)lithium solution was prepared according to the following procedure. To a solution of 5-bromo-2-methoxypyridine (1.13 g, 6.00 mmol, 1.20 equiv.) in anhydrous THF (12.0 mL) in an oven-dried 100 mL round-bottom flask was added *n*-butyllithium solution (2.40 mL, 2.50 M in

hexanes, 6.00 mmol, 1.20 equiv.) dropwise at -78 °C under a nitrogen atmosphere. The reaction was stirred at the same temperature for 40 min.

Preparation of sulfinamidine

Prepared according to **General Procedure A** using TIPS-NSO (1.10 g, 5.00 mmol, 1.00 equiv.), THF (10.0 mL), LiHMDS solution (5.00 mL, 1.00 M in THF, 5.00 mmol, 1.00 equiv.), TMSCl (0.64 mL, 5.00 mmol, 1.00 equiv.), (6-methoxypyridin-3-yl)lithium solution (6.00 mmol, 1.20 equiv.), CH_2Cl_2 (25.0 mL), Et_3N (0.84 mL, 6.00 mmol, 1.20 equiv.), NsCl (1.11 g, 5.00 mmol, 1.00 equiv.) and TBAF solution (5.50 mL, 1.00 M in THF, 5.50 mmol, 1.10 equiv.).

The primary sulfinamidine **10d** was isolated and purified according to **Work-up Procedure A2**. Filtration and flash column chromatography (CH_2Cl_2/ethyl acetate, 2:1 to 1:1) afforded the desired product **10d** as a white solid (1.31 g, 3.68 mmol, 74%).

mp 158-160 °C;

R*f* 0.27 (CH_2Cl_2/ethyl acetate, 2:1);

^{1}H NMR (400 MHz, $(CD_3)_2SO$): δ (ppm) = 8.44 (dd, J = 2.7, 0.7 Hz, 1H), 8.33 (d, J = 9.0 Hz, 2H), 8.05 (d, J = 9.0 Hz, 2H), 7.98 (dd, J = 8.9, 2.7 Hz, 1H), 7.15 (s, 2H), 7.02 (dd, J = 8.9, 0.7 Hz, 1H), 3.90 (s, 3H);

^{13}C NMR (101 MHz, $(CD_3)_2SO$): δ (ppm) = 165.5, 150.5, 148.8, 146.1, 137.6, 128.1, 127.2, 124.3, 111.5, 54.1;

IR (ATR): $\tilde{\nu}$ (cm^{-1}) = 1587, 1518, 1475, 1380, 1353, 1280, 1145, 1085, 966, 856, 831, 785, 731, 685;

HRMS (ESI$^+$) calcd. for $C_{12}H_{13}N_4O_5S_2^+$ [M+H]$^+$: 357.0322, found: 357.0323.

N-(Amino(2,4-dimethoxypyrimidin-5-yl)-λ^4-sulfaneylidene)-4-nitrobenzenesulfonamide (10e)

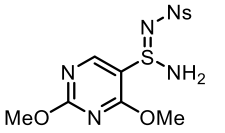

Preparation of Grignard reagent

(2,4-Dimethoxypyrimidin-5-yl)magnesium chloride lithium chloride complex solution was prepared according to the following procedure. To a solution of 5-bromo-2,4-dimethoxypyrimidine (1.31 g, 6.00 mmol, 1.20 equiv.) in anhydrous THF (3.0 mL) in an oven-dried 50 mL round-bottom flask was

added *iso*-propylmagnesium chloride lithium chloride complex solution (Turbo Grignard Reagent, 4.80 mL, 1.25 M in THF, 6.00 mmol, 1.20 equiv.) dropwise at 0 °C under a nitrogen atmosphere. The reaction was stirred at the same temperature for 1 h.

Preparation of sulfinamidine

Prepared according to **General Procedure A** using TIPS-NSO (1.10 g, 5.00 mmol, 1.00 equiv.), THF (10.0 mL), LiHMDS solution (5.00 mL, 1.00 M in THF, 5.00 mmol, 1.00 equiv.), TMSCl (0.64 mL, 5.00 mmol, 1.00 equiv.), (2,4-dimethoxypyrimidin-5-yl)magnesium chloride lithium chloride complex solution (6.00 mmol, 1.20 equiv.), CH_2Cl_2 (25.0 mL), Et_3N (0.84 mL, 6.00 mmol, 1.20 equiv.), NsCl (1.11 g, 5.00 mmol, 1.00 equiv.) and TBAF solution (5.50 mL, 1.00 M in THF, 5.50 mmol, 1.10 equiv.).

The primary sulfinamidine **10e** was isolated and purified according to **Work-up Procedure A3**. Flash column chromatography (petrol/ethyl acetate, 1:2 to 1:4 to 1:10) afforded the desired product **10e** as a white solid (1.21 g, 3.13 mmol, 63%).

mp 170-172 °C;

R_f 0.60 (ethyl acetate);

^{1}H NMR (400 MHz, $(CD_3)_2SO$): δ (ppm) = 8.72 (s, 1H), 8.31 (d, J = 8.8 Hz, 2H), 7.96 (d, J = 8.8 Hz, 2H), 7.08 (s, 2H), 3.94 (s, 3H), 3.88 (s, 3H);

^{13}C NMR (101 MHz, $(CD_3)_2SO$): δ (ppm) = 166.5, 166.3, 157.5, 150.4, 148.7, 127.2, 124.1, 113.4, 55.5, 54.9;

IR (ATR): $\tilde{\nu}$ (cm^{-1}) = 2160, 1740, 1591, 1523, 1470, 1386, 1349, 1279, 1143, 1087, 949, 857, 796;

HRMS (ESI$^+$) calcd. for $C_{12}H_{14}N_5O_6S_2^+$ [M+H]$^+$: 388.0380, found: 388.0379.

N-(Amino(thiophen-2-yl)-λ^4-sulfaneylidene)-4-nitrobenzenesulfonamide (10f)

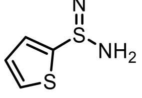

Prepared according to **General Procedure A** using TIPS-NSO (1.10 g, 5.00 mmol, 1.00 equiv.), THF (10.0 mL), LiHMDS solution (5.00 mL, 1.00 M in THF, 5.00 mmol, 1.00 equiv.), TMSCl (0.64 mL, 5.00 mmol, 1.00 equiv.), 2-thienylmagnesium bromide solution (6.00 mL, 1.00 M in THF, 6.00 mmol,

1.20 equiv.), CH_2Cl_2 (25.0 mL), Et_3N (0.84 mL, 6.00 mmol, 1.20 equiv.), NsCl (1.11 g, 5.00 mmol, 1.00 equiv.) and TBAF solution (5.50 mL, 1.00 M in THF, 5.50 mmol, 1.10 equiv.).

The primary sulfinamidine **10f** was isolated and purified according to **Work-up Procedure A1**. Filtration and flash column chromatography (CH_2Cl_2/ethyl acetate, 3:1 to 1:1) afforded the desired product **10f** as a white solid (1.34 g, 4.05 mmol, 81%).

mp 162-164 °C;

R*f* 0.41 (petrol/ethyl acetate, 1:2);

^{1}H NMR (400 MHz, $(CD_3)_2SO$): δ (ppm) = 8.35 (d, J = 8.9 Hz, 2H), 8.05 (d, J = 8.9 Hz, 2H), 7.96 (dd, J = 5.1, 1.4 Hz, 1H), 7.42 (dd, J = 3.8, 1.4 Hz, 1H), 7.38 (s, 2H), 7.21 (dd, J = 5.1, 3.8 Hz, 1H);

^{13}C NMR (101 MHz, $(CD_3)_2SO$): δ (ppm) = 150.3, 148.8, 140.2, 133.9, 131.3, 128.8, 127.2, 124.3;

IR (ATR): $\tilde{\nu}$ (cm^{-1}) = 1535, 1477, 1431, 1401, 1351, 1294, 1143, 1086, 966, 850, 787, 728, 683;

HRMS (ESI$^+$) calcd. for $C_{10}H_{10}N_3O_4S_3^+$ [M+H]$^+$: 331.9828, found: 331.9828.

N-(Amino(benzofuran-2-yl)-λ^4-sulfaneylidene)-4-nitrobenzenesulfonamide (10g)

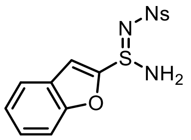

Preparation of organometallic reagent

2-Lithiobenzofuran solution was prepared according to the following procedure. To a solution of benzofuran (850 mg, 7.20 mmol, 1.20 equiv.) in anhydrous THF (14.4 mL) in an oven-dried 100 mL round-bottom flask was added *n*-butyllithium solution (2.88 mL, 2.50 M in hexanes, 7.20 mmol, 1.20 equiv.) dropwise at 0 °C under a nitrogen atmosphere. The reaction was stirred at the same temperature for 1 h.

Preparation of sulfinamidine

Prepared according to **General Procedure A** using TIPS-NSO (1.31 g, 6.00 mmol, 1.00 equiv.), THF (12.0 mL), LiHMDS solution (6.00 mL, 1.00 M in THF, 6.00 mmol, 1.00 equiv.), TMSCl (0.76 mL, 6.00 mmol, 1.00 equiv.), 2-lithiobenzofuran solution (7.20 mmol, 1.20 equiv.), CH_2Cl_2 (30.0 mL), Et_3N (1.00 mL, 7.20 mmol, 1.20 equiv.), NsCl (1.33 g, 6.00 mmol, 1.00 equiv.) and TBAF solution (6.60 mL, 1.00 M in THF, 6.60 mmol, 1.10 equiv.).

The primary sulfinamidine **10g** was isolated and purified according to **Work-up Procedure A1**. Filtration afforded the desired product **10g** as a white solid (2.05 g, 5.62 mmol, 94%).

mp 188-190 °C;

R*f* 0.50 (petrol/ethyl acetate, 1:2);

^{1}H NMR (400 MHz, $(CD_3)_2SO$): δ (ppm) = 8.32 (d, J = 8.9 Hz, 2H), 8.05 (d, J = 8.9 Hz, 2H), 7.77 (ddd, J = 7.8, 1.3, 0.7 Hz, 1H), 7.66-7.61 (m, 1H), 7.57 (s, 2H), 7.50-7.42 (m, 2H), 7.35 (ddd, J = 8.1, 7.2, 1.0 Hz, 1H);

^{13}C NMR (101 MHz, $(CD_3)_2SO$): δ (ppm) = 156.3, 150.1, 149.3, 148.8, 127.3, 127.1, 126.3, 124.23, 124.19, 122.8, 112.0, 111.9;

IR (ATR): $\tilde{\nu}$ (cm^{-1}) = 1701, 1529, 1349, 1275, 1137, 1080, 980;

HRMS (ESI$^+$) calcd. for $C_{14}H_{12}N_3O_5S_2^+$ [M+H]$^+$: 366.0213, found: 366.0213.

***N*-(Amino(1-methyl-1*H*-indol-5-yl)-λ^4-sulfaneylidene)-4-nitrobenzenesulfonamide (10h)**

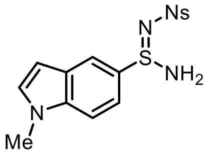

Preparation of organometallic reagent

(1-Methylindole-5-yl)lithium solution was prepared according to the following procedure. To a solution of 5-bromo-1-methylindole (1.26 g, 6.00 mmol, 1.20 equiv.) in anhydrous THF (12.0 mL) in an oven-dried 100 mL round-bottom flask was added *n*-butyllithium solution (2.40 mL, 2.50 M in hexanes, 6.00 mmol, 1.20 equiv.) dropwise at -78 °C under a nitrogen atmosphere. The reaction was stirred at the same temperature for 1 h.

Preparation of sulfinamidine

Prepared according to **General Procedure A** using TIPS-NSO (1.10 g, 5.00 mmol, 1.00 equiv.), THF (10.0 mL), LiHMDS solution (5.00 mL, 1.00 M in THF, 5.00 mmol, 1.00 equiv.), TMSCl (0.64 mL, 5.00 mmol, 1.00 equiv.), (1-methylindole-5-yl)lithium solution (6.00 mmol, 1.20 equiv.), CH_2Cl_2 (25.0 mL), Et_3N (0.84 mL, 6.00 mmol, 1.20 equiv.), NsCl (1.11 g, 5.00 mmol, 1.00 equiv.) and TBAF solution (5.50 mL, 1.00 M in THF, 5.50 mmol, 1.10 equiv.).

The primary sulfinamidine **10h** was isolated and purified according to **Work-up Procedure A1**. Filtration afforded the desired product **10h** as a yellow solid (960 mg, 2.54 mmol, 51%).

mp 162-164 °C;

R*f* 0.57 (CH_2Cl_2/ethyl acetate, 1:1);

^{1}H NMR (400 MHz, $(CD_3)_2SO$): *δ* (ppm) = 8.32 (d, *J* = 8.8 Hz, 2H), 8.05 (d, *J* = 8.8 Hz, 2H), 7.98 (d, *J* = 2.0 Hz, 1H), 7.63 (d, *J* = 8.8 Hz, 1H), 7.52-7.42 (m, 2H), 6.91 (s, 2H), 6.59 (dd, *J* = 3.1, 0.8 Hz, 1H), 3.83 (s, 3H);

^{13}C NMR (101 MHz, $(CD_3)_2SO$): *δ* (ppm) = 150.9, 148.6, 137.5, 132.1, 128.5, 127.8, 127.2, 124.2, 119.5, 118.8, 110.8, 101.6, 32.8;

IR (ATR): $\tilde{\nu}$ (cm^{-1}) = 2160, 2031, 1529, 1348, 1290, 1140, 1084, 974, 732;

HRMS (ESI$^+$) calcd. for $C_{15}H_{15}N_4O_4S_2^+$ [M+H]$^+$: 379.0529, found: 379.0526.

N-(Amino(methyl)-λ^4-sulfaneylidene)-4-nitrobenzenesulfonamide (10i)

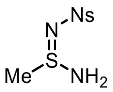

Prepared according to **General Procedure A** using TIPS-NSO (2.19 g, 10.00 mmol, 1.00 equiv.), THF (20.0 mL), LiHMDS solution (10.00 mL, 1.00 M in THF, 10.00 mmol, 1.00 equiv.), TMSCl (1.27 mL, 10.00 mmol, 1.00 equiv.), methylmagnesium bromide solution (4.00 mL, 3.00 M in THF, 12.00 mmol, 1.20 equiv.), CH_2Cl_2 (50.0 mL), Et_3N (1.67 mL, 12.00 mmol, 1.20 equiv.), NsCl (2.22 g, 10.00 mmol, 1.00 equiv.) and TBAF solution (11.00 mL, 1.00 M in THF, 11.00 mmol, 1.10 equiv.).

The primary sulfinamidine **10i** was isolated and purified according to **Work-up Procedure A1**. Filtration afforded the desired product **10i** as a white solid (2.10 g, 7.98 mmol, 80%).

mp 158-160 °C;

R*f* 0.33 (ethyl acetate);

^{1}H NMR (400 MHz, $(CD_3)_2SO$): *δ* (ppm) = 8.31 (d, *J* = 8.8 Hz, 2H), 7.99 (d, *J* = 8.8 Hz, 2H), 6.44 (s, 2H), 2.82 (s, 3H).;

^{13}C NMR (101 MHz, $(CD_3)_2SO$): *δ* (ppm) = 151.0, 148.6, 127.2, 124.2, 38.7.;

IR (ATR): $\tilde{\nu}$ (cm^{-1}) = 1517, 1381, 1355, 1285, 1141, 1087, 950;

HRMS (ESI$^+$) calcd. for $C_7H_{10}N_3O_4S_2^+$ $[M+H]^+$: 264.0107, found: 264.0107.

N-(Amino(cyclopentyl)-λ^4-sulfaneylidene)-4-nitrobenzenesulfonamide (10j)

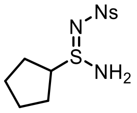

Prepared according to **General Procedure A** using TIPS-NSO (1.10 g, 5.00 mmol, 1.00 equiv.), THF (10.0 mL), LiHMDS solution (5.00 mL, 1.00 M in THF, 5.00 mmol, 1.00 equiv.), TMSCl (0.64 mL, 5.00 mmol, 1.00 equiv.), cyclopentylmagnesium bromide solution (3.11 mL, 1.93 M in Et_2O, 6.00 mmol, 1.20 equiv.), CH_2Cl_2 (25.0 mL), Et_3N (0.84 mL, 6.00 mmol, 1.20 equiv.), NsCl (1.11 g, 5.00 mmol, 1.00 equiv.) and TBAF solution (5.50 mL, 1.00 M in THF, 5.50 mmol, 1.10 equiv.).

The primary sulfinamidine **10j** was isolated and purified according to **Work-up Procedure A3**. Flash column chromatography (CH_2Cl_2/ethyl acetate, 3:1 to 1:1) afforded the desired product **10j** as a white solid (1.11 g, 3.50 mmol, 70%).

mp 154-156 °C;

R*f* 0.40 (CH_2Cl_2/ethyl acetate, 1:1);

^{1}H NMR (400 MHz, $(CD_3)_2SO$): δ (ppm) = 8.32 (d, J = 8.9 Hz, 2H), 7.98 (d, J = 8.9 Hz, 2H), 6.32 (s, 2H), 3.55 (tt, J = 8.3, 5.7 Hz, 1H), 1.92-1.75 (m, 2H), 1.74-1.60 (m, 2H), 1.60-1.46 (m, 4H);

^{13}C NMR (101 MHz, $(CD_3)_2SO$): δ (ppm) = 151.0, 148.5, 127.2, 124.1, 62.0, 27.5, 27.2, 25.0, 24.8 (Note: For 4 secondary carbons in cyclopentane ring, 4 peaks were found instead of 2 due to the loss of symmetry caused by chiral sulfur atom);

IR (ATR): $\tilde{\nu}$ (cm^{-1}) = 1538, 1382, 1350, 1291, 1143, 1085, 970, 853, 732, 683, 620;

HRMS (ESI$^+$) calcd. for $C_{11}H_{15}N_3O_4S_2Na^+$ $[M+Na]^+$: 340.0396, found: 340.0397.

N-(Amino(cyclopropyl)-λ^4-sulfaneylidene)-4-nitrobenzenesulfonamide (10k)

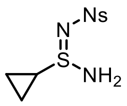

Prepared according to **General Procedure A** using TIPS-NSO (1.10 g, 5.00 mmol, 1.00 equiv.), THF (10.0 mL), LiHMDS solution (5.00 mL, 1.00 M in THF, 5.00 mmol, 1.00 equiv.), TMSCl (0.64 mL, 5.00 mmol, 1.00 equiv.), cyclopropylmagnesium bromide solution (6.00 mL, 1.00 M in THF,

6.00 mmol, 1.20 equiv.), CH_2Cl_2 (25.0 mL), Et_3N (0.84 mL, 6.00 mmol, 1.20 equiv.), NsCl (1.11 g, 5.00 mmol, 1.00 equiv.) and TBAF solution (5.50 mL, 1.00 M in THF, 5.50 mmol, 1.10 equiv.).

The primary sulfinamidine **10k** was isolated and purified according to **Work-up Procedure A2**. Filtration and flash column chromatography (CH_2Cl_2/ethyl acetate, 1:1 to 0:1) afforded the desired product **10k** as a white solid (1.19 g, 4.12 mmol, 82%).

mp 154-156°C;

R*f* 0.38 (CH_2Cl_2/ethyl acetate, 1:1);

^{1}H NMR (400 MHz, $(CD_3)_2SO$): δ (ppm) = 8.32 (d, J = 8.8 Hz, 2H), 7.97 (d, J = 8.8 Hz, 2H), 6.45 (s, 2H), 2.62 (tt, J = 7.8, 4.6 Hz, 1H), 1.11-1.01 (m, 1H), 0.96-0.83 (m, 2H), 0.73-0.61 (m, 1H);

^{13}C NMR (101 MHz, $(CD_3)_2SO$): δ (ppm) = 150.9, 148.6, 127.2, 124.2, 29.7, 3.8, 1.8 (Note: For 2 secondary carbons in cyclopropane ring, 2 peaks were found instead of 1 due to the loss of symmetry caused by chiral sulfur atom);

IR (ATR): $\tilde{\nu}$ (cm^{-1}) = 1526, 1382, 1350, 1284, 1146, 1088, 983, 855, 735, 682, 616;

HRMS (ESI^+) calcd. for $C_9H_{12}N_3O_4S_2^+$ $[M+H]^+$: 290.0264, found: 290.0266.

N-(Amino(*tert*-butyl)-λ^4-sulfaneylidene)-4-nitrobenzenesulfonamide (10l)

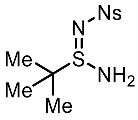

Prepared according to **General Procedure A** using TIPS-NSO (1.31 g, 6.00 mmol, 1.00 equiv.), THF (12.0 mL), LiHMDS solution (6.00 mL, 1.00 M in THF, 6.00 mmol, 1.00 equiv.), TMSCl (0.76 mL, 6.00 mmol, 1.00 equiv.), *tert*-butylmagnesium chloride solution (7.58 mL, 0.95 M in THF, 7.20 mmol, 1.20 equiv.), CH_2Cl_2 (30.0 mL), Et_3N (1.00 mL, 7.20 mmol, 1.20 equiv.), NsCl (1.33 g, 6.00 mmol, 1.00 equiv.) and TBAF solution (6.60 mL, 1.00 M in THF, 6.60 mmol, 1.10 equiv.).

The primary sulfinamidine **10l** was isolated and purified according to **Work-up Procedure A1**. Filtration and flash column chromatography (CH_2Cl_2/ethyl acetate, 3:1 to 1:1) afforded the desired product **10l** as a white solid (1.61 g, 5.28 mmol, 88%).

mp 174-176 °C;

R$_f$ 0.45 (CH_2Cl_2/ethyl acetate, 2:1);

^{1}H NMR (400 MHz, $(CD_3)_2SO$): δ (ppm) = 8.32 (d, J = 8.9 Hz, 2H), 7.98 (d, J = 8.9 Hz, 2H), 6.19 (s, 2H), 1.15 (s, 9H);

^{13}C NMR (101 MHz, $(CD_3)_2SO$): δ (ppm) = 150.8, 148.5, 127.3, 124.1, 57.0, 22.7;

IR (ATR): $\tilde{\nu}$ (cm^{-1}) = 1531, 1350, 1289, 1145, 1084, 972, 853, 733, 683, 624;

HRMS (ESI$^+$) calcd. for $C_{10}H_{15}N_3O_4S_2Na^+$ [M+Na]$^+$: 328.0396, found: 328.0397.

N-(Amino(benzyl)-λ^4-sulfaneylidene)-4-nitrobenzenesulfonamide (10m)

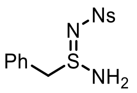

Prepared according to **General Procedure A** using TIPS-NSO (1.10 g, 5.00 mmol, 1.00 equiv.), THF (10.0 mL), LiHMDS solution (5.00 mL, 1.00 M in THF, 5.00 mmol, 1.00 equiv.), TMSCl (0.64 mL, 5.00 mmol, 1.00 equiv.), benzylmagnesium bromide solution (7.89 mL, 0.76 M in THF, 6.00 mmol, 1.20 equiv.), CH_2Cl_2 (25.0 mL), Et_3N (0.84 mL, 6.00 mmol, 1.20 equiv.), NsCl (1.11 g, 5.00 mmol, 1.00 equiv.) and TBAF solution (5.50 mL, 1.00 M in THF, 5.50 mmol, 1.10 equiv.).

The primary sulfinamidine **10m** was isolated and purified according to **Work-up Procedure A1**. Filtration and flash column chromatography (CH_2Cl_2/ethyl acetate, 2:1 to 1:2) afforded the desired product **10m** as a white solid (1.34 g, 3.95 mmol, 79%).

mp 157-159 °C;

R$_f$ 0.37 (CH_2Cl_2/ethyl acetate, 1:1);

^{1}H NMR (400 MHz, $(CD_3)_2SO$): δ (ppm) = 8.13 (d, J = 8.9 Hz, 2H), 7.68 (d, J = 8.9 Hz, 2H), 7.28-7.21 (m, 5H), 6.55 (s, 2H), 4.43 (d, J = 12.6 Hz, 1H), 4.32 (d, J = 12.6 Hz, 1H);

^{13}C NMR (101 MHz, $(CD_3)_2SO$): δ (ppm) = 150.6, 148.3, 130.9, 129.8, 128.4, 128.3, 126.8, 123.9, 57.4;

IR (ATR): $\tilde{\nu}$ (cm^{-1}) = 1526, 1381, 1354, 1283, 1139, 1087, 971, 685, 617;

HRMS (ESI$^+$) calcd. for $C_{13}H_{14}N_3O_4S_2^+$ [M+H]$^+$: 340.0420, found: 340.0421.

***N*-(Amino(2-methylprop-1-en-1-yl)-λ⁴-sulfaneylidene)-4-nitrobenzenesulfonamide (10n)**

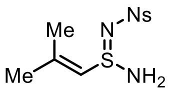

Prepared according to **General Procedure A** using TIPS-NSO (1.10 g, 5.00 mmol, 1.00 equiv.), THF (10.0 mL), LiHMDS solution (5.00 mL, 1.00 M in THF, 5.00 mmol, 1.00 equiv.), TMSCl (0.64 mL, 5.00 mmol, 1.00 equiv.), 2-methyl-1-propenylmagnesium bromide solution (13.3 mL, 0.45 M in THF, 6.00 mmol, 1.20 equiv.), CH_2Cl_2 (25.0 mL), Et_3N (0.84 mL, 6.00 mmol, 1.20 equiv.), NsCl (1.11 g, 5.00 mmol, 1.00 equiv.) and TBAF solution (5.50 mL, 1.00 M in THF, 5.50 mmol, 1.10 equiv.).

The primary sulfinamidine **10n** was isolated and purified according to **Work-up Procedure A2**. Filtration and flash column chromatography (petrol/ethyl acetate, 1:2 to 1:4) afforded the desired product **10n** as a white solid (607 mg, 2.00 mmol, 40%).

mp 145-147 °C;

R*f* 0.42 (petrol/ethyl acetate, 1:4);

^{1}H NMR (400 MHz, $(CD_3)_2SO$): δ (ppm) = 8.32 (d, J = 8.9 Hz, 2H), 7.97 (d, J = 8.9 Hz, 2H), 6.54 (s, 2H), 6.27-6.21 (m, 1H), 1.82 (d, J = 1.4 Hz, 3H), 1.78 (d, J = 1.2 Hz, 3H);

^{13}C NMR (101 MHz, $(CD_3)_2SO$): δ (ppm) = 151.1, 148.6, 146.5, 127.1, 125.1, 124.1, 24.3, 19.8;

IR (ATR): $\tilde{\nu}$ (cm^{-1}) = 1527, 1349, 1265, 1155, 1086, 982, 784, 688;

HRMS (ESI$^+$) calcd. for $C_{10}H_{14}N_3O_4S_2^+$ [M+H]$^+$: 304.0420, found: 304.0420.

***N*-(Amino(4-(5-(*p*-tolyl)-3-(trifluoromethyl)-1*H*-pyrazol-1-yl)phenyl)-λ⁴-sulfaneylidene)-4-nitrobenzenesulfonamide (10o)**

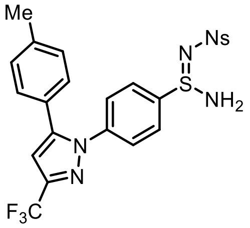

Preparation of organometallic reagent

(4-(5-(*p*-Tolyl)-3-(trifluoromethyl)-1*H*-pyrazol-1-yl)phenyl)lithium solution was prepared according to the following procedure. To a solution of 1-(4-bromophenyl)-5-(*p*-tolyl)-3-(trifluoromethyl)-1*H*-pyrazole (2.28 g, 6.00 mmol, 1.20 equiv.) in anhydrous THF (12.0 mL) in an oven-dried 100 mL round-bottom flask was added *n*-butyllithium solution (2.40 mL, 2.50 M in hexanes, 6.00 mmol,

1.20 equiv.) dropwise at -78 °C under a nitrogen atmosphere. The reaction was stirred at the same temperature for 40 min.

Preparation of sulfinamidine

Prepared according to **General Procedure A** using TIPS-NSO (1.10 g, 5.00 mmol, 1.00 equiv.), THF (10.0 mL), LiHMDS solution (5.00 mL, 1.00 M in THF, 5.00 mmol, 1.00 equiv.), TMSCl (0.64 mL, 5.00 mmol, 1.00 equiv.), (4-(5-(*p*-tolyl)-3-(trifluoromethyl)-1*H*-pyrazol-1-yl)phenyl)lithium solution (6.00 mmol, 1.20 equiv.), CH_2Cl_2 (25.0 mL), Et_3N (0.84 mL, 6.00 mmol, 1.20 equiv.), NsCl (1.11 g, 5.00 mmol, 1.00 equiv.) and TBAF solution (5.50 mL, 1.00 M in THF, 5.50 mmol, 1.10 equiv.).

The primary sulfinamidine **10o** was isolated and purified according to **Work-up Procedure A3**. Flash column chromatography (CH_2Cl_2/ethyl acetate, 5:1 to 3:1) afforded the desired product **10o** as a white solid (2.28 g, 4.15 mmol, 83%).

mp 207-209 °C;

$\mathbf{R}_f$ 0.54 (CH_2Cl_2/ethyl acetate, 3:1);

^{1}H NMR (400 MHz, $(CD_3)_2SO$): δ (ppm) = 8.34 (d, J = 8.9 Hz, 2H), 8.07 (d, J = 8.9 Hz, 2H), 7.81 (d, J = 8.8 Hz, 2H), 7.58 (d, J = 8.8 Hz, 2H), 7.25-7.14 (m, 7H), 2.31 (s, 3H);

^{13}C NMR (101 MHz, $(CD_3)_2SO$): δ (ppm) = 150.4, 148.8, 145.3, 142.4 (q, J = 37.7 Hz), 141.3, 139.1, 138.9, 129.4, 128.8, 128.0, 127.3, 126.4, 125.3, 124.3, 121.3 (q, J = 269.0 Hz), 106.2, 20.8;

^{19}F NMR (377 MHz, $(CD_3)_2SO$): δ (ppm) = -60.9 (s);

IR (ATR): $\tilde{\nu}$ (cm^{-1}) = 1527, 1474, 1381, 1351, 1279, 1237, 1141, 1085, 969, 786, 613;

HRMS (ESI$^+$) calcd. for $C_{23}H_{19}F_3N_5O_4S_2^+$ [M+H]$^+$: 550.0825, found: 550.0828.

N-(Amino(4-fluorophenyl)-λ^4-sulfaneylidene)cyanamide (10p)

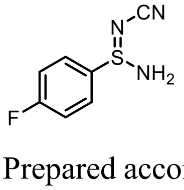

Prepared according to **General Procedure A** using TIPS-NSO (1.10 g, 5.00 mmol, 1.00 equiv.), THF (10.0 mL), LiHMDS solution (5.00 mL, 1.00 M in THF, 5.00 mmol, 1.00 equiv.), TMSCl (0.64 mL, 5.00 mmol, 1.00 equiv.), 4-fluorophenylmagnesium bromide solution (7.00 mL, 0.86 M in THF, 6.00 mmol, 1.20 equiv.), CH_2Cl_2 (25.0 mL), Et_3N (0.84 mL, 6.00 mmol, 1.20 equiv.), BrCN solution (1.00 mL, 5.0 M in CH_3CN, 5.00 mmol, 1.00 equiv.) and TBAF solution (5.50 mL, 1.00 M in THF,

5.50 mmol, 1.10 equiv.).

The primary sulfinamidine **10p** was isolated and purified according to **Work-up Procedure A3**. Flash column chromatography (CH_2Cl_2/ethyl acetate, 1:2 to 0:1) afforded the desired product **10p** as a white solid (734 mg, 4.01 mmol, 80%).

mp 92-94 °C;

R*f* 0.29 (CH_2Cl_2/ethyl acetate, 2:1);

^{1}H NMR (400 MHz, $(CD_3)_2SO$): δ (ppm) = 7.90-7.83 (m, 2H), 7.53-7.46 (m, 2H), 7.10 (s, 2H);

^{13}C NMR (101 MHz, $(CD_3)_2SO$): δ (ppm) = 164.0 (d, J = 250.3 Hz), 134.7 (d, J = 2.8 Hz), 129.7 (d, J = 9.3 Hz), 119.7, 116.6 (d, J = 22.8 Hz);

^{19}F NMR (377 MHz, $(CD_3)_2SO$): δ (ppm) = -108.7 (tt, J = 8.6, 5.2 Hz);

IR (ATR): $\tilde{\nu}$ (cm^{-1}) = 2163, 1588, 1486, 1462, 1383, 1230, 1158, 1085, 1014, 953, 793;

HRMS (ESI$^+$) calcd. for $C_7H_7FN_3S^+$ [M+H]$^+$: 184.0339, found: 184.0342.

N-(Amino(4-fluorophenyl)-λ^4-sulfaneylidene)acetamide (10q)

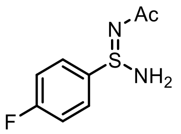

Prepared according to **General Procedure A** using TIPS-NSO (1.10 g, 5.00 mmol, 1.00 equiv.), THF (10.0 mL), LiHMDS solution (5.00 mL, 1.00 M in THF, 5.00 mmol, 1.00 equiv.), TMSCl (0.64 mL, 5.00 mmol, 1.00 equiv.), 4-fluorophenylmagnesium bromide solution (7.00 mL, 0.86 M in THF, 6.00 mmol, 1.20 equiv.), CH_2Cl_2 (25.0 mL), Et_3N (0.84 mL, 6.00 mmol, 1.20 equiv.), Ac_2O (0.47 mL, 5.00 mmol, 1.00 equiv.) and TBAF solution (5.50 mL, 1.00 M in THF, 5.50 mmol, 1.10 equiv.).

The primary sulfinamidine **10q** was isolated and purified according to **Work-up Procedure A1**. Filtration and Flash column chromatography (CH_2Cl_2/ethyl acetate, 1:3 to 1:10) afforded the desired product **10q** as a white solid (740 mg, 3.70 mmol, 74%).

mp 156-158 °C;

R*f* 0.33 (CH_2Cl_2/ethyl acetate, 1:3);

^{1}H NMR (400 MHz, $(CD_3)_2SO$): δ (ppm) = 7.89-7.83 (m, 2H), 7.45-7.38 (m, 2H), 6.28 (s, 2H), 1.91 (s, 3H);

^{13}C NMR (101 MHz, $(CD_3)_2SO$): δ (ppm) = 180.3, 163.5 (d, J = 248.7 Hz), 135.7 (d, J = 2.9 Hz), 129.5 (d, J = 9.1 Hz), 116.2 (d, J = 22.6 Hz), 25.1;

^{19}F NMR (377 MHz, $(CD_3)_2SO$): δ (ppm) = -109.8 (tt, J = 8.7, 5.3 Hz);

IR (ATR): $\tilde{\nu}$ (cm^{-1}) = 1595, 1541, 1490, 1473, 1462, 1382, 1252, 1155, 1073, 956, 836;

HRMS (ESI$^+$) calcd. for $C_8H_{10}FN_2OS^+$ [M+H]$^+$: 201.0492, found: 201.0496.

N-(Amino(4-fluorophenyl)-λ^4-sulfaneylidene)-4-(trifluoromethyl)benzamide (10r)

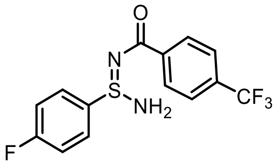

Prepared according to **General Procedure A** using TIPS-NSO (1.10 g, 5.00 mmol, 1.00 equiv.), THF (10.0 mL), LiHMDS solution (5.00 mL, 1.00 M in THF, 5.00 mmol, 1.00 equiv.), TMSCl (0.64 mL, 5.00 mmol, 1.00 equiv.), 4-fluorophenylmagnesium bromide solution (7.00 mL, 0.86 M in THF, 6.00 mmol, 1.20 equiv.), CH_2Cl_2 (25.0 mL), Et_3N (0.84 mL, 6.00 mmol, 1.20 equiv.), 4-(trifluoromethyl)benzoyl chloride (1.04 g, 5.00 mmol, 1.00 equiv.) and TBAF solution (5.50 mL, 1.00 M in THF, 5.50 mmol, 1.10 equiv.).

The primary sulfinamidine **10r** was isolated and purified according to **Work-up Procedure A1**. Filtration and flash column chromatography (petrol/ethyl acetate, 2:1 to 1:1) afforded the desired product **10r** as a white solid (1.24 g, 3.76 mmol, 75%).

mp 170-172 °C;

R*$_f$* 0.38 (petrol/ethyl acetate, 2:1);

^{1}H NMR (400 MHz, $(CD_3)_2SO$): δ (ppm) = 8.24 (d, J = 7.9 Hz, 2H), 8.03-7.96 (m, 2H), 7.78 (d, J = 7.9 Hz, 2H), 7.51-7.43 (m, 2H), 6.60 (s, 2H);

^{13}C NMR (101 MHz, $(CD_3)_2SO$): δ (ppm) = 173.3, 163.7 (d, J = 249.3 Hz), 140.9, 135.2 (d, J = 2.9 Hz), 130.7 (q, J = 31.6 Hz), 129.7 (d, J = 9.1 Hz), 129.0, 124.9 (q, J = 3.8 Hz), 124.2 (q, J = 272.2 Hz), 116.4 (d, J = 22.9 Hz);

^{19}F NMR (377 MHz, $(CD_3)_2SO$): δ (ppm) = -61.2 (s), -109.3 (tt, J = 8.7, 5.0 Hz);

IR (ATR): $\tilde{\nu}$ (cm^{-1}) = 1597, 1541, 1473, 1383, 1252, 1162, 1070, 955, 829;

HRMS (ESI$^+$) calcd. for $C_{14}H_{11}F_4N_2OS^+$ [M+H]$^+$: 331.0523, found: 331.0524.

Benzyl-(amino(4-fluorophenyl)-λ^4-sulfaneylidene)carbamate (10s)

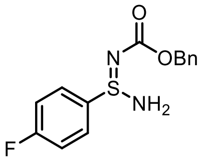

Prepared according to **General Procedure A** using TIPS-NSO (1.10 g, 5.00 mmol, 1.00 equiv.), THF (10.0 mL), LiHMDS solution (5.00 mL, 1.00 M in THF, 5.00 mmol, 1.00 equiv.), TMSCl (0.64 mL, 5.00 mmol, 1.00 equiv.), 4-fluorophenylmagnesium bromide solution (7.00 mL, 0.86 M in THF, 6.00 mmol, 1.20 equiv.), CH_2Cl_2 (25.0 mL), Et_3N (0.84 mL, 6.00 mmol, 1.20 equiv.), benzyl chloroformate (853 mg, 5.00 mmol, 1.00 equiv.) and TBAF solution (5.50 mL, 1.00 M in THF, 5.50 mmol, 1.10 equiv.).

The primary sulfinamidine **10s** was isolated and purified according to **Work-up Procedure A2**. Filtration and flash column chromatography (petrol/ethyl acetate, 1:1 to 1:3) afforded the desired product **10s** as a white solid (1.07 g, 3.66 mmol, 73%).

mp 122-124 °C;

R$_f$ 0.42 (petrol/ethyl acetate, 1:2);

^{1}H NMR (400 MHz, $(CD_3)_2SO$): δ (ppm) = 7.90-7.84 (m, 2H), 7.46-7.39 (m, 2H), 7.37-7.26 (m, 5H), 6.50 (s, 2H), 5.06 (d, J = 12.6 Hz, 1H), 5.01 (d, J = 12.6 Hz, 1H);

^{13}C NMR (101 MHz, $(CD_3)_2SO$): δ (ppm) = 163.6 (d, J = 249.1 Hz), 163.4, 138.0, 135.7 (d, J = 2.8 Hz), 129.6 (d, J = 9.1 Hz), 128.3, 127.6, 127.5, 116.2 (d, J = 22.6 Hz), 66.1;

^{19}F NMR (377 MHz, $(CD_3)_2SO$): δ (ppm) = -109.8 (tt, J = 8.6, 5.1 Hz);

IR (ATR): $\tilde{\nu}$ (cm^{-1}) = 1624, 1491, 1461, 1382, 1252, 1156, 1086, 955, 831;

HRMS (ESI$^+$) calcd. for $C_{14}H_{14}FN_2O_2S^+$ [M+H]$^+$: 293.0755, found: 293.0756.

***N*-(Amino(4-fluorophenyl)-λ^4-sulfaneylidene)-4-methylbenzenesulfonamide (10t)**

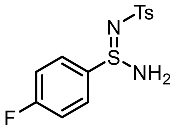

Prepared according to **General Procedure A** using TIPS-NSO (1.31 g, 6.00 mmol, 1.00 equiv.), THF

(12.0 mL), LiHMDS solution (6.00 mL, 1.00 M in THF, 6.00 mmol, 1.00 equiv.), TMSCl (0.76 mL, 6.00 mmol, 1.00 equiv.), 4-fluorophenylmagnesium bromide solution (3.60 mL, 2.00 M in THF, 7.20 mmol, 1.20 equiv.), CH_2Cl_2 (30.0 mL), Et_3N (1.00 mL, 7.20 mmol, 1.20 equiv.), TsCl (1.14 g, 6.00 mmol, 1.00 equiv.) and TBAF solution (6.60 mL, 1.00 M in THF, 6.60 mmol, 1.10 equiv.).

The primary sulfinamidine **10t** was isolated and purified according to **Work-up Procedure A2**. Filtration and flash column chromatography (petrol/ethyl acetate, 1:1 to 1:2) afforded the desired product **10t** as a white solid (1.31 g, 4.20 mmol, 70%).

mp 158-160°C;

R*f* 0.47 (petrol/ethyl acetate, 1:2);

^{1}H NMR (400 MHz, $(CD_3)_2SO$): δ (ppm) = 7.80-7.74 (m, 2H), 7.69 (d, J = 8.2 Hz, 2H), 7.48-7.41 (m, 2H), 7.30 (d, J = 8.2 Hz, 2H), 6.96 (s, 2H), 2.34 (s, 3H);

^{13}C NMR (101 MHz, $(CD_3)_2SO$): δ (ppm) = 163.8 (d, J = 249.8 Hz), 142.3, 141.0, 135.4 (d, J = 2.8 Hz), 129.5 (d, J = 9.4 Hz), 129.2, 125.7, 116.4 (d, J = 23.0 Hz), 20.9;

^{19}F NMR (377 MHz, $(CD_3)_2SO$): δ (ppm) = -109.4 (tt, J = 8.7, 5.2 Hz);

IR (ATR): $\tilde{\nu}$ (cm^{-1}) = 1596, 1487, 1462, 1383, 1252, 1145, 1086, 967, 814;

HRMS (ESI^{+}) calcd. for $C_{13}H_{14}FN_2O_2S_2^{+}$ $[M+H]^{+}$: 313.0475, found: 313.0474.

***N*-(Amino(4-fluorophenyl)-λ^{4}-sulfaneylidene)-2-(trimethylsilyl)ethane-1-sulfonamide (10u)**

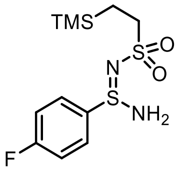

Prepared according to **General Procedure A** using TIPS-NSO (1.10 g, 5.00 mmol, 1.00 equiv.), THF (10.0 mL), LiHMDS solution (5.00 mL, 1.00 M in THF, 5.00 mmol, 1.00 equiv.), TMSCl (0.64 mL, 5.00 mmol, 1.00 equiv.), 4-fluorophenylmagnesium bromide solution (7.00 mL, 0.86 M in THF, 6.00 mmol, 1.20 equiv.), CH_2Cl_2 (25.0 mL), Et_3N (0.84 mL, 6.00 mmol, 1.20 equiv.), 2-(trimethylsilyl)ethanesulfonyl chloride (1.00 g, 5.00 mmol, 1.00 equiv.) and TBAF solution (5.50 mL, 1.00 M in THF, 5.50 mmol, 1.10 equiv.).

The primary sulfinamidine **10u** was isolated and purified according to **Work-up Procedure A3**. Flash column chromatography (petrol/ethyl acetate, 1:1 to 1:2) afforded the desired product **10u** as a white solid (1.19 g, 3.70 mmol, 74%).

mp 126-128 °C;

R_f 0.35 (petrol/ethyl acetate, 1:1);

^{1}H NMR (400 MHz, $(CD_3)_2SO$): δ (ppm) = 7.91-7.86 (m, 2H), 7.51-7.45 (m, 2H), 6.86 (s, 2H), 2.86 (dd, J = 9.5, 8.3 Hz, 2H), 1.01-0.85 (m, 2H), 0.01 (s, 9H);

^{13}C NMR (101 MHz, $(CD_3)_2SO$): δ (ppm) = 163.8 (d, J = 249.5 Hz), 135.9 (d, J = 2.8 Hz), 129.6 (d, J = 9.2 Hz), 116.4 (d, J = 22.8 Hz), 50.8, 10.6, -1.9;

^{19}F NMR (377 MHz, $(CD_3)_2SO$): δ (ppm) = -109.6 (tt, J = 8.6, 5.3 Hz);

IR (ATR): $\tilde{\nu}$ (cm^{-1}) = 1589, 1489, 1473, 1462, 1383, 1251, 1155, 1120, 1083, 957, 831;

HRMS (ESI^+) calcd. for $C_{11}H_{20}FN_2O_2S_2Si^+$ $[M+H]^+$: 323.0714, found: 323.0714.

1.2.2. General Procedure B for Sulfondiimidamide 8 Synthesis

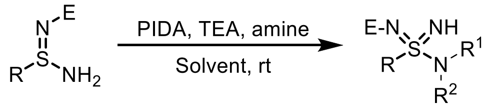

An oven-dried round-bottom flask containing primary sulfinamidine **10** (1.00 equiv.) and $PhI(OAc)_2$ (1.50 equiv.) was sealed and subjected to three N_2 evacuation/refill cycles before pre-sparged anhydrous toluene (0.1-0.2 M) or CH_2Cl_2 (0.1 M) was added. Et_3N (3.00 equiv.) was added to the solution, followed immediately by the addition of amine (1.50-3.00 equiv.). The reaction mixture was then stirred at room temperature for the specified time (judged by TLC). Once the reaction was complete as determined by TLC analysis, the reaction was diluted with ethyl acetate and quenched with sat. aq. NaCl solution. The aqueous phase was separated and extracted with ethyl acetate two times. The combined organic layers were then dried over anhydrous Na_2SO_4, filtered and concentrated under reduced pressure. The sulfondiimidamide **8** was purified by flash column chromatography with the appropriate solvent system.

Notes:

1. Since $PhI(OAc)_2$ and primary sulfinamidines **10** are usually solids, the reaction is generally complete when the reaction changes from a suspension to a clear solution. If the primary sulfinamidines **10** has been consumed by TLC analysis, it is recommended to quench the reaction immediately. Excessive reaction time will result in a slight decrease in yield.

2. For small-scale reactions, when the reaction is complete, the product can be purified by flash column chromatography directly without aqueous work-up. Analytically pure sulfondiimidamide **8** can be obtained in comparable yield. When the scale of reaction becomes larger, aqueous work-up is recommended, because a large amount of solvent will change the separation effect of flash column chromatography.

3. When the primary sulfinamidines **10** are relatively insoluble in toluene (**10b**, **10e**, **10g**, **10h**, **10i**, **10k**, **10l** and **10p**), the reaction mixture is a suspension for extended periods of time, resulting in slow reaction progression. CH_2Cl_2 is used instead of toluene to increase the solubility, thereby increasing the yield.

4. For sterically hindered amines (**8ac-ag**), toluene is still selected as the solvent due to decomposition observed when using CH_2Cl_2. Increasing the concentration of the reactants (0.2 M) and the equivalent of the amine (3.0 equiv.) can increase the yield of product.

N-((4-Fluorophenyl)(imino)(morpholino)-λ^6-sulfaneylidene)-4-nitrobenzenesulfonamide (8a)**

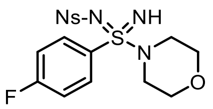

An oven-dried 25 mL round-bottom flask containing primary sulfinamidine **10a** (172 mg, 0.50 mmol, 1.00 equiv.) and $PhI(OAc)_2$ (242 mg, 0.75 mmol, 1.50 equiv.) was sealed and subjected to three N_2 evacuation/refill cycles before pre-sparged anhydrous toluene (5.0 mL) was added. Et_3N (0.21 mL, 1.50 mmol, 3.00 equiv.) was added to the solution, followed immediately by the addition of morpholine (66 µL, 0.75 mmol, 1.50 equiv.). The reaction mixture was then stirred at room temperature for 30 min until completion (judged by TLC). The reaction was diluted with ethyl acetate (60 mL) and quenched with sat. aq. NaCl solution (60 mL). The aqueous phase was then separated and extracted with ethyl acetate (2 × 30 mL). The combined organic layers were then dried over anhydrous Na_2SO_4, filtered and concentrated under reduced pressure. Purification by flash column chromatography (CH_2Cl_2/ethyl acetate, 5:1 to 3:1) afforded *sulfondiimidamide* **8a** as a white solid (200 mg, 0.47 mmol, 93%).

mp 64-66 °C;

R*f* 0.50 (petrol/ethyl acetate, 1:2);

^{1}H NMR (400 MHz, $CDCl_3$): δ (ppm) = 8.21 (d, J = 9.0 Hz, 2H), 8.06 (d, J = 9.0 Hz, 2H), 7.96-7.89 (m, 2H), 7.21-7.13 (m, 2H), 3.67 (t, J = 4.7 Hz, 4H), 3.32 (s, 1H), 3.13-2.98 (m, 4H);

^{13}C NMR (101 MHz, $CDCl_3$): δ (ppm) = 165.7 (d, J = 257.8 Hz), 149.6, 149.2, 130.3 (d, J = 9.5 Hz), 129.3 (d, J = 3.2 Hz), 127.9, 124.0, 116.6 (d, J = 22.9 Hz), 66.1, 46.7;

^{19}F NMR (377 MHz, $CDCl_3$): δ (ppm) = -103.0 (tt, J = 8.0, 4.9 Hz);

IR (ATR): $\tilde{\nu}$ (cm^{-1}) = 1528, 1351, 1300, 1154, 1089, 1067, 1052, 920;

HRMS (ESI^+) calcd. for $C_{16}H_{18}FN_4O_5S_2^+$ $[M+H]^+$: 429.0697, found: 429.0695.

Data for this compound was consistent with previous reports.[3]

***N*-((4-Chlorophenyl)(imino)(morpholino)-λ^6-sulfaneylidene)-4-nitrobenzenesulfonamide (8b)**

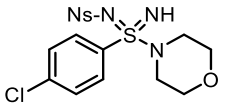

Following **General Procedure B**, sulfinamidine **10b** (179 mg, 0.50 mmol, 1.00 equiv.), $PhI(OAc)_2$

(242 mg, 0.75 mmol, 1.50 equiv.), CH_2Cl_2 (5.0 mL), Et_3N (0.21 mL, 1.50 mmol, 3.00 equiv.) and morpholine (66 µL, 0.75 mmol, 1.50 equiv.) were combined at room temperature for 15 min. Purification by flash column chromatography (CH_2Cl_2/ethyl acetate, 5:1 to 3:1) afforded *sulfondiimidamide* **8b** as a white solid (204 mg, 0.46 mmol, 92%).

mp 150-152 °C;

R*f* 0.50 (CH_2Cl_2/ethyl acetate, 3:1);

^{1}H NMR (400 MHz, $(CD_3)_2SO$): δ (ppm) = 8.31 (d, J = 8.9 Hz, 2H), 8.03 (d, J = 8.9 Hz, 2H), 7.84 (d, J = 8.8 Hz, 2H), 7.68 (d, J = 8.8 Hz, 2H), 5.50 (s, 1H), 3.60-3.49 (m, 4H), 2.97-2.83 (m, 4H);

^{13}C NMR (101 MHz, $(CD_3)_2SO$): δ (ppm) = 149.1, 149.0, 138.5, 133.7, 129.5, 129.3, 127.9, 124.1, 65.4, 45.9;

IR (ATR): $\tilde{\nu}$ (cm^{-1}) = 1532, 1383, 1260, 1157, 1083, 954, 776, 623;

HRMS (ESI^+) calcd. for $C_{16}H_{18}ClN_4O_5S_2^+$ $[M+H]^+$: 445.0402, found: 445.0402.

N-(Imino(morpholino)(*o*-tolyl)-λ^6-sulfaneylidene)-4-nitrobenzenesulfonamide (8c)

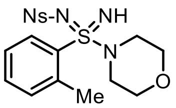

Following **General Procedure B**, sulfinamidine **10c** (170 mg, 0.50 mmol, 1.00 equiv.), $PhI(OAc)_2$ (242 mg, 0.75 mmol, 1.50 equiv.), toluene (5.0 mL), Et_3N (0.21 mL, 1.50 mmol, 3.00 equiv.) and morpholine (66 µL, 0.75 mmol, 1.50 equiv.) were combined at room temperature for 2 h. Purification by flash column chromatography (petrol/ethyl acetate, 1:2 to 1:3) afforded *sulfondiimidamide* **8c** as a colourless oil (176 mg, 0.42 mmol, 83%).

R*f* 0.54 (CH_2Cl_2/ethyl acetate, 2:1);

^{1}H NMR (400 MHz, CD_3CN): δ (ppm) = 8.16 (d, J = 8.9 Hz, 2H), 7.99 (dd, J = 8.5, 1.4 Hz, 1H), 7.95 (d, J = 8.9 Hz, 2H), 7.47 (td, J = 7.4, 1.4 Hz, 1H), 7.32-7.26 (m, 2H), 3.74-3.44 (m, 5H), 3.14-3.02 (m, 4H), 2.56 (s, 3H);

^{13}C NMR (101 MHz, CD_3CN): δ (ppm) = 150.5, 149.9, 139.5, 135.2, 134.6, 134.5, 131.2, 128.7, 127.1, 124.9, 66.6, 45.7, 20.9;

IR (ATR): $\tilde{\nu}$ (cm^{-1}) = 1526, 1382, 1350, 1297, 1259, 1151, 1069, 954, 770;

HRMS (ESI$^+$) calcd. for $C_{17}H_{21}N_4O_5S_2^+$ [M+H]$^+$: 425.0948, found: 425.0948.

N-(Imino(6-methoxypyridin-3-yl)(morpholino)-λ^6-sulfaneylidene)-4-nitrobenzenesulfonamide (8d)

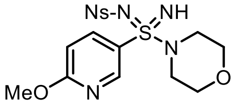

Following **General Procedure B**, sulfinamidine **10d** (178 mg, 0.50 mmol, 1.00 equiv.), $PhI(OAc)_2$ (242 mg, 0.75 mmol, 1.50 equiv.), toluene (5.0 mL), Et_3N (0.21 mL, 1.50 mmol, 3.00 equiv.) and morpholine (66 μL, 0.75 mmol, 1.50 equiv.) were combined at room temperature for 2 h. Purification by flash column chromatography (CH_2Cl_2/ethyl acetate, 3:1 to 1:1) afforded *sulfondiimidamide* **8d** as a white solid (196 mg, 0.44 mmol, 89%).

mp 152-154 °C;

R*f* 0.42 (CH_2Cl_2/ethyl acetate, 2:1);

^{1}H NMR (400 MHz, $(CD_3)_2SO$): δ (ppm) = 8.58 (dd, J = 2.7, 0.7 Hz, 1H), 8.31 (d, J = 8.9 Hz, 2H), 8.06-8.01 (m, 3H), 6.98 (dd, J = 8.9, 0.7 Hz, 1H), 6.00-5.00 (br. s, 1H), 3.92 (s, 3H), 3.61-3.51 (m, 4H), 3.00-2.89 (m, 4H);

^{13}C NMR (101 MHz, $(CD_3)_2SO$): δ (ppm) = 166.2, 149.1, 149.0, 147.8, 138.5, 128.0, 124.4, 124.1, 111.1, 65.4, 54.3, 45.8;

IR (ATR): $\tilde{\nu}$ (cm^{-1}) = 1521, 1474, 1462, 1381, 1255, 1155, 1087, 955;

HRMS (ESI$^+$) calcd. for $C_{16}H_{20}N_5O_6S_2^+$ [M+H]$^+$: 442.0850, found: 442.0851.

N-((2,4-Dimethoxypyrimidin-5-yl)(imino)(morpholino)-λ^6-sulfaneylidene)-4-nitrobenzenesulfonamide (8e)

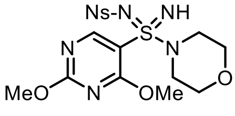

Following **General Procedure B**, sulfinamidine **10e** (194 mg, 0.50 mmol, 1.00 equiv.), $PhI(OAc)_2$ (242 mg, 0.75 mmol, 1.50 equiv.), CH_2Cl_2 (5.0 mL), Et_3N (0.21 mL, 1.50 mmol, 3.00 equiv.) and morpholine (66 μL, 0.75 mmol, 1.50 equiv.) were combined at room temperature for 15 min. Purification by flash column chromatography (CH_2Cl_2/ethyl acetate, 1:3 to 0:1) afforded

sulfondiimidamide **8e** as a white solid (205 mg, 0.43 mmol, 87%).

mp 88-90 °C;

R*f* 0.38 (ethyl acetate);

^{1}H NMR (400 MHz, CD_3CN): *δ* (ppm) = 8.74 (s, 1H), 8.21 (d, *J* = 8.4 Hz, 2H), 7.94 (d, *J* = 8.4 Hz, 2H), 3.96 (s, 3H), 3.95 (s, 3H), 3.63-3.56 (m, 4H), 3.54 (s, 1H), 3.27-3.18 (m, 4H);

^{13}C NMR (101 MHz, CD_3CN): *δ* (ppm) = 168.2, 167.8, 163.8, 150.5, 150.1, 128.8, 124.8, 112.9, 67.2, 56.5, 55.6, 46.8;

IR (ATR): $\tilde{\nu}$ (cm^{-1}) =1572, 1550, 1529, 1468, 1384, 1350, 1301, 1155, 1109, 1066;

HRMS (ESI$^+$) calcd. for $C_{16}H_{21}N_6O_7S_2^+$ [M+H]$^+$: 473.0908, found: 473.0903.

N-(Imino(morpholino)(thiophen-2-yl)-λ^6-sulfaneylidene)-4-nitrobenzenesulfonamide (8f)

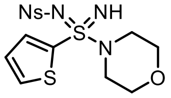

Following **General Procedure B**, sulfinamidine **10f** (165 mg, 0.50 mmol, 1.00 equiv.), $PhI(OAc)_2$ (242 mg, 0.75 mmol, 1.50 equiv.), toluene (5.0 mL), Et_3N (0.21 mL, 1.50 mmol, 3.00 equiv.) and morpholine (66 µL, 0.75 mmol, 1.50 equiv.) were combined at room temperature for 1 h. Purification by flash column chromatography (petrol/ethyl acetate, 1:2 to 1:5) afforded *sulfondiimidamide* **8f** as a white solid (180 mg, 0.43 mmol, 87%).

mp 146-148 °C;

R*f* 0.44 (petrol/ethyl acetate, 1:3);

^{1}H NMR (400 MHz, $(CD_3)_2SO$): *δ* (ppm) = 8.33 (d, *J* = 8.9 Hz, 2H), 8.06 (d, *J* = 8.9 Hz, 2H), 8.01 (dd, *J* = 5.1, 1.3 Hz, 1H), 7.56 (dd, *J* = 3.9, 1.3 Hz, 1H), 7.20 (dd, *J* = 5.1, 3.9 Hz, 1H), 5.54 (s, 1H), 3.62-3.51 (m, 4H), 2.94 (t, *J* = 4.8 Hz, 4H);

^{13}C NMR (101 MHz, $(CD_3)_2SO$): *δ* (ppm) = 149.2, 149.0, 135.2, 135.0, 133.4, 128.4, 128.0, 124.2, 65.3, 46.2;

IR (ATR): $\tilde{\nu}$ (cm^{-1}) = 1531, 1397, 1352, 1321, 1260, 1153, 1069, 955, 915, 700;

HRMS (ESI$^+$) calcd. for $C_{14}H_{16}N_4O_5S_3Na^+$ [M+Na]$^+$: 439.0175, found: 439.0174.

***N*-(Benzofuran-2-yl(imino)(morpholino)-λ[6]-sulfaneylidene)-4-nitrobenzenesulfonamide (8g)**

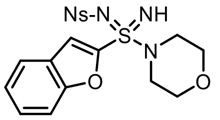

Following **General Procedure B**, sulfinamidine **10g** (183 mg, 0.50 mmol, 1.00 equiv.), $PhI(OAc)_2$ (242 mg, 0.75 mmol, 1.50 equiv.), CH_2Cl_2 (5.0 mL), Et_3N (0.21 mL, 1.50 mmol, 3.00 equiv.) and morpholine (66 µL, 0.75 mmol, 1.50 equiv.) were combined at room temperature for 20 min. Purification by flash column chromatography (CH_2Cl_2/ethyl acetate, 7:1 to 3:1) afforded *sulfondiimidamide* **8g** as a white solid (208 mg, 0.46 mmol, 92%).

mp 166-168 °C;

R_f 0.56 (CH_2Cl_2/ethyl acetate, 4:1);

^{1}H NMR (400 MHz, $(CD_3)_2SO$): δ (ppm) = 8.18 (d, J = 8.9 Hz, 2H), 8.00 (d, J = 8.9 Hz, 2H), 7.79-7.72 (m, 1H), 7.62-7.56 (m, 2H), 7.47 (ddd, J = 8.2, 7.1, 1.3 Hz, 1H), 7.38-7.32 (m, 1H), 5.98 (br. s, 1H), 3.66-3.56 (m, 4H), 3.24 (ddd, J = 12.2, 5.5, 3.8 Hz, 2H), 3.17 (ddd, J = 12.0, 5.7, 3.8 Hz, 2H).

^{13}C NMR (101 MHz, $(CD_3)_2SO$): δ (ppm) = 155.4, 149.0, 148.3, 146.9, 127.94, 127.91, 125.5, 124.4, 123.9, 123.2, 115.4, 112.1, 65.6, 46.0.

IR (ATR): $\tilde{\nu}$ (cm^{-1}) = 1528, 1350, 1303, 1159, 1085, 1069, 1052, 926;

HRMS (ESI$^+$) calcd. for $C_{18}H_{19}N_4O_6S_2^+$ [M+H]$^+$: 451.0741, found: 451.0740.

***N*-(Imino(1-methyl-1*H*-indol-5-yl)(morpholino)-λ[6]-sulfaneylidene)-4-nitrobenzenesulfonamide (8h)**

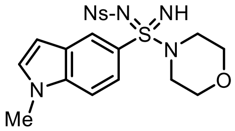

Following **General Procedure B**, sulfinamidine **10h** (189 mg, 0.50 mmol, 1.00 equiv.), $PhI(OAc)_2$ (242 mg, 0.75 mmol, 1.50 equiv.), CH_2Cl_2 (5.0 mL), Et_3N (0.21 mL, 1.50 mmol, 3.00 equiv.) and morpholine (66 µL, 0.75 mmol, 1.50 equiv.) were combined at room temperature for 10 min. Purification by flash column chromatography (petrol/ethyl acetate, 1:3 to 1:5) afforded *sulfondiimidamide* **8h** as a yellow oil (201 mg, 0.43 mmol, 87%).

R*f* 0.33 (petrol/ethyl acetate, 1:3);

^{1}H NMR (400 MHz, CD_3CN): *δ* (ppm) = 8.12 (dd, *J* = 2.0, 0.6 Hz, 1H), 8.09 (d, *J* = 8.9 Hz, 2H), 7.96 (d, *J* = 8.9 Hz, 2H), 7.64 (dd, *J* = 8.9, 2.0 Hz, 1H), 7.44 (d, *J* = 8.9 Hz, 1H), 7.28 (d, *J* = 3.2 Hz, 1H), 6.56 (dd, *J* = 3.2, 0.8 Hz, 1H), 3.78 (s, 3H), 3.58-3.47 (m, 5H), 2.97 (ddd, *J* = 11.9, 5.9, 3.6 Hz, 2H), 2.91 (ddd, *J* = 11.8, 6.0, 3.5 Hz, 2H);

^{13}C NMR (101 MHz, CD_3CN): *δ* (ppm) = 150.5, 150.3, 139.6, 133.3, 128.7, 128.6, 124.8, 124.5, 123.0, 121.0, 110.9, 103.3, 66.7, 47.2, 33.6;

IR (ATR): $\tilde{\nu}$ (cm^{-1}) = 1526, 1348, 1297, 1150, 1056, 917, 855;

HRMS (ESI$^+$) calcd. for $C_{19}H_{22}N_5O_5S_2^+$ [M+H]$^+$: 464.1057, found: 464.1057.

N-(Imino(methyl)(morpholino)-λ^6-sulfaneylidene)-4-nitrobenzenesulfonamide (8i)

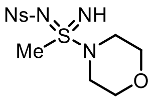

Following **General Procedure B**, sulfinamidine **10i** (132 mg, 0.50 mmol, 1.00 equiv.), $PhI(OAc)_2$ (242 mg, 0.75 mmol, 1.50 equiv.), CH_2Cl_2 (5.0 mL), Et_3N (0.21 mL, 1.50 mmol, 3.00 equiv.) and morpholine (66 µL, 0.75 mmol, 1.50 equiv.) were combined at room temperature for 15 min. Purification by flash column chromatography (ethyl acetate/MeOH, 1:0 to 25:1) afforded *sulfondiimidamide* **8i** as a white solid (138 mg, 0.40 mmol, 79%).

mp 112-114 °C;

R*f* 0.20 (ethyl acetate);

^{1}H NMR (400 MHz, $CDCl_3$): *δ* (ppm) = 8.27 (d, *J* = 8.8 Hz, 2H), 8.07 (d, *J* = 8.8 Hz, 2H), 3.70 (t, *J* = 4.7 Hz, 4H), 3.29 (dt, *J* = 11.8, 4.7 Hz, 2H), 3.23 (dt, *J* = 11.8, 4.7 Hz, 2H), 3.02 (d, *J* = 1.6 Hz, 3H), 2.92 (s, 1H);

^{13}C NMR (101 MHz, $CDCl_3$): *δ* (ppm) = 149.6, 149.3, 127.8, 124.1, 66.3, 46.9, 37.9;

IR (ATR): $\tilde{\nu}$ (cm^{-1}) = 2361, 1528, 1352, 1296, 1259, 1150, 1110, 1090, 1064, 922;

HRMS (ESI$^+$) calcd. for $C_{11}H_{17}N_4O_5S_2^+$ [M+H]$^+$: 349.0635, found: 349.0640.

N-(Cyclopentyl(imino)(morpholino)-λ^6-sulfaneylidene)-4-nitrobenzenesulfonamide (8j)

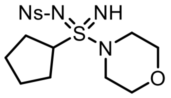

Following **General Procedure B**, sulfinamidine **10j** (159 mg, 0.50 mmol, 1.00 equiv.), $PhI(OAc)_2$ (242 mg, 0.75 mmol, 1.50 equiv.), toluene (5.0 mL), Et_3N (0.21 mL, 1.50 mmol, 3.00 equiv.) and morpholine (66 μL, 0.75 mmol, 1.50 equiv.) were combined at room temperature for 1 h. Purification by flash column chromatography (CH_2Cl_2/ethyl acetate, 3:2 to 2:3) afforded *sulfondiimidamide* **8j** as a colourless oil (165 mg, 0.41 mmol, 82%).

$\mathbf{R}_f$ 0.40 (CH_2Cl_2/ethyl acetate, 1:1);

^{1}H NMR (400 MHz, CD_3CN): δ (ppm) = 8.27 (d, J = 9.0 Hz, 2H), 8.03 (d, J = 9.0 Hz, 2H), 3.75 (dddd, J = 16.2, 8.6, 7.4, 2.2 Hz, 1H), 3.55 (dt, J = 11.6, 4.7 Hz, 2H), 3.48 (dt, J = 11.6, 4.7 Hz, 2H), 3.24 (s, 1H), 3.22 (t, J = 4.7 Hz, 4H), 1.92-1.75 (m, 4H), 1.66-1.46 (m, 4H);

^{13}C NMR (101 MHz, CD_3CN): δ (ppm) = 151.0, 150.5, 128.5, 125.0, 67.2, 64.2, 47.7, 28.8, 27.9, 26.5, 26.2 (Note: For 4 secondary carbons in cyclopentane ring, 4 peaks were found instead of 2 due to the loss of symmetry caused by chiral sulfur atom);

IR (ATR): $\tilde{\nu}$ (cm^{-1}) = 1527, 1382, 1350, 1296, 1258, 1149, 1090, 1051, 935;

HRMS (ESI$^+$) calcd. for $C_{15}H_{22}N_4O_5S_2Na^+$ [M+Na]$^+$: 425.0924, found: 425.0924

N-(Cyclopropyl(imino)(morpholino)-λ^6-sulfaneylidene)-4-nitrobenzenesulfonamide (8k)

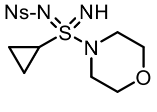

Following **General Procedure B**, sulfinamidine **10k** (145 mg, 0.50 mmol, 1.00 equiv.), $PhI(OAc)_2$ (242 mg, 0.75 mmol, 1.50 equiv.), CH_2Cl_2 (5.0 mL), Et_3N (0.21 mL, 1.50 mmol, 3.00 equiv.) and morpholine (66 μL, 0.75 mmol, 1.50 equiv.) were combined at room temperature for 15 min. Purification by flash column chromatography (ethyl acetate) afforded *sulfondiimidamide* **8k** as pale-yellow solid (168 mg, 0.45 mmol, 90%).

mp 130-132 °C;

$\mathbf{R}_f$ 0.38 (ethyl acetate);

^{1}**H NMR** (400 MHz, $(CD_3)_2SO$): δ (ppm) = 8.36 (d, J = 8.8 Hz, 2H), 8.08 (d, J = 8.8 Hz, 2H), 4.58 (s, 1H), 3.56 (ddd, J = 11.6, 6.2, 3.2 Hz, 2H), 3.49 (ddd, J = 11.6, 6.2, 3.2 Hz, 2H), 3.20 (ddd, J = 12.0, 6.2, 3.2 Hz, 2H), 3.13 (ddd, J = 12.0, 6.2, 3.2 Hz, 2H), 2.89-2.81 (m, 1H), 1.06-0.93 (m, 4H);

^{13}**C NMR** (101 MHz, $(CD_3)_2SO$): δ (ppm) = 149.7, 149.1, 127.9, 124.1, 65.6, 46.2, 29.1, 5.1, 4.7 (Note: For 2 secondary carbons in cyclopropane ring, 2 peaks were found instead of 1 due to the loss of symmetry caused by chiral sulfur atom);

IR (ATR): $\tilde{\nu}$ (cm^{-1}) = 1524, 1351, 1296, 1148, 1090, 1073, 920, 770, 609;

HRMS (ESI$^+$) calcd. for $C_{13}H_{19}N_4O_5S_2^+$ [M+H]$^+$: 375.0791, found: 375.0792.

***N*-(*tert*-Butyl(imino)(morpholino)-λ^6-sulfaneylidene)-4-nitrobenzenesulfonamide (8l)**

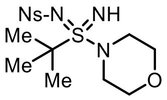

Following **General Procedure B**, sulfinamidine **10l** (153 mg, 0.50 mmol, 1.00 equiv.), $PhI(OAc)_2$ (242 mg, 0.75 mmol, 1.50 equiv.), CH_2Cl_2 (5.0 mL), Et_3N (0.21 mL, 1.50 mmol, 3.00 equiv.) and morpholine (66 μL, 0.75 mmol, 1.50 equiv.) were combined at room temperature for 20 min. Purification by flash column chromatography (petrol/ethyl acetate, 1:2 to 1:4) afforded *sulfondiimidamide* **8l** as a colourless oil (136 mg, 0.35 mmol, 70%).

R*f* 0.33 (petrol/ethyl acetate, 1:2);

^{1}**H NMR** (400 MHz, CD_3CN): δ (ppm) = 8.30 (d, J = 9.0 Hz, 2H), 8.08 (d, J = 9.0 Hz, 2H), 3.73-3.61 (m, 4H), 3.53-3.41 (m, 4H), 3.25 (s, 1H), 1.32 (s, 9H);

^{13}**C NMR** (101 MHz, CD_3CN): δ (ppm) = 151.1, 150.5, 128.4, 125.0, 67.9, 67.6, 49.8, 24.1;

IR (ATR): $\tilde{\nu}$ (cm^{-1}) = 1527, 1350, 1295, 1258, 1148, 1089, 1038, 934, 746, 610;

HRMS (ESI$^+$) calcd. for $C_{14}H_{22}N_4O_5S_2Na^+$ [M+Na]$^+$: 413.0924, found: 413.0923.

***N*-(Benzyl(imino)(morpholino)-λ^6-sulfaneylidene)-4-nitrobenzenesulfonamide (8m)**

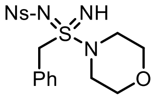

Following **General Procedure B**, sulfinamidine **10m** (170 mg, 0.50 mmol, 1.00 equiv.), $PhI(OAc)_2$

(242 mg, 0.75 mmol, 1.50 equiv.), toluene (5.0 mL), Et_3N (0.21 mL, 1.50 mmol, 3.00 equiv.) and morpholine (66 µL, 0.75 mmol, 1.50 equiv.) were combined at room temperature for 1 h. Purification by flash column chromatography (CH_2Cl_2/ethyl acetate, 3:2 to 2:3) afforded *sulfondiimidamide* **8m** as a white solid (171 mg, 0.40 mmol, 81%).

mp 102-104 °C;

$\mathbf{R}_f$ 0.44 (CH_2Cl_2/ethyl acetate, 1:1);

^{1}H NMR (400 MHz, $(CD_3)_2SO$): δ (ppm) = 8.26 (d, J = 8.9 Hz, 2H), 7.94 (d, J = 8.9 Hz, 2H), 7.44-7.30 (m, 5H), 4.68 (d, J = 13.8 Hz, 1H), 4.61 (d, J = 13.8 Hz, 1H), 4.60 (s, 1H), 3.56 (ddd, J = 11.5, 6.4, 3.0 Hz, 2H), 3.47 (ddd, J = 11.5, 6.4, 3.0 Hz, 2H), 3.23 (ddd, J = 12.4, 6.5, 3.0 Hz, 2H), 3.12 (ddd, J = 12.4, 6.5, 3.0 Hz, 2H);

^{13}C NMR (101 MHz, $(CD_3)_2SO$): δ (ppm) = 149.6, 148.9, 131.9, 128.7, 128.1, 128.0, 127.6, 123.9, 65.7, 58.3, 46.2;

IR (ATR): $\tilde{\nu}$ (cm^{-1}) = 1528, 1382, 1352, 1292, 1256, 1146, 1060, 746;

HRMS (ESI$^+$) calcd. for $C_{17}H_{21}N_4O_5S_2^+$ [M+H]$^+$: 425.0948, found: 425.0948.

***N*-((4-(2-Chlorodibenzo[*b*,*f*][1,4]oxazepin-11-yl)piperazin-1-yl)(imino)(2-methylprop-1-en-1-yl)-λ^6-sulfaneylidene)-4-nitrobenzenesulfonamide (8n)**

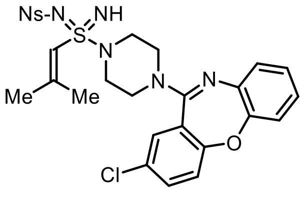

Following **General Procedure B**, sulfinamidine **10n** (152 mg, 0.50 mmol, 1.00 equiv.), $PhI(OAc)_2$ (242 mg, 0.75 mmol, 1.50 equiv.), toluene (5.0 mL), Et_3N (0.21 mL, 1.50 mmol, 3.00 equiv.) and amoxapine (235 mg, 0.75 mmol, 1.50 equiv.) were combined at room temperature for 3 h. Purification by flash column chromatography (petrol/ethyl acetate, 3:2 to 2:3) afforded *sulfondiimidamide* **8n** as a pale-yellow oil (215 mg, 0.35 mmol, 70%).

$\mathbf{R}_f$ 0.55 (petrol/ethyl acetate, 1:1);

^{1}H NMR (400 MHz, CD_3CN): δ (ppm) = 8.27 (d, J = 8.9 Hz, 2H), 8.05 (d, J = 8.9 Hz, 2H), 7.45 (dd, J = 8.7, 2.6 Hz, 1H), 7.33 (d, J = 2.6 Hz, 1H), 7.24 (d, J = 8.7 Hz, 1H), 7.14-6.98 (m, 4H), 5.96 (d,

J = 1.4 Hz, 1H), 3.60-3.38 (app. s, 4H), 3.44 (s, 1H), 3.35-3.04 (app. s, 4H), 2.07 (d, J = 1.3 Hz, 3H), 1.86 (d, J = 1.4 Hz, 3H);

^{13}C NMR (101 MHz, CD_3CN): δ (ppm) = 160.2, 159.4, 159.0, 152.6, 150.8, 150.6, 140.9, 134.0, 131.1, 129.9, 128.9, 127.7, 126.9, 125.8, 125.5, 125.0, 123.7, 121.2, 121.0, 47.6, 46.1, 27.2, 20.2;

IR (ATR): $\tilde{\nu}$ (cm^{-1}) =1603, 1587, 1559, 1526, 1471, 1349, 1299, 1151, 1089, 1048, 1010, 932, 855;

HRMS (ESI$^+$) calcd. for $C_{27}H_{28}ClN_6O_5S_2^+$ [M+H]$^+$: 615.1246, found: 615.1241.

_N_-(Imino(morpholino)(4-(5-(_p_-tolyl)-3-(trifluoromethyl)-1_H_-pyrazol-1-yl)phenyl)-λ^6-sulfaneylidene)-4-nitrobenzenesulfonamide (8o)

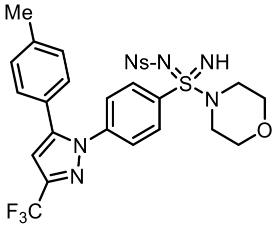

Following **General Procedure B**, sulfinamidine **10o** (275 mg, 0.50 mmol, 1.00 equiv.), $PhI(OAc)_2$ (242 mg, 0.75 mmol, 1.50 equiv.), toluene (5.0 mL), Et_3N (0.21 mL, 1.50 mmol, 3.00 equiv.) and morpholine (66 µL, 0.75 mmol, 1.50 equiv.) were combined at room temperature for 2 h. Purification by flash column chromatography (CH_2Cl_2/ethyl acetate, 10:1 to 5:1) afforded *sulfondiimidamide* **8o** as a colourless oil (297 mg, 0.47 mmol, 94%).

R$_f$ 0.43 (CH_2Cl_2/ethyl acetate, 7:1);

^{1}H NMR (400 MHz, CD_3CN): δ (ppm) = 8.23 (d, J = 8.9 Hz, 2H), 8.04 (d, J = 8.9 Hz, 2H), 7.89 (d, J = 8.8 Hz, 2H), 7.47 (d, J = 8.8 Hz, 2H), 7.17 (d, J = 8.1 Hz, 2H), 7.12 (d, J = 8.1 Hz, 2H), 6.90 (s, 1H), 3.70 (s, 1H), 3.60-3.50 (m, 4H), 3.00-2.94 (m, 4H), 2.32 (s, 3H);

^{13}C NMR (101 MHz, CD_3CN): δ (ppm) = 150.7, 150.3, 146.6, 144.2 (q, J = 38.1 Hz), 144.1, 140.8, 134.9, 130.4, 129.9, 129.6, 128.8, 126.7, 126.6, 125.1, 122.4 (q, J = 268.2 Hz), 107.2 (q, J = 1.7 Hz), 66.7, 47.4, 21.3;

^{19}F NMR (377 MHz, CD_3CN): δ (ppm) = -62.8 (s);

IR (ATR): $\tilde{\nu}$ (cm^{-1}) = 1529, 1472, 1381, 1237, 1154, 1088, 1069, 969;

HRMS (ESI$^+$) calcd. for $C_{27}H_{26}F_3N_6O_5S_2^+$ [M+H]$^+$: 635.1353, found: 635.1351.

N-((4-Fluorophenyl)(imino)(morpholino)-λ^6-sulfaneylidene)cyanamide (8p)

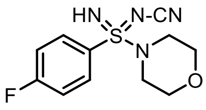

Following **General Procedure B**, sulfinamidine **10p** (92 mg, 0.50 mmol, 1.00 equiv.), $PhI(OAc)_2$ (242 mg, 0.75 mmol, 1.50 equiv.), CH_2Cl_2 (5.0 mL), Et_3N (0.21 mL, 1.50 mmol, 3.00 equiv.) and morpholine (66 μL, 0.75 mmol, 1.50 equiv.) were combined at room temperature for 10 min. Purification by flash column chromatography (petrol/ethyl acetate, 1:2 to 0:1) afforded *sulfondiimidamide* **8p** as a colourless oil that solidified upon standing to give a white solid (117 mg, 0.44 mmol, 87%).

mp 92-94 °C;

R_f 0.42 (ethyl acetate);

^{1}H NMR (400 MHz, CD_3CN): δ (ppm) = 8.05-7.97 (m, 2H), 7.42-7.34 (m, 2H), 4.03-3.59 (br. s, 1H), 3.67 (t, J = 4.7 Hz, 4H), 3.08-2.96 (m, 4H);

^{13}C NMR (101 MHz, CD_3CN): δ (ppm) = 166.8 (d, J = 254.3 Hz), 132.0 (d, J = 9.9 Hz), 130.2 (d, J = 3.0 Hz), 117.7 (d, J = 23.1 Hz), 114.0, 66.7, 47.5;

^{19}F NMR (377 MHz, CD_3CN): δ (ppm) = -105.6 (tt, J = 8.6, 5.1 Hz);

IR (ATR): $\tilde{\nu}$ (cm^{-1}) = 2188, 1588, 1491, 1227, 1110, 927, 839;

HRMS (ESI^+) calcd. for $C_{11}H_{14}FN_4OS^+$ $[M+H]^+$: 269.0867, found: 269.0865.

N-((4-(2-Chlorodibenzo[*b,f*][1,4]oxazepin-11-yl)piperazin-1-yl)(4-fluorophenyl)(imino)-λ^6-sulfaneylidene)acetamide (8q)

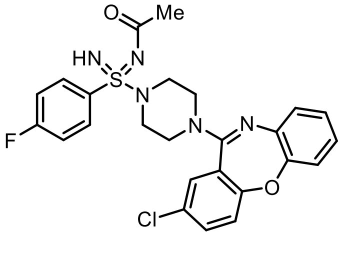

Following **General Procedure B**, sulfinamidine **10q** (100 mg, 0.50 mmol, 1.00 equiv.), $PhI(OAc)_2$ (242 mg, 0.75 mmol, 1.50 equiv.), toluene (5.0 mL), Et_3N (0.21 mL, 1.50 mmol, 3.00 equiv.) and amoxapine (235 mg, 0.75 mmol, 1.50 equiv.) were combined at room temperature for 4 h. Purification

by flash column chromatography (CH_2Cl_2/ethyl acetate, 2:1 to 1:2) afforded *sulfondiimidamide* **8q** as a colourless oil (190 mg, 0.37 mmol, 74%).

R$_f$ 0.55 (CH_2Cl_2/ethyl acetate, 1:1);

^{1}H NMR (400 MHz, CD_3CN): δ (ppm) = 8.05-8.00 (m, 2H), 7.43 (dd, J = 8.7, 2.6 Hz, 1H), 7.33-7.25 (m, 3H), 7.22 (d, J = 8.7 Hz, 1H), 7.12-6.97 (m, 4H), 3.70-3.35 (br. s, 1H), 3.48 (app. s, 4H), 3.11 (app. s, 4H), 2.04 (s, 3H);

^{13}C NMR (101 MHz, CD_3CN): δ (ppm) = 179.9, 166.1 (d, J = 252.3 Hz), 160.2, 159.4, 152.6, 140.9, 133.9, 132.3 (d, J = 3.1 Hz), 131.2 (d, J = 9.5 Hz), 131.1, 129.9, 127.7, 126.8, 125.7, 125.5, 123.7, 121.1, 117.1 (d, J = 23.0 Hz), 47.8, 46.5, 27.1;

^{19}F NMR (377 MHz, CD_3CN): δ (ppm) = -107.7 (tt, J = 8.7, 5.1 Hz);

IR (ATR): $\tilde{\nu}$ (cm^{-1}) = 1588, 1558, 1488, 1471, 1363, 1240, 1155, 1101, 934, 833;

HRMS (ESI$^+$) calcd. for $C_{25}H_{24}ClFN_5O_2S^+$ [M+H]$^+$: 512.1318, found: 512.1318.

N-((4-Fluorophenyl)(imino)(morpholino)-λ^6-sulfaneylidene)-4-(trifluoromethyl)benzamide (8r)

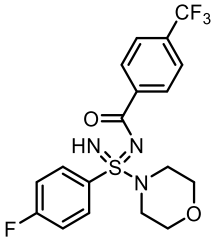

Following **General Procedure B**, sulfinamidine **10r** (165 mg, 0.50 mmol, 1.00 equiv.), $PhI(OAc)_2$ (242 mg, 0.75 mmol, 1.50 equiv.), toluene (5.0 mL), Et_3N (0.21 mL, 1.50 mmol, 3.00 equiv.) and morpholine (66 µL, 0.75 mmol, 1.50 equiv.) were combined at room temperature for 4 h. Purification by flash column chromatography (petrol/ethyl acetate, 3:2 to 2:3 to 1:2) afforded *sulfondiimidamide* **8r** as a colourless oil (172 mg, 0.41 mmol, 83%).

R$_f$ 0.50 (petrol/ethyl acetate, 2:3);

^{1}H NMR (400 MHz, CD_3CN): δ (ppm) = 8.28 (dq, J = 8.7, 0.9 Hz, 2H), 8.16-8.11 (m, 2H), 7.73 (d, J = 8.7 Hz, 2H), 7.38-7.30 (m, 2H), 3.76-3.49 (br. s, 1H), 3.65 (t, J = 4.7 Hz, 4H), 3.13-3.00 (m, 4H).

^{13}C NMR (101 MHz, CD_3CN): δ (ppm) = 172.5, 166.4 (d, J = 252.8 Hz), 141.2, 133.3 (q, J = 32.0 Hz), 131.7 (d, J = 3.0 Hz), 131.5 (d, J = 9.6 Hz), 130.4, 126.0 (q, J = 3.8 Hz), 125.2 (q, J = 271.8 Hz), 117.2 (d, J = 22.9 Hz), 67.0, 47.2;

^{19}F NMR (377 MHz, CD_3CN): δ (ppm) = -63.2 (s), -107.3 (tt, J = 8.6, 5.1 Hz).

IR (ATR): $\tilde{\nu}$ (cm^{-1}) = 1620, 1491, 1383, 1312, 1285, 1257, 1129, 1111, 1065, 1015, 921, 828;

HRMS (ESI$^+$) calcd. for $C_{18}H_{18}F_4N_3O_2S^+$ [M+H]$^+$: 416.1050, found: 416.1050.

Benzyl((4-(2-chlorodibenzo[*b*,*f*][1,4]oxazepin-11-yl)piperazin-1-yl)(4-fluorophenyl)(imino)-λ^6-sulfaneylidene)carbamate (8s)

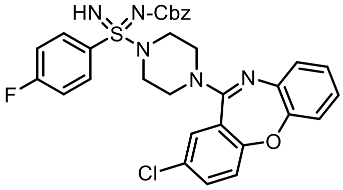

Following **General Procedure B**, sulfinamidine **10s** (146 mg, 0.50 mmol, 1.00 equiv.), $PhI(OAc)_2$ (242 mg, 0.75 mmol, 1.50 equiv.), toluene (5.0 mL), Et_3N (0.21 mL, 1.50 mmol, 3.00 equiv.) and amoxapine (235 mg, 0.75 mmol, 1.50 equiv.) were combined at room temperature for 1 h. Purification by flash column chromatography (petrol/ethyl acetate, 2:1 to 1:1) afforded *sulfondiimidamide* **8s** as a pale-yellow oil (232mg, 0.38 mmol, 77%).

R*f* 0.65 (petrol/ethyl acetate, 1:1);

^{1}H NMR (400 MHz, CD_3CN): δ (ppm) = 8.05-7.97 (m, 2H), 7.39 (dd, J = 8.6, 2.6 Hz, 1H), 7.35-7.24 (m, 7H), 7.21-7.17 (m, 2H), 7.10-6.96 (m, 4H), 5.08 (s, 2H), 3.77-3.28 (br. s, 1H), 3.43 (app. s, 4H), 3.09 (app. s, 4H);

^{13}C NMR (101 MHz, CD_3CN): δ (ppm) = 166.1 (d, J = 253.0 Hz), 160.1, 159.4, 159.3, 152.6, 140.8, 138.4, 133.9, 132.0 (d, J = 3.0 Hz), 131.3 (d, J = 9.5 Hz), 131.0, 129.8, 129.4, 128.72, 128.70, 127.7, 126.8, 125.7, 125.4, 123.7, 121.1, 117.1 (d, J = 22.8 Hz), 67.8, 47.7, 46.7;

^{19}F NMR (377 MHz, CD_3CN): δ (ppm) = -107.1 (tt, J = 8.4, 5.1 Hz);

IR (ATR): $\tilde{\nu}$ (cm^{-1}) = 1662, 1588, 1470, 1239, 1102, 1008, 933, 905;

HRMS (ESI$^+$) calcd. for $C_{31}H_{28}ClFN_5O_3S^+$ [M+H]$^+$: 604.1580, found: 604.1578.

***N*-((4-Fluorophenyl)(imino)(morpholino)-λ⁶-sulfaneylidene)-4-methylbenzenesulfonamide (8t)**

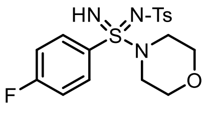

Following **General Procedure B**, sulfinamidine **10t** (156 mg, 0.50 mmol, 1.00 equiv.), $PhI(OAc)_2$ (242 mg, 0.75 mmol, 1.50 equiv.), toluene (5.0 mL), Et_3N (0.21 mL, 1.50 mmol, 3.00 equiv.) and morpholine (66 µL, 0.75 mmol, 1.50 equiv.) were combined at room temperature for 1 h. Purification by flash column chromatography (CH_2Cl_2/ethyl acetate, 2:1) afforded *sulfondiimidamide* **8t** as a colourless oil (185 mg, 0.47 mmol, 93%).

$\mathbf{R}_f$ 0.44 (CH_2Cl_2/ethyl acetate, 2:1);

^{1}H NMR (400 MHz, CD_3CN): δ (ppm) = 7.94-7.89 (m, 2H), 7.73 (d, J = 8.3 Hz, 2H), 7.29-7.22 (m, 4H), 3.62-3.25 (br. s, 1H), 3.54 (ddd, J = 11.8, 5.9, 3.7 Hz, 2H), 3.49 (ddd, J = 11.6, 7.6, 3.6 Hz, 2H), 2.98-2.84 (m, 4H), 2.35 (s, 3H);

^{13}C NMR (101 MHz, CD_3CN): δ (ppm) = 166.3 (d, J = 253.2 Hz), 143.7, 142.2, 131.5 (d, J = 9.7 Hz), 131.4 (d, J = 2.9 Hz), 130.2, 127.4, 117.1 (d, J = 23.1 Hz), 66.6, 47.4, 21.4;

^{19}F NMR (377 MHz, CD_3CN): δ (ppm) = -106.8 (tt, J = 8.6, 5.2 Hz);

IR (ATR): $\tilde{\nu}$ (cm^{-1}) = 1589, 1473, 1462, 1383, 1252, 1151, 1087, 1071, 954;

HRMS (ESI$^+$) calcd. for $C_{17}H_{21}FN_3O_3S_2^+$ [M+H]$^+$: 398.1003, found: 398.0997.

***N*-((4-(2-Chlorodibenzo[*b*,*f*][1,4]oxazepin-11-yl)piperazin-1-yl)(4-fluorophenyl)(imino)-λ⁶-sulfaneylidene)-2-(trimethylsilyl)ethane-1-sulfonamide (8u)**

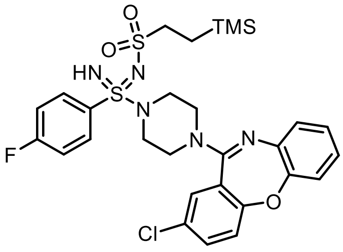

Following **General Procedure B**, sulfinamidine **10u** (161 mg, 0.50 mmol, 1.00 equiv.), $PhI(OAc)_2$ (242 mg, 0.75 mmol, 1.50 equiv.), toluene (5.0 mL), Et_3N (0.21 mL, 1.50 mmol, 3.00 equiv.) and amoxapine (235 mg, 0.75 mmol, 1.50 equiv.) were combined at room temperature for 1 h. Purification by flash column chromatography (petrol/ethyl acetate, 2:1) afforded *sulfondiimidamide* **8u** as a pale-

yellow oil (245 mg, 0.39 mmol, 77%).

R*f* 0.40 (petrol/ethyl acetate, 2:1);

^{1}H NMR (400 MHz, CD_3CN): δ (ppm) = 8.08-8.03 (m, 2H), 7.44 (dd, J = 8.7, 2.6 Hz, 1H), 7.36-7.30 (m, 2H), 7.28 (d, J = 2.6 Hz, 1H), 7.23 (d, J = 8.7 Hz, 1H), 7.12-6.97 (m, 4H), 3.60 (s, 1H), 3.53 (app. s, 4H), 3.17 (app. s, 4H), 3.06-3.01 (m, 2H), 1.06-0.99 (m, 2H), 0.00 (s, 9H);

^{13}C NMR (101 MHz, CD_3CN): δ (ppm) = 166.4 (d, J = 253.2 Hz), 160.2, 159.4, 152.6, 140.9, 134.0, 132.9 (d, J = 3.0 Hz), 131.5 (d, J = 9.6 Hz), 131.1, 129.9, 127.7, 126.8, 125.8, 125.5, 123.7, 121.2, 117.2 (d, J = 23.0 Hz), 53.3, 47.7, 47.0, 11.3, -1.9;

^{19}F NMR (377 MHz, CD_3CN): δ (ppm) = -107.0 (tt, J = 8.6, 5.1 Hz);

IR (ATR): $\tilde{\nu}$ (cm^{-1}) = 1589, 1471, 1383, 1304, 1250, 1132, 1092, 1008, 833;

HRMS (ESI$^+$) calcd. for $C_{28}H_{34}ClFN_5O_3S_2Si^+$ [M+H]$^+$: 634.1539, found: 634.1534.

N-((6,7-Dihydrothieno[3,2-*c*]pyridin-5(4*H*)-yl)(4-fluorophenyl)(imino)-λ^6-sulfaneylidene)-4-nitrobenzenesulfonamide (8v)

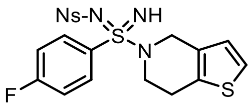

Following **General Procedure B**, sulfinamidine **10a** (172 mg, 0.50 mmol, 1.00 equiv.), $PhI(OAc)_2$ (242 mg, 0.75 mmol, 1.50 equiv.), toluene (5.0 mL), Et_3N (0.21 mL, 1.50 mmol, 3.00 equiv.) and 4,5,6,7-tetrahydrothieno[3,2-*c*]pyridine (104 mg, 0.75 mmol, 1.50 equiv.) were combined at room temperature for 1 h. Purification by flash column chromatography (CH_2Cl_2/ethyl acetate, 12:1) afforded *sulfondiimidamide* **8v** as a pale-yellow oil (221 mg, 0.46 mmol, 92%).

R*f* 0.60 (CH_2Cl_2/ethyl acetate, 10:1);

^{1}H NMR (400 MHz, CD_3CN): δ (ppm) = 8.11 (d, J = 9.0 Hz, 2H), 8.01 (d, J = 9.0 Hz, 2H), 8.00-7.96 (m, 2H), 7.27-7.21 (m, 2H), 7.05 (d, J = 5.1 Hz, 1H), 6.56 (d, J = 5.1 Hz, 1H), 4.09 (dt, J = 14.9, 1.8 Hz, 1H), 3.91 (dt, J = 14.9, 1.8 Hz, 1H), 3.85 (s, 1H), 3.68-3.59 (m, 1H), 3.18 (ddd, J = 12.4, 7.4, 5.1 Hz, 1H), 2.81-2.67 (m, 2H);

^{13}C NMR (101 MHz, CD_3CN): δ (ppm) = 166.3 (d, J = 253.7 Hz), 150.3, 149.9, 133.6, 132.8 (d, J = 3.0 Hz), 131.5, 131.1 (d, J = 9.7 Hz), 128.7, 125.9, 124.8, 124.7, 117.3 (d, J = 23.0 Hz), 47.4, 45.1, 25.6;

^{19}F NMR (377 MHz, CD_3CN): δ (ppm) = -106.2 (tt, J = 8.6, 5.0 Hz);

IR (ATR): $\tilde{\nu}$ (cm^{-1}) = 1589, 1526, 1382, 1349, 1299, 1236, 1151, 1088, 1049, 968, 734;

HRMS (ESI^+) calcd. for $C_{19}H_{18}FN_4O_4S_3^+$ $[M+H]^+$: 481.0469, found: 481.0467.

N-((4-Fluorophenyl)(imino)(1,4-dioxa-8-azaspiro[4.5]decan-8-yl)-λ^6-sulfaneylidene)-4-nitrobenzenesulfonamide (8w)

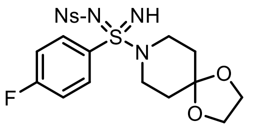

Following **General Procedure B**, sulfinamidine **10a** (172 mg, 0.50 mmol, 1.00 equiv.), $PhI(OAc)_2$ (242 mg, 0.75 mmol, 1.50 equiv.), toluene (5.0 mL), Et_3N (0.21 mL, 1.50 mmol, 3.00 equiv.) and 1,4-dioxa-8-azaspiro[4.5]decane (107 mg, 0.75 mmol, 1.50 equiv.) were combined at room temperature for 1 h. Purification by flash column chromatography (CH_2Cl_2/ethyl acetate, 4:1 to 2:1) afforded *sulfondiimidamide* **8w** as a pale-yellow solid (203 mg, 0.42 mmol, 84%).

mp 122-124 °C;

R*f* 0.57 (CH_2Cl_2/ethyl acetate, 2:1);

^{1}H NMR (400 MHz, CD_3CN): δ (ppm) = 8.23 (d, J = 8.9 Hz, 2H), 8.02 (d, J = 8.9 Hz, 2H), 7.96-7.91 (m, 2H), 7.29-7.22 (m, 2H), 3.82 (app. s, 4H), 3.63 (s, 1H), 3.13 (t, J = 5.8 Hz, 4H), 1.67-1.56 (m, 4H);

^{13}C NMR (101 MHz, CD_3CN): δ (ppm) = 166.3 (d, J = 253.8 Hz), 150.6, 150.3, 132.5 (d, J = 3.0 Hz), 131.3 (d, J = 9.6 Hz), 128.8, 125.0, 117.3 (d, J = 23.1 Hz), 106.4, 65.1, 45.7, 35.0;

^{19}F NMR (377 MHz, CD_3CN): δ (ppm) = -106.4 (tt, J = 8.8, 5.0 Hz);

IR (ATR): $\tilde{\nu}$ (cm^{-1}) = 1587, 1530, 1471, 1382, 1230, 1153, 1084, 1037, 964;

HRMS (ESI^+) calcd. for $C_{19}H_{22}FN_4O_6S_2^+$ $[M+H]^+$: 485.0959, found: 485.0951.

N-((4-Cyanopiperidin-1-yl)(4-fluorophenyl)(imino)-λ^6-sulfaneylidene)-4-nitrobenzenesulfonamide (8x)

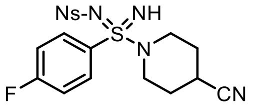

Following **General Procedure B**, sulfinamidine **10a** (172 mg, 0.50 mmol, 1.00 equiv.), $PhI(OAc)_2$ (242 mg, 0.75 mmol, 1.50 equiv.), toluene (5.0 mL), Et_3N (0.21 mL, 1.50 mmol, 3.00 equiv.) and piperidine-4-carbonitrile (83 mg, 0.75 mmol, 1.50 equiv.) were combined at room temperature for 1 h. Purification by flash column chromatography (CH_2Cl_2/ethyl acetate, 10:1 to 4:1) afforded *sulfondiimidamide* **8x** as a white solid (171 mg, 0.38 mmol, 76%).

mp 154-156 °C;

R*f* 0.50 (CH_2Cl_2/ethyl acetate, 5:1);

^{1}H NMR (400 MHz, $(CD_3)_2SO$): δ (ppm) = 8.32 (d, J = 8.9 Hz, 2H), 8.04 (d, J = 8.9 Hz, 2H), 7.95-7.89 (m, 2H), 7.47-7.41 (m, 2H), 5.41 (s, 1H), 3.31-3.17 (m, 2H), 2.93 (tt, J = 8.5, 4.0 Hz, 1H), 2.88-2.77 (m, 2H), 1.93-1.83 (m, 2H), 1.69 (ddd, J = 13.1, 8.5, 3.6 Hz, 1H), 1.62 (ddd, J = 12.5, 8.5, 3.6 Hz, 1H);

^{13}C NMR (101 MHz, $(CD_3)_2SO$): δ (ppm) = 164.7 (d, J = 252.6 Hz), 149.12, 149.10, 132.1 (d, J = 2.8 Hz), 130.5 (d, J = 9.8 Hz), 128.0, 124.1, 121.5, 116.4 (d, J = 23.0 Hz), 44.3, 43.7, 27.5, 27.5, 24.4 (note: for 4 secondary carbons in piperidine ring, 4 peaks were found instead of 2 due to the loss of symmetry caused by chiral sulfur atom);

^{19}F NMR (377 MHz, $(CD_3)_2SO$): δ (ppm) = -105.8 (tt, J = 8.7, 5.1 Hz);

IR (ATR): $\tilde{\nu}$ (cm^{-1}) = 1526, 1381, 1347, 1294, 1146, 1087, 1055, 952, 836;

HRMS (ESI$^+$) calcd. for $C_{18}H_{19}FN_5O_4S_2^+$ [M+H]$^+$: 452.0857, found: 452.0856.

N-((4-Fluorophenyl)(imino)(4-(pyrimidin-2-yl)piperazin-1-yl)-λ^6-sulfaneylidene)-4-nitrobenzenesulfonamide (8y)

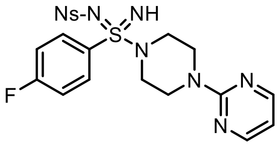

Following **General Procedure B**, sulfinamidine **10a** (172 mg, 0.50 mmol, 1.00 equiv.), $PhI(OAc)_2$ (242 mg, 0.75 mmol, 1.50 equiv.), toluene (5.0 mL), Et_3N (0.21 mL, 1.50 mmol, 3.00 equiv.) and 2-(piperazin-1-yl)pyrimidine (123 mg, 0.75 mmol, 1.50 equiv.) were combined at room temperature for 1 h. Purification by flash column chromatography (petrol/ethyl acetate, 1:1 to 1:3) afforded *sulfondiimidamide* **8y** as a colourless oil (233 mg, 0.46 mmol, 92%).

R$_f$ 0.31 (petrol/ethyl acetate, 1:2);

^{1}H NMR (400 MHz, CD_3CN): δ (ppm) = 8.26 (d, J = 4.8 Hz, 2H), 8.23 (d, J = 8.9 Hz, 2H), 8.04 (d, J = 8.9 Hz, 2H), 7.95-7.91 (m, 2H), 7.27-7.22 (m, 2H), 6.54 (t, J = 4.8 Hz, 1H), 3.78-3.69 (m, 5H), 3.08-3.02 (m, 4H);

^{13}C NMR (101 MHz, CD_3CN): δ (ppm) = 166.5 (d, J = 253.9 Hz), 162.2, 158.8, 150.6, 150.3, 131.7 (d, J = 3.2 Hz), 131.6 (d, J = 9.9 Hz), 128.8, 125.0, 117.3 (d, J = 23.1 Hz), 111.6, 47.0, 43.8;

^{19}F NMR (377 MHz, CD_3CN): δ (ppm) = -106.4 (tt, J = 8.6, 5.0 Hz);

IR (ATR): $\tilde{\nu}$ (cm^{-1}) = 1585, 1528, 1488, 1448, 1352, 1303, 1154, 1088, 1049, 950;

HRMS (ESI^+) calcd. for $C_{20}H_{21}FN_7O_4S_2^+$ $[M+H]^+$: 506.1075, found: 506.1073.

***N*-((4-(2-Chlorodibenzo[*b*,*f*][1,4]oxazepin-11-yl)piperazin-1-yl)(4-fluorophenyl)(imino)-λ^6-sulfaneylidene)-4-nitrobenzenesulfonamide (8z)**

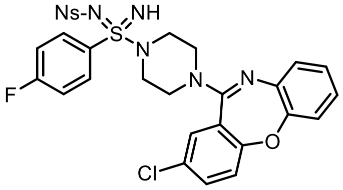

Following **General Procedure B**, sulfinamidine **10a** (172 mg, 0.50 mmol, 1.00 equiv.), $PhI(OAc)_2$ (242 mg, 0.75 mmol, 1.50 equiv.), toluene (5.0 mL), Et_3N (0.21 mL, 1.50 mmol, 3.00 equiv.) and amoxapine (235 mg, 0.75 mmol, 1.50 equiv.) were combined at room temperature for 2 h. Purification by flash column chromatography (petrol/ethyl acetate, 2:1 to 1:1) afforded *sulfondiimidamide* **8z** as a pale-yellow oil (293 mg, 0.45 mmol, 90%).

R$_f$ 0.68 (petrol/ethyl acetate, 1:1);

^{1}H NMR (400 MHz, $(CD_3)_2CO$): δ (ppm) = 8.31 (d, J = 9.0 Hz, 2H), 8.17 (d, J = 9.0 Hz, 2H), 8.11-8.04 (m, 2H), 7.48 (dd, J = 8.7, 2.6 Hz, 1H), 7.36-7.27 (m, 4H), 7.14-7.04 (m, 3H), 7.00 (ddd, J = 7.8, 6.8, 2.3 Hz, 1H), 4.32 (s, 1H), 3.58 (app. s, 4H), 3.29 (app. s, 4H).

^{13}C NMR (101 MHz, $(CD_3)_2CO$): δ (ppm) = 166.2 (d, J = 253.9 Hz), 160.1, 159.0, 152.5, 150.6, 150.4, 140.6, 133.8, 132.3 (d, J = 3.0 Hz), 131.6 (d, J = 9.7 Hz), 130.9, 129.6, 128.9, 127.8, 126.5, 125.6, 125.4, 124.8, 123.8, 121.0, 117.1 (d, J = 23.0 Hz), 47.5, 46.8;

^{19}F NMR (377 MHz, $(CD_3)_2CO$): δ (ppm) = -106.2 (tt, J = 8.5, 5.0 Hz);

IR (ATR): $\tilde{\nu}$ (cm^{-1}) = 1588, 1528, 1472, 1382, 1241, 1151, 1087, 731;

HRMS (ESI$^+$) calcd. for $C_{29}H_{25}ClFN_6O_5S_2^+$ [M+H]$^+$: 655.0995, found: 655.0993.

***N*-((4-(Benzo[*d*]isothiazol-3-yl)piperazin-1-yl)(4-fluorophenyl)(imino)-λ^6-sulfaneylidene)-4-nitrobenzenesulfonamide (8aa)**

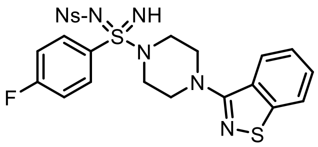

Following **General Procedure B**, sulfinamidine **10a** (172 mg, 0.50 mmol, 1.00 equiv.), PhI(OAc)$_2$ (242 mg, 0.75 mmol, 1.50 equiv.), toluene (5.0 mL), Et_3N (0.21 mL, 1.50 mmol, 3.00 equiv.) and 3-(piperazin-1-yl)benzo[*d*]isothiazole (165 mg, 0.75 mmol, 1.50 equiv.) were combined at room temperature for 1 h. Purification by flash column chromatography (CH_2Cl_2/ethyl acetate, 12:1 to 7:1) afforded *sulfondiimidamide* **8aa** as a pale-yellow oil (253 mg, 0.45 mmol, 90%).

R*f* 0.54 (CH_2Cl_2/ethyl acetate, 10:1);

^{1}H NMR (400 MHz, $(CD_3)_2CO$): δ (ppm) = 8.30 (d, J = 8.9 Hz, 2H), 8.18 (d, J = 8.9 Hz, 2H), 8.13-8.08 (m, 2H), 7.98-7.90 (m, 2H), 7.50 (ddd, J = 8.2, 7.0, 1.0 Hz, 1H), 7.40-7.33 (m, 3H), 4.35 (s, 1H), 3.57-3.48 (m, 4H), 3.36 (t, J = 4.9 Hz, 4H);

^{13}C NMR (101 MHz, $(CD_3)_2CO$): δ (ppm) = 166.2 (d, J = 253.9 Hz), 163.7, 153.4, 150.5, 150.3, 132.1 (d, J = 3.0 Hz), 131.5 (d, J = 9.7 Hz), 128.8, 128.6, 128.3, 125.0, 124.7, 124.6, 121.4, 117.1 (d, J = 23.0 Hz), 50.0, 46.9;

^{19}F NMR (377 MHz, $(CD_3)_2CO$): δ (ppm) = -106.2 (tt, J = 9.1, 5.1 Hz);

IR (ATR): $\tilde{\nu}$ (cm^{-1}) = 1589, 1528, 1489, 1383, 1350, 1263, 1087, 1056, 891, 731;

HRMS (ESI$^+$) calcd. for $C_{23}H_{22}FN_6O_4S_3^+$ [M+H]$^+$: 561.0843, found: 561.0842.

***N*-((4-Fluorophenyl)(imino)(pyrrolidin-1-yl)-λ^6-sulfaneylidene)-4-nitrobenzenesulfonamide (8ab)**

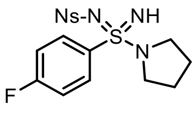

Following **General Procedure B**, sulfinamidine **10a** (172 mg, 0.50 mmol, 1.00 equiv.), PhI(OAc)$_2$

(242 mg, 0.75 mmol, 1.50 equiv.), toluene (5.0 mL), Et_3N (0.21 mL, 1.50 mmol, 3.00 equiv.) and pyrrolidine (53 mg, 0.75 mmol, 1.50 equiv.) were combined at room temperature for 1 h. Purification by flash column chromatography (CH_2Cl_2/ethyl acetate, 10:1 to 5:1) afforded *sulfondiimidamide* **8ab** as a white solid (160 mg, 0.39 mmol, 78%).

mp 124-126 °C;

$\mathbf{R}_f$ 0.50 (CH_2Cl_2/ethyl acetate, 9:1);

^{1}H NMR (400 MHz, CD_3CN): δ (ppm) = 8.22 (d, J = 9.0 Hz, 2H), 8.00 (d, J = 9.0 Hz, 2H), 7.99-7.95 (m, 2H), 7.27-7.21 (m, 2H), 3.65 (s, 1H), 3.24-3.17 (m, 2H), 3.16-3.09 (m, 2H), 1.73-1.67 (m, 4H);

^{13}C NMR (101 MHz, CD_3CN): δ (ppm) = 166.3 (d, J = 253.4 Hz), 150.6, 150.5, 133.7 (d, J = 3.1 Hz), 131.5 (d, J = 9.7 Hz), 128.8, 124.9, 117.2 (d, J = 23.1 Hz), 49.3, 25.9;

^{19}F NMR (377 MHz, CD_3CN): δ (ppm) = -106.9 (tt, J = 8.7, 5.1 Hz);

IR (ATR): $\tilde{\nu}$ (cm^{-1}) = 1588, 1521, 1381, 1348, 1293, 1234, 1148, 1083, 954, 849, 616;

HRMS (ESI$^+$) calcd. for $C_{16}H_{18}FN_4O_4S_2^+$ $[M+H]^+$: 413.0748, found: 413.0747.

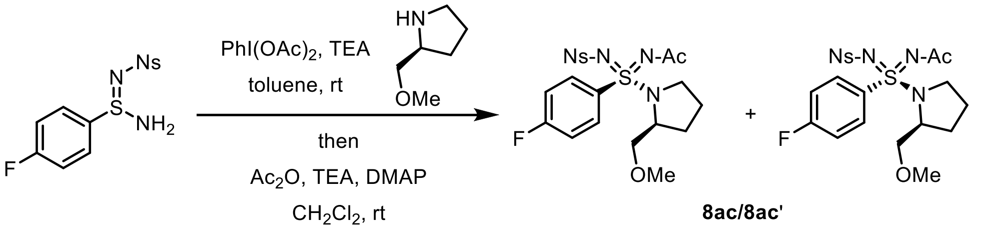

8ac/8ac'

Step 1

An oven-dried 25 mL round-bottom flask containing primary sulfinamidine **10a** (343 mg, 1.00 mmol, 1.00 equiv.) and $PhI(OAc)_2$ (483 mg, 1.50 mmol, 1.50 equiv.) was sealed and subjected to three N_2 evacuation/refill cycles before pre-sparged anhydrous toluene (5.0 mL) was added. Et_3N (0.42 mL, 3.00 mmol, 3.00 equiv.) was added to the solution, followed immediately by the addition of (*S*)-(+)-2-(methoxymethyl)pyrrolidine (346 mg, 3.00 mmol, 3.00 equiv.). The reaction mixture was then stirred at room temperature for 30 min until completion (judged by TLC). The crude product was purified by flash column chromatography (CH_2Cl_2/ethyl acetate, 20:1 to 4:1) without an aqueous work-up to afford a mixture of sulfondiimidamide diastereoisomers as a colourless oil (344 mg, 0.75 mmol, 75%).

Step 2

This diastereoisomers mixture (344 mg, 0.75 mmol, 1.00 equiv.) was then dissolved in anhydrous

CH_2Cl_2 (3.7 mL). Et_3N (0.42 mL, 1.50 mmol, 2.00 equiv.) was then added, followed by the addition of Ac_2O (106 µL, 1.13 mmol, 1.50 equiv.) and DMAP (18.3 mg, 0.15 mmol, 0.20 equiv.). After being stirred at room temperature for 3h, the reaction mixture was diluted with CH_2Cl_2 (70 mL) and quenched with sat. aq. NaCl solution (100 mL). The aqueous phase was separated and extracted with CH_2Cl_2 (2 × 30 mL). The combined organic layers were dried over anhydrous Na_2SO_4 and concentrated under reduced pressure. The crude product was purified by flash column chromatography (petrol/ethyl acetate, 1:1 to 1:2) to afford *diastereoisomer* **8ac** as a colourless oil (174 mg, 0.35 mmol, 35% over two steps) and *diastereoisomer* **8ac'** as a colourless oil (173 mg, 0.35 mmol, 35% over two steps). (Sulfur stereochemistry of these two compounds remains unknown).

***N*-((*R*)-(4-Fluorophenyl)((*S*)-2-(methoxymethyl)pyrrolidin-1-yl)(((4-nitrophenyl)sulfonyl)imino)-λ⁶-sulfaneylidene)acetamide**

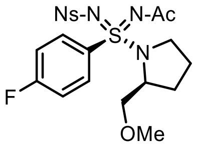

***N*-((*S*)-(4-Fluorophenyl)((*S*)-2-(methoxymethyl)pyrrolidin-1-yl)(((4-nitrophenyl)sulfonyl)imino)-λ⁶-sulfaneylidene)acetamide**

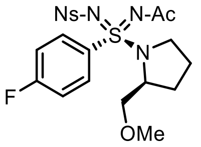

Diastereoisomer 1 (Sulfur stereochemistry is unknown)

R*f* 0.59 (petrol/ethyl acetate, 2:3);

^{1}H NMR (400 MHz, $CDCl_3$): δ (ppm) = 8.29 (d, J = 8.9 Hz, 2H), 8.14 (d, J = 8.9 Hz, 2H), 8.05-8.00 (m, 2H), 7.22-7.15 (m, 2H), 4.61 (ddt, J = 8.5, 5.7, 2.8 Hz, 1H), 3.65-3.53 (m, 2H), 3.47 (dd, J = 9.7, 3.0 Hz, 1H), 3.36 (s, 3H), 2.96-2.87 (m, 1H), 2.30-2.16 (m, 1H), 2.13-1.95 (m, 3H), 1.69 (s, 3H);

^{13}C NMR (101 MHz, $CDCl_3$): δ (ppm) = 176.8, 165.9 (d, J = 258.1 Hz), 149.8, 148.1, 132.6 (d, J = 3.1 Hz), 131.6 (d, J = 9.6 Hz), 128.5, 123.9, 116.8 (d, J = 23.1 Hz), 74.6, 61.3, 59.2, 48.1, 29.0, 26.4, 24.6;

^{19}F NMR (377 MHz, $CDCl_3$): δ (ppm) = -102.6 (tt, J = 8.0, 4.9 Hz);

IR (ATR): $\tilde{\nu}$ (cm^{-1}) =2360, 2341, 1656, 1530, 1381, 1234, 1162, 1059;

HRMS (ESI$^+$) calcd. for $C_{20}H_{24}FN_4O_6S_2^+$ [M+H]$^+$: 499.1116, found: 499.1113;

$[\alpha]_D^{25}$: +26.7° (c = 1.0, $CHCl_3$).

Diastereoisomer 2 (Sulfur stereochemistry is unknown)

R*f* 0.50 (petrol/ethyl acetate, 2:3);

^{1}H NMR (400 MHz, $CDCl_3$): δ (ppm) = 8.22 (d, J = 8.9 Hz, 2H), 8.04-7.98 (m, 4H), 7.20-7.13 (m, 2H), 4.53-4.43 (m, 1H), 3.48-3.38 (m, 2H), 3.28 (s, 3H), 3.21-3.12 (m, 1H), 2.99 (dtd, J = 10.7, 4.2, 2.3 Hz, 1H), 2.10-1.90 (m, 3H), 1.87-1.78 (m, 4H);

^{13}C NMR (101 MHz, $CDCl_3$): δ (ppm) = 177.7, 165.9 (d, J = 258.2 Hz), 149.6, 148.2, 132.7 (d, J = 3.1 Hz), 131.3 (d, J = 9.7 Hz), 128.4, 123.8, 116.8 (d, J = 23.0 Hz), 74.3, 61.3, 59.1, 48.1, 28.5, 26.4, 24.5;

^{19}F NMR (377 MHz, $CDCl_3$): δ (ppm) = -102.4 (tt, J = 8.0, 4.9 Hz);

IR (ATR): $\tilde{\nu}$ (cm^{-1}) = 2360, 2341, 1657, 1530, 1382, 1236, 1162, 1086, 955;

HRMS (ESI^+) calcd. for $C_{20}H_{24}FN_4O_6S_2^+$ $[M+H]^+$: 499.1116, found: 499.1114;

$[\alpha]_D^{25}$: +13.1° (c = 1.0, $CHCl_3$).

***N*-(Azepan-1-yl(4-fluorophenyl)(imino)-λ⁶-sulfaneylidene)-4-nitrobenzenesulfonamide (8ad)**

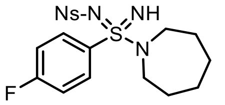

Following **General Procedure B**, sulfinamidine **10a** (172 mg, 0.50 mmol, 1.00 equiv.), $PhI(OAc)_2$ (242 mg, 0.75 mmol, 1.50 equiv.), toluene (2.5 mL), Et_3N (0.21 mL, 1.50 mmol, 3.00 equiv.) and azepane (150 mg, 1.50 mmol, 3.00 equiv.) were combined at room temperature for 15 min. Purification by flash column chromatography (petrol/ethyl acetate, 2:1 to 2:3) afforded *sulfondiimidamide* **8ad** as a colourless oil (190 mg, 0.43 mmol, 86%).

R*f* 0.50 (petrol/ethyl acetate, 1:1);

^{1}H NMR (400 MHz, CD_3CN): δ (ppm) = 8.21 (d, J = 8.9 Hz, 2H), 7.99 (d, J = 8.9 Hz, 2H), 7.93-7.88 (m, 2H), 7.23-7.17 (m, 2H), 3.53 (s, 1H), 3.35 (ddd, J = 13.8, 7.0, 4.7 Hz, 2H), 3.26 (ddd, J = 13.8, 6.9, 4.5 Hz, 2H), 1.71-1.49 (m, 8H);

^{13}C NMR (101 MHz, CD_3CN): δ (ppm) = 166.0 (d, J = 253.1 Hz), 150.7, 150.4, 135.7 (d, J = 3.1 Hz), 130.9 (d, J = 9.8 Hz), 128.7, 124.9, 117.1 (d, J = 23.0 Hz), 49.5, 29.6, 27.1;

^{19}F NMR (377 MHz, CD_3CN): δ (ppm) = -107.2 (tt, J = 8.5, 5.1 Hz);

IR (ATR): $\tilde{\nu}$ (cm^{-1}) = 1589, 1528, 1489, 1350, 1300, 1235, 1151, 1088, 1046, 1009;

HRMS (ESI$^+$) calcd. for $C_{18}H_{22}FN_4O_4S_2^+$ [M+H]$^+$: 441.1061, found: 441.1054.

N-((Benzyl(methyl)amino)(4-fluorophenyl)(imino)-λ^6-sulfaneylidene)-4-nitrobenzenesulfonamide (8ae)

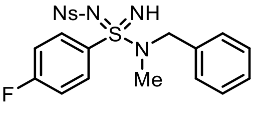

Following **General Procedure B**, sulfinamidine **10a** (172 mg, 0.50 mmol, 1.00 equiv.), $PhI(OAc)_2$ (242 mg, 0.75 mmol, 1.50 equiv.), toluene (2.5 mL), Et_3N (0.21 mL, 1.50 mmol, 3.00 equiv.) and *N*-benzylmethylamine (182 mg, 1.50 mmol, 3.00 equiv.) were combined at room temperature for 10 min. Purification by flash column chromatography (petrol/ethyl acetate, 2:1 to 1:1) afforded *sulfondiimidamide* **8ae** as a colourless oil (213 mg, 0.46 mmol, 92%).

R$_f$ 0.46 (petrol/ethyl acetate, 2:1);

^{1}H NMR (400 MHz, CD_3CN): δ (ppm) = 8.20 (d, J = 8.9 Hz, 2H), 8.04 (d, J = 8.9 Hz, 2H), 8.03-7.98 (m, 2H), 7.34-7.28 (m, 7H), 4.29 (d, J = 14.2 Hz, 1H), 4.21 (d, J = 14.2 Hz, 1H), 3.80 (s, 1H), 2.55 (s, 3H);

^{13}C NMR (101 MHz, CD_3CN): δ (ppm) = 166.3 (d, J = 253.6 Hz), 150.5, 150.3, 136.7, 133.2 (d, J = 3.0 Hz), 131.3 (d, J = 9.7 Hz), 129.5, 129.3, 128.8, 128.7, 125.0, 117.3 (d, J = 23.1 Hz), 54.6, 35.5;

^{19}F NMR (377 MHz, CD_3CN): δ (ppm) = -106.4 (tt, J = 8.4, 5.0 Hz);

IR (ATR): $\tilde{\nu}$ (cm^{-1}) = 2361, 1589, 1528, 1490, 1350, 1301, 1153, 1088, 1051;

HRMS (ESI$^+$) calcd. for $C_{20}H_{20}FN_4O_4S_2^+$ [M+H]$^+$: 463.0905, found: 463.0907.

N-((Diethylamino)(4-fluorophenyl)(imino)-λ^6-sulfaneylidene)-4-nitrobenzenesulfonamide (8af)

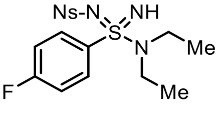

Following **General Procedure B**, sulfinamidine **10a** (172 mg, 0.50 mmol, 1.00 equiv.), $PhI(OAc)_2$ (242 mg, 0.75 mmol, 1.50 equiv.), toluene (2.5 mL), Et_3N (0.21 mL, 1.50 mmol, 3.00 equiv.) and

diethylamine (110 mg, 1.50 mmol, 3.00 equiv.) were combined at room temperature for 1 h. Purification by flash column chromatography (petrol/ethyl acetate, 2:1 to 2:3) afforded *sulfondiimidamide* **8af** as a colourless oil (170 mg, 0.41 mmol, 82%).

R*f* 0.40 (CH_2Cl_2/ethyl acetate, 15:1);

^{1}H NMR (400 MHz, CD_3CN): δ (ppm) = 8.20 (d, J = 9.0 Hz, 2H), 7.97 (d, J = 9.0 Hz, 2H), 7.94-7.89 (m, 2H), 7.23-7.17 (m, 2H), 3.43 (s, 1H), 3.35-3.19 (m, 4H), 1.05 (t, J = 7.1 Hz, 6H);

^{13}C NMR (101 MHz, CD_3CN): δ (ppm) = 166.1 (d, J = 253.1 Hz), 150.53, 150.47, 136.4 (d, J = 3.1 Hz), 131.1 (d, J = 9.6 Hz), 128.7, 124.9, 117.1 (d, J = 23.1 Hz), 43.1, 14.3;

^{19}F NMR (377 MHz, CD_3CN): δ (ppm) = -107.3 (tt, J = 8.6, 5.1 Hz);

IR (ATR): $\tilde{\nu}$ (cm^{-1}) = 1589, 1526, 1489, 1382, 1349, 1298, 1235, 1151, 1087, 1045, 1003, 766, 613;

HRMS (ESI^+) calcd. for $C_{16}H_{20}FN_4O_4S_2^+$ $[M+H]^+$: 415.0905, found: 415.0904.

N-((Diallylamino)(4-fluorophenyl)(imino)-λ^6-sulfaneylidene)-4-nitrobenzenesulfonamide (8ag)

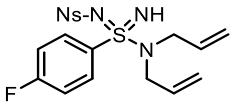

Following **General Procedure B**, sulfinamidine **10a** (343 mg, 1.00 mmol, 1.00 equiv.), $PhI(OAc)_2$ (483 mg, 1.50 mmol, 1.50 equiv.), toluene (5.0 mL), Et_3N (0.42 mL, 3.00 mmol, 3.00 equiv.) and diallyl amine (292 mg, 3.00 mmol, 3.00 equiv.) were combined at room temperature for 1 h. Purification by flash column chromatography (petrol/ethyl acetate, 3:1 to 1:1) afforded *sulfondiimidamide* **8ag** as a pale-yellow oil (400 mg, 0.91 mmol, 91%).

R*f* 0.40 (petrol/ethyl acetate, 2:1);

^{1}H NMR (400 MHz, CD_3CN): δ (ppm) = 8.21 (d, J = 8.9 Hz, 2H), 8.01-7.93 (m, 4H), 7.24-7.18 (m, 2H), 5.63 (ddt, J = 16.6, 10.1, 6.3 Hz, 2H), 5.23-5.09 (m, 4H), 3.93-3.77 (m, 4H), 3.63 (s, 1H);

^{13}C NMR (101 MHz, CD_3CN): δ (ppm) = 166.2 (d, J = 253.5 Hz), 150.6, 150.3, 136.1 (d, J = 3.1 Hz), 133.5, 131.3 (d, J = 9.8 Hz), 128.8, 125.0, 119.9, 117.2 (d, J = 23.0 Hz), 50.5;

^{19}F NMR (377 MHz, CD_3CN): δ (ppm) = -106.9 (tt, J = 8.4, 5.1 Hz);

IR (ATR): $\tilde{\nu}$ (cm^{-1}) = 1589, 1526, 1491, 1350, 1294, 1233, 1150, 1089, 1007, 744;

HRMS (ESI^+) calcd. for $C_{18}H_{20}FN_4O_4S_2^+$ $[M+H]^+$: 439.0905, found: 439.0895.

1.2.3 The Synthesis of Sulfondiimidamide 12

***N*-((Benzylimino)(diallylamino)(4-fluorophenyl)-λ^6-sulfaneylidene)-4-nitrobenzenesulfonamide (11a)**

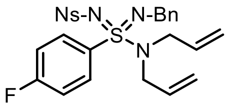

To an oven-dried 25 mL round-bottom flask under nitrogen atmosphere was added sulfondiimidamide **8ag** (300 mg, 0.68 mmol, 1.00 equiv.) and anhydrous CH_3CN (2.20 mL) at room temperature. DBU (0.41 mL, 2.74 mmol, 4.00 equiv.) was then added and the reaction was stirred at room temperature for 10 min, followed by the addition of BnBr (350 mg, 2.05 mmol, 3.00 equiv.). After being stirred at room temperature for 20 h, the reaction was diluted with ethyl acetate (70 mL) and quenched with sat. aq. NaCl solution (100 mL). The aqueous phase was separated and extracted with ethyl acetate (2 × 50 mL). The combined organic layers were dried over anhydrous Na_2SO_4 and concentrated under reduced pressure. The crude product was purified by flash column chromatography (petrol/ethyl acetate, 3:1 to 2:1) to afford *sulfondiimidamide* **11a** as a pale-yellow oil (240 mg, 0.45 mmol, 67%).

R_f 0.40 (petrol/ethyl acetate, 4:1);

^{1}H NMR (400 MHz, $CDCl_3$): δ (ppm) = 8.06 (d, J = 8.8 Hz, 2H), 8.00-7.96 (m, 2H), 7.93 (d, J = 8.8 Hz, 2H), 7.34-7.28 (m, 4H), 7.27-7.21 (m, 1H), 7.16-7.10 (m, 2H), 5.54 (dddd, J = 17.1, 10.2, 7.1, 6.1 Hz, 2H), 5.22-5.11 (m, 4H), 4.20 (d, J = 15.1 Hz, 1H), 4.11 (d, J = 15.1 Hz, 1H), 3.96 (dd, J = 15.6, 6.2 Hz, 2H), 3.76 (dd, J = 15.1, 7.2 Hz, 2H);

^{13}C NMR (101 MHz, $CDCl_3$): δ (ppm) = 165.2 (d, J = 256.6 Hz), 149.3, 149.0, 139.5, 134.5 (d, J = 3.1 Hz), 131.9, 130.8 (d, J = 9.4 Hz), 128.3, 127.9, 127.0, 126.9, 123.6, 120.2, 116.3 (d, J = 22.7 Hz), 49.2, 46.5;

^{19}F NMR (377 MHz, $CDCl_3$): δ (ppm) = -104.3 (tt, J = 8.1, 4.9 Hz);

IR (ATR): $\tilde{\nu}$ (cm^{-1}) = 1590, 1528, 1489, 1350, 1302, 1154, 1089, 1056, 930;

HRMS (ESI^+) calcd. for $C_{25}H_{26}FN_4O_4S_2^+$ $[M+H]^+$: 529.1374, found: 529.1373.

N-(Amino(benzylimino)(4-fluorophenyl)-λ^6-sulfaneylidene)-4-nitrobenzenesulfonamide (12a)

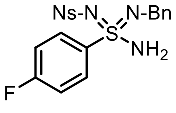

Sulfondiimidamide **11a** (260 mg, 0.49 mmol, 1.00 equiv.), 1,3-dimethylbarbituric acid (459 mg, 2.94 mmol, 6.00 equiv.), $Pd(PPh_3)_4$ (142 mg, 0.12 mmol, 0.25 equiv.) were dissolved in anhydrous, degassed CH_2Cl_2 (2.50 mL) in an oven-dried 25 mL round-bottom flask under a nitrogen atmosphere. After being stirred at 35 °C for 12 h, the reaction mixture was diluted with ethyl acetate (50 mL) and quenched with 0.2 M aq. Na_2CO_3 solution (60 mL). The aqueous phase was separated and extracted with ethyl acetate (2 × 30 mL). The combined organic layers were dried over anhydrous Na_2SO_4 and concentrated under reduced pressure. The crude product was purified by flash column chromatography (petrol/ethyl acetate, 3:2 to 2:3) to afford *sulfondiimidamide* **12a** as a pale-yellow solid (132 mg, 0.29 mmol, 60%).

mp 122-124 °C;

R*f* 0.60 (petrol/ethyl acetate, 1:1);

^{1}H NMR (400 MHz, $CDCl_3$): δ (ppm) = 8.19 (d, J = 8.9 Hz, 2H), 8.04-7.99 (m, 2H), 7.98 (d, J = 8.9 Hz, 2H), 7.30-7.25 (m, 3H), 7.18-7.09 (m, 4H), 6.69-1.87 (br. s, 2H), 4.17 (d, J = 14.1 Hz, 1H), 4.04 (d, J = 14.1 Hz, 1H);

^{13}C NMR (101 MHz, $CDCl_3$): δ (ppm) = 165.6 (d, J = 257.5 Hz), 149.6, 148.9, 135.6, 133.7 (d, J = 3.1 Hz), 130.5 (d, J = 9.5 Hz), 128.9, 128.3, 128.1, 128.0, 124.0, 116.6 (d, J = 22.8 Hz), 46.4;

^{19}F NMR (377 MHz, $CDCl_3$): δ (ppm) = -103.4 (tt, J = 8.0, 5.0 Hz);

IR (ATR): $\tilde{\nu}$ (cm^{-1}) = 1589, 1527, 1492, 1350, 1282, 1236, 1151, 1092, 1025, 1009, 967;

HRMS (ESI$^+$) calcd. for $C_{19}H_{18}FN_4O_4S_2^+$ [M+H]$^+$: 449.0748, found: 449.0744.

N-((Diallylamino)(4-fluorophenyl)(methylimino)-λ^6-sulfaneylidene)-4-nitrobenzenesulfonamide (11b)

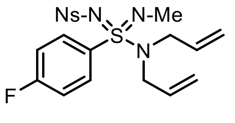

To an oven-dried 25 mL round-bottom flask under nitrogen atmosphere was added

sulfondiimidamide **8ag** (387 mg, 0.88 mmol, 1.00 equiv.) and anhydrous CH_3CN (4.40 mL) at room temperature. DBU (0.40 mL, 2.64 mmol, 3.00 equiv.) was then added and the reaction was stirred at room temperature for 10 min, followed by the addition of MeI (0.55 mL, 8.83 mmol, 10.00 equiv.). After being stirred at room temperature for 18 h, the reaction was diluted with ethyl acetate (60 mL) and quenched with sat. aq. NaCl solution (100 mL). The aqueous phase was separated and extracted with ethyl acetate (2 × 50 mL). The combined organic layers were dried over anhydrous Na_2SO_4 and concentrated under reduced pressure. The crude product was purified by flash column chromatography (petrol/ethyl acetate, 2:1 to 1:1) to afford *sulfondiimidamide* **11b** as a white solid (288 mg, 0.64 mmol, 72%).

mp 64-66 °C;

R*f* 0.48 (petrol/ethyl acetate, 2:1);

^{1}H NMR (400 MHz, $CDCl_3$): δ (ppm) = 8.17 (d, *J* = 8.9 Hz, 2H), 7.95 (d, *J* = 8.9 Hz, 2H), 7.88-7.83 (m, 2H), 7.10-7.04 (m, 2H), 5.52 (dddd, *J* = 17.1, 10.2, 7.0, 6.1 Hz, 2H), 5.18-5.09 (m, 4H), 3.90 (dd, *J* = 15.5, 6.2 Hz, 2H), 3.74 (dd, *J* = 15.3, 7.0 Hz, 2H), 2.61 (s, 3H);

^{13}C NMR (101 MHz, $CDCl_3$): δ (ppm) = 165.2 (d, *J* = 256.6 Hz), 149.4, 149.3, 134.6 (d, *J* = 3.2 Hz), 132.0, 130.7 (d, *J* = 9.3 Hz), 128.0, 123.7, 120.1, 116.3 (d, *J* = 22.7 Hz), 49.0, 29.7;

^{19}F NMR (377 MHz, $CDCl_3$): δ (ppm) = -104.6 (tt, *J* = 8.0, 5.1 Hz);

IR (ATR): $\tilde{\nu}$ (cm^{-1}) = 1588, 1528, 1490, 1350, 1301, 1231, 1154, 1089, 1052, 1010;

HRMS (ESI^+) calcd. for $C_{19}H_{22}FN_4O_4S_2^+$ $[M+H]^+$: 453.1061, found: 453.1061.

N-(Amino(4-fluorophenyl)(methylimino)-λ^6-sulfaneylidene)-4-nitrobenzenesulfonamide (12b)

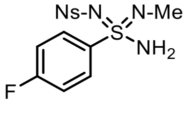

Sulfondiimidamide **11b** (180 mg, 0.40 mmol, 1.00 equiv.), 1,3-dimethylbarbituric acid (374 mg, 2.40 mmol, 6.00 equiv.) and $Pd(PPh_3)_4$ (139 mg, 0.12 mmol, 0.30 equiv.) were dissolved in anhydrous, degassed CH_2Cl_2 (2.00 mL) in an oven-dried 25 mL round-bottom flask under a nitrogen atmosphere. After being stirred at 30 °C for 16 h, the reaction mixture was diluted with ethyl acetate (40 mL) and quenched with 0.2 M aq. Na_2CO_3 solution (60 mL). The aqueous phase was separated and extracted with ethyl acetate (2 × 30 mL). The combined organic layers were dried over anhydrous

Na_2SO_4 and concentrated under reduced pressure. The crude product was purified by flash column chromatography (petrol/ethyl acetate, 1:1 to 1:2) to afford *sulfondiimidamide* **12b** as a pale-yellow solid (79 mg, 0.21 mmol, 53%).

mp 116-118 °C;

R*f* 0.44 (petrol/ethyl acetate, 1:2);

^{1}H NMR (400 MHz, $(CD_3)_2SO$): δ (ppm) = 8.24 (d, J = 8.8 Hz, 2H), 7.95-7.70 (m, 5H), 7.40-7.29 (m, 2H), 4.94 (s, 1H), 2.31 (s, 3H);

^{13}C NMR (101 MHz, $(CD_3)_2SO$): δ (ppm) = 164.3 (d, J = 251.6 Hz), 149.3, 148.9, 134.6 (d, J = 3.8 Hz), 130.3 (d, J = 9.7 Hz), 127.8, 123.9, 116.1 (d, J = 22.9 Hz), 26.9;

^{19}F NMR (377 MHz, $(CD_3)_2SO$): δ (ppm) = -106.9 (s);

IR (ATR): $\tilde{\nu}$ (cm^{-1}) = 1588, 1528, 1489, 1350, 1299, 1236, 1154, 1091, 1034, 1009;

HRMS (ESI$^+$) calcd. for $C_{13}H_{14}FN_4O_4S_2^+$ [M+H]$^+$: 373.0435, found: 373.0433.

***N*-((Cyanoimino)(diallylamino)(4-fluorophenyl)-λ^6-sulfaneylidene)-4-nitrobenzenesulfonamide (11c)**

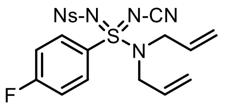

To an oven-dried 50 mL round-bottom flask under nitrogen atmosphere was added sulfondiimidamide **8ag** (573 mg, 1.31 mmol, 1.00 equiv.) and anhydrous CH_3CN (6.5 mL) at room temperature. Et_3N (0.37 mL, 2.65 mmol, 2.00 equiv.) was then added, followed by the addition of BrCN solution (0.39 mL, 5.0 M in CH_3CN, 1.95 mmol, 1.50 equiv.). After being stirred at room temperature for 20 min, the reaction mixture was diluted with ethyl acetate (80 mL) and quenched with sat. aq. $NaHCO_3$ solution (100 mL). The aqueous phase was separated and extracted with ethyl acetate (2 × 40 mL). The combined organic layers were dried over anhydrous Na_2SO_4 and concentrated under reduced pressure. The crude product was purified by flash column chromatography (petrol/ethyl acetate, 2:1 to 1:1) to afford *sulfondiimidamide* **11c** as a colourless oil (598 mg, 1.29 mmol, 99%).

R*f* 0.40 (petrol/ethyl acetate, 2:1);

^{1}H NMR (400 MHz, $CDCl_3$): δ (ppm) = 8.34 (d, J = 8.9 Hz, 2H), 8.18 (d, J = 8.9 Hz, 2H), 8.05-7.99 (m, 2H), 7.33-7.26 (m, 2H), 5.68 (ddt, J = 16.9, 10.1, 6.7 Hz, 2H), 5.32-5.21 (m, 4H), 4.05 (dd, J = 14.9, 6.5 Hz, 2H), 3.94 (dd, J = 15.3, 6.8 Hz, 2H);

^{13}C NMR (101 MHz, $CDCl_3$): δ (ppm) = 166.7 (d, J = 261.2 Hz), 150.3, 147.5, 131.4 (d, J = 10.0 Hz), 130.8 (d, J = 3.2 Hz), 130.2, 128.5, 124.4, 122.0, 117.7 (d, J = 23.3 Hz), 109.6, 50.2;

^{19}F NMR (377 MHz, $CDCl_3$): δ (ppm) = -99.2 (tt, J = 7.8, 4.7 Hz);

IR (ATR): $\tilde{\nu}$ (cm^{-1}) = 2206, 1530, 1350, 1216, 1165, 1082, 939, 744;

HRMS (ESI^+) calcd. for $C_{19}H_{19}FN_5O_4S_2^+$ $[M+H]^+$: 464.0857, found: 464.0858.

Sodium *N*-(amino(cyanoimino)(4-fluorophenyl)-λ^6-sulfaneylidene)-4-nitrobenzenesulfonamide (12c)

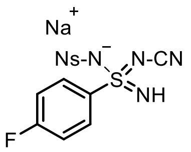

Sulfondiimidamide **11c** (250 mg, 0.54 mmol, 1.00 equiv.), 1,3-dimethylbarbituric acid (505 mg, 3.24 mmol, 6.00 equiv.) and $Pd(PPh_3)_4$ (125 mg, 0.11 mmol, 0.20 equiv.) were dissolved in anhydrous, degassed CH_2Cl_2 (2.70 mL) in an oven-dried 25 mL round-bottom flask a under nitrogen atmosphere. After being stirred at room temperature for 1.5 h, the reaction mixture was diluted with ethyl acetate (40 mL) and quenched with 0.2 M aq. Na_2CO_3 solution (80 mL). The aqueous phase was separated and extracted with ethyl acetate (2 × 40 mL). The combined organic layers were dried over anhydrous Na_2SO_4 and concentrated under reduced pressure. The crude product was purified by flash column chromatography (ethyl acetate/ethanol, 10:1 to 4:1) to afford *sulfondiimidamide salt* **12c** as a colourless oil (90 mg, 0.22 mmol, 41%).

R*f* 0.50 (ethyl acetate/ethanol, 5:1);

^{1}H NMR (400 MHz, $(CD_3)_2SO$): δ (ppm) = 8.21 (d, J = 8.9 Hz, 2H), 7.88 (d, J = 8.9 Hz, 2H), 7.87-7.83 (m, 2H), 7.27-7.21 (m, 2H), 3.99 (s, 1H).

^{13}C NMR (101 MHz, $(CD_3)_2SO$): δ (ppm) = 163.6 (d, J = 249.3 Hz), 150.5, 148.5, 141.2 (d, J = 2.9 Hz), 129.0 (d, J = 9.2 Hz), 127.9, 123.6, 116.3, 115.2 (d, J = 22.6 Hz).

^{19}F NMR (377 MHz, $(CD_3)_2SO$): δ (ppm) = -109.1 (tt, J = 9.5, 5.4 Hz).

IR (ATR): $\tilde{\nu}$ (cm^{-1}) = 2166, 1473, 1383, 1252, 1152, 1056, 1026, 1007, 955.

HRMS (ESI$^-$) calcd. for $C_{13}H_9FN_5O_4S_2^-$ [M-Na]$^-$: 382.0085, found: 382.0082.

***N*-((Diallylamino)(4-fluorophenyl)(((4-nitrophenyl)sulfonyl)imino)-λ^6-sulfaneylidene)-4-(trifluoromethyl)benzamide (11d)**

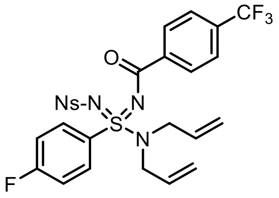

To an oven-dried 25 mL round-bottom flask under nitrogen atmosphere was added sulfondiimidamide **8ag** (285 mg, 0.65 mmol, 1.00 equiv.) and anhydrous CH_2Cl_2 (3.3 mL) at room temperature. Et_3N (0.18 mL, 1.29 mmol, 2.00 equiv.) was then added, followed by the addition of 4-(trifluoromethyl)benzoyl chloride (204 mg, 0.98 mmol, 1.50 equiv.). After being stirred at room temperature for 3 h, the reaction mixture was diluted with CH_2Cl_2 (60 mL) and quenched with sat. aq. NaCl solution (100 mL). The aqueous phase was separated and extracted with CH_2Cl_2 (2 × 40 mL). The combined organic layers were dried over anhydrous Na_2SO_4 and concentrated under reduced pressure. The crude product was purified by flash column chromatography (petrol/ethyl acetate, 3:1 to 2:1) to afford *sulfondiimidamide* **11d** as a white solid (335 mg, 0.55 mmol, 84%).

mp 138-140 °C;

R*f* 0.41 (petrol/ethyl acetate, 3:1);

^{1}H NMR (400 MHz, $CDCl_3$): δ (ppm) = 8.12-8.06 (m, 2H), 7.97 (d, J = 8.9 Hz, 2H), 7.91 (d, J = 8.9 Hz, 2H), 7.83 (d, J = 8.1 Hz, 2H), 7.57 (d, J = 8.1 Hz, 2H), 7.30-7.22 (m, 2H), 5.82 (ddt, J = 16.5, 9.9, 6.6 Hz, 2H), 5.31-5.22 (m, 4H), 4.16 (dd, J = 15.6, 6.6 Hz, 2H), 4.05 (dd, J = 15.6, 6.6 Hz, 2H);

^{13}C NMR (101 MHz, $CDCl_3$): δ (ppm) = 169.6, 166.2 (d, J = 259.2 Hz), 149.5, 147.5, 137.6, 134.3 (q, J = 32.7 Hz), 132.3 (d, J = 3.1 Hz), 131.6, 131.5 (d, J = 9.7 Hz), 129.4, 128.6, 125.2 (q, J = 3.7 Hz), 123.74, 123.68 (q, J = 272.6 Hz), 120.9, 117.3 (d, J = 23.1 Hz), 50.6;

^{19}F NMR (377 MHz, $CDCl_3$): δ (ppm) = -63.1 (s), -101.4 (tt, J = 8.0, 4.9 Hz);

IR (ATR): $\tilde{\nu}$ (cm^{-1}) = 2981, 2888, 1633, 1586, 1528, 1382, 1277, 1249, 1164, 1126, 1065, 949;

HRMS (ESI^+) calcd. for $C_{26}H_{23}F_4N_4O_5S_2^+$ $[M+H]^+$: 611.1041, found: 611.1039.

Sodium *N*-(Amino(4-fluorophenyl)(((4-nitrophenyl)sulfonyl)imino)-λ⁶-sulfaneylidene)-4-(trifluoromethyl)benzamide (12d)

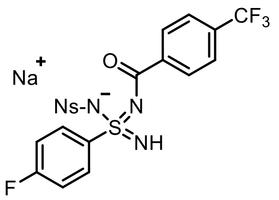

Sulfondiimidamide **11d** (230 mg, 0.38 mmol, 1.00 equiv.), 1,3-dimethylbarbituric acid (356 mg, 2.28 mmol, 6.00 equiv.) and $Pd(PPh_3)_4$ (88 mg, 0.076 mmol, 0.20 equiv.) were dissolved in anhydrous, degassed CH_2Cl_2 (1.90 mL) in an oven-dried 25 mL round-bottom flask under a nitrogen atmosphere. After being stirred at room temperature for 1 h, the reaction mixture was diluted with ethyl acetate (50 mL) and quenched with 0.2 M aq. Na_2CO_3 solution (60 mL). The aqueous phase was separated and extracted with ethyl acetate (2 × 40 mL). The combined organic layers were dried over anhydrous Na_2SO_4 and concentrated under reduced pressure to approximately $1/10^{th}$ of the original volume. At this point, a large amount of solid product precipitated out of solution, which was then separated by filtration and washed with cold CH_2Cl_2 (10 mL) to afford *sulfondiimidamide salt* **12d** as a white solid (131 mg, 0.24 mmol, 62%).

mp 218-220 °C;

$\mathbf{R}_f$ 0.40 (ethyl acetate);

^{1}**H NMR** (400 MHz, $(CD_3)_2SO$): δ (ppm) = 8.10 (d, J = 8.8 Hz, 2H), 8.04-7.98 (m, 2H), 7.91 (d, J = 8.8 Hz, 2H), 7.83 (d, J = 8.1 Hz, 2H), 7.60 (d, J = 8.1 Hz, 2H), 7.29-7.22 (m, 2H), 3.60 (s, 1H);

^{13}**C NMR** (101 MHz, $(CD_3)_2SO$): δ (ppm) = 169.1, 163.4 (d, J = 248.5 Hz), 151.3, 148.1, 141.58, 141.57 (d, J = 3.5 Hz), 130.4 (q, J = 31.5 Hz), 129.4 (d, J = 9.1 Hz), 128.8, 128.1, 124.4 (q, J = 3.6 Hz), 124.2 (q, J = 272.6 Hz), 123.3, 114.8 (d, J = 22.5 Hz);

^{19}**F NMR** (377 MHz, $(CD_3)_2SO$): δ (ppm) = -61.2 (s), -110.0 (tt, J = 8.9, 5.4 Hz);

IR (ATR): $\tilde{\nu}$ (cm^{-1}) = 2981, 2888, 1531, 1462, 1382, 1318, 1251, 1149, 1088, 1068, 1015, 955, 858;

HRMS (ESI^-) calcd. for $C_{20}H_{13}F_4N_4O_5S_2^-$ $[M-Na]^-$: 529.0269, found: 529.0259.

4-Bromo-*N*-((diallylamino)(4-fluorophenyl)(((4-nitrophenyl)sulfonyl)imino)-λ⁶-sulfaneylidene)benzenesulfonamide (11e)

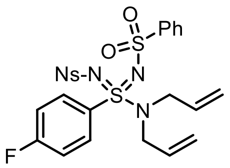

To an oven-dried 25 mL round-bottom flask under nitrogen atmosphere was dissolved sulfondiimidamide **8ag** (300 mg, 0.68 mmol, 1.00 equiv.) in anhydrous CH_2Cl_2 (3.4 mL) at room temperature. Et_3N (0.19 mL, 1.36 mmol, 2.00 equiv.) was then added, followed by the addition of benzenesulfonyl chloride (180 mg, 1.02 mmol, 1.50 equiv.) and DMAP (17 mg, 0.14 mmol, 0.20 equiv.). After being stirred at room temperature for 16 h, the reaction mixture was diluted with CH_2Cl_2 (60 mL) and quenched with sat. aq. NaCl solution (100 mL). The aqueous phase was separated and extracted with CH_2Cl_2 (2 × 50 mL). The combined organic layers were dried over anhydrous Na_2SO_4 and concentrated under reduced pressure. The crude product was purified by flash column chromatography (petrol/ethyl acetate, 2:1 to 1:1) to afford *sulfondiimidamide* **11e** as a pale-yellow oil (390 mg, 0.67 mmol, 99%).

R*f* 0.39 (petrol/ethyl acetate, 2:1);

^{1}H NMR (400 MHz, $CDCl_3$): δ (ppm) = 8.19 (d, J = 8.9 Hz, 2H), 8.00 (d, J = 8.9 Hz, 2H), 7.81-7.74 (m, 2H), 7.57-7.51 (m, 2H), 7.47-7.41 (m, 1H), 7.34-7.27 (m, 2H), 7.10-7.04 (m, 2H), 5.72 (ddt, J = 17.0, 10.3, 6.7 Hz, 2H), 5.26-5.14 (m, 4H), 4.05 (dd, J = 15.4, 6.7 Hz, 2H), 3.95 (dd, J = 15.4, 6.9 Hz, 2H);

^{13}C NMR (101 MHz, $CDCl_3$): δ (ppm) = 166.1(d, J = 259.7 Hz), 149.7, 147.5, 141.9, 132.5, 131.8 (d, J = 9.9 Hz), 131.0, 130.7 (d, J = 3.2 Hz), 128.6, 128.3, 126.3, 123.8, 121.3, 116.8 (d, J = 23.1 Hz), 50.5;

^{19}F NMR (377 MHz, $CDCl_3$): δ (ppm) = -100.7 (tt, J = 8.0, 5.0 Hz);

IR (ATR): $\tilde{\nu}$ (cm^{-1}) = 1589, 1529, 1489, 1350, 1327, 1308, 1167, 1068;

HRMS (ESI^+) calcd. for $C_{24}H_{24}FN_4O_6S_3^+$ $[M+H]^+$: 579.0837, found: 579.0830.

Sodium *N*-(Amino(((4-bromophenyl)sulfonyl)imino)(4-fluorophenyl)-λ^{6}-sulfaneylidene)-4-nitrobenzenesulfonamide (12e)

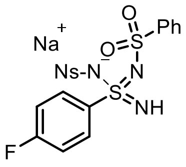

Sulfondiimidamide **11e** (355 mg, 0.61 mmol, 1.00 equiv.), 1,3-dimethylbarbituric acid (571 mg, 3.66 mmol, 6.00 equiv.), $Pd(PPh_3)_4$ (141 mg, 0.12 mmol, 0.20 equiv.) were dissolved in anhydrous, degassed CH_2Cl_2 (3.00 mL) in an oven-dried 25 mL round-bottom flask under a nitrogen atmosphere. After being stirred at room temperature for 3 h, the reaction mixture was diluted with ethyl acetate (50 mL) and quenched with 0.2 M aq. Na_2CO_3 solution (60 mL). The aqueous phase was separated and extracted with ethyl acetate (3 × 40 mL). The combined organic layers were dried over anhydrous Na_2SO_4 and concentrated under reduced pressure. The crude product was purified by flash column chromatography (ethyl acetate/ethanol, 1:0 to 20:1 to 10:1) to afford *sulfondiimidamide salt* **12e** as a pale-yellow oil (160 mg, 0.31 mmol, 50%).

R*f* 0.50 (ethyl acetate/ethanol, 10:1);

^{1}H NMR (400 MHz, $(CD_3)_2SO$): *δ* (ppm) = 8.16 (d, *J* = 8.8 Hz, 2H), 7.82 (d, *J* = 8.8 Hz, 2H), 7.79-7.74 (m, 2H), 7.57-7.53 (m, 2H), 7.43-7.38 (m, 1H), 7.35-7.29 (m, 2H), 7.16-7.10 (m, 2H), 3.56 (s, 1H);

^{13}C NMR (101 MHz, $(CD_3)_2SO$): *δ* (ppm) = 163.3 (d, *J* = 248.8 Hz), 151.0, 148.2, 145.0, 141.4 (d, *J* = 2.7 Hz), 130.6, 129.2 (d, *J* = 9.3 Hz), 128.0, 127.9, 126.1, 123.3, 114.7 (d, *J* = 22.5 Hz);

^{19}F NMR (377 MHz, $(CD_3)_2SO$): *δ* (ppm) = -109.8 (tt, *J* = 9.2, 5.4 Hz);

IR (ATR): $\tilde{\nu}$ (cm^{-1}) = 1588, 1528, 1491, 1350, 1283, 1149, 1087, 1029, 1008, 996;

HRMS (ESI^+) calcd. For $C_{18}H_{14}FN_4O_6S_3^-$ $[M-Na]^-$: 497.0065, found: 497.0064.

1.2.4 The Synthesis of a Sulfondiimidamide Analogue of Celecoxib

N-(Amino(4-(5-(p-tolyl)-3-(trifluoromethyl)-1H-pyrazol-1-yl)phenyl)-λ⁴-sulfaneylidene)-cyanamide (14)

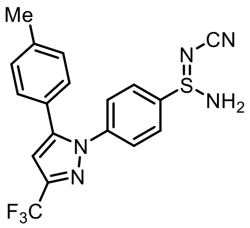

Preparation of organometallic reagent

(4-(5-(*p*-Tolyl)-3-(trifluoromethyl)-1*H*-pyrazol-1-yl)phenyl)lithium solution was prepared according to the following procedure. To a solution of 1-(4-bromophenyl)-5-(*p*-tolyl)-3-(trifluoromethyl)-1*H*-pyrazole **13** (6.84 g, 18.00 mmol, 1.20 equiv.) in anhydrous THF (36.0 mL) in an oven-dried 250 mL round-bottom flask was added *n*-butyllithium solution (7.20 mL, 2.50 M in hexanes, 18.00 mmol, 1.20 equiv.) dropwise at -78 °C under a nitrogen atmosphere. The reaction was stirred at the same temperature for 40 min.

Preparation of sulfinamidine

TIPS-NSO (3.29 g, 15.00 mmol, 1.00 equiv.) was dissolved in anhydrous THF (30.0 mL) in an oven-dried 250 mL round-bottom flask under a nitrogen atmosphere. The mixture was cooled to -30 °C before LiHMDS solution (15.00 mL, 1.00 M in THF, 15.00 mmol, 1.00 equiv.) was added. After being stirred at - 30 °C for 5 min, the reaction was warmed to 0 °C and stirred for another 5 min. TMSCl (1.90 mL, 15.00 mmol, 1.00 equiv.) was added and the reaction was stirred at 0 °C for 10 min. Then the reaction solution was added to the (4-(5-(*p*-tolyl)-3-(trifluoromethyl)-1*H*-pyrazol-1-yl)phenyl)lithium solution (18.00 mmol, 1.20 equiv.) at - 78 °C and stirred at 0 °C for 10 min. The reaction mixture was then diluted with ethyl acetate (150 mL) and quenched with sat. aq. tetrasodium EDTA solution (300 mL). The aqueous layer was further extracted with ethyl acetate (2 × 80 mL). The combined organic layers were dried over anhydrous Na_2SO_4, filtered and concentrated under reduced pressure. This crude mixture was then dissolved in anhydrous CH_2Cl_2 (50.0 mL) in an oven-dried 250 mL round-bottom flask and cooled to 0 °C. Then Et_3N (2.51 mL, 18.00 mmol, 1.20 equiv.) and BrCN solution (3.00 mL, 5.0 M in CH_3CN, 15.00 mmol, 1.00 equiv.) were added. The reaction was stirred at 0 °C for 20 min prior to the addition of TBAF solution (16.50 mL, 1.00 M in THF, 16.50 mmol, 1.10 equiv.). The reaction was stirred at 0 °C for another 10 min until completion (judged

by TLC). The reaction was then diluted with ethyl acetate (150 mL) and quenched with sat. aq. $NaHCO_3$ solution (250 mL). The aqueous layer was further extracted with ethyl acetate (2 × 80 mL). The combined organic layers were dried over anhydrous Na_2SO_4, filtered, concentrated under reduced pressure and purified by flash column chromatography (CH_2Cl_2/ethyl acetate, 2:1 to 1:1 to 1:2) to afford *sulfinamidine* **14** as a pale-yellow oil (5.03 g, 12.93 mmol, 86%).

R*f* 0.50 (CH_2Cl_2/ethyl acetate, 1:1);

^{1}H NMR (400 MHz, $(CD_3)_2SO$): δ (ppm) = 7.88 (d, J = 8.8 Hz, 2H), 7.61 (d, J = 8.8 Hz, 2H), 7.23-7.16 (m, 7H), 2.31 (s, 3H);

^{13}C NMR (101 MHz, $(CD_3)_2SO$): δ (ppm) = 145.3, 142.3 (q, J = 37.7 Hz), 141.4, 139.1, 138.8, 129.5, 128.8, 128.2, 126.3, 125.4, 121.3 (q, J = 268.6 Hz), 119.7, 106.2, 20.8;

^{19}F NMR (377 MHz, $(CD_3)_2SO$): δ (ppm) = -60.9 (s);

IR (ATR): $\tilde{\nu}$ (cm^{-1}) = 2152, 1496, 1472, 1374, 1236, 1161, 1134, 1097, 975;

HRMS (ESI$^+$) calcd. for $C_{18}H_{15}F_3N_5S^+$ [M+H]$^+$: 390.0995, found: 390.0988.

***N,N*-Diallyl-*N'*-cyano-4-(5-(*p*-tolyl)-3-(trifluoromethyl)-1*H*-pyrazol-1-yl)benzenesulfondiimidamide (15)**

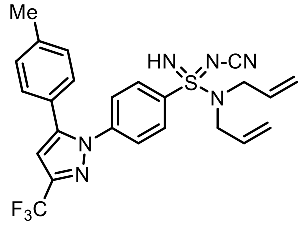

An oven-dried 25 mL round-bottom flask containing primary sulfinamidine **14** (105 mg, 0.27 mmol, 1.00 equiv.) and $PhI(OAc)_2$ (130 mg, 0.40 mmol, 1.50 equiv.) was sealed and subjected to three N_2 evacuation/refill cycles before anhydrous toluene (1.35 mL) was added. Et_3N (113 µL, 0.81 mmol, 3.00 equiv.) was added to the solution, followed immediately by the addition of diallyl amine (130 mg, 1.34 mmol, 5.00 equiv.). After being stirred at room temperature for 40 min, the reaction mixture was diluted with ethyl acetate (30 mL) and quenched with sat. aq. NaCl solution (50 mL). The aqueous layer was further extracted with ethyl acetate (2 × 30 mL). The combined organic layers were dried over anhydrous Na_2SO_4 and concentrated under reduced pressure. The crude product was purified by flash column chromatography (petrol/ethyl acetate, 2:1 to 1:1) to afford *sulfondiimidamide* **15** as a pale-yellow oil (106 mg, 0.22 mmol, 81%).

R*f* 0.40 (petrol/ethyl acetate, 3:2);

^{1}H NMR (400 MHz, CD_3CN): δ (ppm) = 8.03 (d, J = 8.9 Hz, 2H), 7.52 (d, J = 8.9 Hz, 2H), 7.21 (d, J = 8.2 Hz, 2H), 7.17 (d, J = 8.2 Hz, 2H), 6.93 (s, 1H), 5.65 (ddt, J = 16.6, 10.2, 6.3 Hz, 2H), 5.28-5.12 (m, 4H), 3.94 (ddt, J = 15.0, 6.1, 1.7 Hz, 2H), 3.87 (ddt, J = 15.9, 6.4, 1.7 Hz, 2H), 3.82 (s, 1H), 2.35 (s, 3H);

^{13}C NMR (101 MHz, CD_3CN): δ (ppm) = 146.7, 144.2 (q, J = 37.8 Hz), 144.1, 140.9, 139.2, 133.2, 130.4, 129.9, 129.5, 127.1, 126.7, 122.5 (q, J = 268.2 Hz), 120.1, 114.0, 107.1, 50.7, 21.3;

^{19}F NMR (377 MHz, CD_3CN): δ (ppm) = -62.9 (s);

IR (ATR): $\tilde{\nu}$ (cm^{-1}) = 2188, 1495, 1471, 1373, 1235, 1160, 1133, 1097, 974, 808;

HRMS (ESI^+) calcd. for $C_{24}H_{24}F_3N_6S^+$ $[M+H]^+$: 485.1730, found: 485.1729.

Data for this compound was consistent with previous reports.[3]

1.2.5 The Synthesis of a Sulfondiimidamide Analogue of Sildenafil

N-(amino(4-ethoxy-3-(1-methyl-7-oxo-3-propyl-6,7-dihydro-1*H*-pyrazolo[4,3-*d*]pyrimidin-5-yl)phenyl)- λ^4-sulfaneylidene)-cyanamide (18)

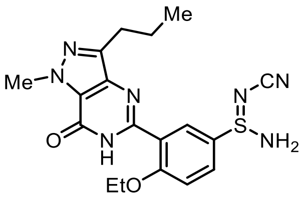

Preparation of organometallic reagent

(4-Ethoxy-3-(1-methyl-7-oxo-3-propyl-6,7-dihydro-1*H*-pyrazolo[4,3-*d*]pyrimidin-5-yl)-phenyl) lithium solution was prepared according to the following procedure. To a solution of 5-(5-Bromo-2-ethoxyphenyl)-1-methyl-3-propyl-1*H*-pyrazolo[4,3-*d*]pyrimidin-7(6H)-one [4] **17** (3.90 g, 10.00 mmol, 1.00 equiv.) in anhydrous THF (50.0 mL) in an oven-dried 250 mL round-bottom flask was added methyllithium solution (7.50 mL, 1.60 M in Et_2O, 12.00 mmol, 1.20 equiv.) dropwise at -78 °C under a nitrogen atmosphere. The reaction was stirred at the same temperature for 10 min. Then *n*-butyllithium solution (6.00 mL, 2.50 M in hexanes, 15.00 mmol, 1.50 equiv.) was added dropwise at -78 °C. The reaction was stirred at the same temperature for 40 min.

Preparation of sulfinamidine

TIPS-NSO (4.38 g, 20.00 mmol, 2.00 equiv.) was dissolved in anhydrous THF (40.0 mL) in an oven-dried 250 mL round-bottom flask and was purged with nitrogen gas. The mixture was cooled to -30 °C before LiHMDS solution (20.00 mL, 1.00 M in THF, 20.00 mmol, 2.00 equiv.) was added. After being stirred at - 30 °C for 5 min, the reaction was warmed to 0 °C and stirred for another 5 min. TMSCl (2.54 mL, 20.00 mmol, 2.00 equiv.) was added and the reaction was stirred at 0 °C for 10 min. Then the reaction solution was added to the (4-ethoxy-3-(1-methyl-7-oxo-3-propyl-6,7-dihydro-1*H*-pyrazolo[4,3-*d*]pyrimidin-5-yl)-phenyl) lithium solution (10.00 mmol, 1.00 equiv.) at - 78 °C and stirred at 0 °C for 10 min. The reaction mixture was then diluted with ethyl acetate (200 mL) and quenched with sat. aq. tetrasodium EDTA solution (400 mL). The aqueous layer was further extracted with ethyl acetate (2 × 100 mL). The combined organic layers were dried over anhydrous Na_2SO_4, filtered and concentrated under reduced pressure. This crude mixture was then dissolved in anhydrous CH_2Cl_2 (50.0 mL) in an oven-dried 250 mL round-bottom flask and cooled to 0 °C. Then Et_3N (2.09 mL, 15.00 mmol, 1.50 equiv.) and BrCN solution (2.40 mL, 5.0 M in CH_3CN, 12.00 mmol, 1.20 equiv.) were added. The reaction was stirred at 0 °C for 20 min prior to the addition of TBAF

solution (15.00 mL, 1.00 M in THF, 15.00 mmol, 1.50 equiv.) at the same temperature. The reaction was stirred for another 10 min and resulted in the precipitation of a white solid (primary sulfinamidine **18**). This white solid was filtered and washed with cold CH_2Cl_2 (30 mL) to give analytically pure product **18**. Sat. aq. NaCl solution (200 mL) was added to the combined filtrates. The aqueous phase was extracted with CH_2Cl_2 (2 × 80 mL). The combined organic layers were dried over anhydrous Na_2SO_4, filtered, concentrated under reduced pressure and purified by flash column chromatography. Filtration and flash column chromatography (ethyl acetate/ethanol, 50:1 to 5:1) afforded the desired product **18** as a white solid (2.03 g, 5.09 mmol, 51%).

mp 162-164 °C;

R*f* 0.50 (ethyl acetate/methanol, 25:1);

^{1}H NMR (400 MHz, $(CD_3)_2SO$): *δ* (ppm) = 12.19 (s, 1H), 7.98 (d, *J* = 2.6 Hz, 1H), 7.88 (dd, *J* = 8.9, 2.6 Hz, 1H), 7.40 (d, *J* = 9.0 Hz, 1H), 7.07 (s, 2H), 4.20 (q, *J* = 7.0 Hz, 2H), 4.16 (s, 3H), 2.78 (t, *J* = 7.5 Hz, 2H), 1.79-1.68 (m, 2H), 1.34 (t, *J* = 6.9 Hz, 3H), 0.93 (t, *J* = 7.4 Hz, 3H).

^{13}C NMR (101 MHz, $(CD_3)_2SO$): *δ* (ppm) = 159.0, 153.8, 148.4, 145.0, 137.8, 130.6, 129.6, 129.1, 124.4, 123.7, 119.9, 113.7, 64.8, 37.9, 27.1, 21.7, 14.3, 13.8.

IR (ATR): $\tilde{\nu}$ (cm^{-1}) = 2154, 2030, 1686, 1463, 1392, 1248, 1152, 1078, 954;

HRMS (ESI$^+$) calcd. for $C_{18}H_{22}N_7O_2S^+$ [M+H]$^+$: 400.1550, found: 400.1550.

***N*-((4-Ethoxy-3-(1-methyl-7-oxo-3-propyl-6,7-dihydro-1*H*-pyrazolo[4,3-*d*]pyrimidin-5-yl)phenyl)(imino)(4-methylpiperazin-1-yl)-λ^6-sulfaneylidene)cyanamide (19)**

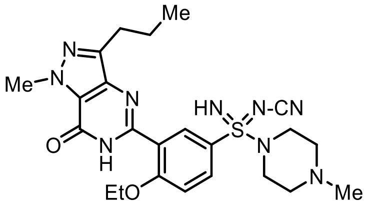

An oven-dried 25 mL round-bottom flask containing primary sulfinamidine **18** (399 mg, 1.00 mmol, 1.00 equiv.) and $PhI(OAc)_2$ (644 mg, 2.00 mmol, 2.00 equiv.) was sealed and subjected to three N_2 evacuation/refill cycles before pre-sparged anhydrous CH_3CN (5.0 mL) was added. DBU (0.30 mL, 2.0 mmol, 2.00 equiv.) was added to the solution, followed immediately by the addition of *N*-methyl piperazine (401 mg, 4.00 mmol, 4.00 equiv.). After being stirred at room temperature for 30 min (judged by TLC), the reaction mixture was diluted with ethyl acetate (60 mL) and quenched with sat.

aq. NaCl solution (100 mL). The aqueous layer was further extracted with ethyl acetate (2 × 50 mL). The combined organic layers were dried over anhydrous Na_2SO_4 and concentrated under reduced pressure. The crude product was purified by flash column chromatography (ethyl acetate/ethanol, 4:1 to 1:1 to 1:4 to 1:100 with 2% Et_3N) to afford *sulfondiimidamide* **19** as a pale-yellow oil (306 mg, 0.62 mmol, 62%).

R*f* 0.18 (ethyl acetate/ethanol, 1:5);

^{1}H NMR (400 MHz, CD_3CN): δ (ppm) = 8.57 (d, *J* = 2.6 Hz, 1H), 7.96 (dd, *J* = 8.9, 2.6 Hz, 1H), 7.81-4.34 (br. s, 2H), 7.26 (d, *J* = 9.0 Hz, 1H), 4.26 (q, *J* = 6.9 Hz, 2H), 4.08 (s, 3H), 3.07 (app. s, 4H), 2.78 (t, *J* = 7.4 Hz, 2H), 2.37 (app. s, 4H), 2.14 (s, 3H), 1.85-1.66 (m, 2H), 1.45 (t, *J* = 6.9 Hz, 3H), 0.94 (t, *J* = 7.3 Hz, 3H);

^{13}C NMR (101 MHz, CD_3CN): δ (ppm) = 161.4, 154.3, 148.0, 146.8, 138.9, 132.8, 131.5, 127.0, 125.4, 122.9, 114.8, 114.3, 67.1, 54.8, 47.4, 45.7, 38.6, 28.4, 22.8, 14.8, 14.4;

IR (ATR): $\tilde{\nu}$ (cm^{-1}) = 2187, 1689, 1596, 1489, 1467, 1394, 1277, 1219, 1128, 1028, 908, 726;

HRMS (ESI^+) calcd. for $C_{23}H_{32}N_9O_2S^+$ $[M+H]^+$: 498.2394, found: 498.2388.

1.2.6 The Synthesis of a Sulfondiimidamide Analogue of Tasisulam Sodium

5-Bromo-*N'*-cyanothiophene-2-primary sulfinamidine (20)

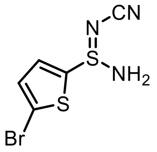

Preparation of LDA reagent

Diisopropylamine (1.35 mL, 9.60 mmol, 1.20 equiv.) was dissolved in anhydrous THF (4.80 mL) in an oven-dried 50 mL round-bottom flask under a nitrogen atmosphere. The mixture was cooled to -78 °C before *n*-butyllithium solution (4.27 mL, 2.25 M in hexane, 9.60 mmol, 1.20 equiv.) was added dropwise. After being stirred at -78 °C for 5 min, the reaction was warmed to 0 °C and stirred for another 20 min.

Preparation of organometallic reagent

(5-Bromothiophen-2-yl)lithium solution was prepared according to the following procedure. 2-bromothiophene (1.56 g, 9.60 mmol, 1.20 equiv.) was dissolved in anhydrous THF (19.2 mL) in an oven-dried 250 mL round-bottom flask under a nitrogen atmosphere. The mixture was cooled to -78 °C before LDA solution (9.60 mmol, 1.20 equiv.) was added dropwise. The reaction was stirred at the same temperature for 30 min.

Preparation of sulfinamidine

TIPS-NSO (1.75 g, 8.00 mmol, 1.00 equiv.) was dissolved in anhydrous THF (16.0 mL) in an oven-dried 100 mL round-bottom flask under a nitrogen atmosphere. The mixture was cooled to -30 °C before LiHMDS solution (8.00 mL, 1.00 M in THF, 8.00 mmol, 1.00 equiv.) was added. After being stirred at - 30 °C for 5 min, the reaction was warmed to 0 °C and stirred for another 5 min. TMSCl (1.02 mL, 8.00 mmol, 1.00 equiv.) was added and the reaction was stirred at 0 °C for 10 min. Then the reaction solution was added to the (5-bromothiophen-2-yl)lithium solution (9.60 mmol, 1.20 equiv.) at - 78 °C and stirred at 0 °C for 10 min. The reaction mixture was then diluted with ethyl acetate (150 mL) and quenched with sat. aq. tetrasodium EDTA solution (200 mL). The aqueous layer was further extracted with ethyl acetate (2 × 60 mL). The combined organic layers were dried over anhydrous Na_2SO_4, filtered and concentrated under reduced pressure. This crude mixture was then dissolved in anhydrous CH_2Cl_2 (40.0 mL) in an oven-dried 250 mL round-bottom flask and cooled

to 0 °C. Then Et_3N (1.34 mL, 9.60 mmol, 1.20 equiv.) and BrCN solution (1.60 mL, 5.0 M in CH_3CN, 8.00 mmol, 1.00 equiv.) were added. The reaction was stirred at 0 °C for 20 min prior to the addition of TBAF solution (8.80 mL, 1.00 M in THF, 8.80 mmol, 1.10 equiv.). The reaction was stirred at 0 °C for another 10 min until completion (judged by TLC). The reaction was then diluted with ethyl acetate (150 mL) and quenched with sat. aq. $NaHCO_3$ solution (250 mL). The aqueous layer was further extracted with ethyl acetate (2 × 80 mL). The combined organic layers were dried over anhydrous Na_2SO_4, filtered, concentrated under reduced pressure to approximately 1/20th of the original volume. At this point, a large amount of solid product (primary sulfinamidine **20**) precipitated out of solution, which was then separated by filtration and washed with cold CH_2Cl_2 (30 mL) to give analytically pure sulfinamidine **20**. The filtrate was concentrated under reduced pressure and resulted in the precipitation of a white solid (primary sulfinamidine **20**) again, which was then separated by filtration again. Two times of filtration afforded the desired product **20** as a white solid (1.20 g, 4.82 mmol, 60%).

mp 114-116 °C;

R*f* 0.50 (ethyl acetate);

^{1}H NMR (400 MHz, $(CD_3)_2SO$): δ (ppm) = 7.43 (d, J = 4.1 Hz, 1H), 7.43 (s, 2H), 7.36 (d, J = 4.1 Hz, 1H);

^{13}C NMR (101 MHz, $(CD_3)_2SO$): δ (ppm) = 140.5, 132.7, 132.4, 119.3, 118.5;

IR (ATR): $\tilde{\nu}$ (cm^{-1}) =2157, 1473, 1383, 1252, 1153, 1073, 955;

HRMS (ESI$^+$) calcd. for $C_5H_5{}^{79}BrN_3S_2{}^+$ [M+H]$^+$: 249.9103, found: 249.9102; calcd. for $C_5H_5{}^{81}BrN_3S_2{}^+$ [M+H]$^+$: 251.9081, found: 251.9081.

***N,N*-Diallyl-*N'*-5-bromothiophene-2-sulfondiimidamide (21)**

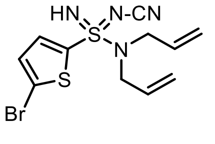

An oven-dried 100 mL round-bottom flask containing primary sulfinamidine **20** (747 mg, 3.00 mmol, 1.00 equiv.) and $PhI(OAc)_2$ (1.45 g, 4.50 mmol, 1.50 equiv.) was sealed and subjected to three N_2 evacuation/refill cycles before anhydrous toluene (15.0 mL) was added. Et_3N (1.25 mL, 9.00 mmol,

3.00 equiv.) was added to the solution, followed immediately by the addition of diallylamine (1.46 g, 15.00 mmol, 5.00 equiv.). After being stirred at room temperature for 30 min, the crude product was purified by flash column chromatography (petrol/ethyl acetate, 2:1 to 1:1) to afford *sulfondiimidamide* **21** as a pale-yellow oil (830 mg, 2.41 mmol, 80%).

R*f* 0.25 (petrol/ethyl acetate, 2:1);

^{1}H NMR (400 MHz, $(CD_3)_2SO$): δ (ppm) = 7.65 (d, J = 4.1 Hz, 1H), 7.42 (d, J = 4.2 Hz, 1H), 5.91 (s, 1H), 5.70 (ddt, J = 16.6, 10.1, 6.2 Hz, 2H), 5.29-5.16 (m, 4H), 3.96 (dd, J = 15.6, 6.1 Hz, 2H), 3.83 (dd, J = 15.5, 6.4 Hz, 2H);

^{13}C NMR (101 MHz, $(CD_3)_2SO$): δ (ppm) = 139.9, 133.4, 132.3, 132.1, 120.6, 119.5, 112.9, 49.9;

IR (ATR): $\tilde{\nu}$ (cm^{-1}) = 2361, 2341, 2188, 1660, 1642, 1399, 1222, 1174, 968, 931;

HRMS (ESI$^+$) calcd. for $C_{11}H_{14}{}^{79}BrN_4S_2^+$ [M+H]$^+$: 344.9838, found: 344.9837; calcd. for $C_{11}H_{14}{}^{81}BrN_4S_2^+$ [M+H]$^+$: 346.9816, found: 346.9813.

***N*-((5-Bromothiophen-2-yl)(cyanoimino)(diallylamino)-λ^6-sulfaneylidene)-2,4-dichlorobenzamide (22)**

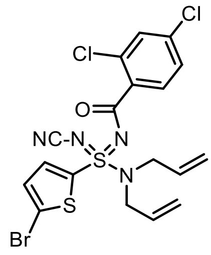

To an oven-dried 50 mL round-bottom flask was added sulfondiimidamide **21** (820 mg, 2.38 mmol, 1.00 equiv.) and anhydrous CH_2Cl_2 (12.0 mL) at room temperature. Et_3N (0.66 mL, 4.73 mmol, 2.00 equiv.) was then added, followed by the addition of 2,4-dichlorobenzoyl chloride (743 mg, 3.57 mmol, 1.50 equiv.) and DMAP (58.2 mg, 0.48 mmol, 0.20 equiv.). After being stirred at room temperature for 15 min, the reaction mixture was diluted with CH_2Cl_2 (60 mL) and quenched with sat. aq. NaCl solution (150 mL). The aqueous layer was further extracted with CH_2Cl_2 (2 × 50 mL). The combined organic layers were dried over anhydrous Na_2SO_4 and concentrated under reduced pressure. The crude product was purified by flash column chromatography (petrol/ethyl acetate, 2:1 to 1:1) to afford *sulfondiimidamide* **22** as a colourless oil (1.22 g, 2.36 mmol, 99%)

R*f* 0.59 (petrol/ethyl acetate, 1:1);

^{1}H NMR (400 MHz, $CDCl_3$): δ (ppm) = 7.89 (d, J = 8.4 Hz, 1H), 7.62 (d, J = 4.2 Hz, 1H), 7.43 (d, J = 2.1 Hz, 1H), 7.28 (dd, J = 8.4, 2.0 Hz, 1H), 7.20 (d, J = 4.2 Hz, 1H), 5.79 (ddt, J = 16.8, 10.2, 6.6 Hz, 2H), 5.35-5.24 (m, 4H), 4.12 (dd, J = 14.7, 6.4 Hz, 2H), 3.98 (dd, J = 15.4, 6.8 Hz, 2H);

^{13}C NMR (101 MHz, $CDCl_3$): δ (ppm) = 170.2, 137.9, 135.4, 134.9, 134.2, 132.9, 132.7, 132.1, 130.9, 130.7, 127.1, 125.3, 121.6, 110.6, 50.3.

IR (ATR): $\tilde{\nu}$ (cm^{-1}) = 2361, 2202, 1660, 1652, 1582, 1394, 1282, 1241, 1139, 1102, 1054, 885, 858;

HRMS (ESI^+) calcd. for $C_{18}H_{15}{}^{79}BrCl_2N_4OS_2^+$ $[M+H]^+$: 516.9320, found: 516.9323; calcd. for $C_{18}H_{15}{}^{81}BrCl_2N_4OS_2^+$ $[M+H]^+$: 518.9296, found: 518.9296.

Sodium *N*-(Amino(5-bromothiophen-2-yl)(cyanoimino)-λ^6-sulfaneylidene)-2,4-dichlorobenzamide (23)

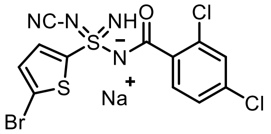

Sulfondiimidamide **22** (530 mg, 1.03 mmol, 1.00 equiv.), 1,3-dimethylbarbituric acid (964 mg, 6.18 mmol, 6.00 equiv.) and $Pd(PPh_3)_4$ (237 mg, 0.21 mmol, 0.20 equiv.) were dissolved in anhydrous, degassed CH_2Cl_2 (5.50 mL) in an oven-dried 25 mL round-bottom flask under a nitrogen atmosphere. After being stirred at room temperature for 1 h, the reaction mixture was diluted with ethyl acetate (60 mL) and quenched with 0.2 M aq. Na_2CO_3 solution (100 mL). The aqueous layer was further extracted with ethyl acetate (2 × 40 mL). The combined organic layers were dried over anhydrous Na_2SO_4 and concentrated under reduced pressure. The crude product was purified by flash column chromatography (ethyl acetate/ethanol, 1:0 to 4:1) to afford *sulfondiimidamide salt* **23** as a colourless oil (284 mg, 0.62 mmol, 60%)

R*f* 0.50 (ethyl acetate/ethanol, 4:1);

^{1}H NMR (400 MHz, $(CD_3)_2SO$): δ (ppm) = 7.65 (d, J = 8.3 Hz, 1H), 7.53 (d, J = 2.1 Hz, 1H), 7.44-7.38 (m, 2H), 7.22 (d, J = 4.0 Hz, 1H), 4.07 (s, 1H);

^{13}C NMR (101 MHz, $(CD_3)_2SO$): δ (ppm) = 171.1, 149.0, 137.2, 134.0, 131.8, 131.5, 130.4, 130.3, 129.4, 126.9, 116.7, 116.4;

IR (ATR): $\tilde{\nu}$ (cm^{-1}) = 2361, 2185, 1584, 1472, 1381, 1330, 1251, 1156, 1053, 967;

HRMS (ESI^+) calcd. for $C_{12}H_6{}^{79}BrCl_2N_4OS_2^-$ [M-Na]$^-$: 434.8549, found: 434.8547; calcd. for $C_{12}H_6{}^{81}BrCl_2N_4OS_2^-$ [M-Na]$^-$: 436.8524, found: 436.8520.

2. Aqueous Stability Study

Table S1. Aqueous stability studies of representative sulfondiimidamides

Compound	$(CD_3)_2SO$	$(CD_3)_2SO$ & pH 1 buffer 10:1 (v/v)	$(CD_3)_2SO$ & pH 7 buffer 10:1 (v/v)	$(CD_3)_2SO$ & pH 10 buffer 10:1 (v/v)
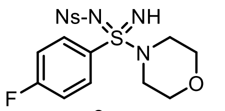 **8a**	6h, 100%<br>24h, 100%<br>48h, 97%<br>72h, 93%	6h, 100%<br>24h, 83%<br>48h, 67%<br>72h, 56%	6h, 96%<br>24h, 85%<br>48h, 69%<br>72h, 58%	6h, 92%<br>24h, 80%<br>48h, 65%<br>72h, 55%
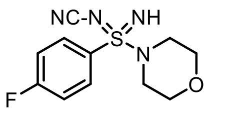 **8p**	6h, 100%<br>24h, 93%<br>48h, 87%<br>72h, 83%	6h, 88%<br>24h, 61%<br>48h, 38%<br>72h, 25%	6h, 89%<br>24h, 60%<br>48h, 38%<br>72h, 25%	6h, 87%<br>24h, 60%<br>48h, 38%<br>72h, 25%
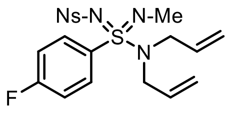 **11b**	6h, 100%<br>24h, 100%<br>48h, 100%<br>72h, 100%	6h, 100%<br>24h, 100%<br>48h, 100%<br>72h, 100%	6h, 100%<br>24h, 100%<br>48h, 100%<br>72h, 100%	6h, 100%<br>24h, 100%<br>48h, 100%<br>72h, 100%
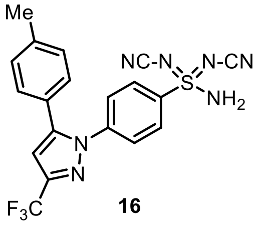 **16**	6h, 100%<br>24h, 100%<br>48h, 100%<br>72h, 100%	6h, 100%<br>24h, 100%<br>48h, 100%<br>72h, 100%	6h, 100%<br>24h, 100%<br>48h, 100%<br>72h, 100%	6h, 100%<br>24h, 100%<br>48h, 100%<br>72h, 100%

Test solutions were prepared by adding 0.05 mmol of test compounds to $(CD_3)_2SO$ (0.7 mL) or a mixture of $(CD_3)_2SO$ (1.0 mL) with different buffer solutions (0.1 mL) in NMR tubes. Pentafluorobenzene (5 mg) was added as an internal standard. Hydrolytic stabilities were measured as recovery (%) of test compounds after 6 h, 24 h, 48 h and 72 h by quantitative ^{19}F NMR.

Mettler Toledo InLab Micro pH-electrode was used to measure pH. The pH meter was calibrated with standard buffers (Fisher Scientific, Pittsburgh, PA), i.e., pH 4.00±0.02 (0.05 M potassium biphthalate), pH 7.00±0.02 (0.05 M potassium phosphate monobasic–sodium hydroxide), pH 10.00±0.02 (0.05 M potassium carbonate–potassium borate–potassium hydroxide).

A pH 1 HCl-buffer solution was prepared by combining a 0.2 M potassium chloride solution (2.5 mL) with 0.2 M hydrochloric acid (6 mL). Final pH adjustments were made with 0. 1 M sodium hydroxide solution or Milli-Q water as necessary. A pH 7 phosphate buffer solution was prepared by combining a 0.2 M Potassium dihydrogen phosphate solution (5.0 mL) with 0.2 M sodium hydroxide solution

(3.0 mL). Final pH adjustments were made with Milli-Q water as necessary. A pH 10 glycine-sodium hydroxide buffer solution was prepared by combining 60 mg glycine with 20 mg sodium hydroxide in 8 mL Milli-Q water. Final pH adjustments were made with Milli-Q water as necessary.

3. References

1 Ding, M.; Zhang, Z. -X.; Davies, T. Q.; Willis, M. C., A silyl sulfinylamine reagent enables the modular synthesis of sulfonimidamides via primary sulfinamides. *Org. Lett.* **2022**, *24*, 1711-1715.

2 Love, B. E.; Jones, E. G., The use of salicylaldehyde phenylhydrazone as an indicator for the titration of organometallic reagents. *J. Org. Chem*. **64(10)**, 3755-3756.

3 Zhang, Z. -X.; Willis, M. C., Sulfondiimidamides as new functional groups for synthetic and medicinal chemistry. *Chem* **2022**, *8*, 1137-1146.

4 Shavnya, A.; Coffey, S. B.; Smith, A. C.; Mascitti, V., Palladium-catalyzed sulfination of aryl and heteroaryl halides: direct access to sulfones and sulfonamides. *Org. Lett*. **15**, 6226–6229.

4. NMR Spectra

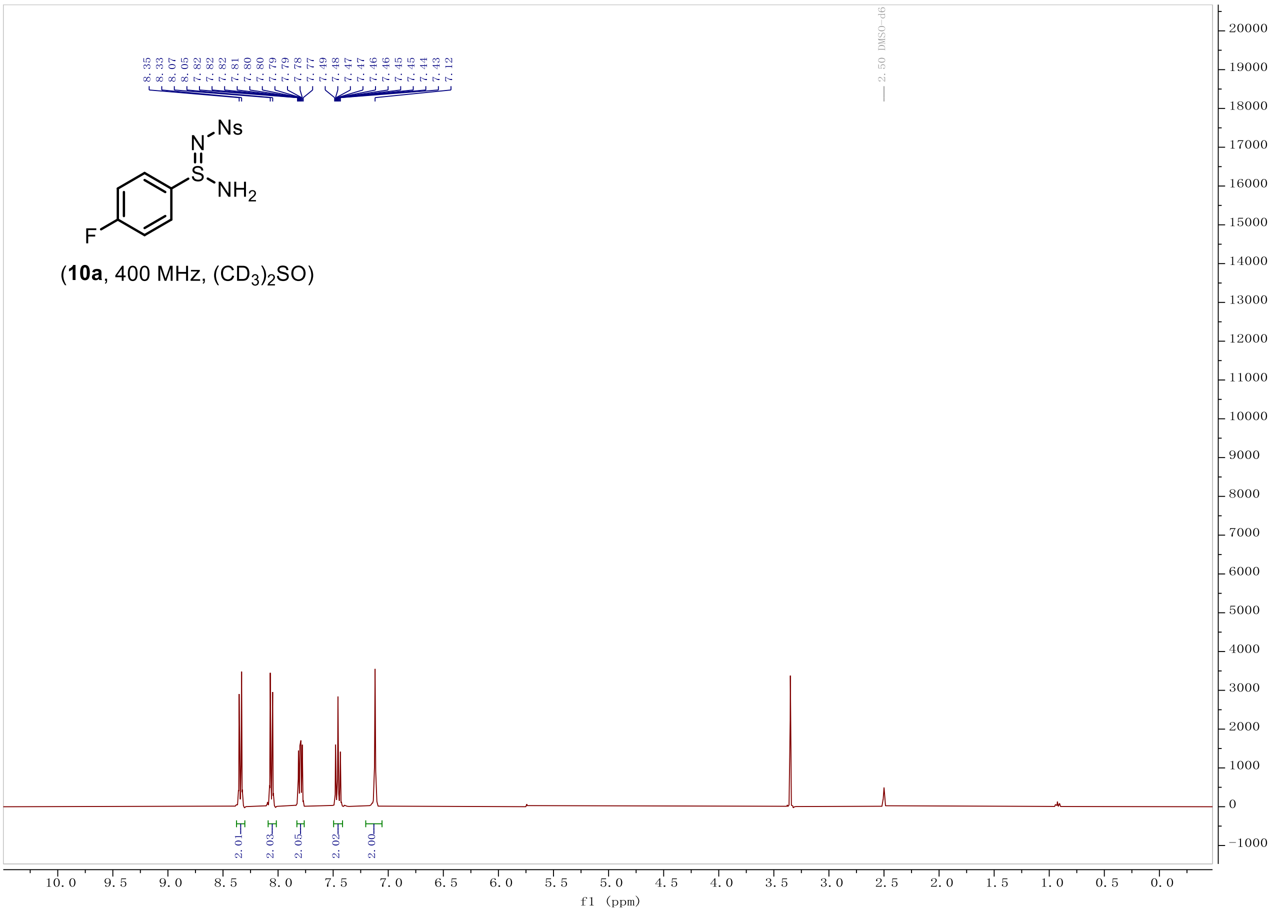

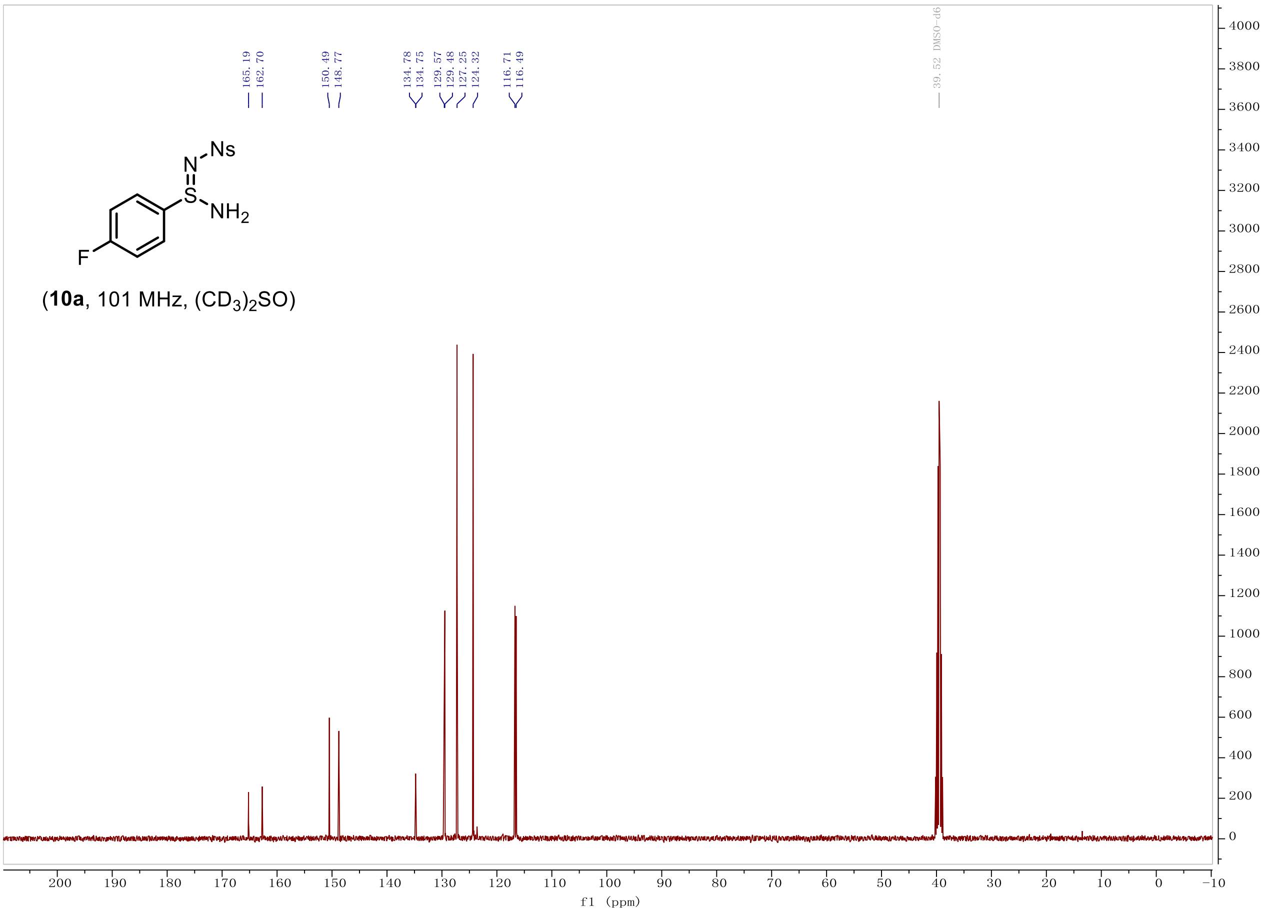

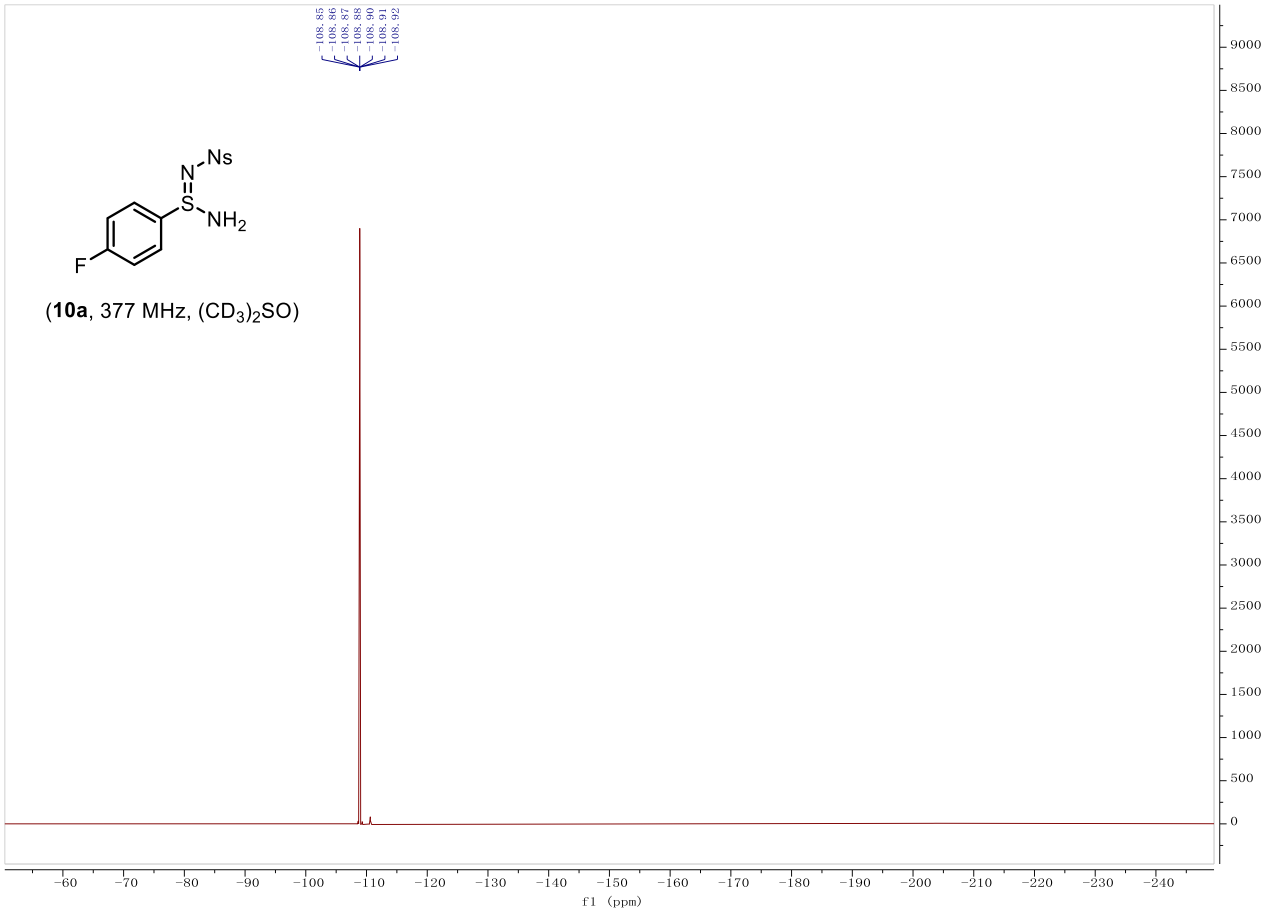

-108.85
-108.86
-108.87
-108.88
-108.90
-108.91
-108.92
Ns
N
S
NH2
F
(10a, 377 MHz, (CD3)2SO)
f1 (ppm)

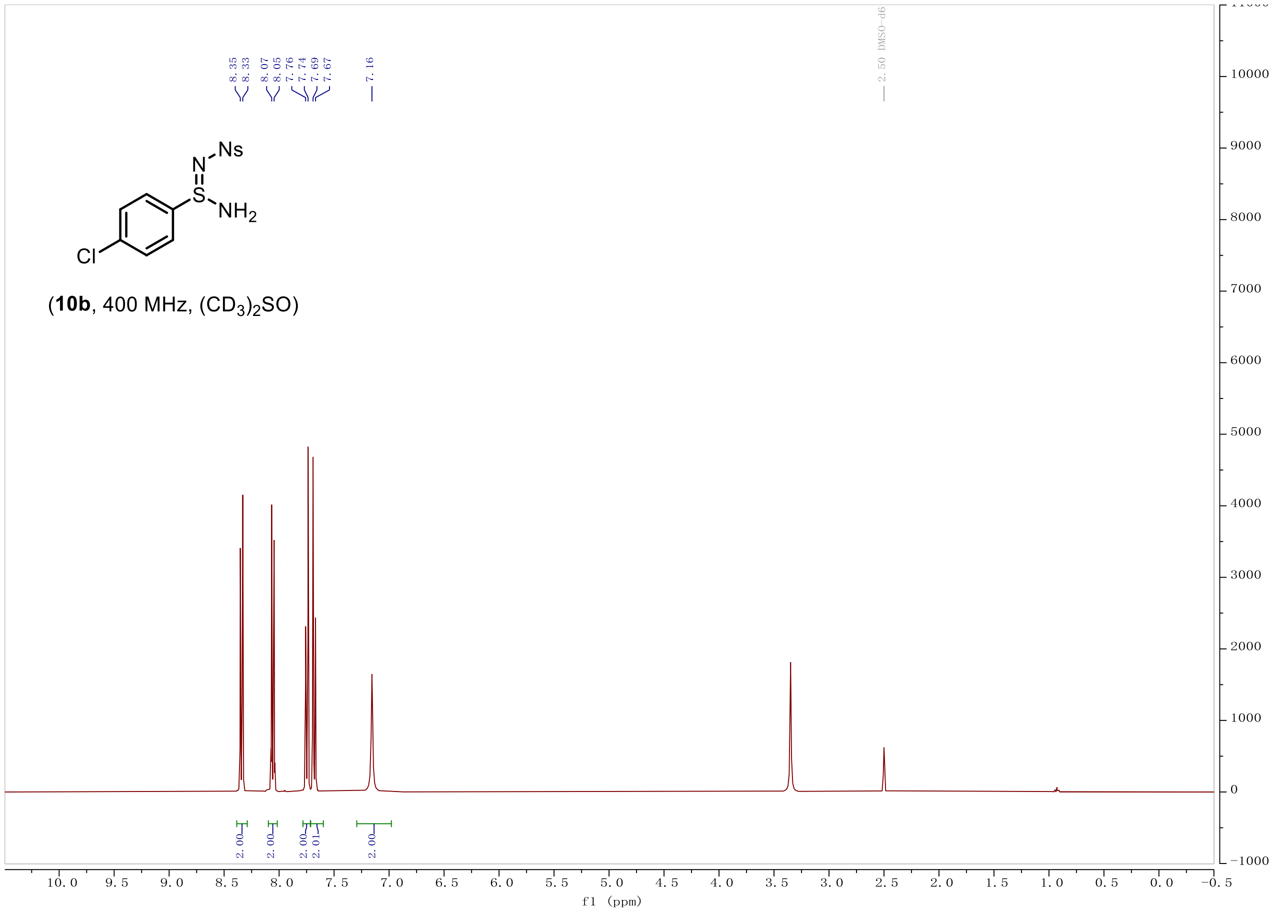

8.35
8.33
8.07
8.05
7.76
7.74
7.69
7.67
7.16
2.50 DMSO-d6
Ns
N
S
NH2
Cl
(10b, 400 MHz, (CD3)2SO)
2.00
2.00
2.00
2.01
2.00
f1 (ppm)

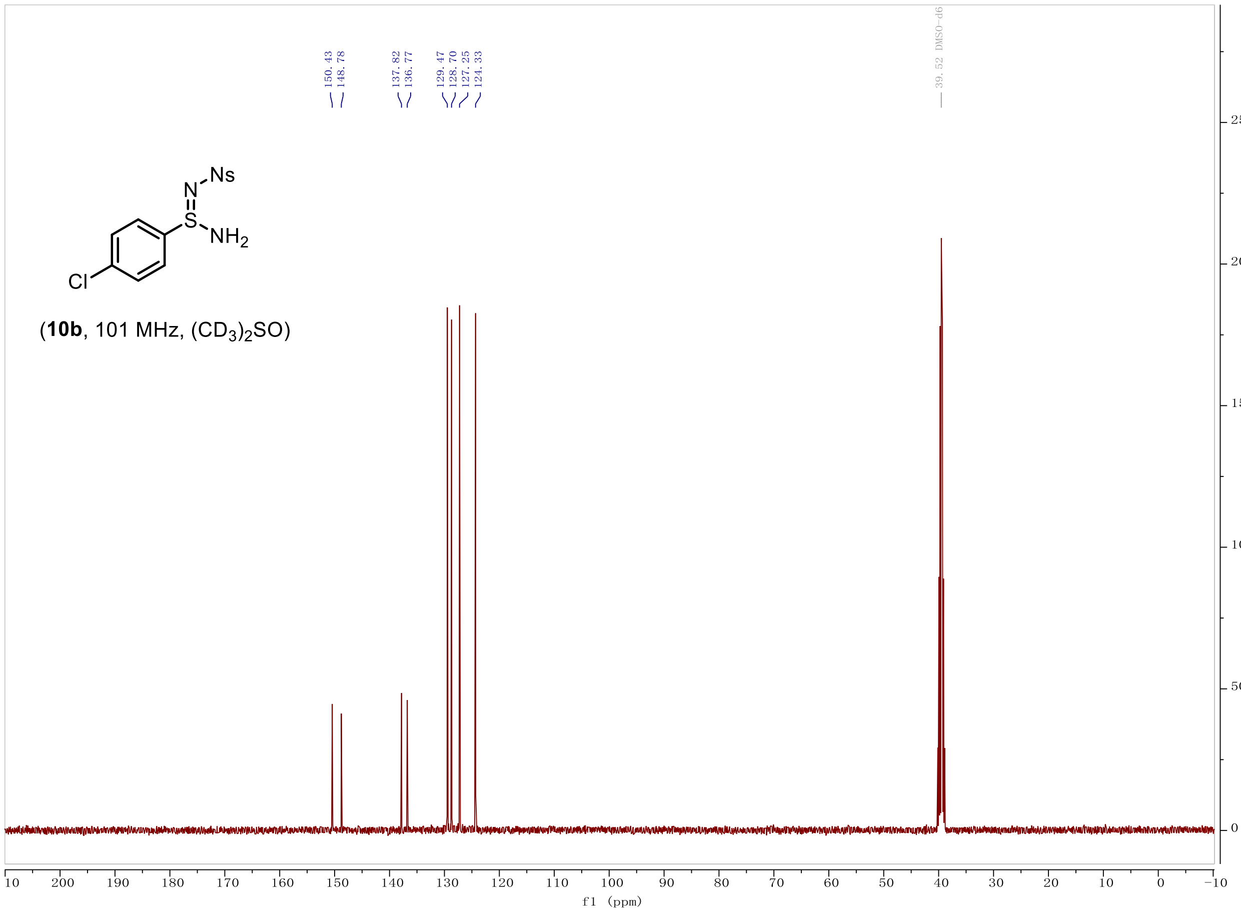
150.43
148.78
137.82
136.77
129.47
128.70
127.25
124.33
39.52 DMSO-d6
Ns
N
S
NH2
Cl
(10b, 101 MHz, (CD3)2SO)
f1 (ppm)

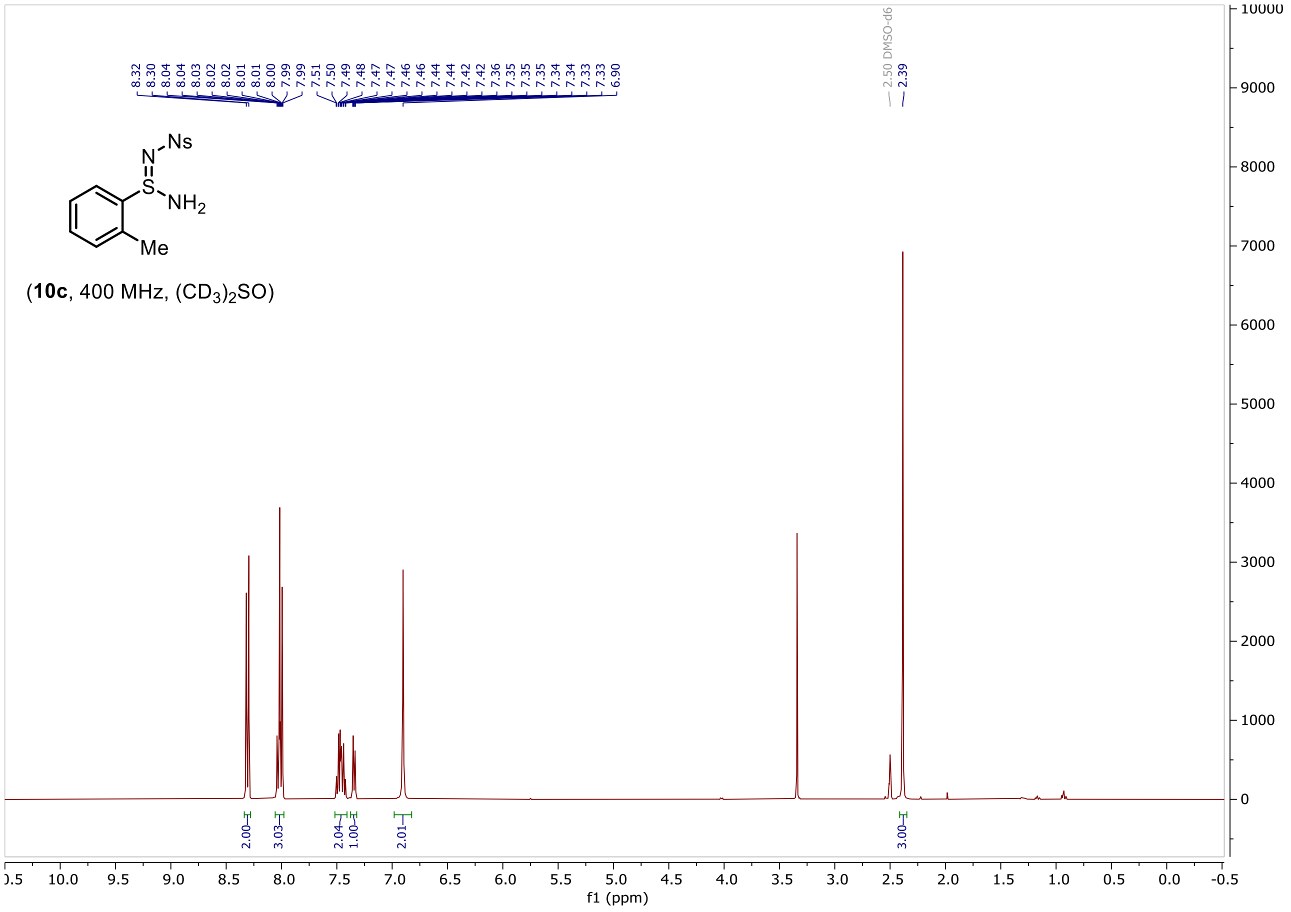
8.32
8.30
8.04
8.04
8.03
8.02
8.02
8.01
8.00
7.99
7.99
7.51
7.50
7.49
7.48
7.47
7.46
7.46
7.44
7.44
7.42
7.42
7.36
7.35
7.35
7.35
7.34
7.33
7.33
6.90
2.50 DMSO-d6
2.39
Ns
N
S
NH2
Me
(10c, 400 MHz, (CD3)2SO)
2.00
3.03
2.04
1.00
2.01
3.00
f1 (ppm)

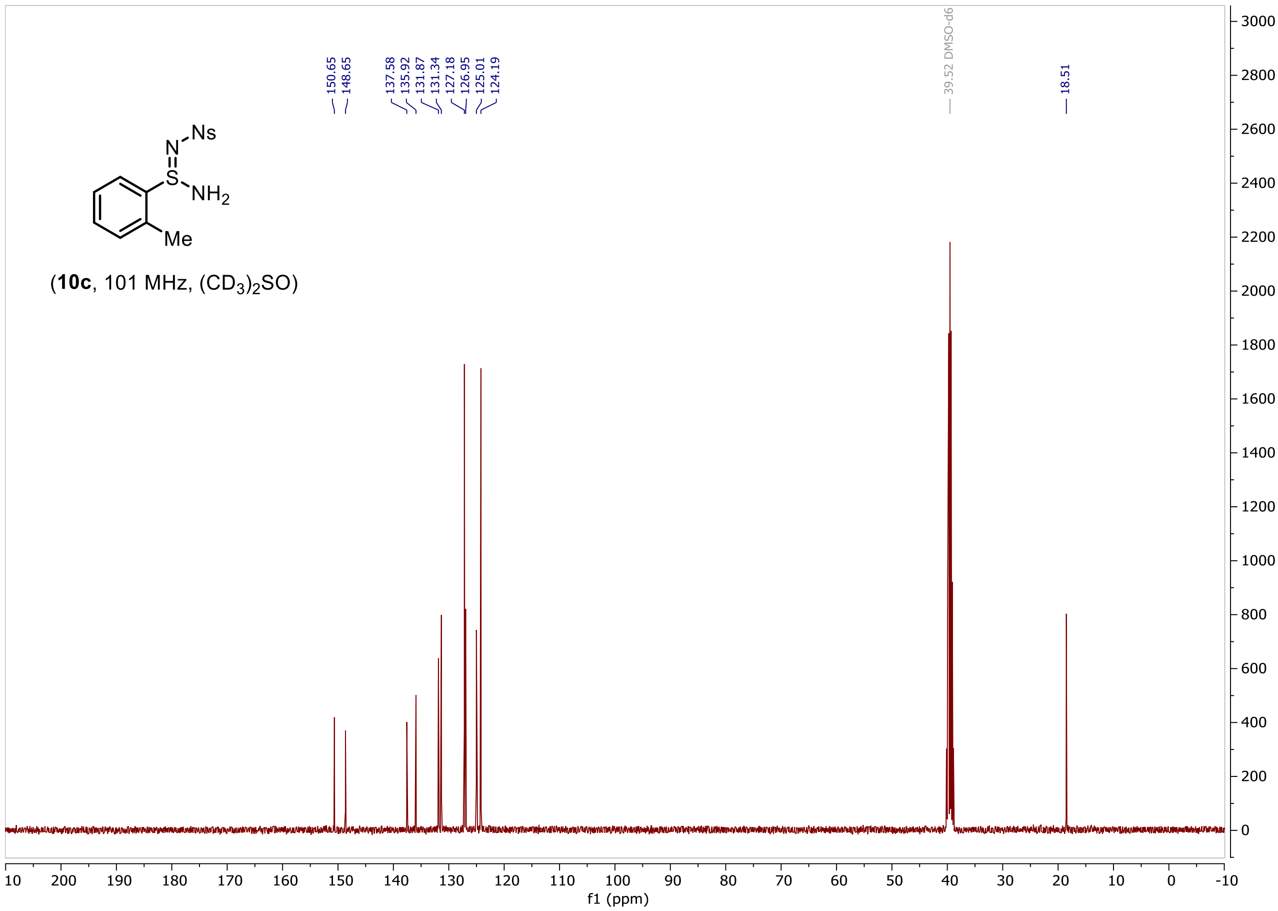
150.65
148.65
137.58
135.92
131.87
131.34
127.18
126.95
125.01
124.19
39.52 DMSO-d6
18.51
Ns
N
S
NH2
Me
(10c, 101 MHz, (CD3)2SO)
f1 (ppm)

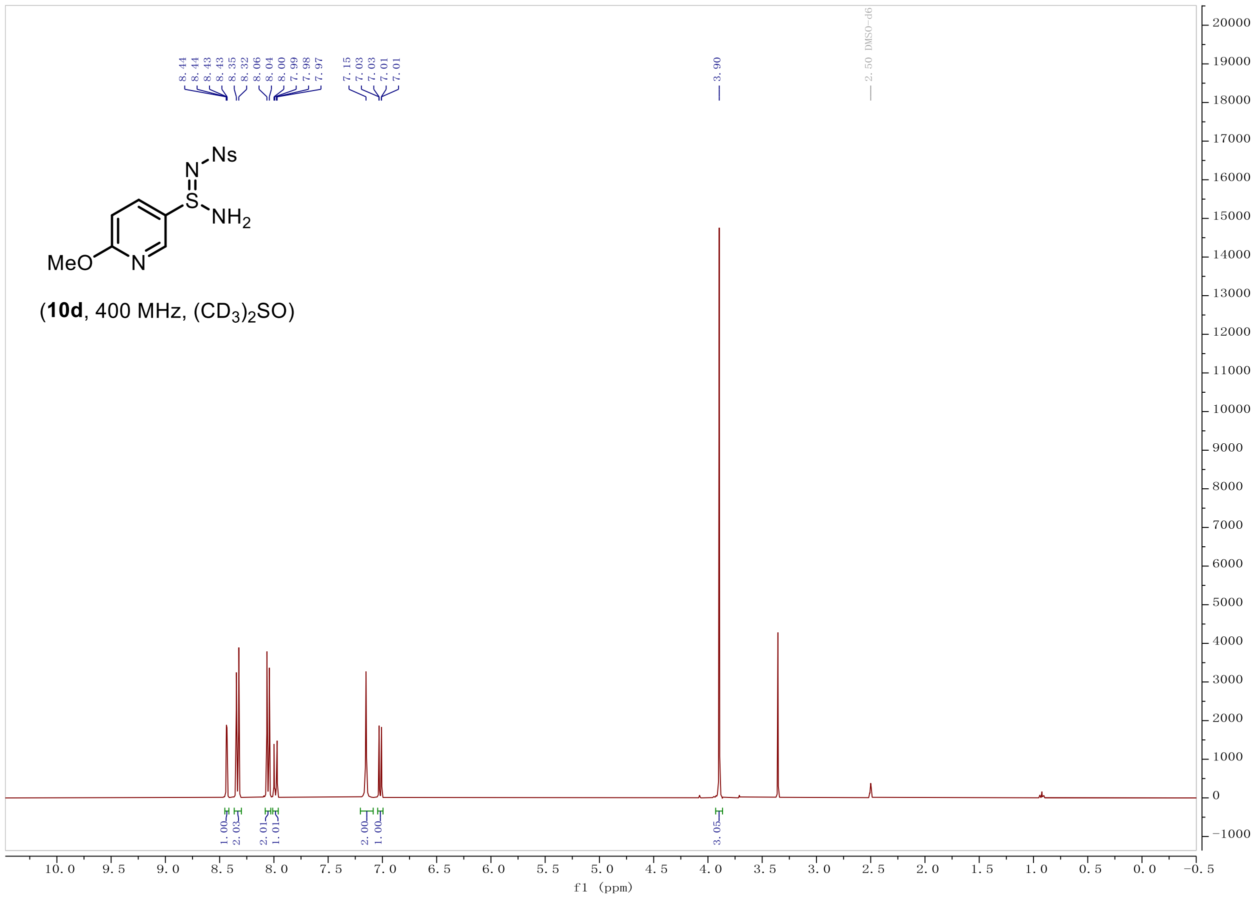
8.44
8.44
8.43
8.43
8.35
8.32
8.06
8.04
8.00
7.99
7.98
7.97
7.15
7.03
7.03
7.01
7.01
3.90
2.50 DMSO-d6
Ns
N
S
NH2
MeO
N
(10d, 400 MHz, (CD3)2SO)
1.00
2.03
2.01
1.01
2.00
1.00
3.05
f1 (ppm)

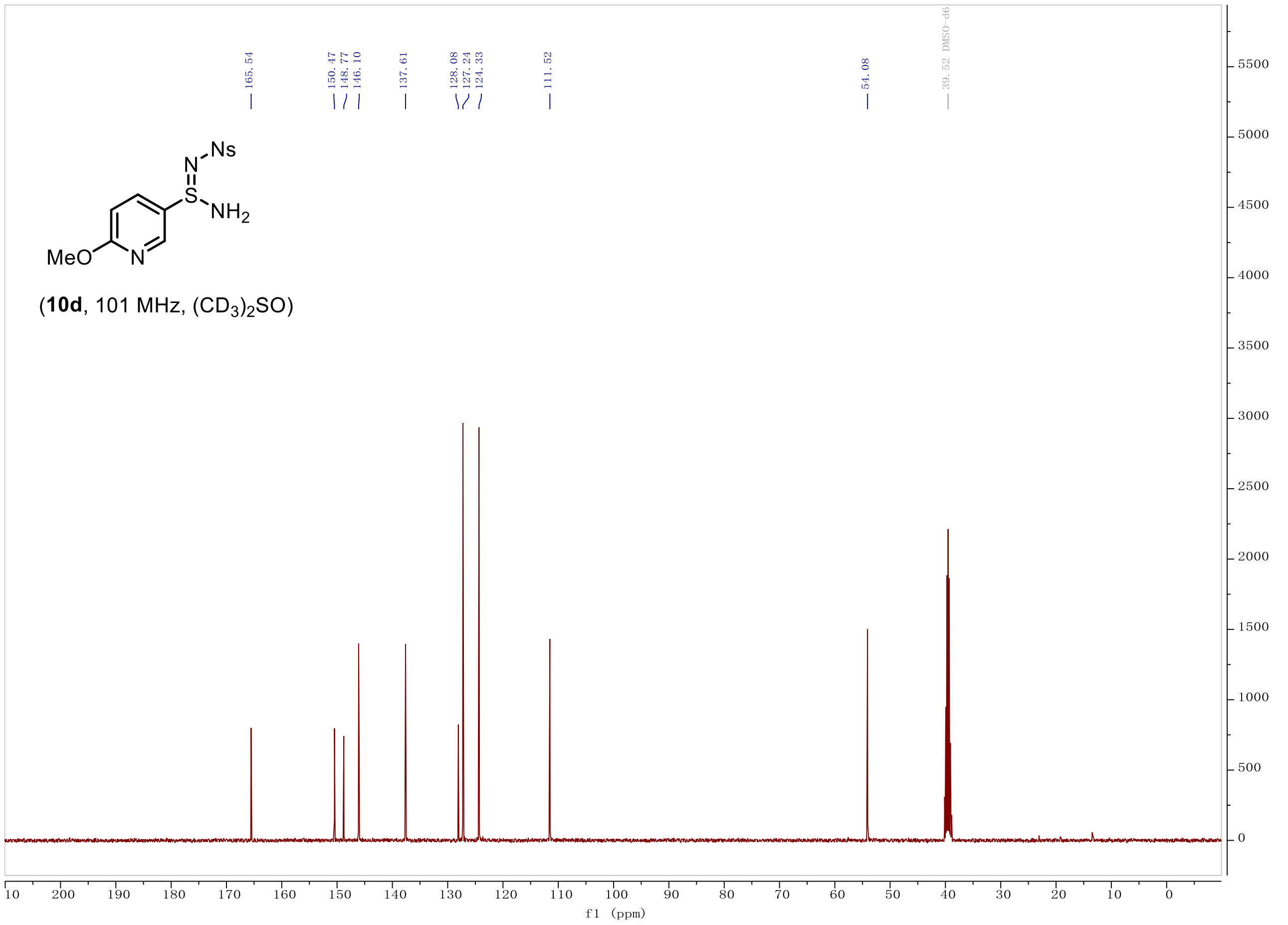

(**10d**, 101 MHz, $(CD_3)_2SO$)

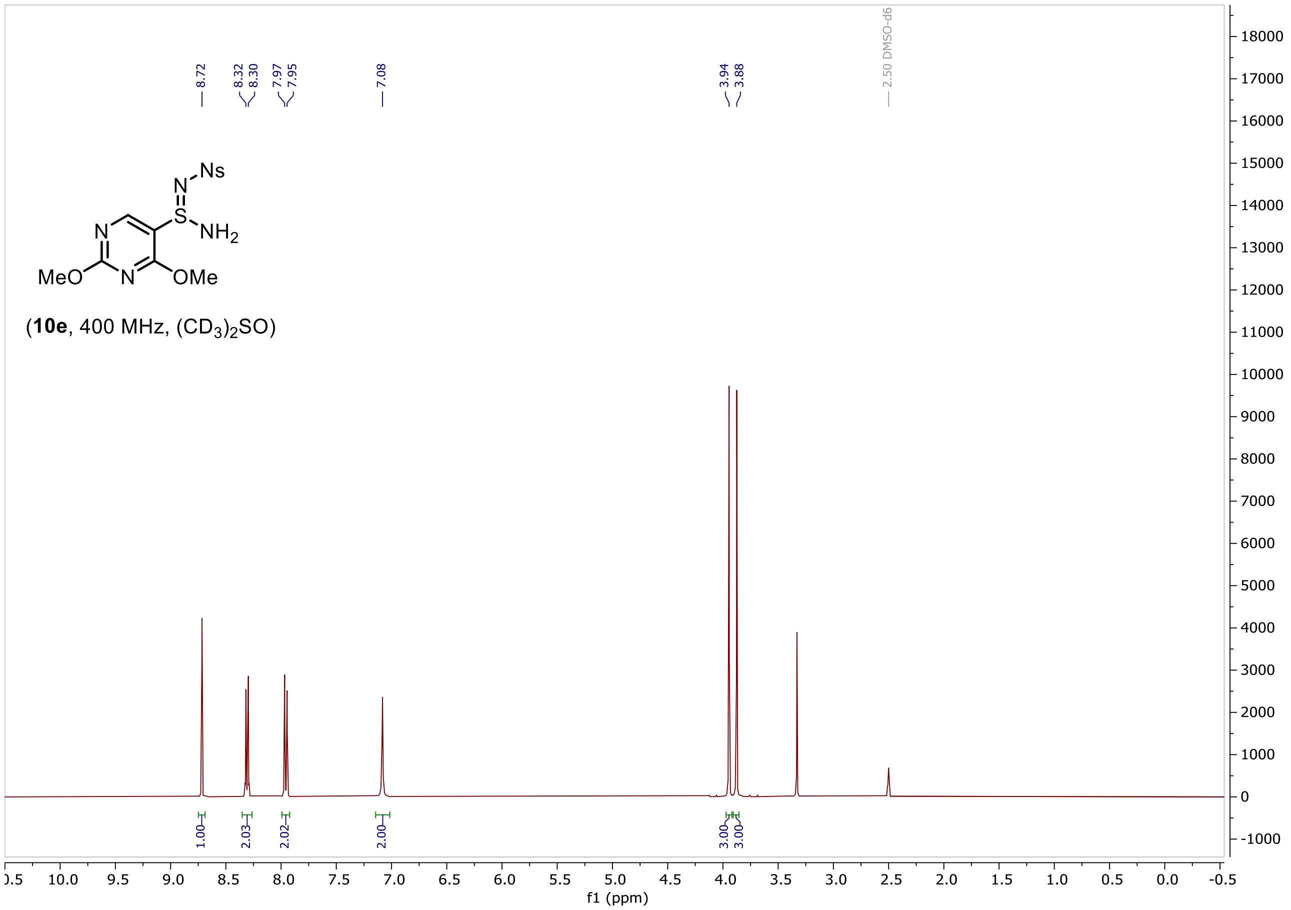

(**10e**, 400 MHz, $(CD_3)_2SO$)

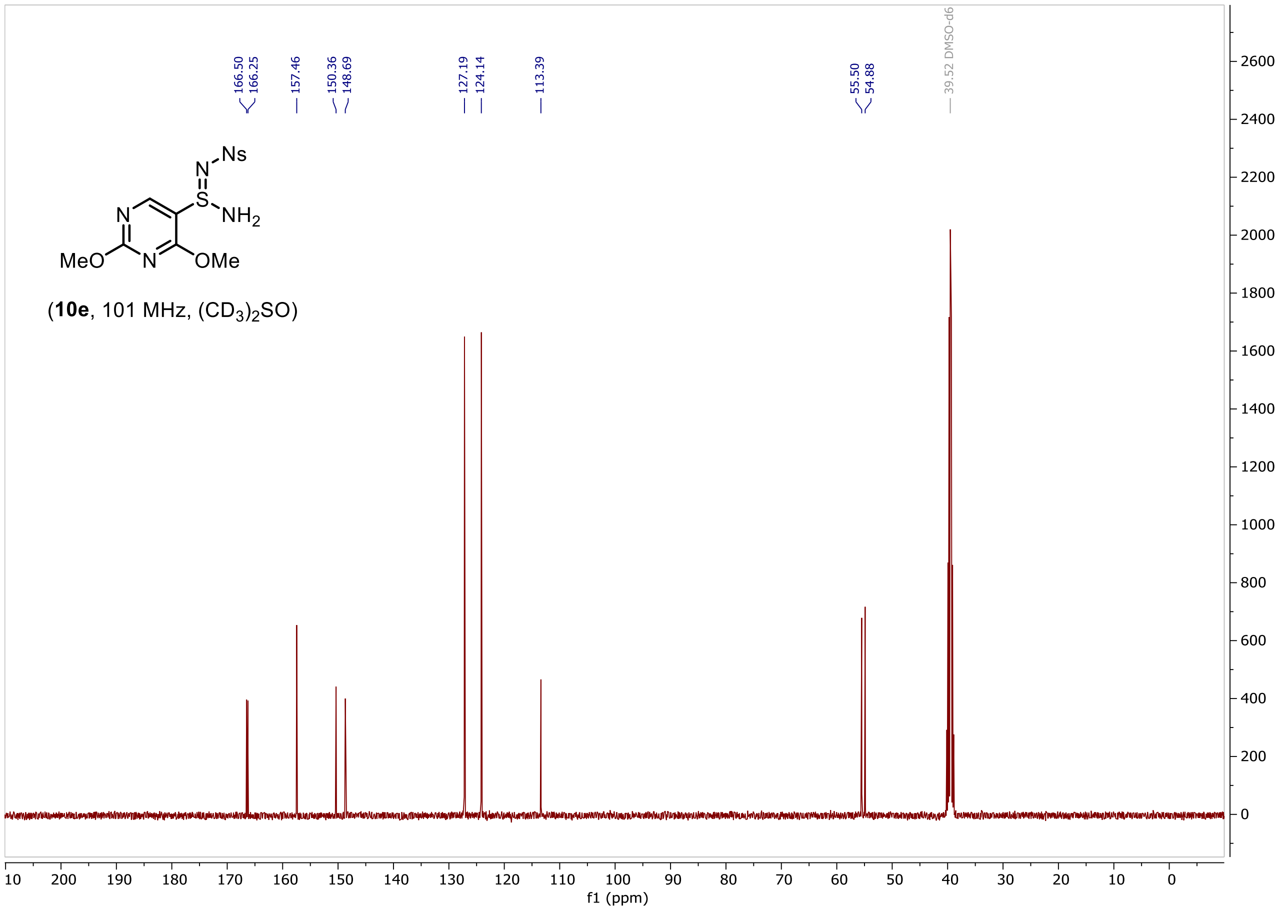
166.50
166.25
157.46
150.36
148.69
127.19
124.14
113.39
55.50
54.88
39.52 DMSO-d6
(10e, 101 MHz, (CD3)2SO)
f1 (ppm)

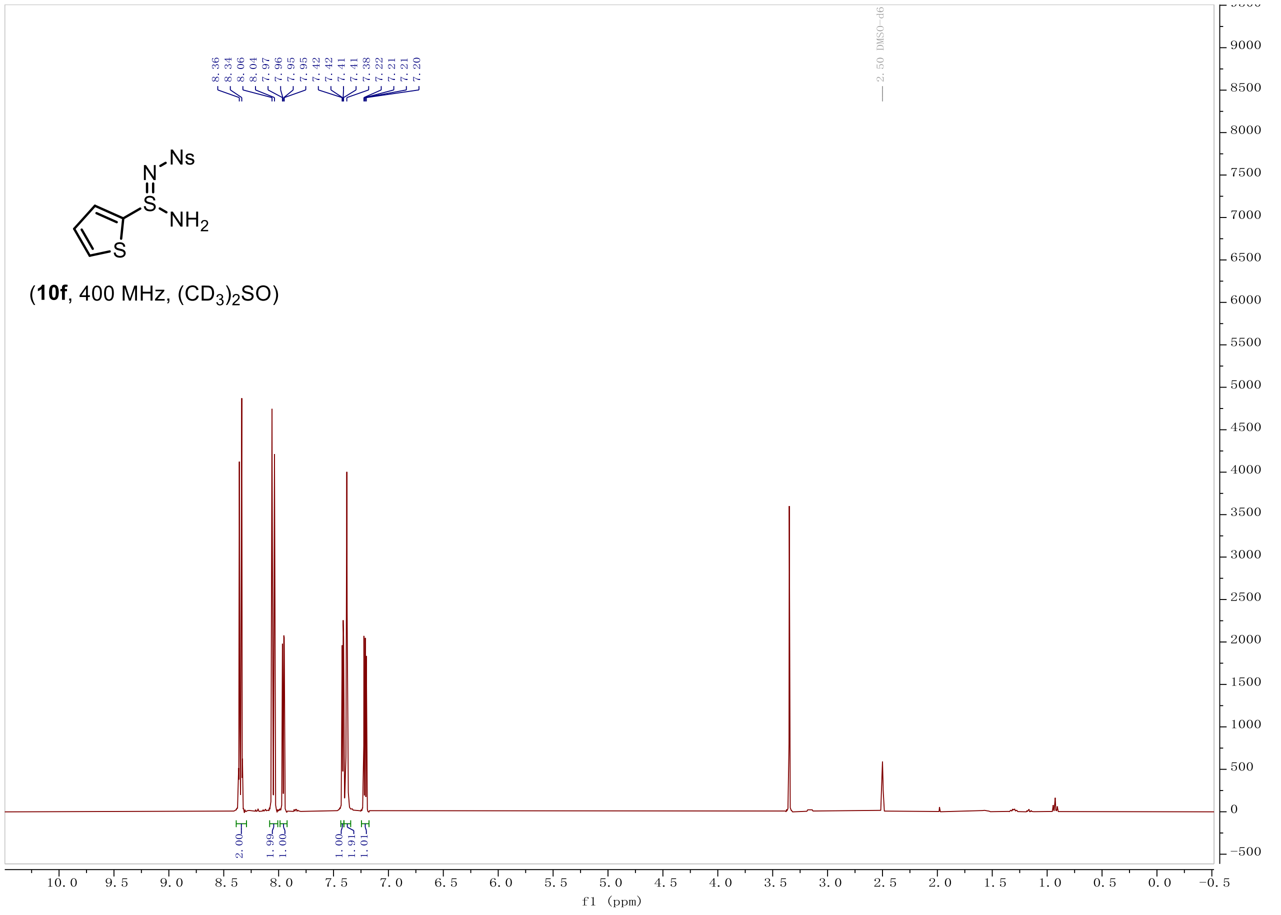
8.36
8.34
8.06
8.04
7.97
7.96
7.95
7.95
7.42
7.42
7.41
7.41
7.38
7.22
7.21
7.21
7.20
2.50 DMSO-d6
(10f, 400 MHz, (CD3)2SO)
2.00
1.99
1.00
1.00
1.91
1.01
f1 (ppm)

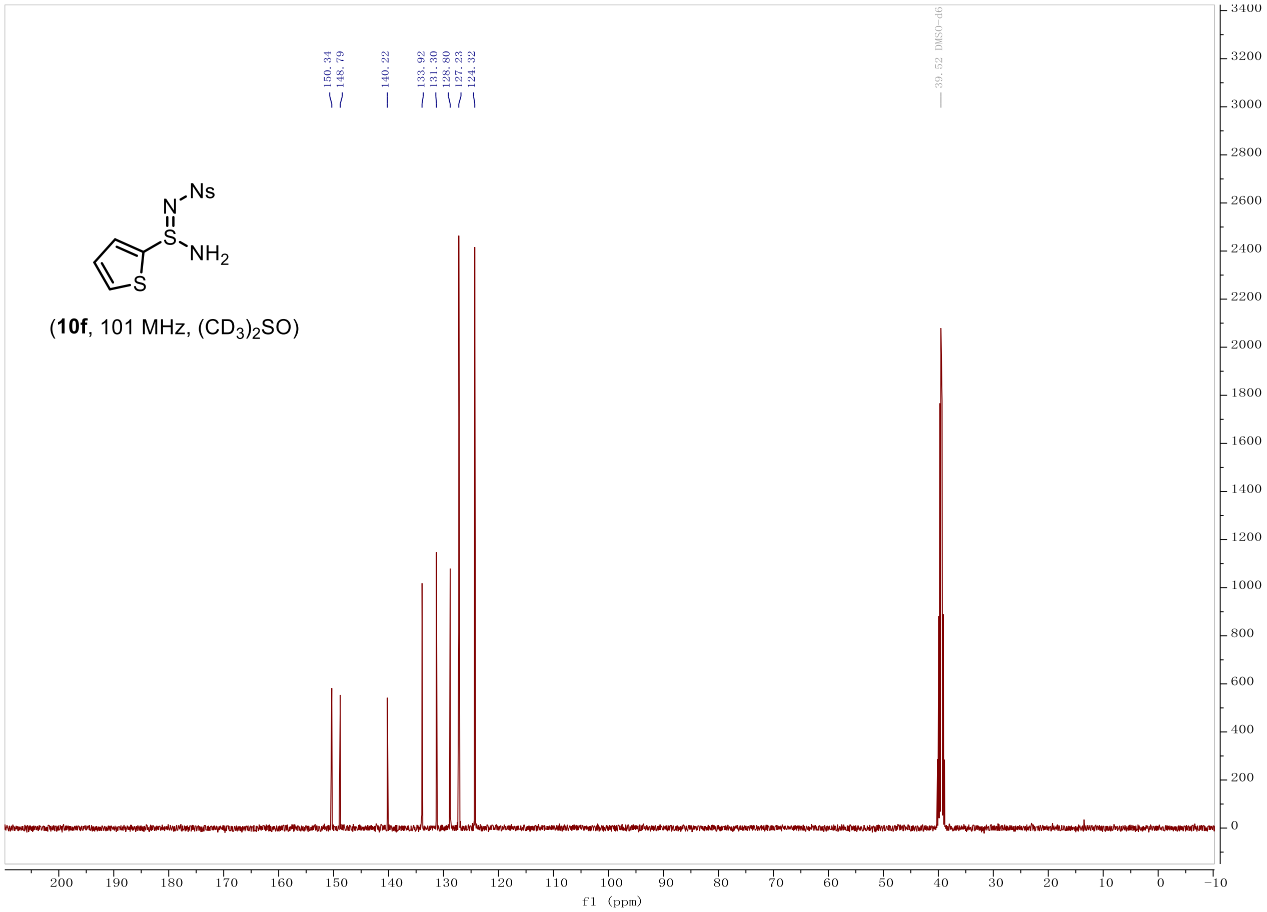
150.34
148.79
140.22
133.92
131.30
128.80
127.23
124.32
39.52 DMSO-d6
N
Ns
S
NH2
S
(10f, 101 MHz, (CD3)2SO)
f1 (ppm)

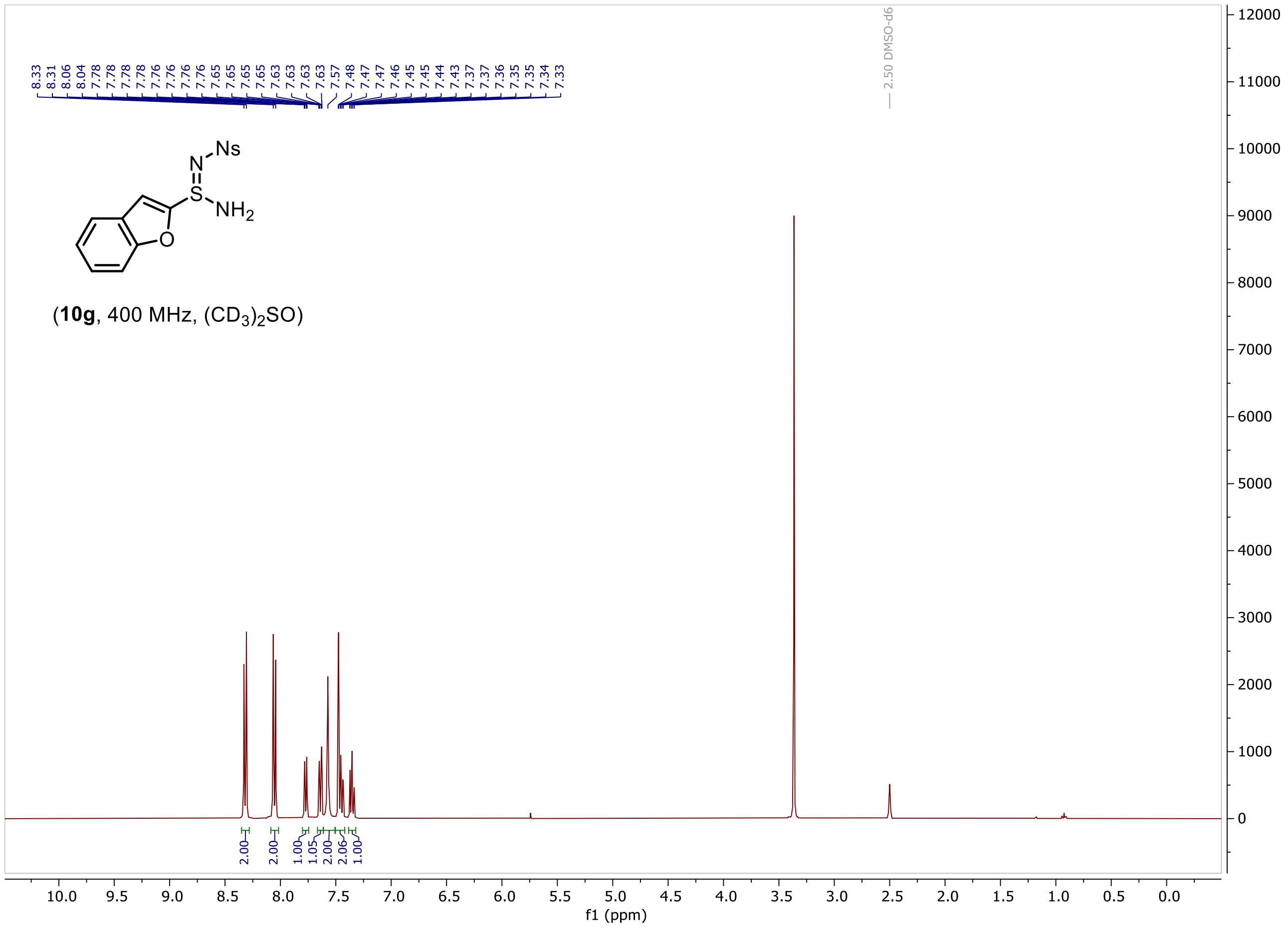
2.50 DMSO-d6
N
Ns
S
NH2
O
(10g, 400 MHz, (CD3)2SO)
2.00
2.00
1.00
1.05
2.00
2.06
1.00
f1 (ppm)

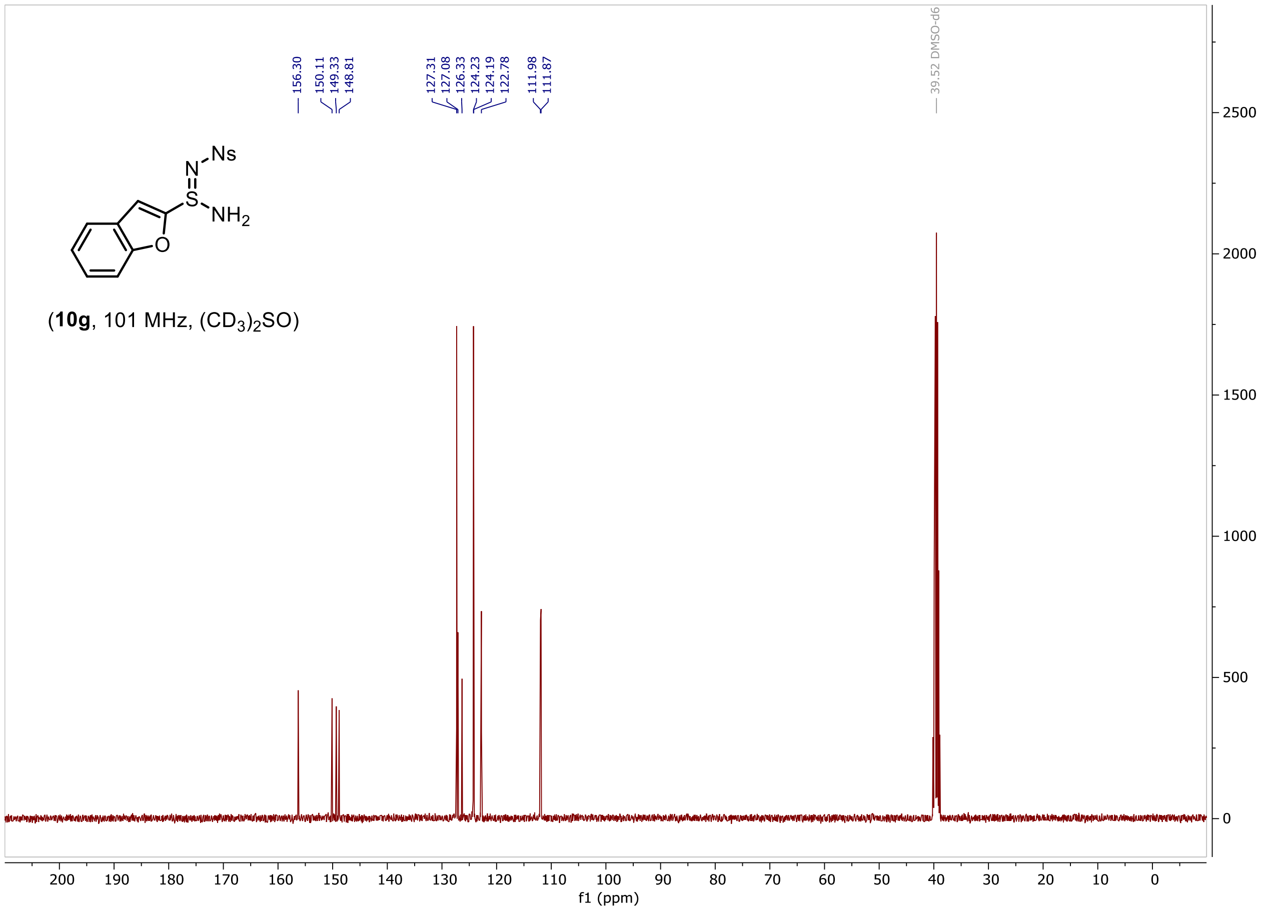

(**10g**, 101 MHz, $(CD_3)_2SO$)

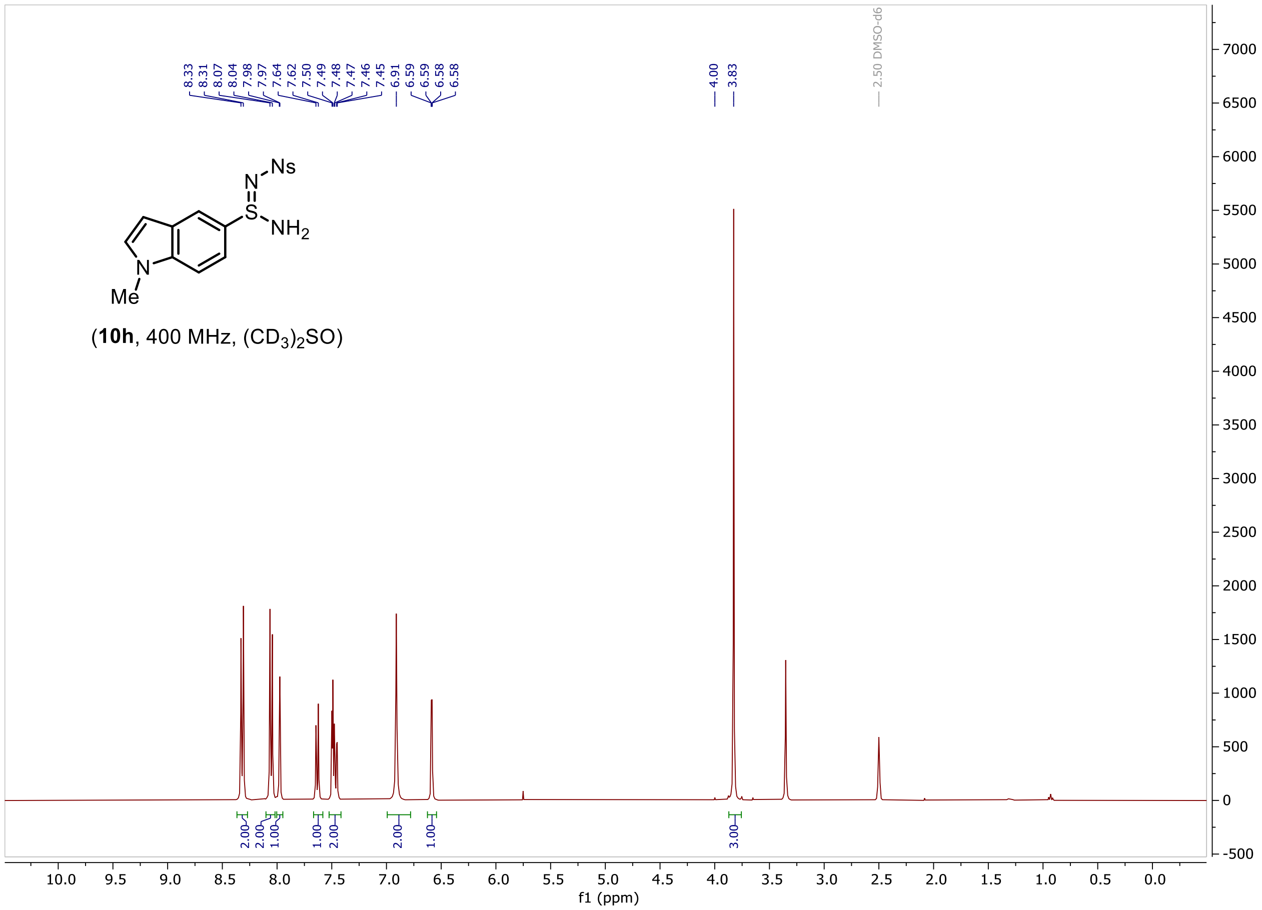

(**10h**, 400 MHz, $(CD_3)_2SO$)

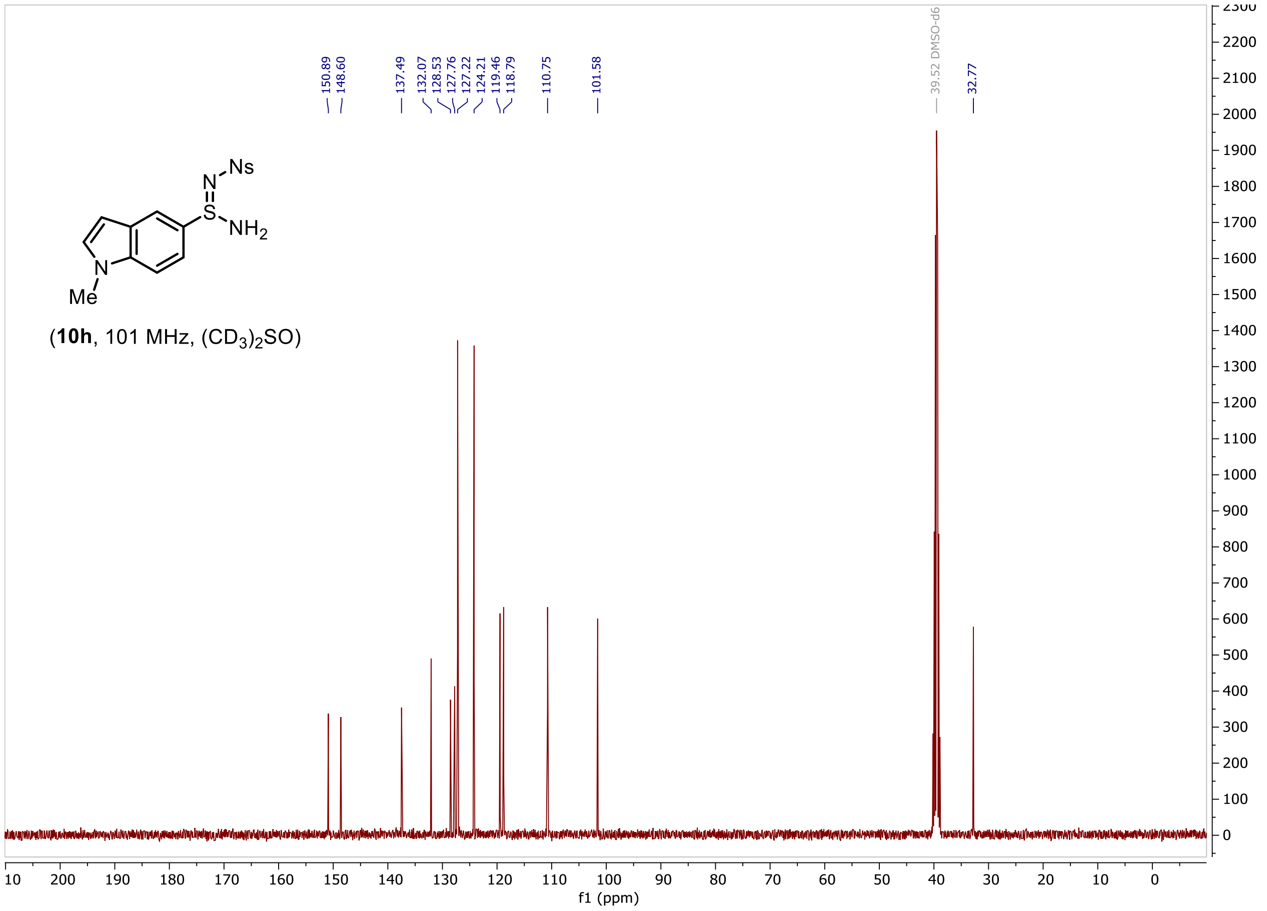
150.89
148.60
137.49
132.07
128.53
127.76
127.22
124.21
119.46
118.79
110.75
101.58
39.52 DMSO-d6
32.77
Ns
N
S
NH2
N
Me
(10h, 101 MHz, (CD3)2SO)
f1 (ppm)

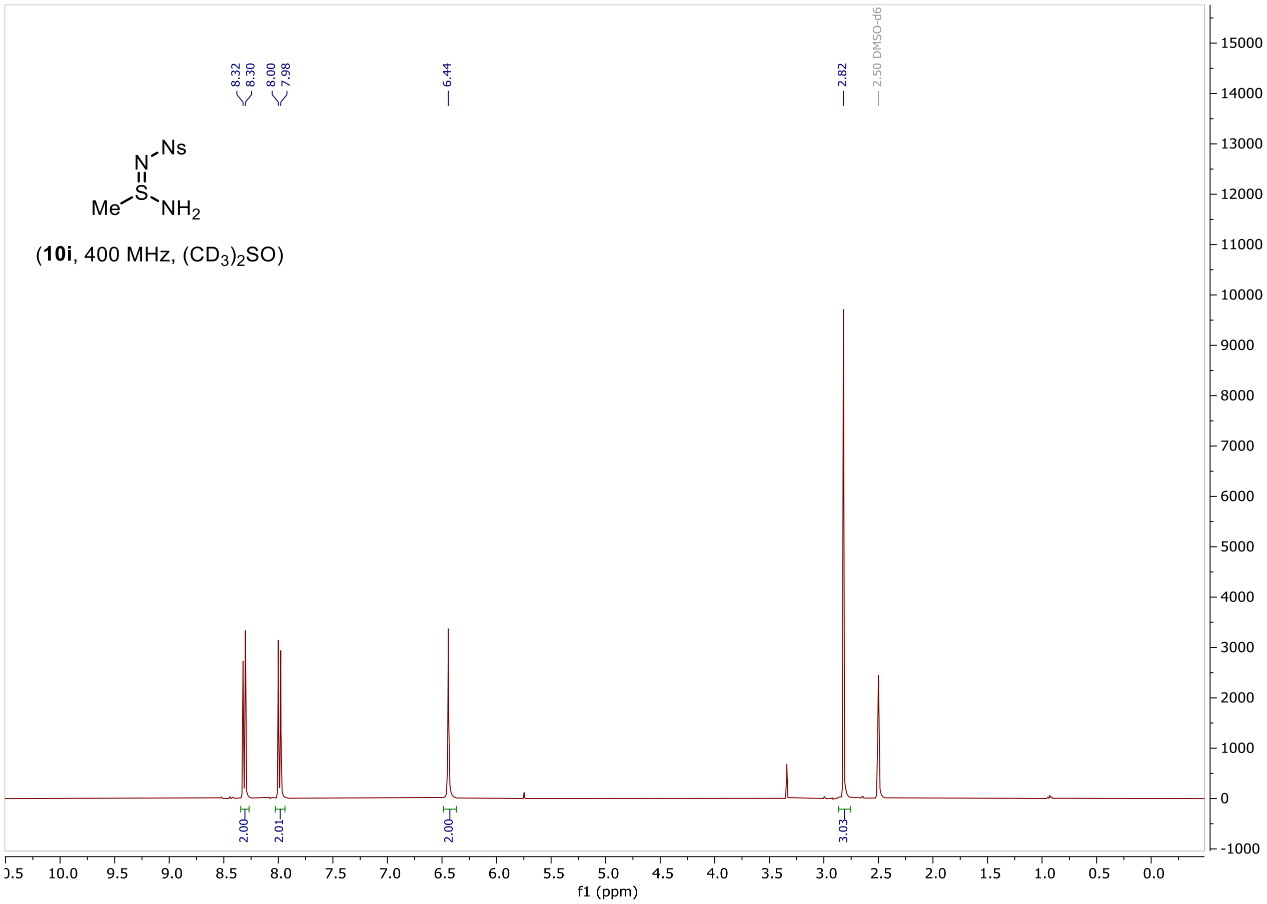
8.32
8.30
8.00
7.98
6.44
2.82
2.50 DMSO-d6
Ns
N
Me
S
NH2
(10i, 400 MHz, (CD3)2SO)
2.00
2.01
2.00
3.03
f1 (ppm)

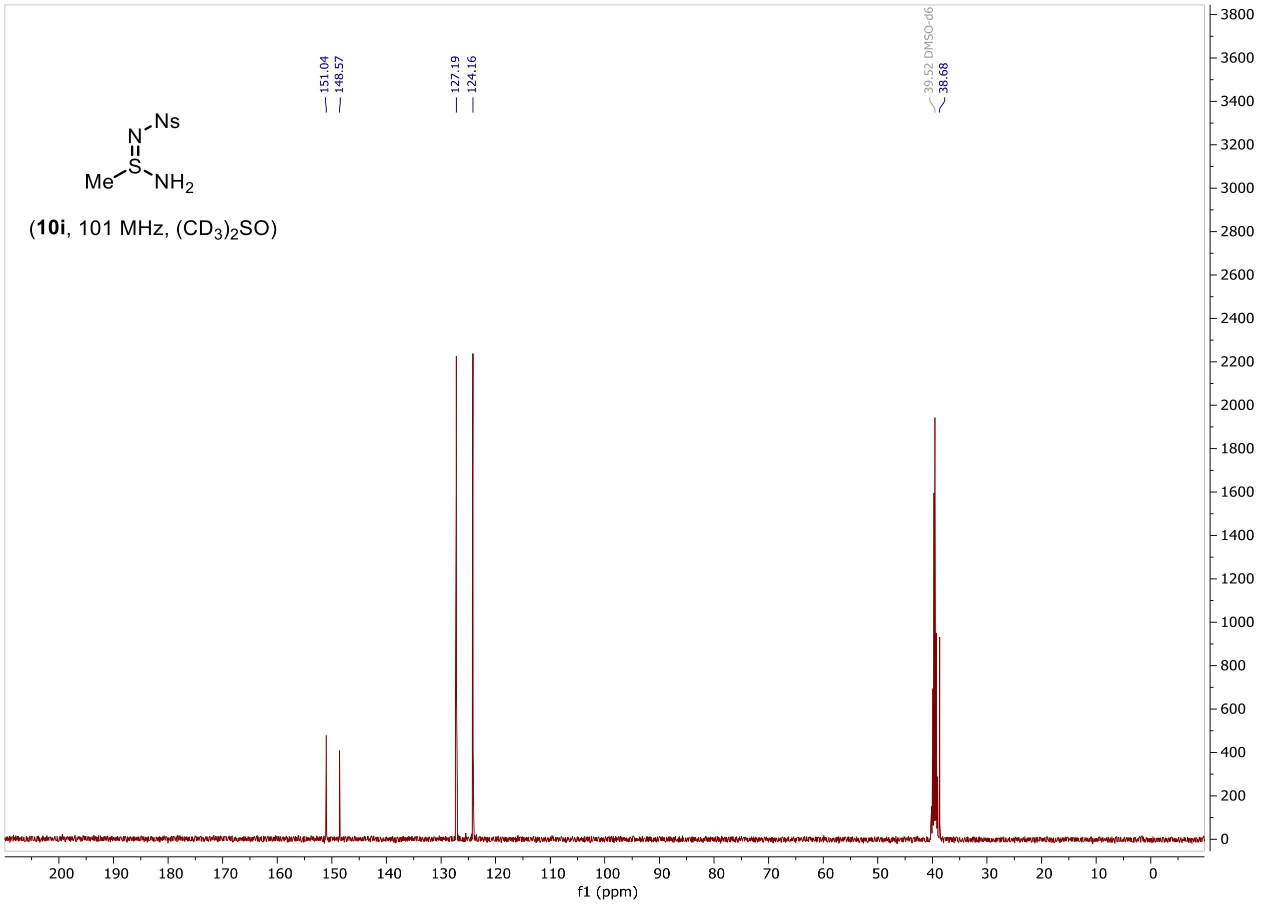

N-Ns
Me S NH2
(10i, 101 MHz, (CD3)2SO)
151.04
148.57
127.19
124.16
39.52 DMSO-d6
38.68
f1 (ppm)

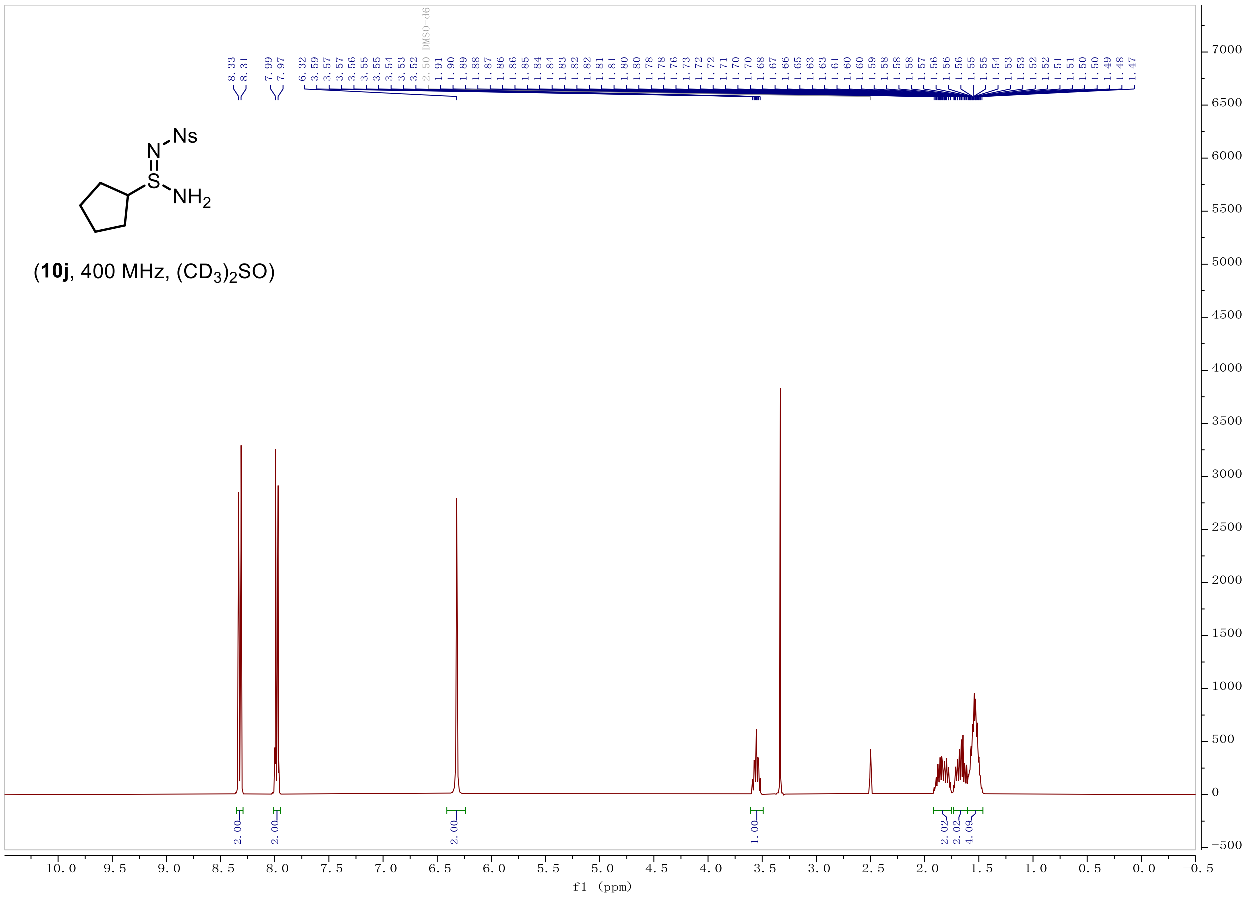

N-Ns
S NH2
(10j, 400 MHz, (CD3)2SO)
8.33
8.31
7.99
7.97
6.32
3.59
3.57
3.57
3.56
3.55
3.55
3.54
3.53
3.52
2.50 DMSO-d6
2.00
2.00
2.00
1.00
2.02
2.02
4.09
f1 (ppm)

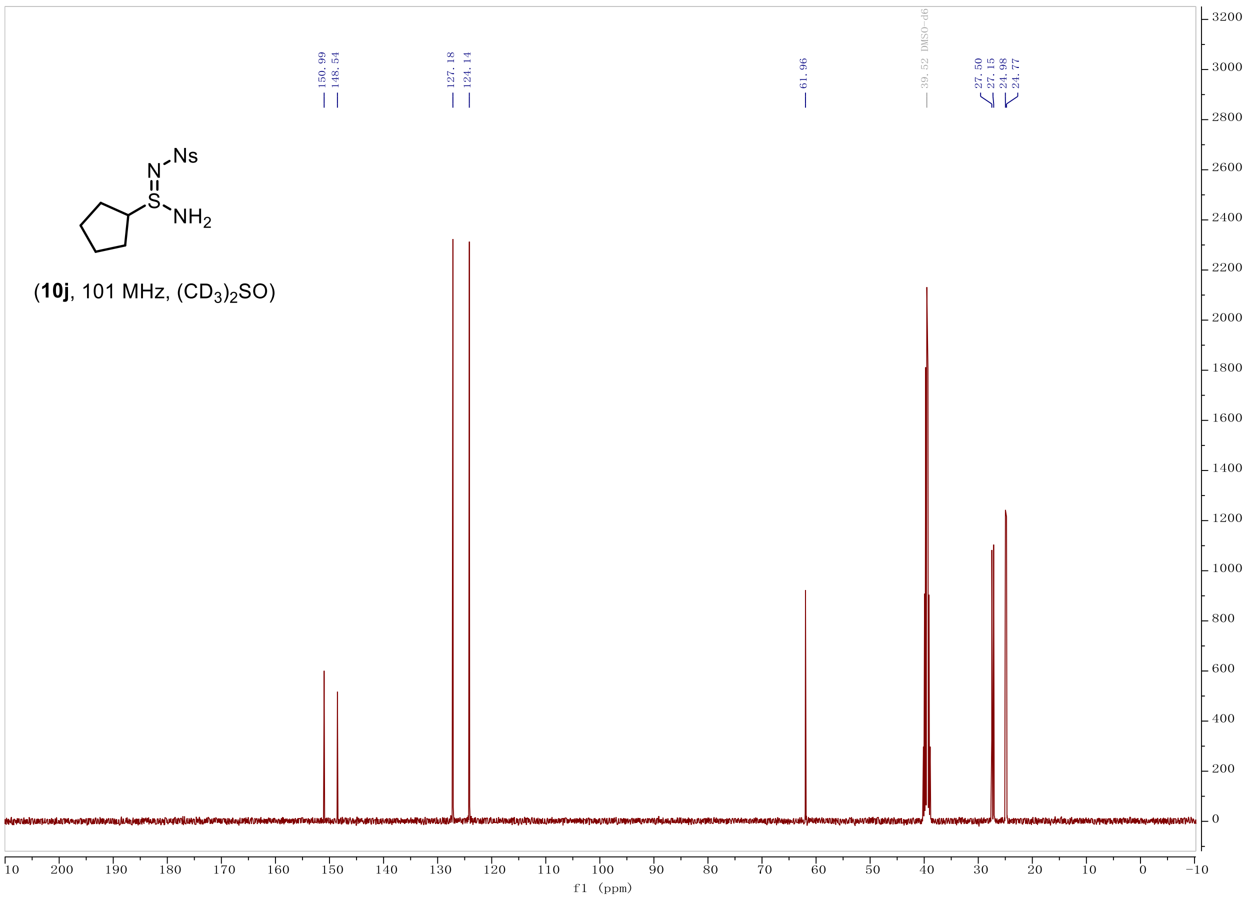
150.99
148.54
127.18
124.14
61.96
39.52 DMSO-d6
27.50
27.15
24.98
24.77
Ns
N
S
NH2
(10j, 101 MHz, (CD3)2SO)
f1 (ppm)

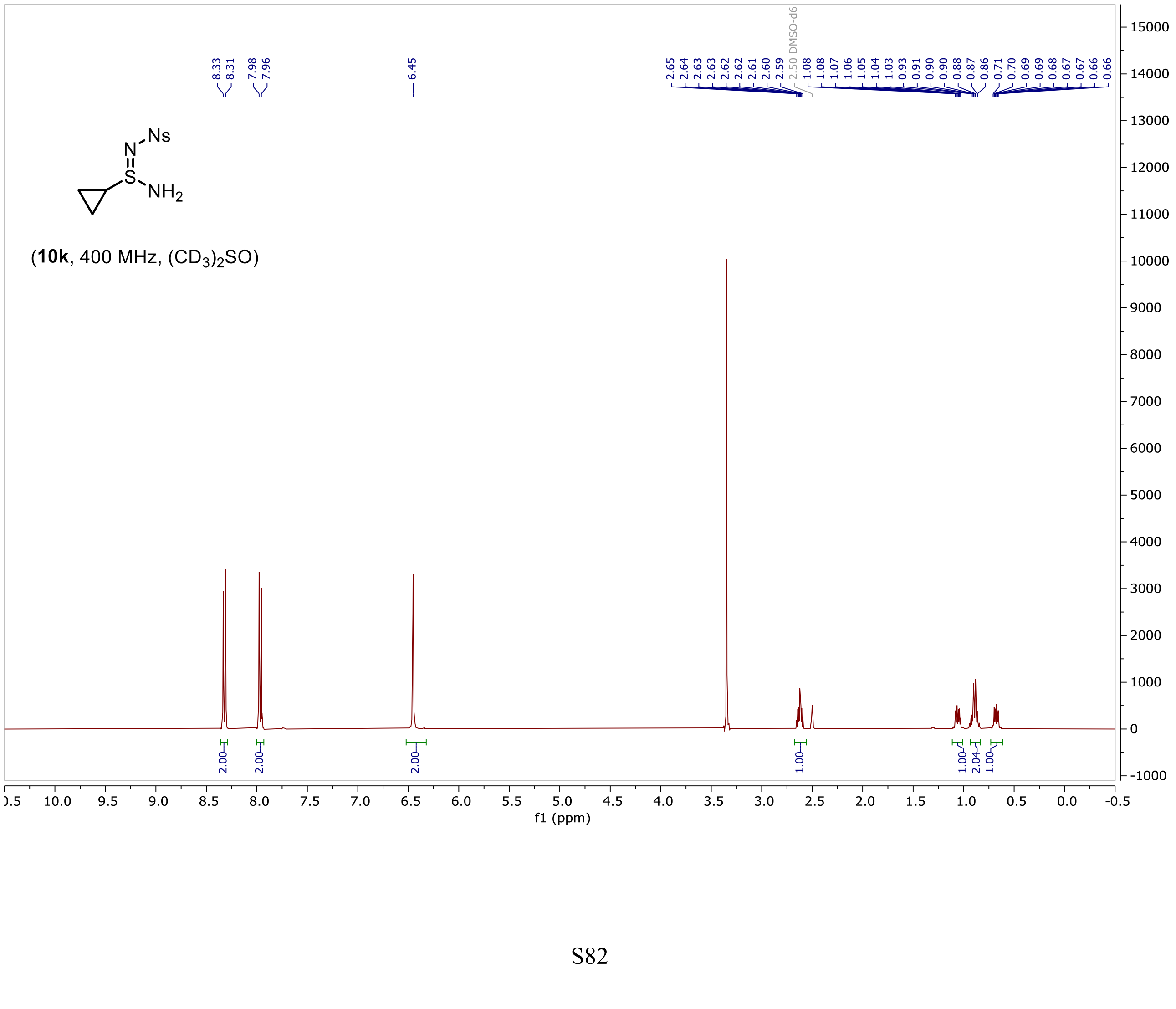
8.33
8.31
7.98
7.96
6.45
2.65
2.64
2.63
2.62
2.62
2.61
2.60
2.59
2.50 DMSO-d6
1.08
1.08
1.07
1.06
1.05
1.04
1.03
0.93
0.91
0.90
0.90
0.88
0.87
0.86
0.71
0.70
0.69
0.69
0.68
0.67
0.67
0.66
0.66
Ns
N
S
NH2
(10k, 400 MHz, (CD3)2SO)
2.00
2.00
2.00
1.00
1.00
2.04
1.00
f1 (ppm)

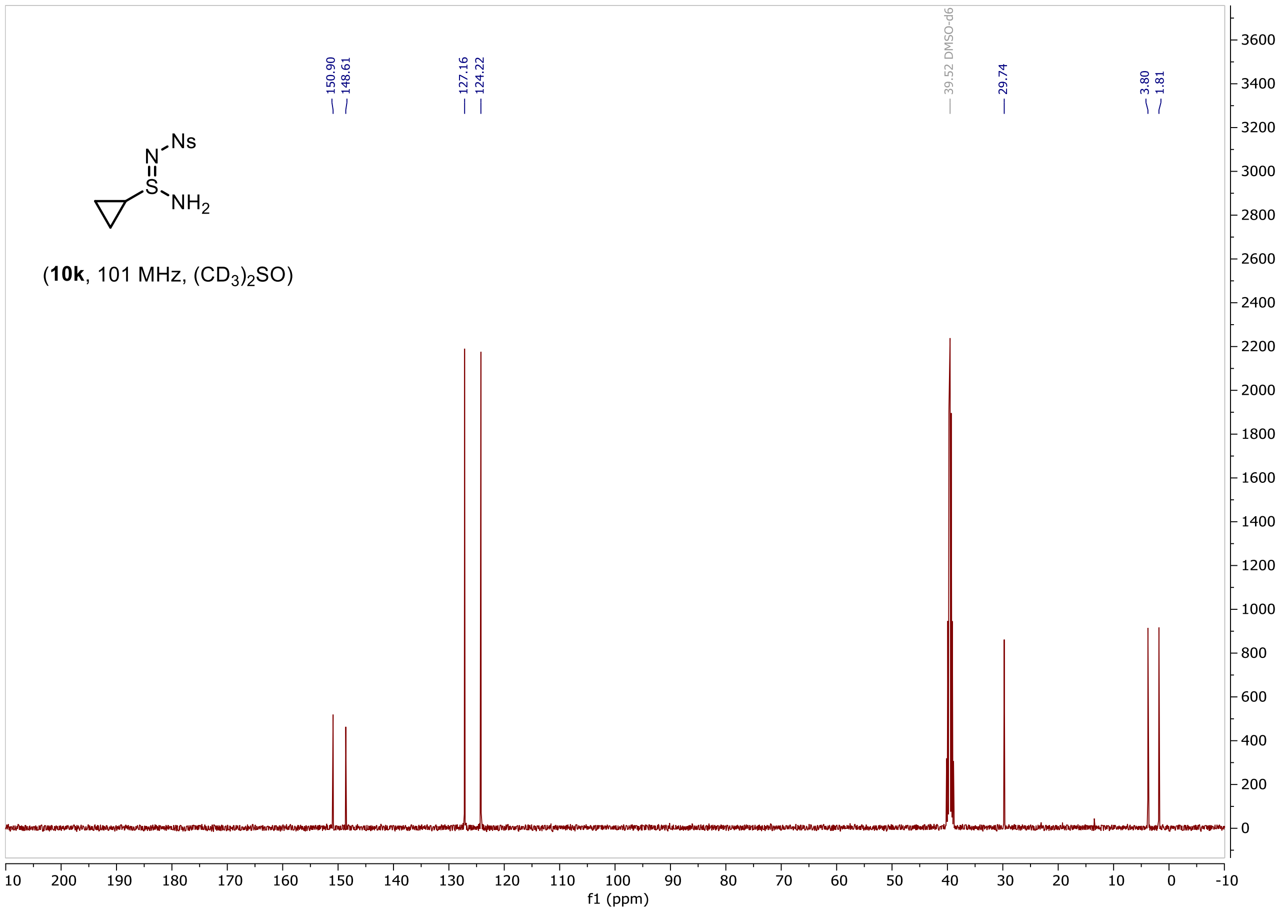
150.90
148.61
127.16
124.22
39.52 DMSO-d6
29.74
3.80
1.81
(10k, 101 MHz, (CD3)2SO)
f1 (ppm)

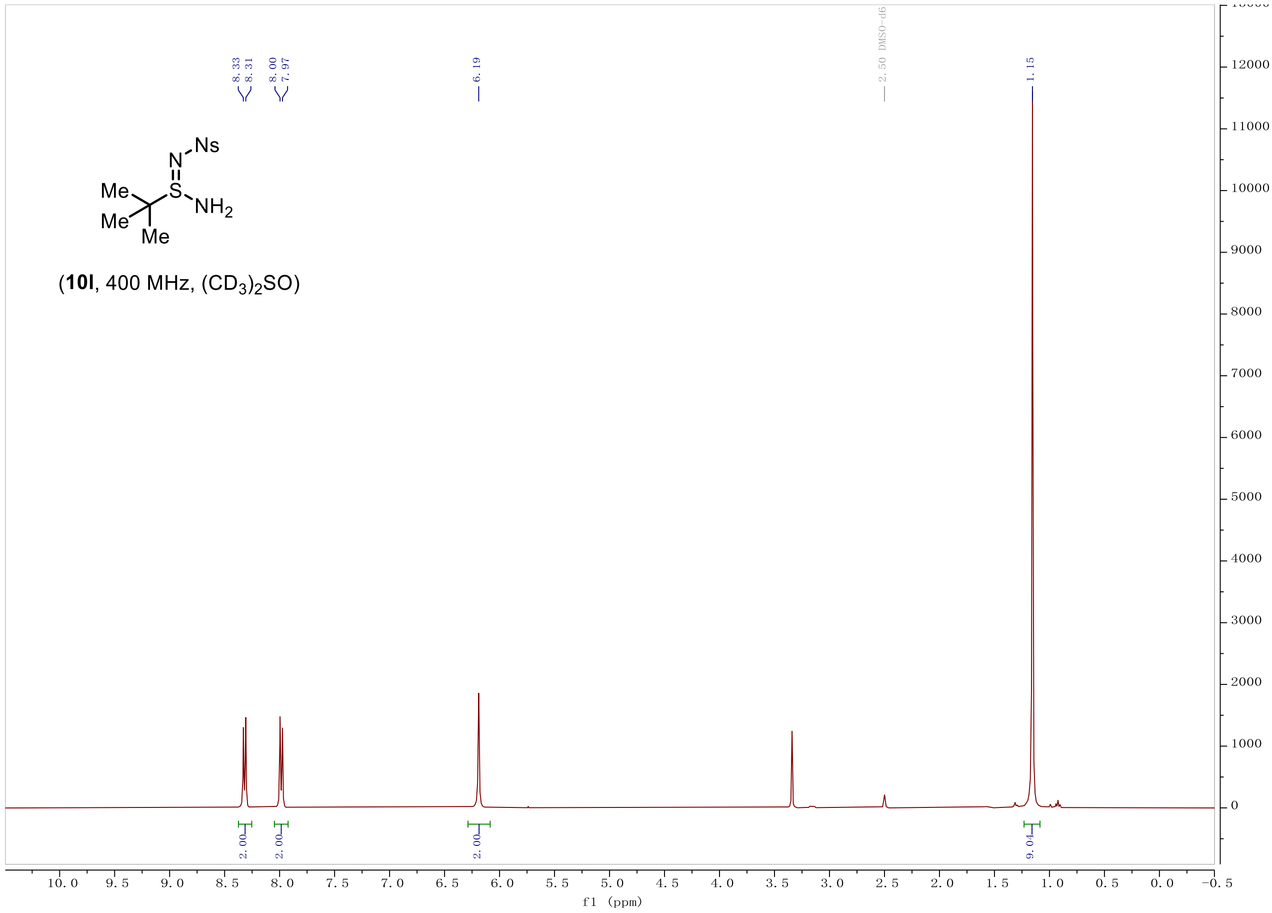
8.33
8.31
8.00
7.97
6.19
2.50 DMSO-d6
1.15
(10l, 400 MHz, (CD3)2SO)
2.00
2.00
2.00
9.04
f1 (ppm)

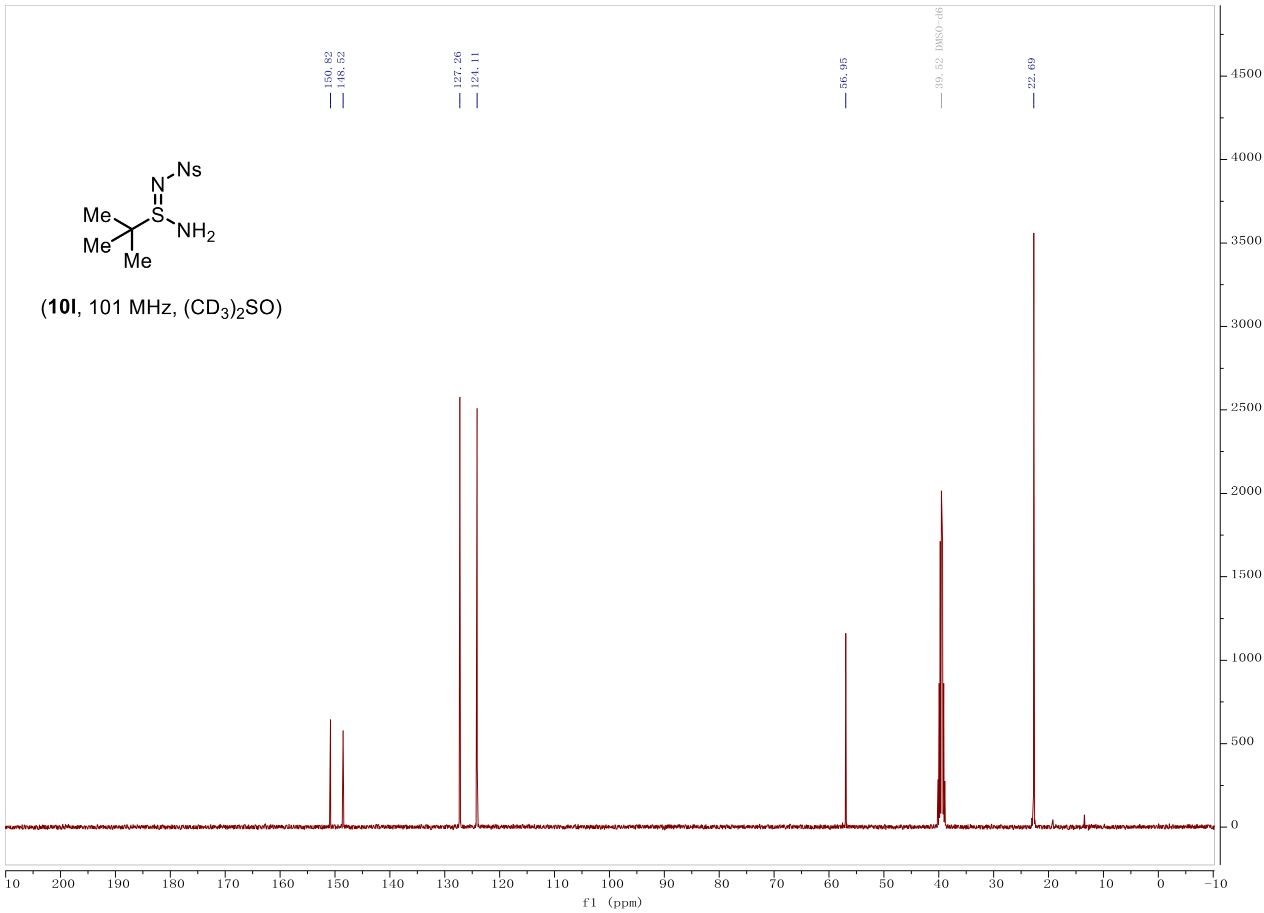

150.82
148.52
127.26
124.11
56.95
39.52 DMSO-d6
22.69
Ns
N
S
NH2
Me
Me
Me
(10l, 101 MHz, (CD3)2SO)
f1 (ppm)

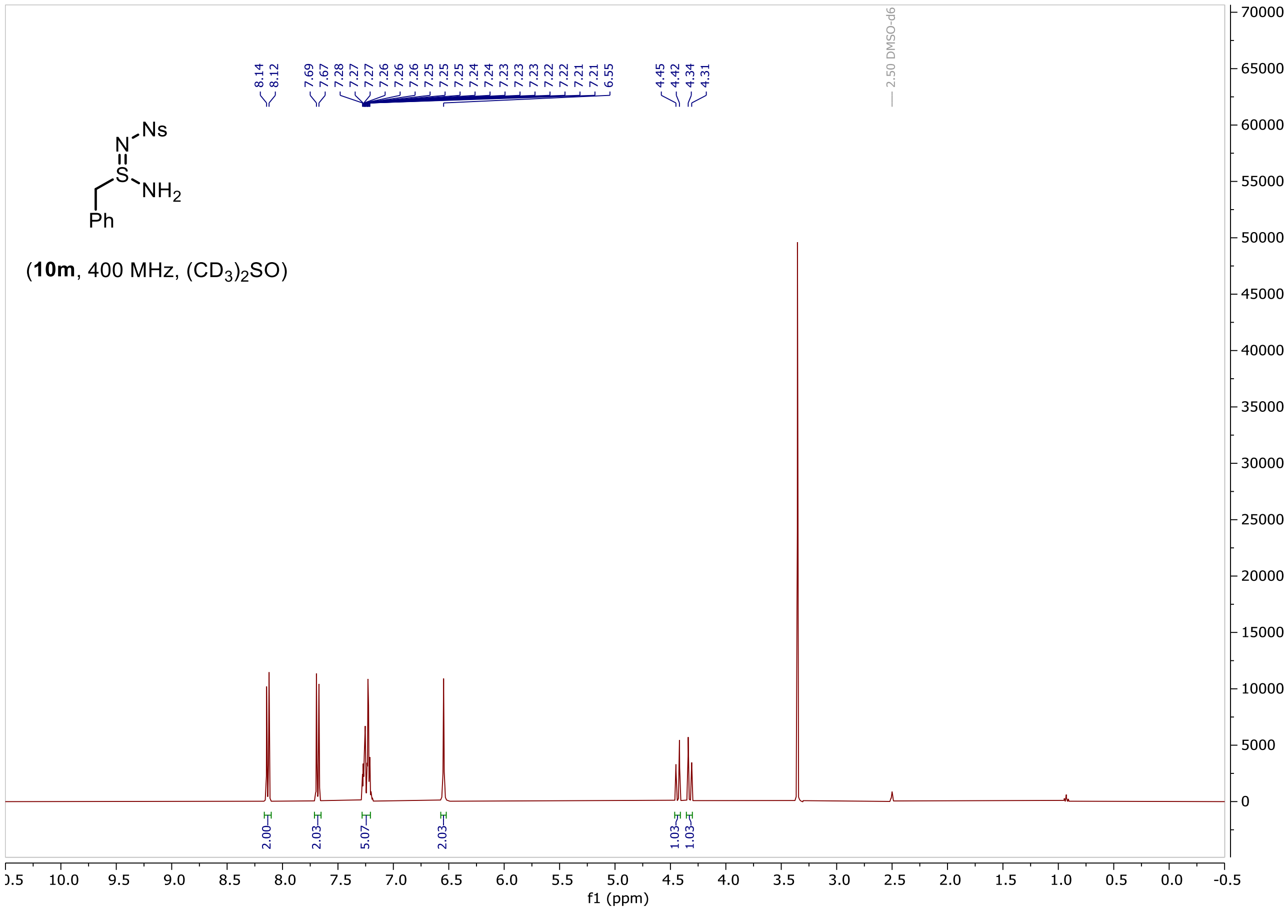

8.14
8.12
7.69
7.67
4.45
4.42
4.34
4.31
2.50 DMSO-d6
Ns
N
S
NH2
Ph
(10m, 400 MHz, (CD3)2SO)
2.00
2.03
5.07
2.03
1.03
1.03
f1 (ppm)

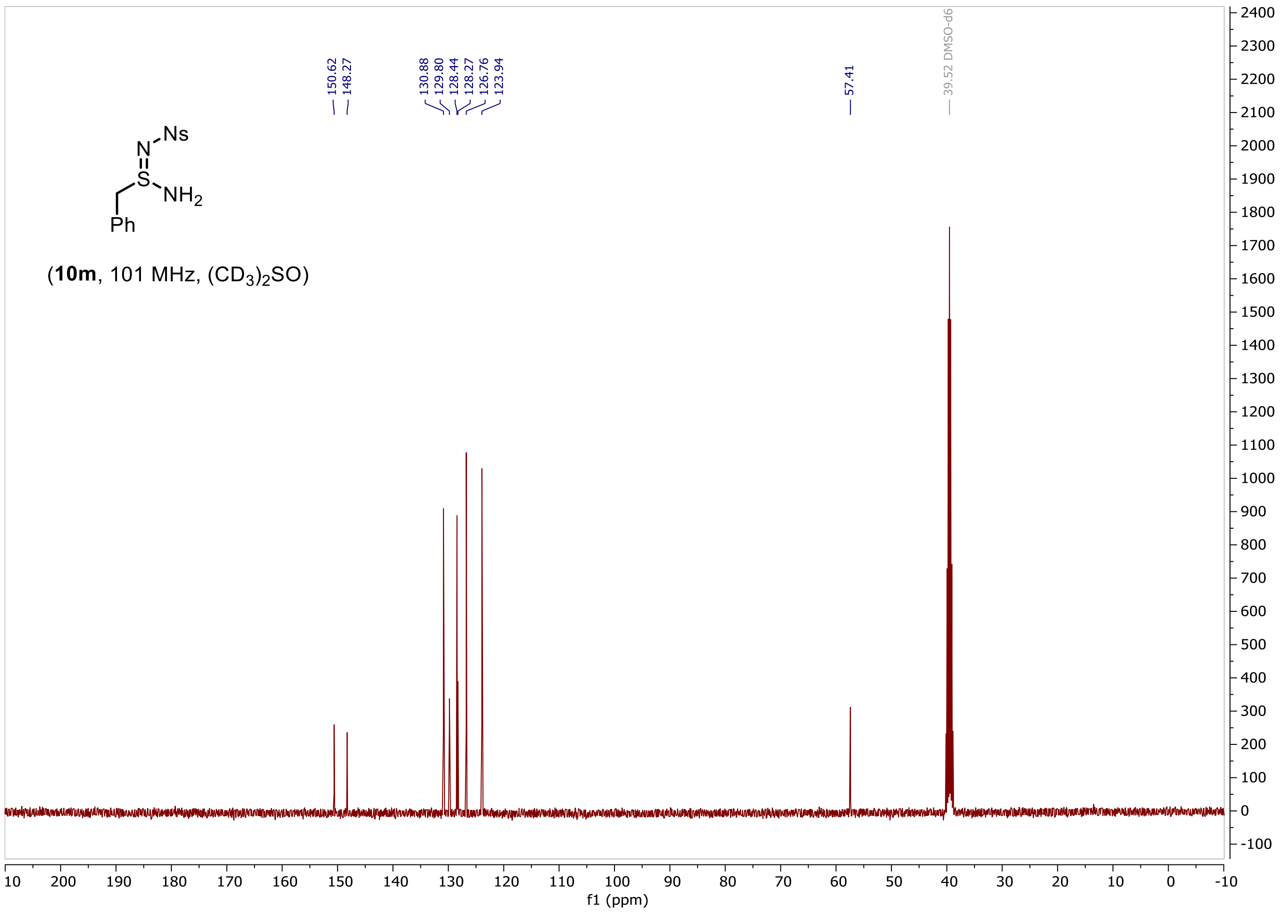

150.62
148.27
130.88
129.80
128.44
128.27
126.76
123.94
57.41
39.52 DMSO-d6
N
Ns
S
NH2
Ph
(10m, 101 MHz, (CD3)2SO)
f1 (ppm)

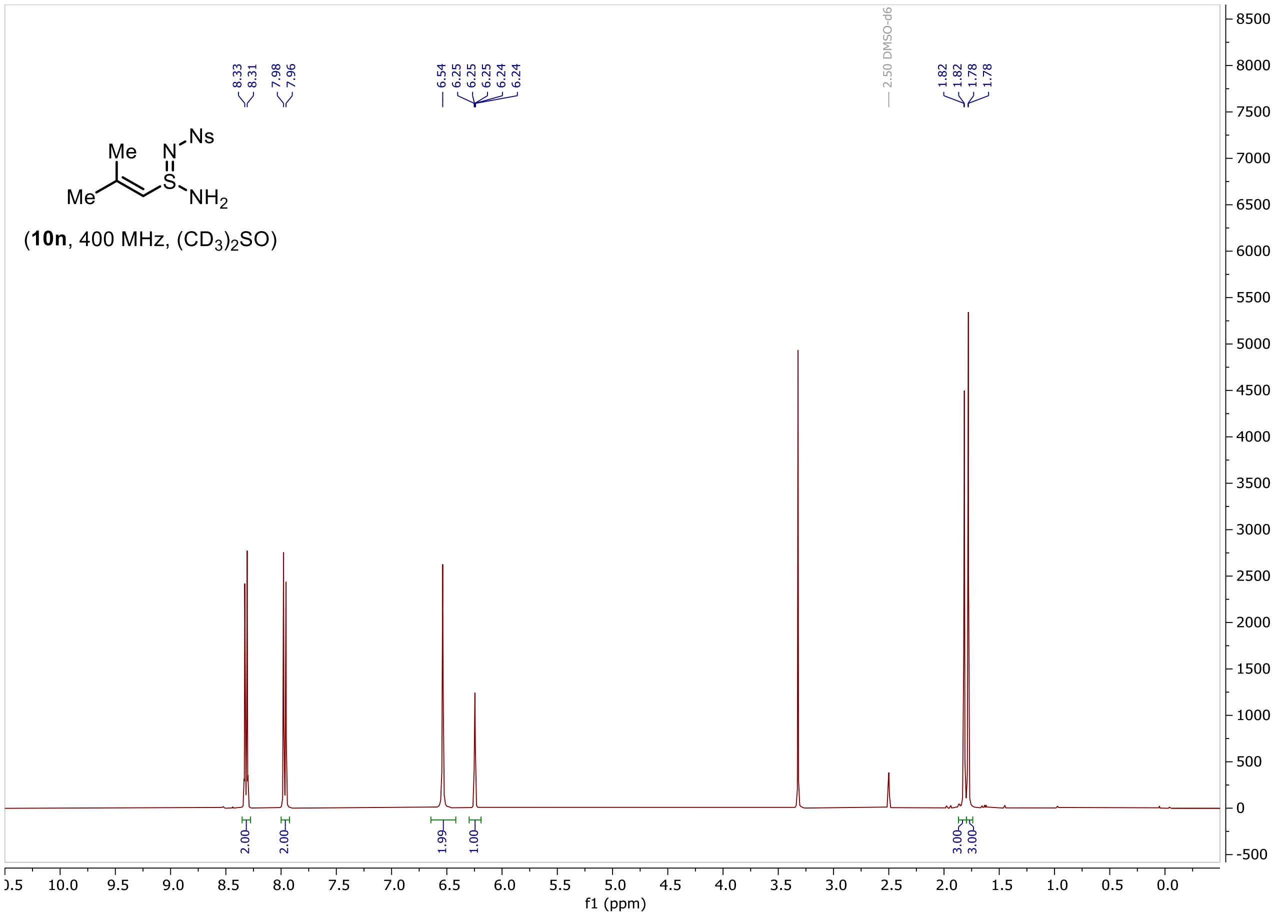

8.33
8.31
7.98
7.96
6.54
6.25
6.25
6.25
6.24
6.24
2.50 DMSO-d6
1.82
1.82
1.78
1.78
Me
Me
N
Ns
S
NH2
(10n, 400 MHz, (CD3)2SO)
2.00
2.00
1.99
1.00
3.00
3.00
f1 (ppm)

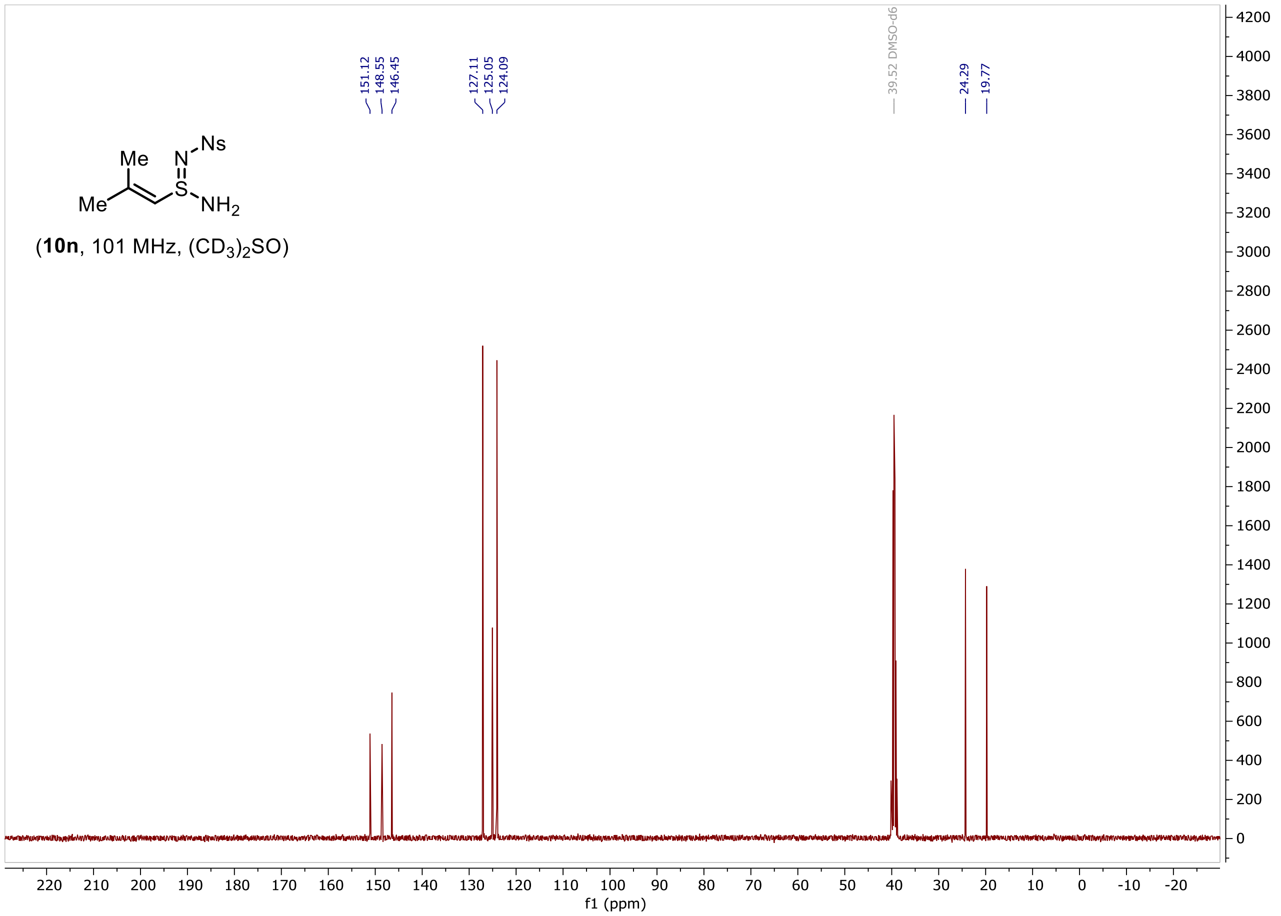

151.12
148.55
146.45
127.11
125.05
124.09
39.52 DMSO-d6
24.29
19.77
Me
N
Ns
S
NH2
Me
(10n, 101 MHz, (CD3)2SO)
f1 (ppm)

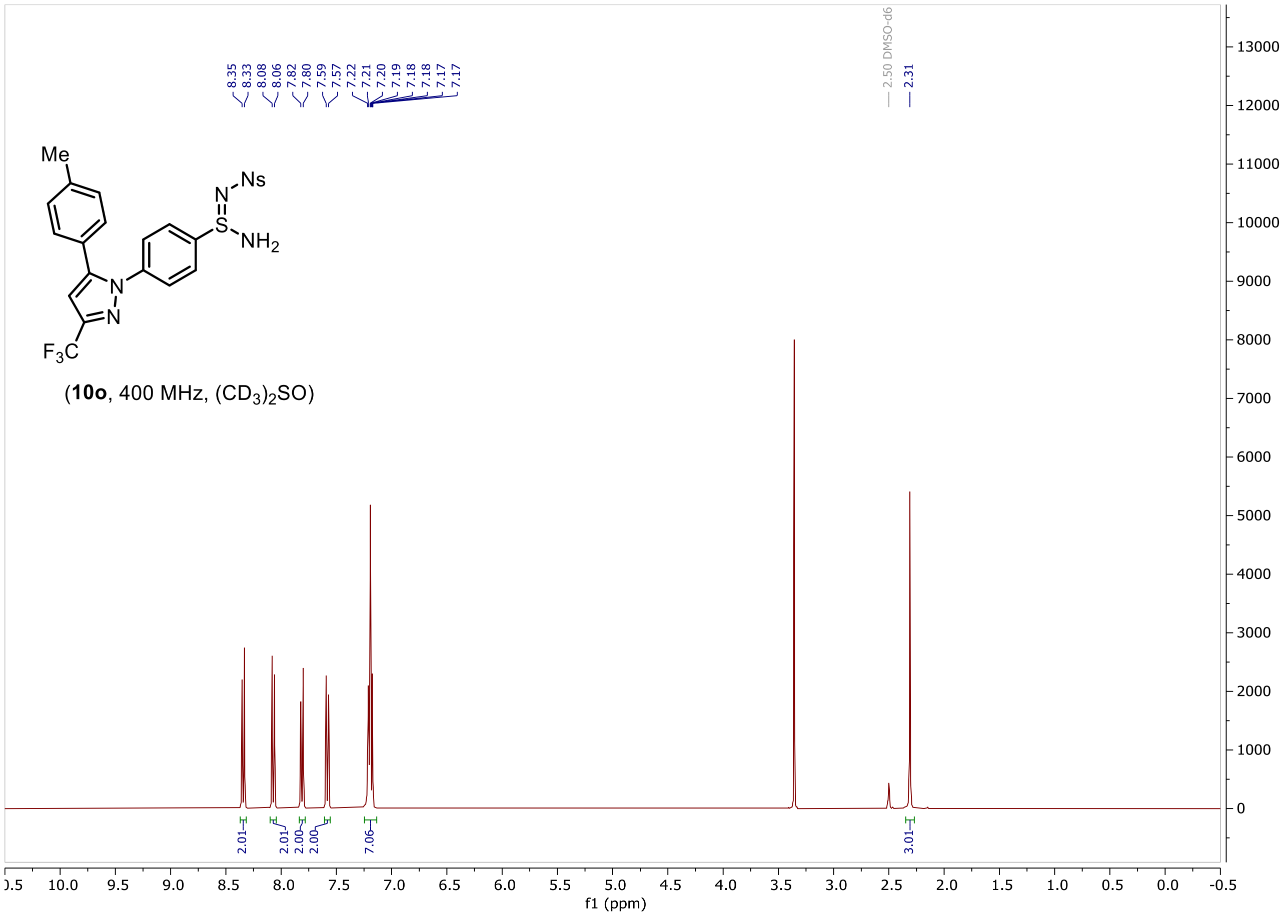

8.35
8.33
8.08
8.06
7.82
7.80
7.59
7.57
7.22
7.21
7.20
7.19
7.18
7.18
7.17
7.17
2.50 DMSO-d6
2.31
Me
N
Ns
S
NH2
N
N
F3C
(10o, 400 MHz, (CD3)2SO)
2.01
2.01
2.00
2.00
7.06
3.01
f1 (ppm)

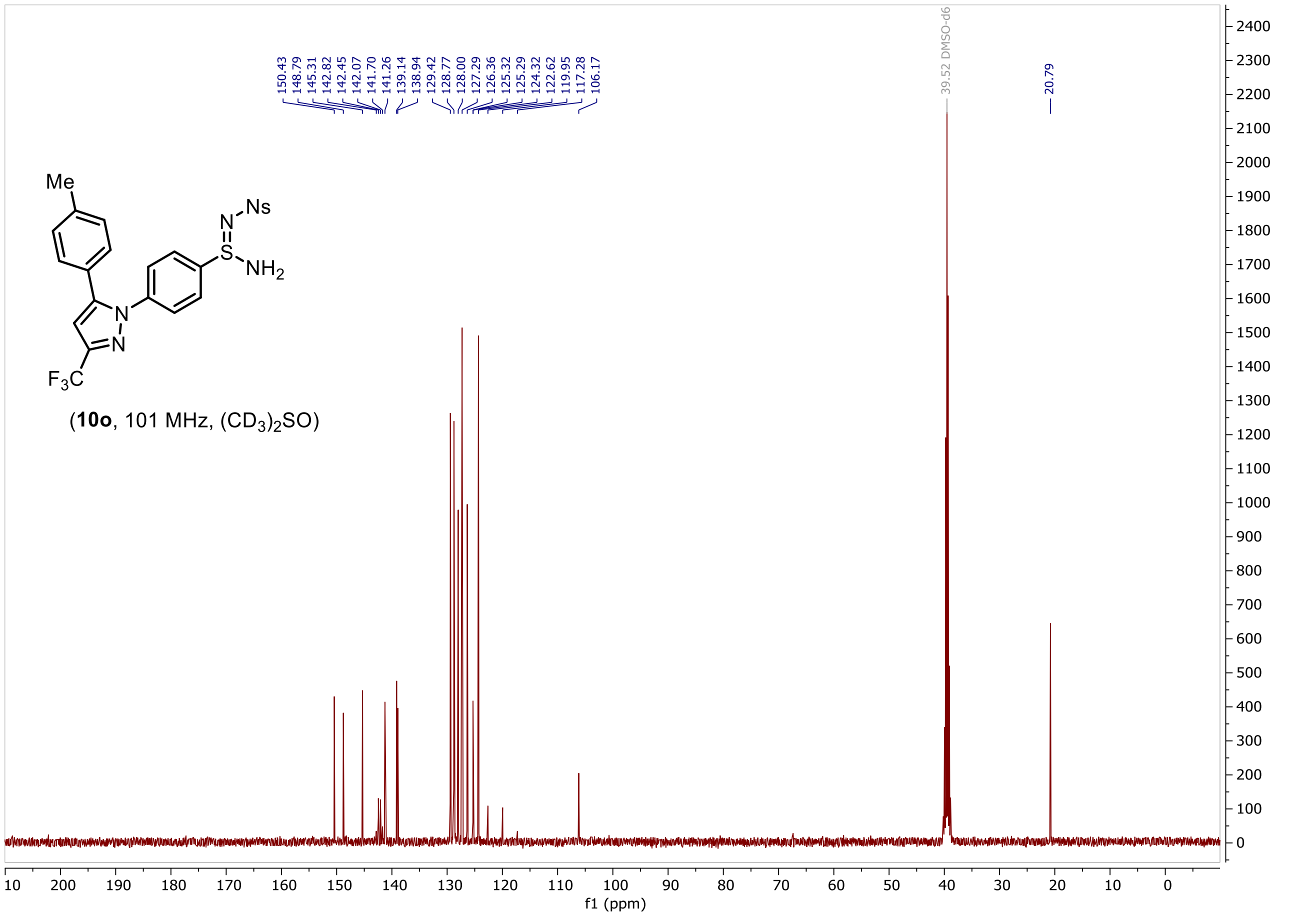
150.43
148.79
145.31
142.82
142.45
142.07
141.70
141.26
139.14
138.94
129.42
128.77
128.00
127.29
126.36
125.32
125.29
124.32
122.62
119.95
117.28
106.17
39.52 DMSO-d6
20.79
Me
N
Ns
S
NH2
N
N
F3C
(10o, 101 MHz, (CD3)2SO)
f1 (ppm)

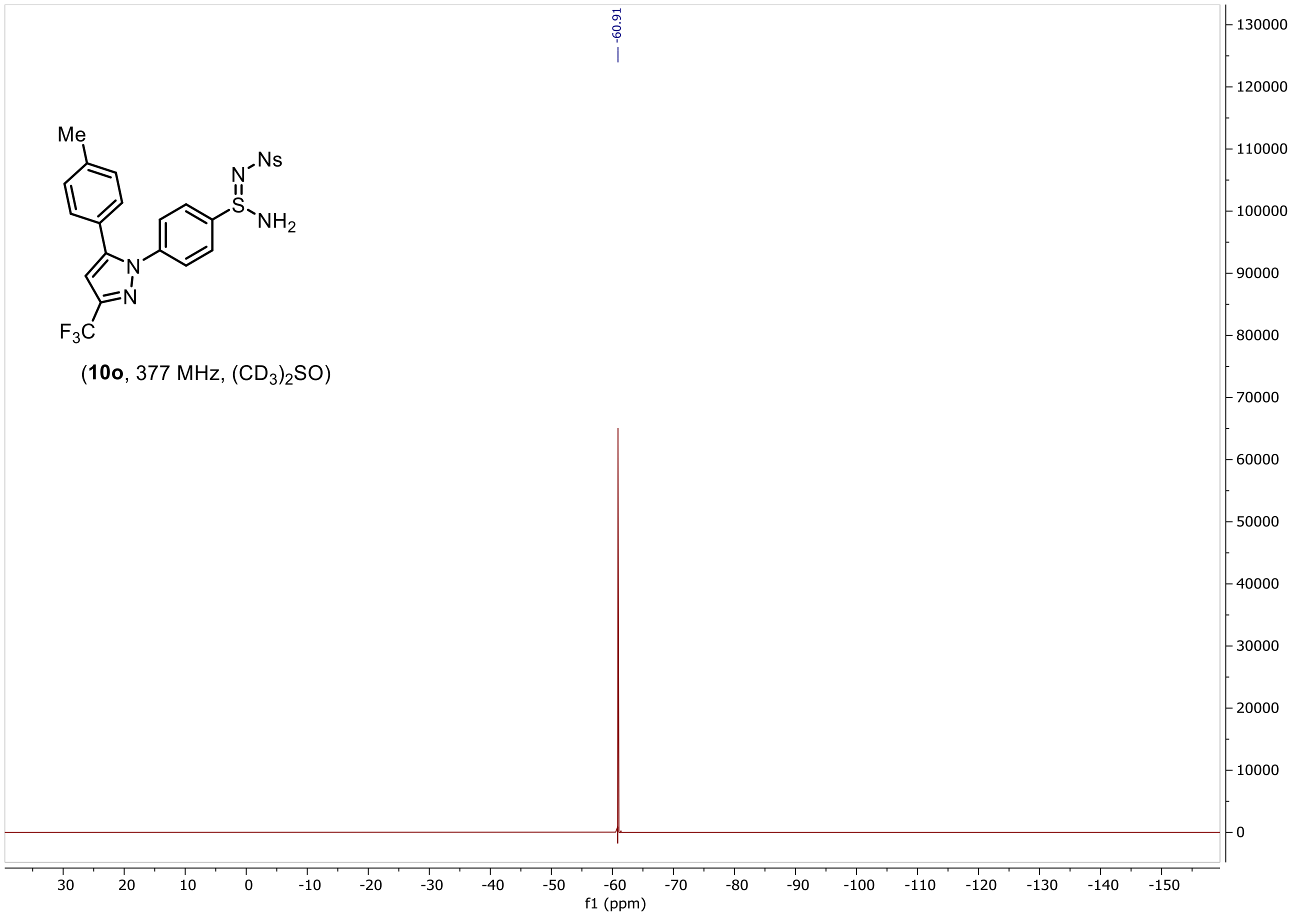
-60.91
Me
N
Ns
S
NH2
N
N
F3C
(10o, 377 MHz, (CD3)2SO)
f1 (ppm)

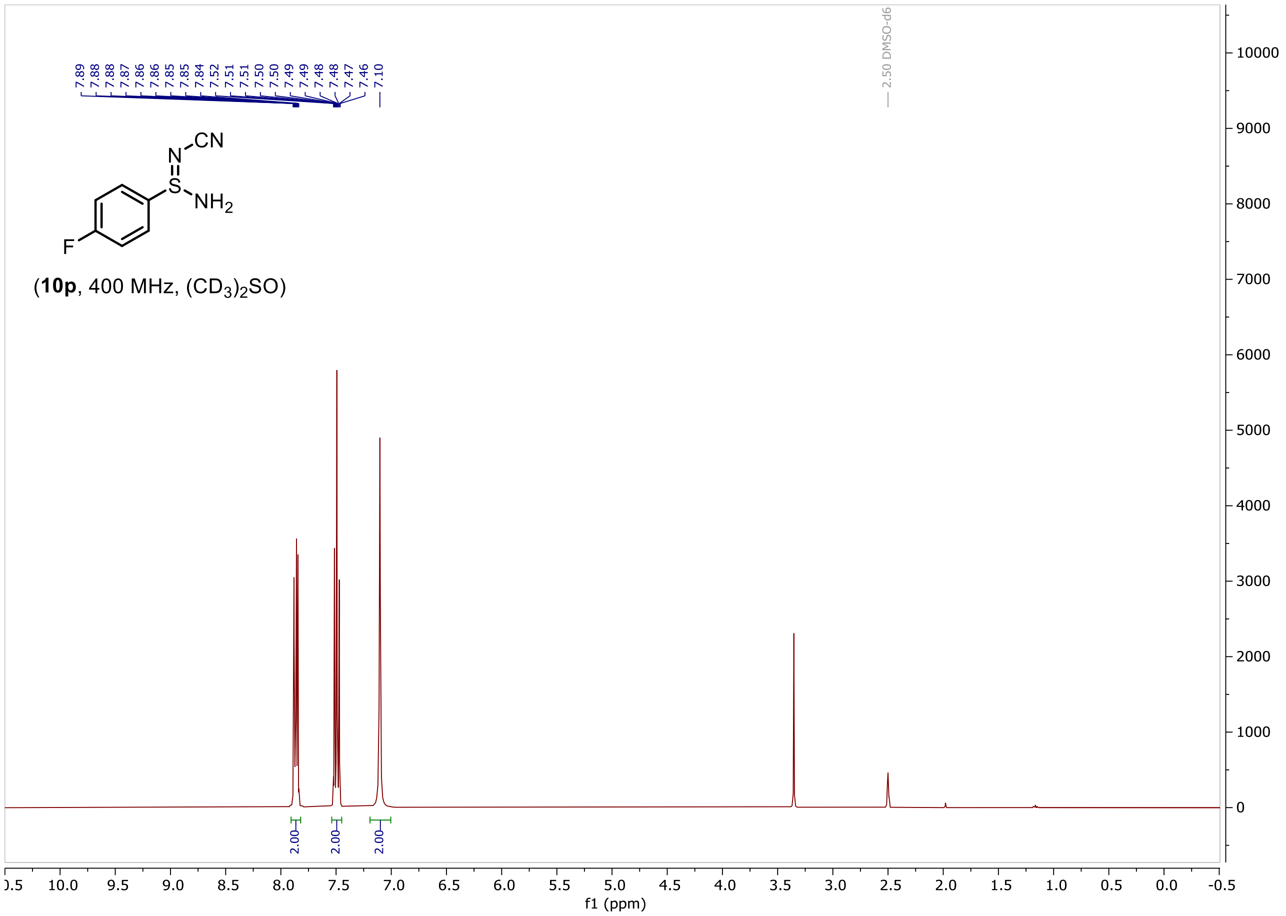
7.89
7.88
7.88
7.87
7.86
7.86
7.85
7.85
7.84
7.52
7.51
7.51
7.50
7.50
7.49
7.49
7.48
7.48
7.47
7.46
7.10
2.50 DMSO-d6
N
CN
S
NH2
F
(10p, 400 MHz, (CD3)2SO)
2.00
2.00
2.00
f1 (ppm)

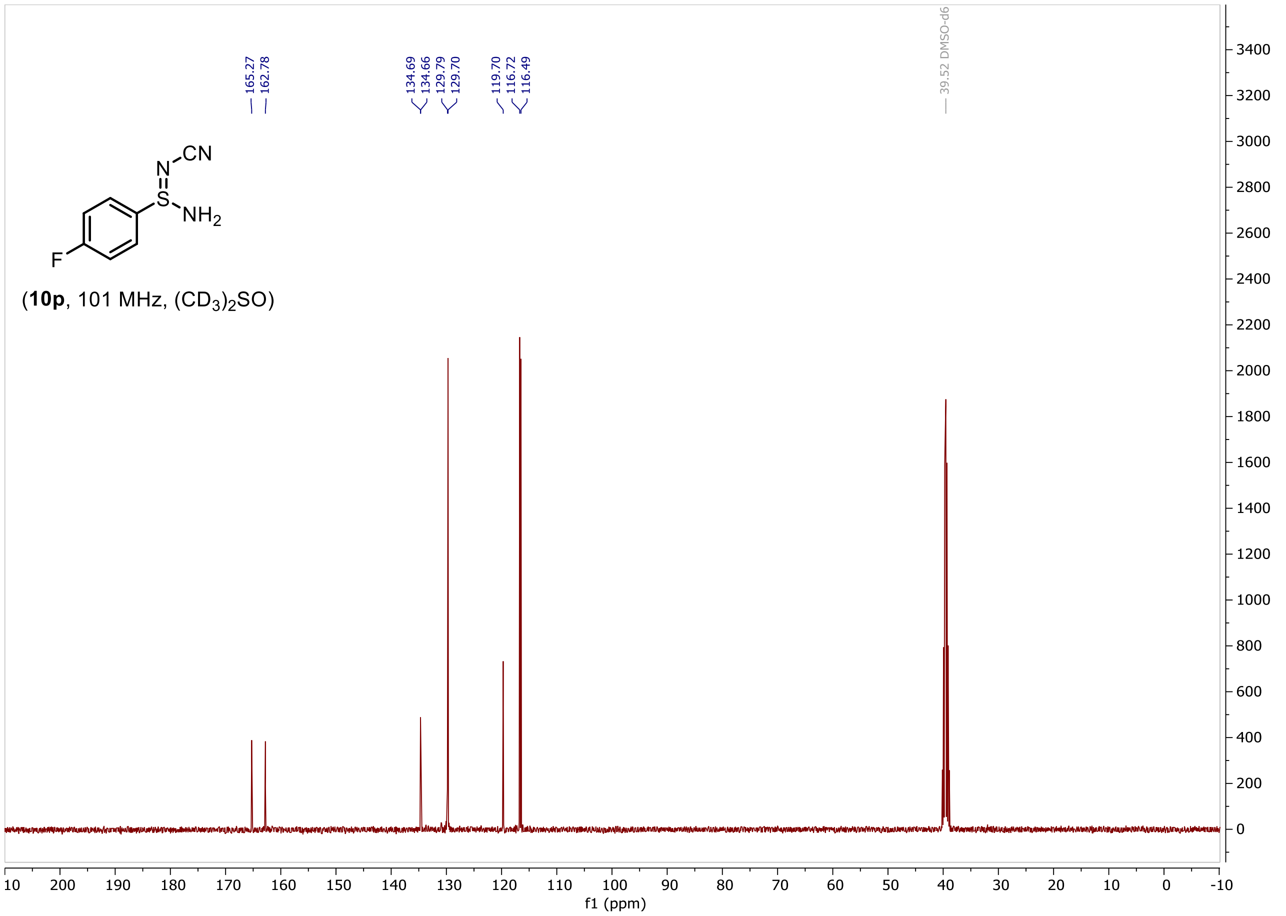
165.27
162.78
134.69
134.66
129.79
129.70
119.70
116.72
116.49
39.52 DMSO-d6
N
CN
S
NH2
F
(10p, 101 MHz, (CD3)2SO)
f1 (ppm)

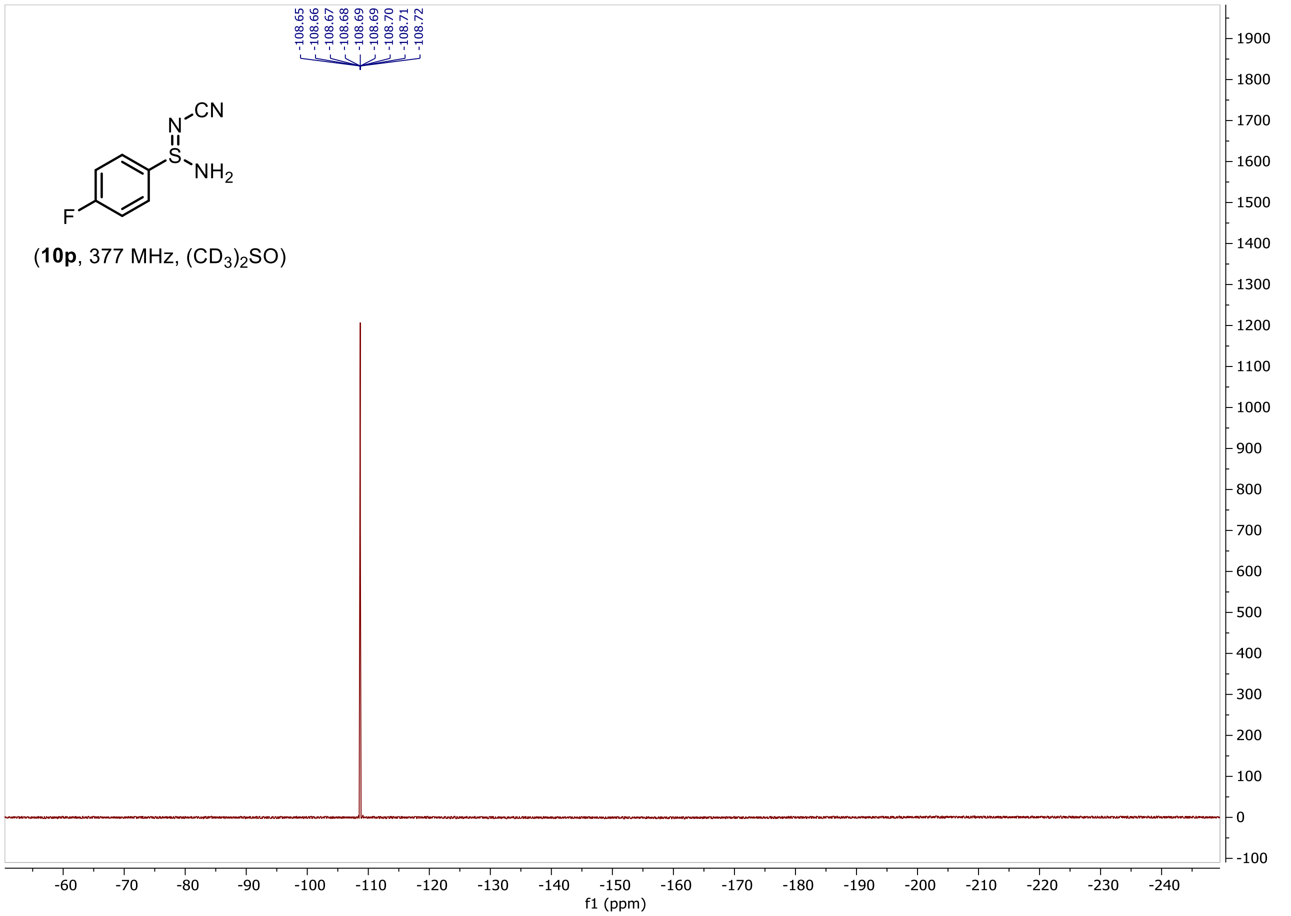

-108.65
-108.66
-108.67
-108.68
-108.69
-108.69
-108.70
-108.71
-108.72
N
CN
S
NH2
F
(10p, 377 MHz, (CD3)2SO)
f1 (ppm)

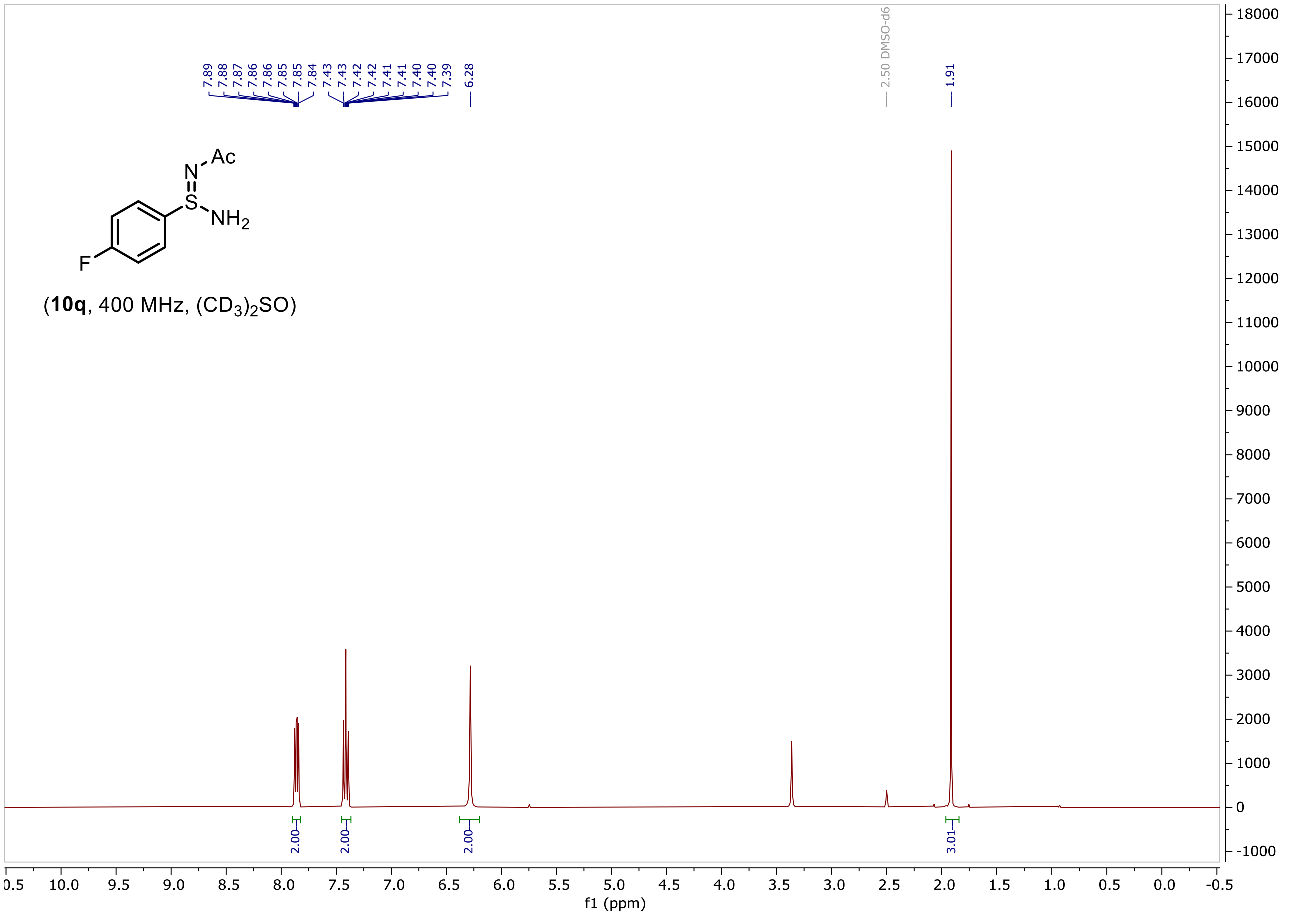

7.89
7.88
7.87
7.86
7.86
7.85
7.85
7.84
7.43
7.43
7.42
7.42
7.41
7.41
7.40
7.40
7.39
6.28
2.50 DMSO-d6
1.91
N
Ac
S
NH2
F
(10q, 400 MHz, (CD3)2SO)
2.00
2.00
2.00
3.01
f1 (ppm)

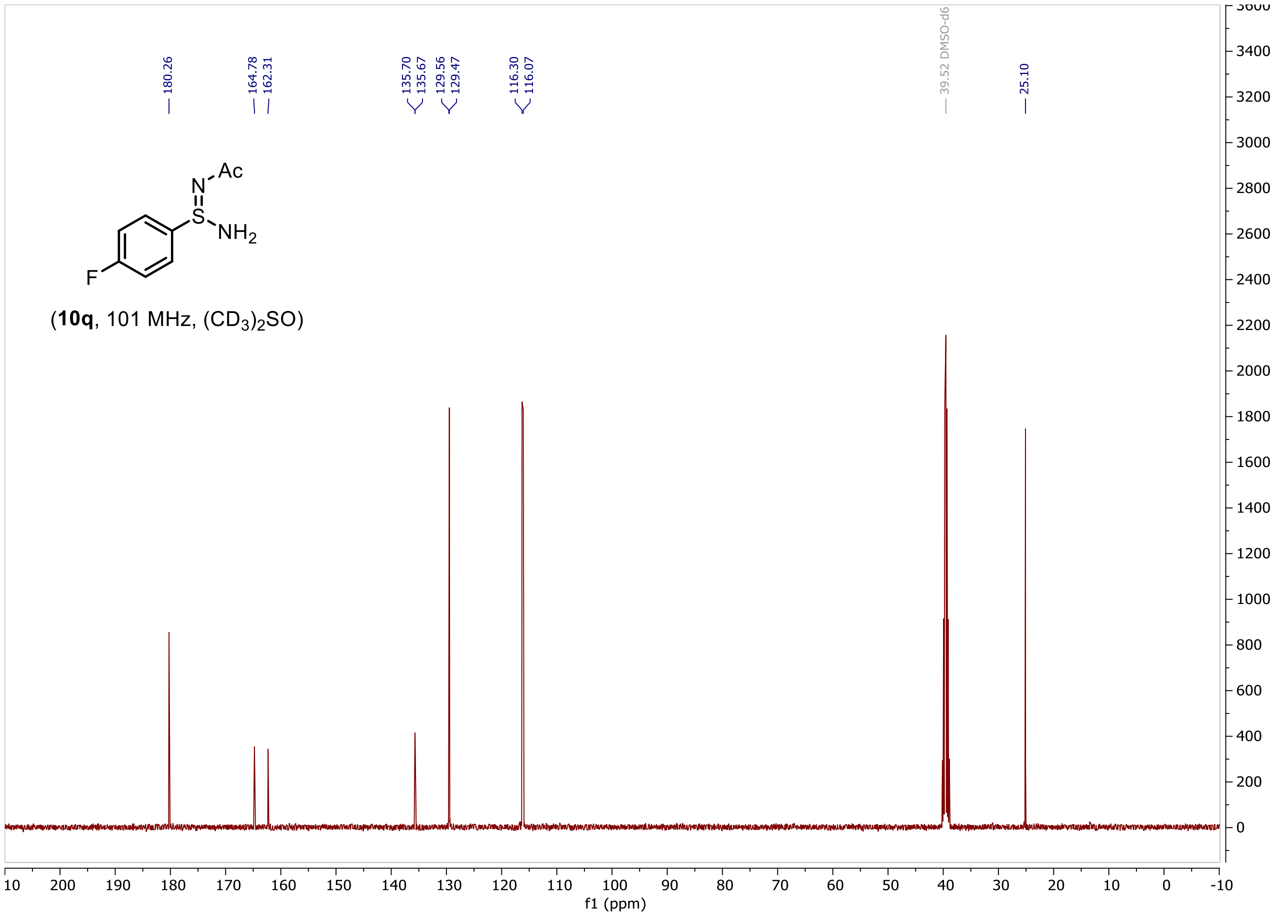
180.26
164.78
162.31
135.70
135.67
129.56
129.47
116.30
116.07
39.52 DMSO-d6
25.10
N
Ac
S
NH2
F
(10q, 101 MHz, (CD3)2SO)
f1 (ppm)

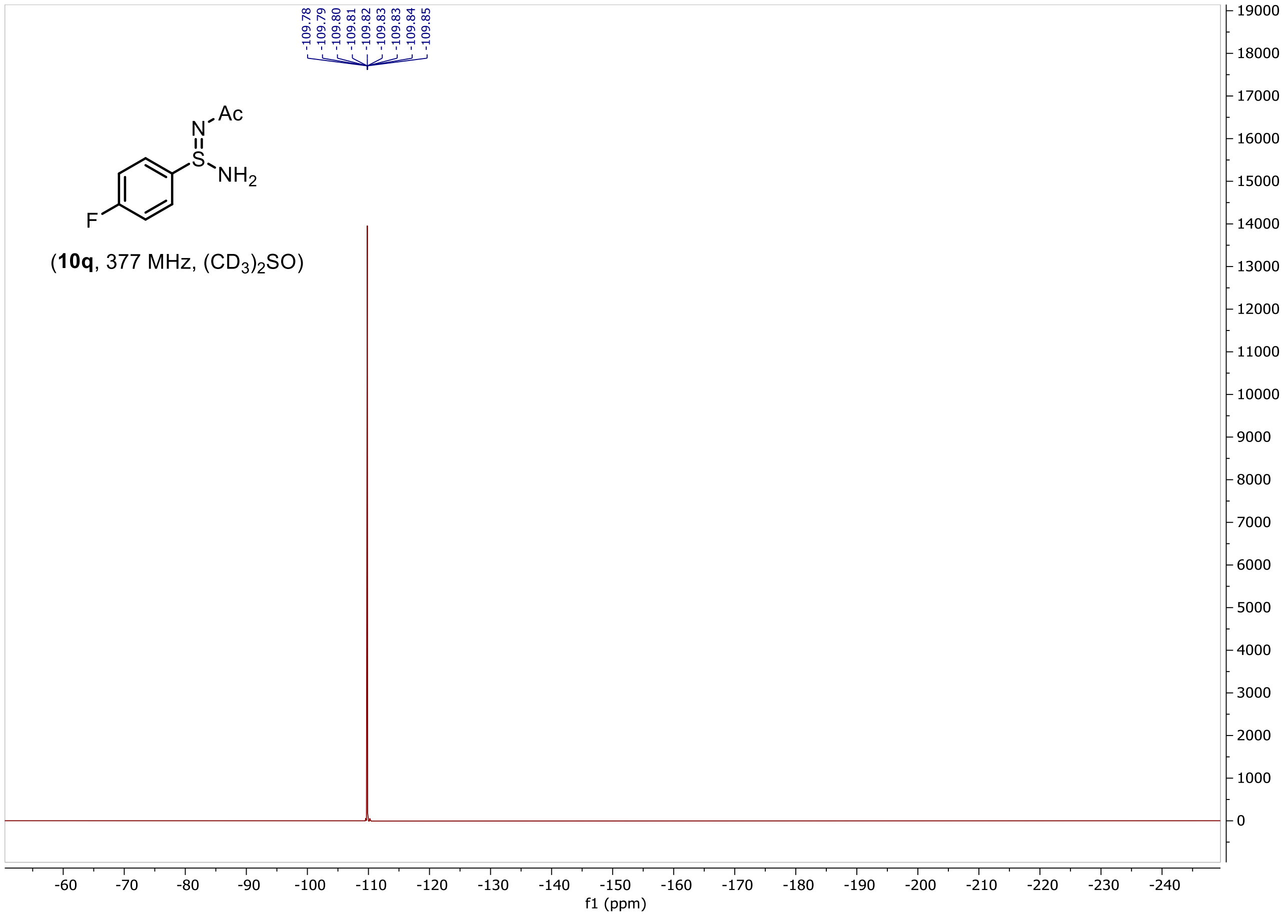
-109.78
-109.79
-109.80
-109.81
-109.82
-109.83
-109.83
-109.84
-109.85
N
Ac
S
NH2
F
(10q, 377 MHz, (CD3)2SO)
f1 (ppm)

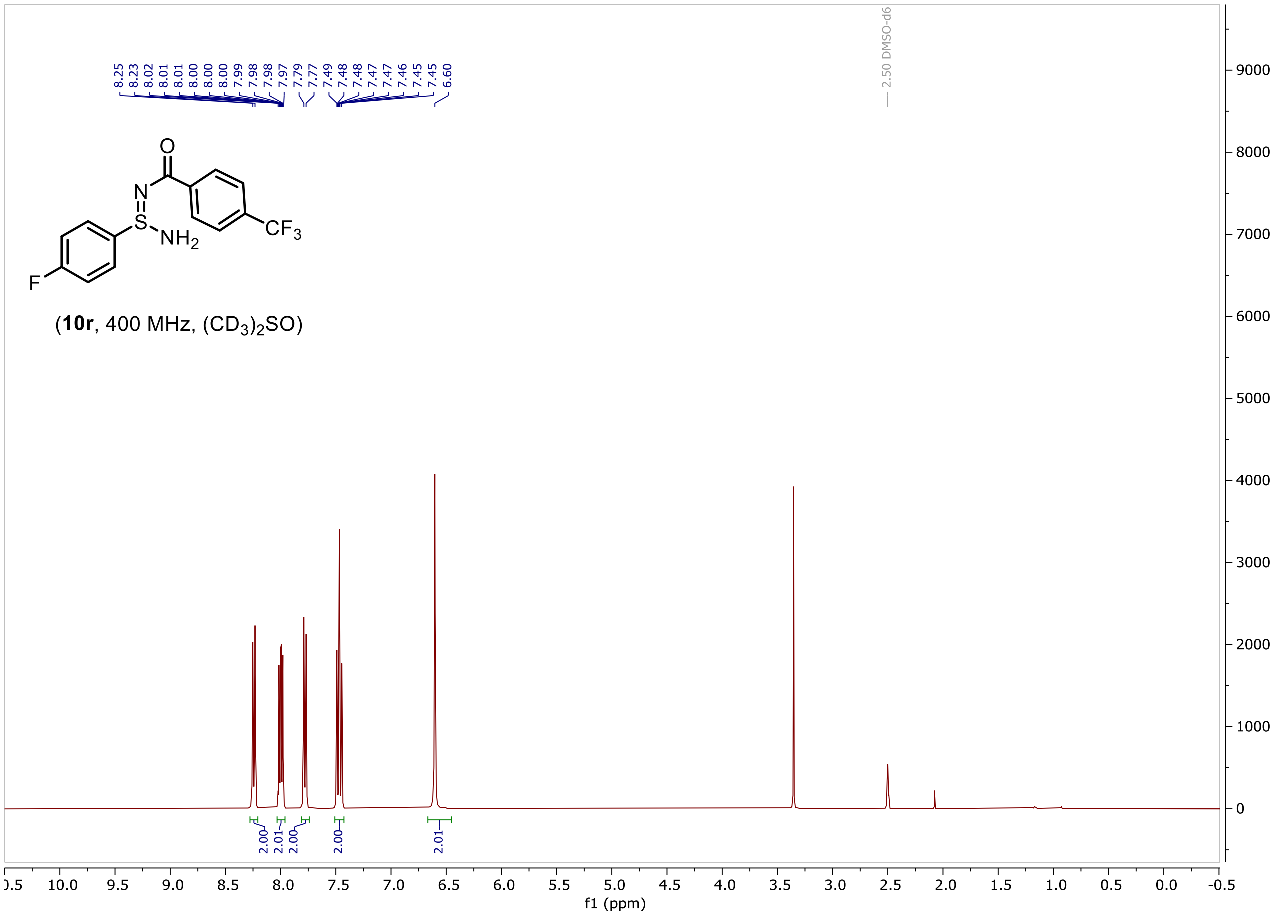
8.25 8.23 8.02 8.01 8.01 8.00 8.00 8.00 7.99 7.98 7.98 7.97 7.79 7.77 7.49 7.48 7.48 7.47 7.47 7.46 7.45 7.45 6.60
2.50 DMSO-d6
O
N
S
NH2
CF3
F
(10r, 400 MHz, (CD3)2SO)
2.00 2.01 2.00 2.00 2.01
f1 (ppm)

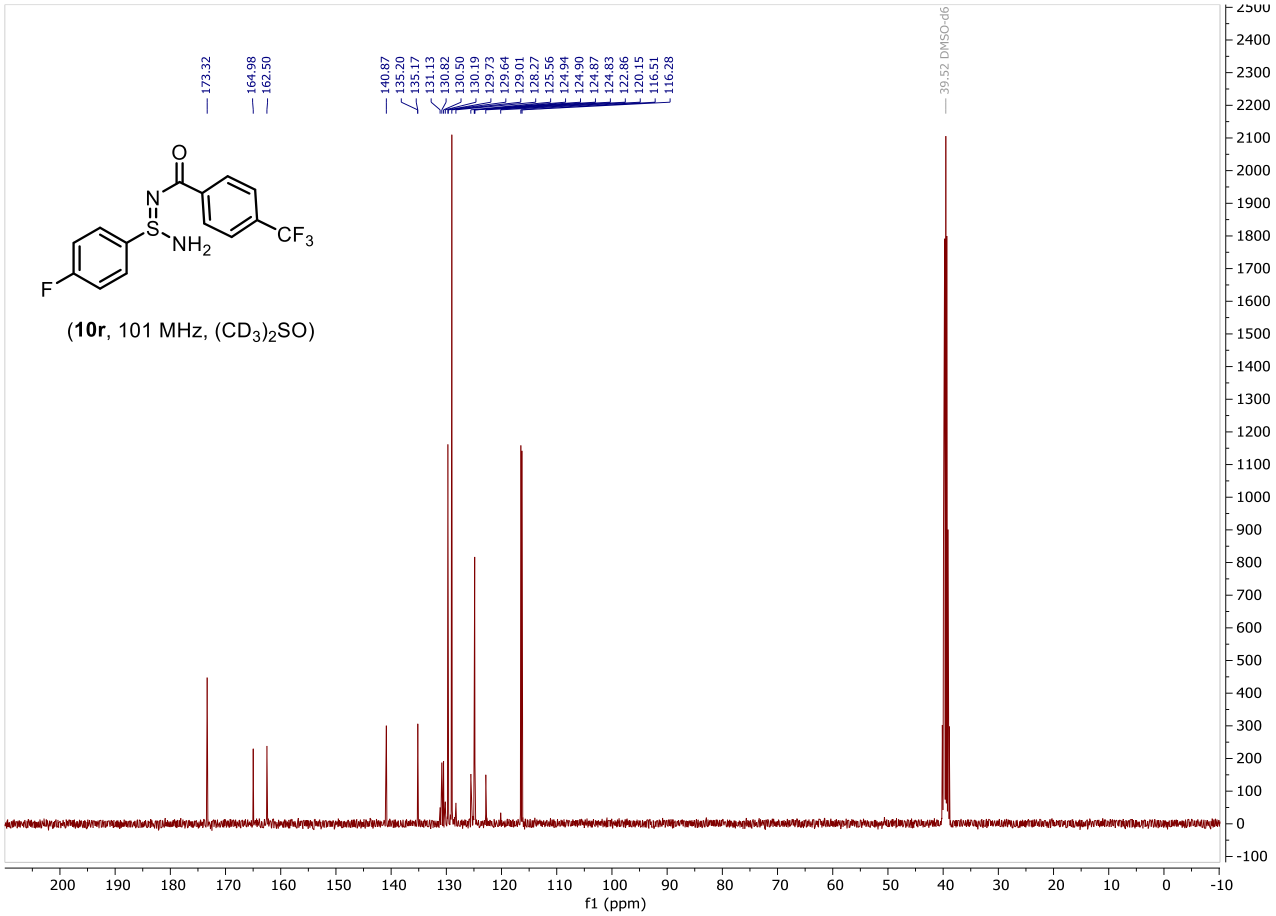
173.32
164.98 162.50
140.87 135.20 135.17 131.13 130.82 130.50 130.19 129.73 129.64 129.01 128.27 125.56 124.94 124.90 124.87 124.83 122.86 120.15 116.51 116.28
39.52 DMSO-d6
O
N
S
NH2
CF3
F
(10r, 101 MHz, (CD3)2SO)
f1 (ppm)

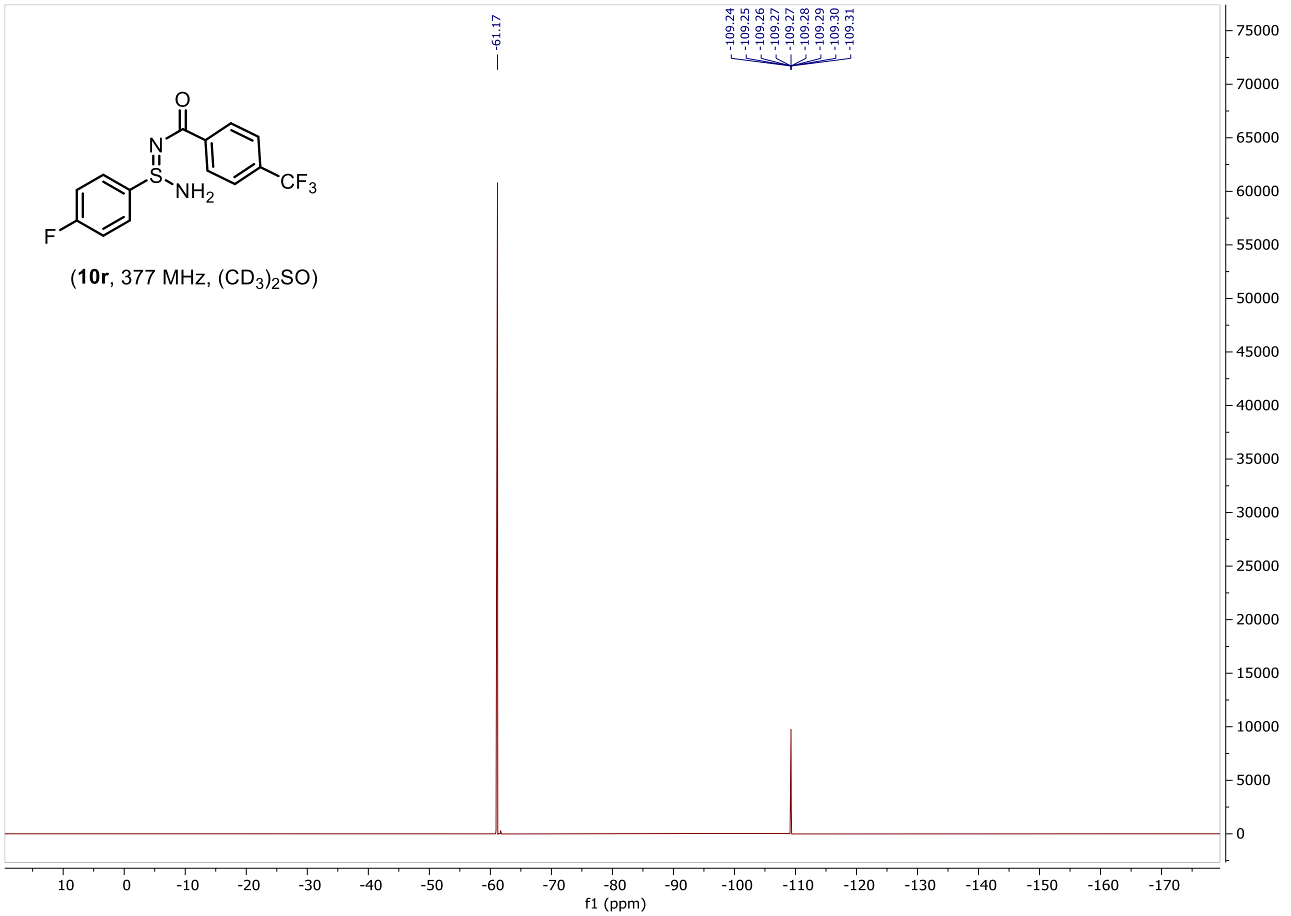
(**10r**, 377 MHz, $(CD_3)_2SO$)
-61.17
-109.24
-109.25
-109.26
-109.27
-109.27
-109.28
-109.29
-109.30
-109.31
f1 (ppm)

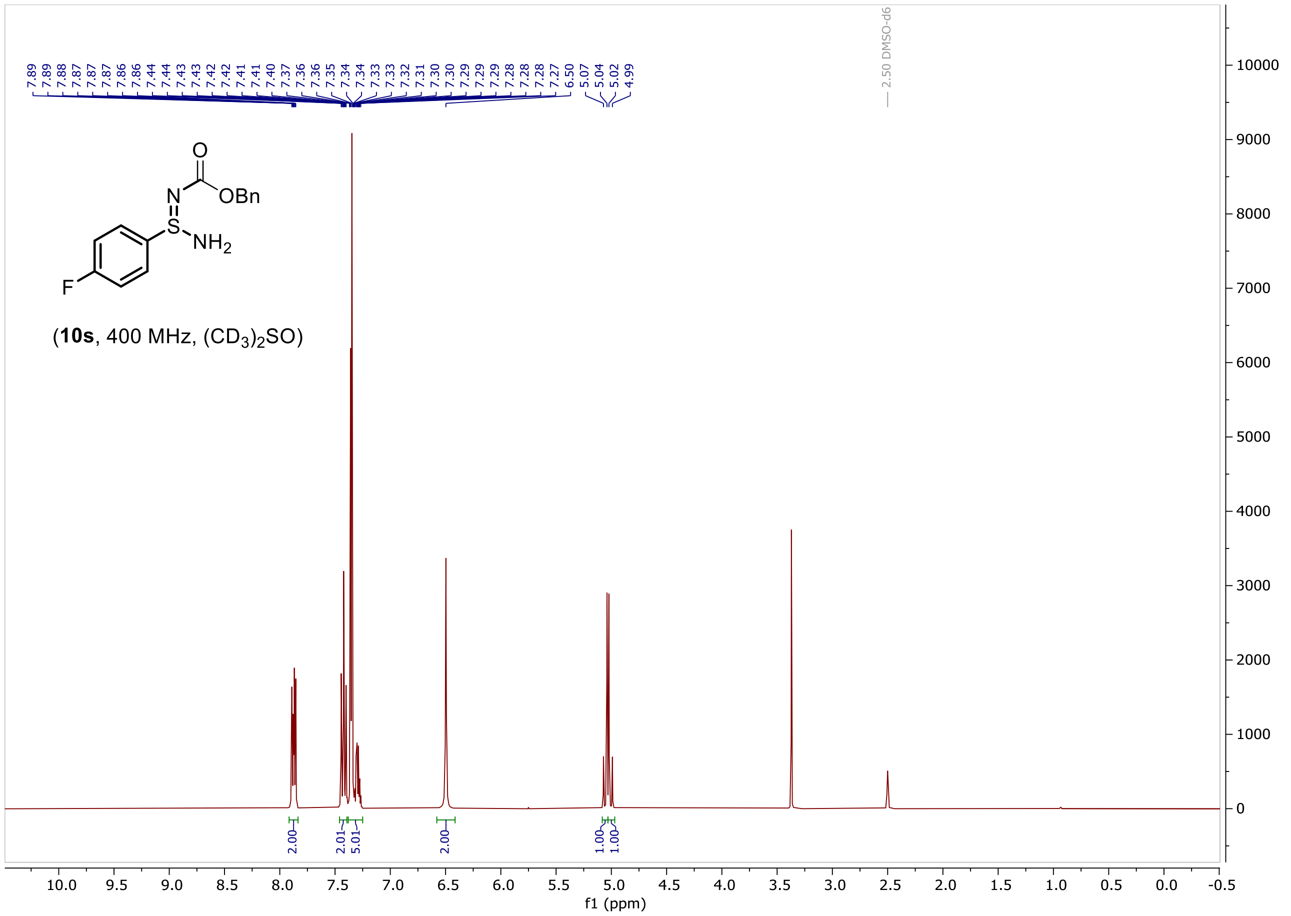
(**10s**, 400 MHz, $(CD_3)_2SO$)
2.50 DMSO-d6
f1 (ppm)

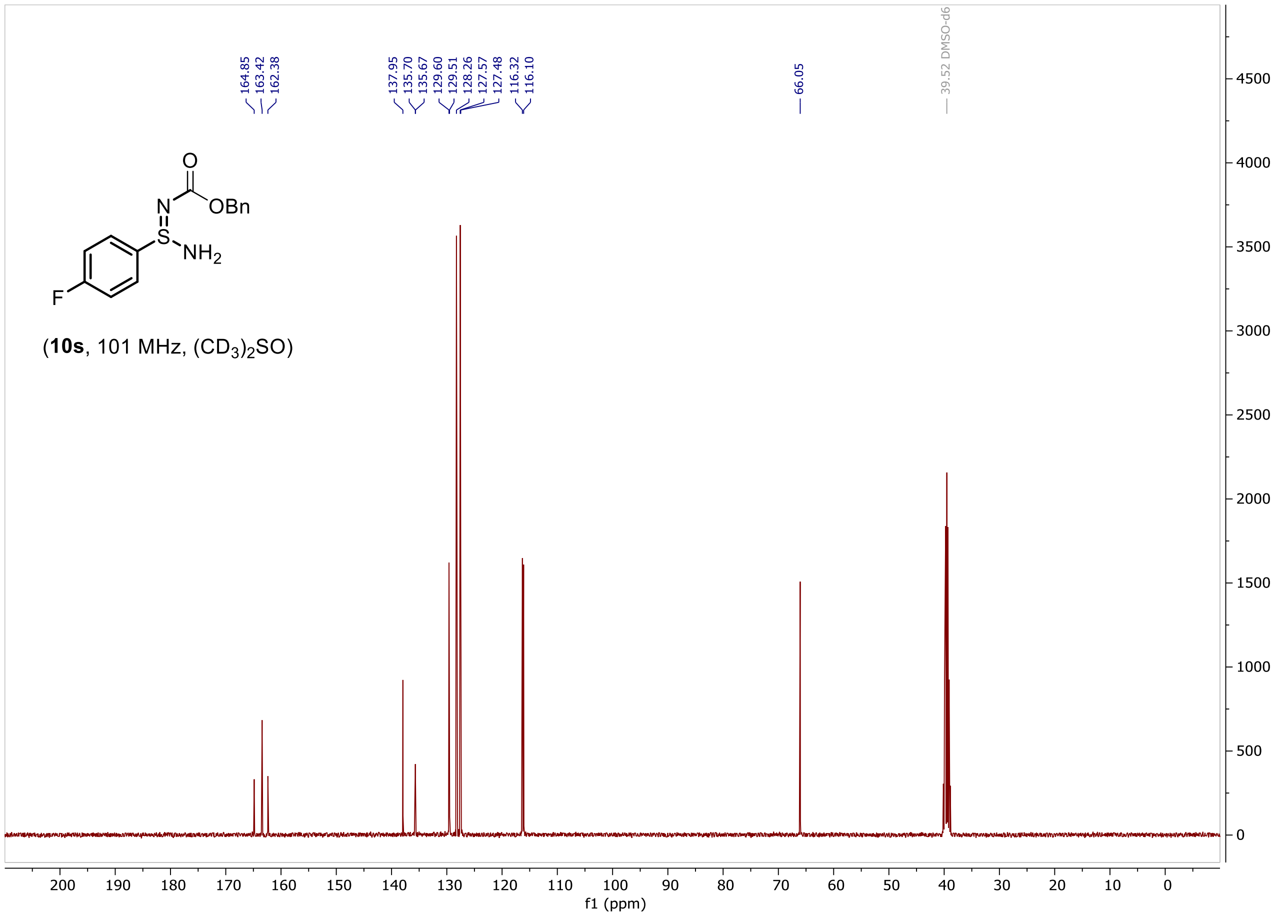
164.85
163.42
162.38
137.95
135.70
135.67
129.60
129.51
128.26
127.57
127.48
116.32
116.10
66.05
39.52 DMSO-d6
O
N
OBn
S
NH2
F
(10s, 101 MHz, (CD3)2SO)
f1 (ppm)

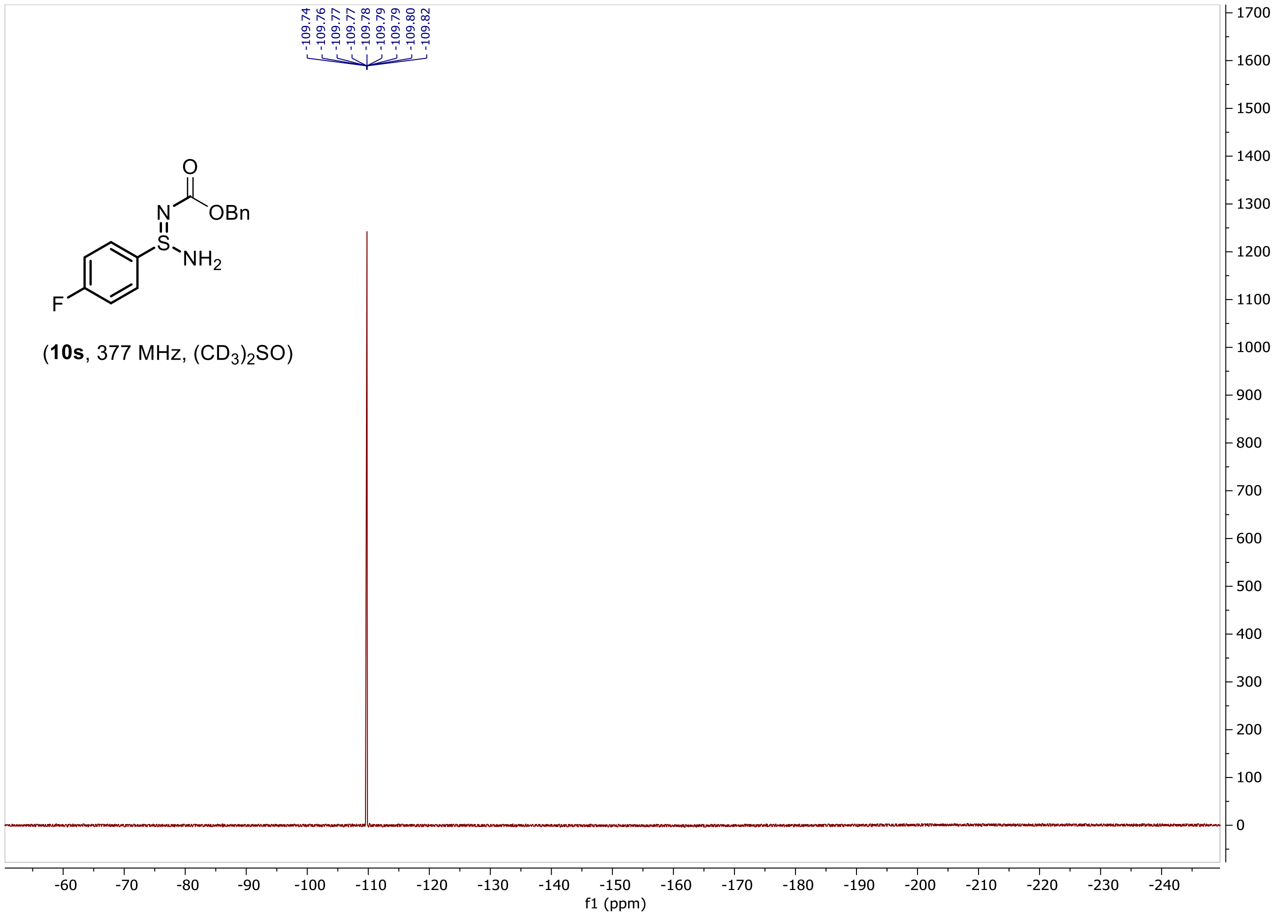
-109.74
-109.76
-109.77
-109.77
-109.78
-109.79
-109.79
-109.80
-109.82
O
N
OBn
S
NH2
F
(10s, 377 MHz, (CD3)2SO)
f1 (ppm)

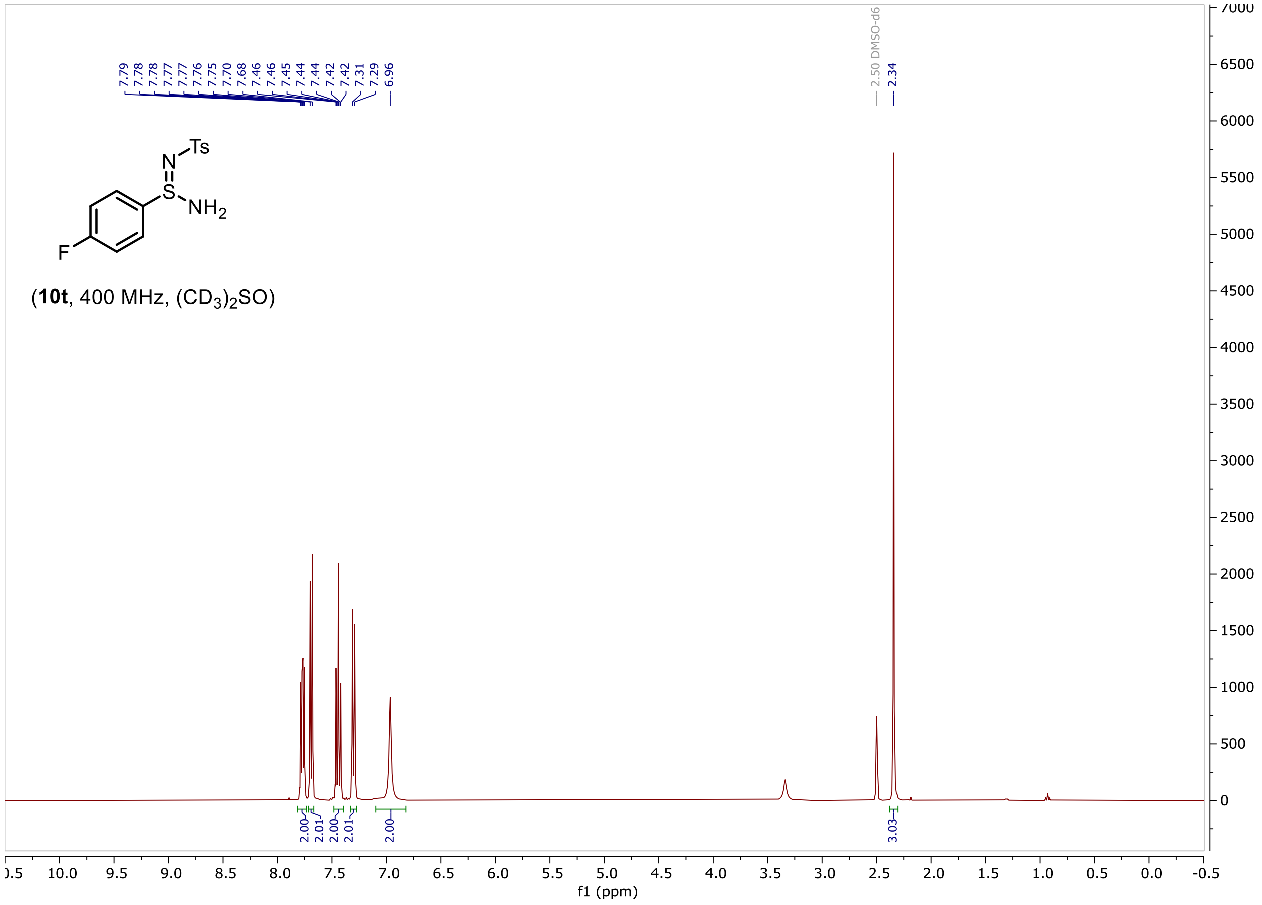

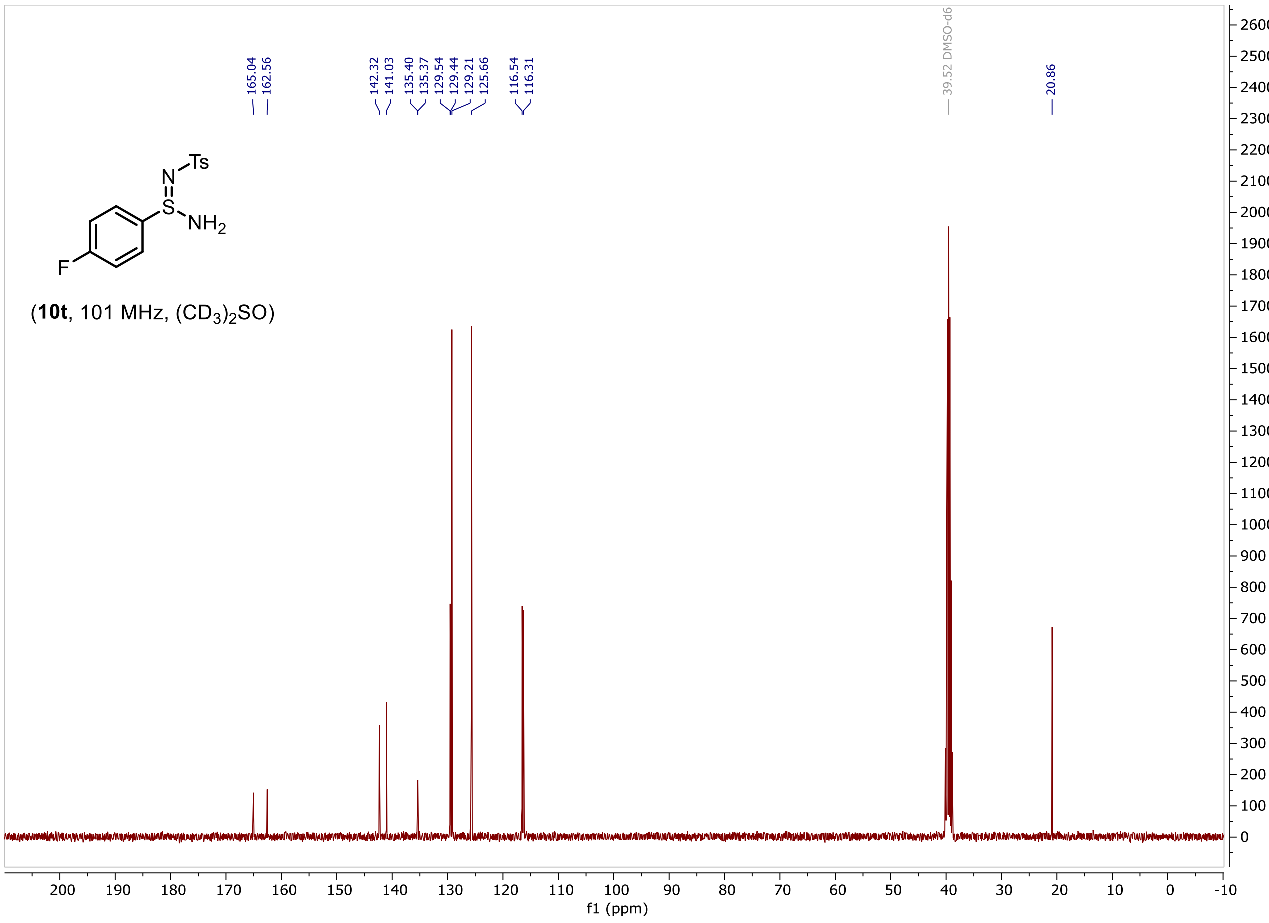

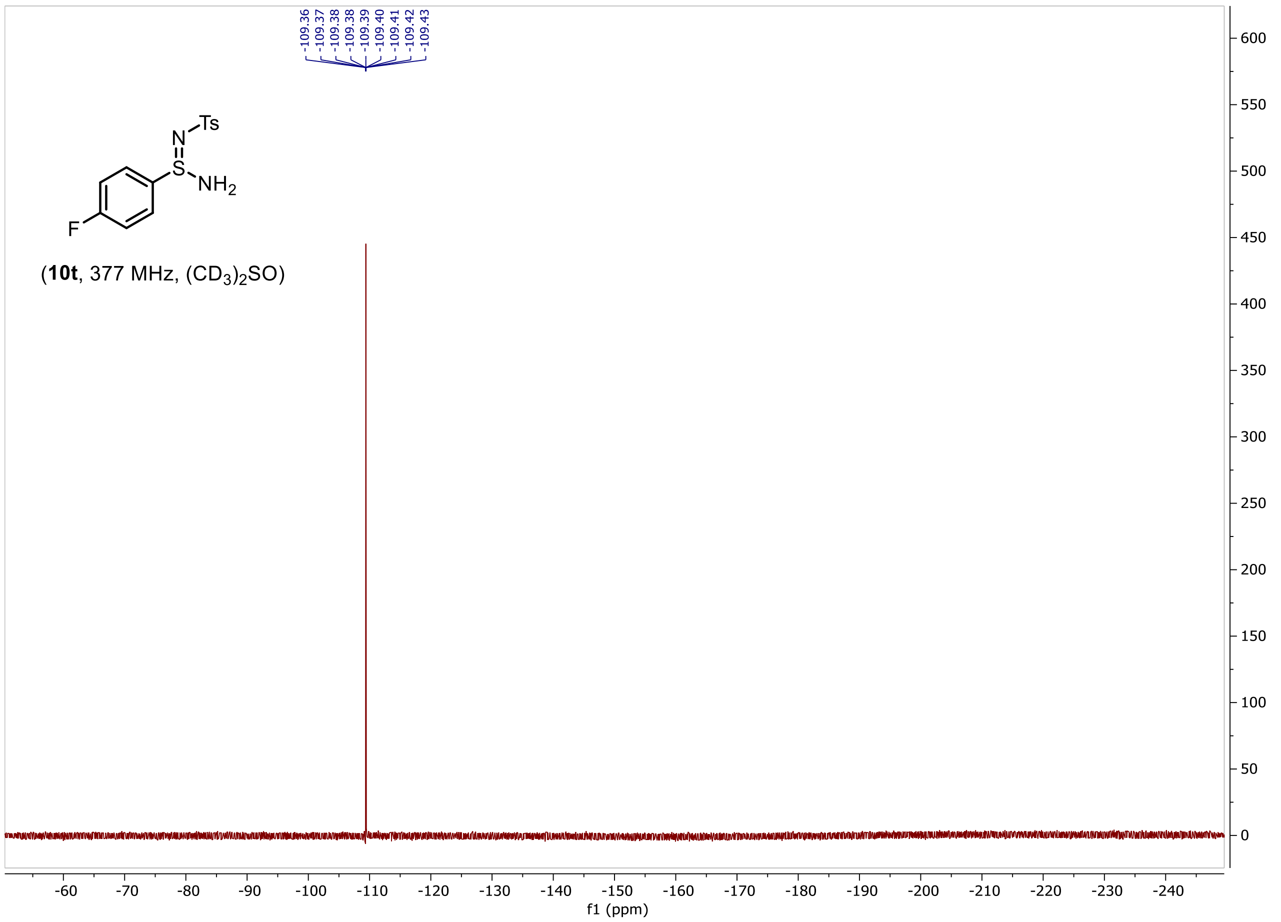
(10t, 377 MHz, (CD3)2SO)
f1 (ppm)

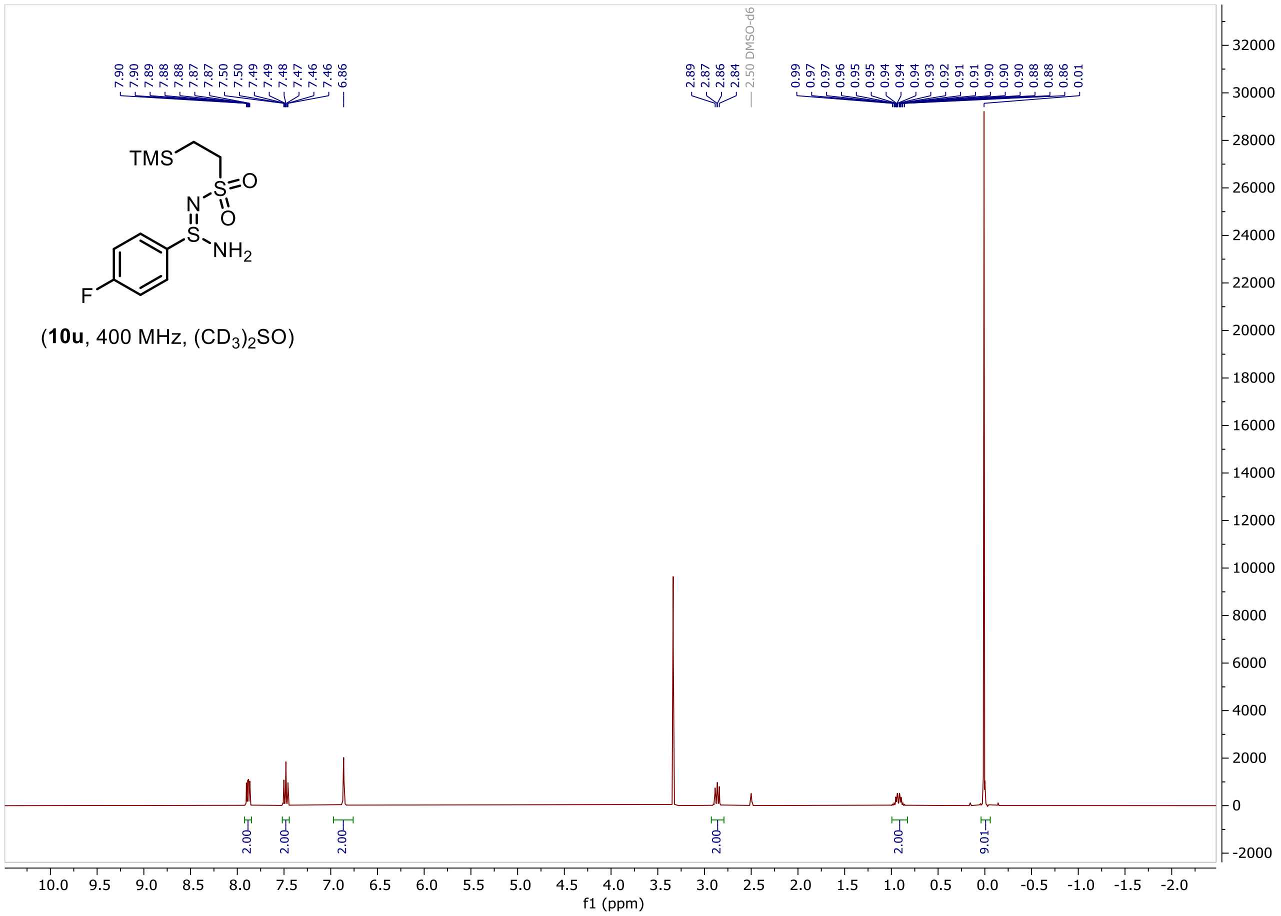
(10u, 400 MHz, (CD3)2SO)
2.50 DMSO-d6
f1 (ppm)

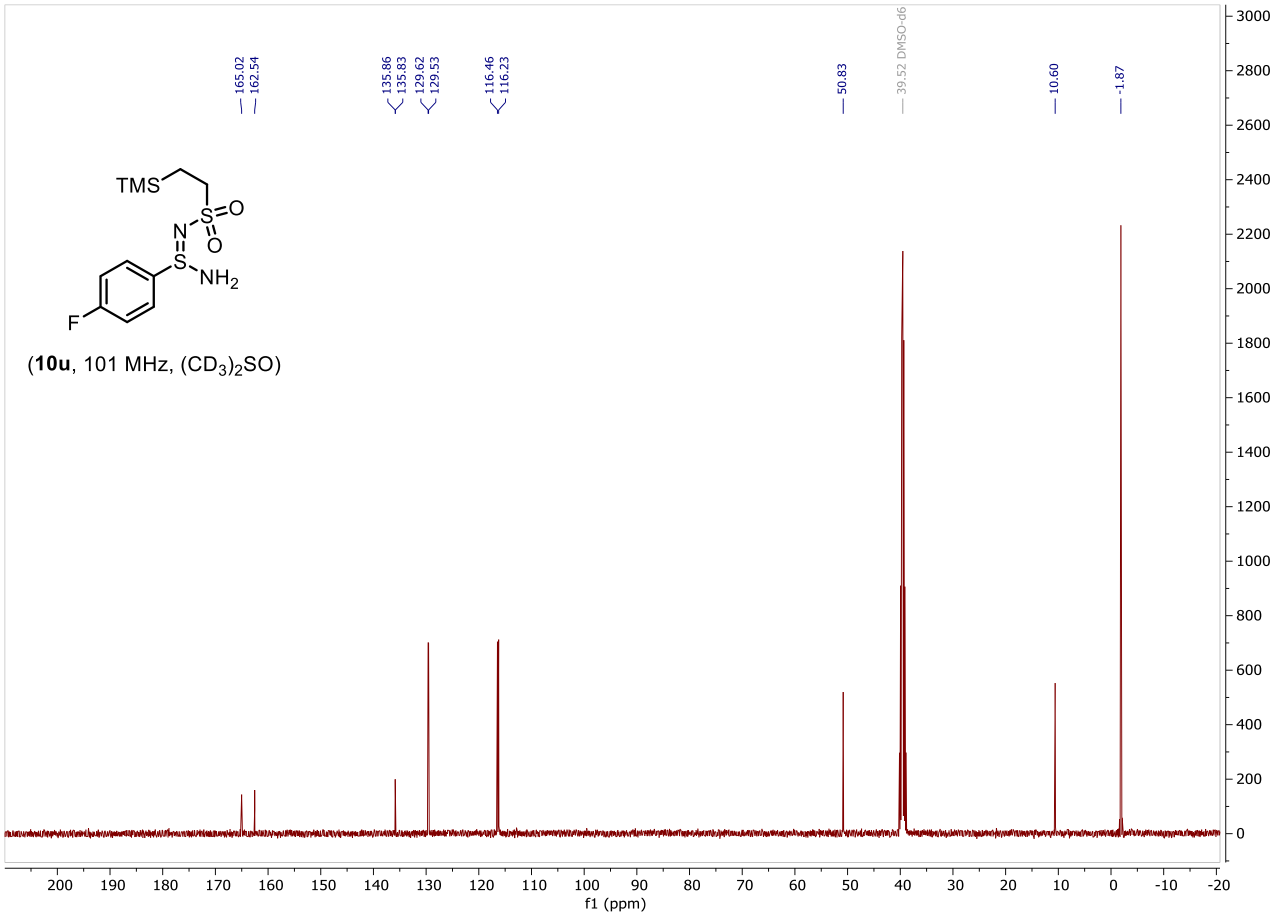
165.02
162.54
135.86
135.83
129.62
129.53
116.46
116.23
50.83
39.52 DMSO-d6
10.60
-1.87
TMS
NH2
F
(10u, 101 MHz, (CD3)2SO)
f1 (ppm)

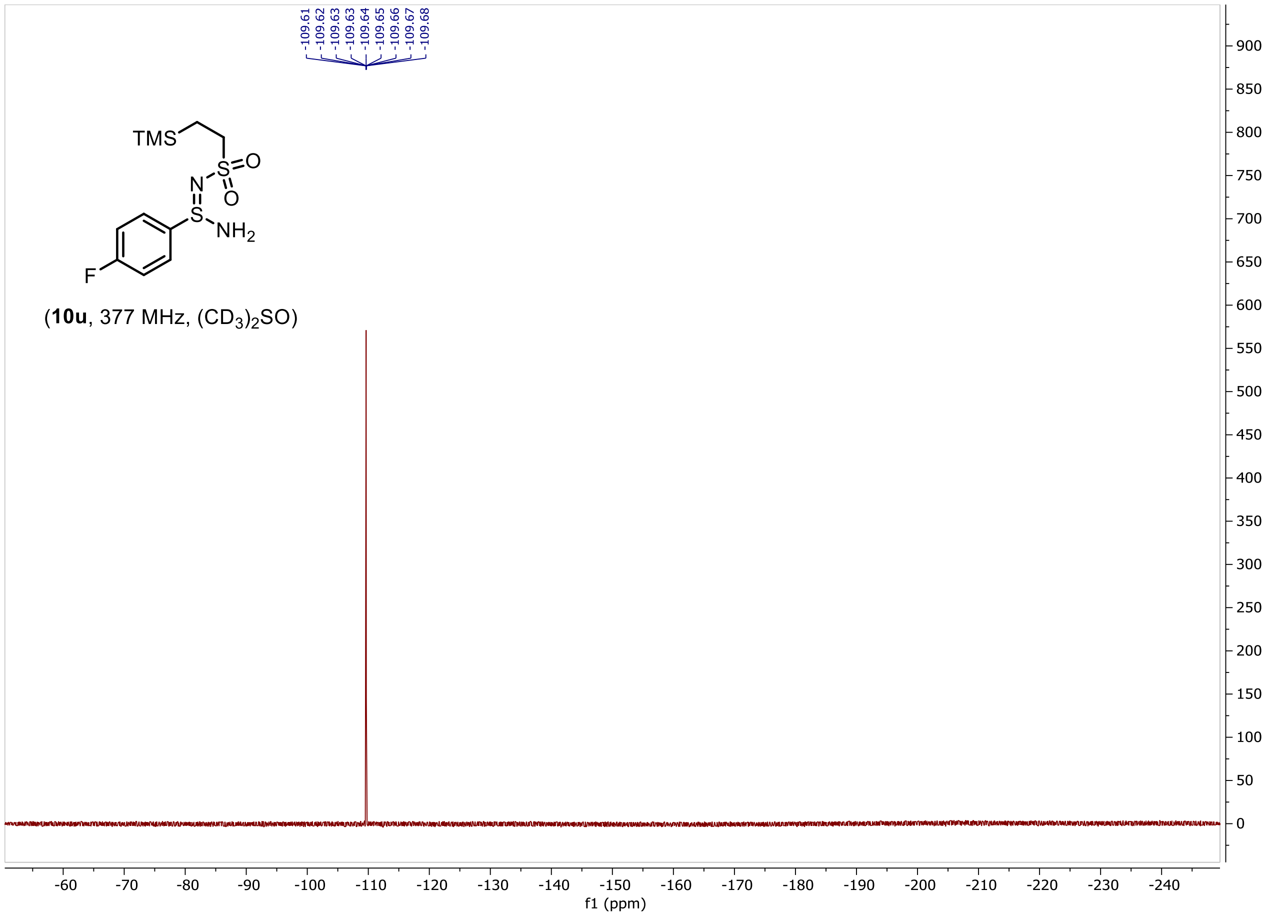
-109.61
-109.62
-109.63
-109.63
-109.64
-109.65
-109.66
-109.67
-109.68
TMS
NH2
F
(10u, 377 MHz, (CD3)2SO)
f1 (ppm)

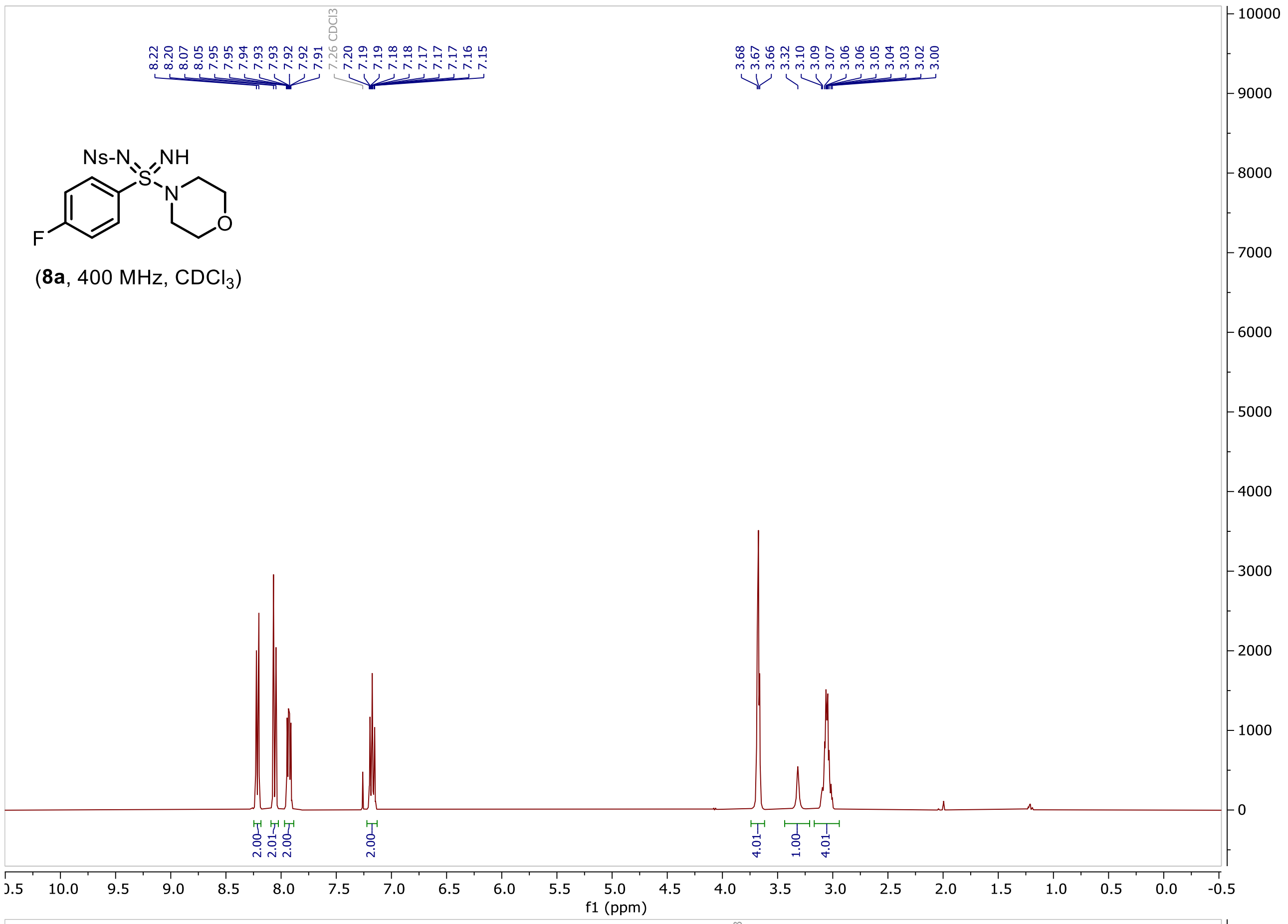

8.22
8.20
8.07
8.05
7.95
7.95
7.94
7.93
7.93
7.92
7.92
7.91
7.26 CDCl3
7.20
7.19
7.19
7.18
7.18
7.17
7.17
7.17
7.16
7.15
3.68
3.67
3.66
3.32
3.10
3.09
3.07
3.06
3.06
3.05
3.04
3.03
3.02
3.00
Ns-N
NH
S
N
O
F
(8a, 400 MHz, CDCl3)
2.00
2.01
2.00
2.00
4.01
1.00
4.01
f1 (ppm)

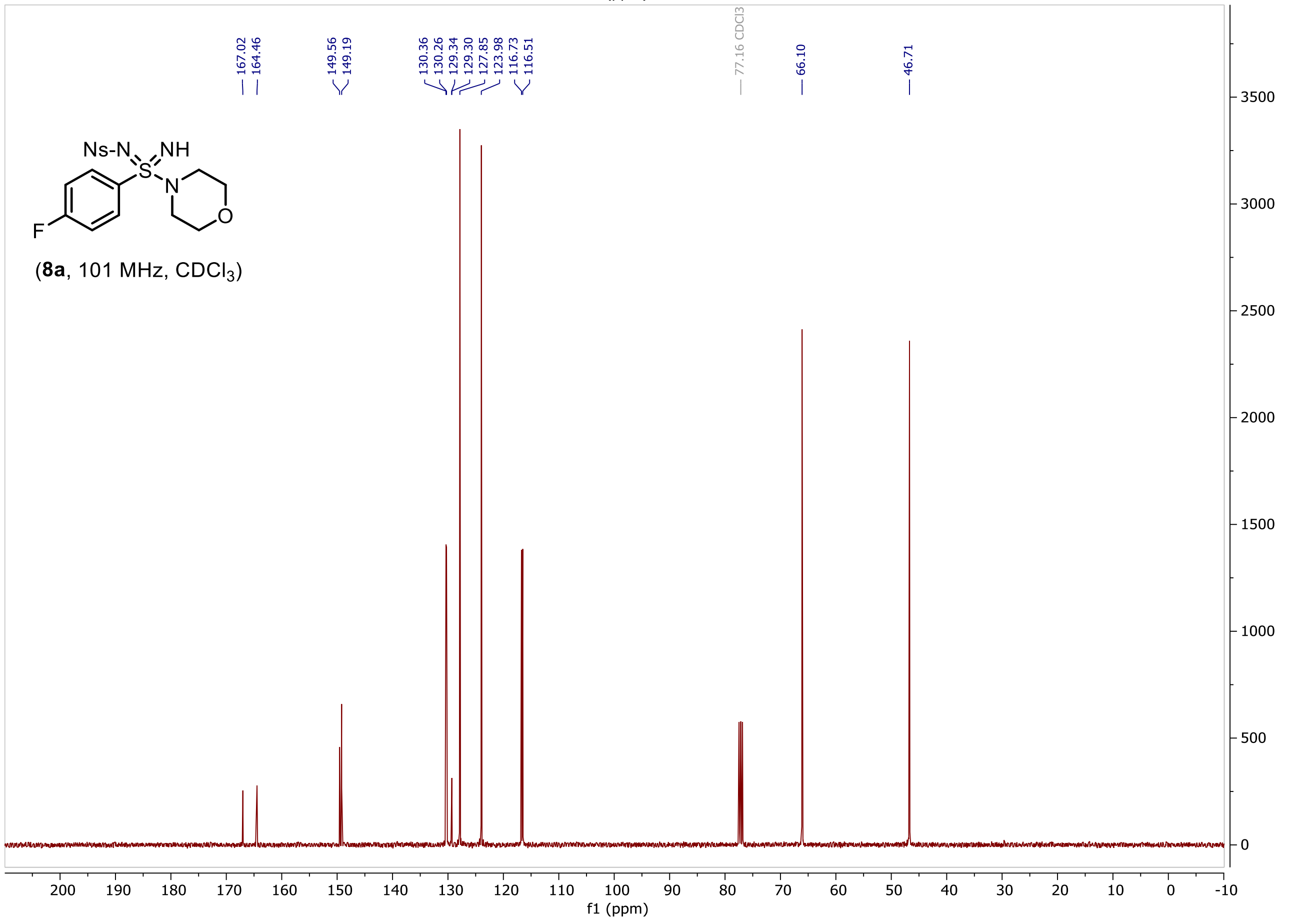

167.02
164.46
149.56
149.19
130.36
130.26
129.34
129.30
127.85
123.98
116.73
116.51
77.16 CDCl3
66.10
46.71
Ns-N
NH
S
N
O
F
(8a, 101 MHz, CDCl3)
f1 (ppm)

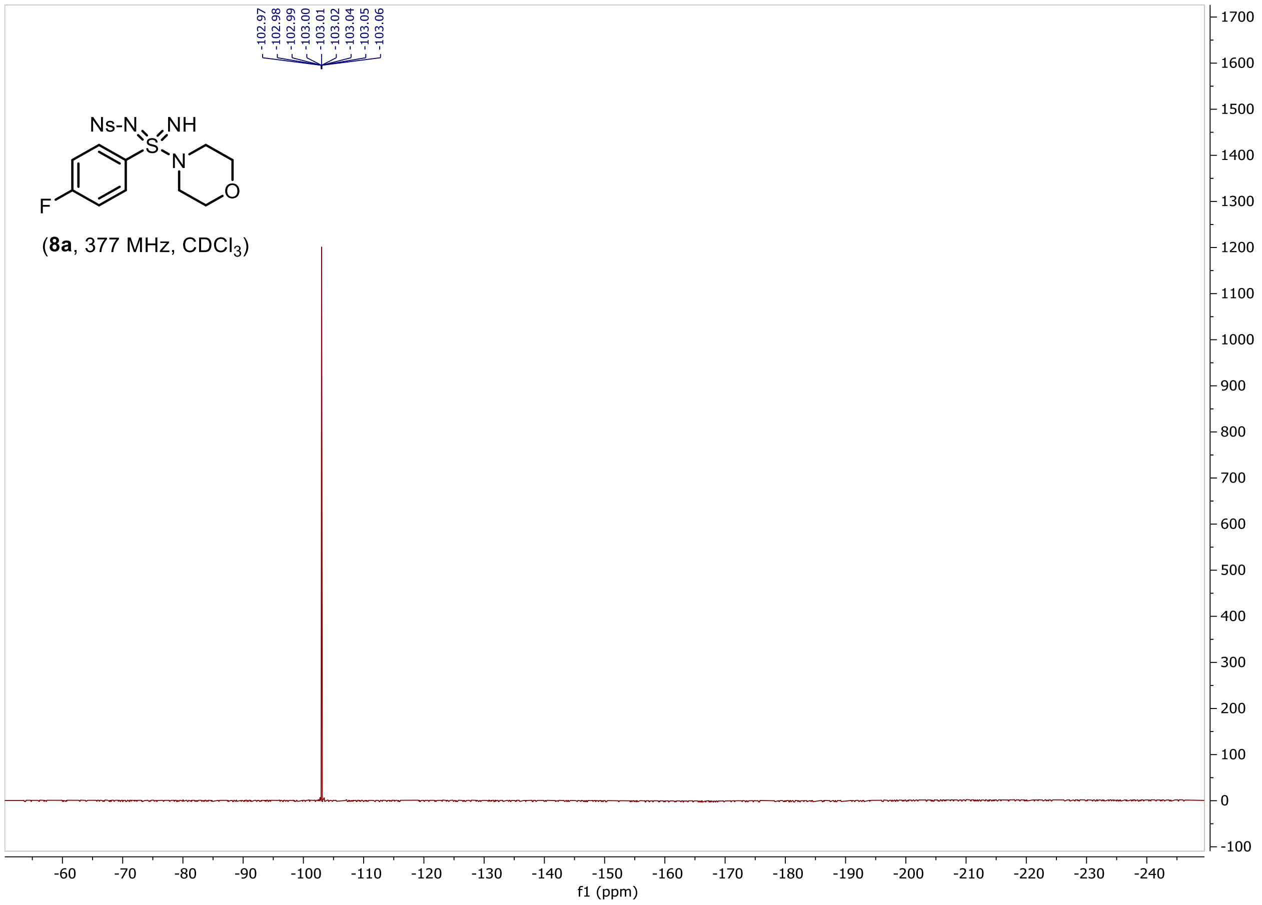

(**8a**, 377 MHz, $CDCl_3$)

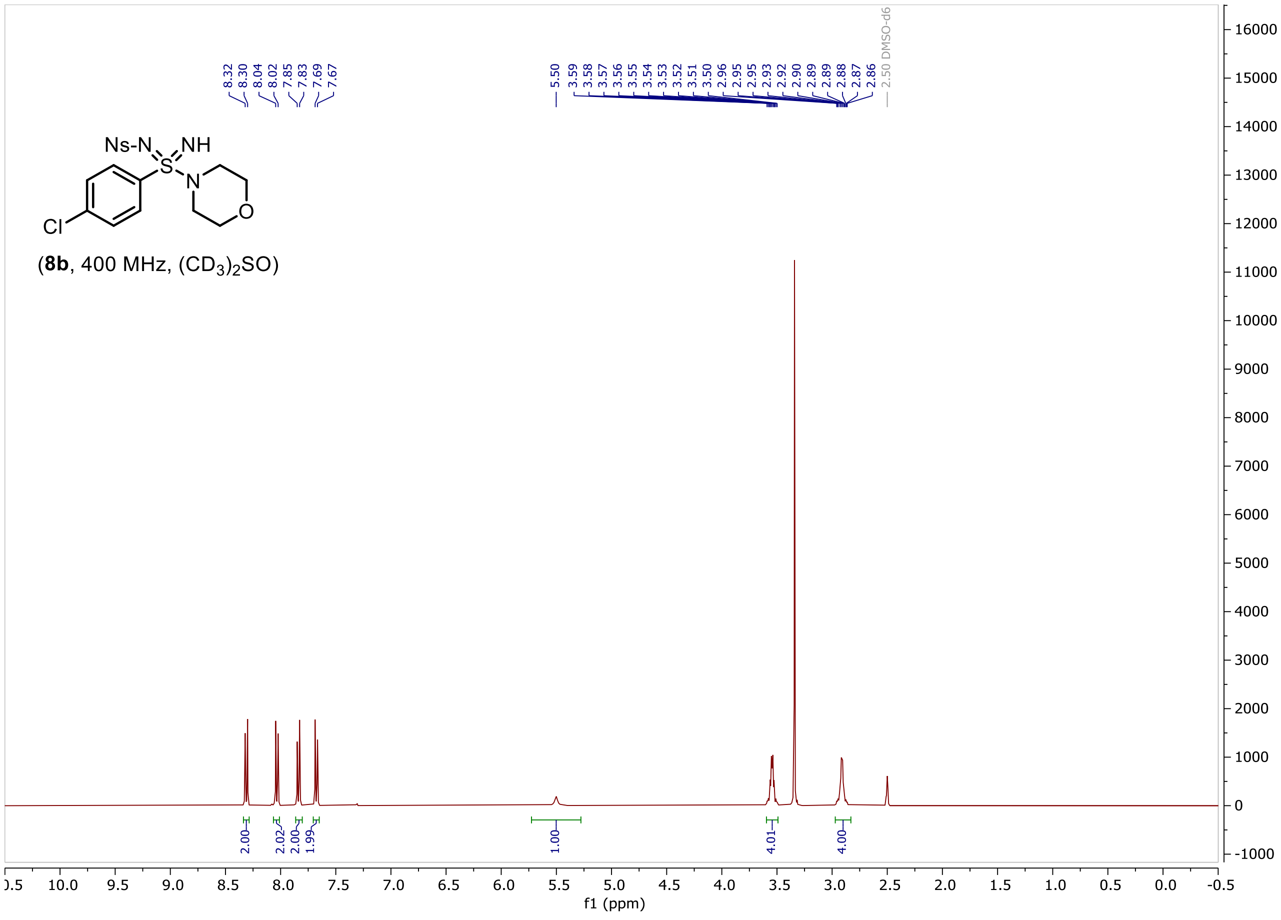

(**8b**, 400 MHz, $(CD_3)_2SO$)

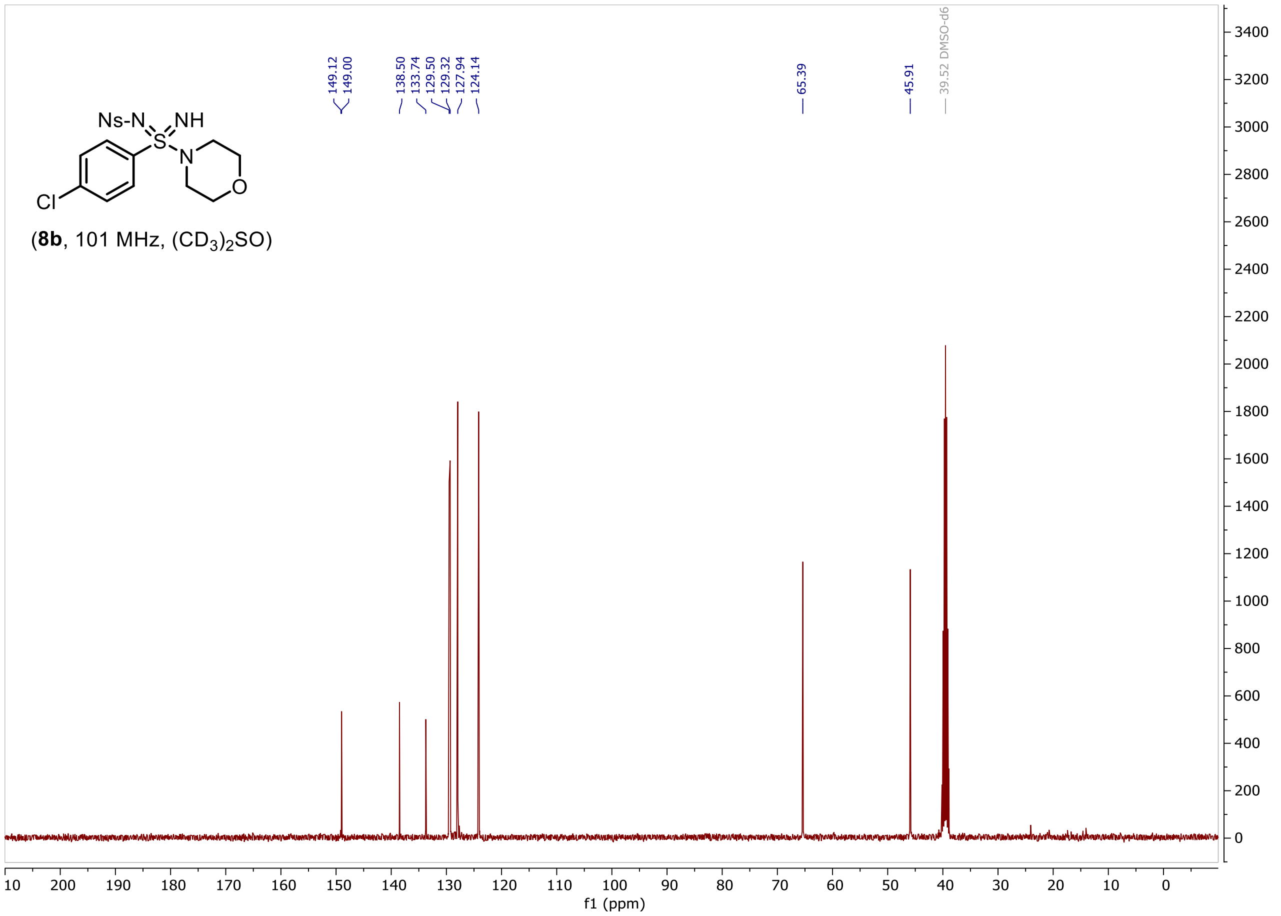

(**8b**, 101 MHz, $(CD_3)_2SO$)

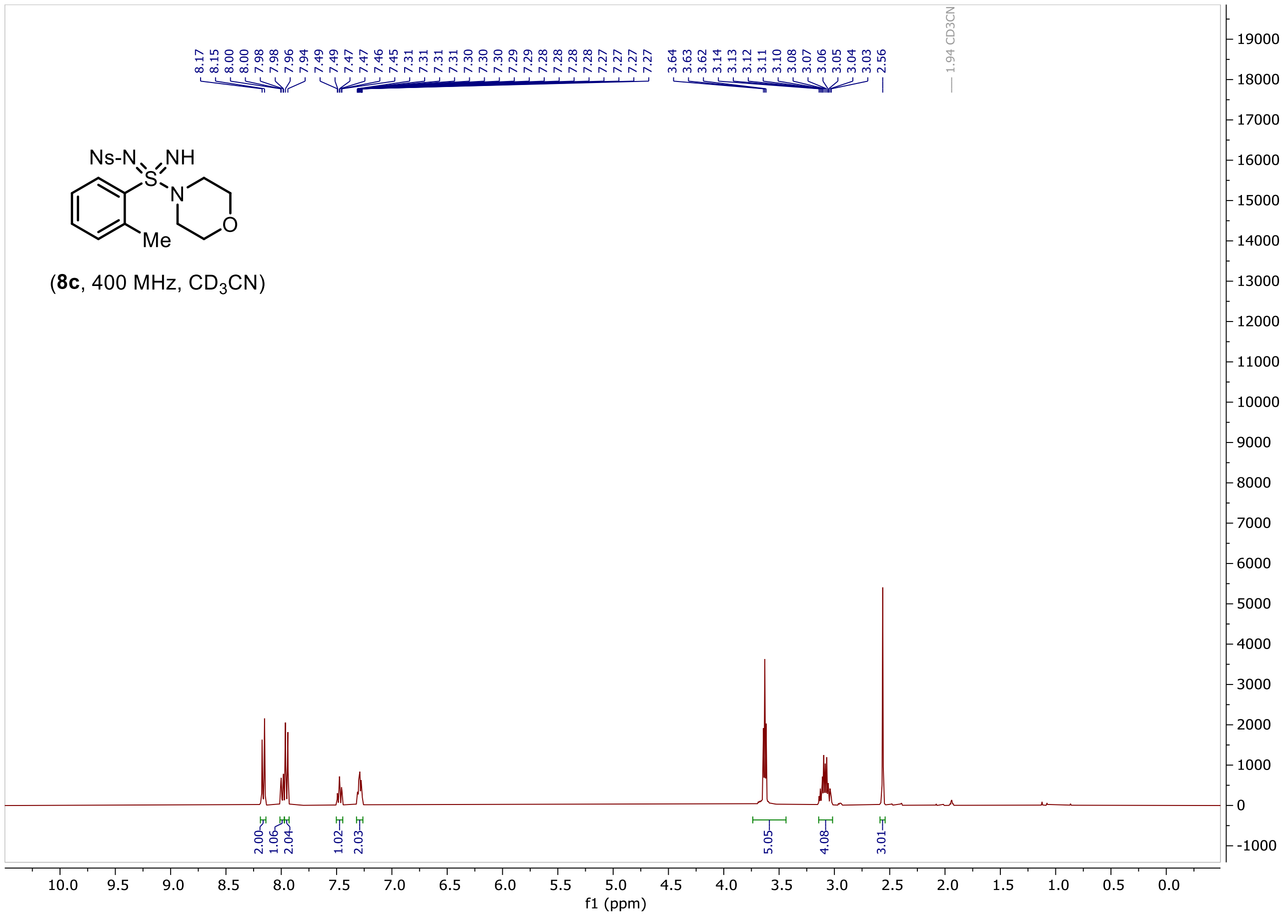

(**8c**, 400 MHz, CD_3CN)

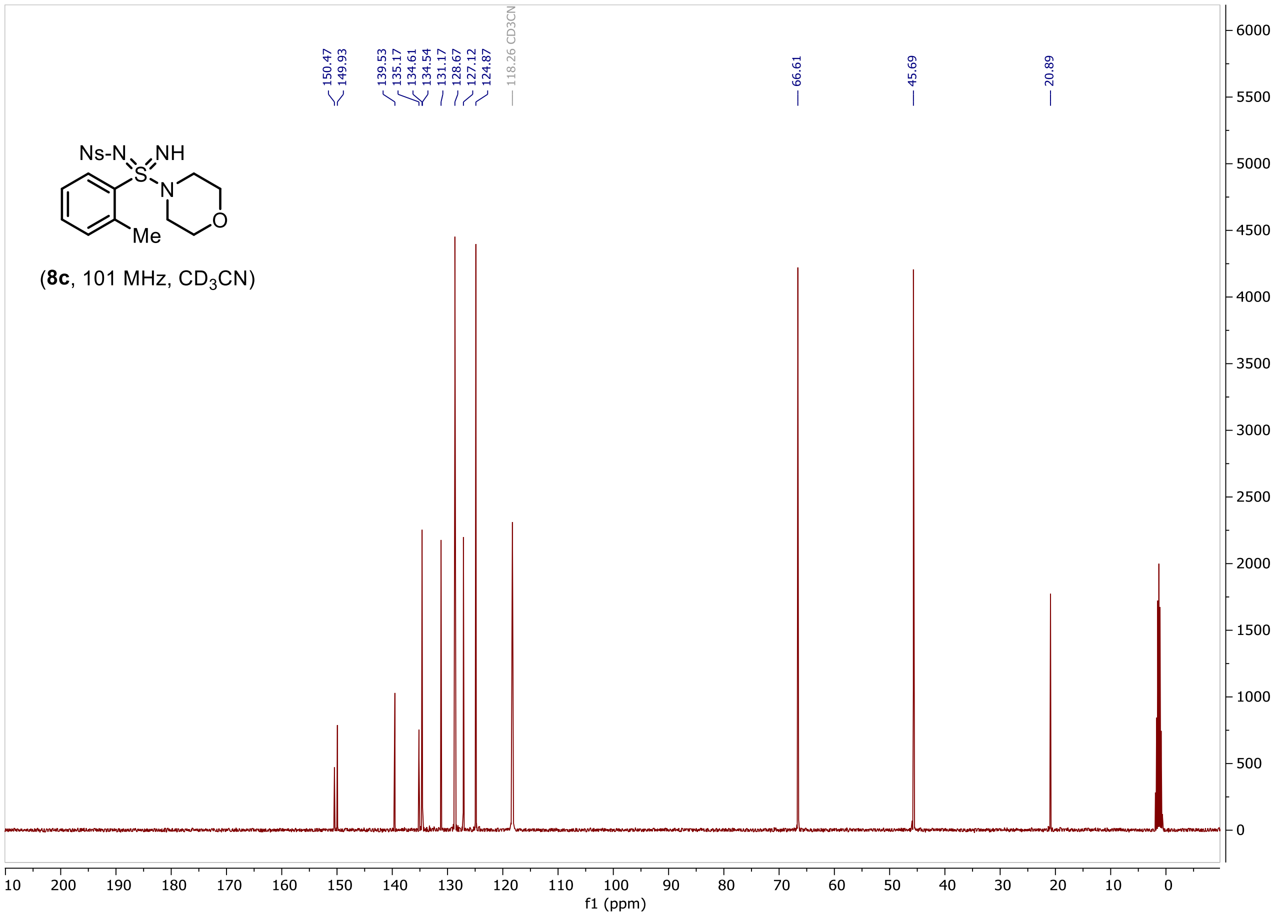
150.47
149.93
139.53
135.17
134.61
134.54
131.17
128.67
127.12
124.87
118.26 CD3CN
66.61
45.69
20.89
Ns-N
NH
S
N
O
Me
(8c, 101 MHz, CD3CN)
f1 (ppm)

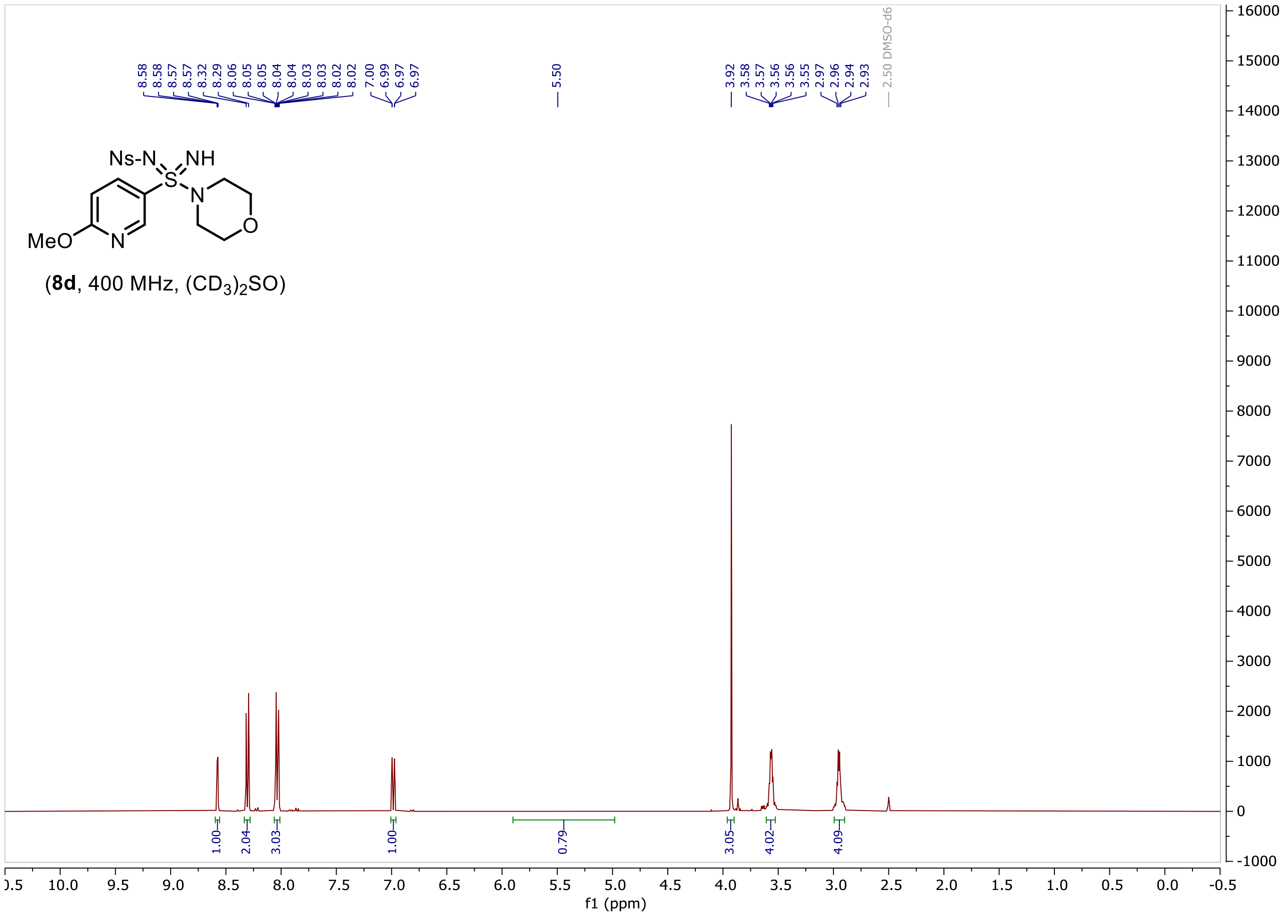
8.58
8.58
8.57
8.57
8.32
8.29
8.06
8.05
8.05
8.04
8.04
8.03
8.02
8.02
7.00
6.99
6.97
6.97
5.50
3.92
3.58
3.57
3.56
3.55
2.97
2.96
2.94
2.93
2.50 DMSO-d6
Ns-N
NH
S
N
O
MeO
N
(8d, 400 MHz, (CD3)2SO)
1.00
2.04
3.03
1.00
0.79
3.05
4.02
4.09
f1 (ppm)

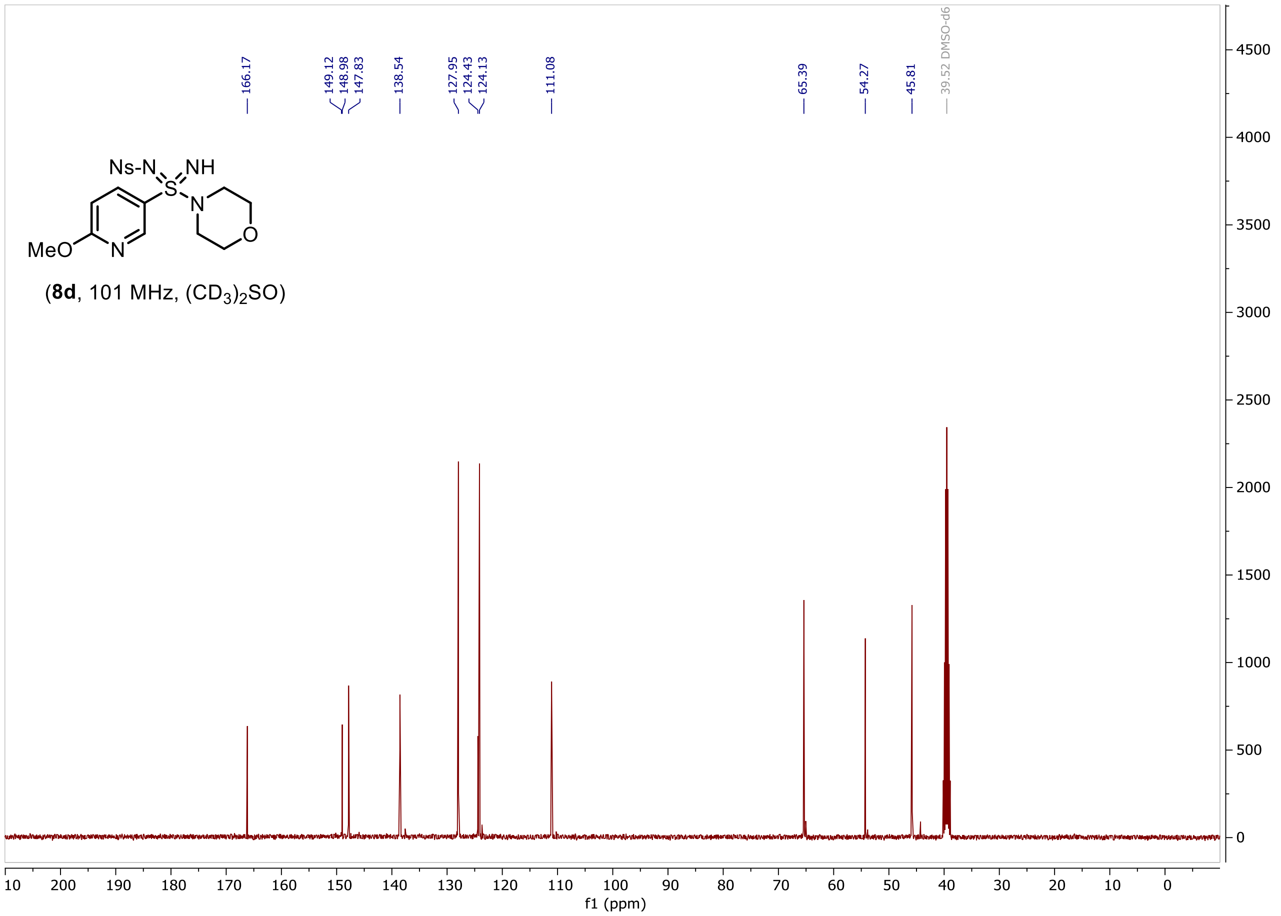
166.17
149.12
148.98
147.83
138.54
127.95
124.43
124.13
111.08
65.39
54.27
45.81
39.52 DMSO-d6
Ns-N
NH
S
N
O
MeO
N
(8d, 101 MHz, (CD3)2SO)
f1 (ppm)

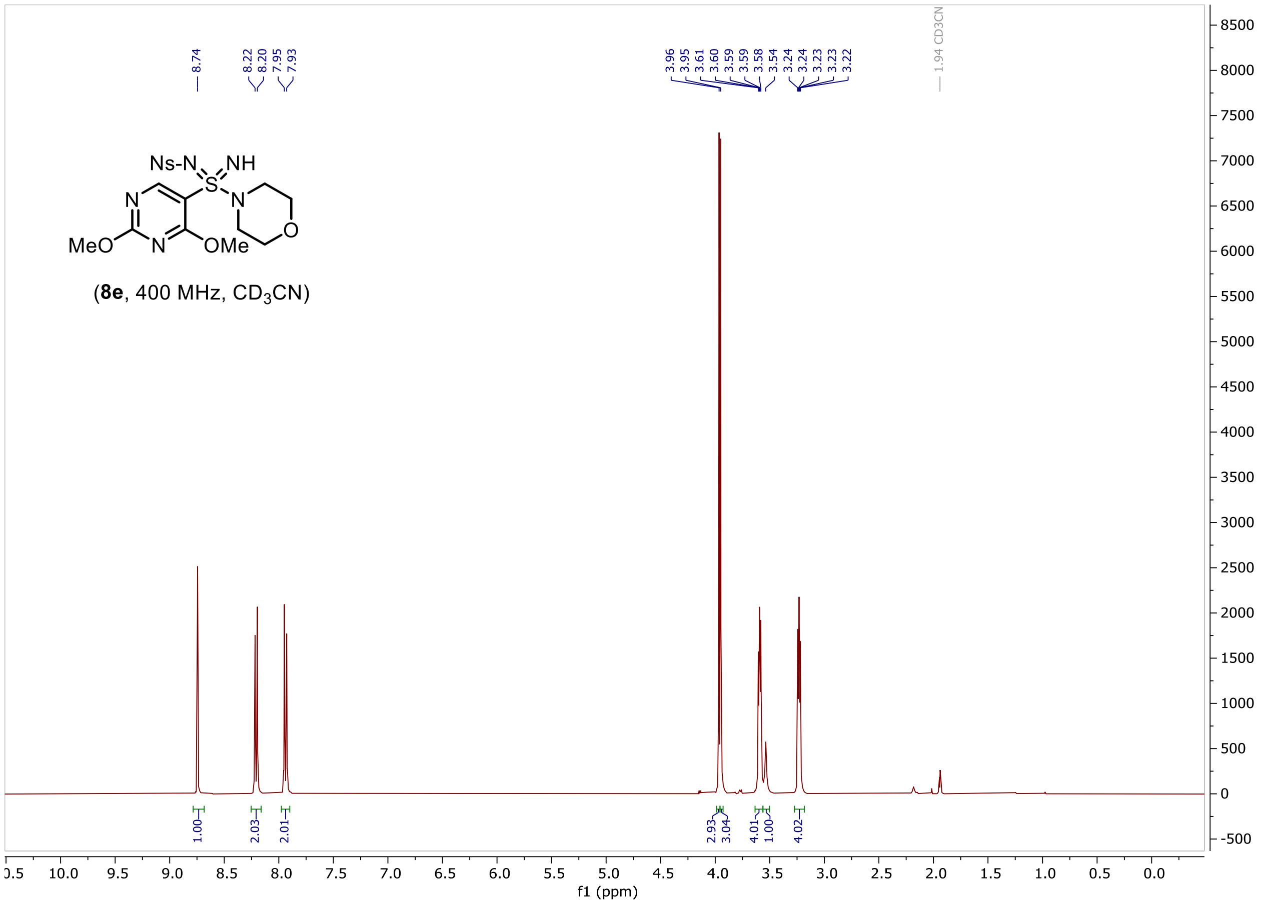
8.74
8.22
8.20
7.95
7.93
3.96
3.95
3.61
3.60
3.59
3.59
3.58
3.54
3.24
3.24
3.23
3.23
3.22
1.94 CD3CN
Ns-N
NH
S
N
N
O
MeO
N
OMe
(8e, 400 MHz, CD3CN)
1.00
2.03
2.01
2.93
3.04
4.01
1.00
4.02
f1 (ppm)

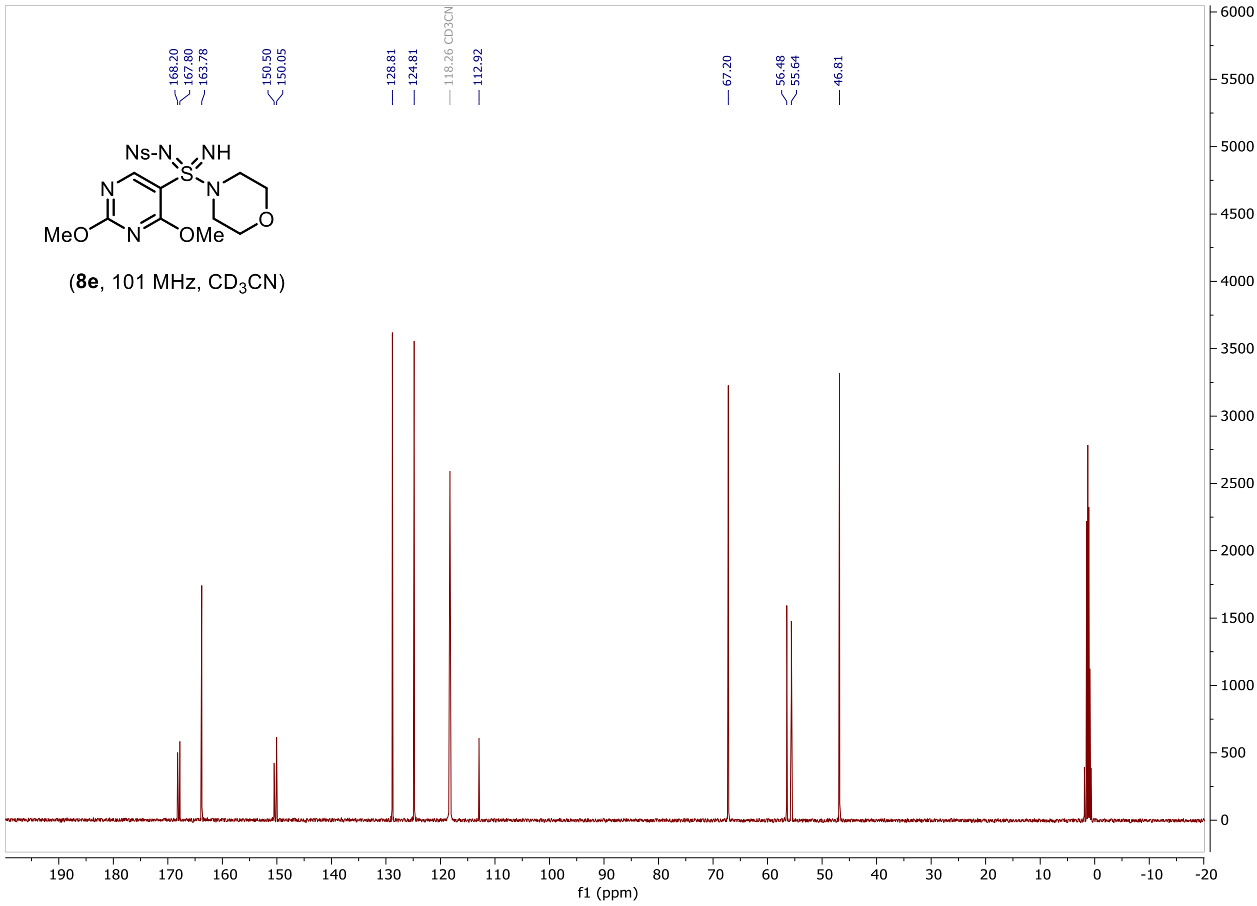

168.20
167.80
163.78
150.50
150.05
128.81
124.81
118.26 CD3CN
112.92
67.20
56.48
55.64
46.81
Ns-N
NH
S
N
O
N
N
MeO
OMe
(8e, 101 MHz, CD3CN)
f1 (ppm)

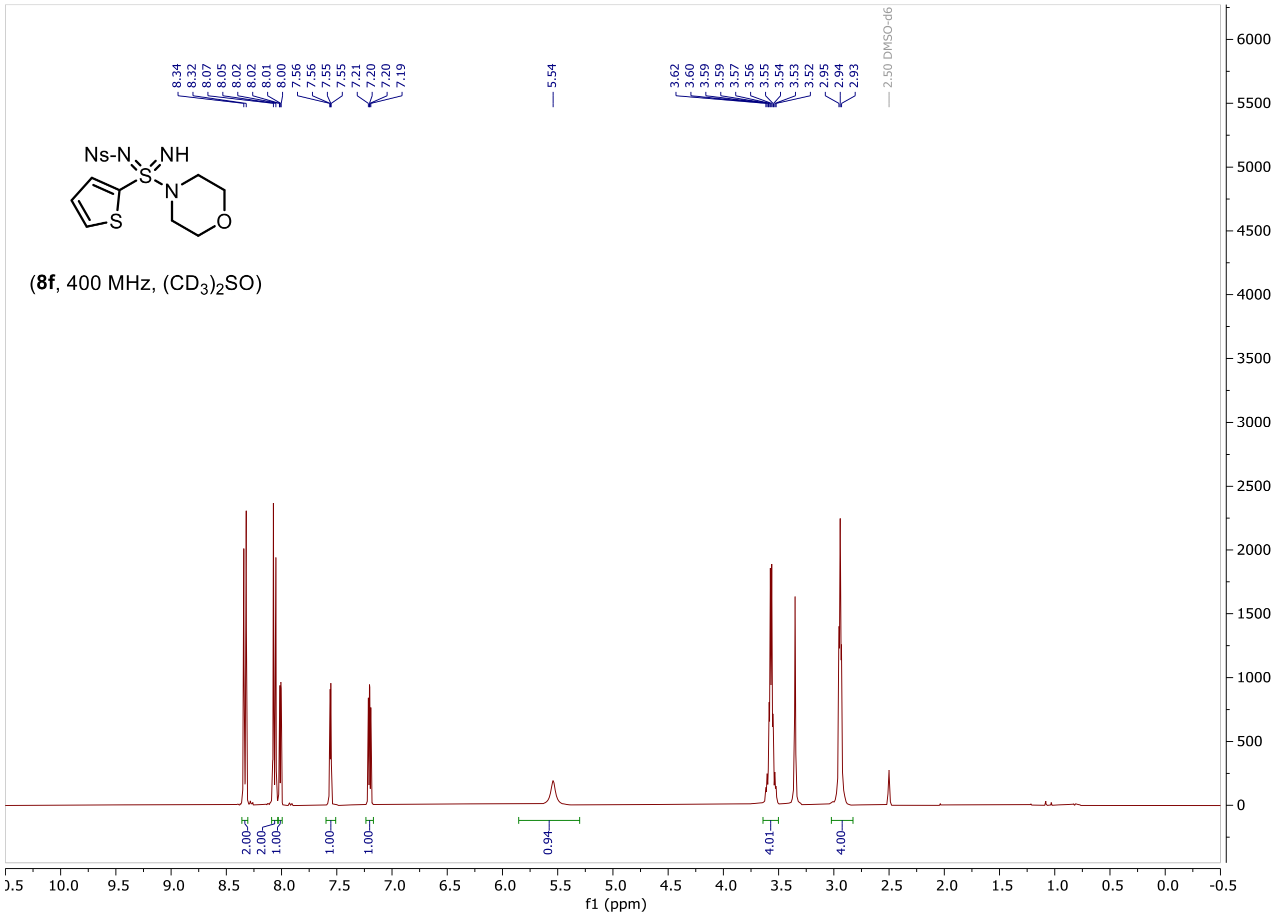

8.34
8.32
8.07
8.05
8.02
8.02
8.01
8.00
7.56
7.56
7.55
7.55
7.21
7.20
7.20
7.19
5.54
3.62
3.60
3.59
3.59
3.57
3.56
3.55
3.54
3.53
3.52
2.95
2.94
2.93
2.50 DMSO-d6
Ns-N
NH
S
N
S
O
(8f, 400 MHz, (CD3)2SO)
2.00
2.00
1.00
1.00
1.00
0.94
4.01
4.00
f1 (ppm)

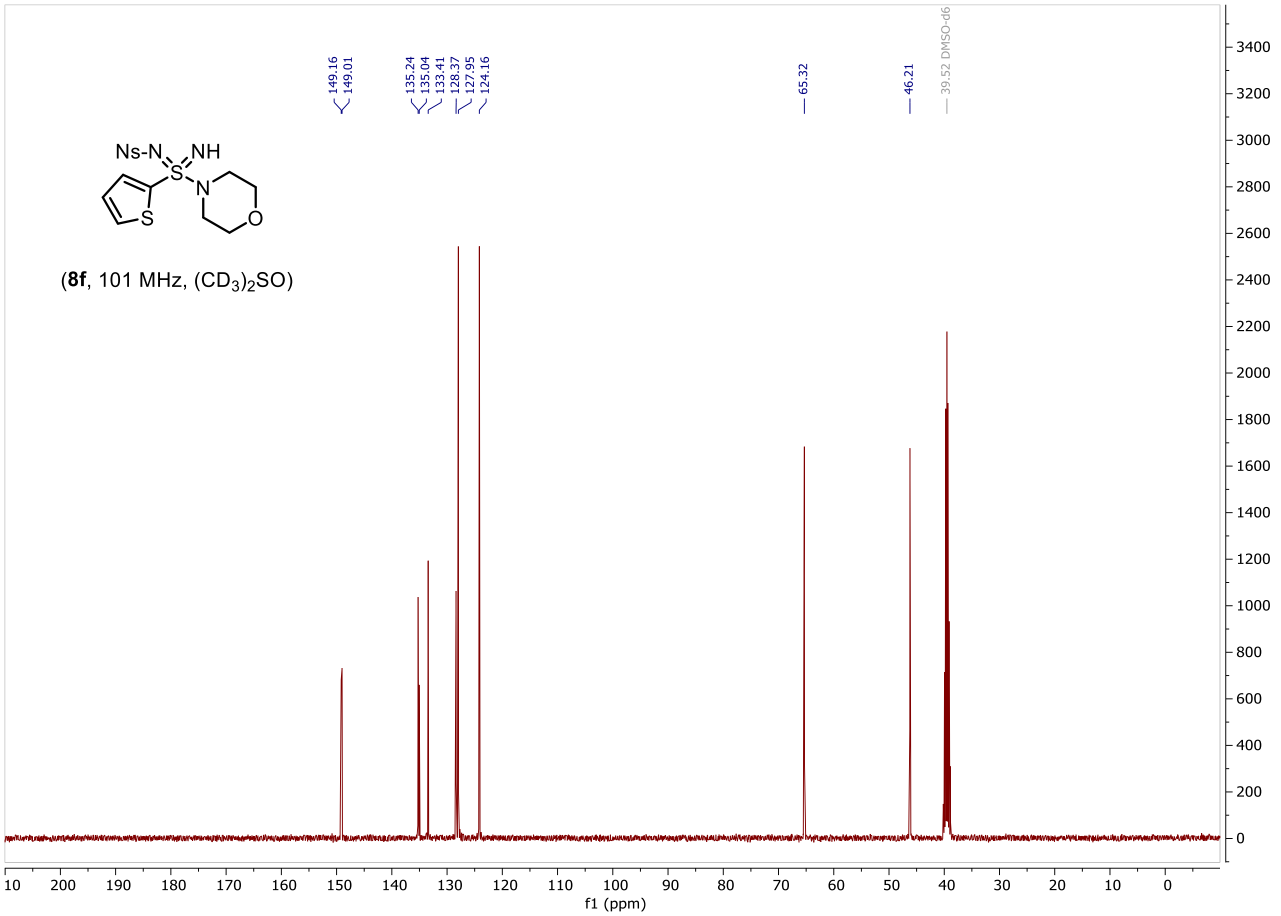
149.16
149.01
135.24
135.04
133.41
128.37
127.95
124.16
65.32
46.21
39.52 DMSO-d6
Ns-N
NH
S
N
O
S
(8f, 101 MHz, (CD3)2SO)
f1 (ppm)

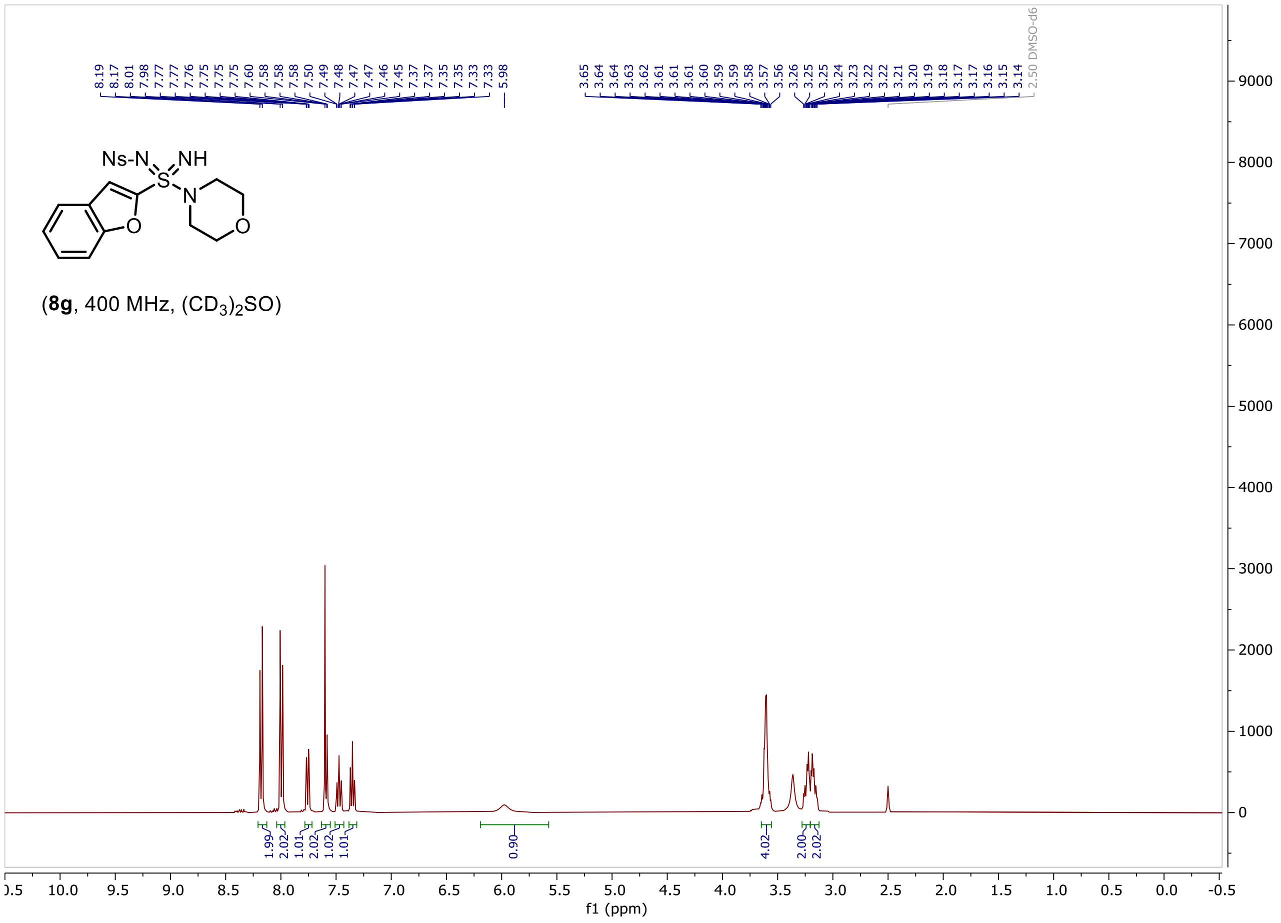
8.19 8.17 8.01 7.98 7.77 7.77 7.76 7.75 7.75 7.75 7.60 7.58 7.58 7.58 7.50 7.49 7.48 7.47 7.47 7.46 7.45 7.37 7.37 7.35 7.35 7.33 7.33 5.98
3.65 3.64 3.64 3.63 3.62 3.61 3.61 3.61 3.60 3.59 3.59 3.58 3.57 3.56 3.26 3.25 3.25 3.24 3.23 3.22 3.22 3.21 3.20 3.19 3.18 3.17 3.16 3.15 3.14
2.50 DMSO-d6
Ns-N
NH
S
N
O
O
(8g, 400 MHz, (CD3)2SO)
1.99 2.02 1.01 2.02 1.02 1.01 0.90 4.02 2.00 2.02
f1 (ppm)

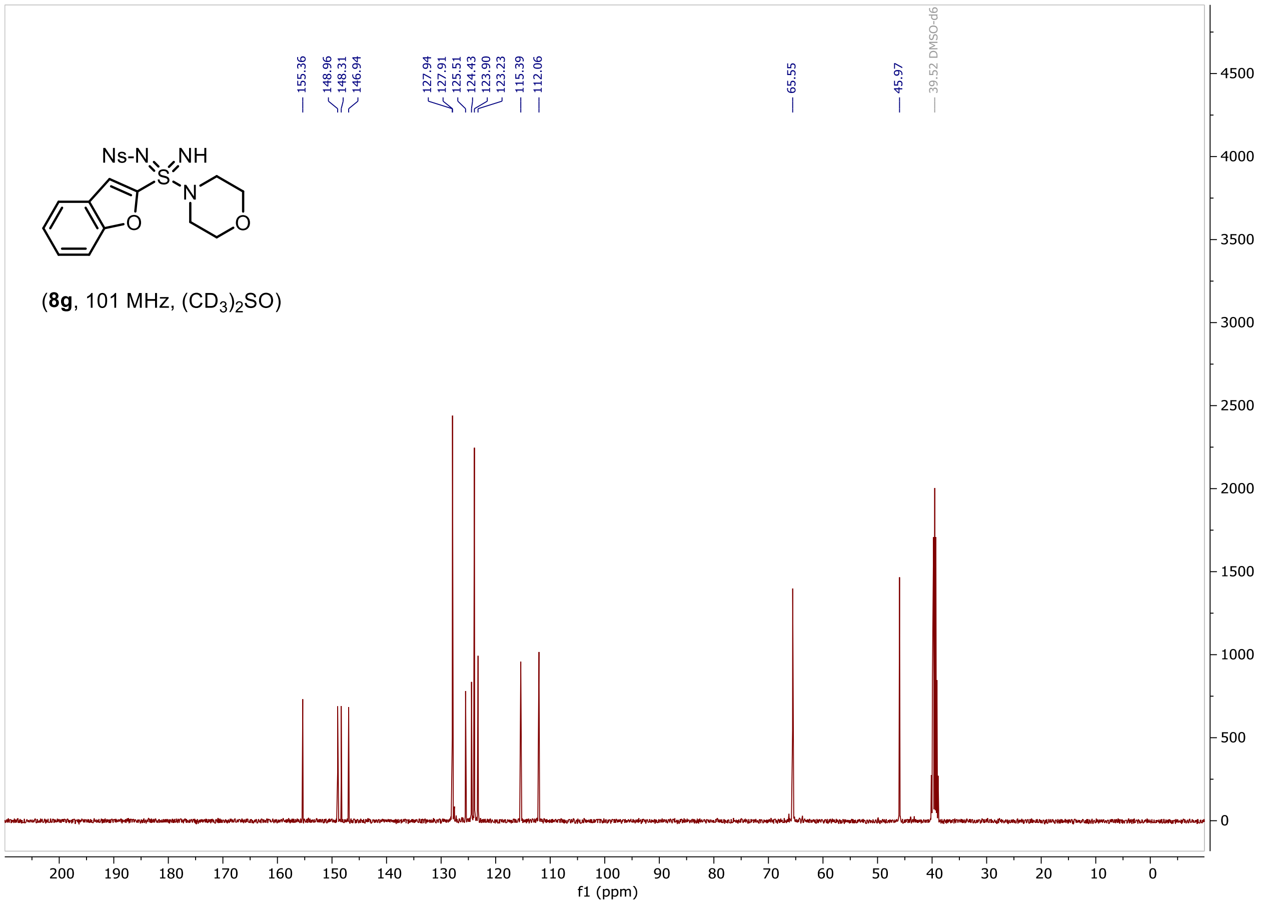
155.36
148.96
148.31
146.94
127.94
127.91
125.51
124.43
123.90
123.23
115.39
112.06
65.55
45.97
39.52 DMSO-d6
Ns-N
NH
S
N
O
O
(8g, 101 MHz, (CD3)2SO)
f1 (ppm)

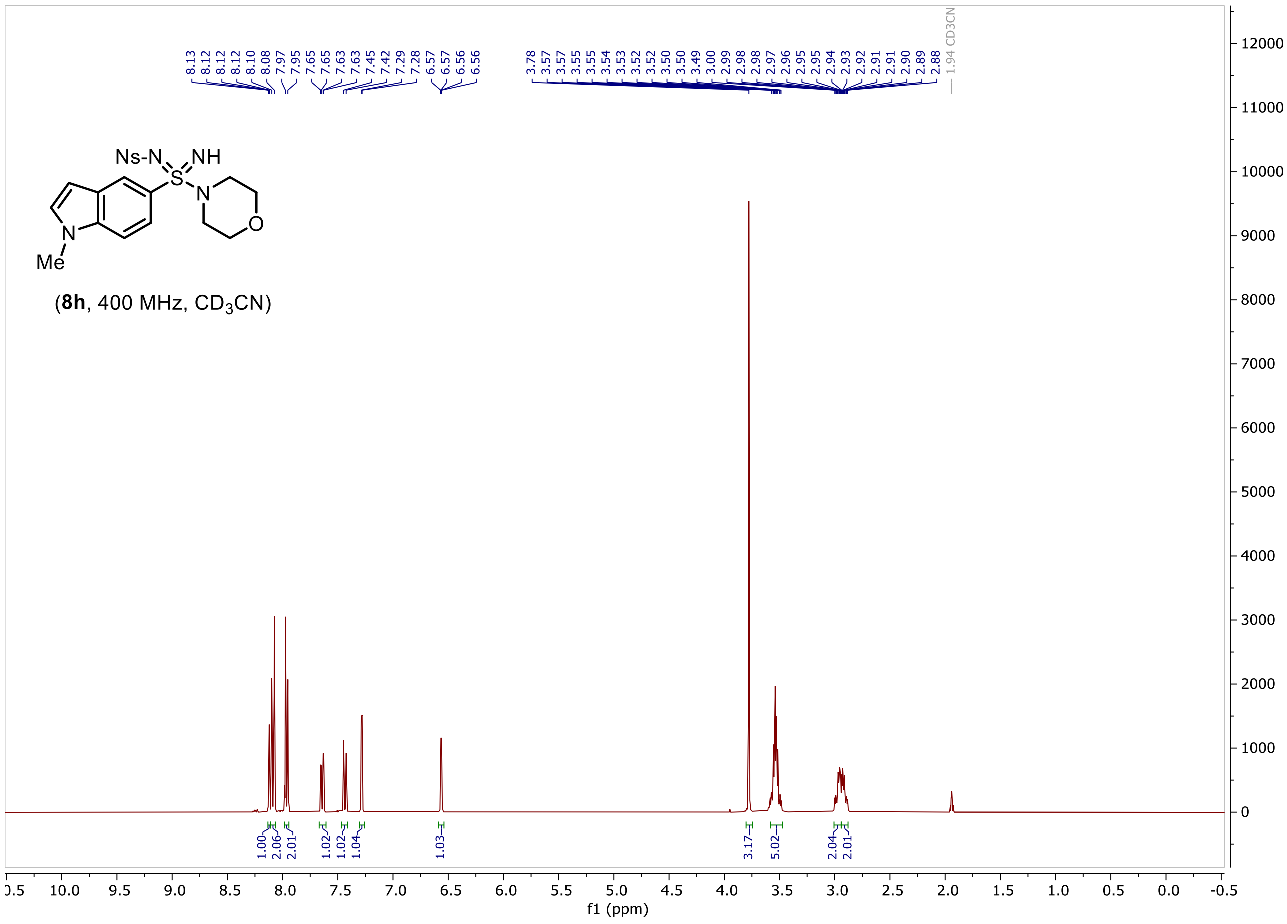
1.94 CD3CN
Ns-N
NH
S
N
O
N
Me
(8h, 400 MHz, CD3CN)
f1 (ppm)

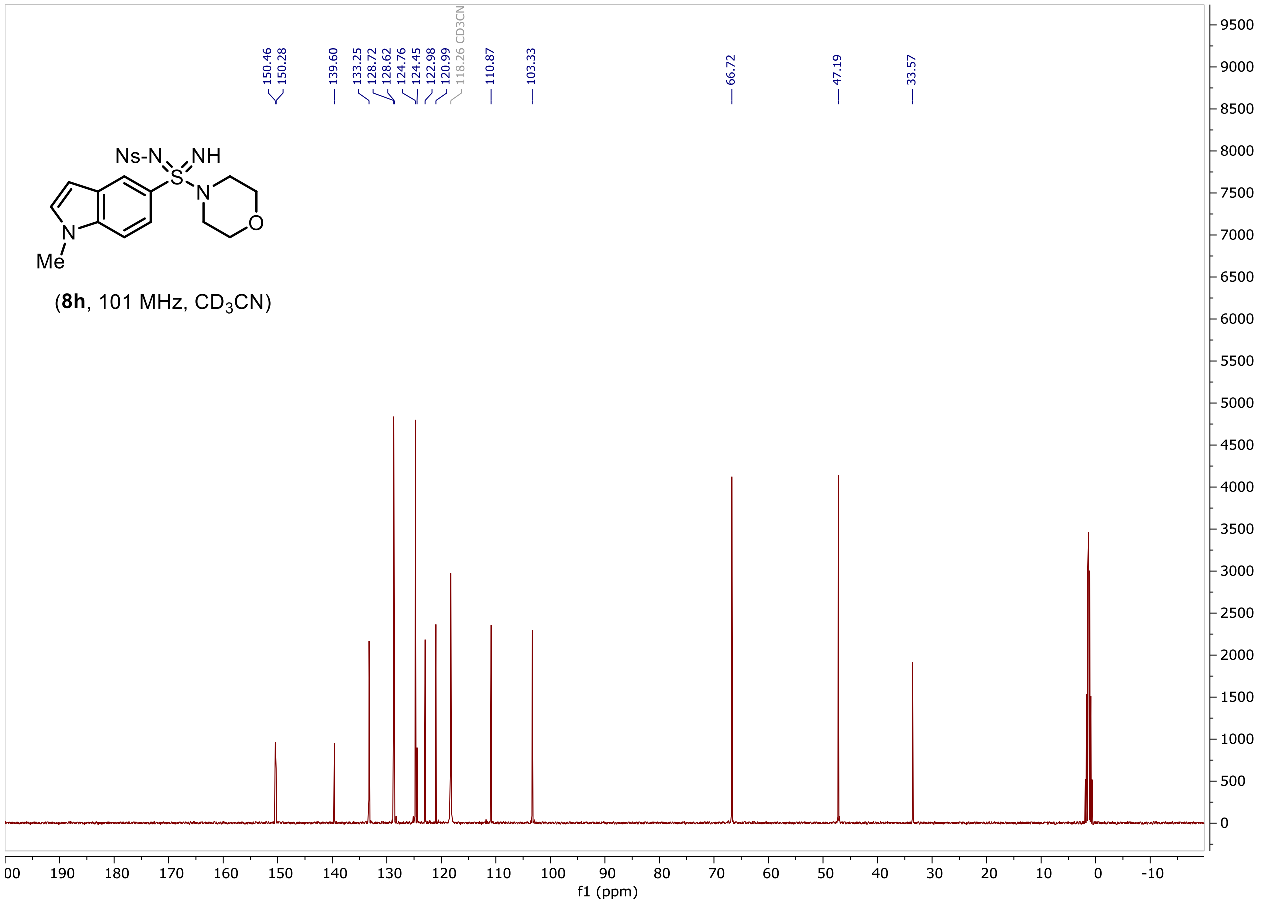
150.46
150.28
139.60
133.25
128.72
128.62
124.76
124.45
122.98
120.99
118.26 CD3CN
110.87
103.33
66.72
47.19
33.57
Ns-N
NH
S
N
O
N
Me
(8h, 101 MHz, CD3CN)
f1 (ppm)

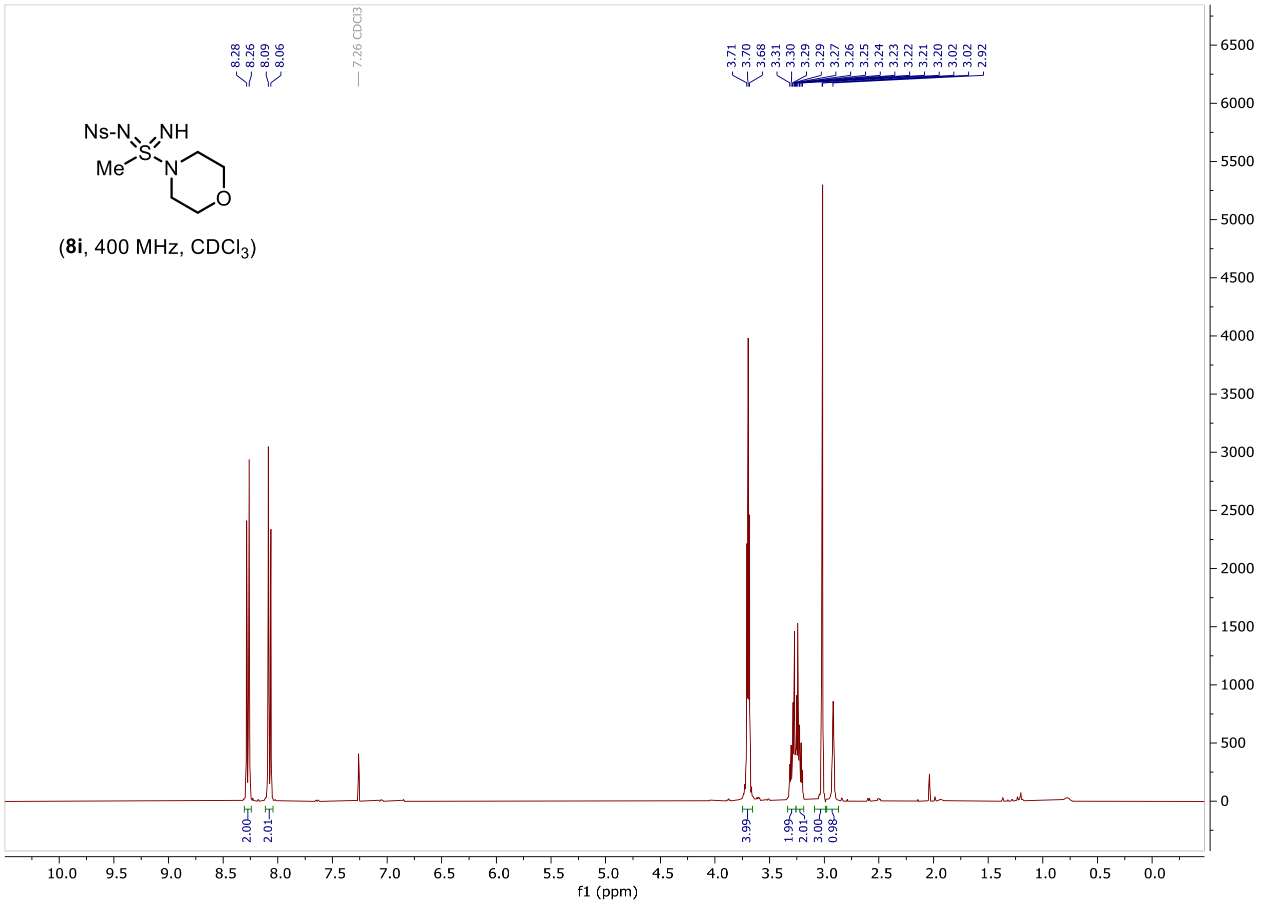
8.28
8.26
8.09
8.06
7.26 CDCl3
3.71
3.70
3.68
3.31
3.30
3.29
3.29
3.27
3.26
3.25
3.24
3.23
3.22
3.21
3.20
3.02
2.92
Ns-N
NH
S
Me
N
O
(8i, 400 MHz, CDCl3)
2.00
2.01
3.99
1.99
2.01
3.00
0.98
f1 (ppm)

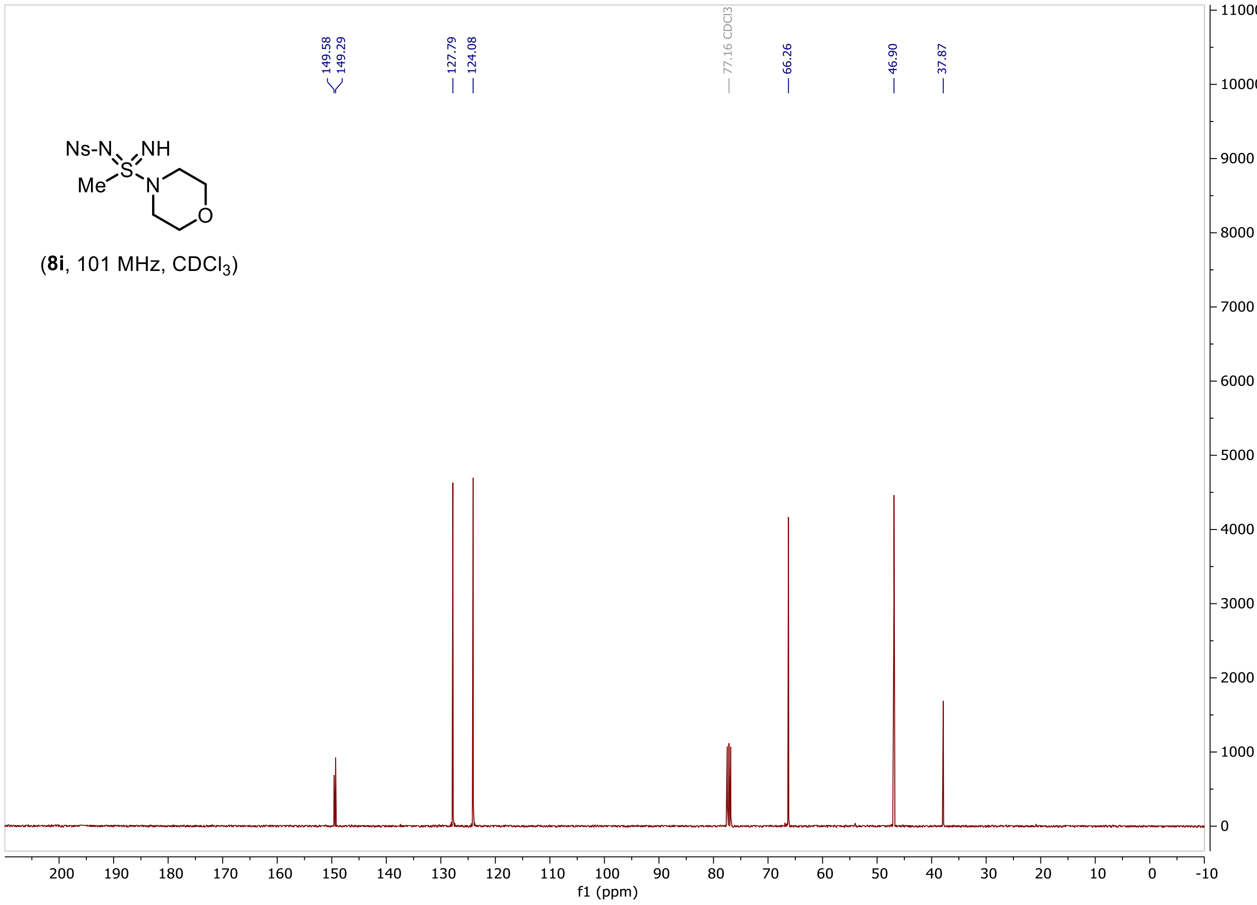

(**8i**, 101 MHz, $CDCl_3$)

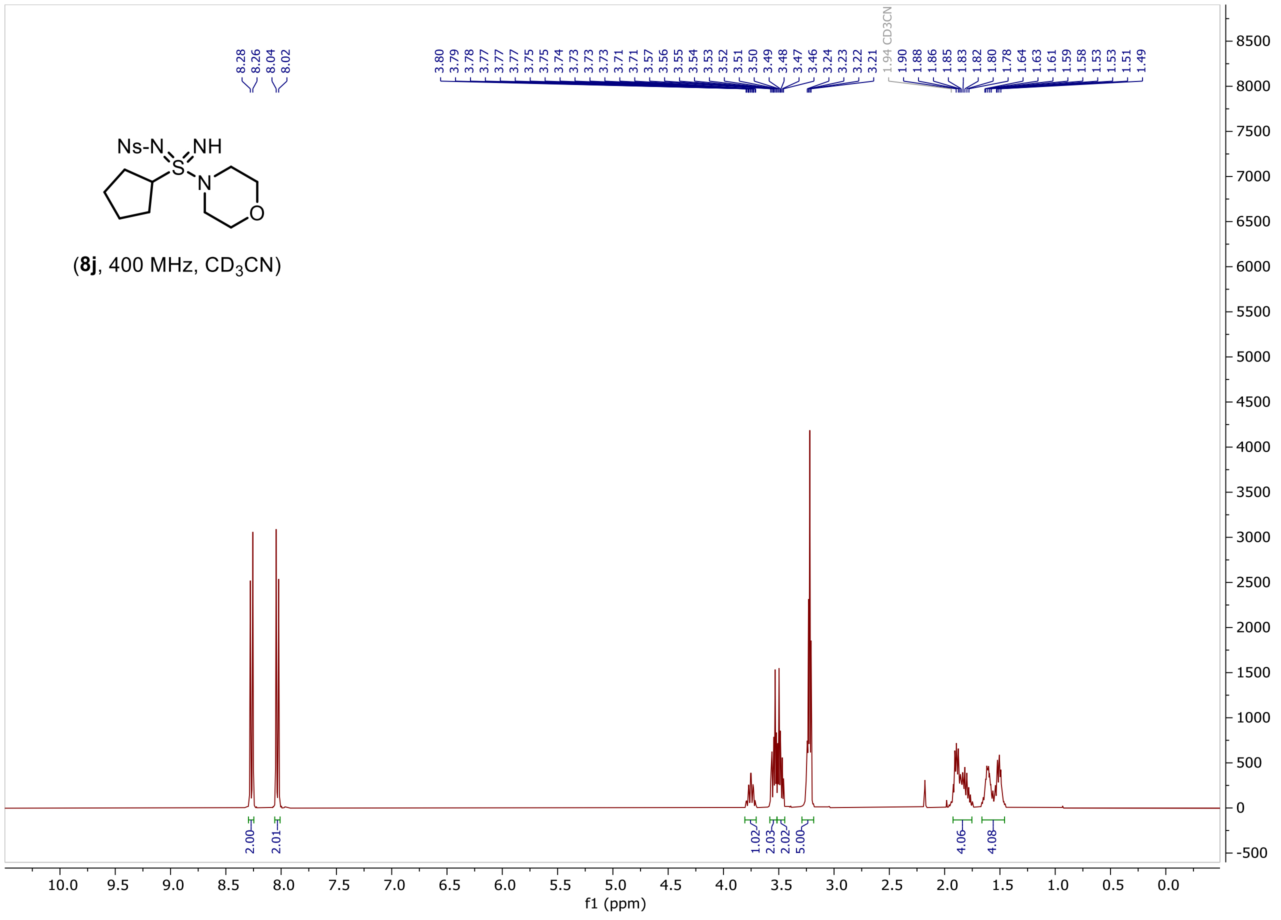

(**8j**, 400 MHz, CD_3CN)

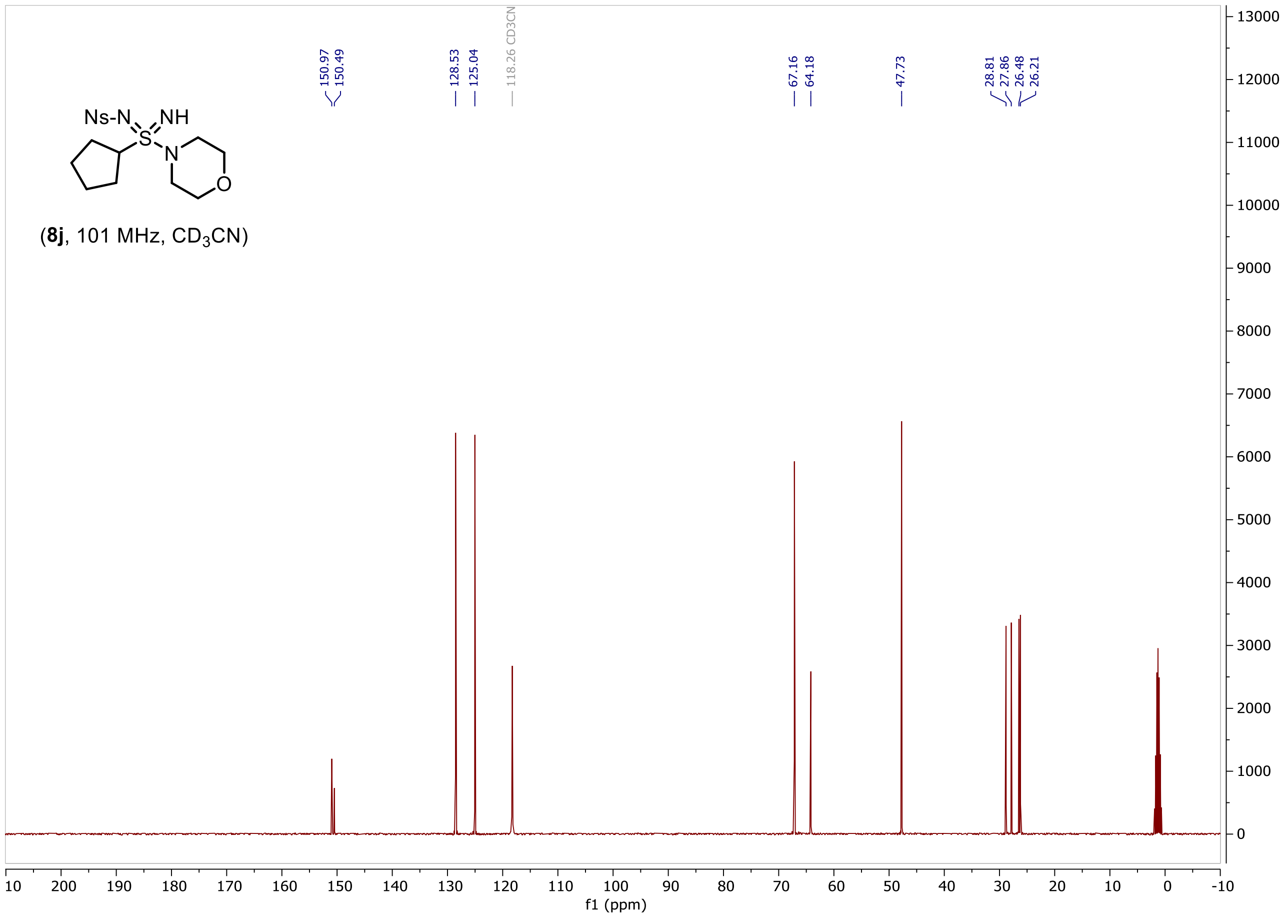
Ns-N
NH
S
N
O
(8j, 101 MHz, CD3CN)
150.97
150.49
128.53
125.04
118.26 CD3CN
67.16
64.18
47.73
28.81
27.86
26.48
26.21
f1 (ppm)

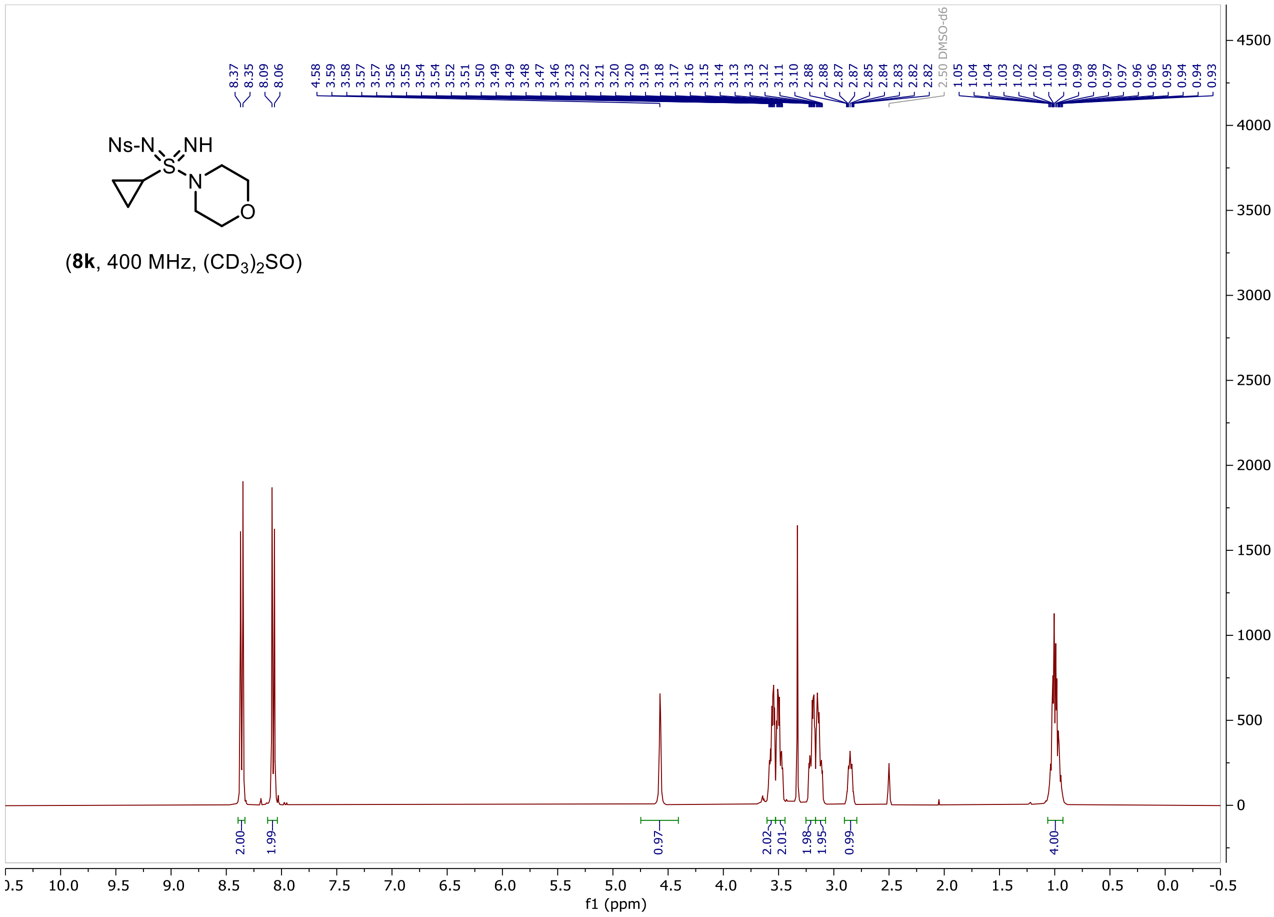
Ns-N
NH
S
N
O
(8k, 400 MHz, (CD3)2SO)
8.37
8.35
8.09
8.06
4.58
2.50 DMSO-d6
2.00
1.99
0.97
2.02
2.01
1.98
1.95
0.99
4.00
f1 (ppm)

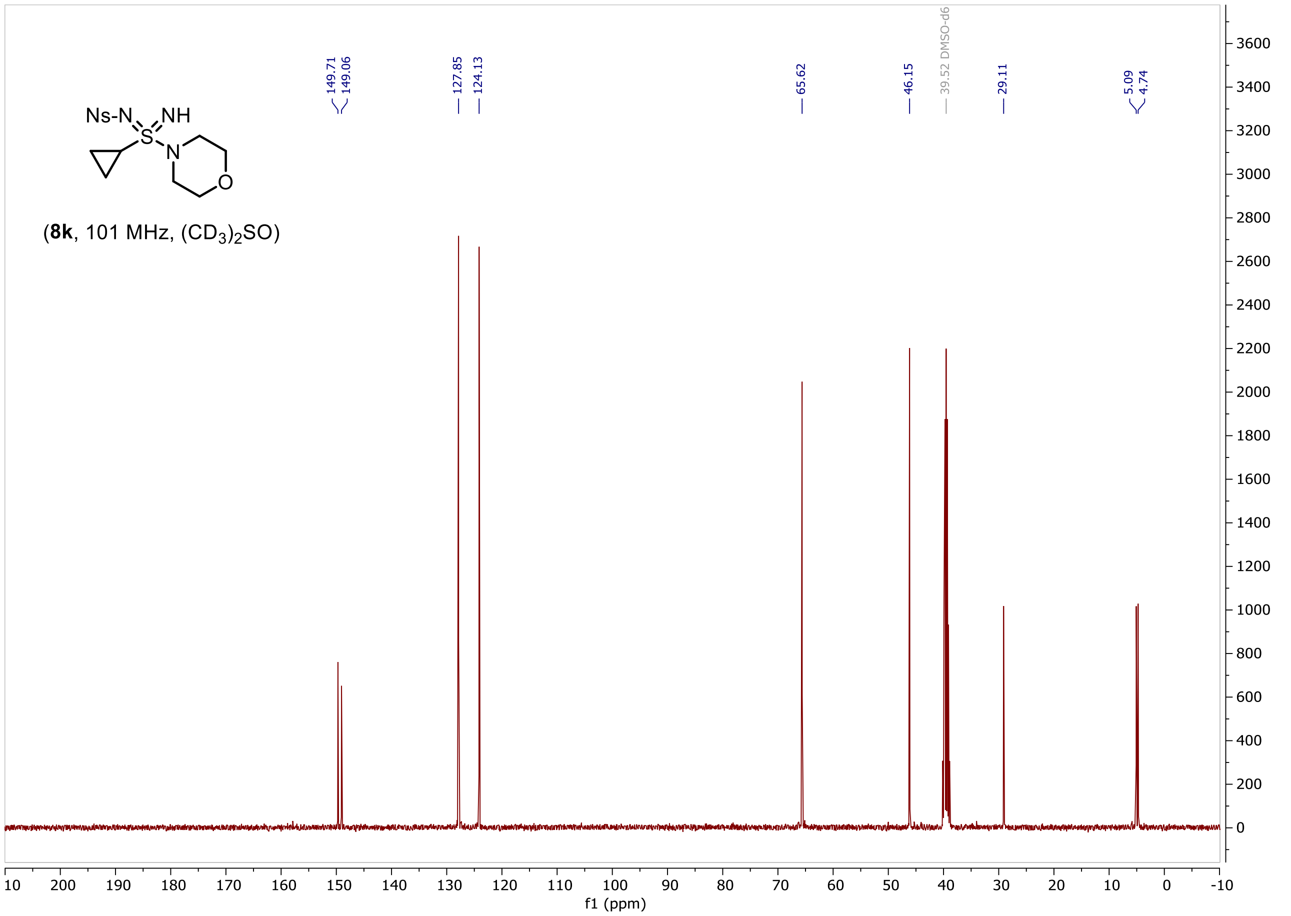
(8k, 101 MHz, (CD3)2SO)
149.71
149.06
127.85
124.13
65.62
46.15
39.52 DMSO-d6
29.11
5.09
4.74
f1 (ppm)

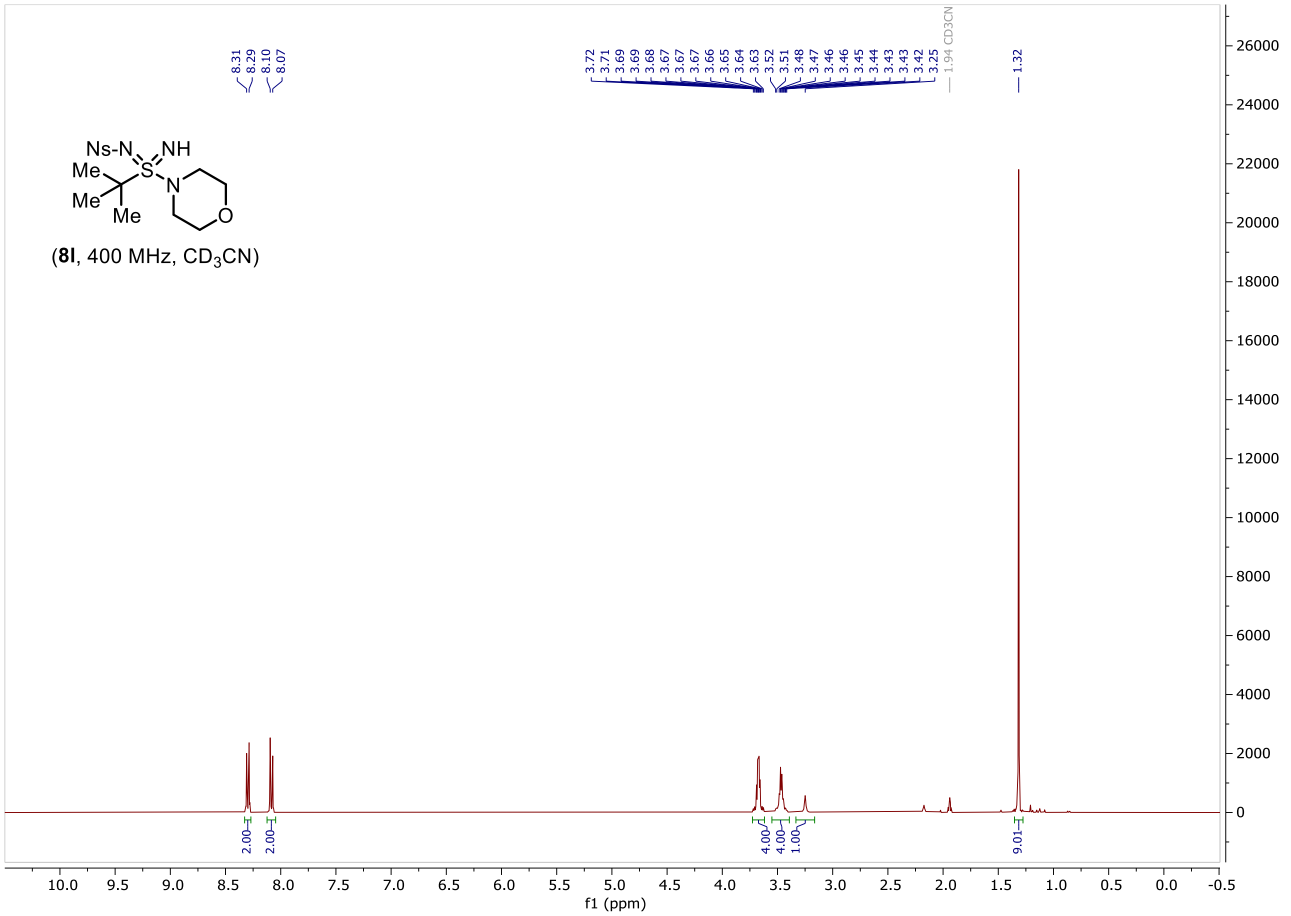
(8l, 400 MHz, CD3CN)
8.31
8.29
8.10
8.07
3.72
3.71
3.69
3.69
3.68
3.67
3.67
3.66
3.65
3.64
3.63
3.52
3.51
3.48
3.47
3.46
3.45
3.44
3.43
3.42
3.25
1.94 CD3CN
1.32
2.00
2.00
4.00
4.00
1.00
9.01
f1 (ppm)

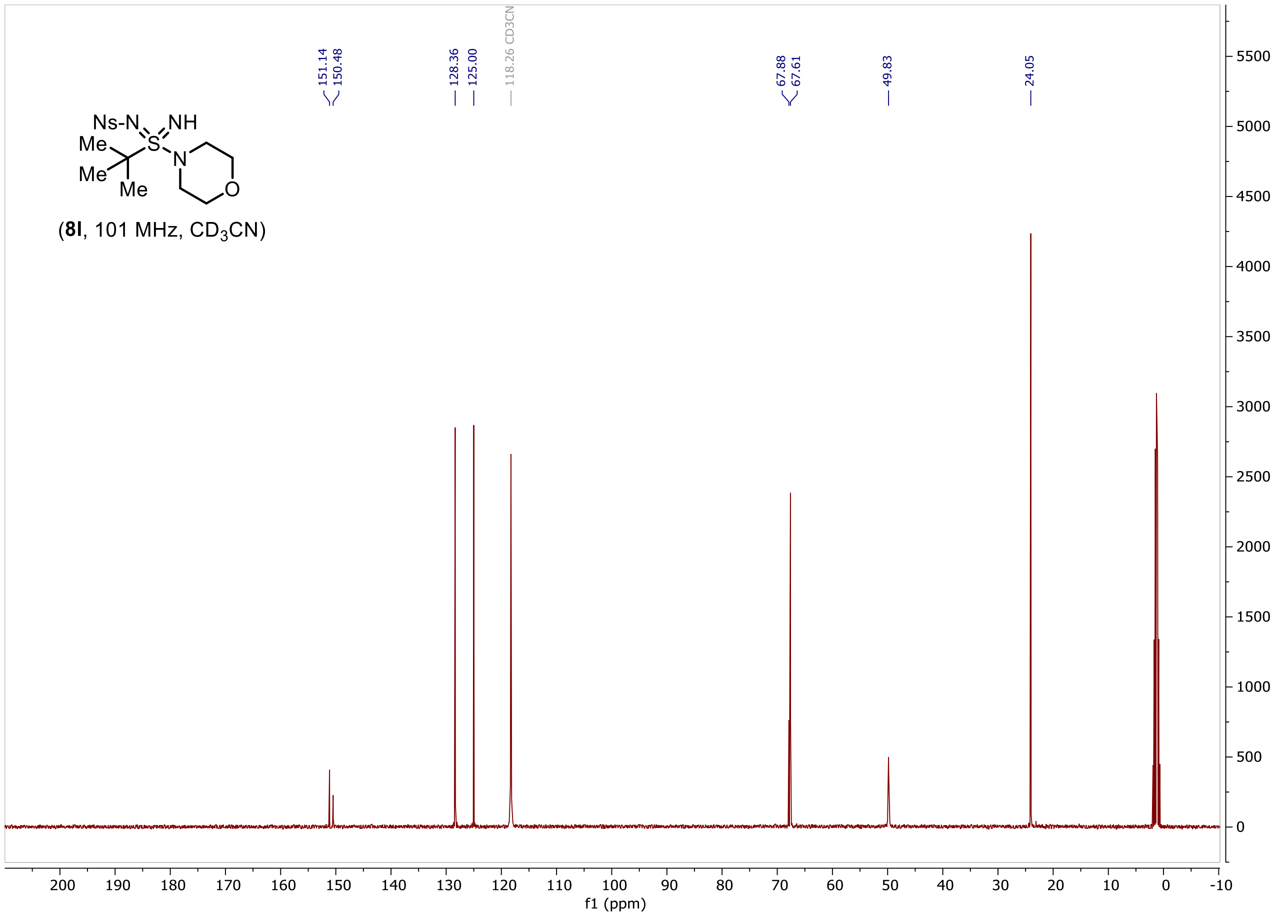

(**8l**, 101 MHz, CD_3CN)

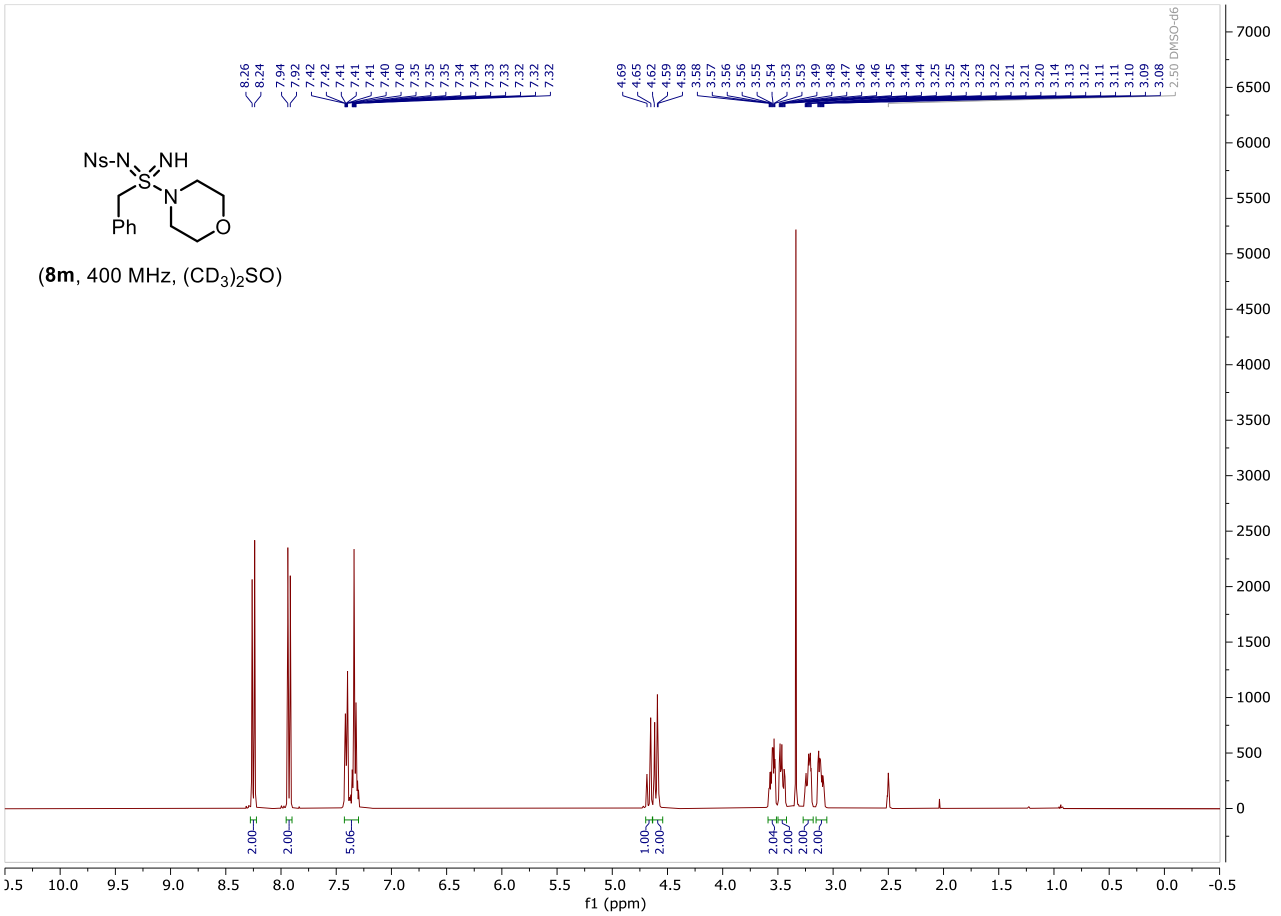

(**8m**, 400 MHz, $(CD_3)_2SO$)

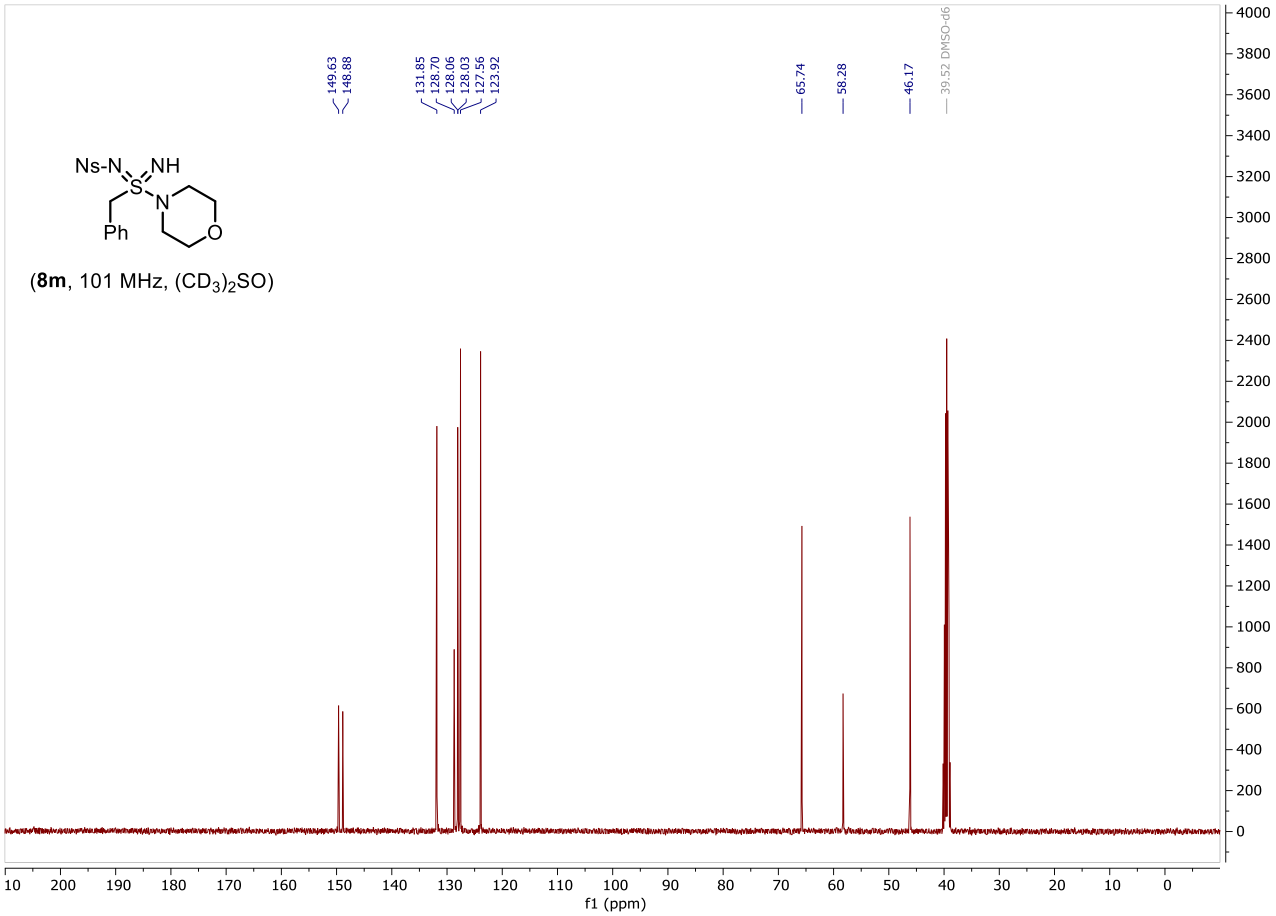
149.63
148.88
131.85
128.70
128.06
128.03
127.56
123.92
65.74
58.28
46.17
39.52 DMSO-d6
Ns-N
NH
S
N
O
Ph
(8m, 101 MHz, (CD3)2SO)
f1 (ppm)

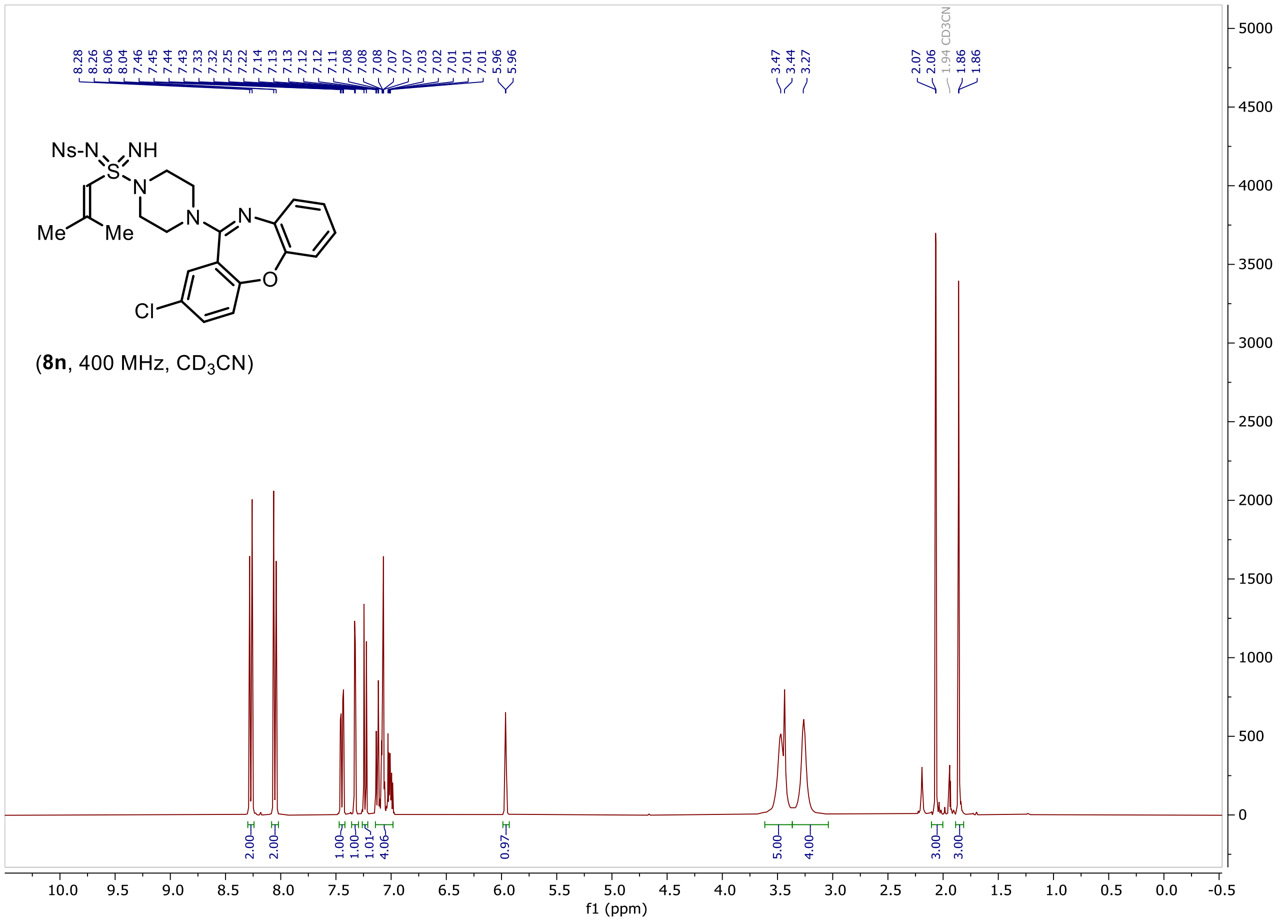
8.28
8.26
8.06
8.04
7.46
7.45
7.44
7.43
7.33
7.32
7.25
7.22
7.14
7.13
7.13
7.12
7.12
7.11
7.08
7.08
7.08
7.07
7.07
7.03
7.02
7.01
7.01
7.01
5.96
5.96
3.47
3.44
3.27
2.07
2.06
1.94 CD3CN
1.86
1.86
Ns-N
NH
S
N
N
N
O
Me
Me
Cl
(8n, 400 MHz, CD3CN)
2.00
2.00
1.00
1.00
1.01
4.06
0.97
5.00
4.00
3.00
3.00
f1 (ppm)

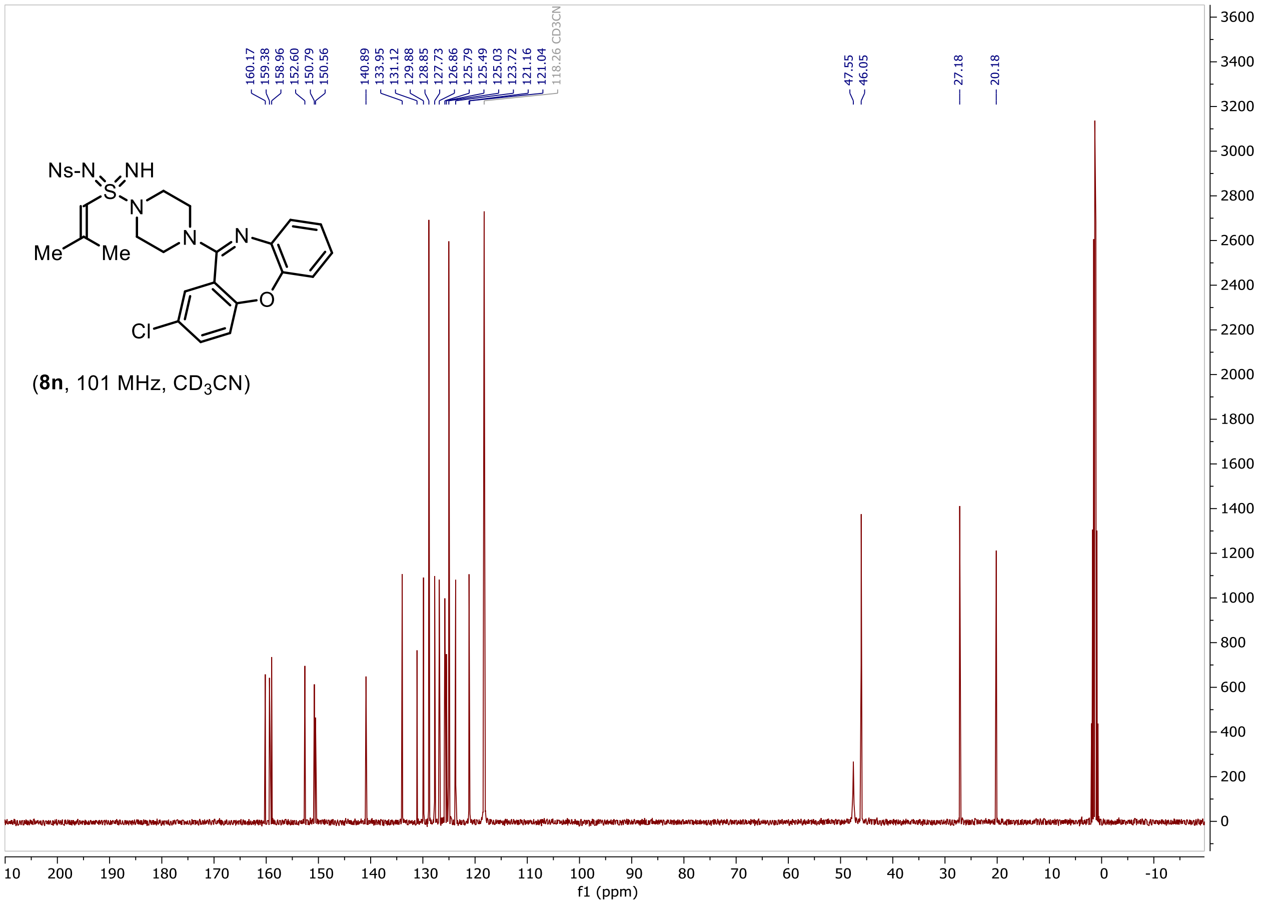
(8n, 101 MHz, CD3CN)
f1 (ppm)

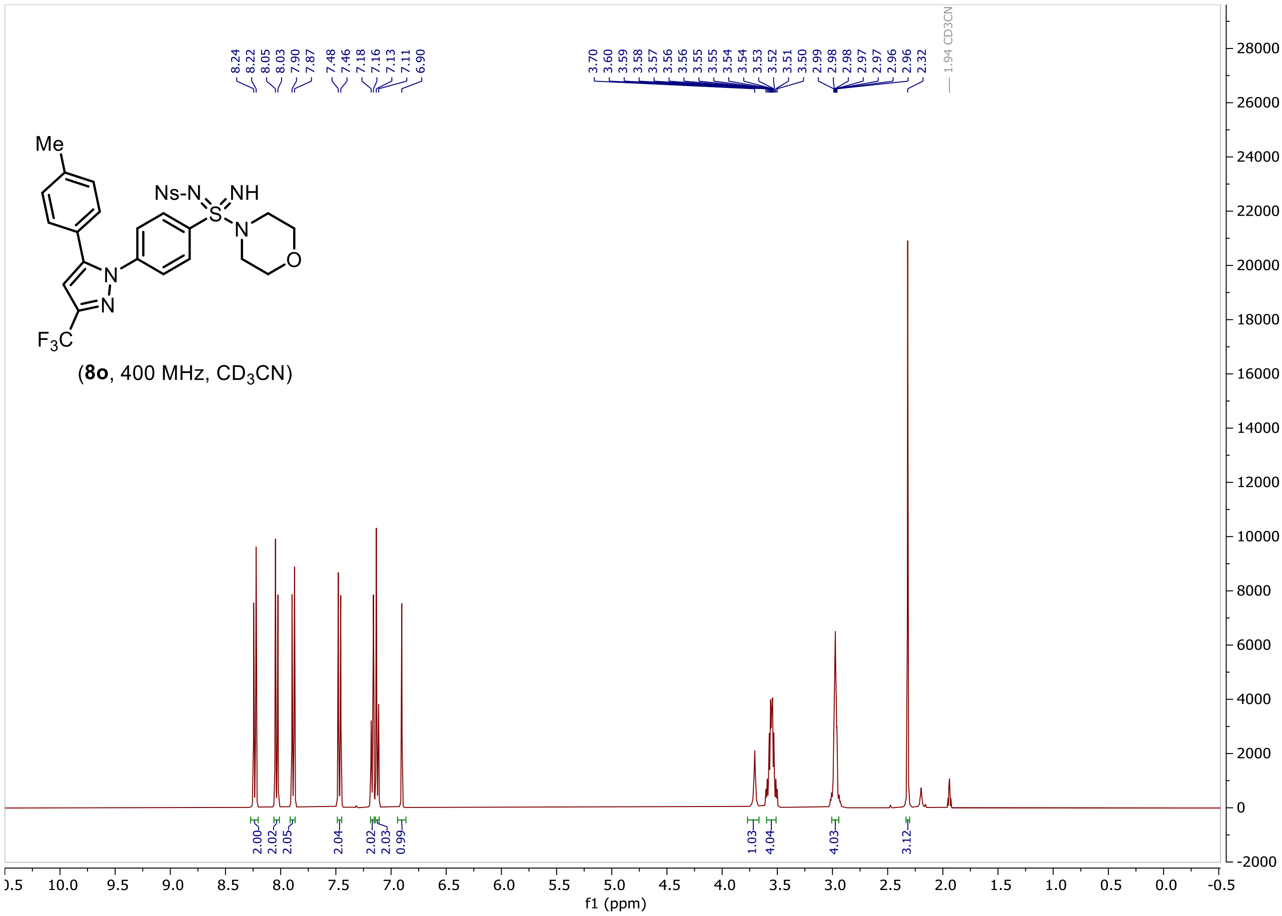
(8o, 400 MHz, CD3CN)
f1 (ppm)

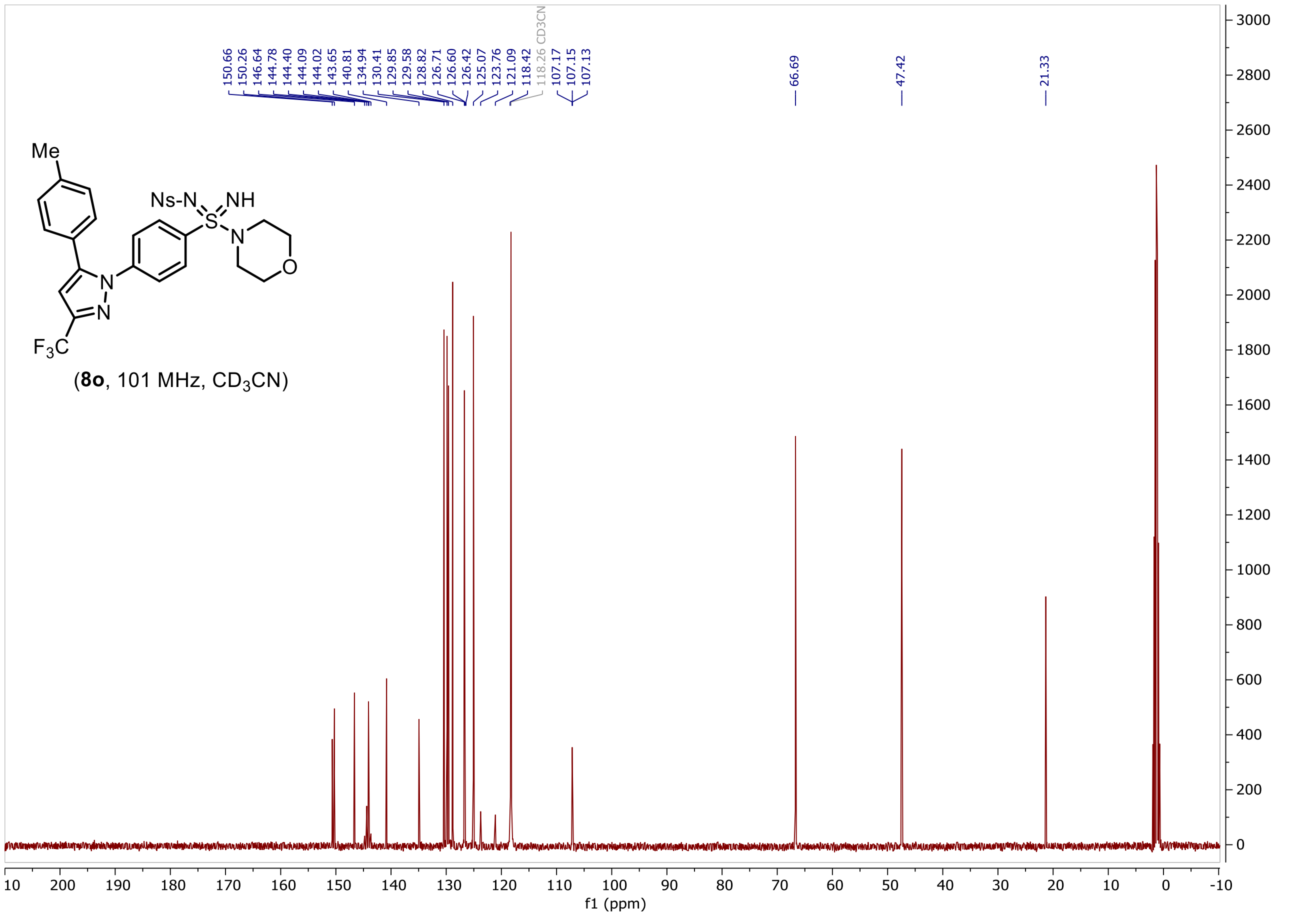

(**8o**, 101 MHz, CD_3CN)

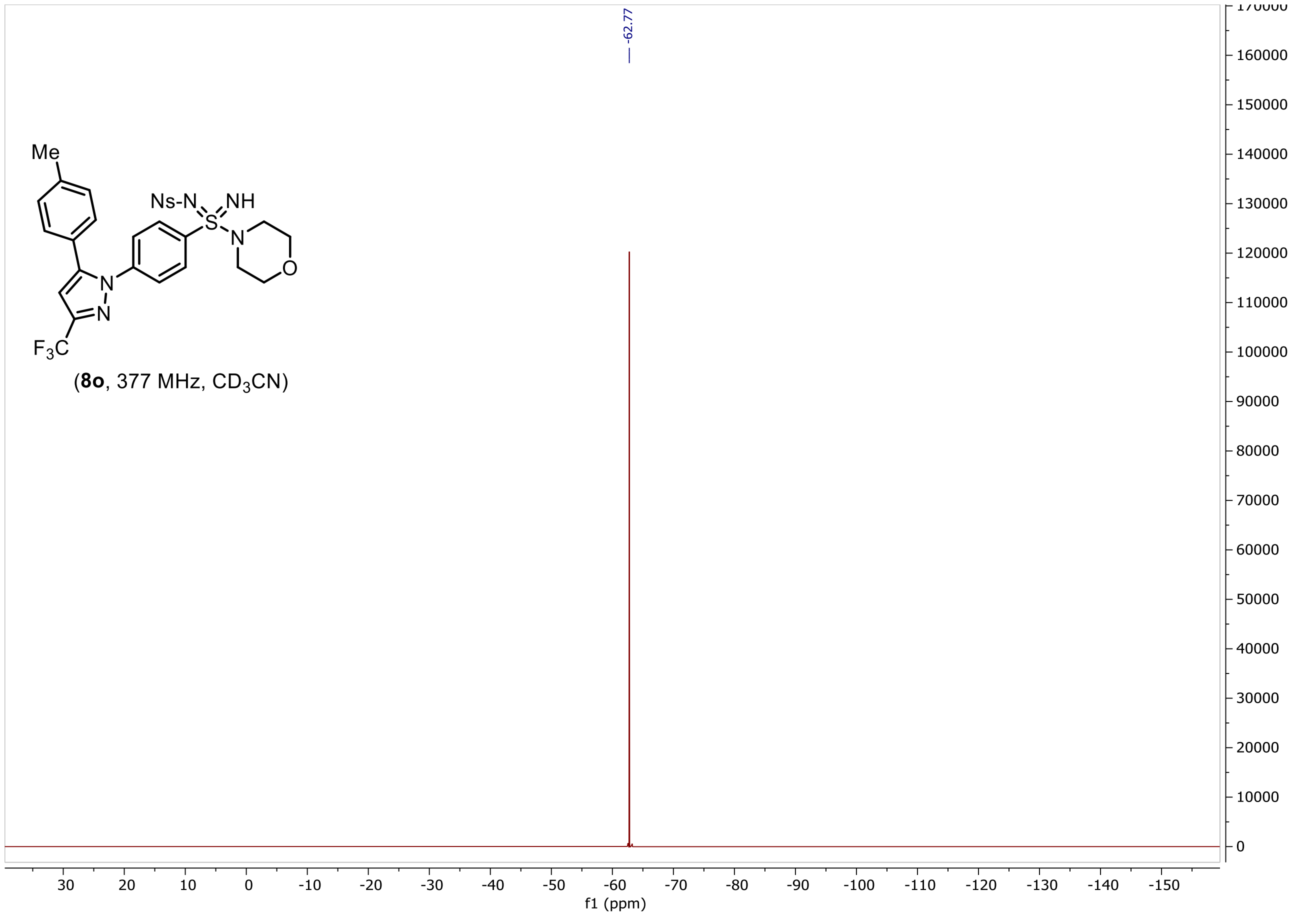

(**8o**, 377 MHz, CD_3CN)

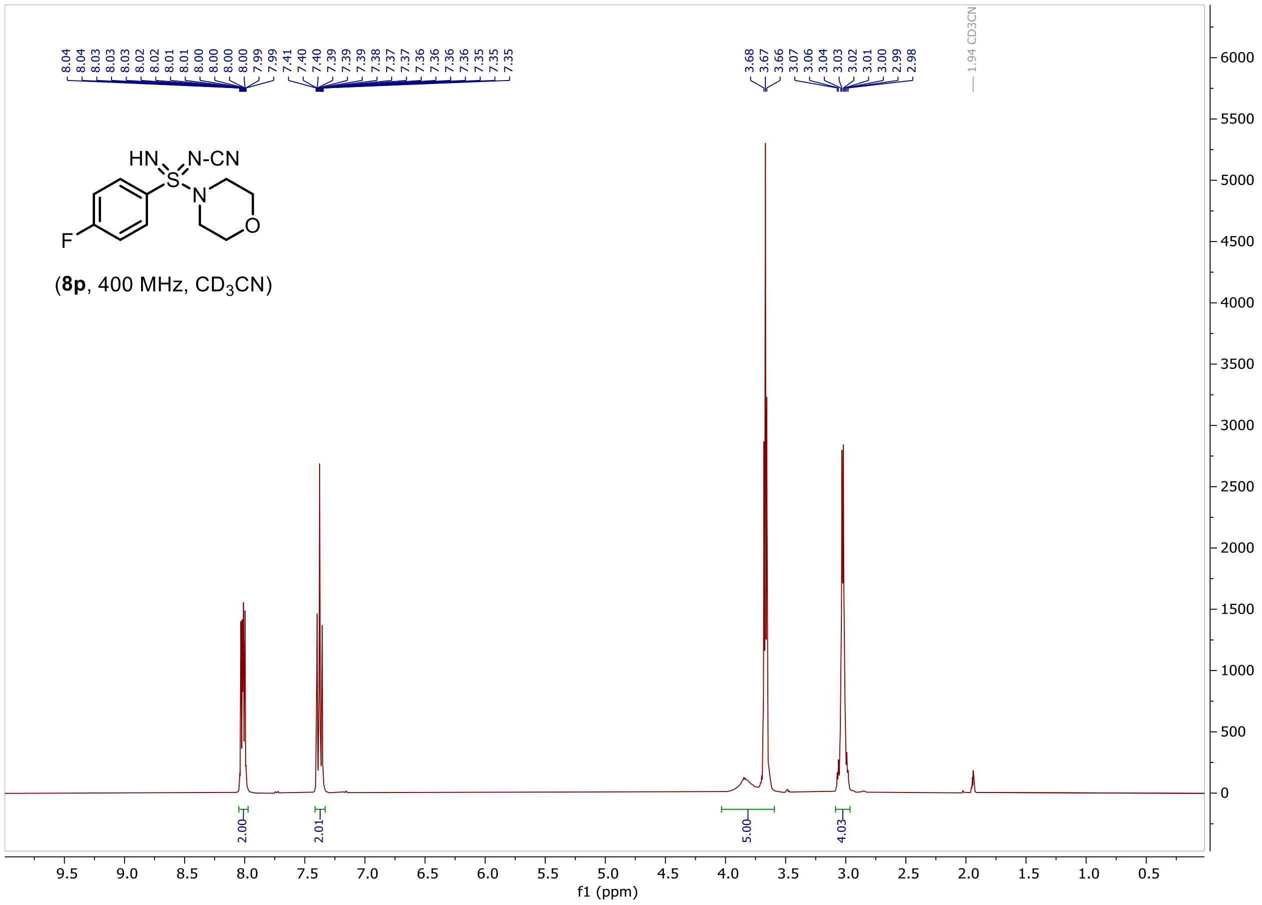
8.04
8.04
8.03
8.03
8.02
8.02
8.01
8.01
8.00
8.00
8.00
8.00
7.99
7.41
7.40
7.40
7.39
7.39
7.39
7.38
7.37
7.36
7.36
7.36
7.36
7.35
7.35
7.35
3.68
3.67
3.66
3.07
3.06
3.04
3.03
3.02
3.01
3.00
2.99
2.98
1.94 CD3CN
HN
N-CN
S
N
O
F
(**8p**, 400 MHz, CD_3CN)
2.00
2.01
5.00
4.03
f1 (ppm)

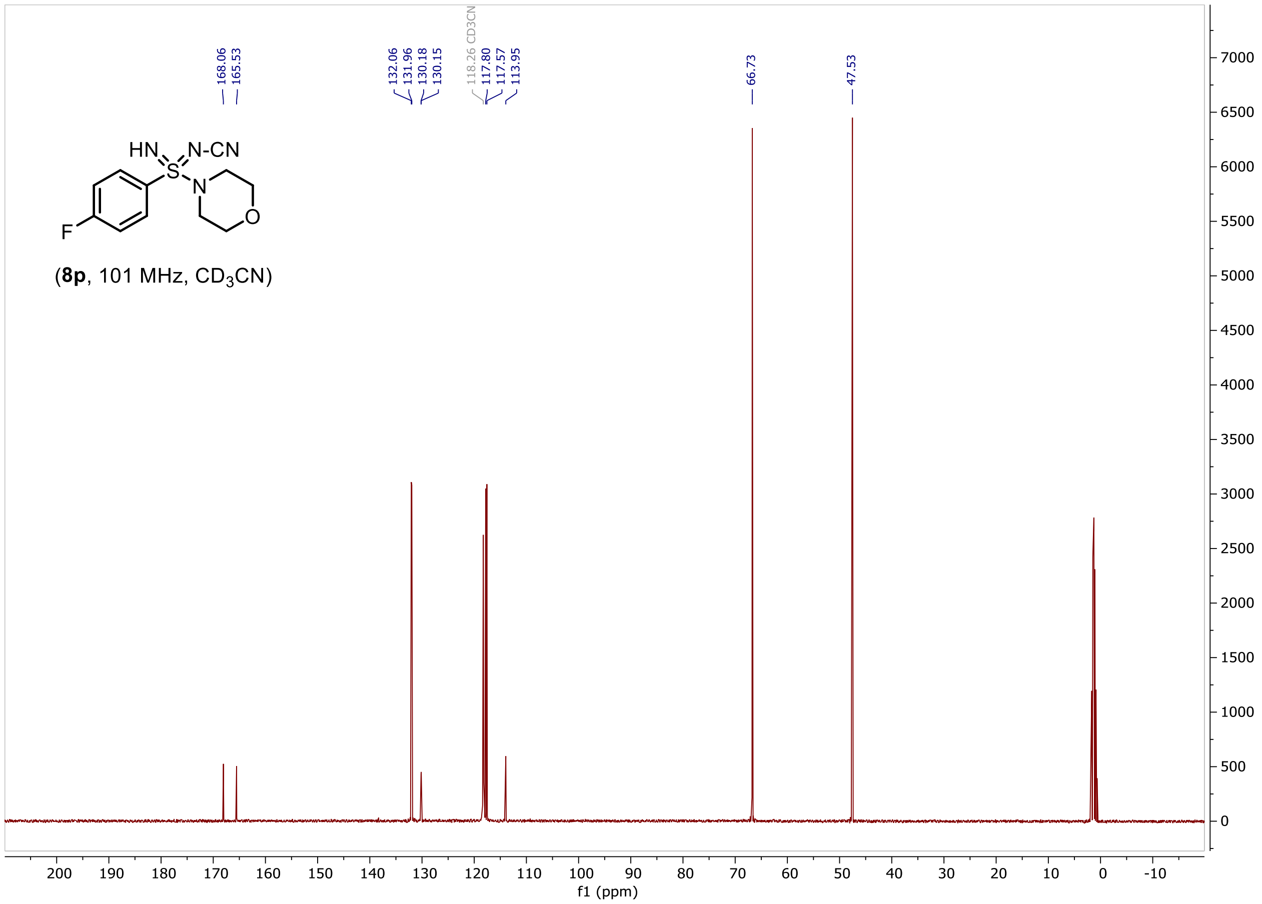
168.06
165.53
132.06
131.96
130.18
130.15
118.26 CD3CN
117.80
117.57
113.95
66.73
47.53
HN
N-CN
S
N
O
F
(**8p**, 101 MHz, CD_3CN)
f1 (ppm)

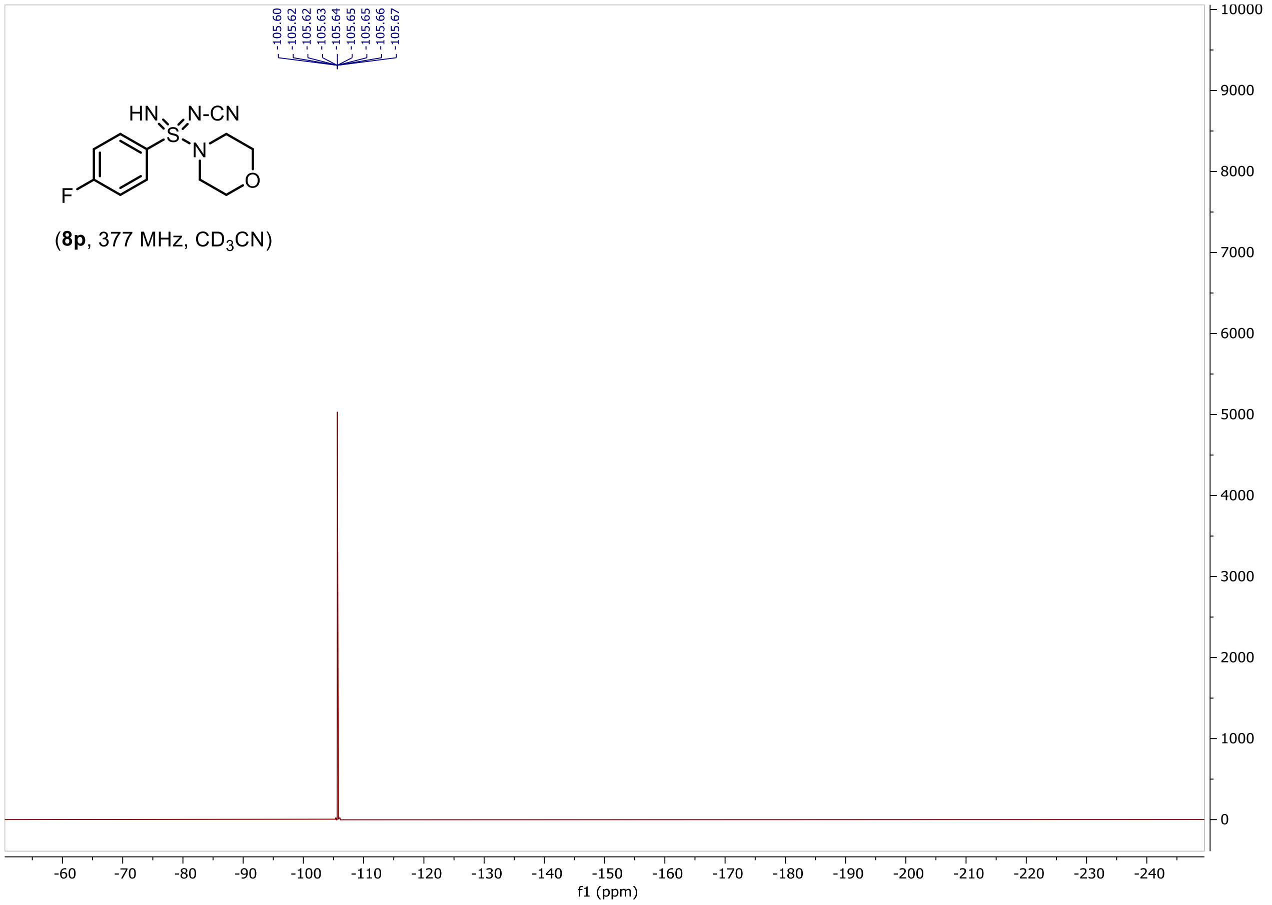
-105.60
-105.62
-105.62
-105.63
-105.64
-105.65
-105.65
-105.66
-105.67
HN
N-CN
S
N
O
F
(8p, 377 MHz, CD3CN)
f1 (ppm)

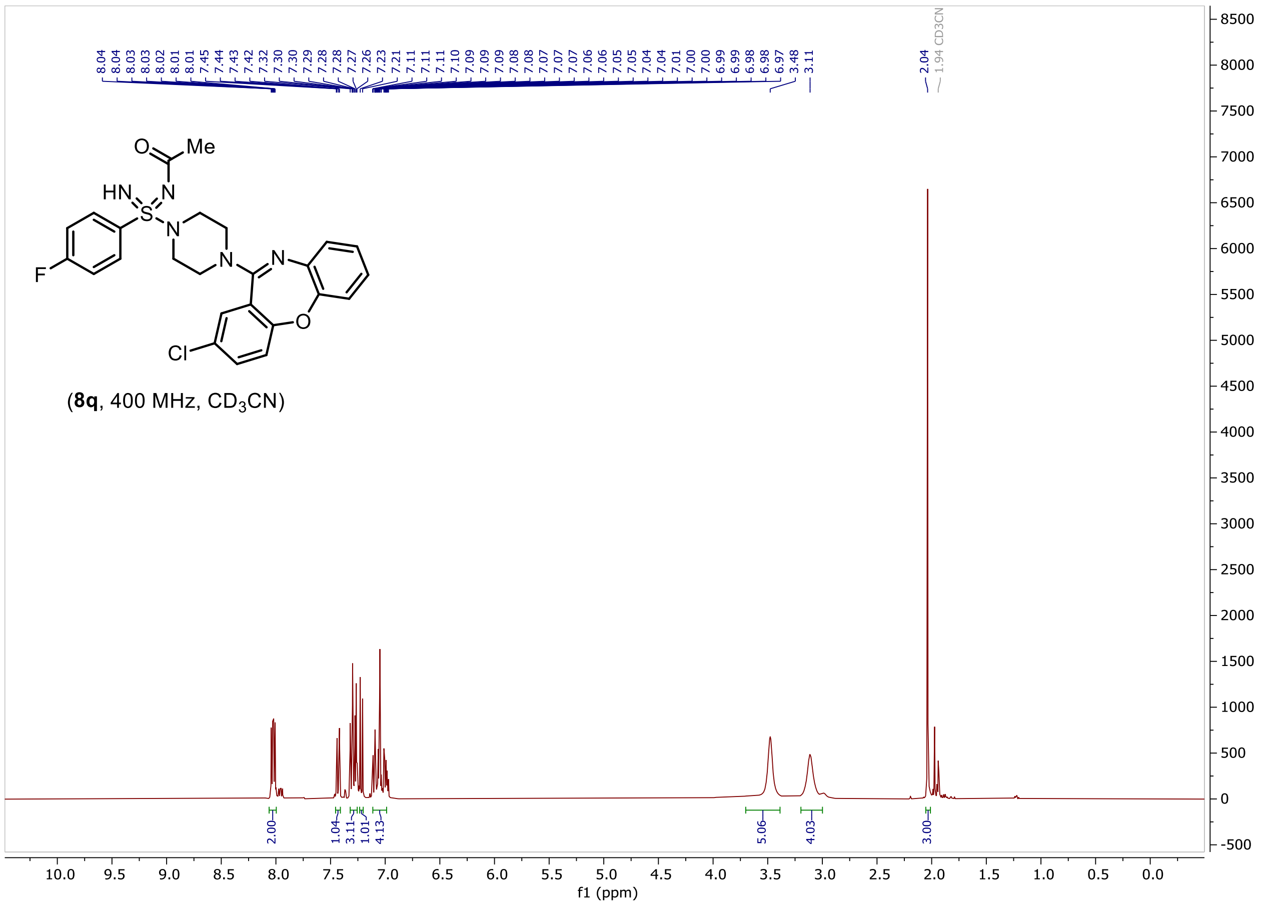
8.04
8.04
8.03
8.02
8.01
8.01
7.45
7.44
7.43
7.42
7.32
7.30
7.30
7.29
7.28
7.28
7.27
7.26
7.23
7.21
7.11
7.11
7.11
7.10
7.09
7.09
7.09
7.08
7.08
7.07
7.07
7.06
7.05
7.05
7.04
7.04
7.01
7.00
7.00
6.99
6.99
6.98
6.97
3.48
3.11
2.04
1.94 CD3CN
O
Me
HN
N
S
N
N
N
O
F
Cl
(8q, 400 MHz, CD3CN)
2.00
1.04
3.11
1.01
4.13
5.06
4.03
3.00
f1 (ppm)

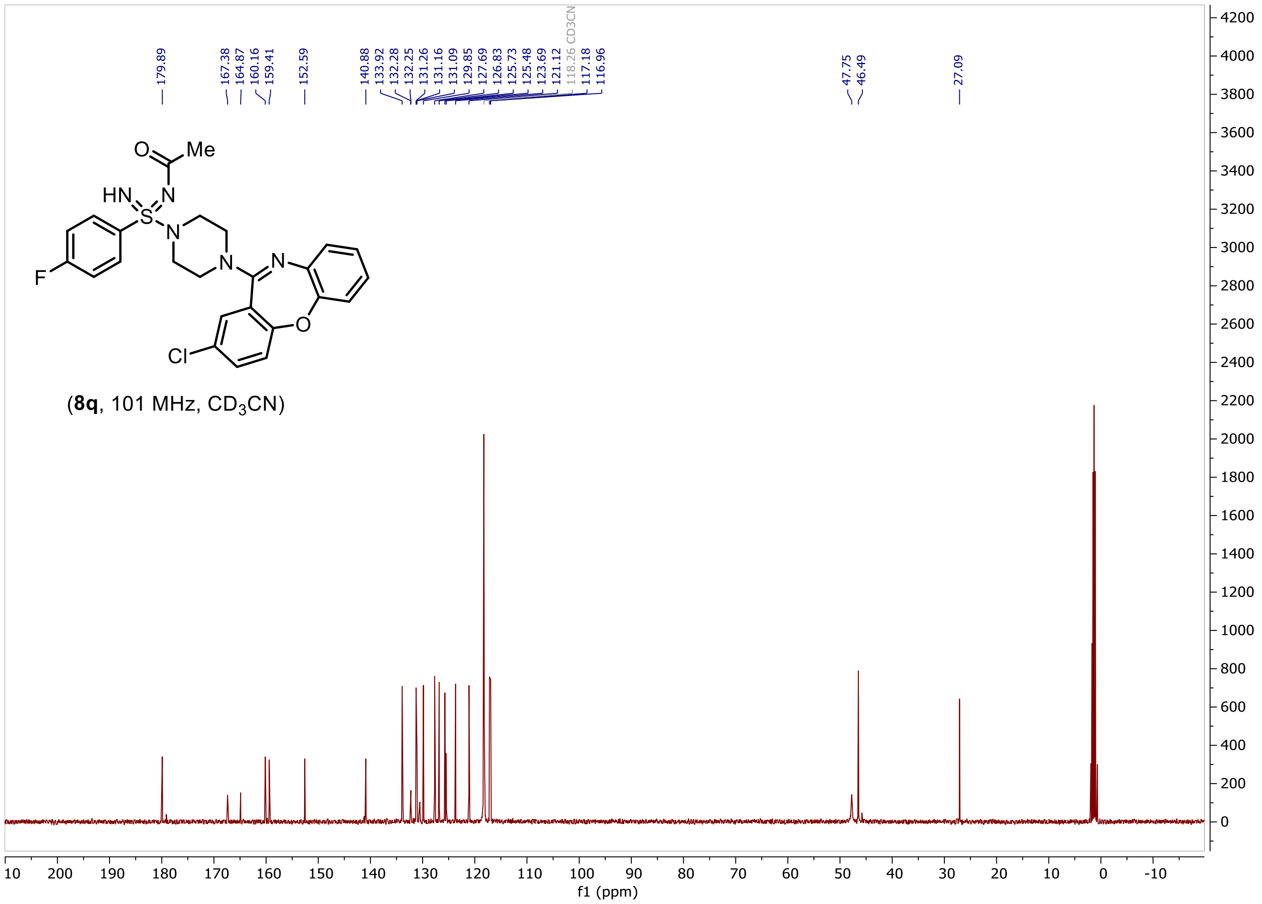
179.89
167.38
164.87
160.16
159.41
152.59
140.88
133.92
132.28
132.25
131.26
131.16
131.09
129.85
127.69
126.83
125.73
125.48
123.69
121.12
118.26 CD3CN
117.18
116.96
47.75
46.49
27.09
O
Me
HN
N
S
N
N
N
F
O
Cl
(**8q**, 101 MHz, CD$_3$CN)
f1 (ppm)

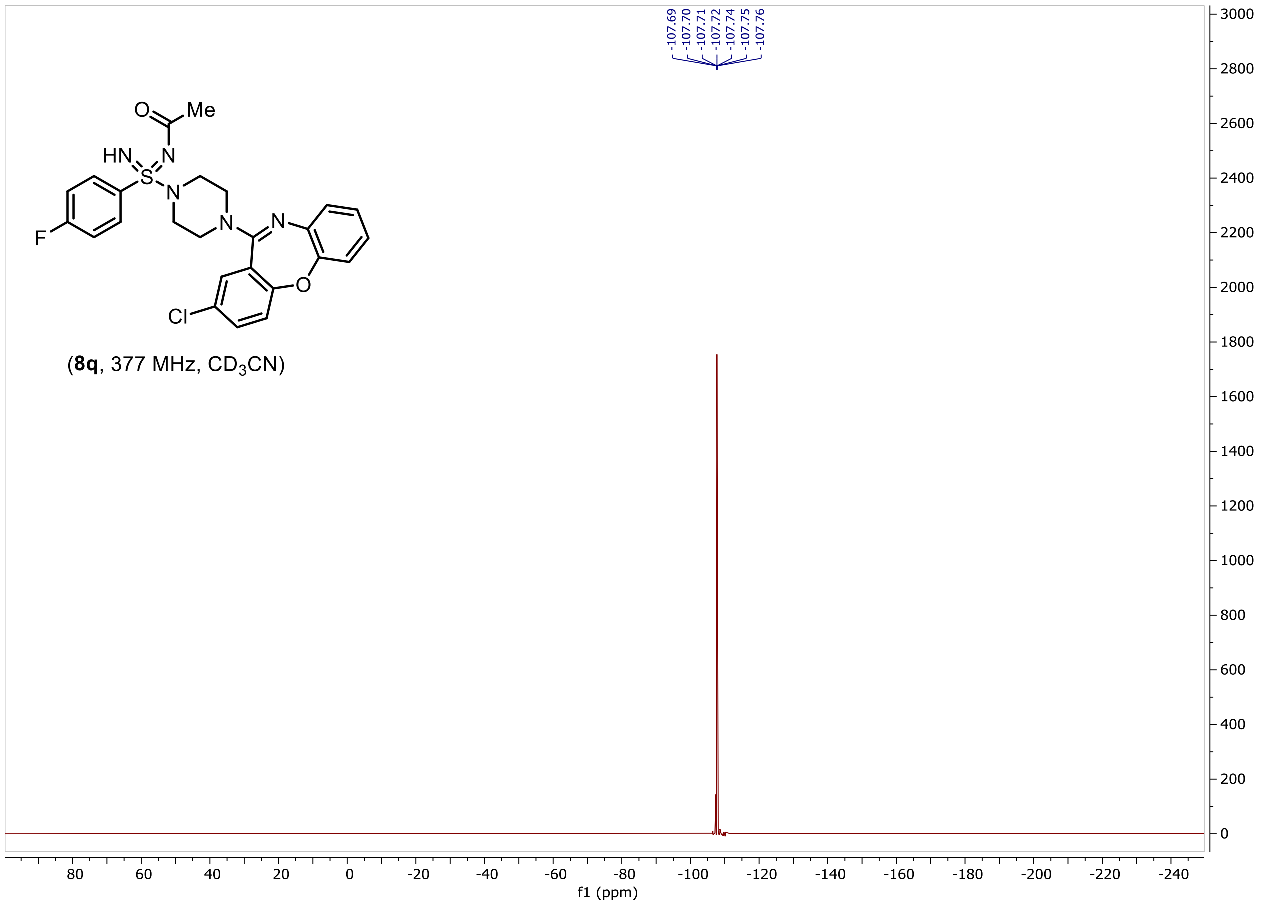
-107.69
-107.70
-107.71
-107.72
-107.74
-107.75
-107.76
O
Me
HN
N
S
N
N
N
F
O
Cl
(**8q**, 377 MHz, CD$_3$CN)
f1 (ppm)

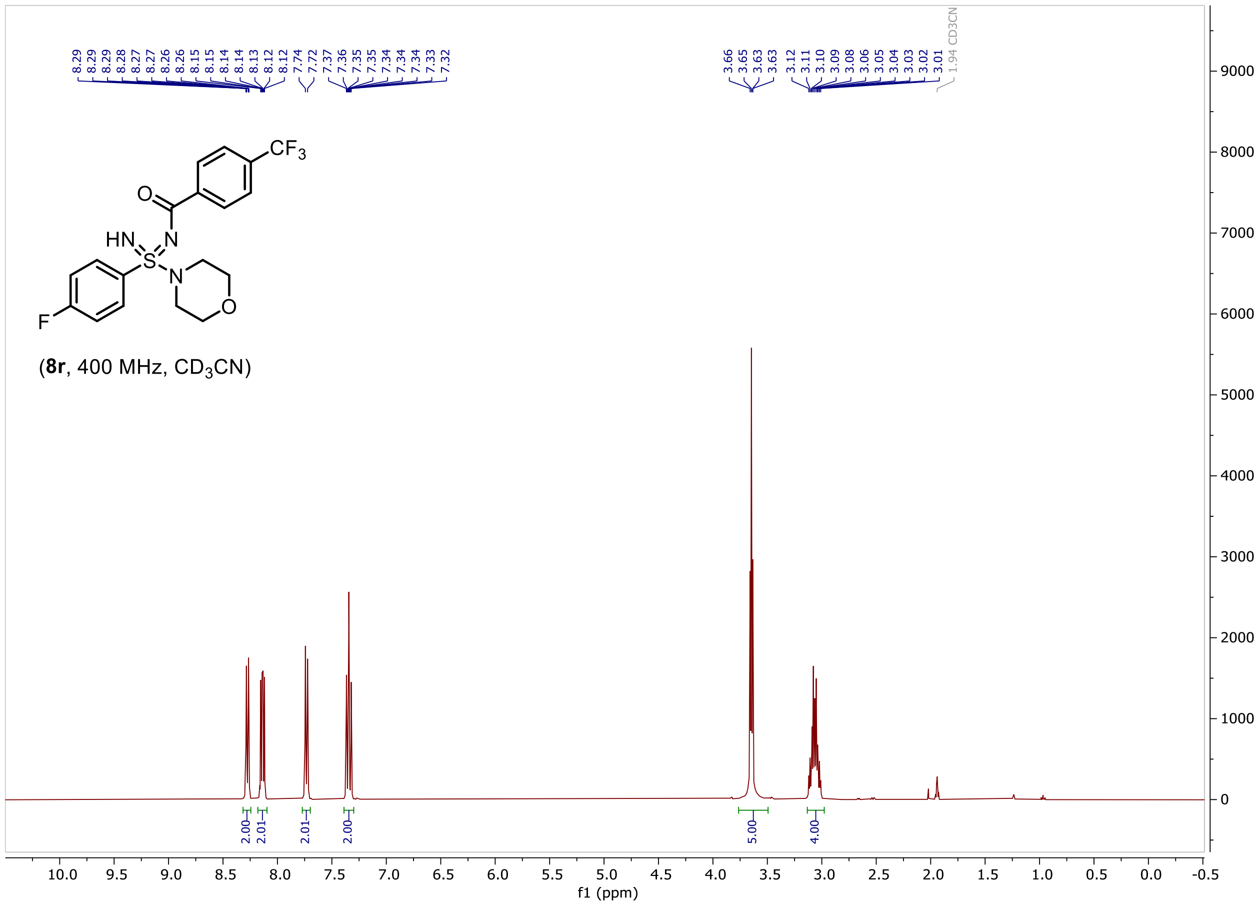
(8r, 400 MHz, CD3CN)
f1 (ppm)

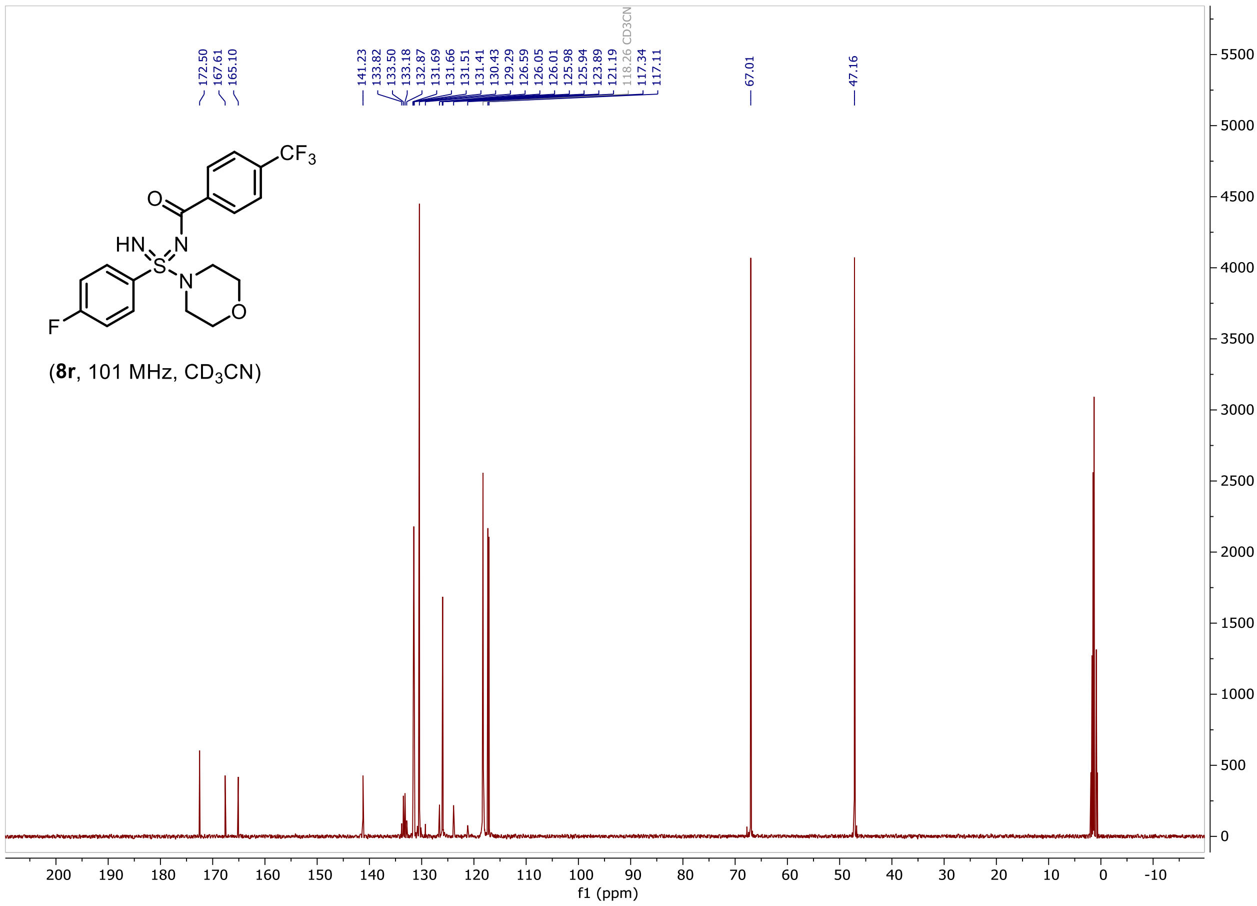
(8r, 101 MHz, CD3CN)
f1 (ppm)

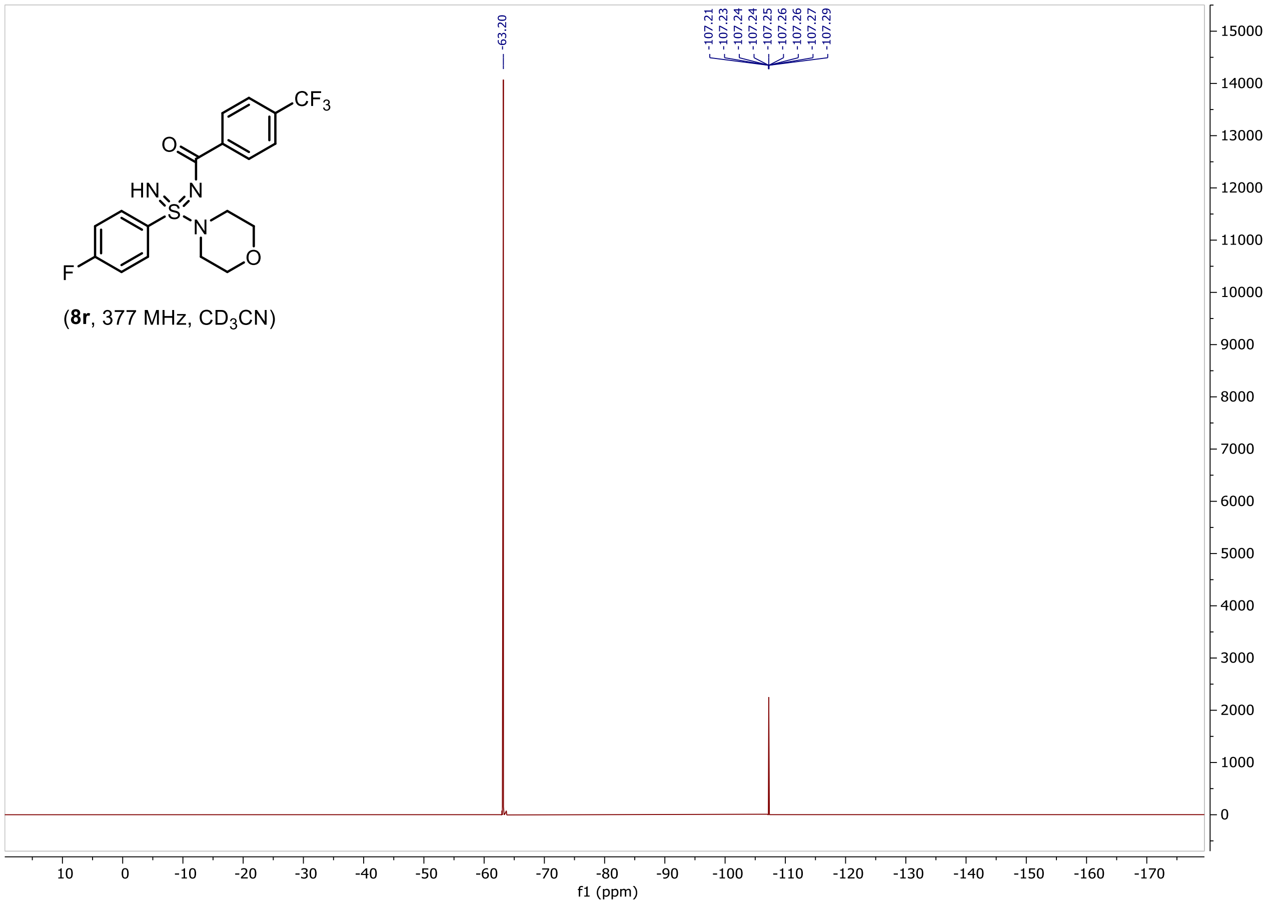

(8r, 377 MHz, CD3CN)
f1 (ppm)

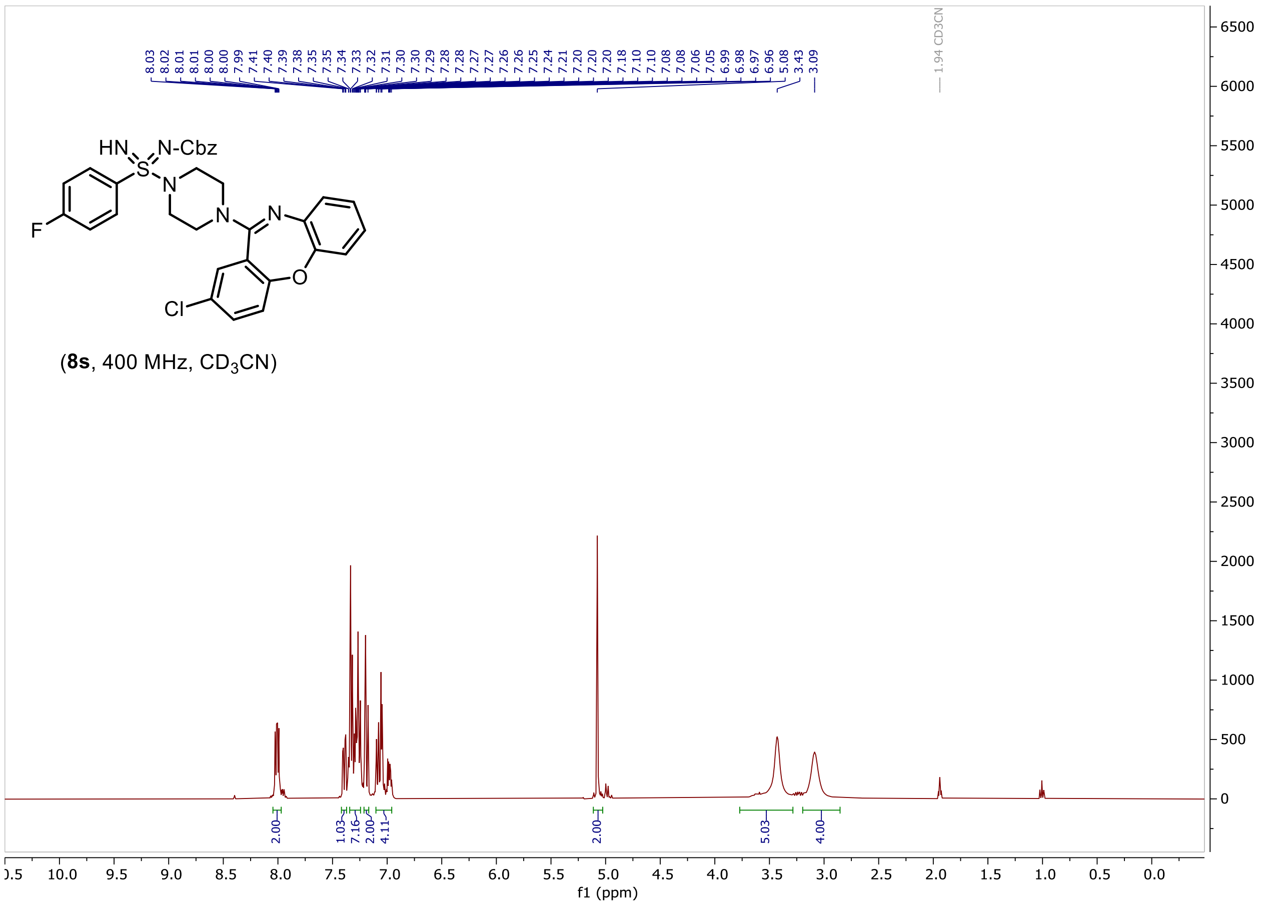

HN
N-Cbz
F
Cl
(8s, 400 MHz, CD3CN)
f1 (ppm)

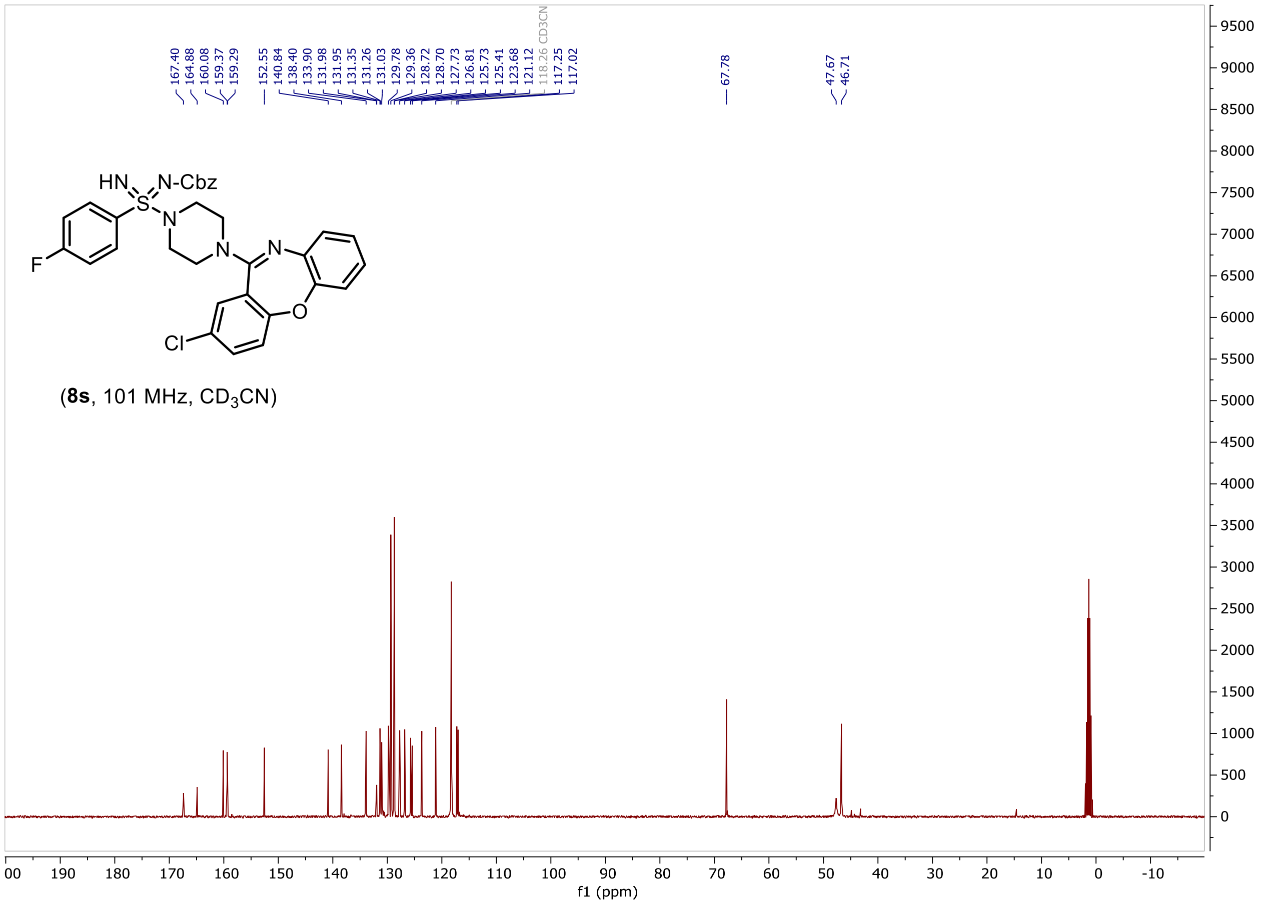
167.40
164.88
160.08
159.37
159.29
152.55
140.84
138.40
133.90
131.98
131.95
131.35
131.26
131.03
129.78
129.36
128.72
128.70
127.73
126.81
125.73
125.41
123.68
121.12
118.26 CD3CN
117.25
117.02
67.78
47.67
46.71
HN
N-Cbz
S
N
N
N
O
F
Cl
(8s, 101 MHz, CD_3CN)
f1 (ppm)

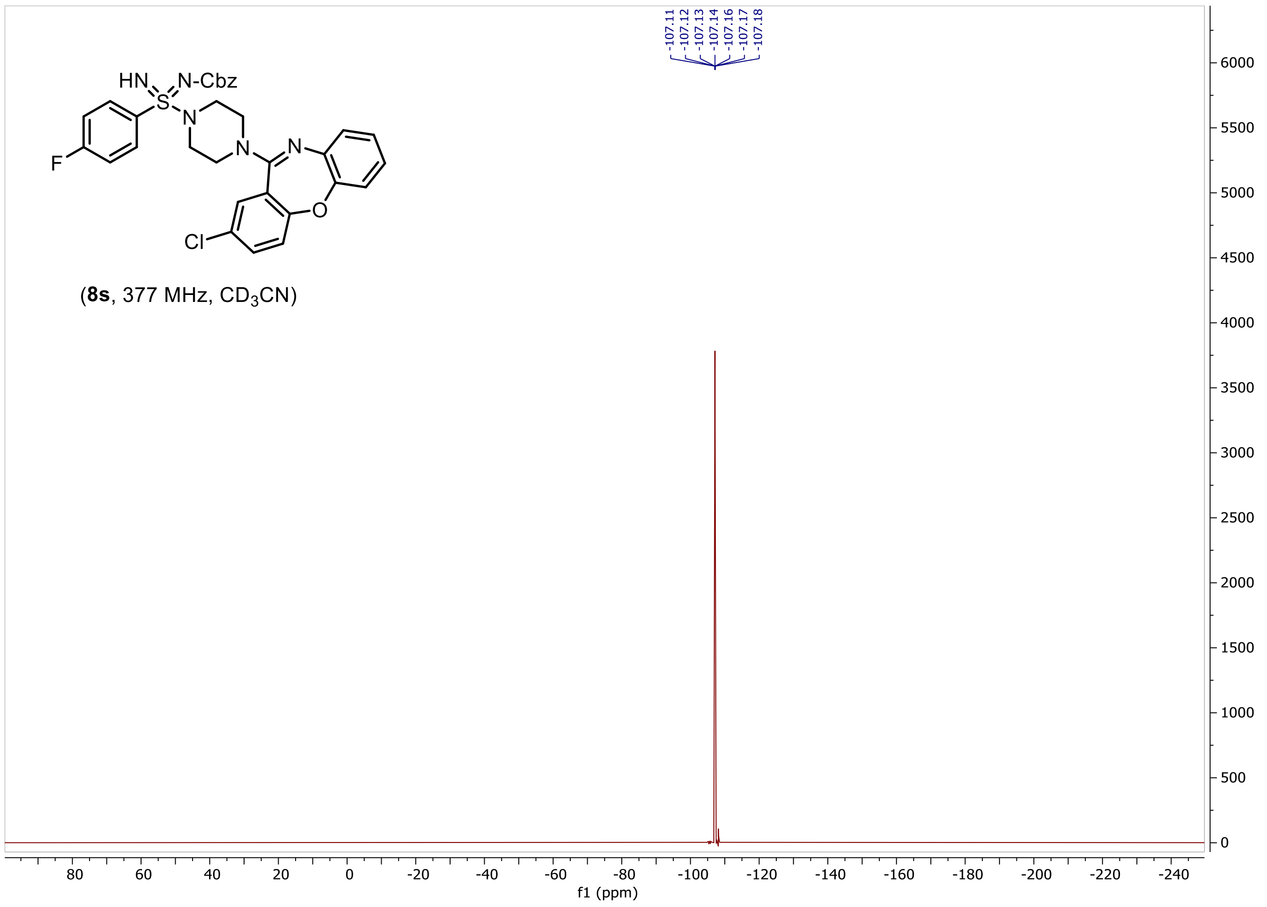
-107.11
-107.12
-107.13
-107.14
-107.16
-107.17
-107.18
HN
N-Cbz
S
N
N
N
O
F
Cl
(8s, 377 MHz, CD_3CN)
f1 (ppm)

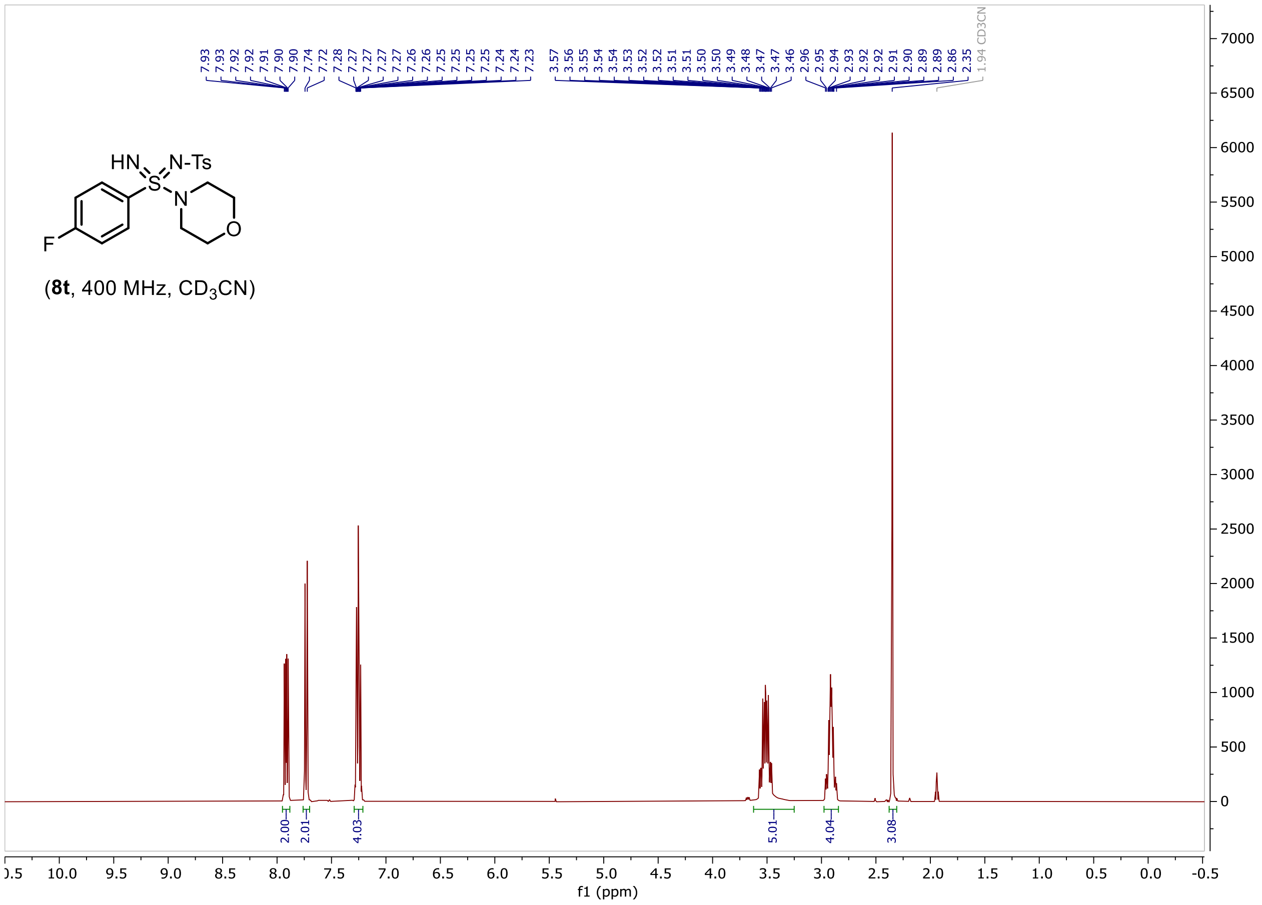
7.93
7.93
7.92
7.92
7.91
7.90
7.90
7.74
7.72
7.28
7.27
7.27
7.27
7.27
7.26
7.26
7.25
7.25
7.25
7.25
7.24
7.24
7.23
3.57
3.56
3.55
3.54
3.54
3.53
3.52
3.52
3.51
3.51
3.50
3.50
3.49
3.48
3.47
3.47
3.46
2.96
2.95
2.94
2.93
2.92
2.92
2.91
2.90
2.89
2.89
2.86
2.35
1.94 CD3CN
HN
N-Ts
S
N
O
F
(8t, 400 MHz, CD3CN)
2.00
2.01
4.03
5.01
4.04
3.08
f1 (ppm)

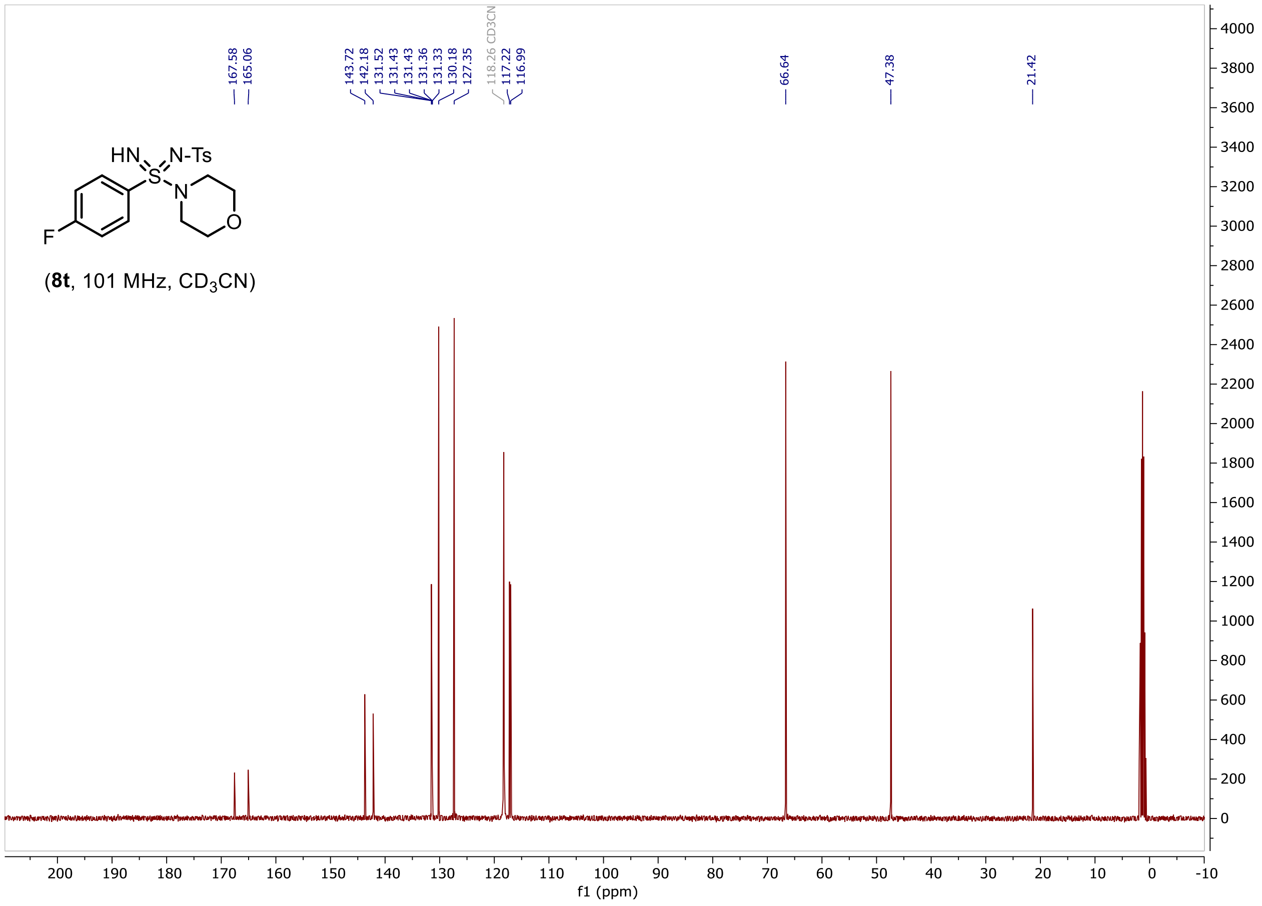
167.58
165.06
143.72
142.18
131.52
131.43
131.43
131.36
131.33
130.18
127.35
118.26 CD3CN
117.22
116.99
66.64
47.38
21.42
HN
N-Ts
S
N
O
F
(8t, 101 MHz, CD3CN)
f1 (ppm)

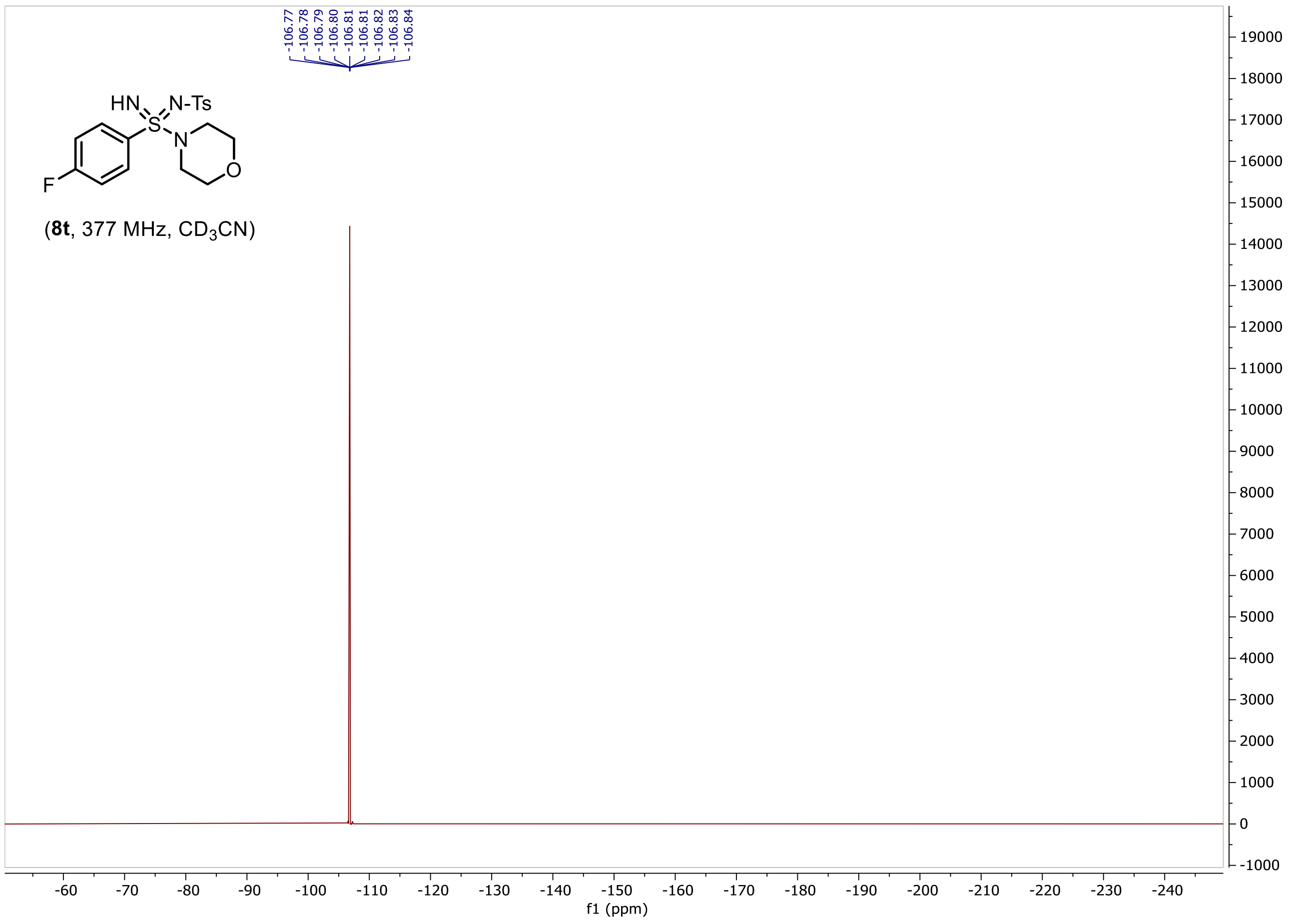

(**8t**, 377 MHz, CD_3CN)

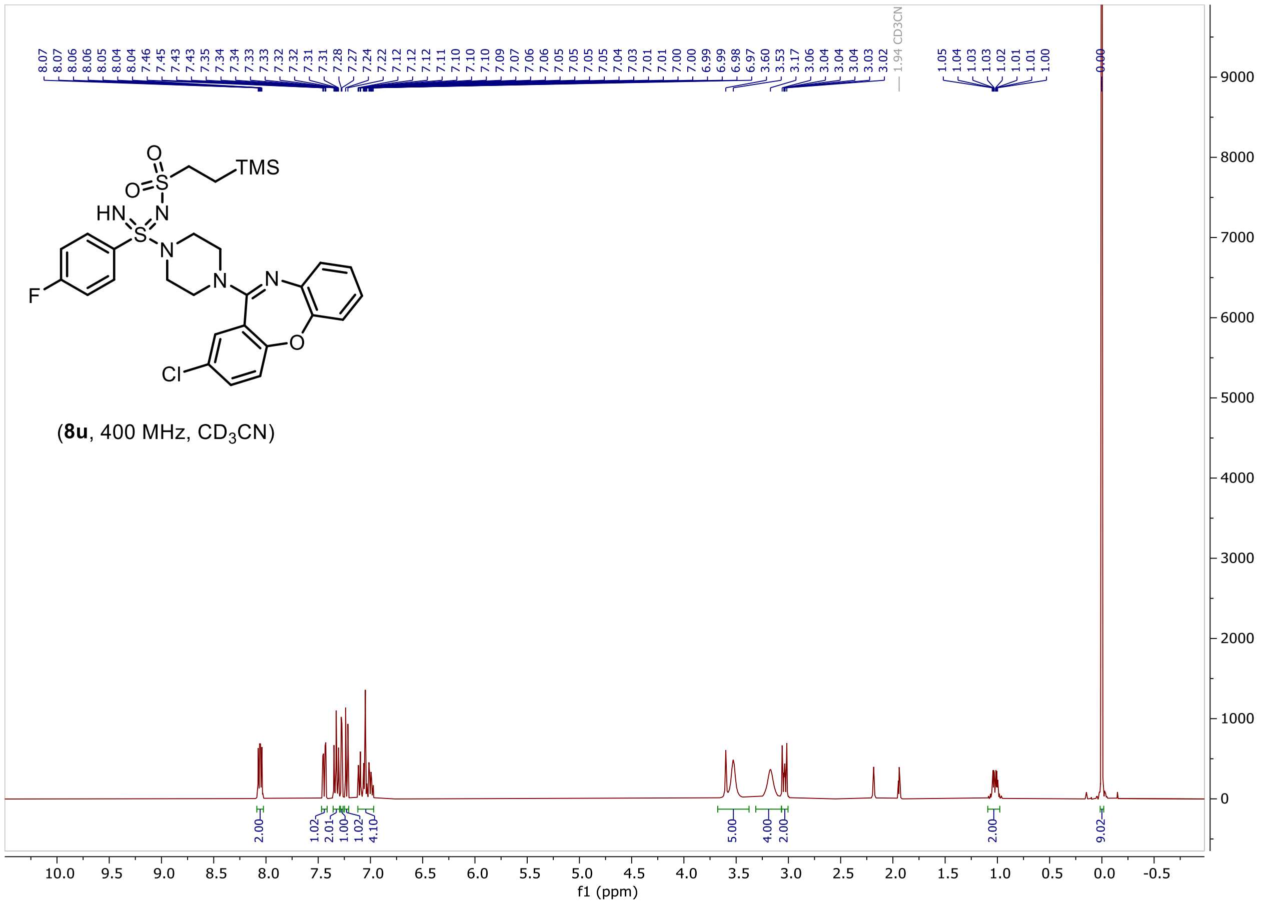

(**8u**, 400 MHz, CD_3CN)

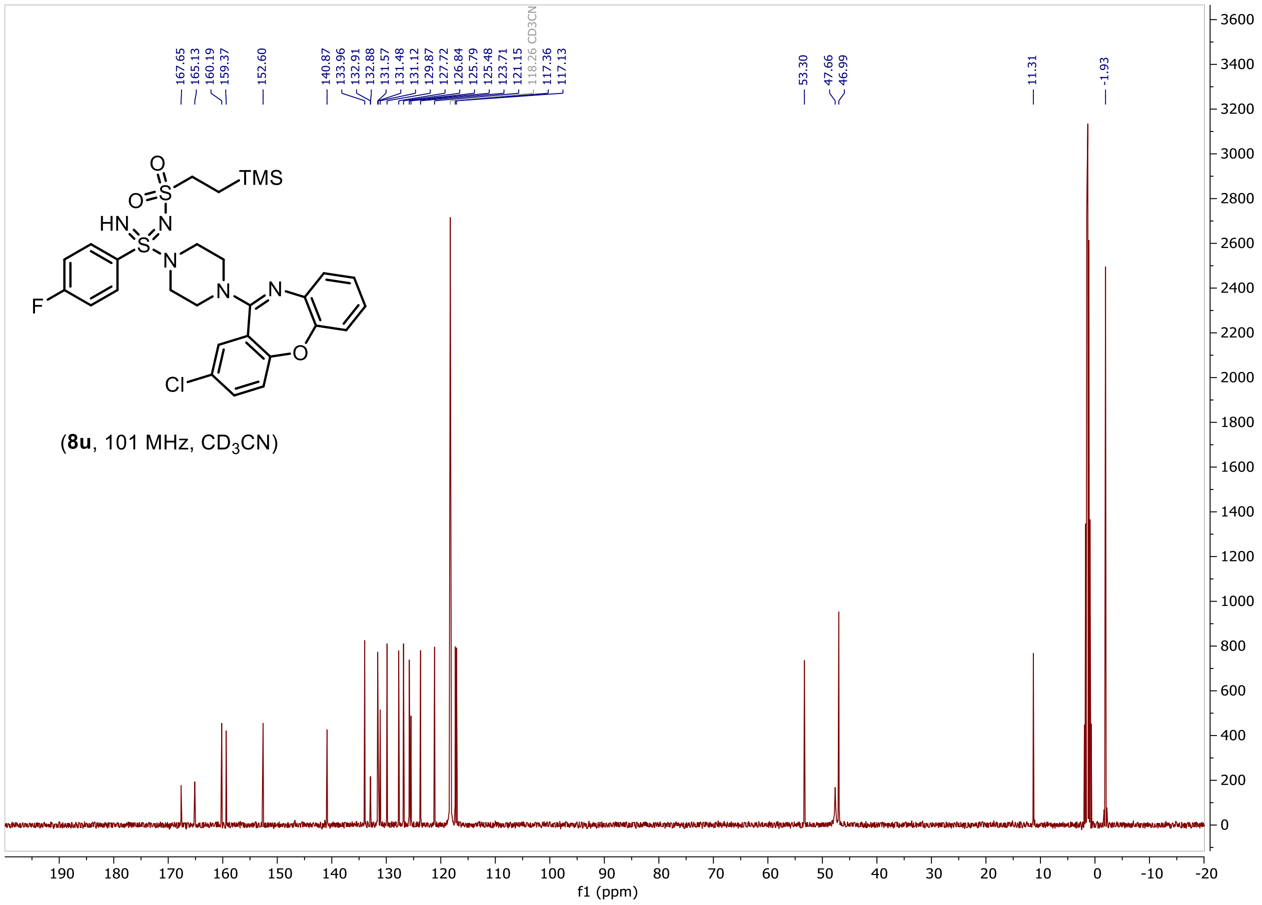
167.65
165.13
160.19
159.37
152.60
140.87
133.96
132.91
132.88
131.57
131.48
131.12
129.87
127.72
126.84
125.79
125.48
123.71
121.15
118.26 CD3CN
117.36
117.13
53.30
47.66
46.99
11.31
-1.93
TMS
O
HN
S
N
F
N
N
N
Cl
O
(8u, 101 MHz, CD3CN)
f1 (ppm)

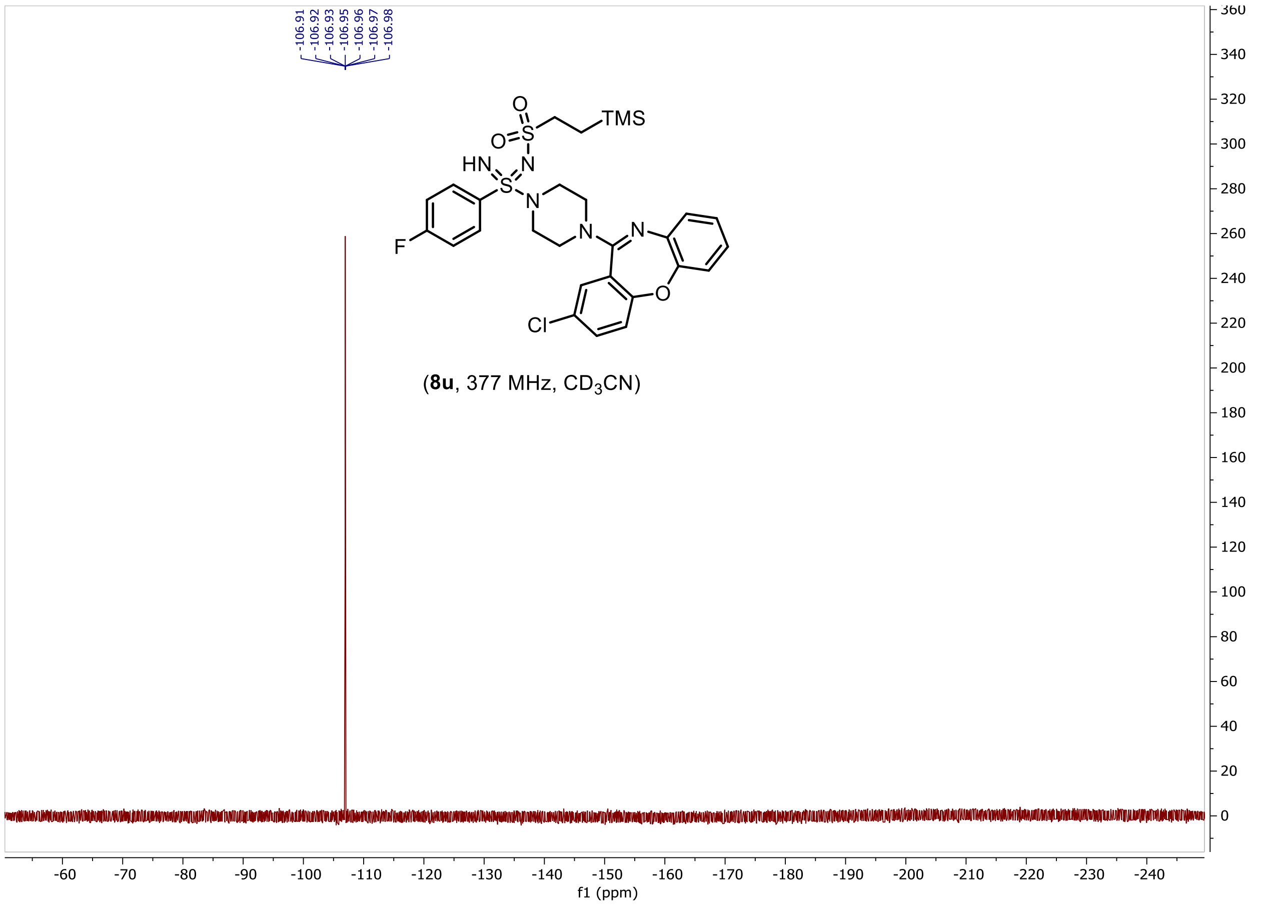
-106.91
-106.92
-106.93
-106.95
-106.96
-106.97
-106.98
TMS
O
HN
S
N
F
N
N
N
Cl
O
(8u, 377 MHz, CD3CN)
f1 (ppm)

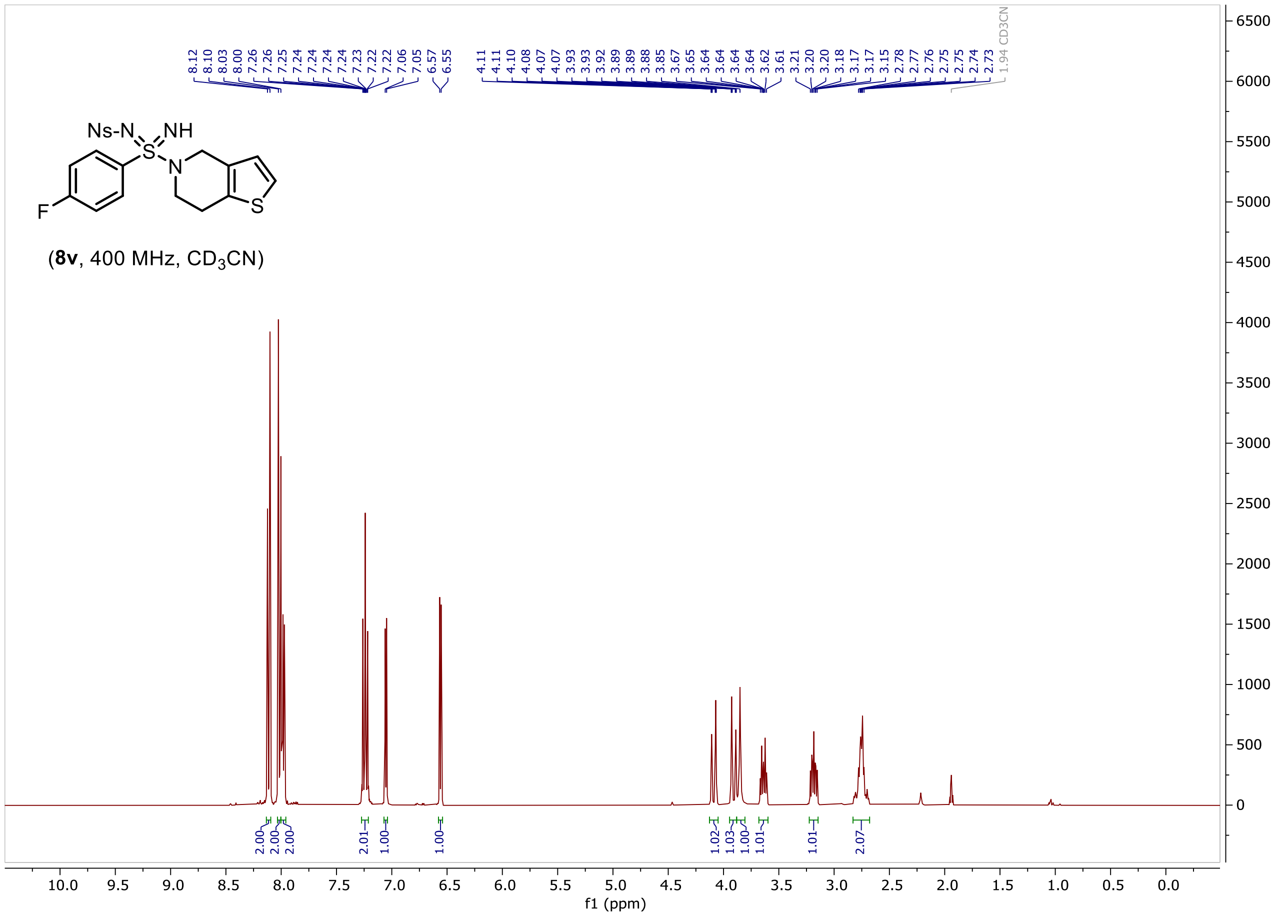
(8v, 400 MHz, CD3CN)

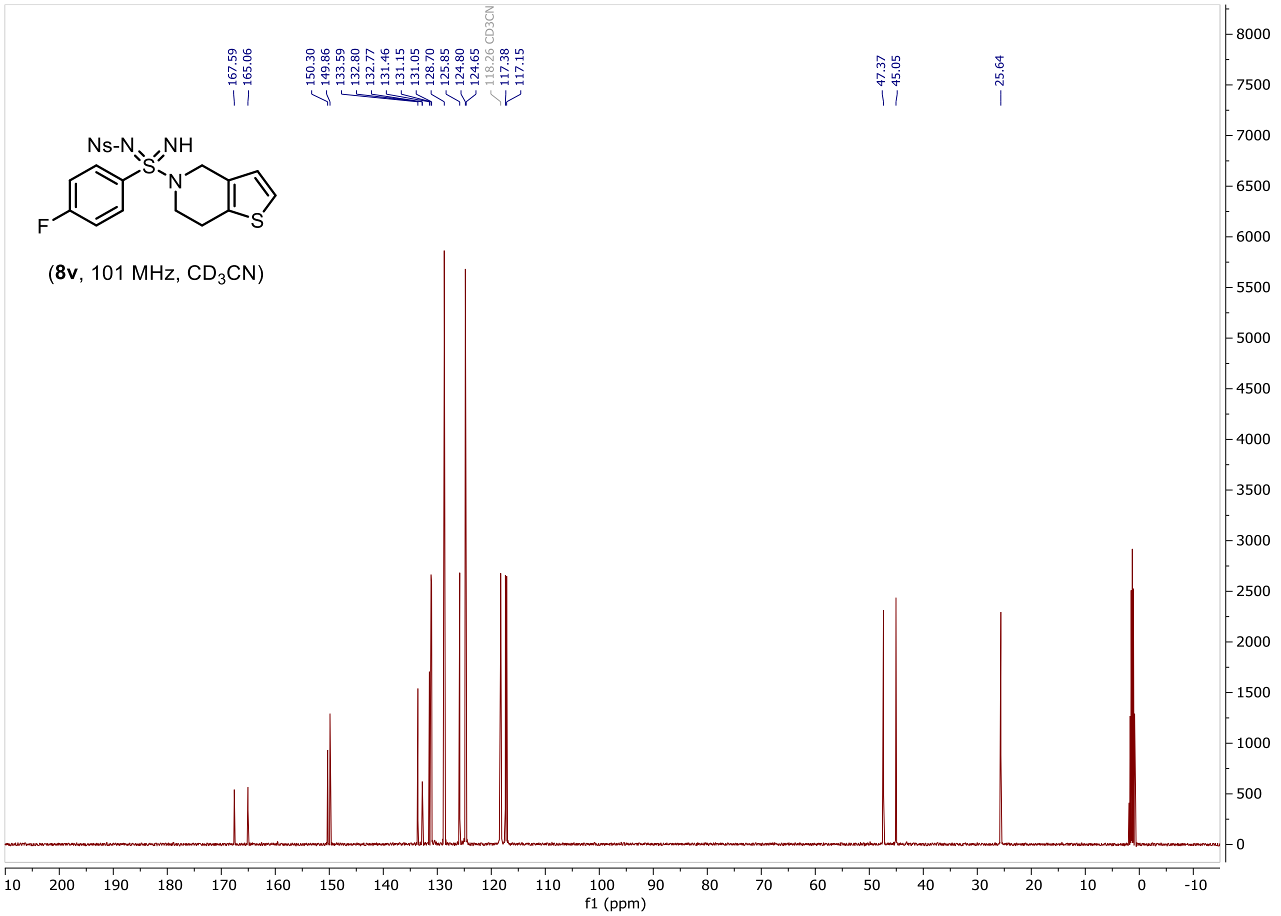
(8v, 101 MHz, CD3CN)

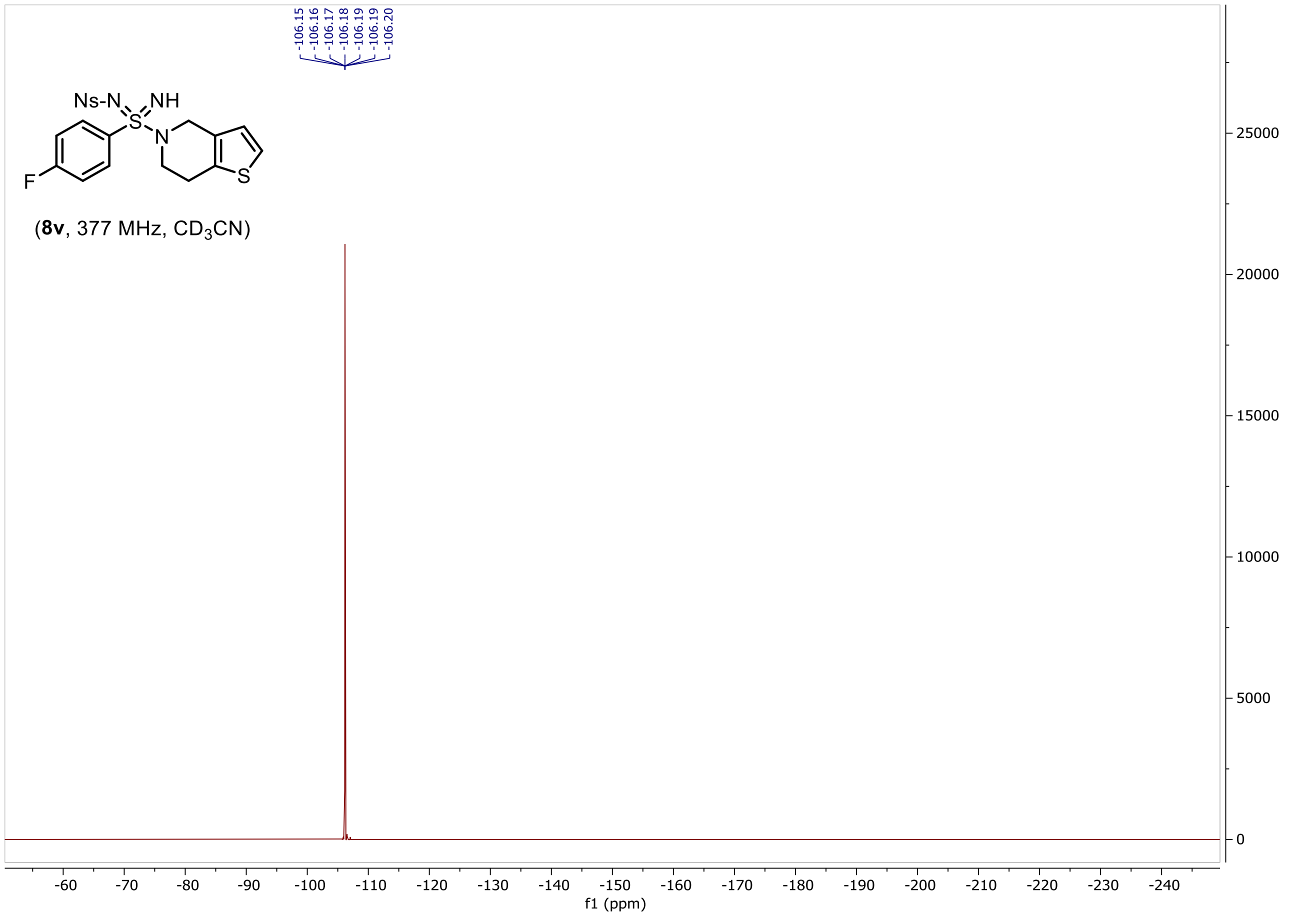

(**8v**, 377 MHz, CD_3CN)

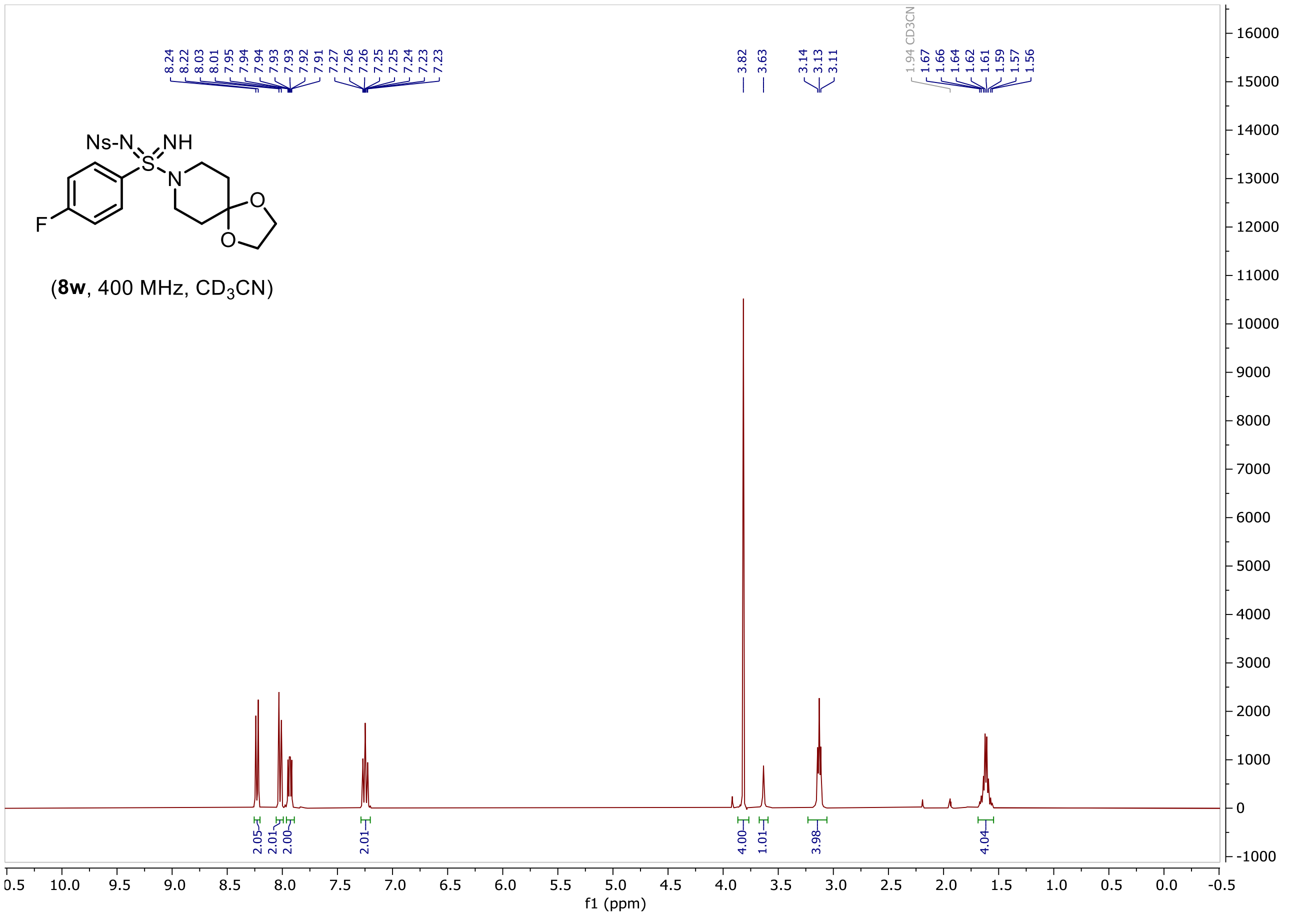

(**8w**, 400 MHz, CD_3CN)

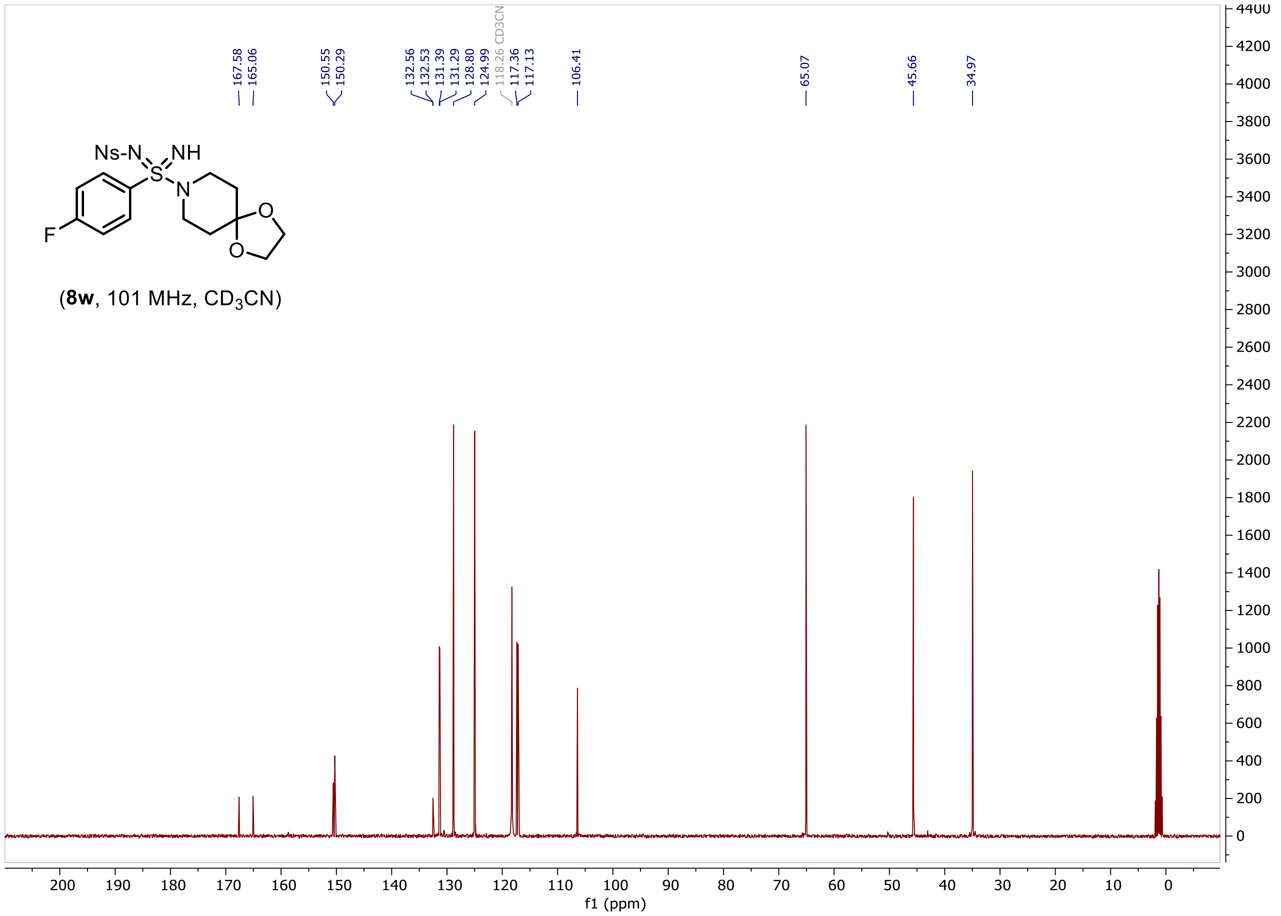

(**8w**, 101 MHz, CD_3CN)

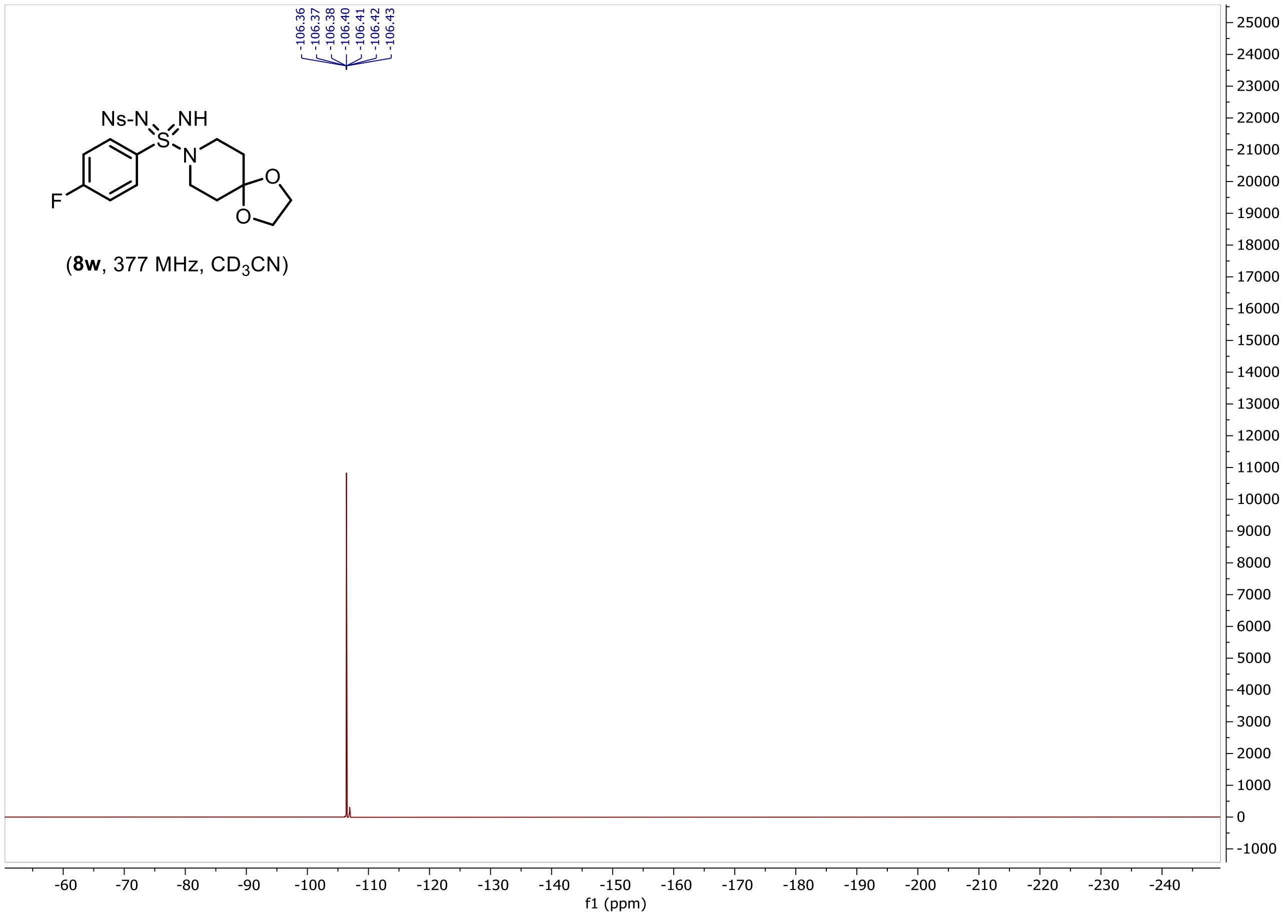

(**8w**, 377 MHz, CD_3CN)

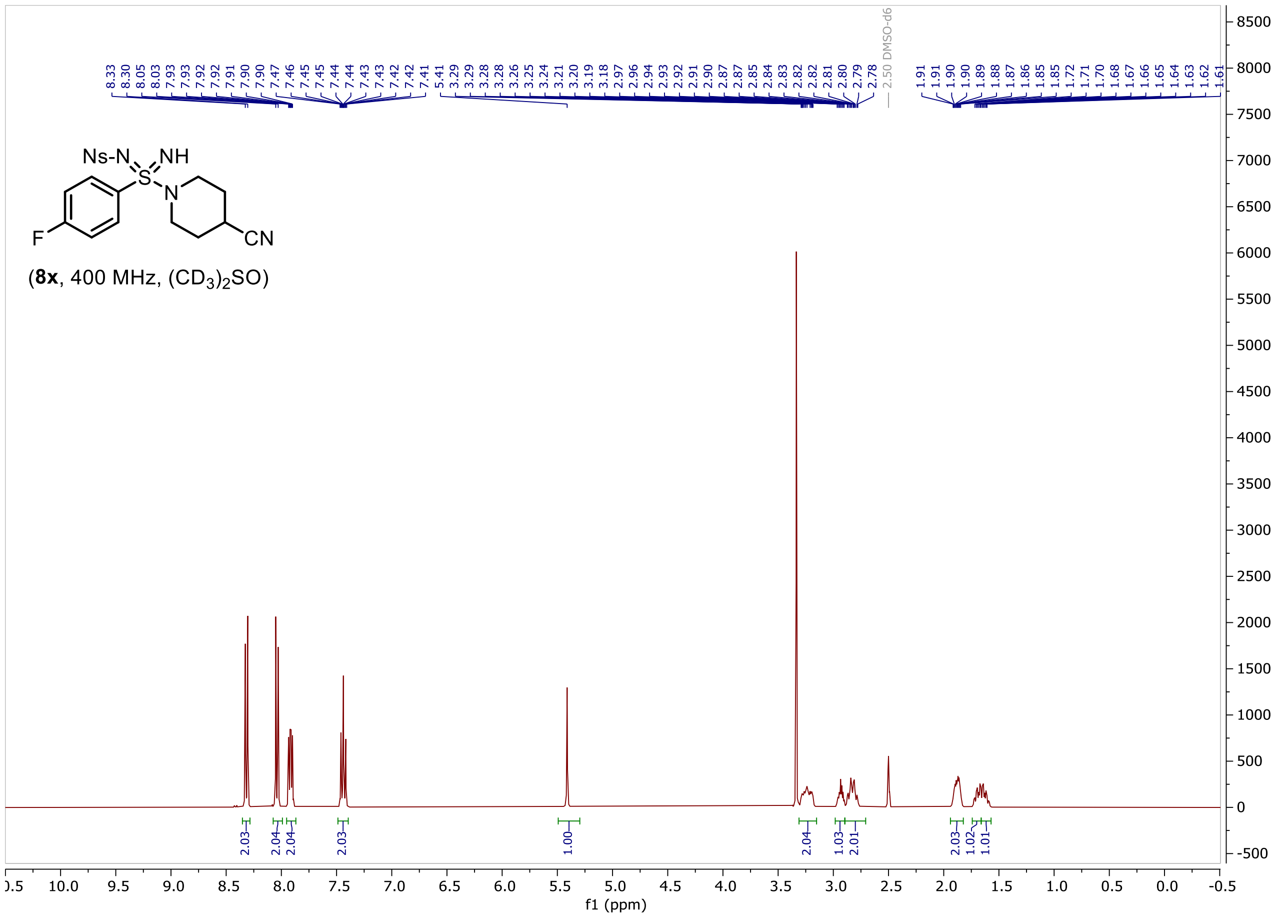
Ns-N
NH
S
N
F
CN
(8x, 400 MHz, (CD3)2SO)
f1 (ppm)

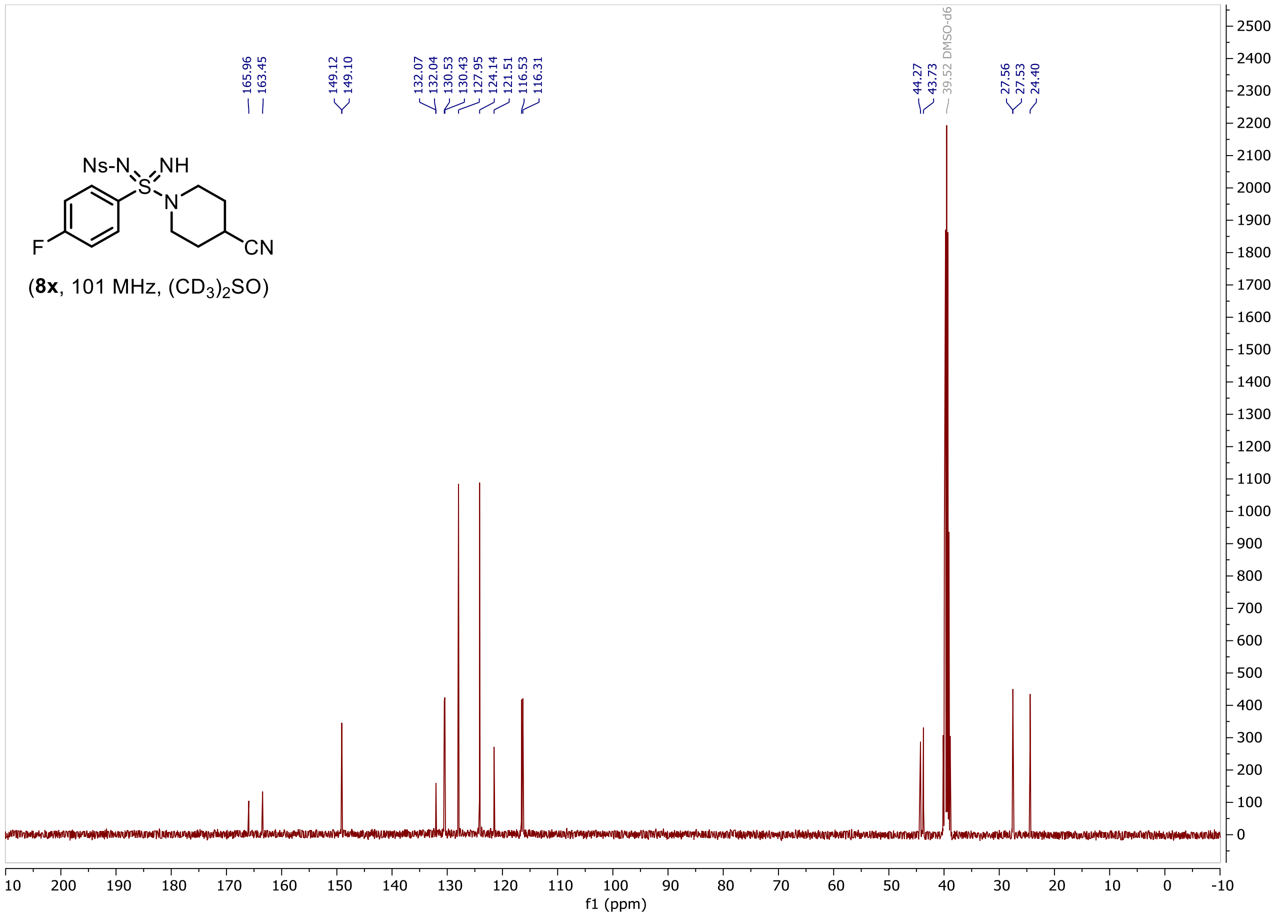
Ns-N
NH
S
N
F
CN
(8x, 101 MHz, (CD3)2SO)
f1 (ppm)

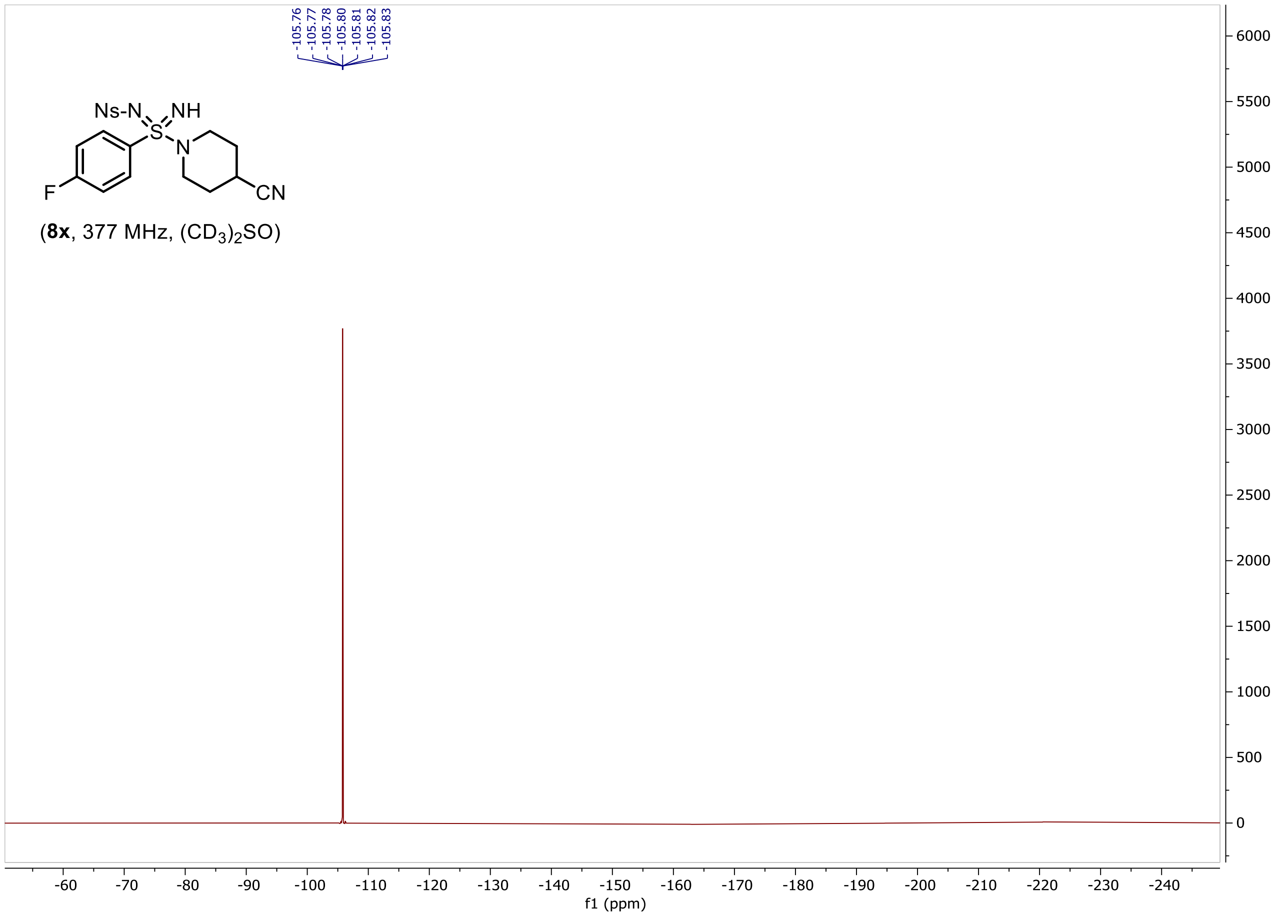
-105.76
-105.77
-105.78
-105.80
-105.81
-105.82
-105.83
Ns-N
NH
S
N
F
CN
(8x, 377 MHz, (CD3)2SO)
f1 (ppm)

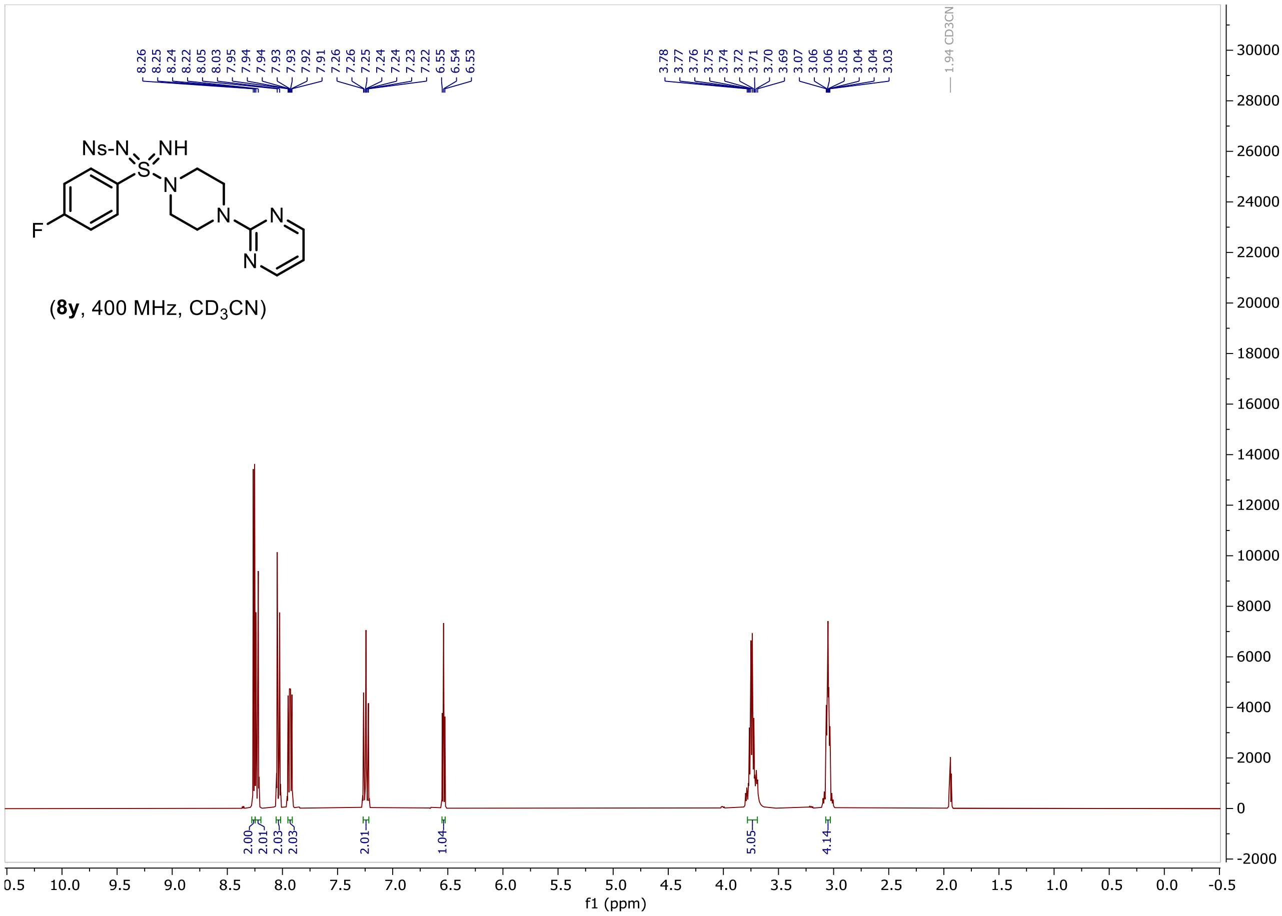
8.26
8.25
8.24
8.22
8.05
8.03
7.95
7.94
7.93
7.93
7.92
7.91
7.26
7.26
7.25
7.24
7.24
7.23
7.22
6.55
6.54
6.53
3.78
3.77
3.76
3.75
3.74
3.72
3.71
3.70
3.69
3.07
3.06
3.06
3.05
3.04
3.04
3.03
1.94 CD3CN
Ns-N
NH
S
N
N
N
N
F
(8y, 400 MHz, CD3CN)
2.00
2.01
2.03
2.03
2.01
1.04
5.05
4.14
f1 (ppm)

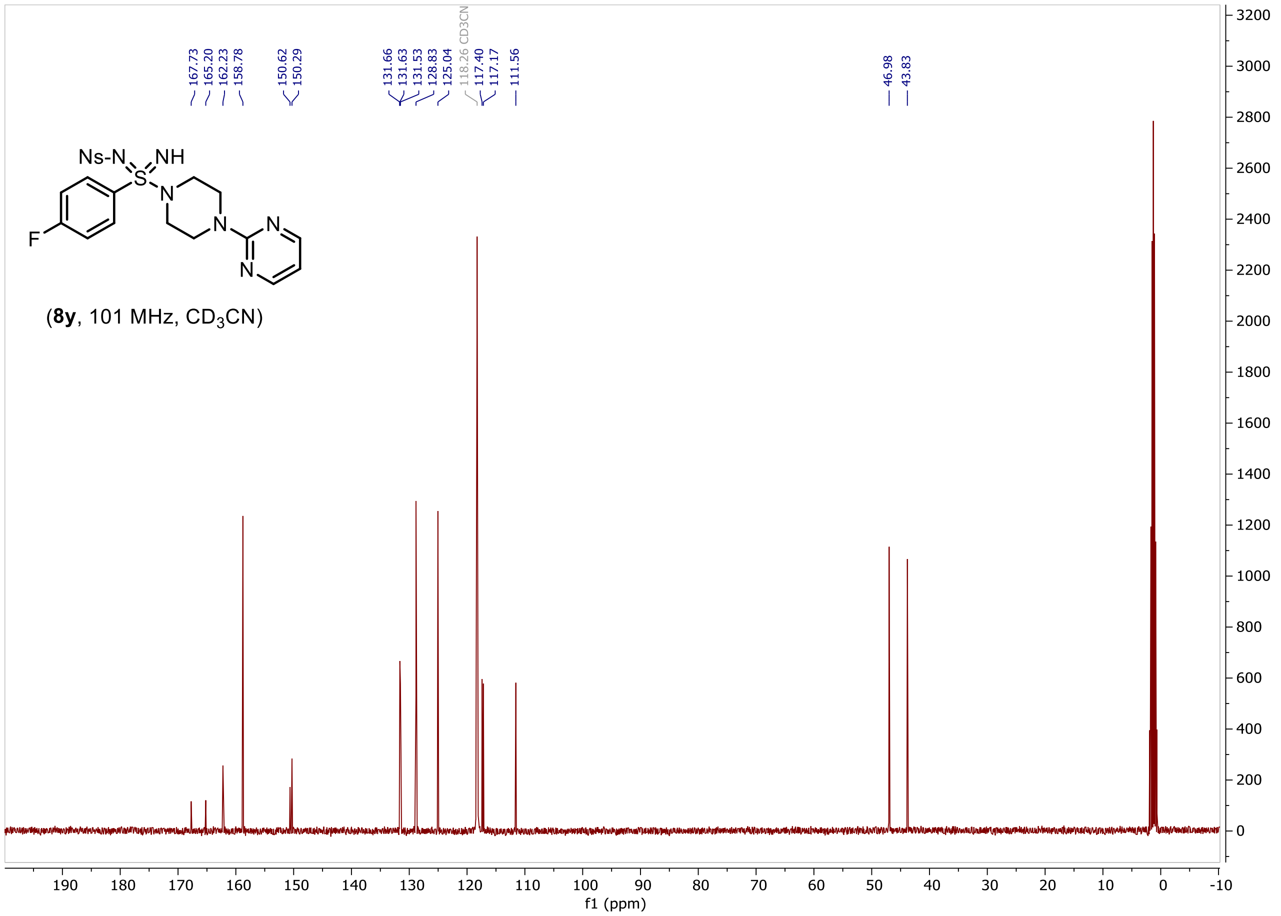
167.73
165.20
162.23
158.78
150.62
150.29
131.66
131.63
131.53
128.83
125.04
118.26 CD3CN
117.40
117.17
111.56
46.98
43.83
Ns-N
NH
S
N
N
N
N
F
(8y, 101 MHz, CD3CN)
f1 (ppm)

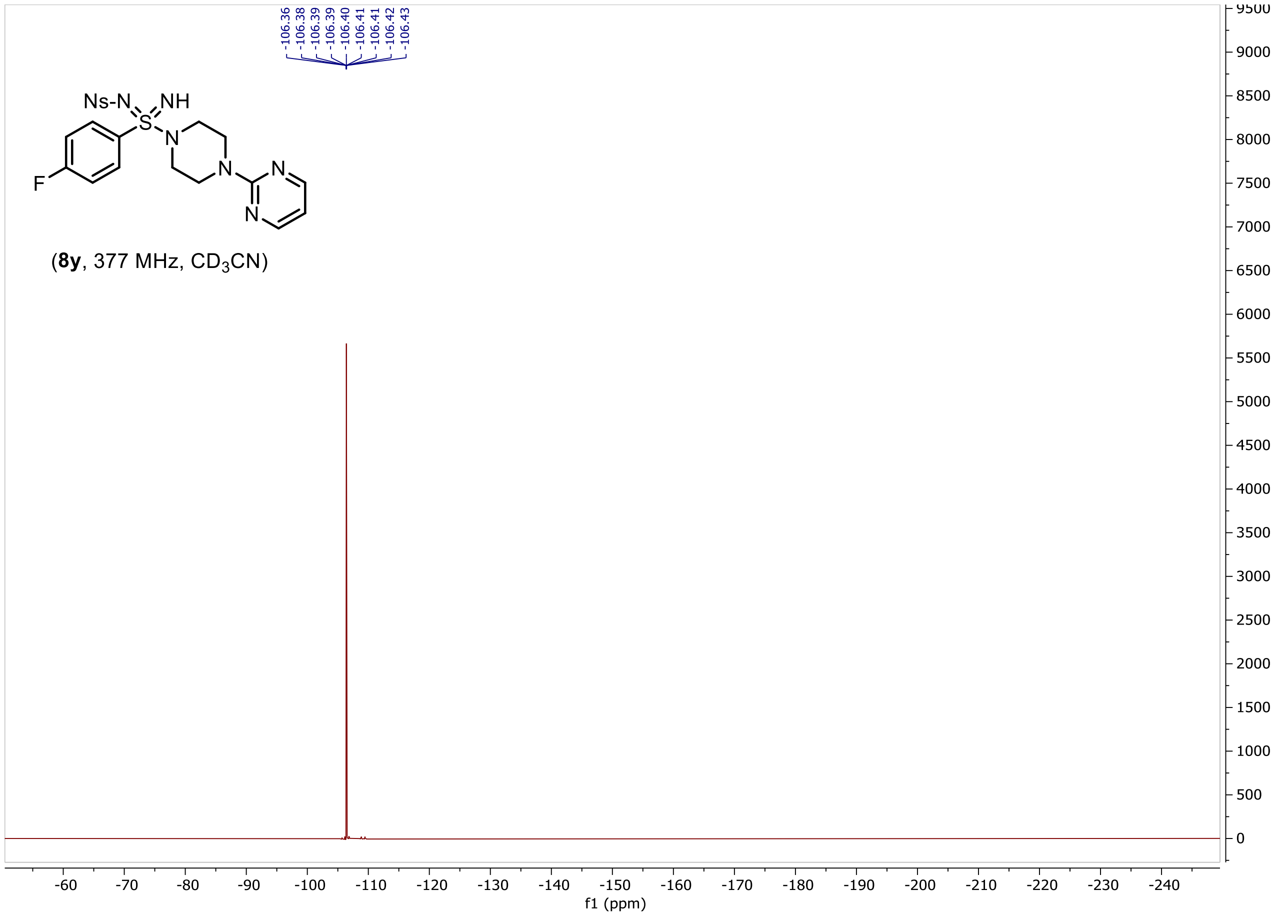
-106.36
-106.38
-106.39
-106.39
-106.40
-106.41
-106.41
-106.42
-106.43
Ns-N
NH
S
N
N
N
N
F
(8y, 377 MHz, CD3CN)
f1 (ppm)

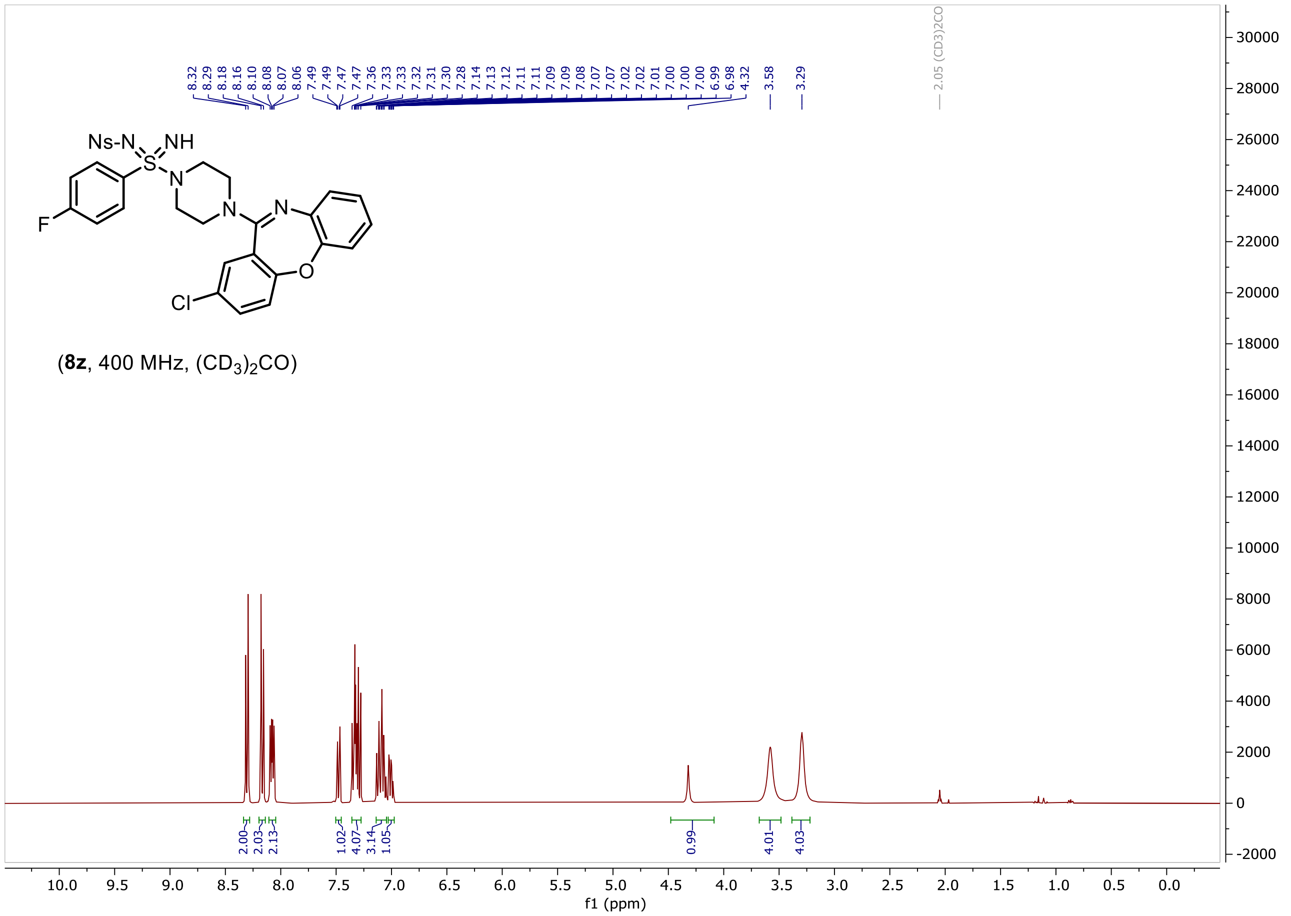

8.32 8.29 8.18 8.16 8.10 8.08 8.07 8.06 7.49 7.49 7.47 7.47 7.36 7.33 7.33 7.32 7.31 7.30 7.28 7.14 7.13 7.12 7.11 7.11 7.09 7.09 7.08 7.07 7.07 7.02 7.02 7.01 7.00 7.00 6.99 6.98 4.32 3.58 3.29
2.05 (CD3)2CO
Ns-N NH S N N N O Cl F
(8z, 400 MHz, (CD3)2CO)
2.00 2.03 2.13 1.02 4.07 3.14 1.05 0.99 4.01 4.03
f1 (ppm)

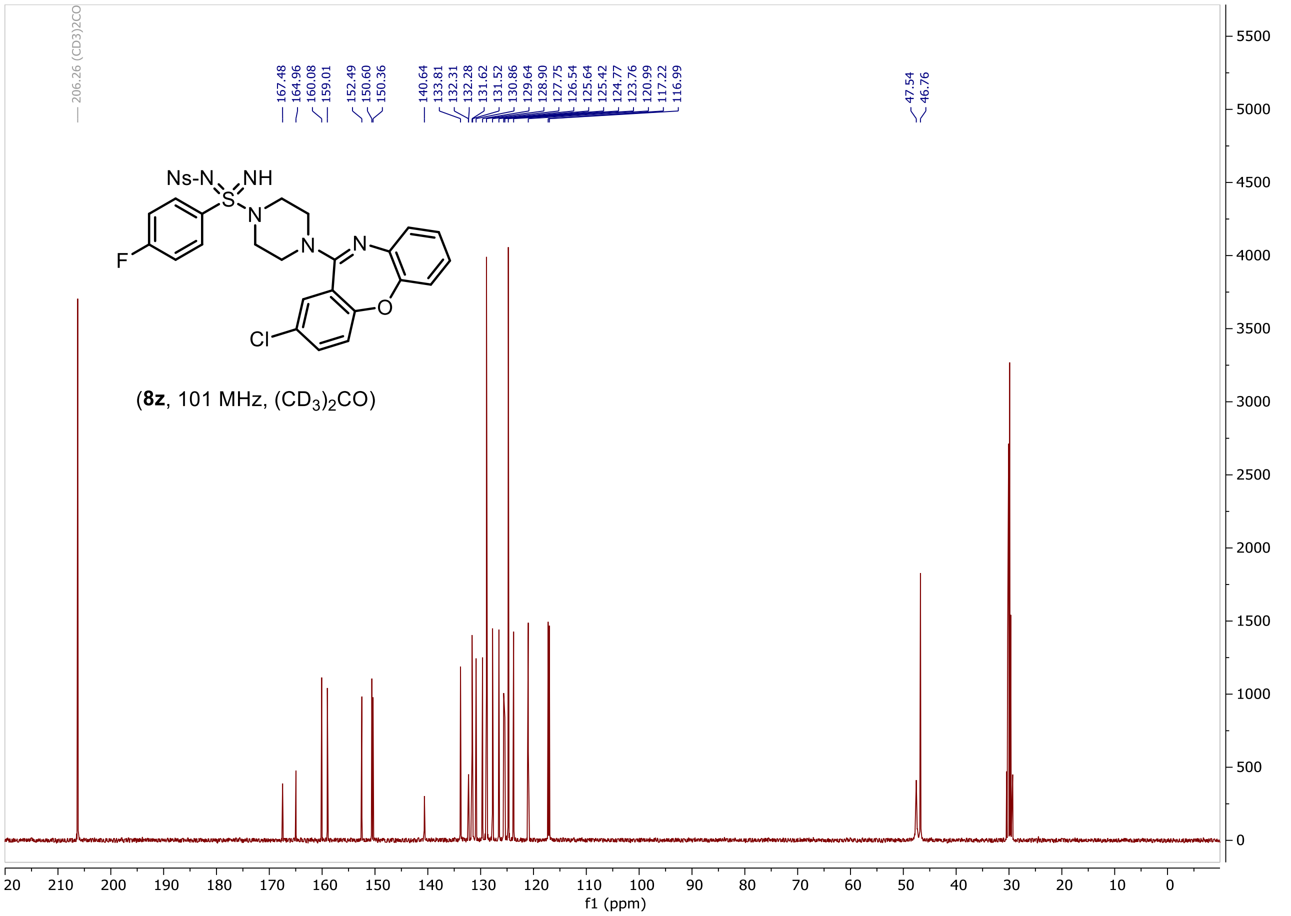

206.26 (CD3)2CO
167.48 164.96 160.08 159.01 152.49 150.60 150.36 140.64 133.81 132.31 132.28 131.62 131.52 130.86 129.64 128.90 127.75 126.54 125.64 125.42 124.77 123.76 120.99 117.22 116.99 47.54 46.76
Ns-N NH S N N N O Cl F
(8z, 101 MHz, (CD3)2CO)
f1 (ppm)

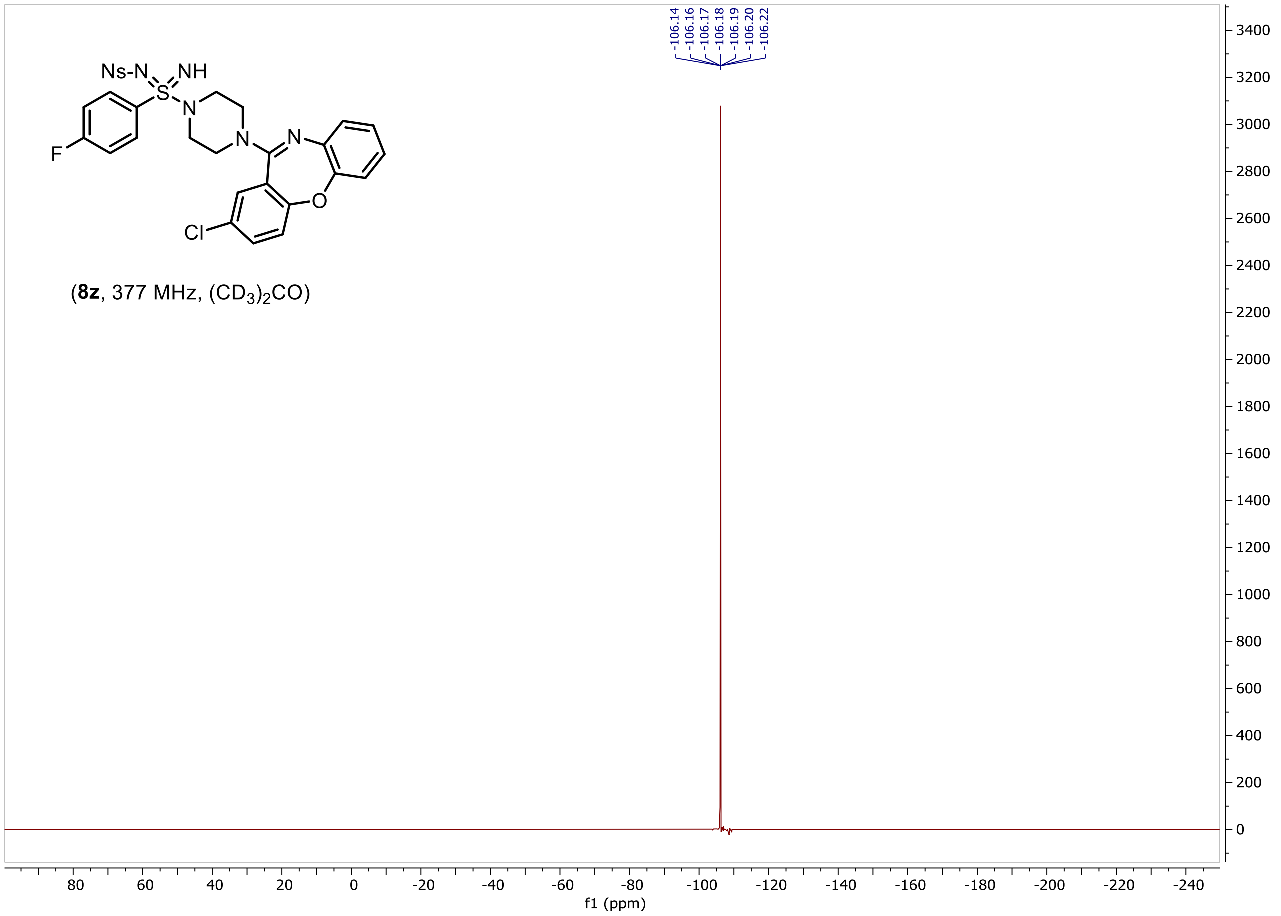

(**8z**, 377 MHz, $(CD_3)_2CO$)

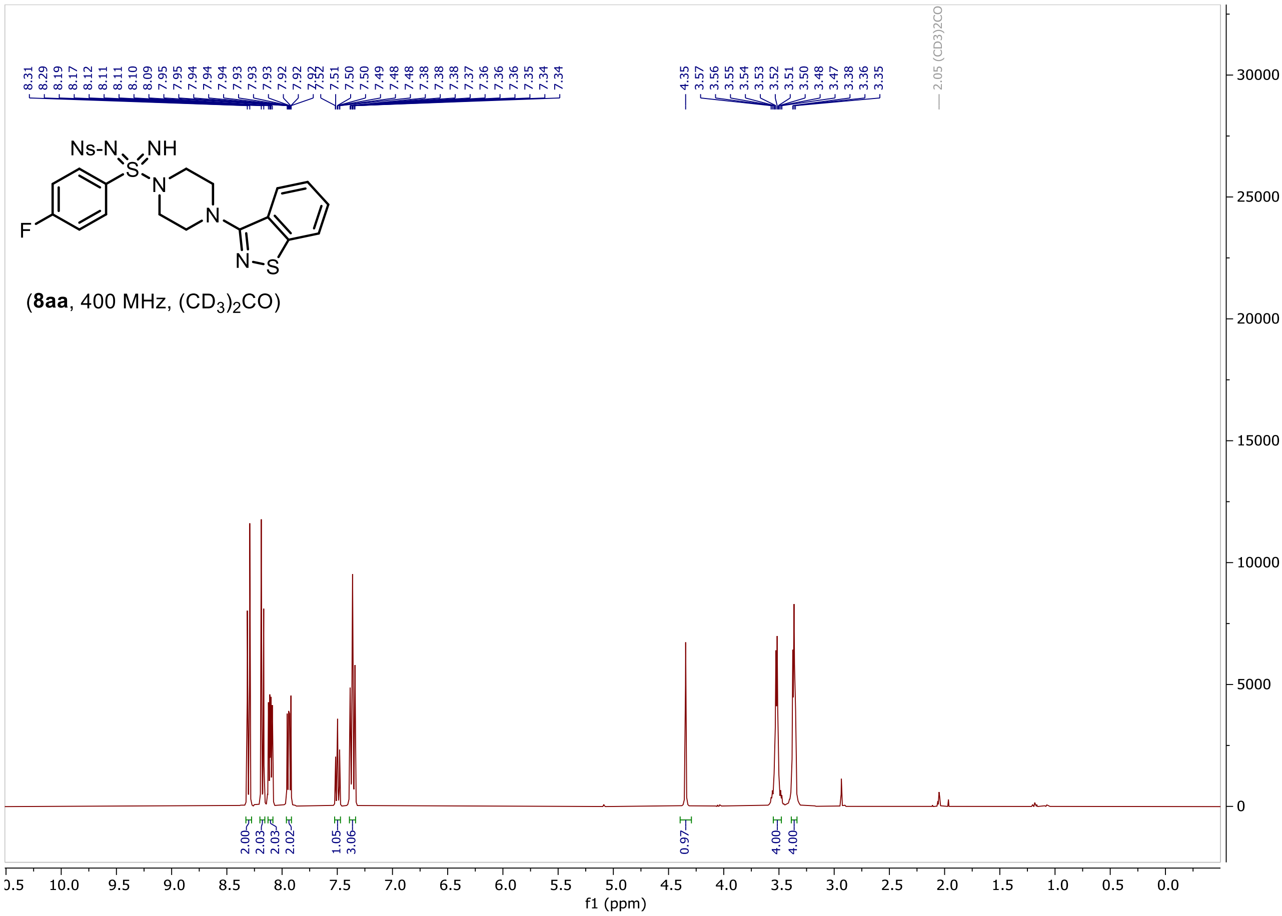

(**8aa**, 400 MHz, $(CD_3)_2CO$)

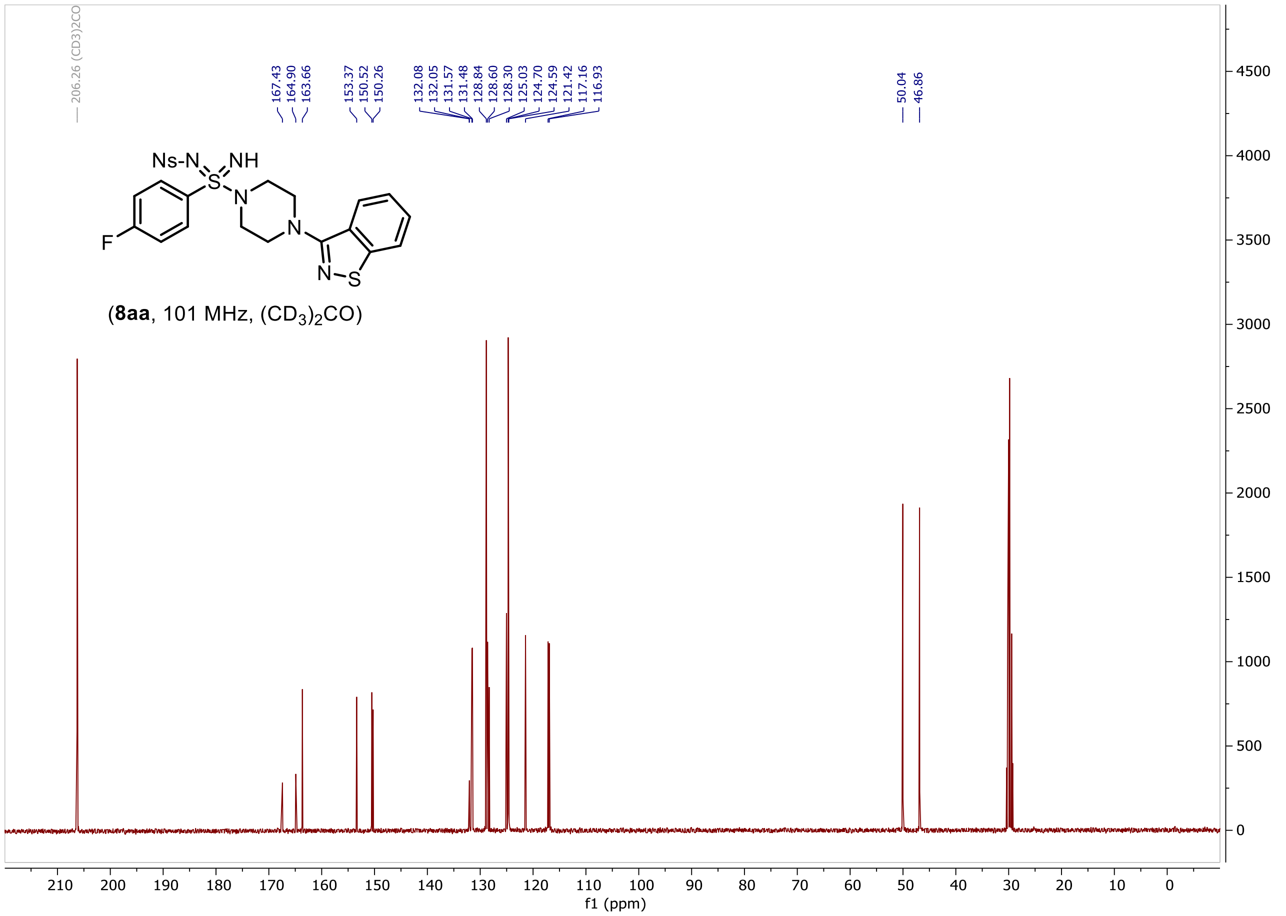
206.26 (CD3)2CO
167.43
164.90
163.66
153.37
150.52
150.26
132.08
132.05
131.57
131.48
128.84
128.60
128.30
125.03
124.70
124.59
121.42
117.16
116.93
50.04
46.86
Ns-N
NH
S
N
N
F
N
S
(**8aa**, 101 MHz, $(CD_3)_2CO$)
f1 (ppm)

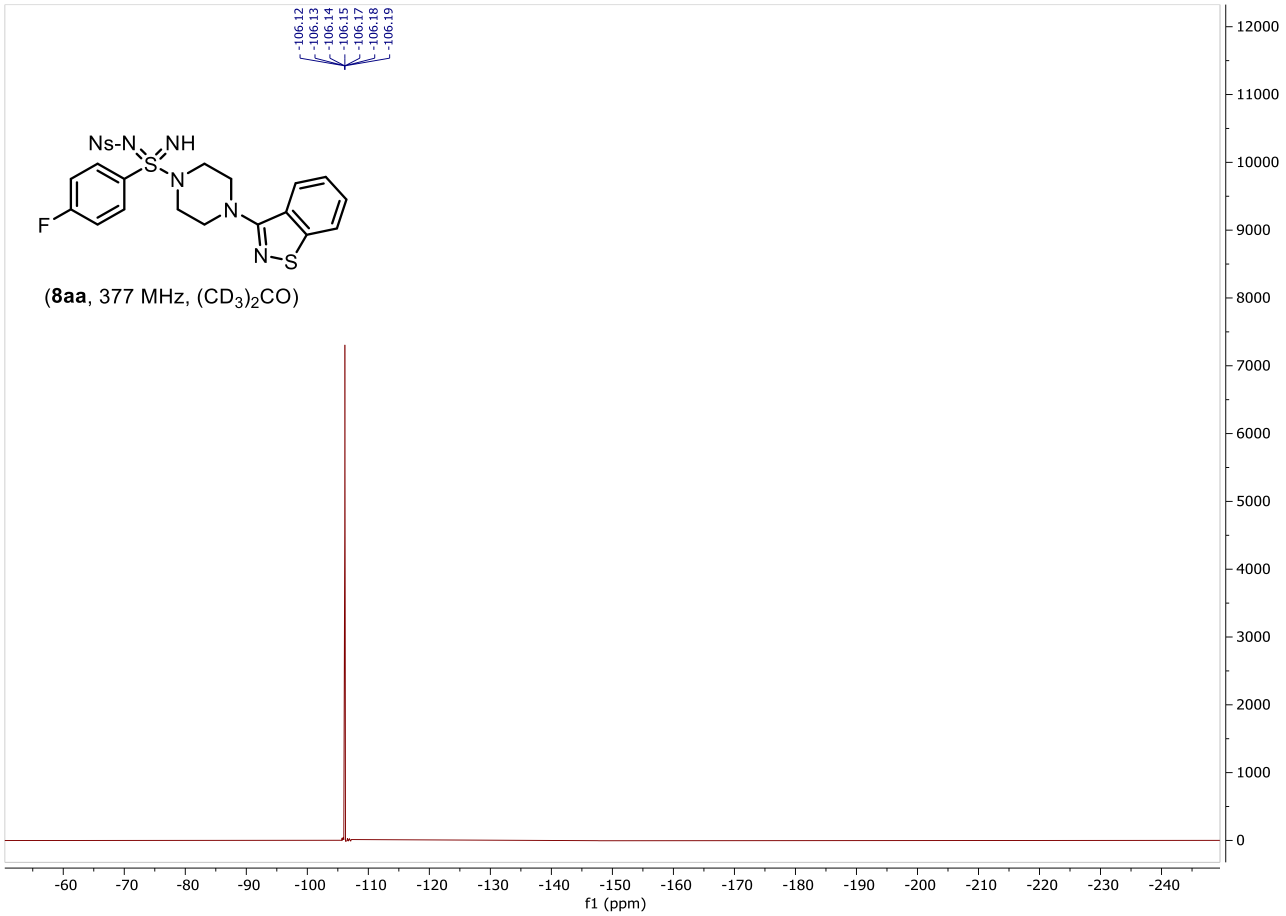
-106.12
-106.13
-106.14
-106.15
-106.17
-106.18
-106.19
Ns-N
NH
S
N
N
F
N
S
(**8aa**, 377 MHz, $(CD_3)_2CO$)
f1 (ppm)

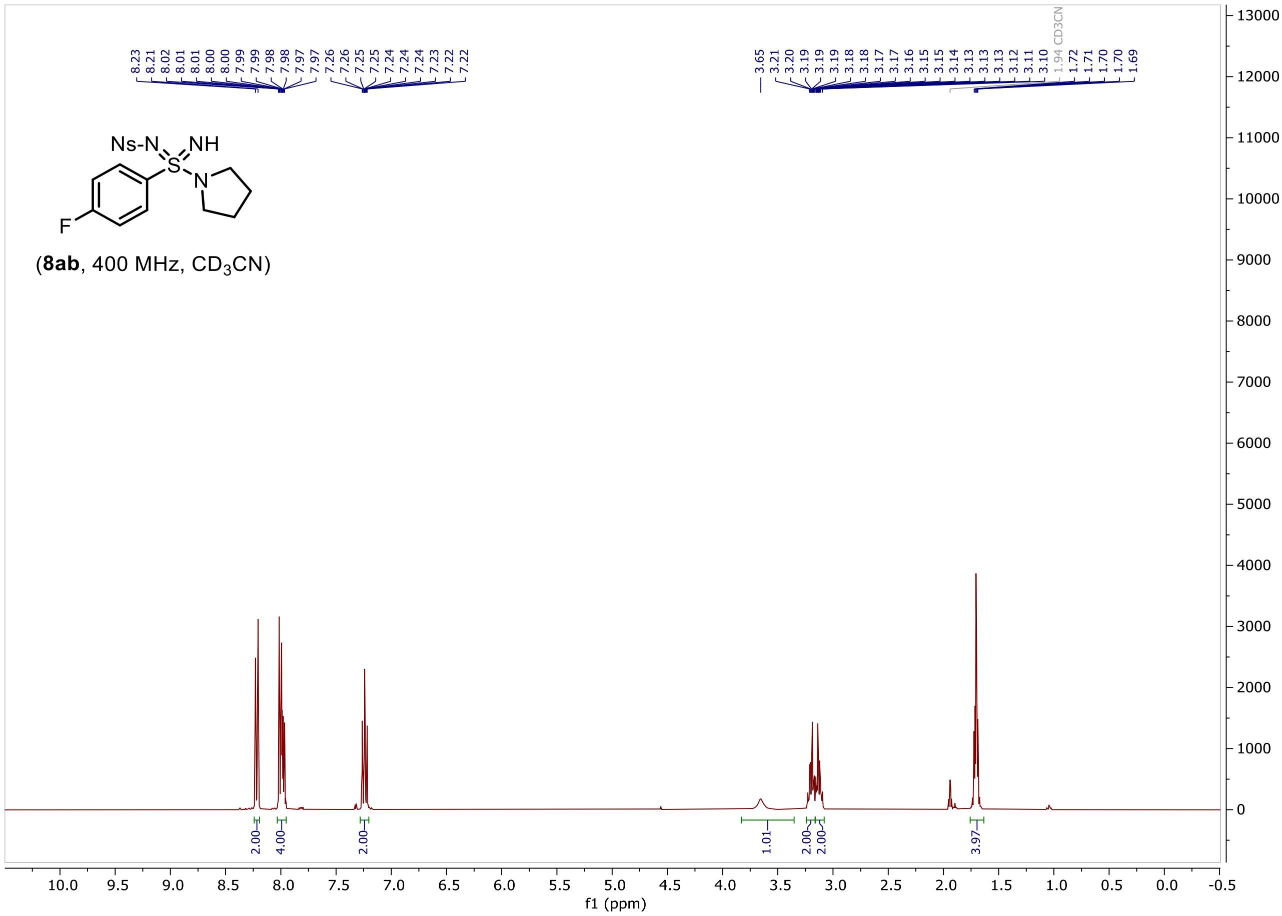

(**8ab**, 400 MHz, CD_3CN)

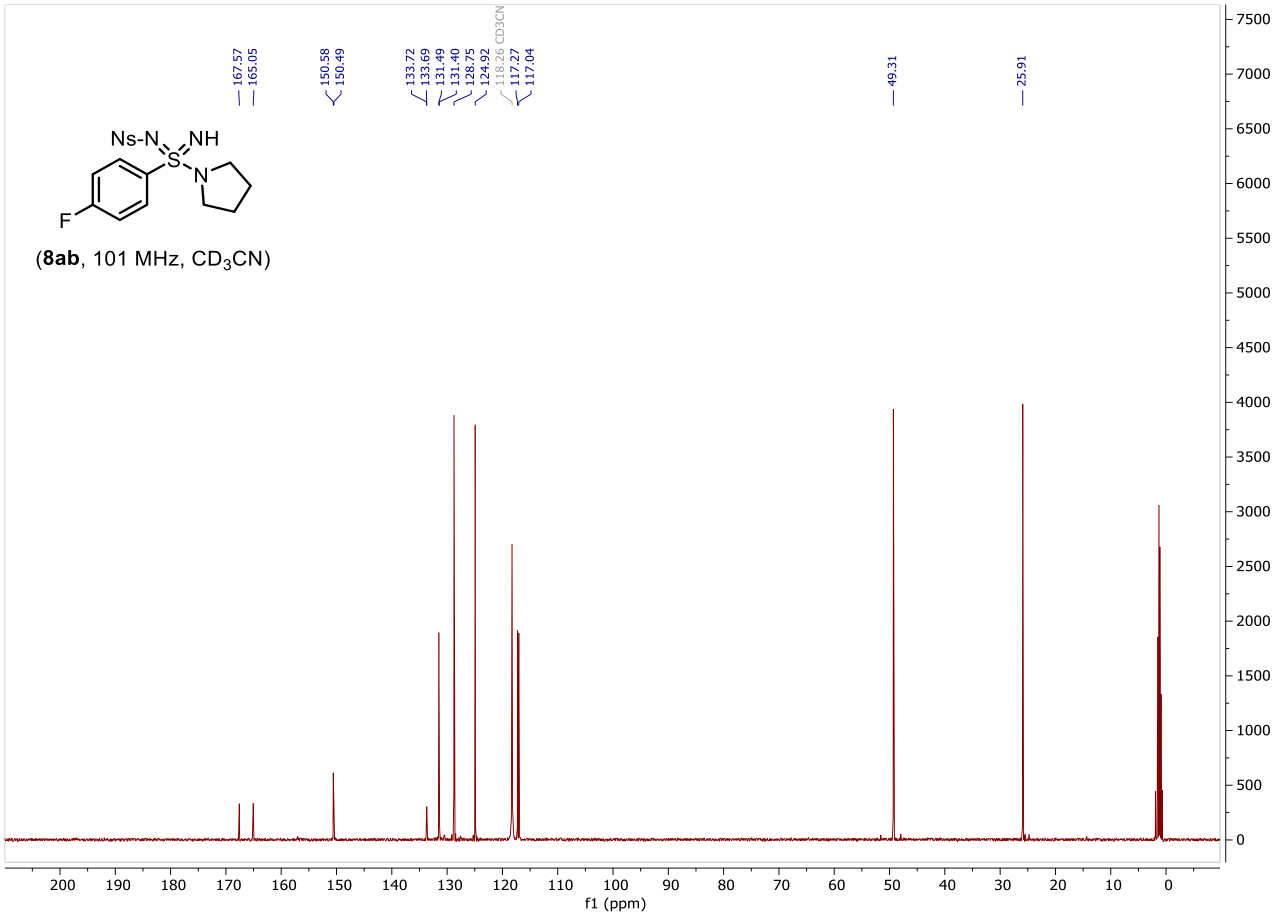

(**8ab**, 101 MHz, CD_3CN)

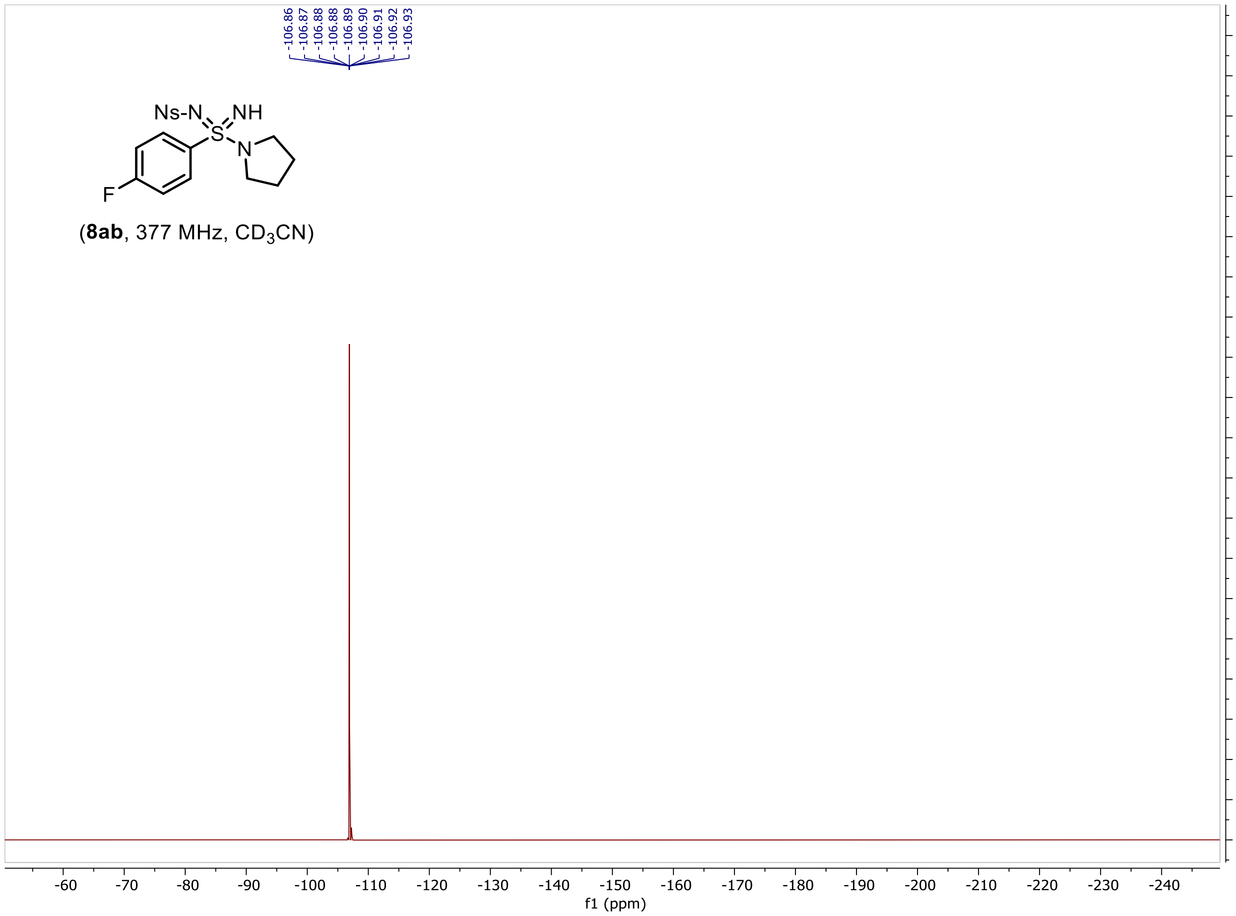
-106.86
-106.87
-106.88
-106.88
-106.89
-106.90
-106.91
-106.92
-106.93
Ns-N
NH
S
N
F
(8ab, 377 MHz, CD3CN)
f1 (ppm)

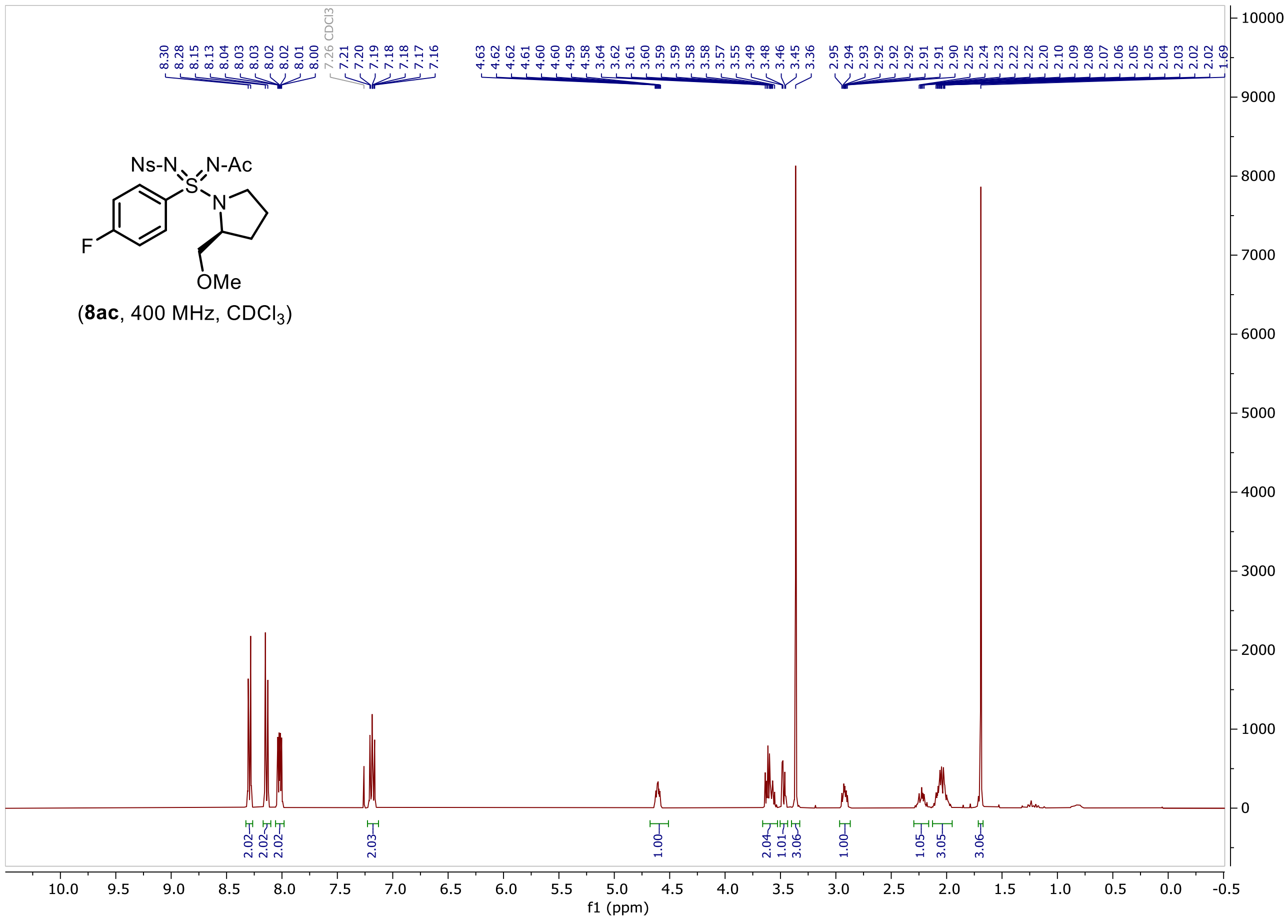
Ns-N
N-Ac
S
N
F
OMe
(8ac, 400 MHz, CDCl3)
f1 (ppm)

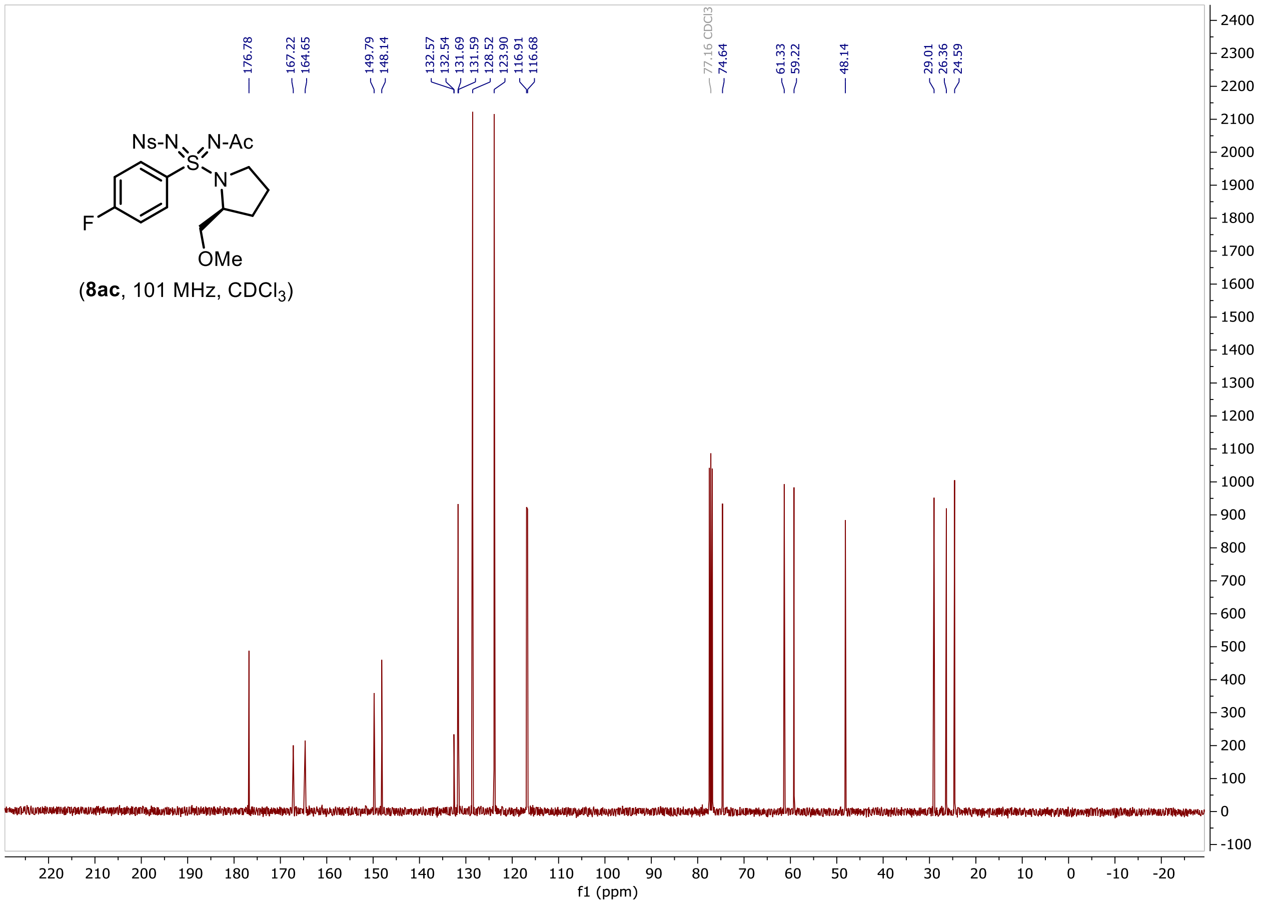

176.78
167.22
164.65
149.79
148.14
132.57
132.54
131.69
131.59
128.52
123.90
116.91
116.68
77.16 CDCl3
74.64
61.33
59.22
48.14
29.01
26.36
24.59
Ns-N
N-Ac
S
N
F
OMe
(8ac, 101 MHz, CDCl3)
f1 (ppm)

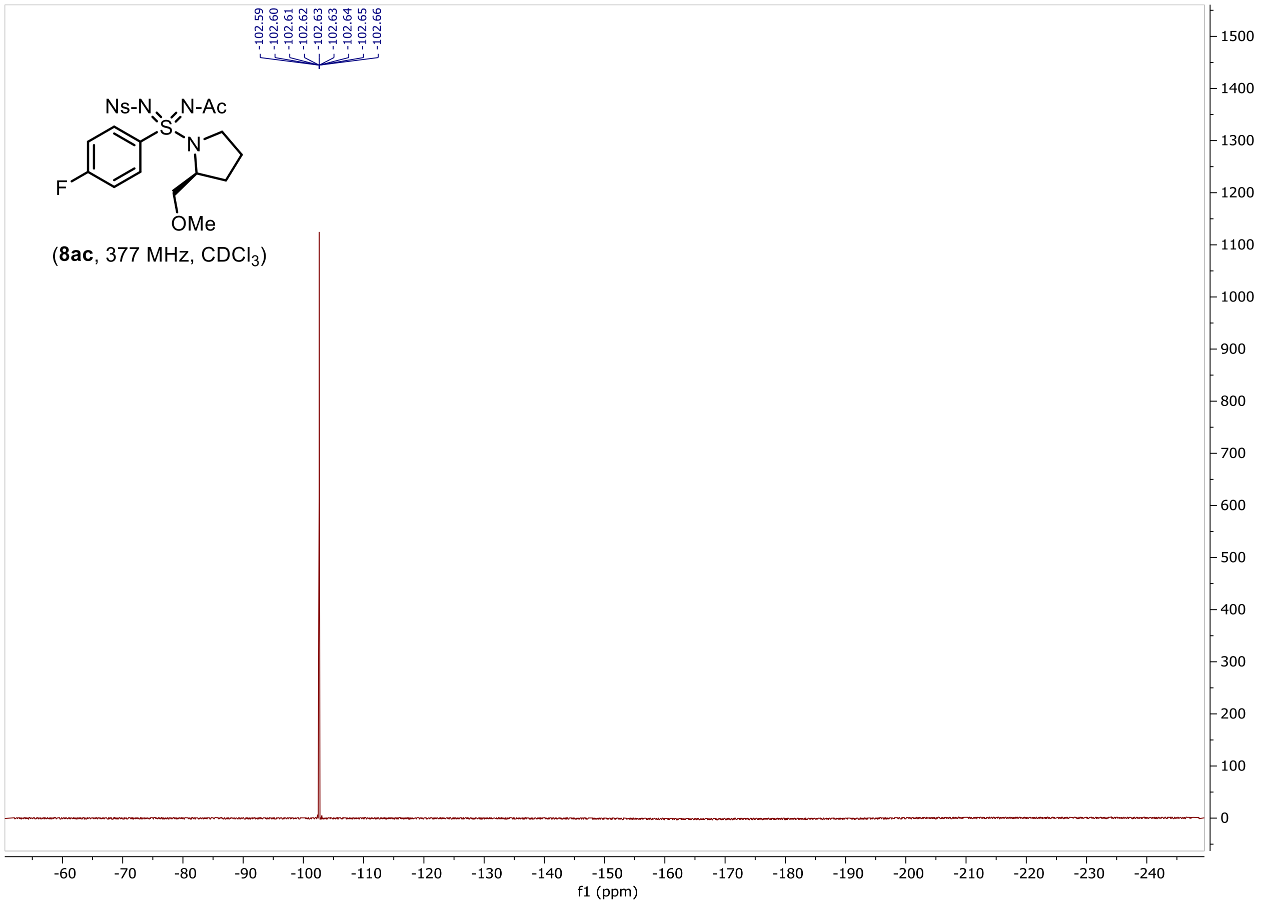

-102.59
-102.60
-102.61
-102.62
-102.63
-102.63
-102.64
-102.65
-102.66
Ns-N
N-Ac
S
N
F
OMe
(8ac, 377 MHz, CDCl3)
f1 (ppm)

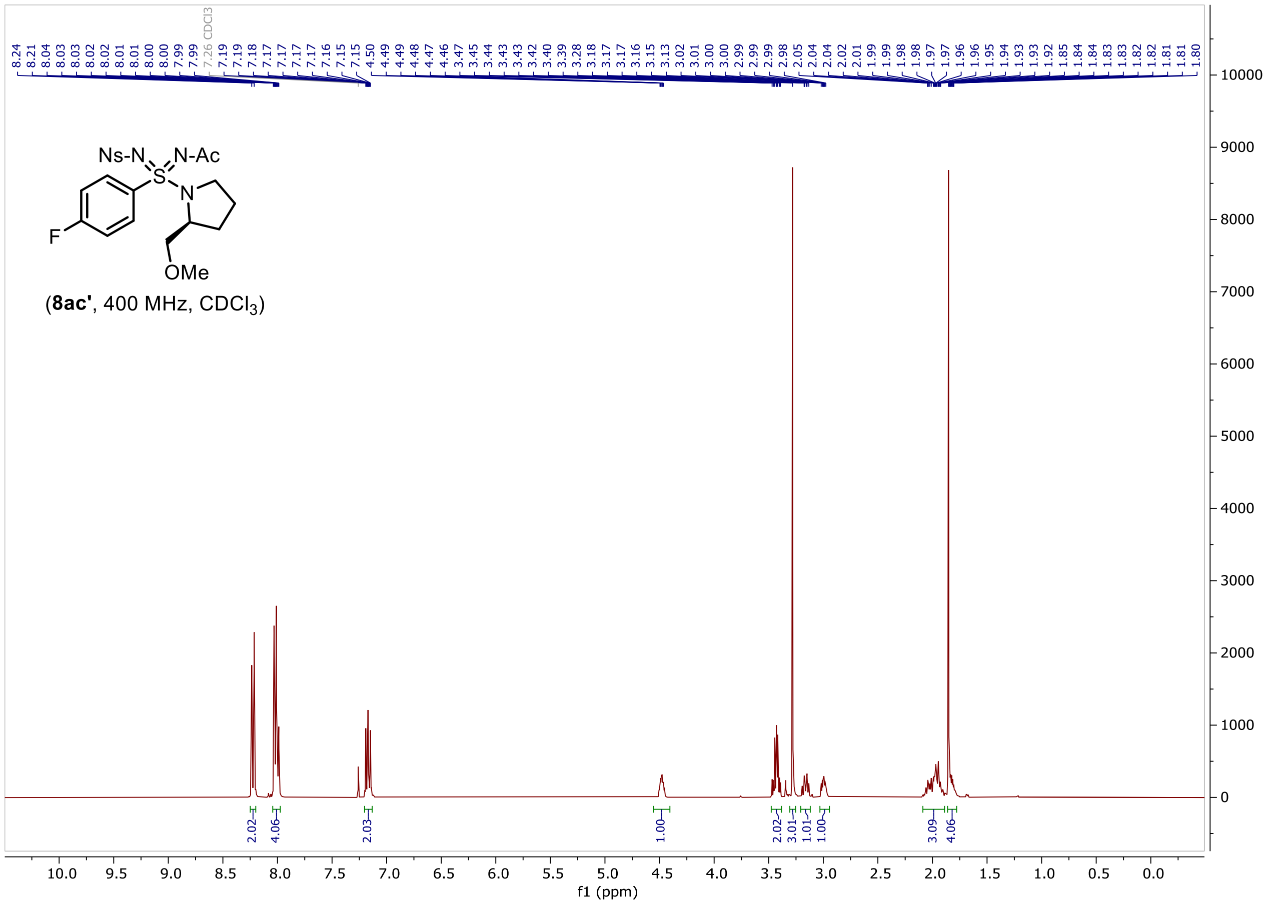

(**8ac'**, 400 MHz, $CDCl_3$)

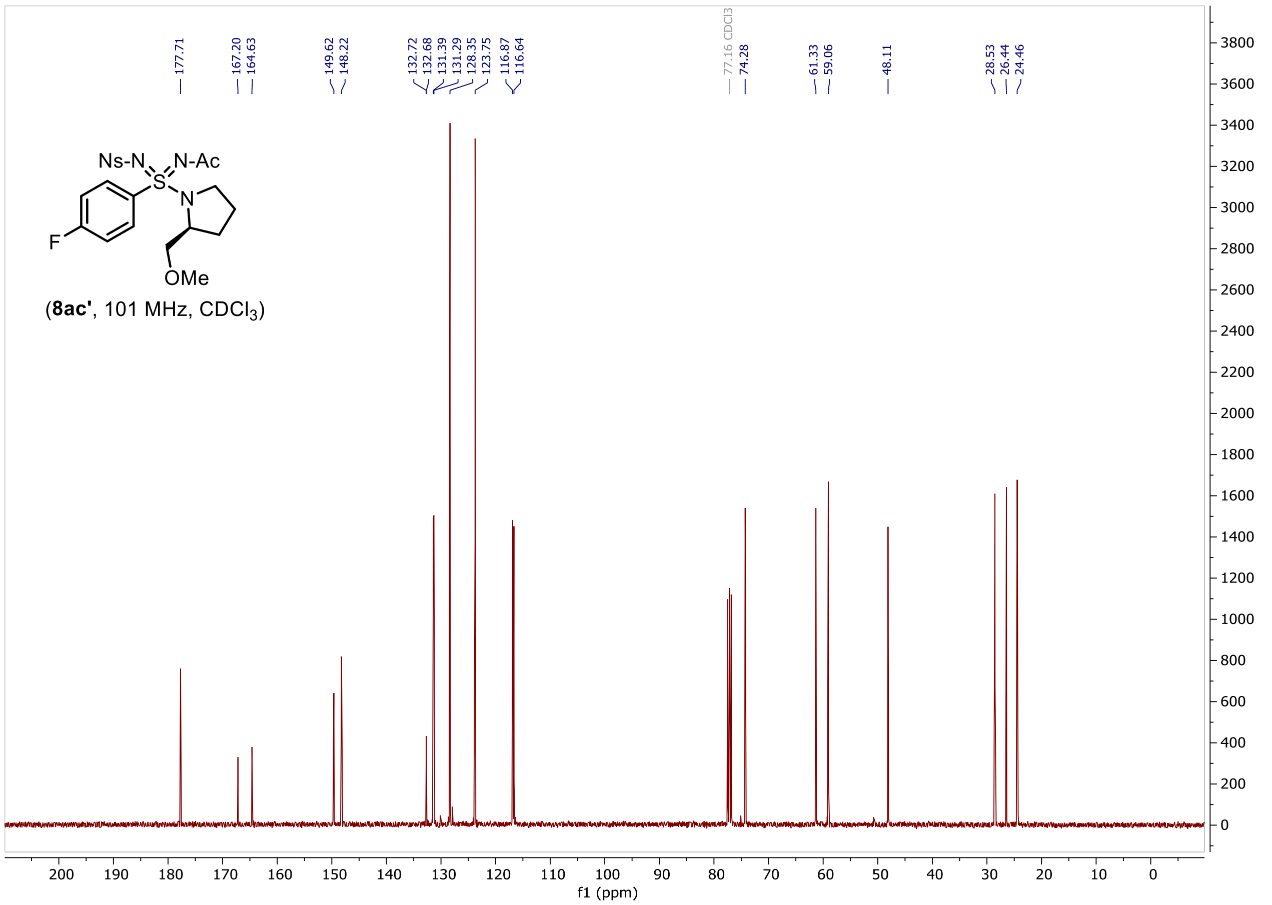

(**8ac'**, 101 MHz, $CDCl_3$)

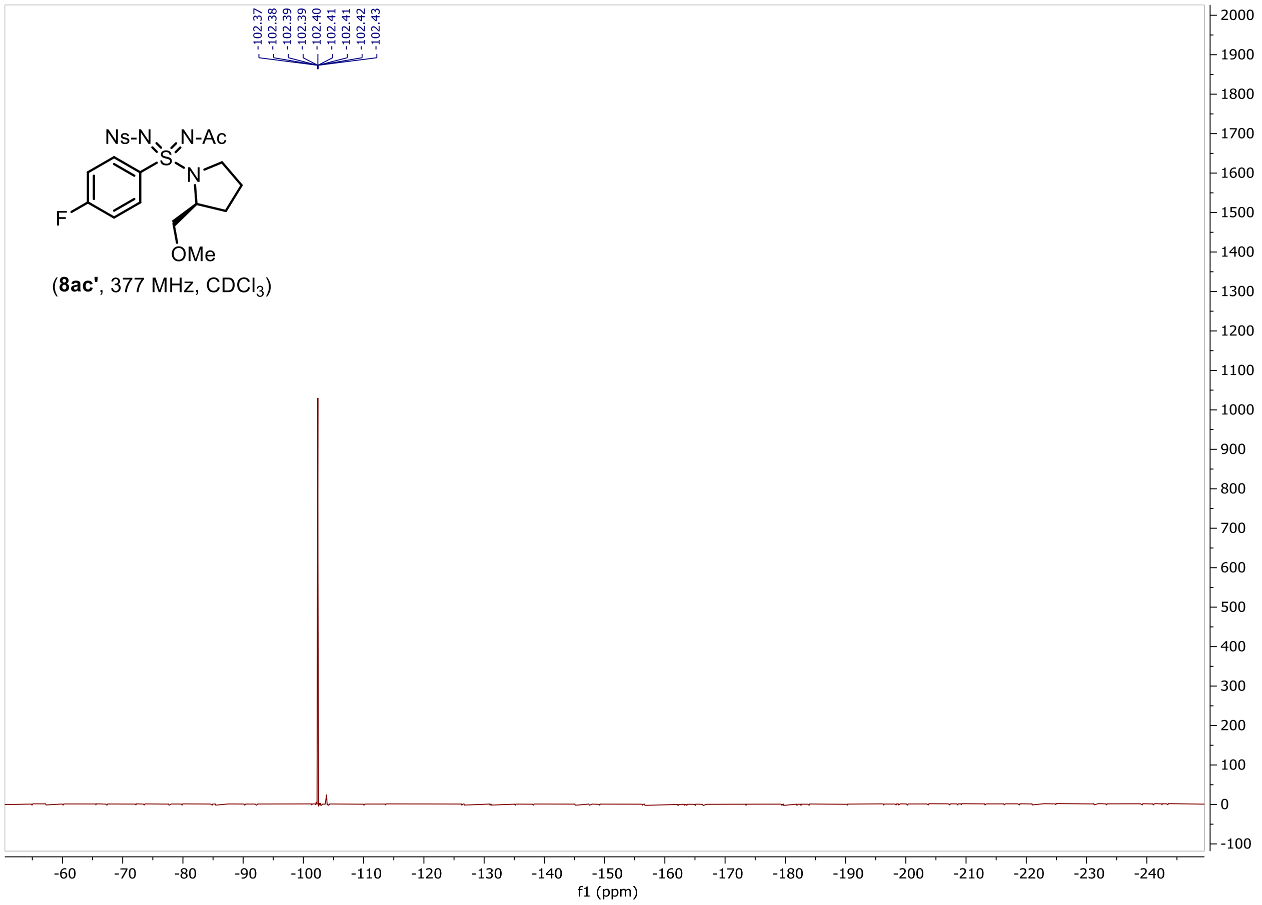
-102.37
-102.38
-102.39
-102.39
-102.40
-102.41
-102.41
-102.42
-102.43
Ns-N
N-Ac
S
N
F
OMe
(8ac', 377 MHz, CDCl3)
f1 (ppm)

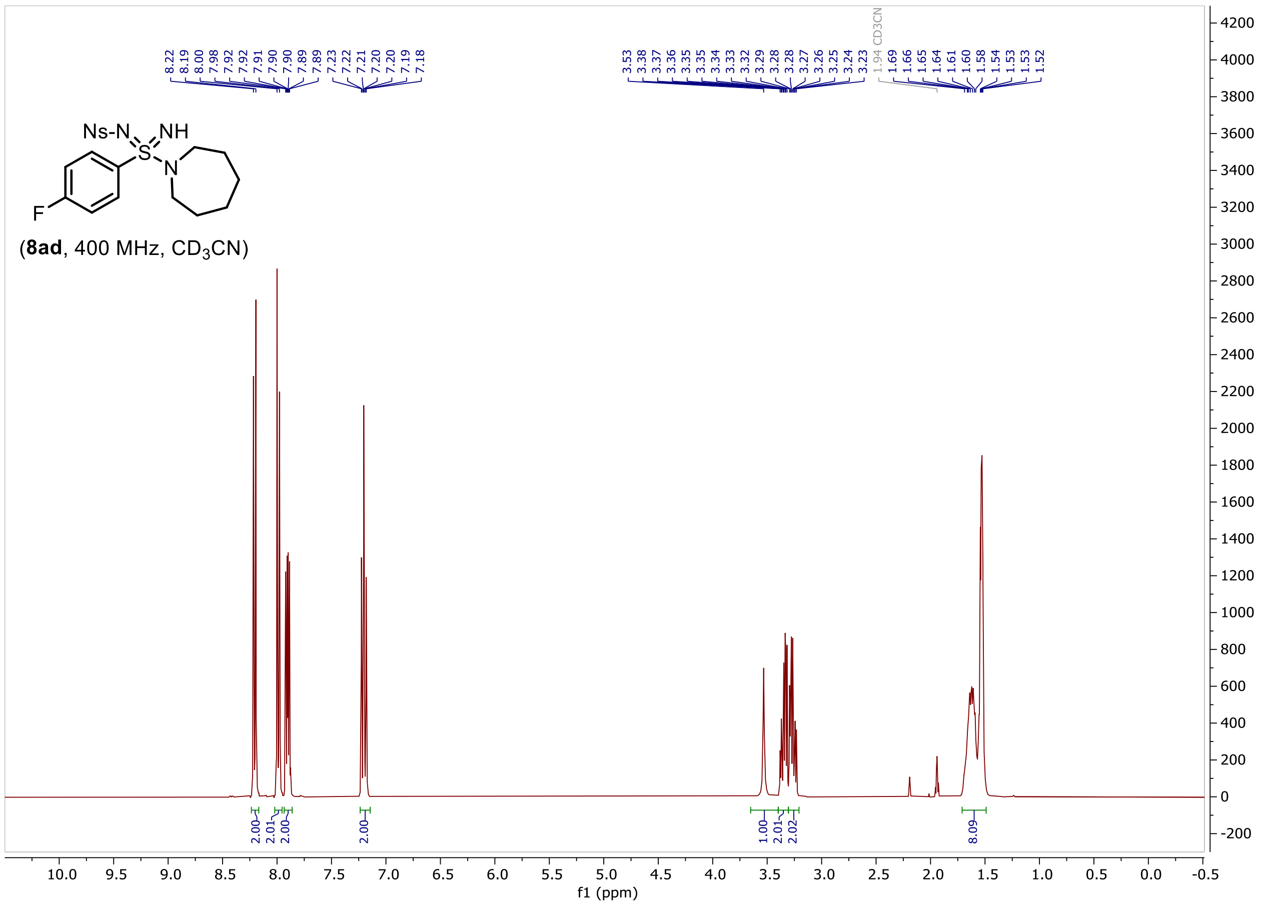
8.22
8.19
8.00
7.98
7.92
7.92
7.91
7.90
7.90
7.89
7.23
7.22
7.21
7.21
7.20
7.20
7.19
7.18
3.53
3.38
3.37
3.36
3.35
3.35
3.34
3.33
3.32
3.29
3.28
3.28
3.27
3.26
3.25
3.24
3.23
1.94 CD3CN
1.69
1.66
1.65
1.64
1.61
1.60
1.58
1.54
1.53
1.53
1.52
Ns-N
NH
S
N
F
(8ad, 400 MHz, CD3CN)
2.00
2.01
2.00
2.00
1.00
2.01
2.02
8.09
f1 (ppm)

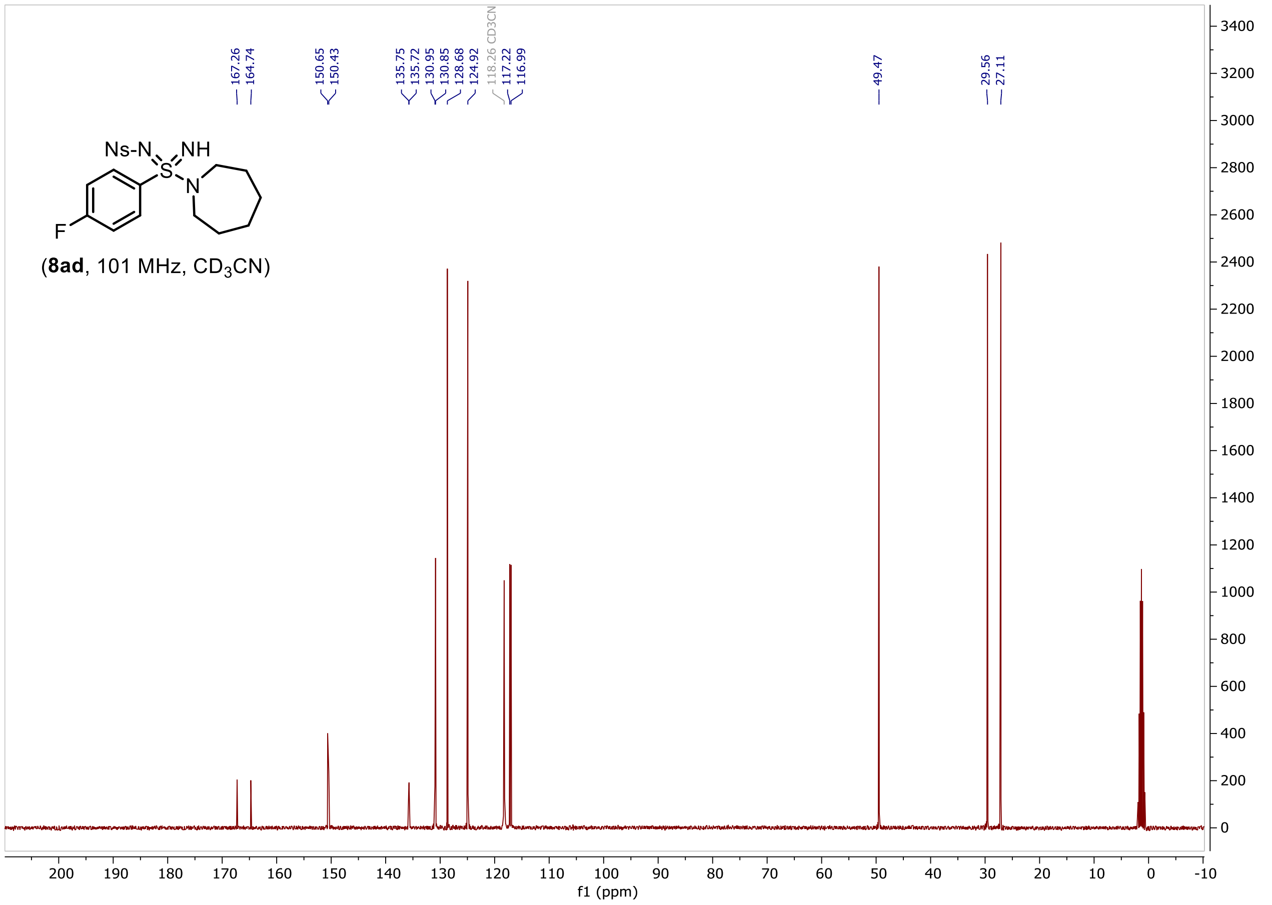
Ns-N
NH
S
N
F
(**8ad**, 101 MHz, CD_3CN)
167.26
164.74
150.65
150.43
135.75
135.72
130.95
130.85
128.68
124.92
118.26 CD3CN
117.22
116.99
49.47
29.56
27.11
f1 (ppm)

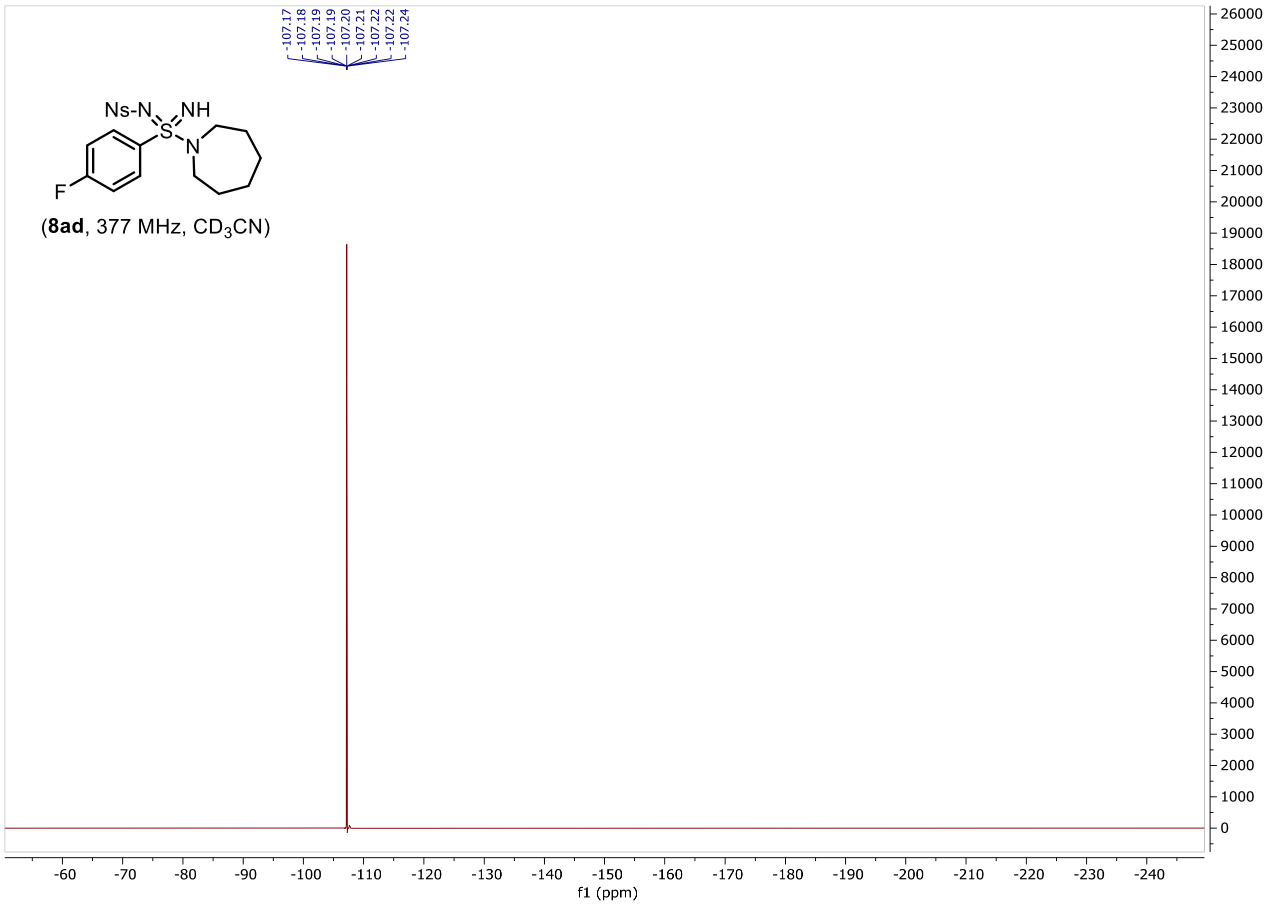
Ns-N
NH
S
N
F
(**8ad**, 377 MHz, CD_3CN)
-107.17
-107.18
-107.19
-107.19
-107.20
-107.21
-107.22
-107.22
-107.24
f1 (ppm)

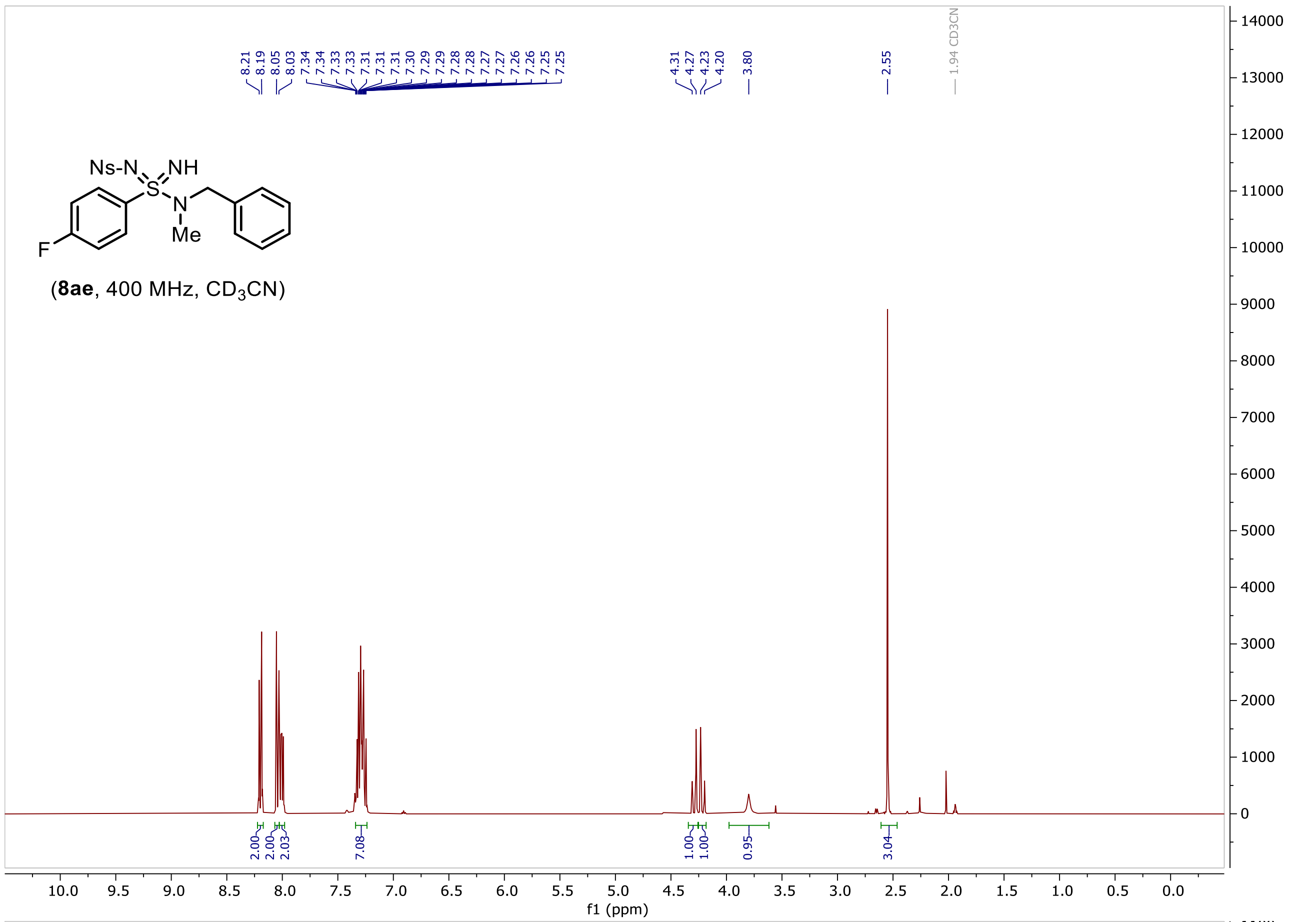

8.21
8.19
8.05
8.03
7.34
7.34
7.33
7.33
7.31
7.31
7.31
7.30
7.29
7.29
7.28
7.28
7.27
7.27
7.26
7.26
7.25
7.25
4.31
4.27
4.23
4.20
3.80
2.55
1.94 CD3CN
Ns-N
NH
S
N
Me
F
(**8ae**, 400 MHz, CD_3CN)
2.00
2.00
2.03
7.08
1.00
1.00
0.95
3.04
f1 (ppm)

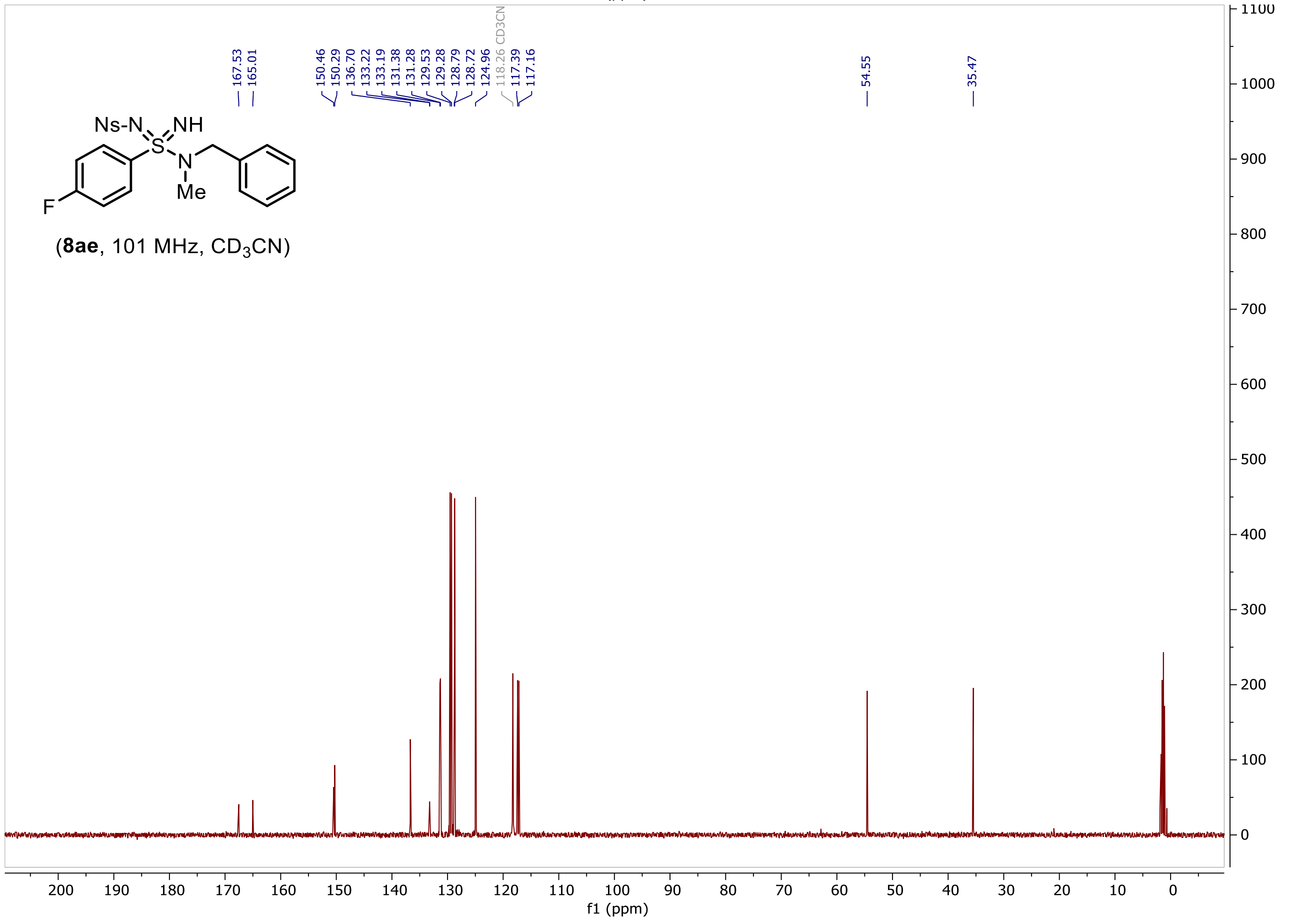

167.53
165.01
150.46
150.29
136.70
133.22
133.19
131.38
131.28
129.53
129.28
128.79
128.72
124.96
118.26 CD3CN
117.39
117.16
54.55
35.47
Ns-N
NH
S
N
Me
F
(**8ae**, 101 MHz, CD_3CN)
f1 (ppm)

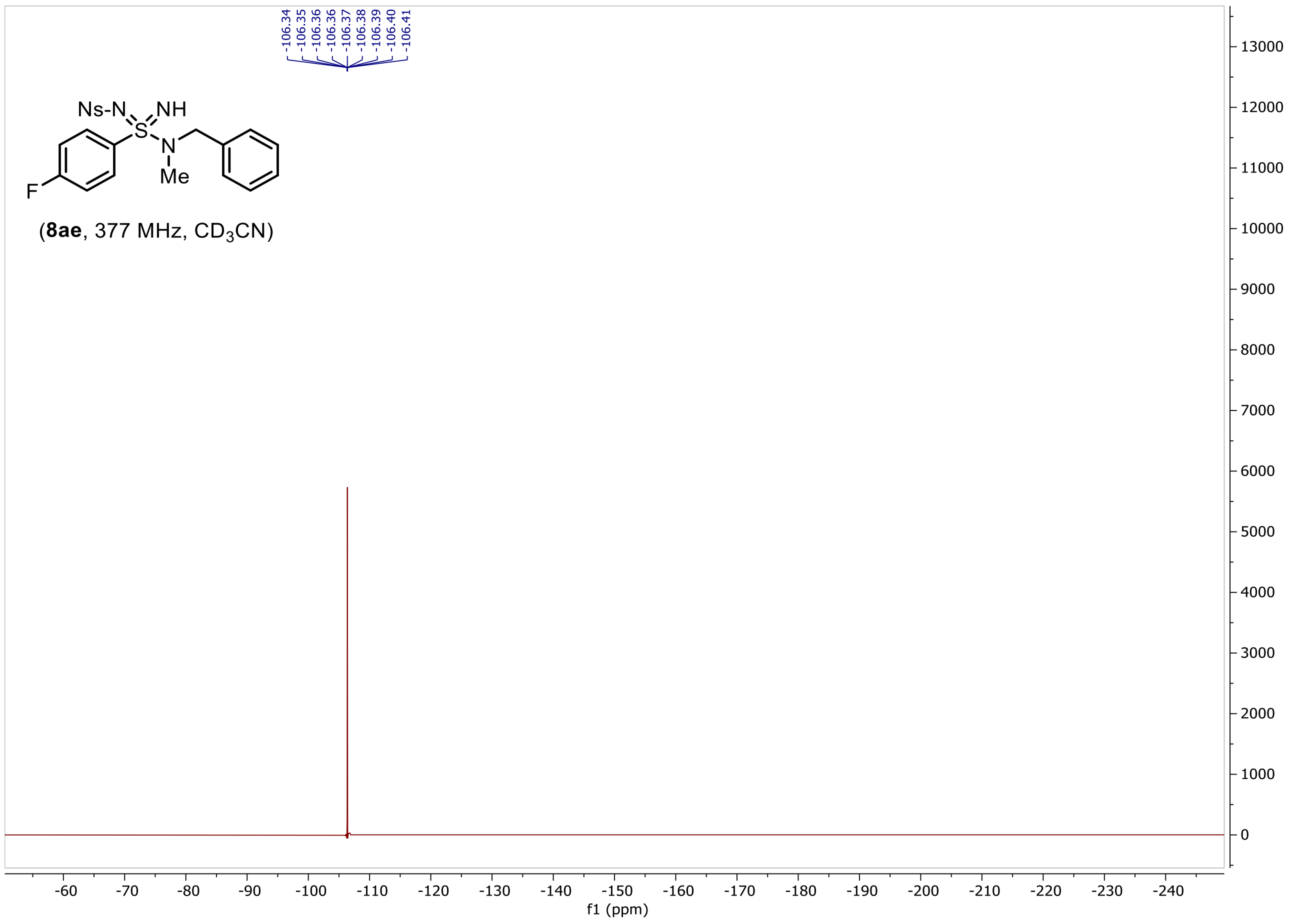
-106.34
-106.35
-106.36
-106.36
-106.37
-106.38
-106.39
-106.40
-106.41
Ns-N
NH
S
N
Me
F
(**8ae**, 377 MHz, CD3CN)
f1 (ppm)

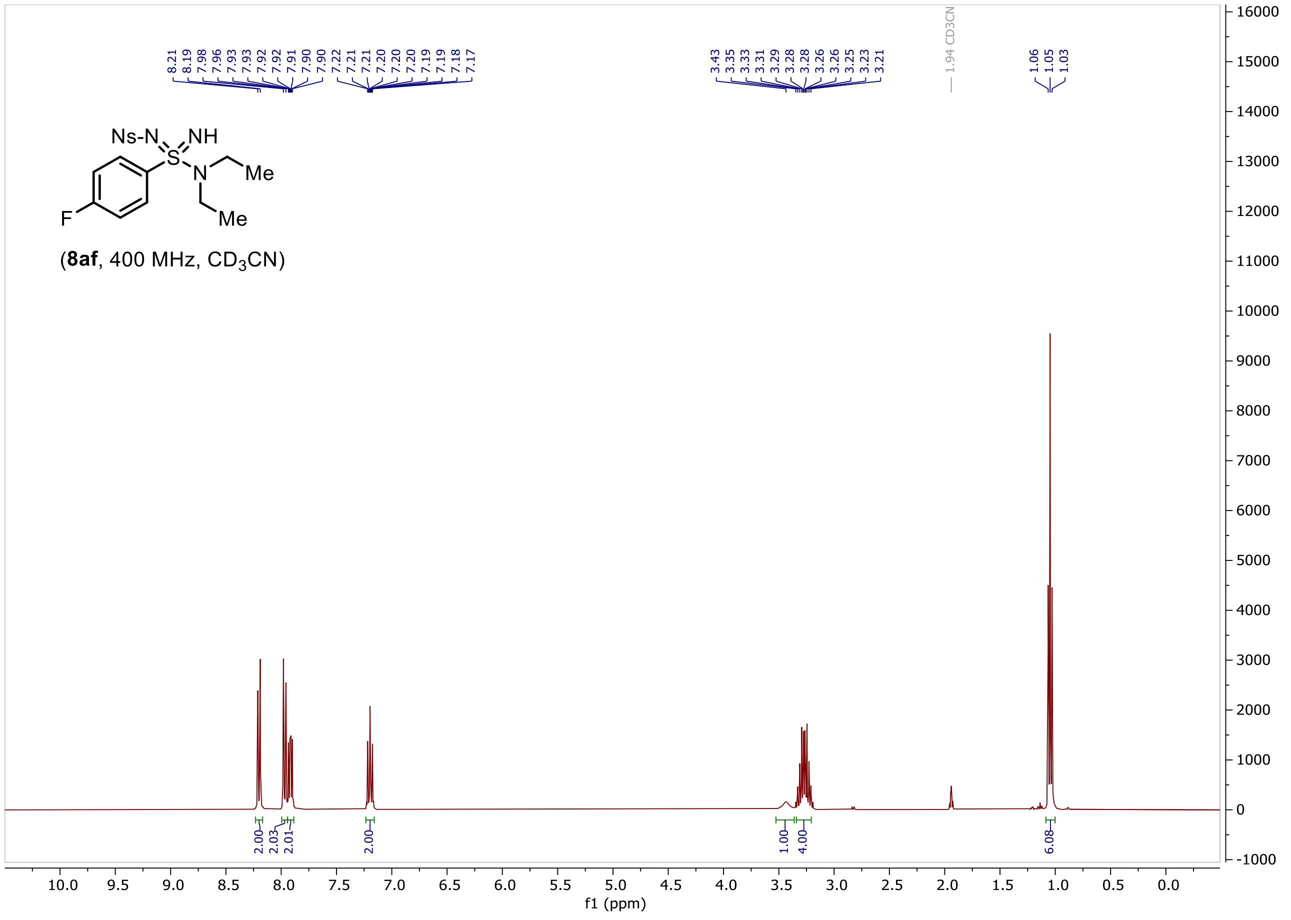
8.21
8.19
7.98
7.96
7.93
7.93
7.92
7.92
7.91
7.90
7.90
7.22
7.21
7.21
7.20
7.20
7.20
7.19
7.19
7.18
7.17
3.43
3.35
3.33
3.31
3.29
3.28
3.28
3.26
3.26
3.25
3.23
3.21
1.94 CD3CN
1.06
1.05
1.03
Ns-N
NH
S
N
Me
Me
F
(**8af**, 400 MHz, CD3CN)
2.00
2.03
2.01
2.00
1.00
4.00
6.08
f1 (ppm)

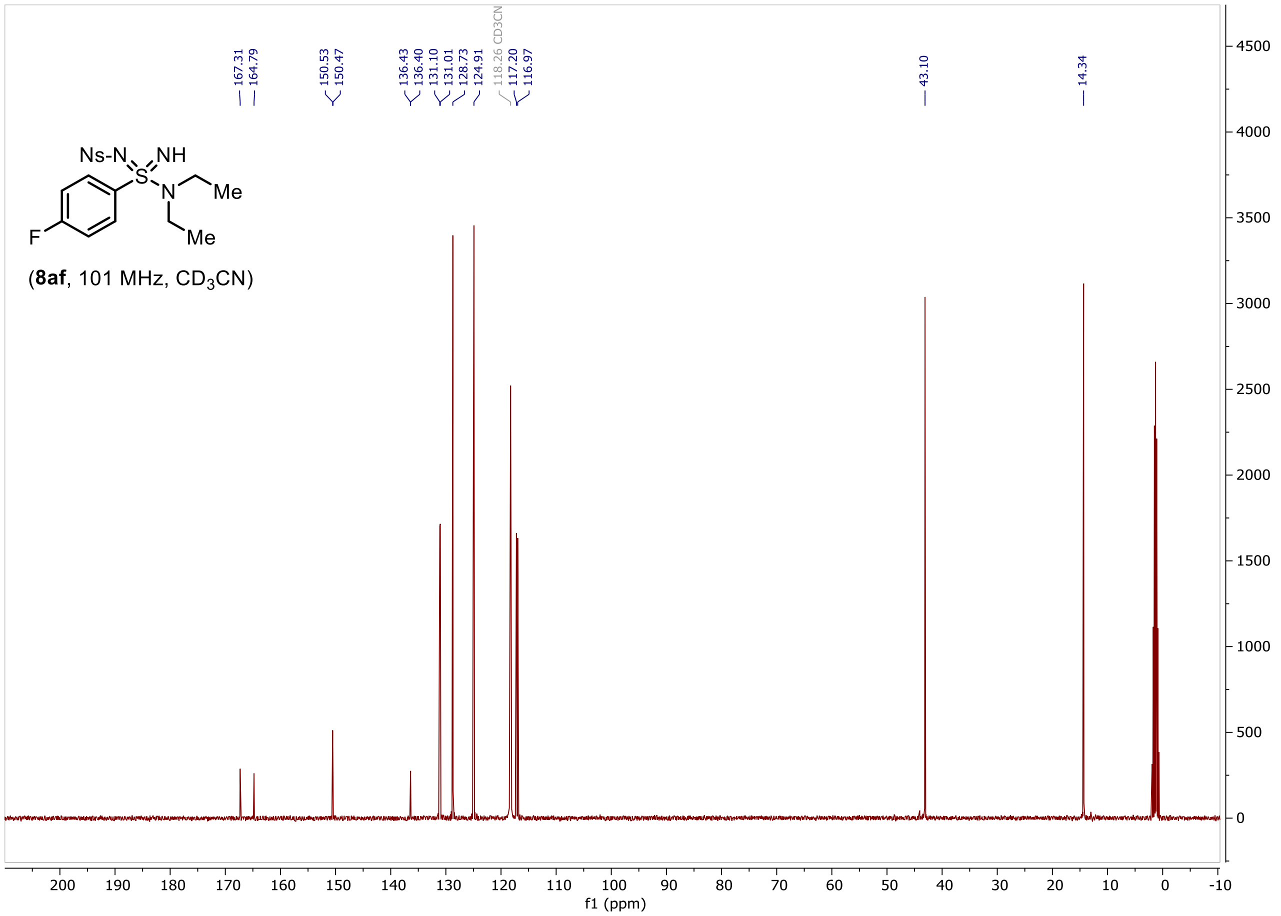

167.31
164.79
150.53
150.47
136.43
136.40
131.10
131.01
128.73
124.91
118.26 CD3CN
117.20
116.97
43.10
14.34
Ns-N
NH
S
N
Me
Me
F
(8af, 101 MHz, CD3CN)
f1 (ppm)

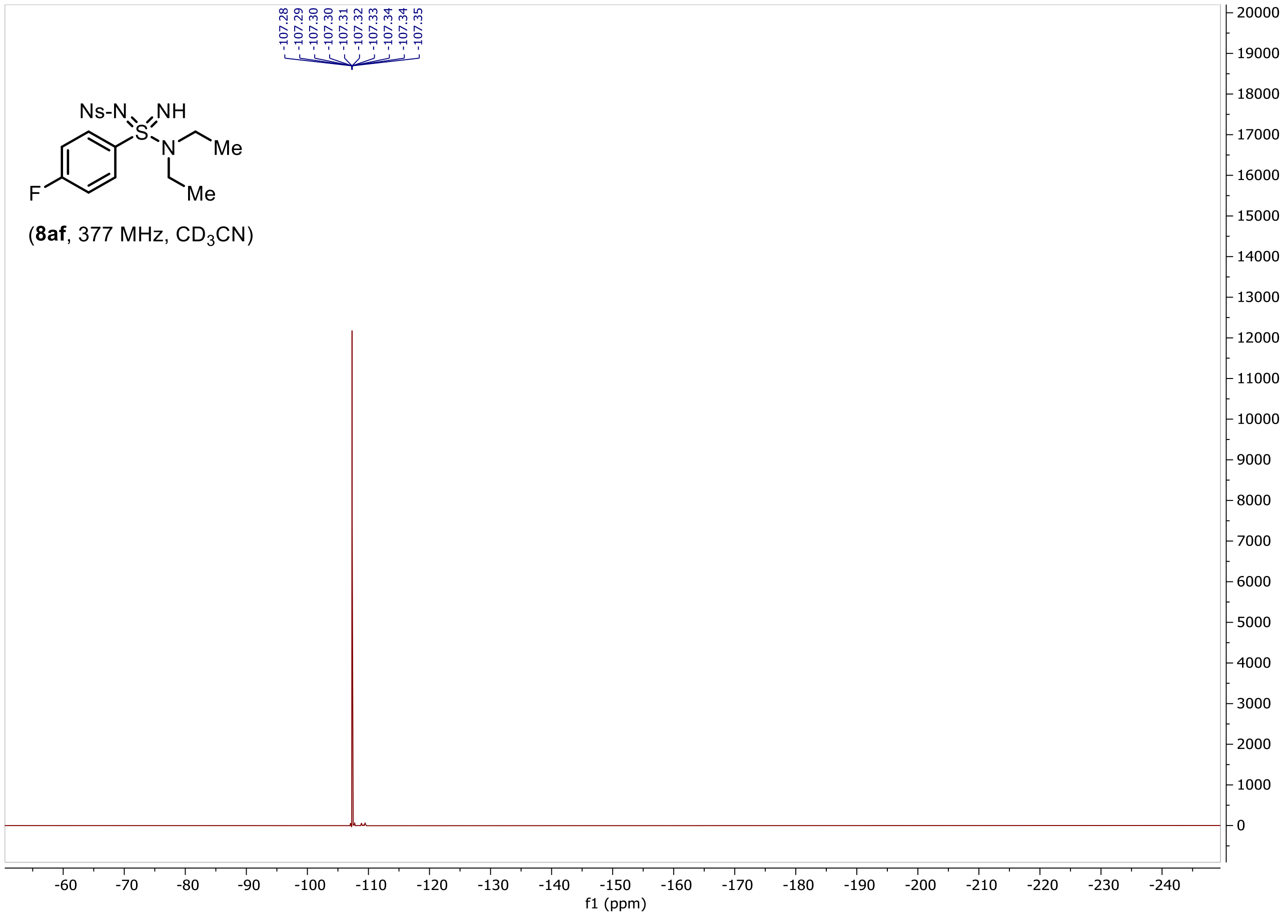

-107.28
-107.29
-107.30
-107.30
-107.31
-107.32
-107.33
-107.34
-107.34
-107.35
Ns-N
NH
S
N
Me
Me
F
(8af, 377 MHz, CD3CN)
f1 (ppm)

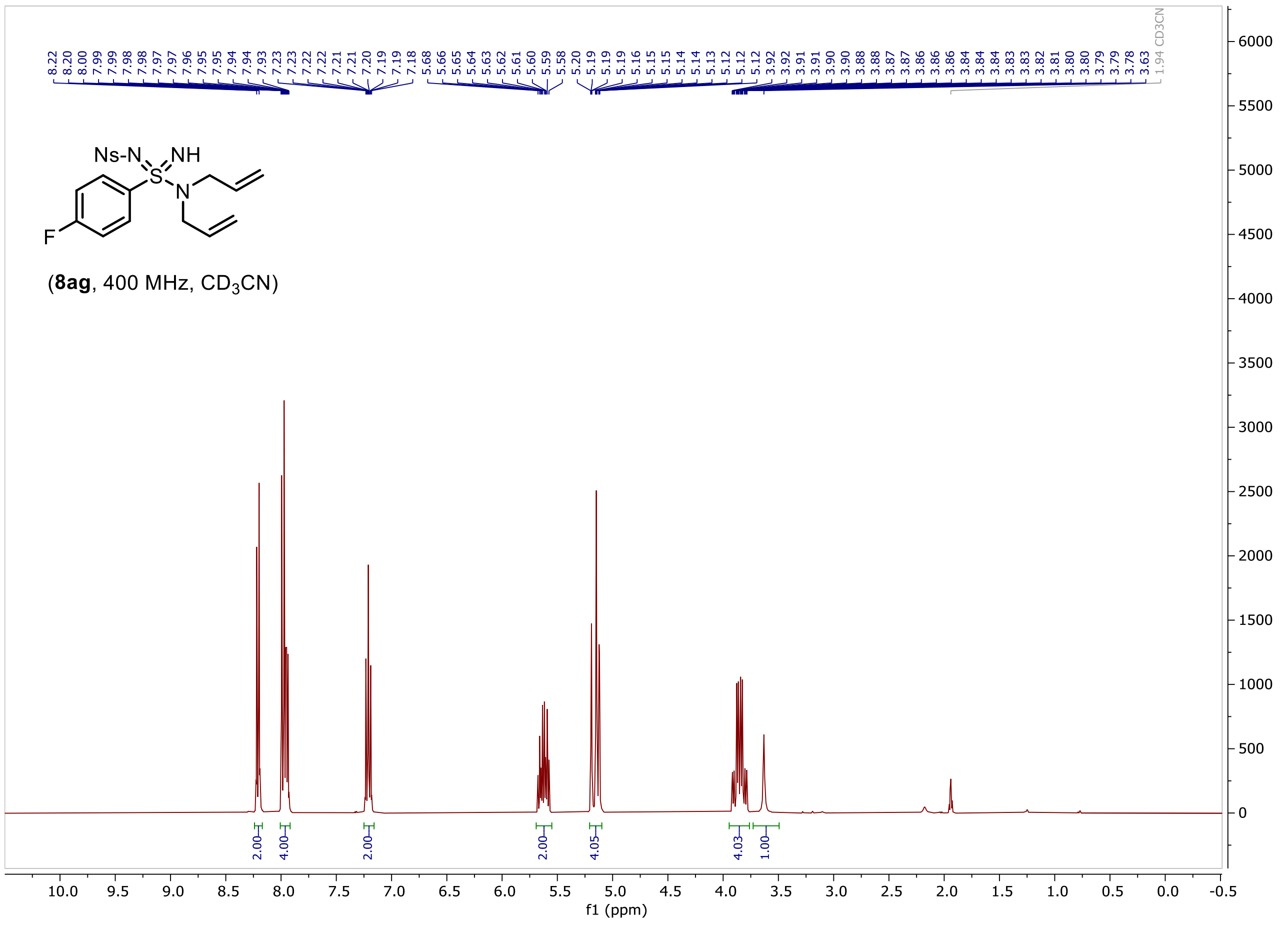
Ns-N
NH
S
N
F
(8ag, 400 MHz, CD3CN)
f1 (ppm)

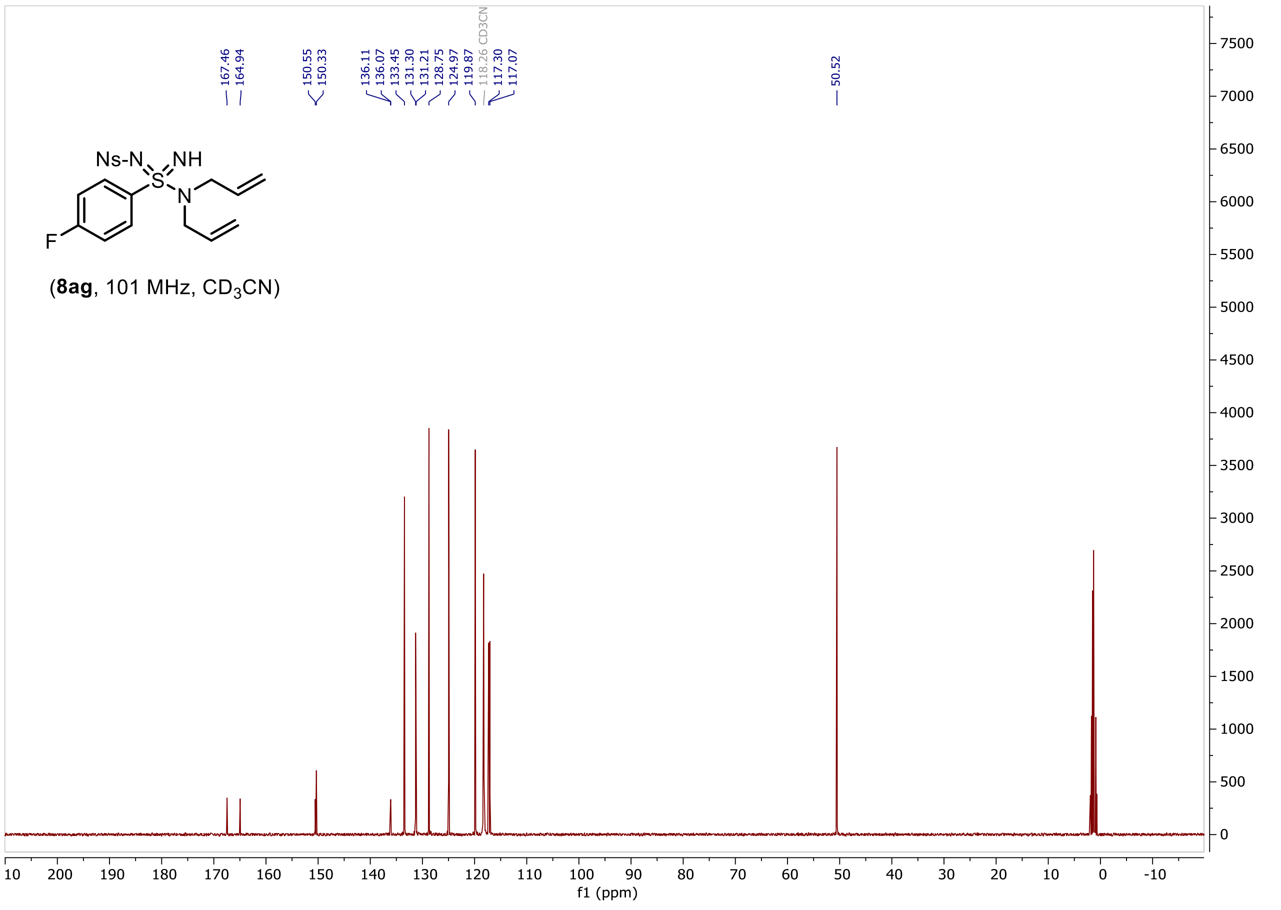
Ns-N
NH
S
N
F
(8ag, 101 MHz, CD3CN)
f1 (ppm)

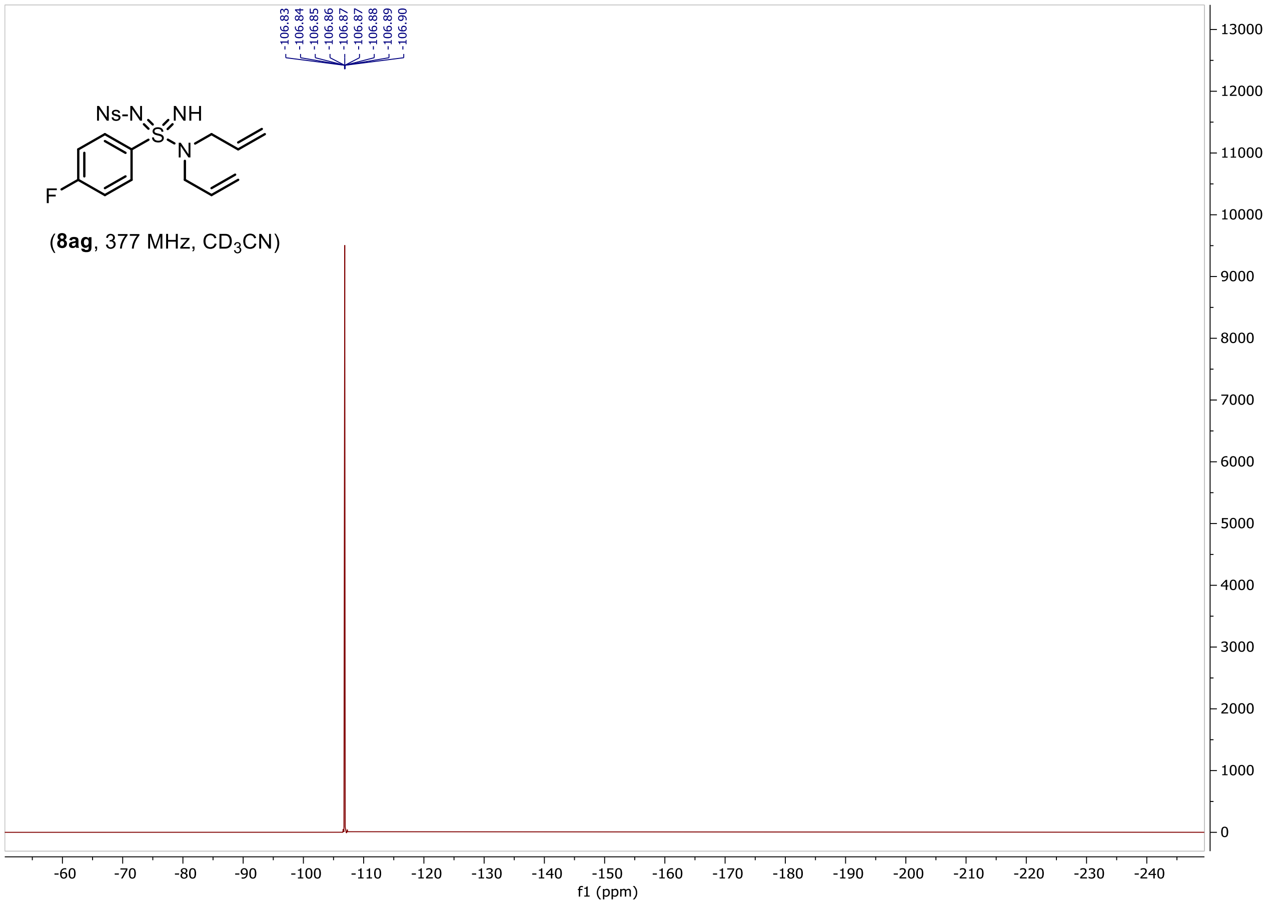
Ns-N
NH
S
N
F
(8ag, 377 MHz, CD3CN)
f1 (ppm)

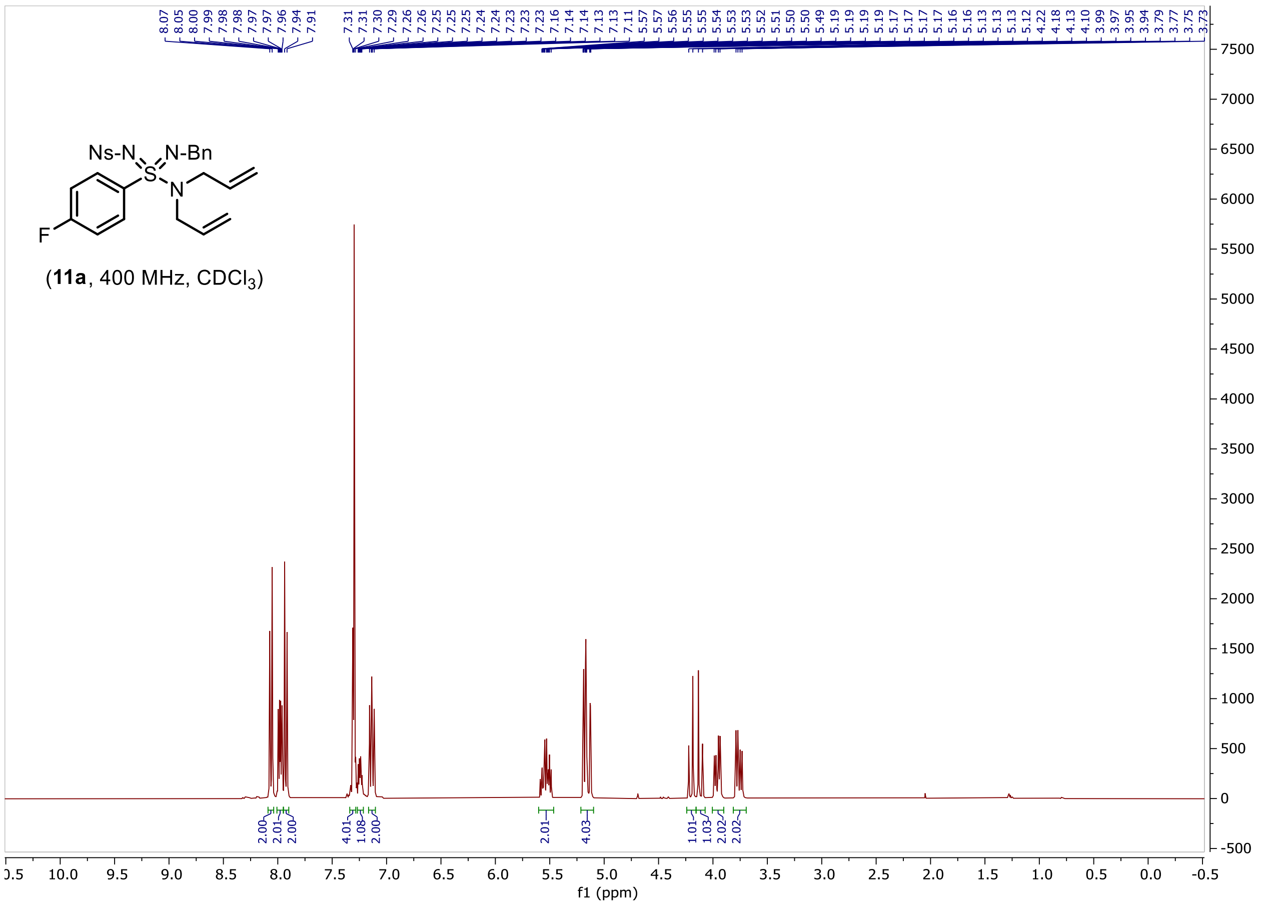
Ns-N
N-Bn
S
N
F
(11a, 400 MHz, CDCl3)
f1 (ppm)

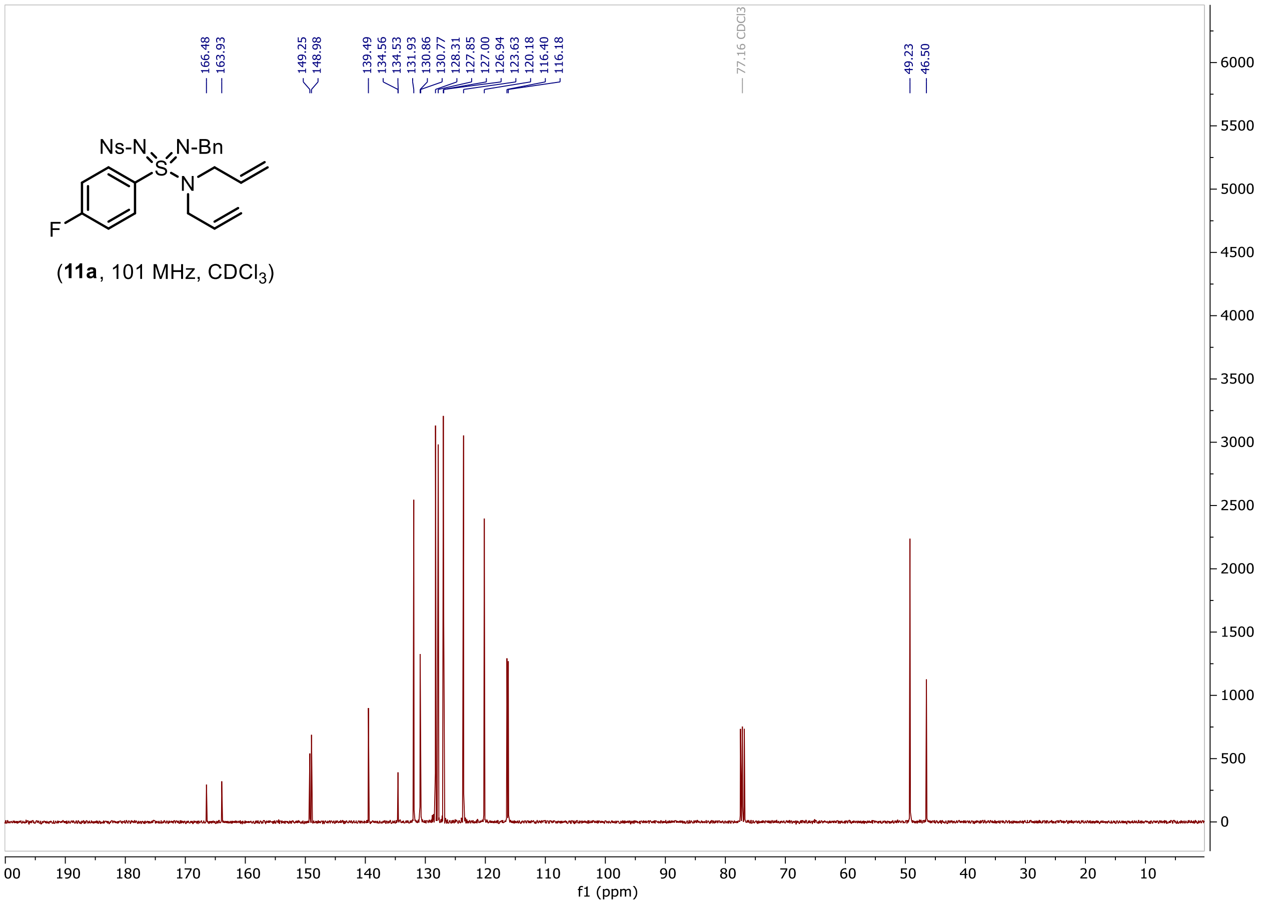
166.48
163.93
149.25
148.98
139.49
134.56
134.53
131.93
130.86
130.77
128.31
127.85
127.00
126.94
123.63
120.18
116.40
116.18
77.16 CDCl3
49.23
46.50
Ns-N
N-Bn
S
N
F
(11a, 101 MHz, CDCl3)
f1 (ppm)

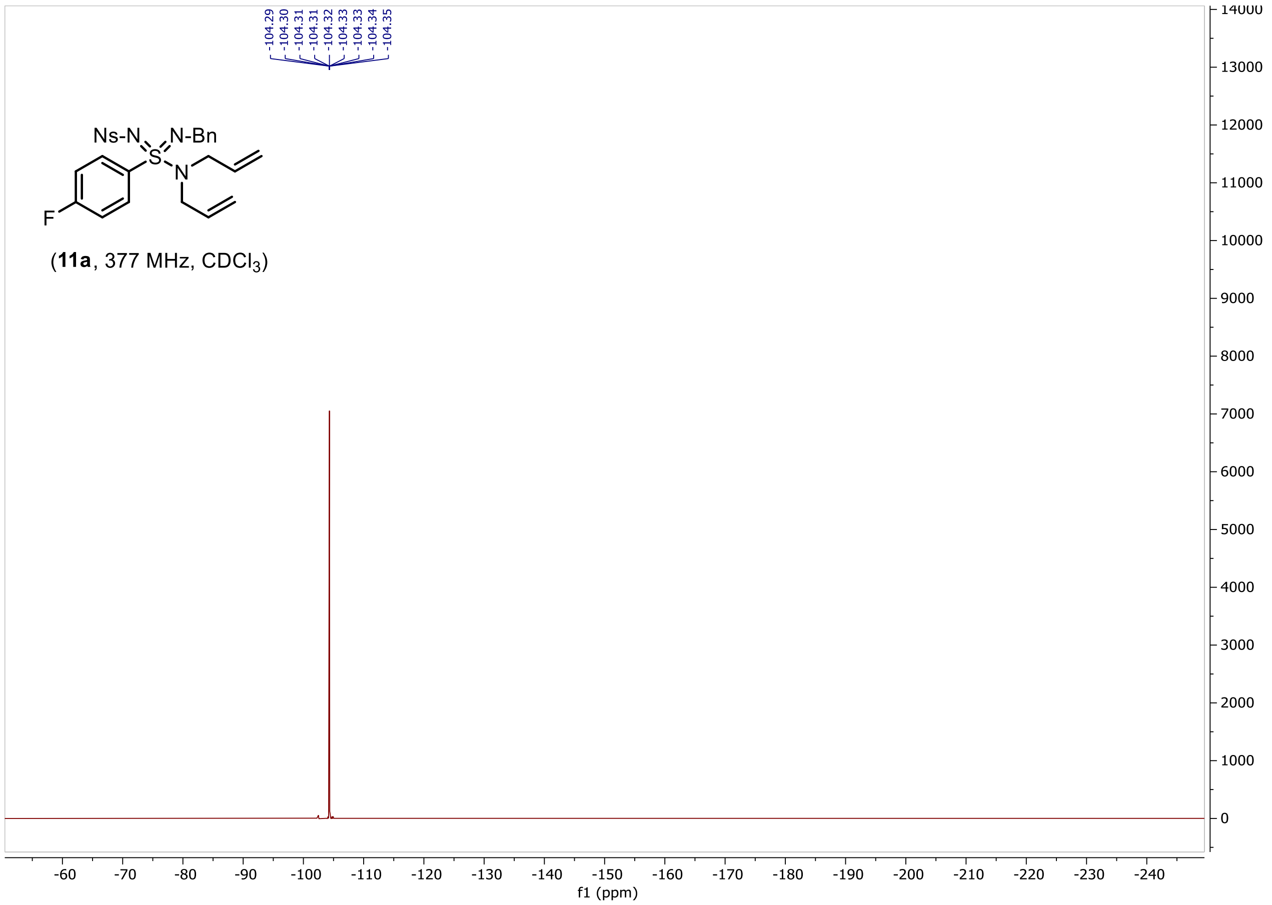
-104.29
-104.30
-104.31
-104.31
-104.32
-104.33
-104.33
-104.34
-104.35
Ns-N
N-Bn
S
N
F
(11a, 377 MHz, CDCl3)
f1 (ppm)

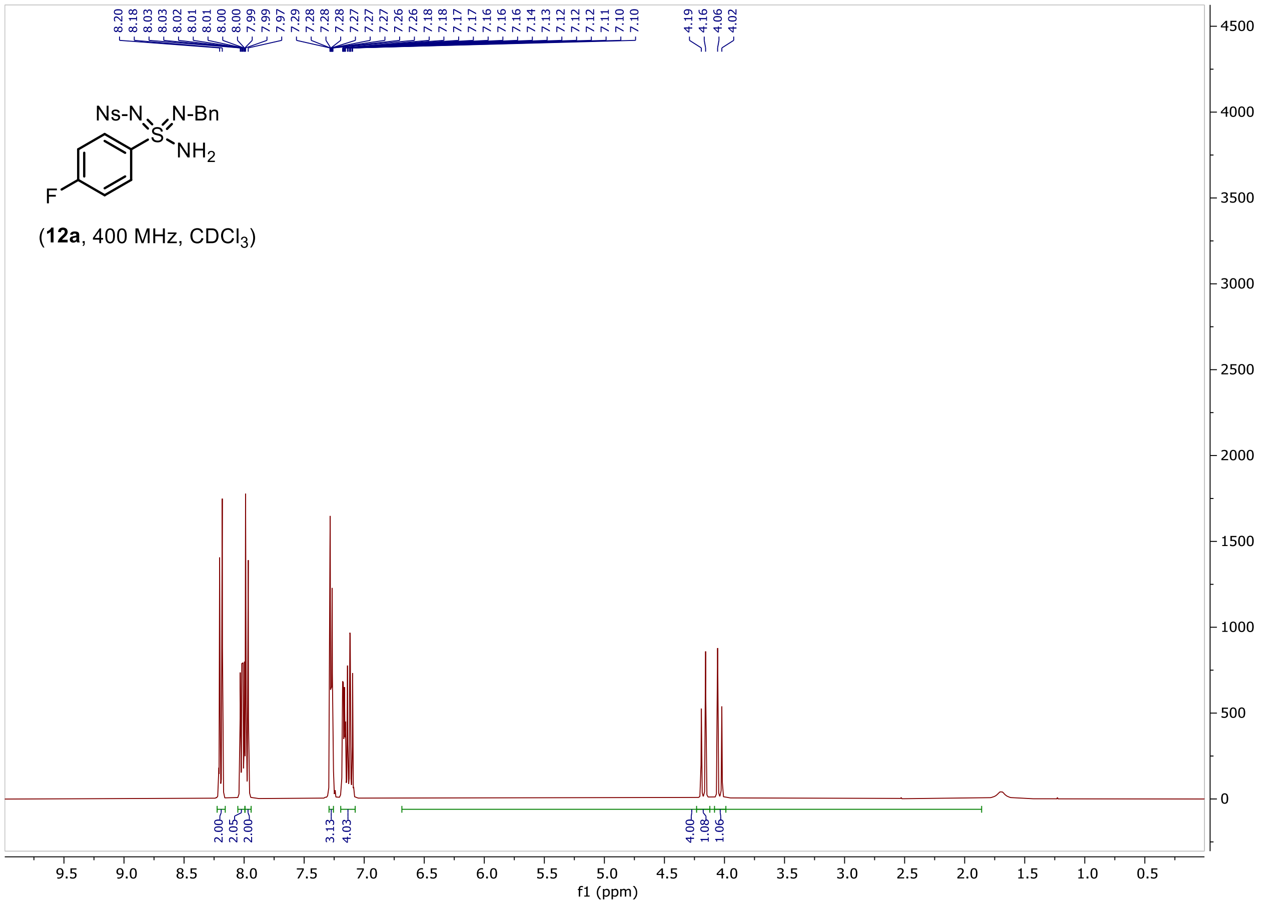

Ns-N
N-Bn
S
NH2
F
(12a, 400 MHz, CDCl3)
8.20 8.18 8.03 8.02 8.01 8.01 8.00 8.00 7.99 7.99 7.97 7.29 7.28 7.28 7.28 7.27 7.27 7.27 7.26 7.26 7.18 7.18 7.17 7.17 7.16 7.16 7.16 7.14 7.13 7.12 7.12 7.12 7.11 7.10 7.10
4.19 4.16 4.06 4.02
2.00 2.05 2.00 3.13 4.03 4.00 1.08 1.06
f1 (ppm)

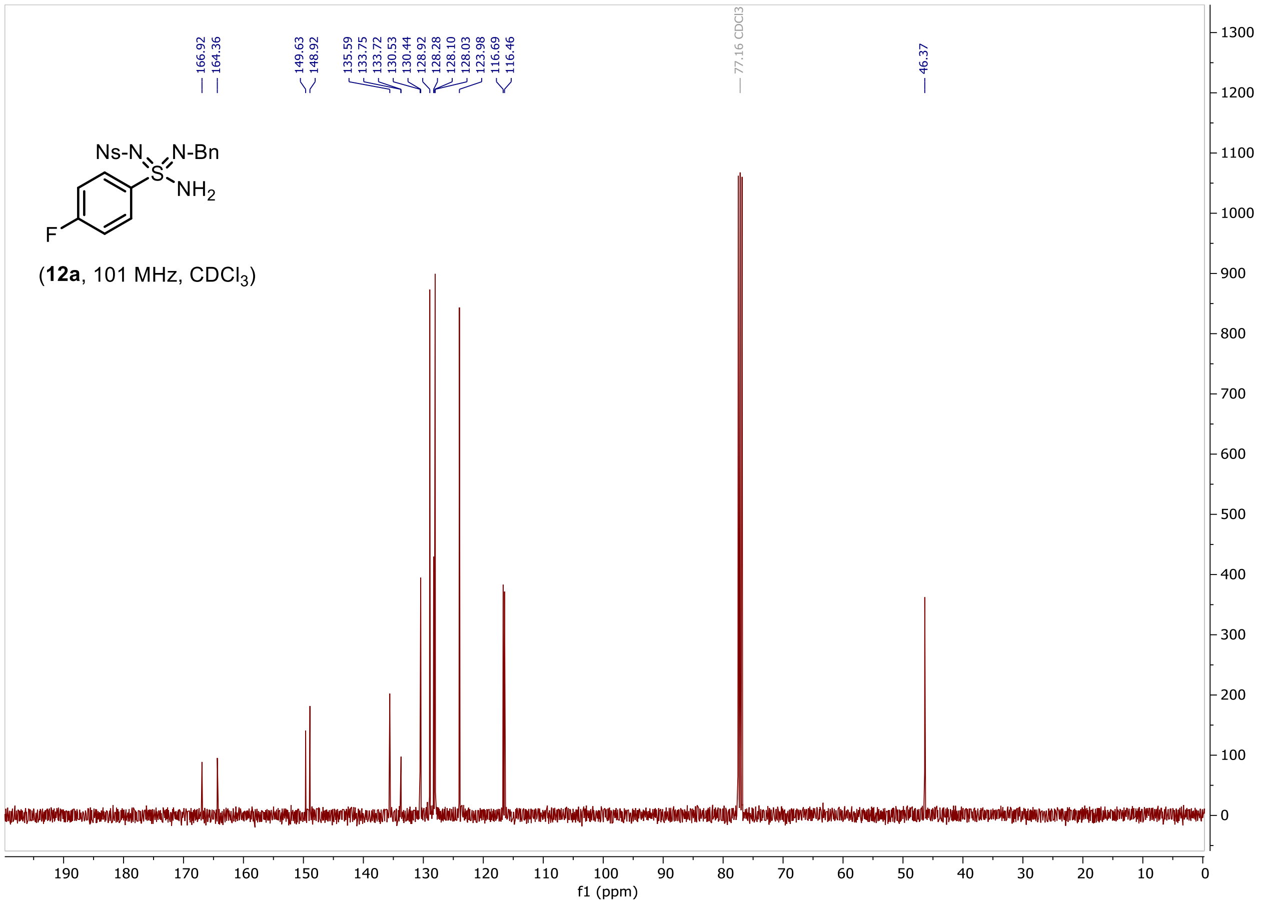

Ns-N
N-Bn
S
NH2
F
(12a, 101 MHz, CDCl3)
166.92 164.36 149.63 148.92 135.59 133.75 133.72 130.53 130.44 128.92 128.28 128.10 128.03 123.98 116.69 116.46
77.16 CDCl3
46.37
f1 (ppm)

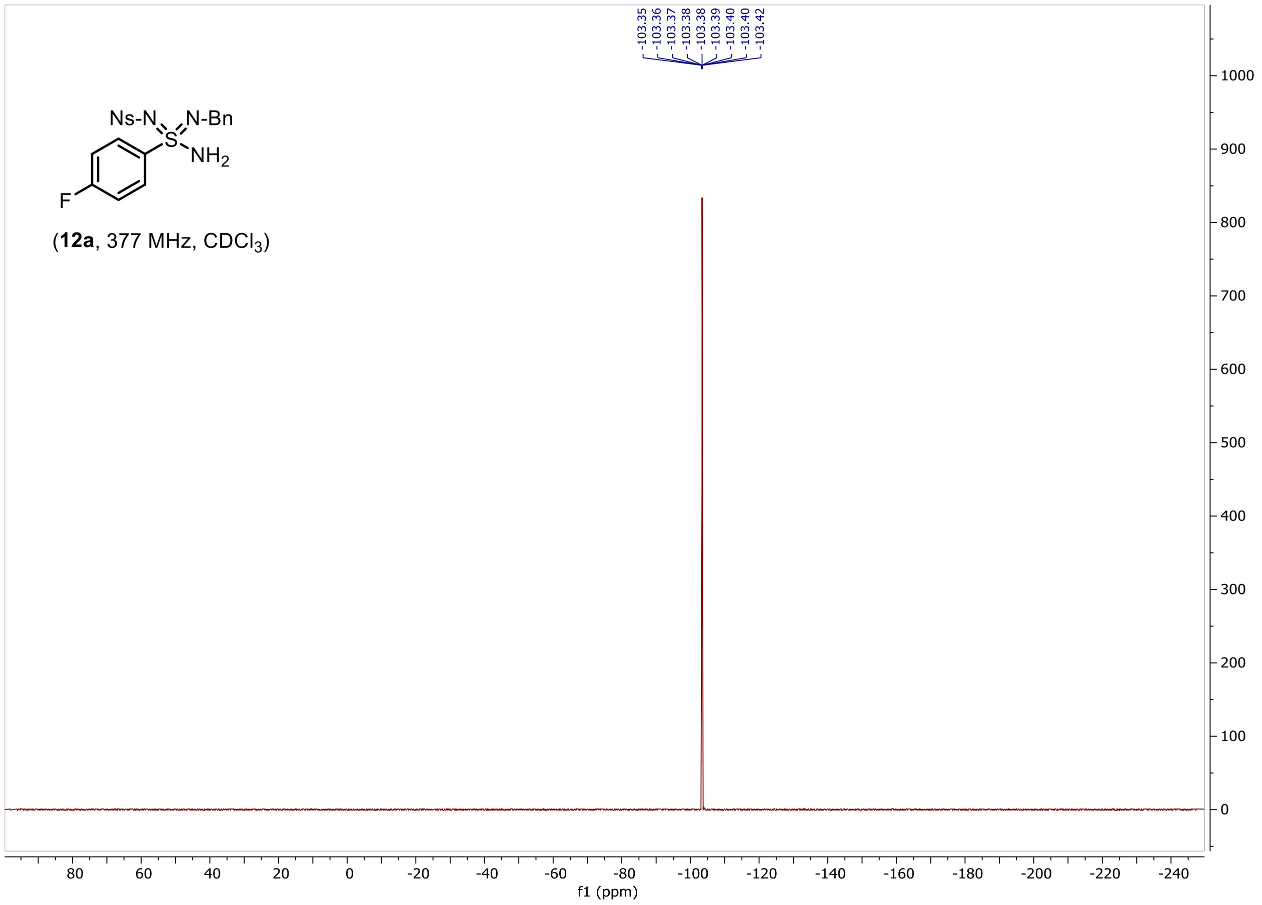
Ns-N
N-Bn
S
NH2
F
(12a, 377 MHz, CDCl3)
-103.35 -103.36 -103.37 -103.38 -103.38 -103.39 -103.40 -103.40 -103.42
f1 (ppm)

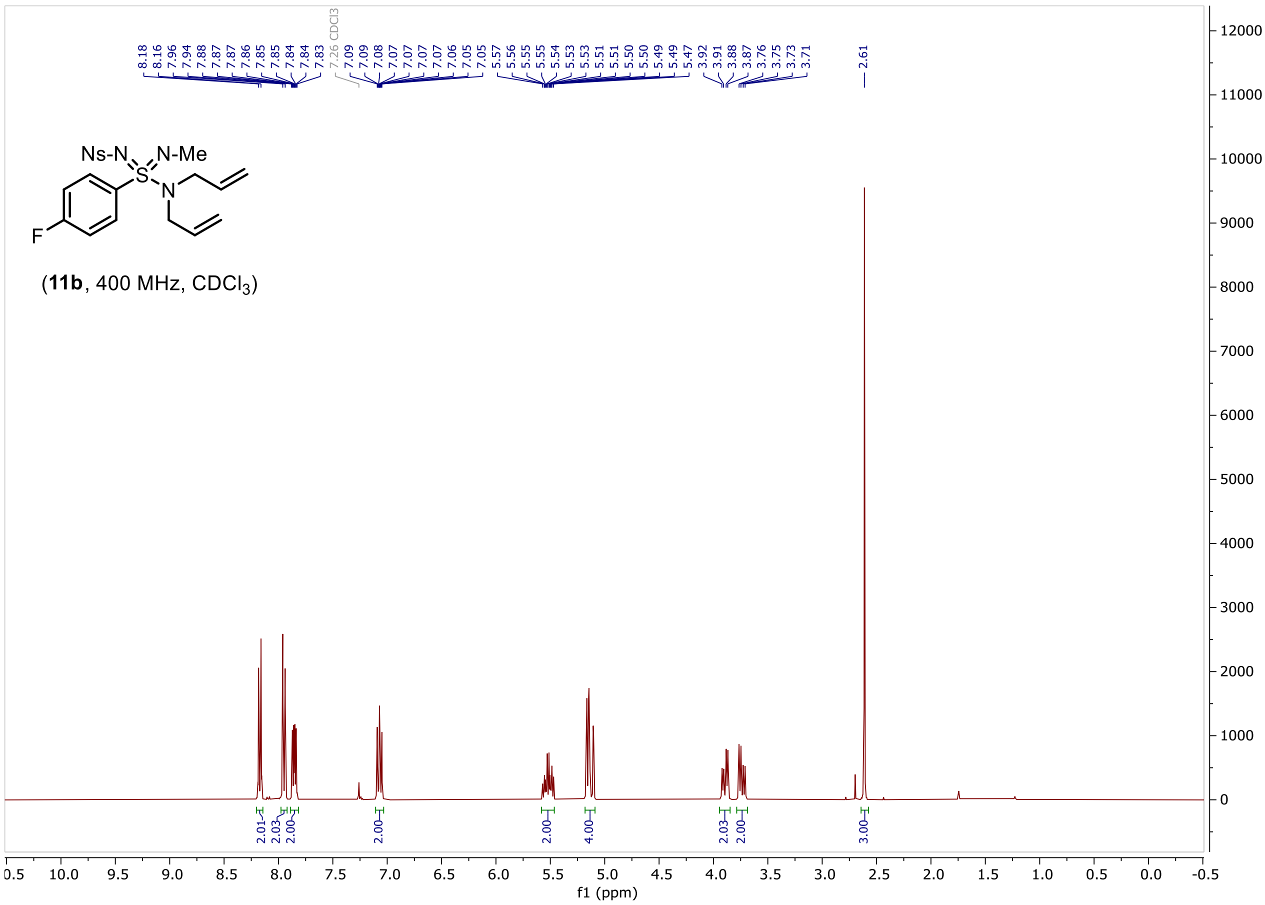
Ns-N
N-Me
S
N
F
(11b, 400 MHz, CDCl3)
8.18 8.16 7.96 7.94 7.88 7.87 7.87 7.86 7.85 7.85 7.84 7.84 7.83
7.26 CDCl3
7.09 7.09 7.08 7.07 7.07 7.07 7.06 7.05 7.05
5.57 5.56 5.55 5.55 5.54 5.53 5.53 5.51 5.51 5.50 5.50 5.49 5.49 5.47
3.92 3.91 3.88 3.87 3.76 3.75 3.73 3.71
2.61
2.01 2.03 2.00 2.00 2.00 4.00 2.03 2.00 3.00
f1 (ppm)

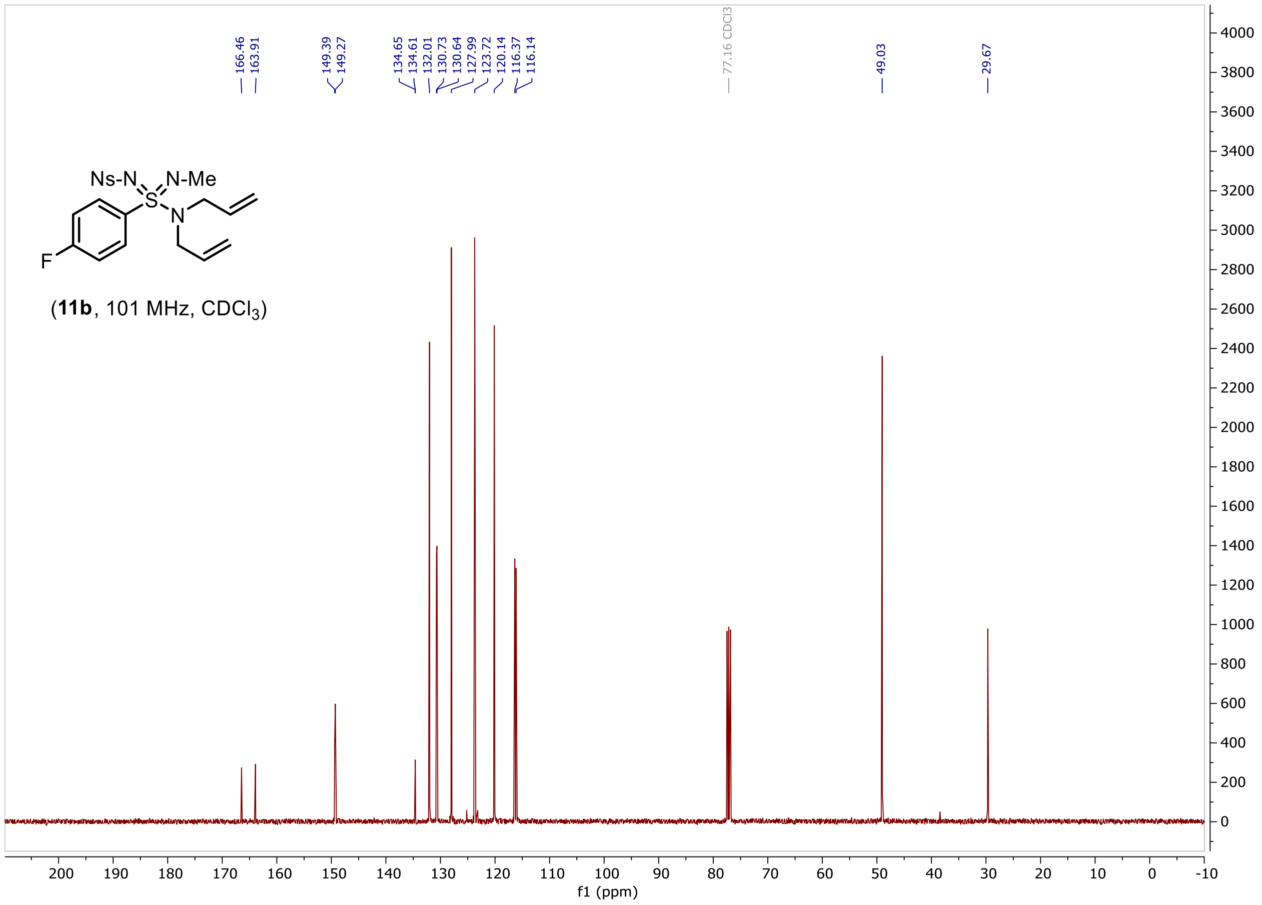

(**11b**, 101 MHz, $CDCl_3$)

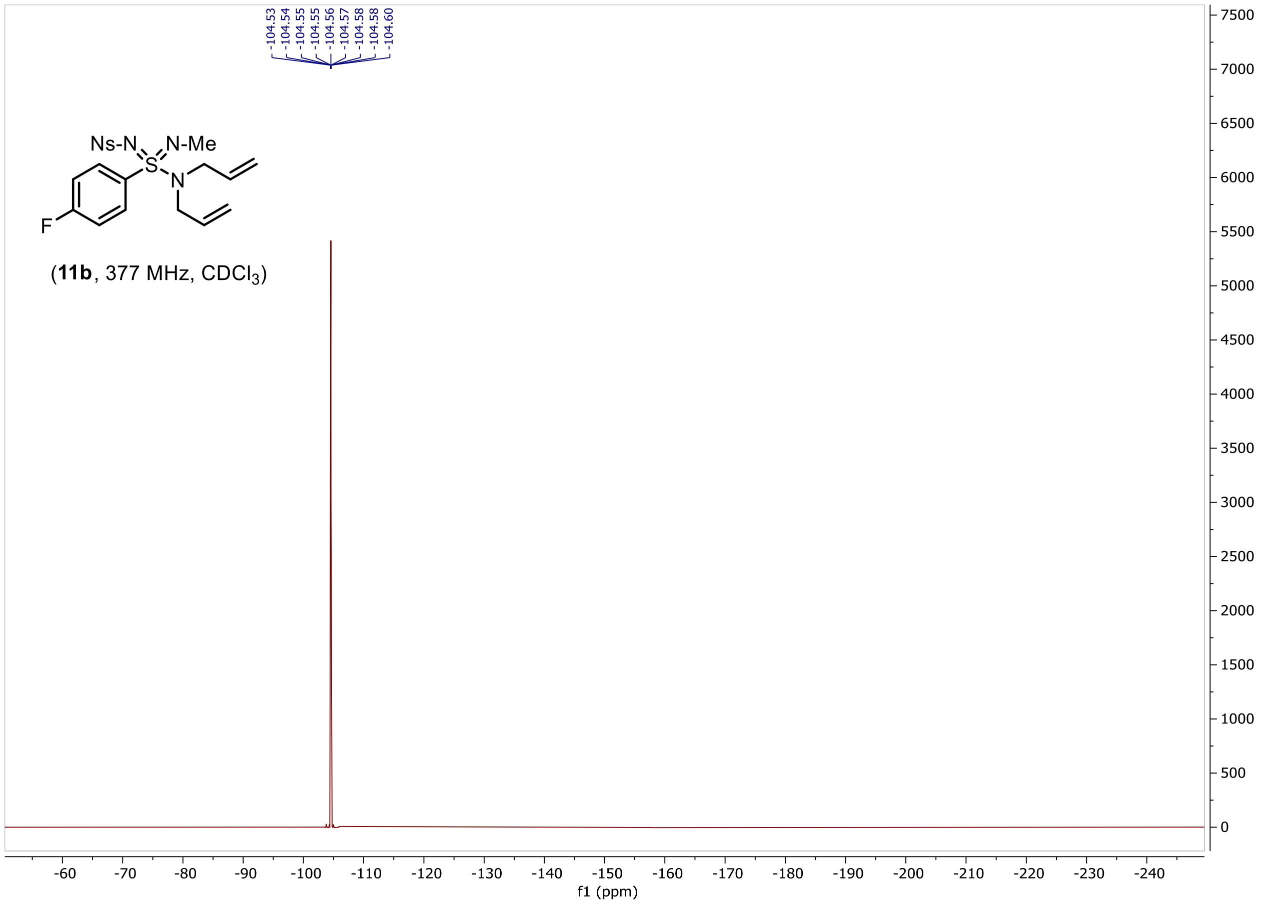

(**11b**, 377 MHz, $CDCl_3$)

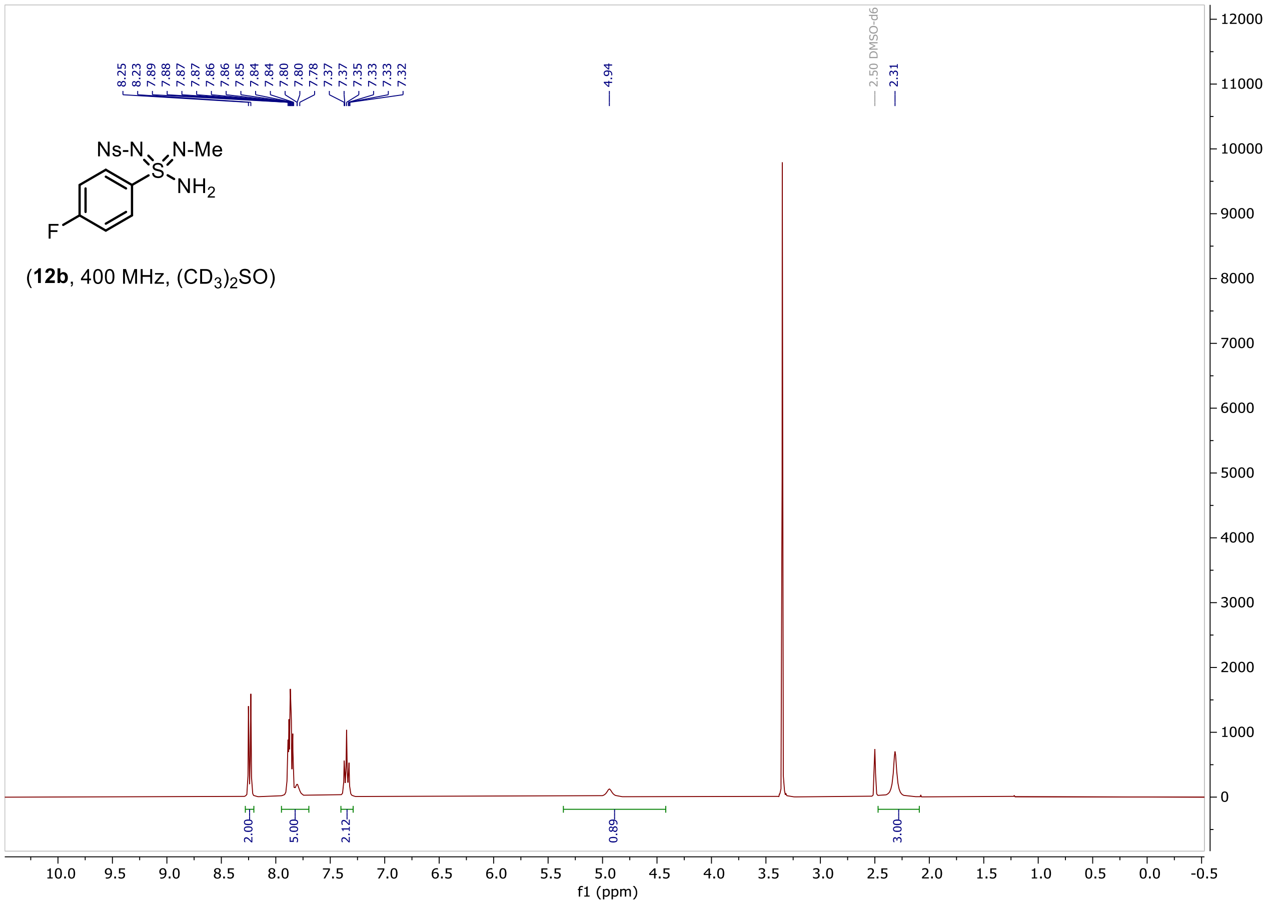
8.25
8.23
7.89
7.88
7.87
7.87
7.86
7.86
7.85
7.84
7.84
7.80
7.80
7.78
7.37
7.37
7.35
7.33
7.33
7.32
4.94
2.50 DMSO-d6
2.31
Ns-N
N-Me
S
NH2
F
(12b, 400 MHz, (CD3)2SO)
2.00
5.00
2.12
0.89
3.00
f1 (ppm)

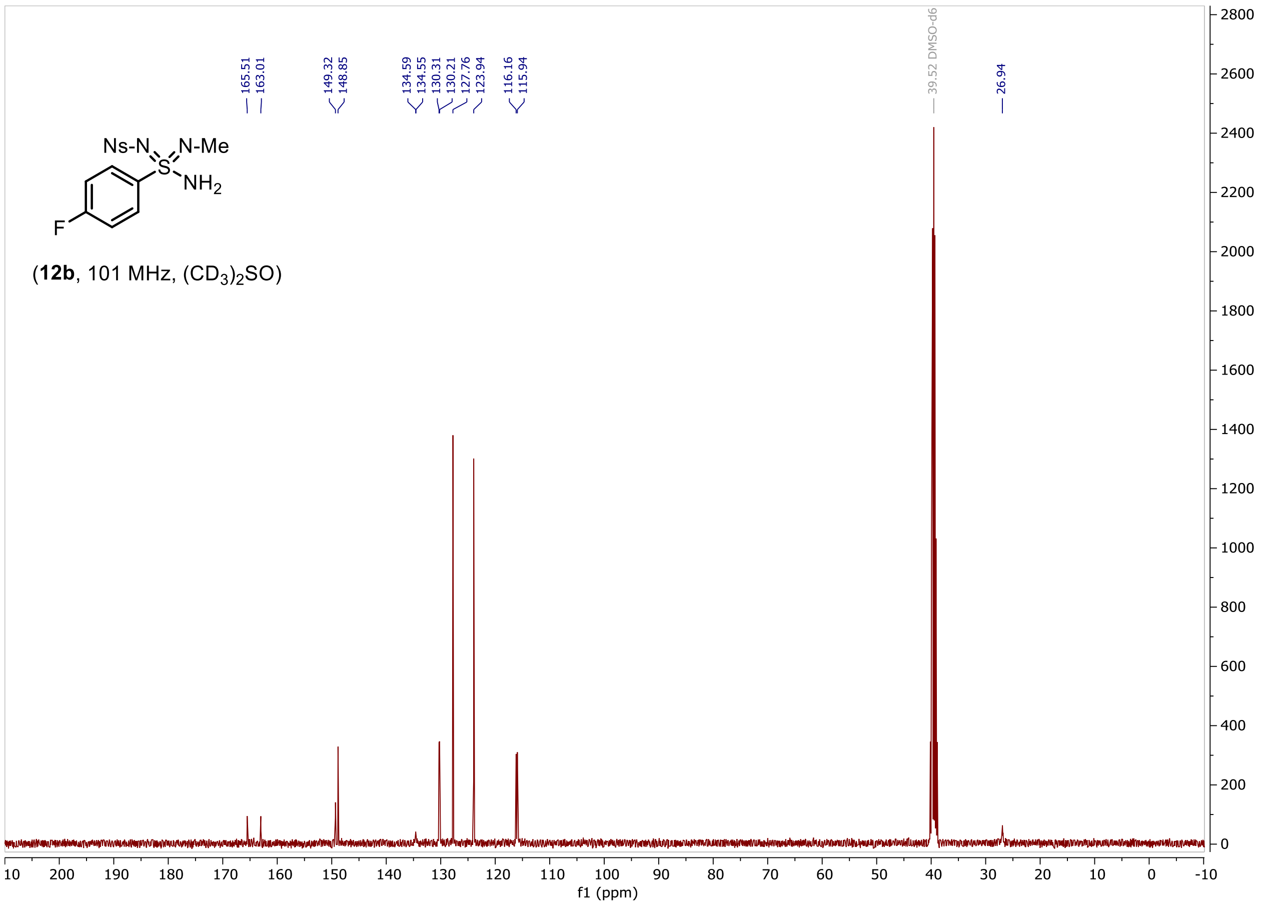
165.51
163.01
149.32
148.85
134.59
134.55
130.31
130.21
127.76
123.94
116.16
115.94
39.52 DMSO-d6
26.94
Ns-N
N-Me
S
NH2
F
(12b, 101 MHz, (CD3)2SO)
f1 (ppm)

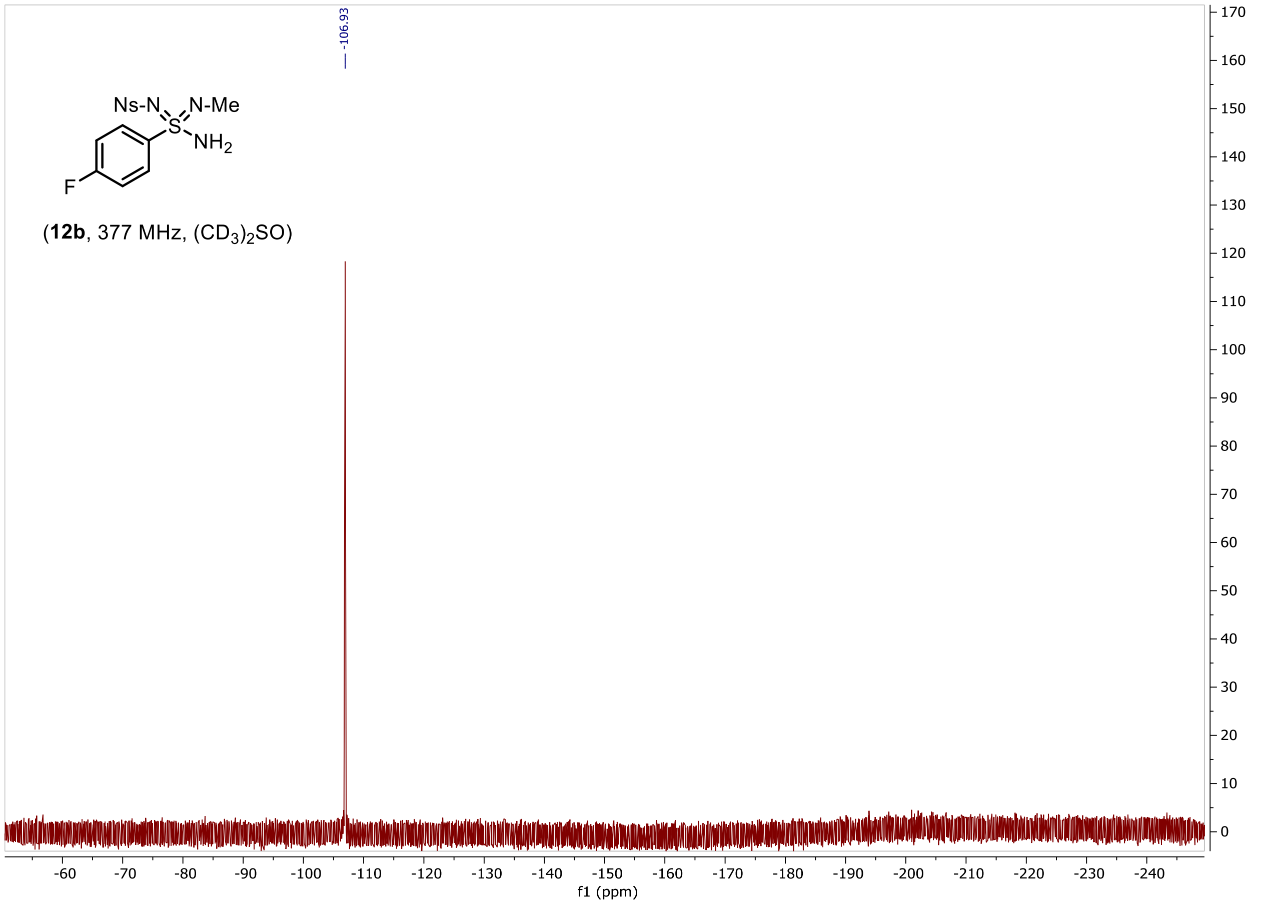

(**12b**, 377 MHz, $(CD_3)_2SO$)

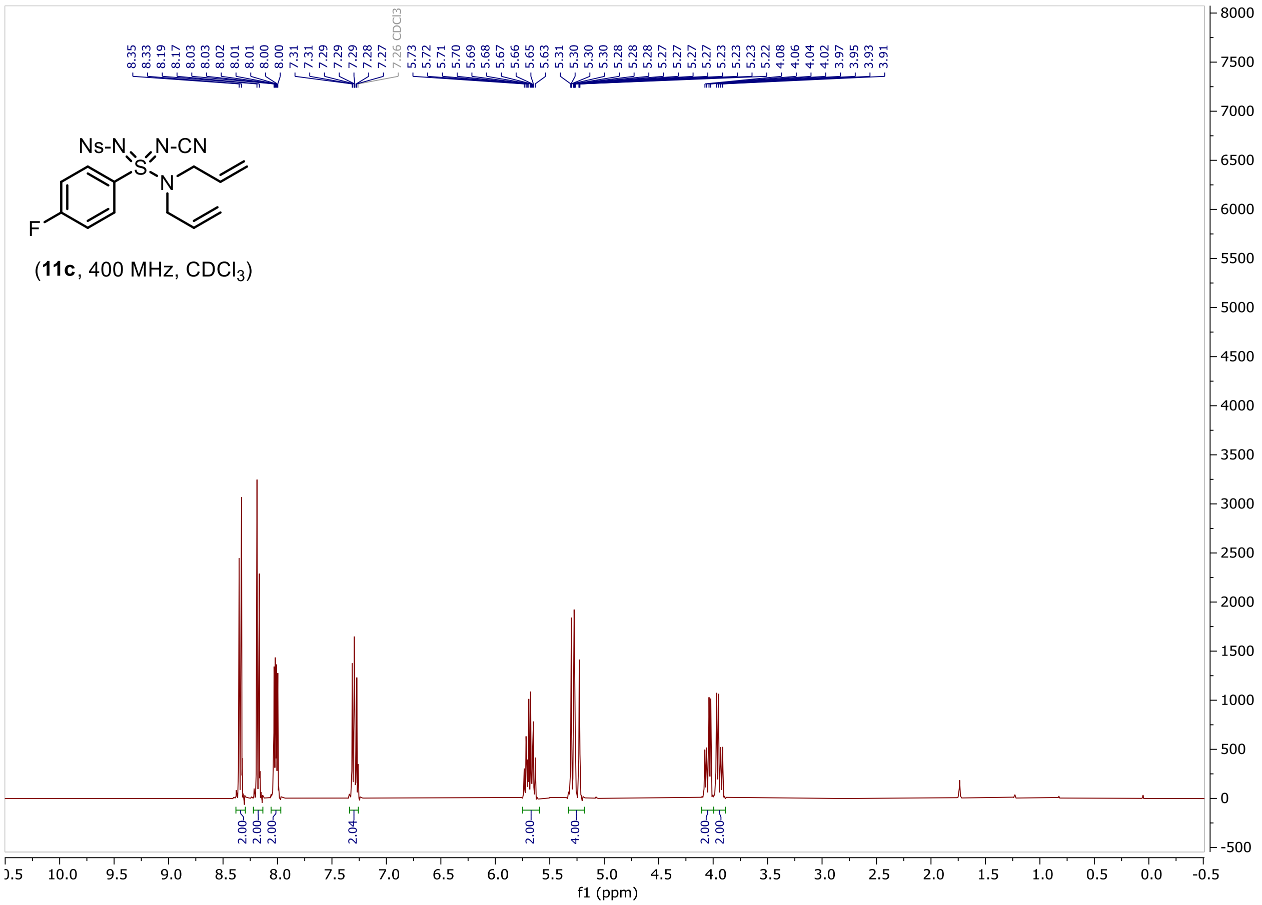

(**11c**, 400 MHz, $CDCl_3$)

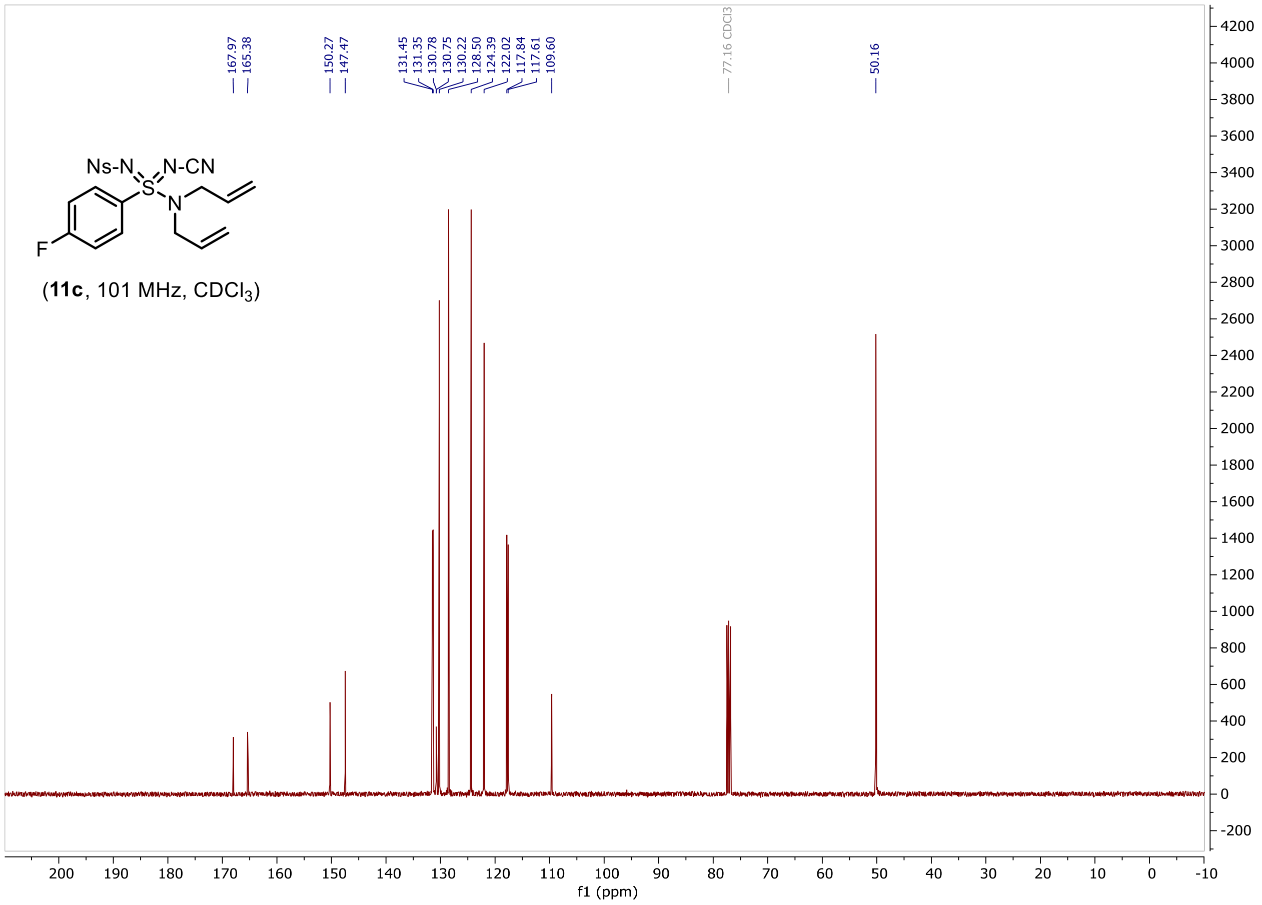
167.97
165.38
150.27
147.47
131.45
131.35
130.78
130.75
130.22
128.50
124.39
122.02
117.84
117.61
109.60
77.16 CDCl3
50.16
Ns-N
N-CN
S
N
F
(11c, 101 MHz, CDCl3)
f1 (ppm)

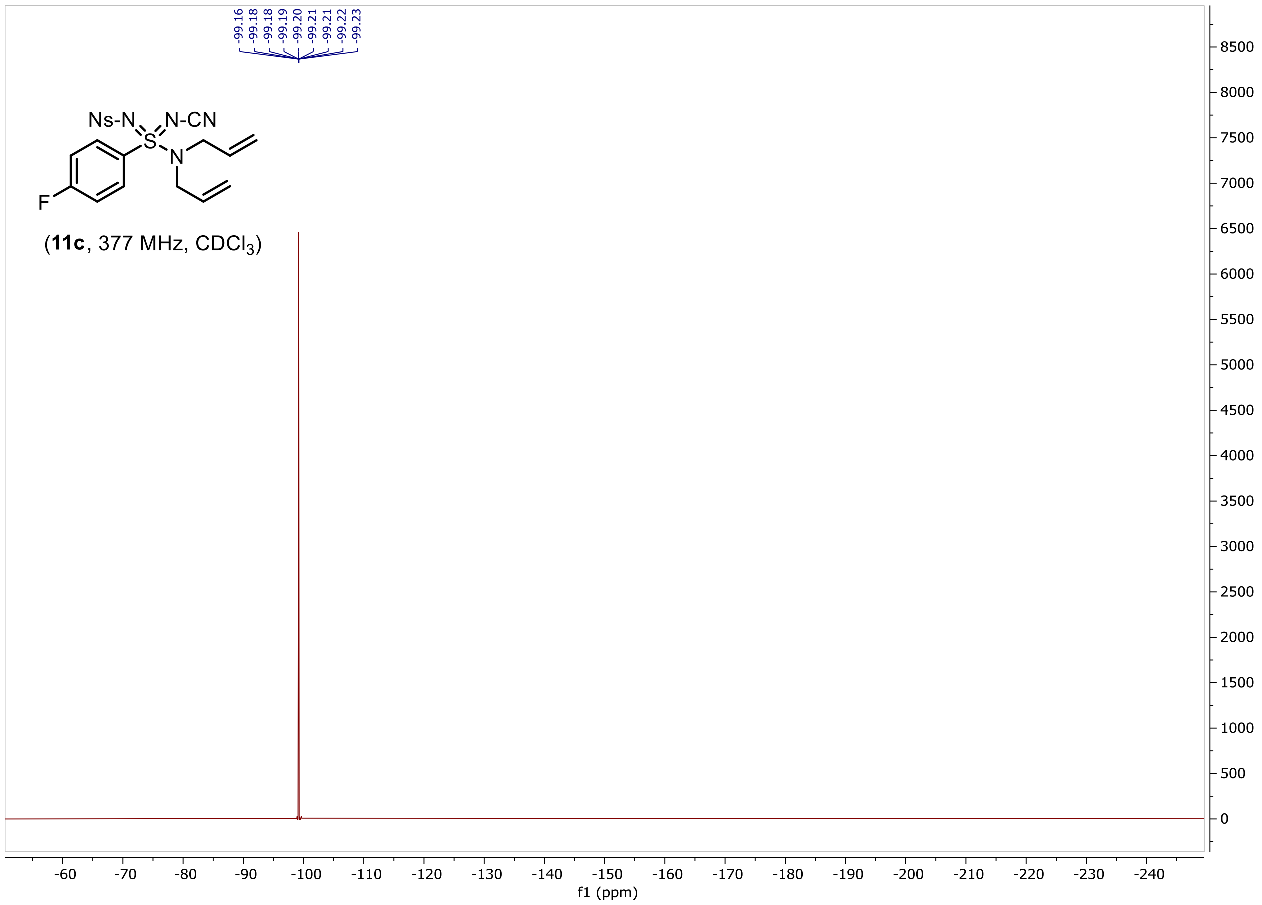
-99.16
-99.18
-99.18
-99.19
-99.20
-99.21
-99.21
-99.22
-99.23
Ns-N
N-CN
S
N
F
(11c, 377 MHz, CDCl3)
f1 (ppm)

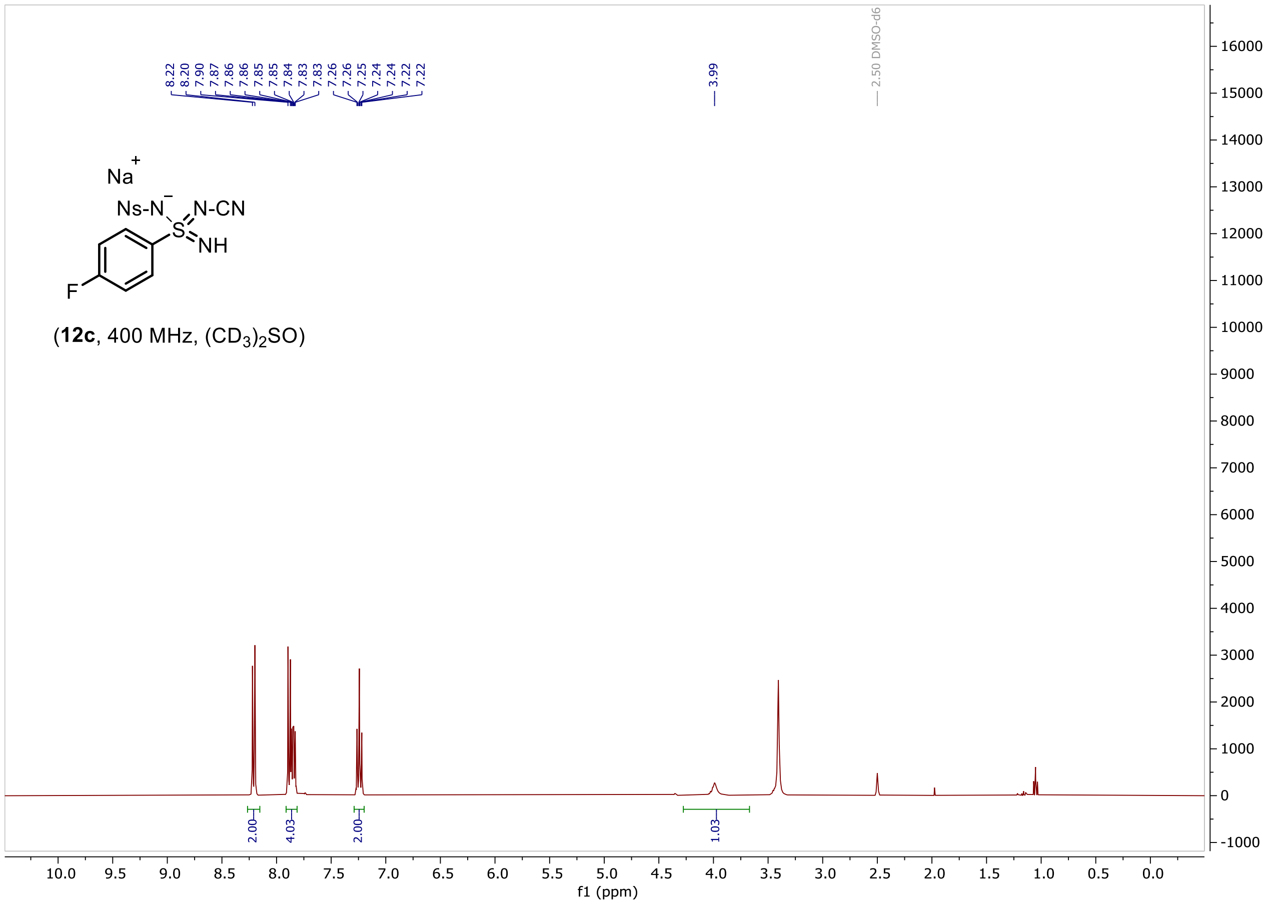

8.22 8.20 7.90 7.87 7.86 7.86 7.85 7.85 7.84 7.83 7.83 7.26 7.26 7.25 7.24 7.24 7.22 7.22
3.99
2.50 DMSO-d6
Na+
Ns-N− N-CN
S
NH
F
(**12c**, 400 MHz, $(CD_3)_2SO$)
2.00
4.03
2.00
1.03
f1 (ppm)

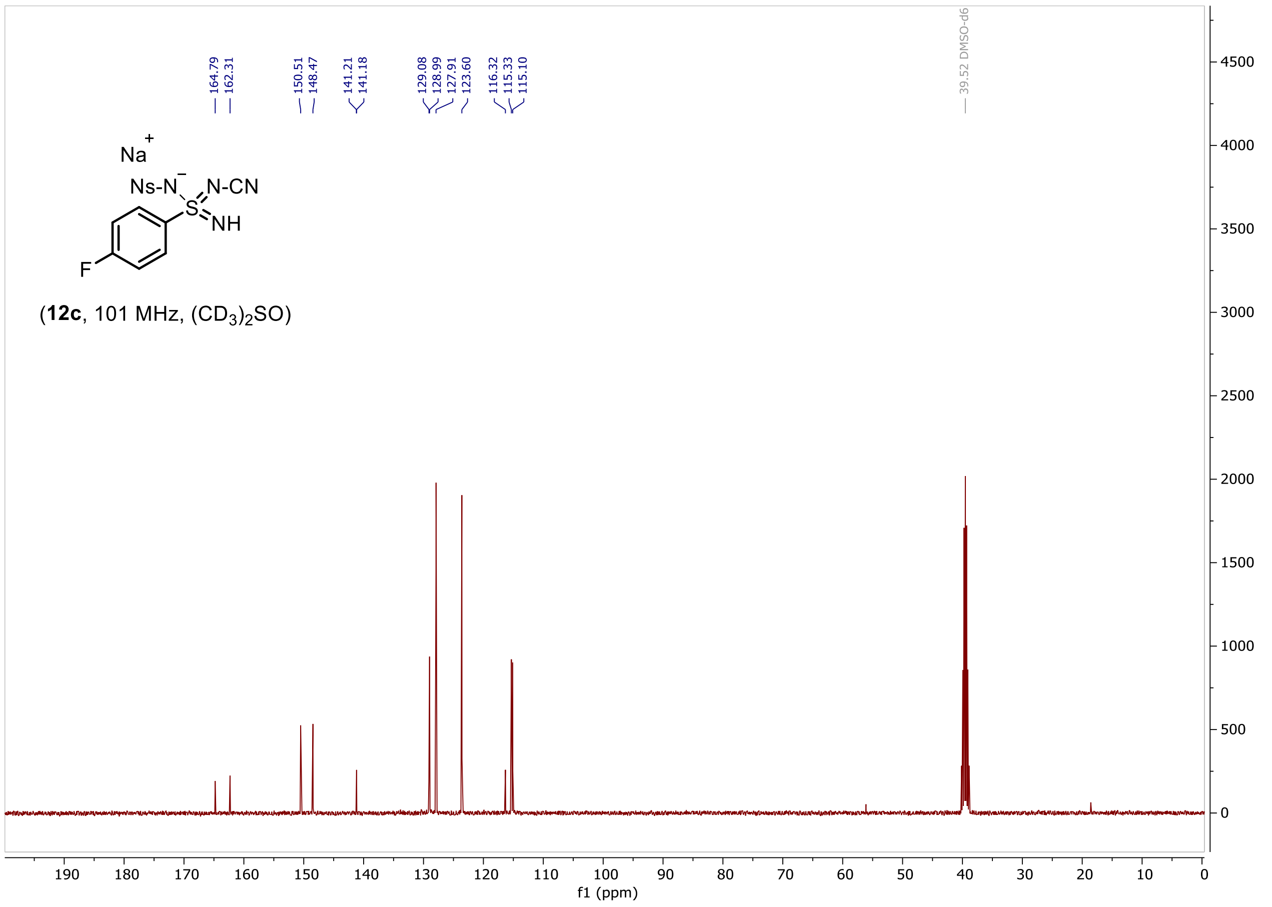

164.79 162.31
150.51 148.47
141.21 141.18
129.08 128.99 127.91 123.60
116.32 115.33 115.10
39.52 DMSO-d6
Na+
Ns-N− N-CN
S
NH
F
(**12c**, 101 MHz, $(CD_3)_2SO$)
f1 (ppm)

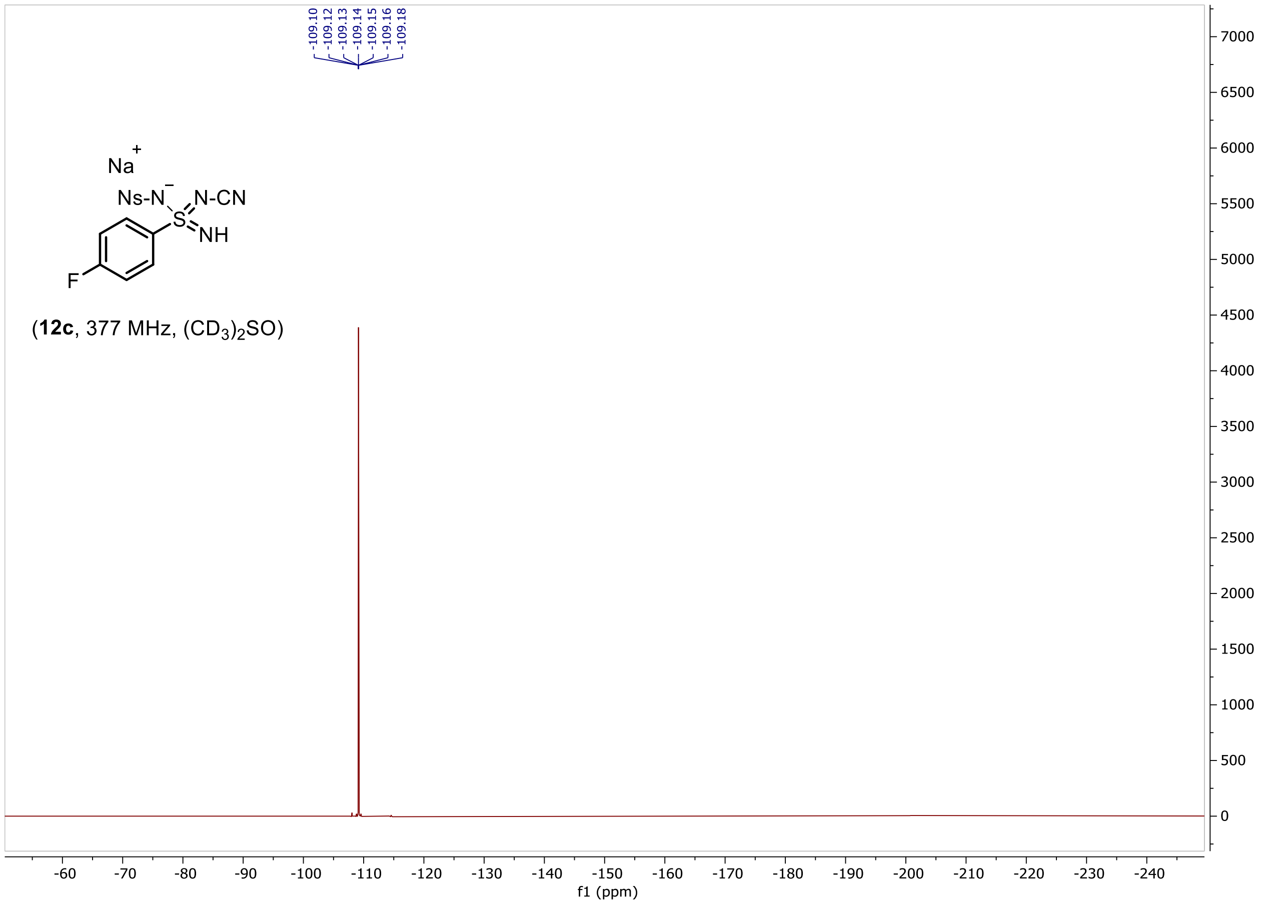

-109.10
-109.12
-109.13
-109.14
-109.15
-109.16
-109.18
Na+
Ns-N
N-CN
S
NH
F
(12c, 377 MHz, (CD3)2SO)
f1 (ppm)

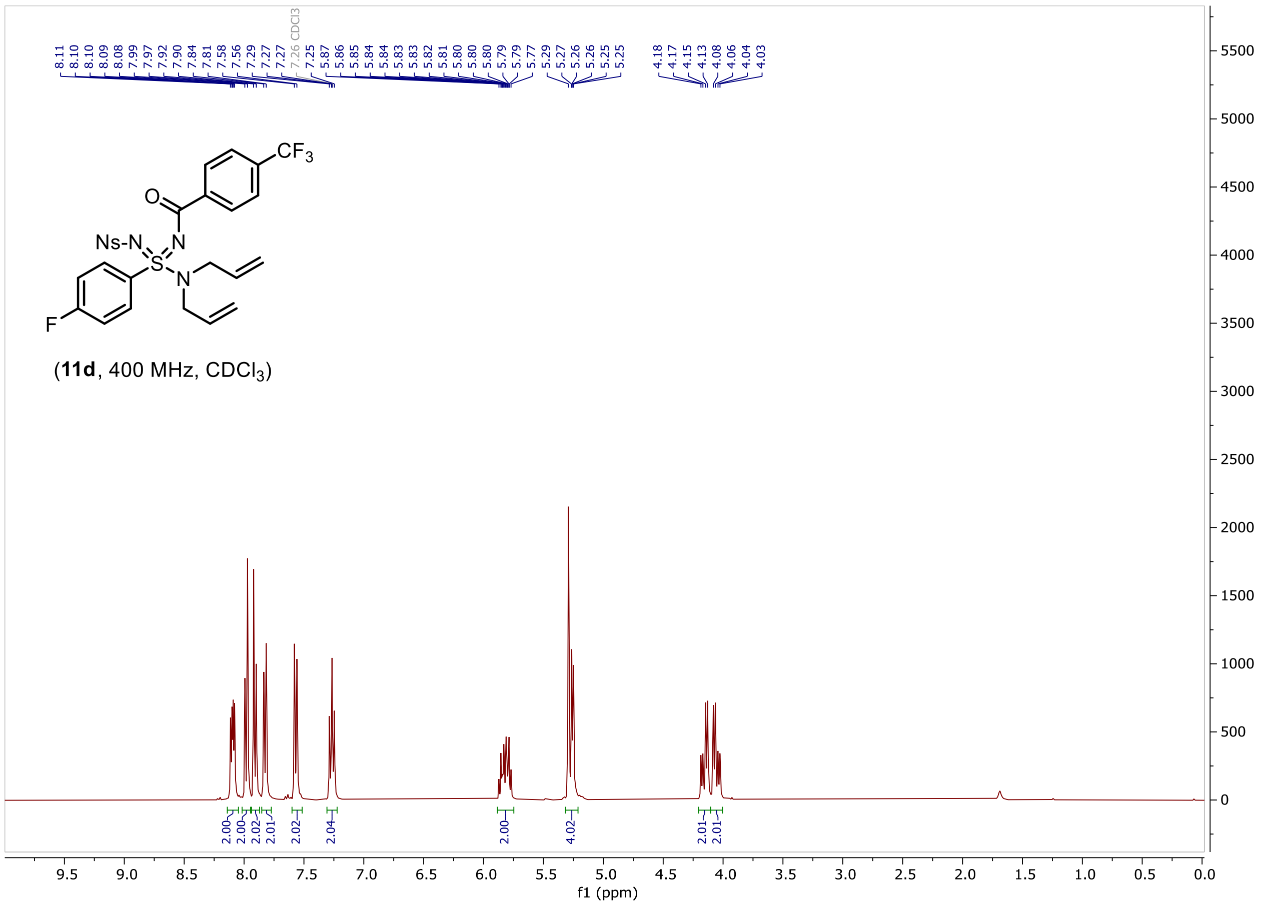

CDCl3
CF3
O
Ns-N
N
S
N
F
(11d, 400 MHz, CDCl3)
f1 (ppm)

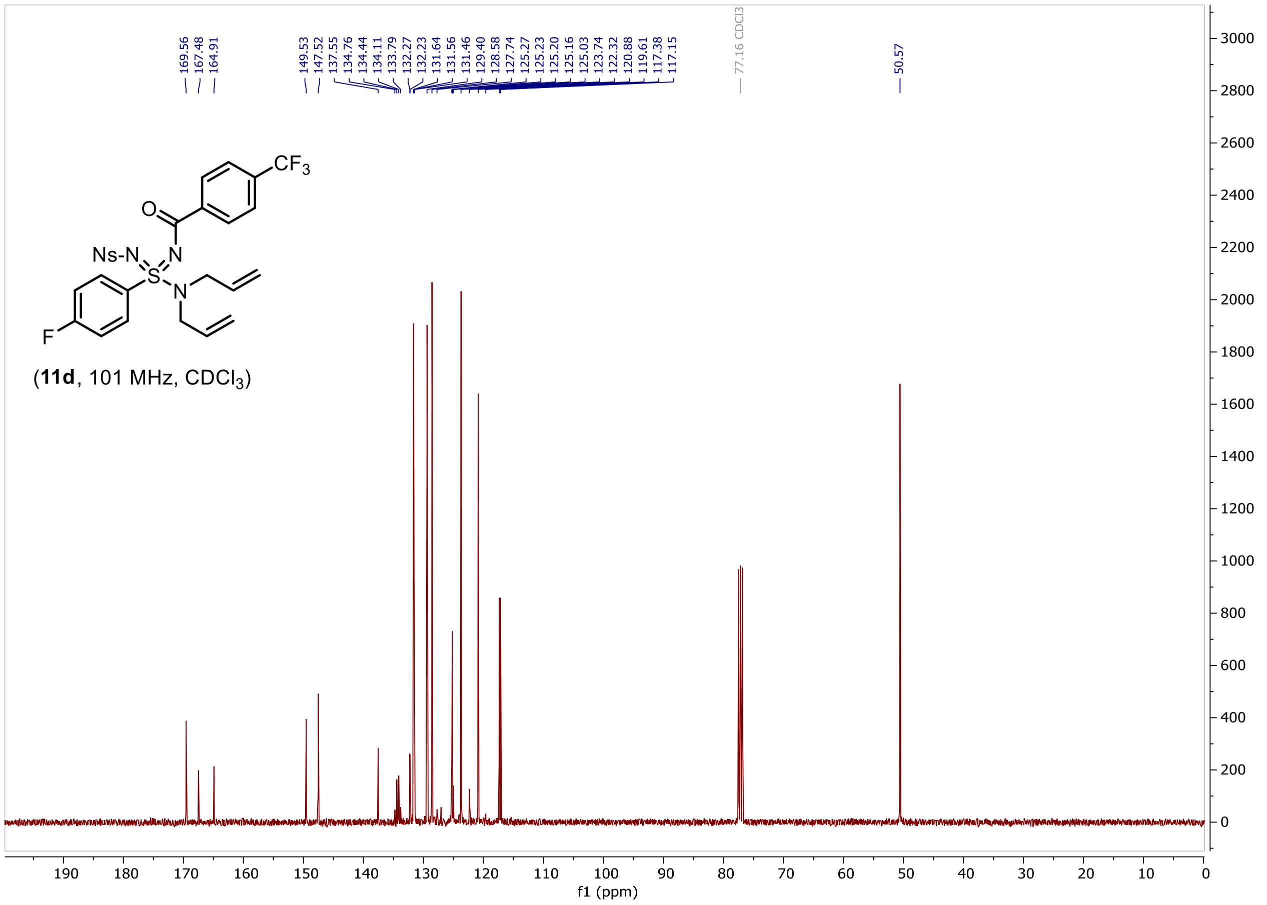
169.56
167.48
164.91
149.53
147.52
137.55
134.76
134.44
134.11
133.79
132.27
132.23
131.64
131.56
131.46
129.40
128.58
127.74
125.27
125.23
125.20
125.16
125.03
123.74
122.32
120.88
119.61
117.38
117.15
77.16 CDCl3
50.57
CF3
O
N
Ns-N
S
N
F
(11d, 101 MHz, CDCl3)
f1 (ppm)

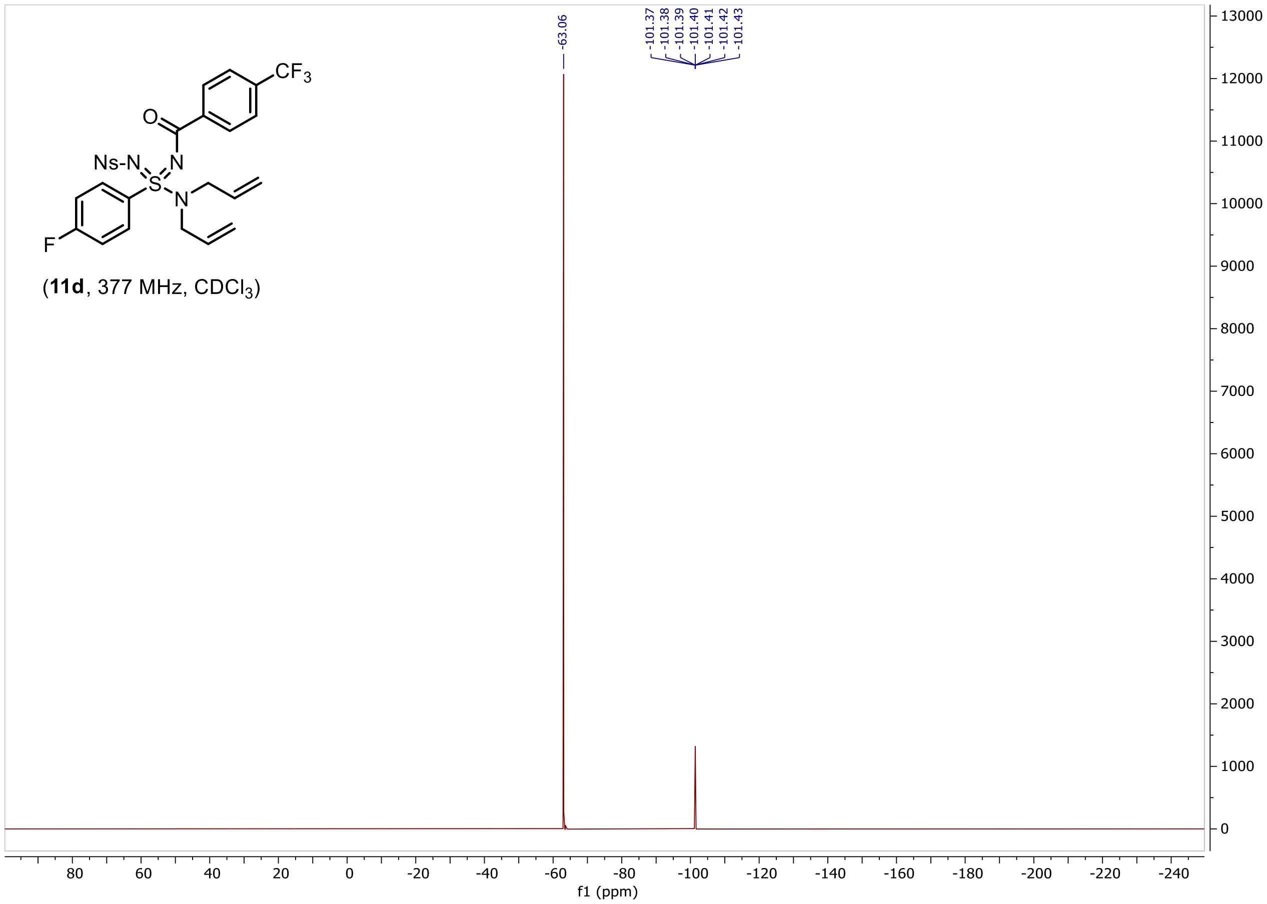
-63.06
-101.37
-101.38
-101.39
-101.40
-101.41
-101.42
-101.43
CF3
O
N
Ns-N
S
N
F
(11d, 377 MHz, CDCl3)
f1 (ppm)

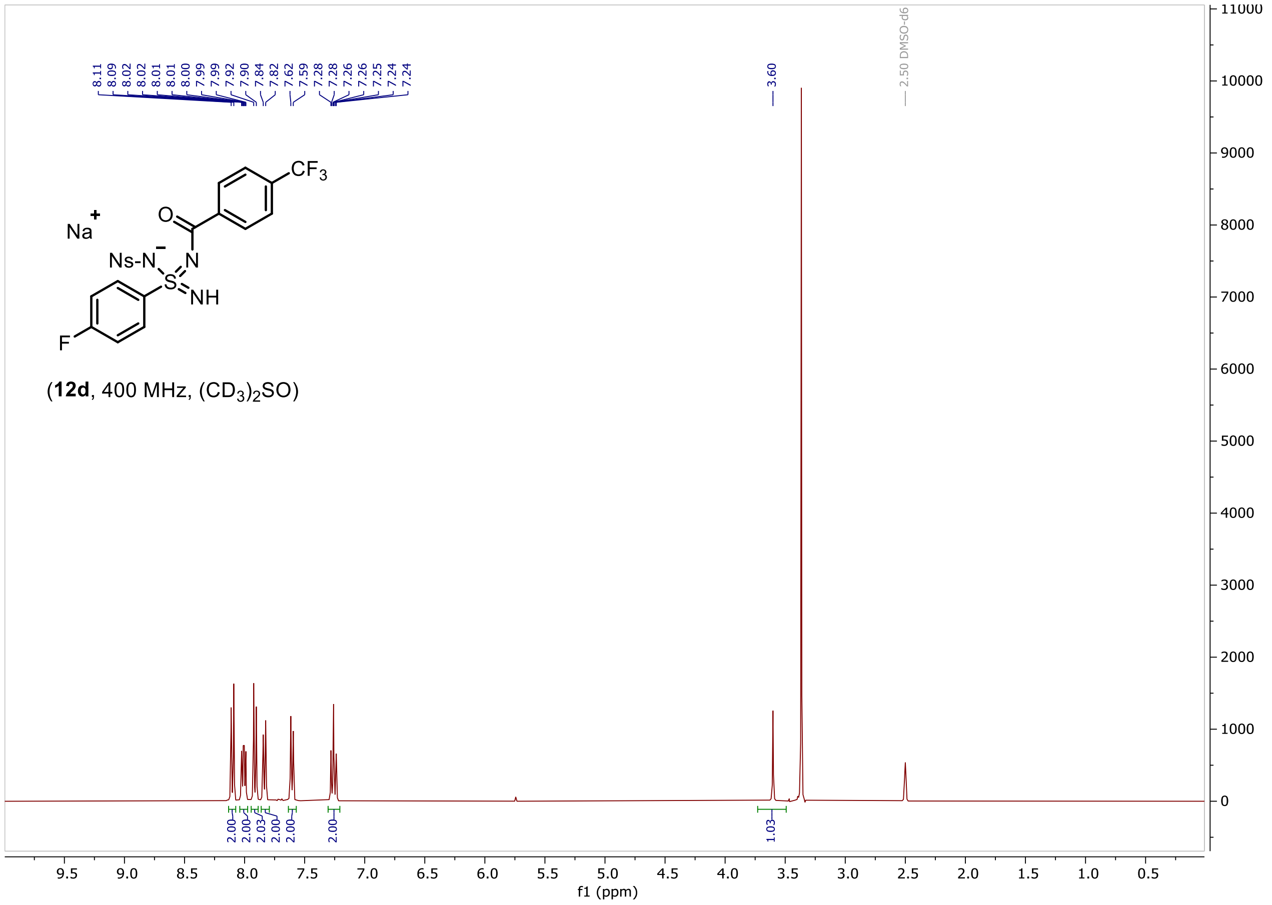

8.11
8.09
8.02
8.02
8.01
8.01
8.00
7.99
7.99
7.92
7.90
7.84
7.82
7.62
7.59
7.28
7.28
7.26
7.26
7.25
7.24
7.24
3.60
2.50 DMSO-d6
CF3
O
Na+
Ns-N
N
S
NH
F
(12d, 400 MHz, (CD3)2SO)
2.00
2.00
2.03
2.00
2.00
2.00
1.03
f1 (ppm)

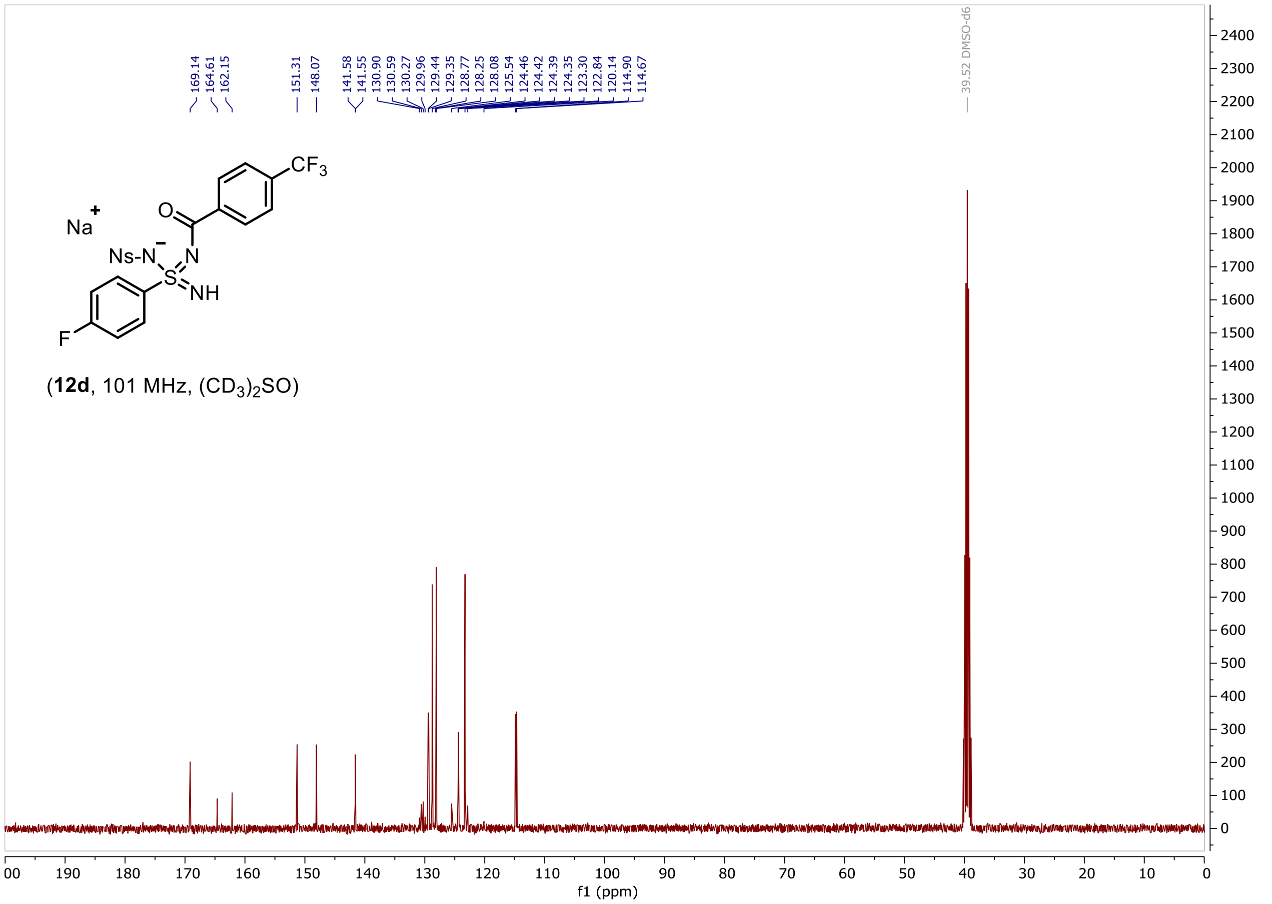

169.14
164.61
162.15
151.31
148.07
141.58
141.55
130.90
130.59
130.27
129.96
129.44
129.35
128.77
128.25
128.08
125.54
124.46
124.42
124.39
124.35
123.30
122.84
120.14
114.90
114.67
39.52 DMSO-d6
CF3
O
Na+
Ns-N
N
S
NH
F
(12d, 101 MHz, (CD3)2SO)
f1 (ppm)

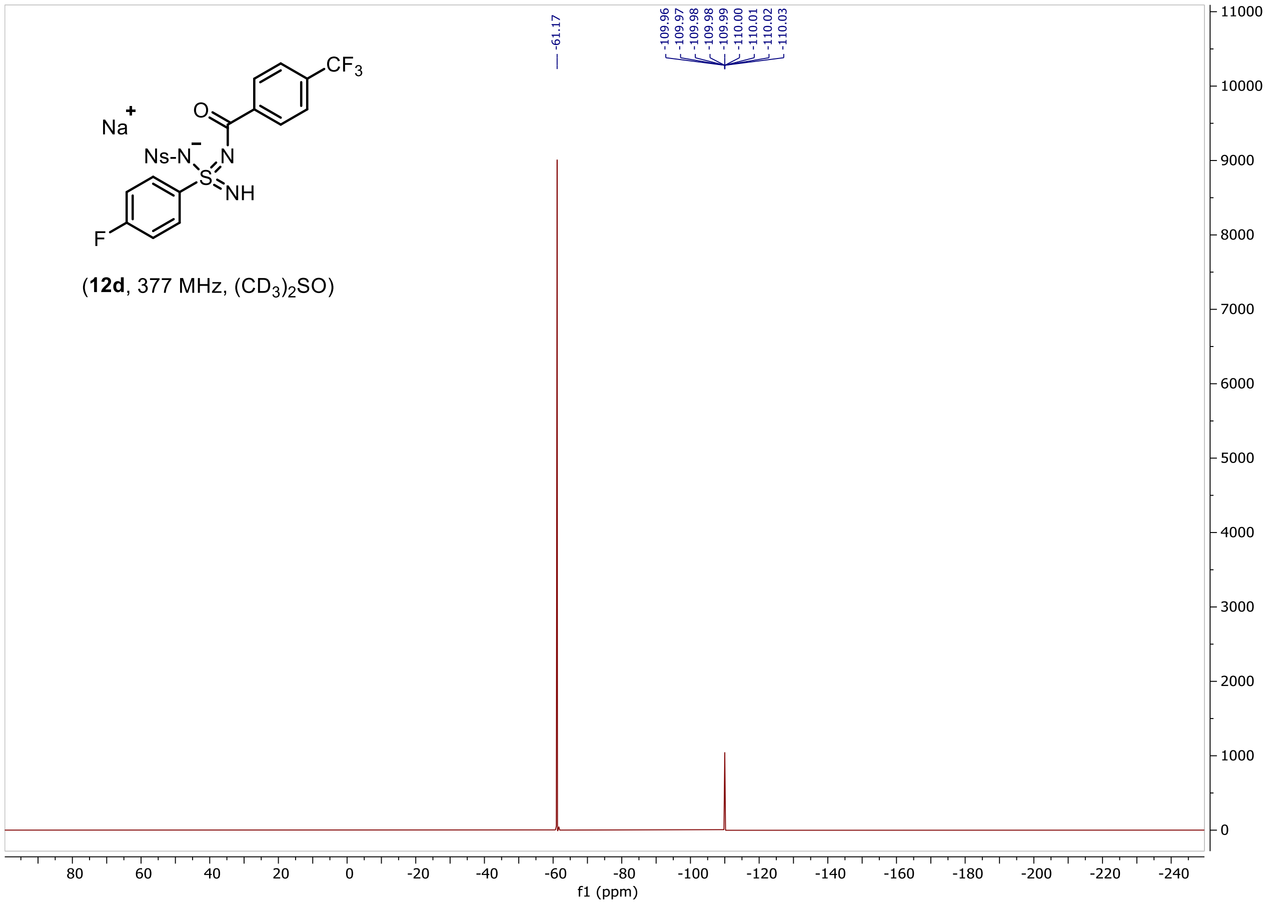
Na+
Ns-N
O
CF3
N
S
NH
F
(12d, 377 MHz, (CD3)2SO)
-61.17
-109.96
-109.97
-109.98
-109.98
-109.99
-110.00
-110.01
-110.02
-110.03
f1 (ppm)

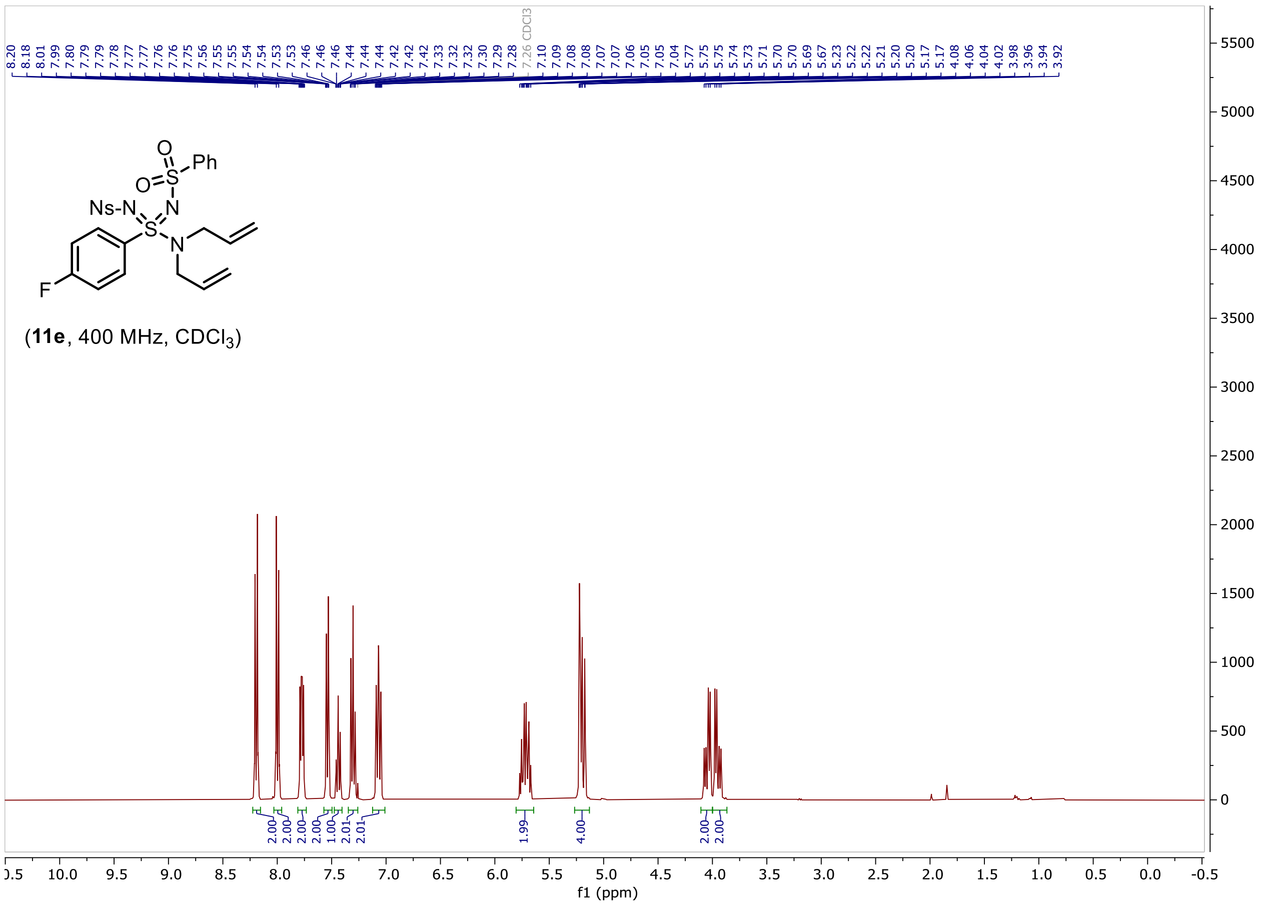
O
Ph
O
S
Ns-N
N
S
N
F
(11e, 400 MHz, CDCl3)
7.26 CDCl3
f1 (ppm)

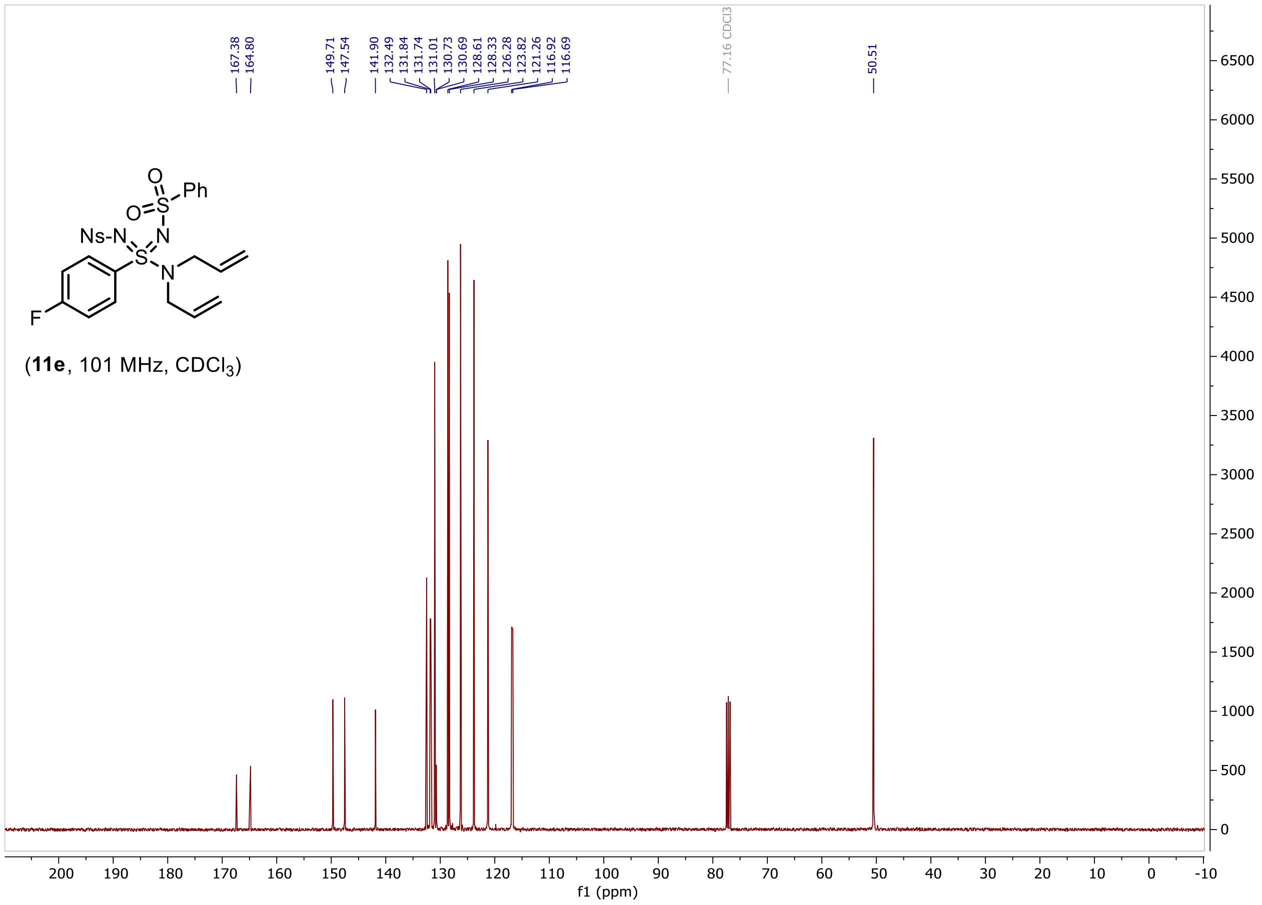

($\mathbf{11e}$, 101 MHz, $CDCl_3$)

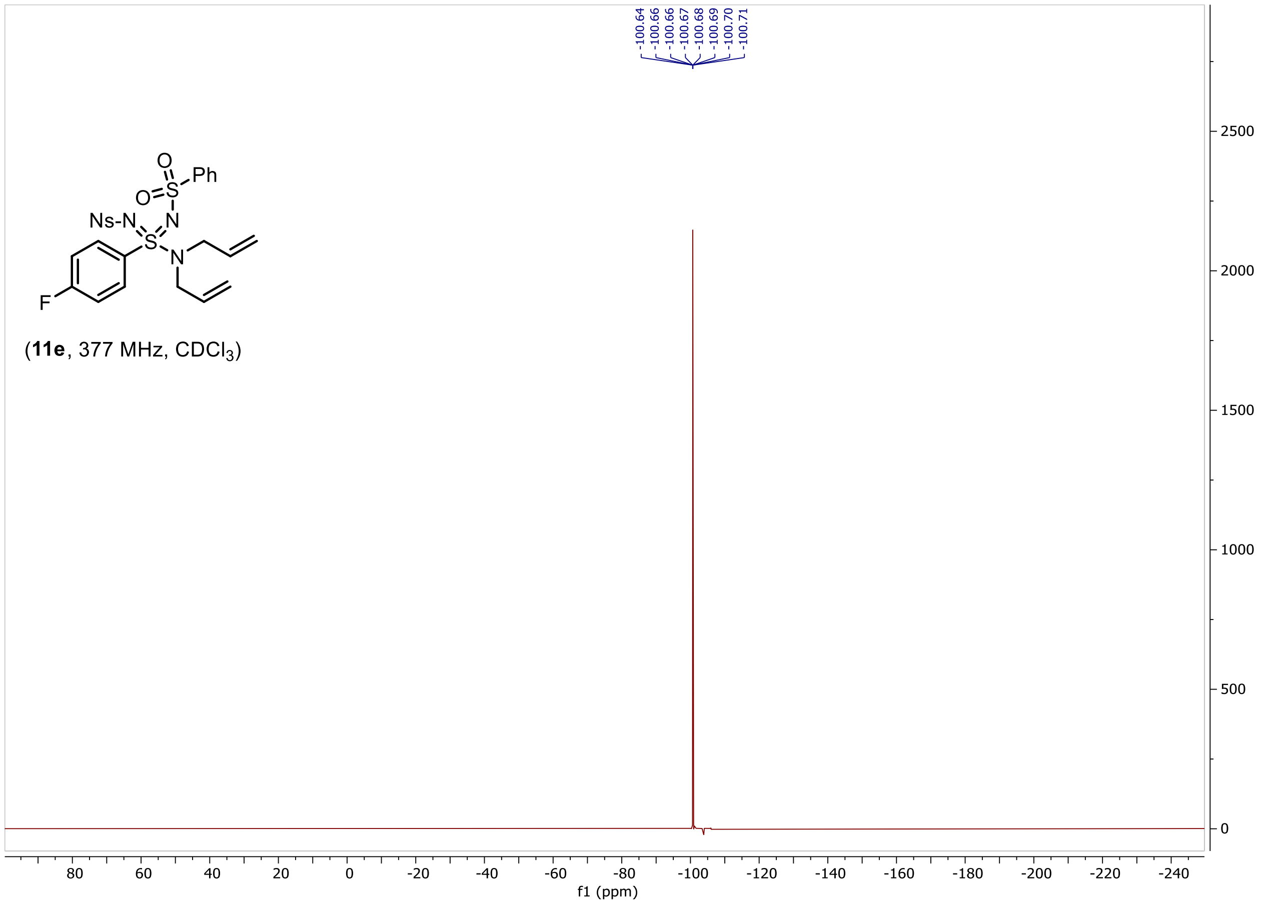

($\mathbf{11e}$, 377 MHz, $CDCl_3$)

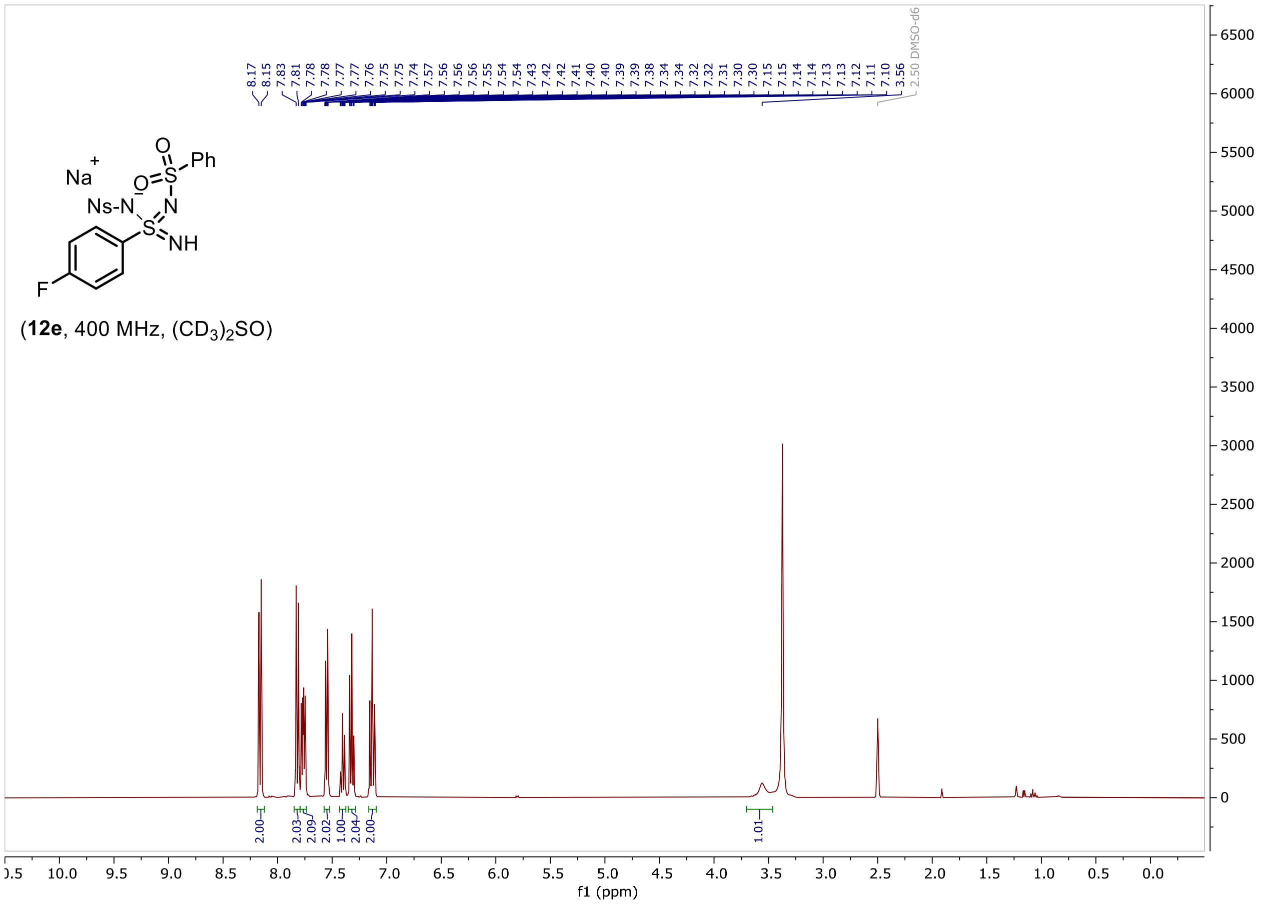
8.17
8.15
7.83
7.81
7.78
7.78
7.77
7.77
7.76
7.75
7.75
7.74
7.57
7.56
7.56
7.56
7.55
7.54
7.54
7.43
7.42
7.42
7.41
7.40
7.40
7.39
7.39
7.38
7.34
7.34
7.32
7.32
7.31
7.30
7.30
7.15
7.15
7.14
7.14
7.13
7.13
7.12
7.11
7.10
3.56
2.50 DMSO-d6
Na+
O
Ph
O
S
Ns-N
N
S
NH
F
(12e, 400 MHz, (CD3)2SO)
2.00
2.03
2.09
2.02
1.00
2.04
2.00
1.01
f1 (ppm)

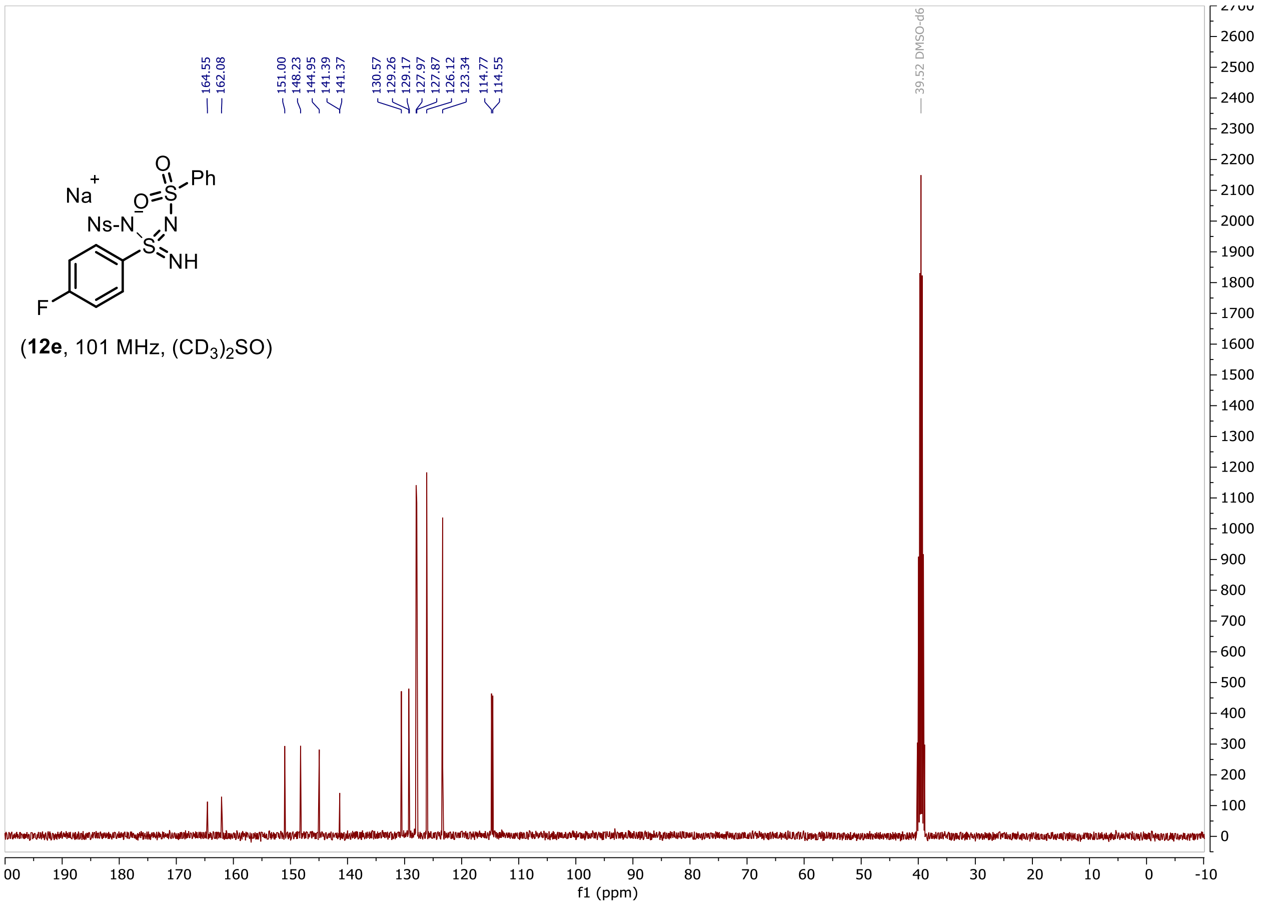
164.55
162.08
151.00
148.23
144.95
141.39
141.37
130.57
129.26
129.17
127.97
127.87
126.12
123.34
114.77
114.55
39.52 DMSO-d6
Na+
O
Ph
O
S
Ns-N
N
S
NH
F
(12e, 101 MHz, (CD3)2SO)
f1 (ppm)

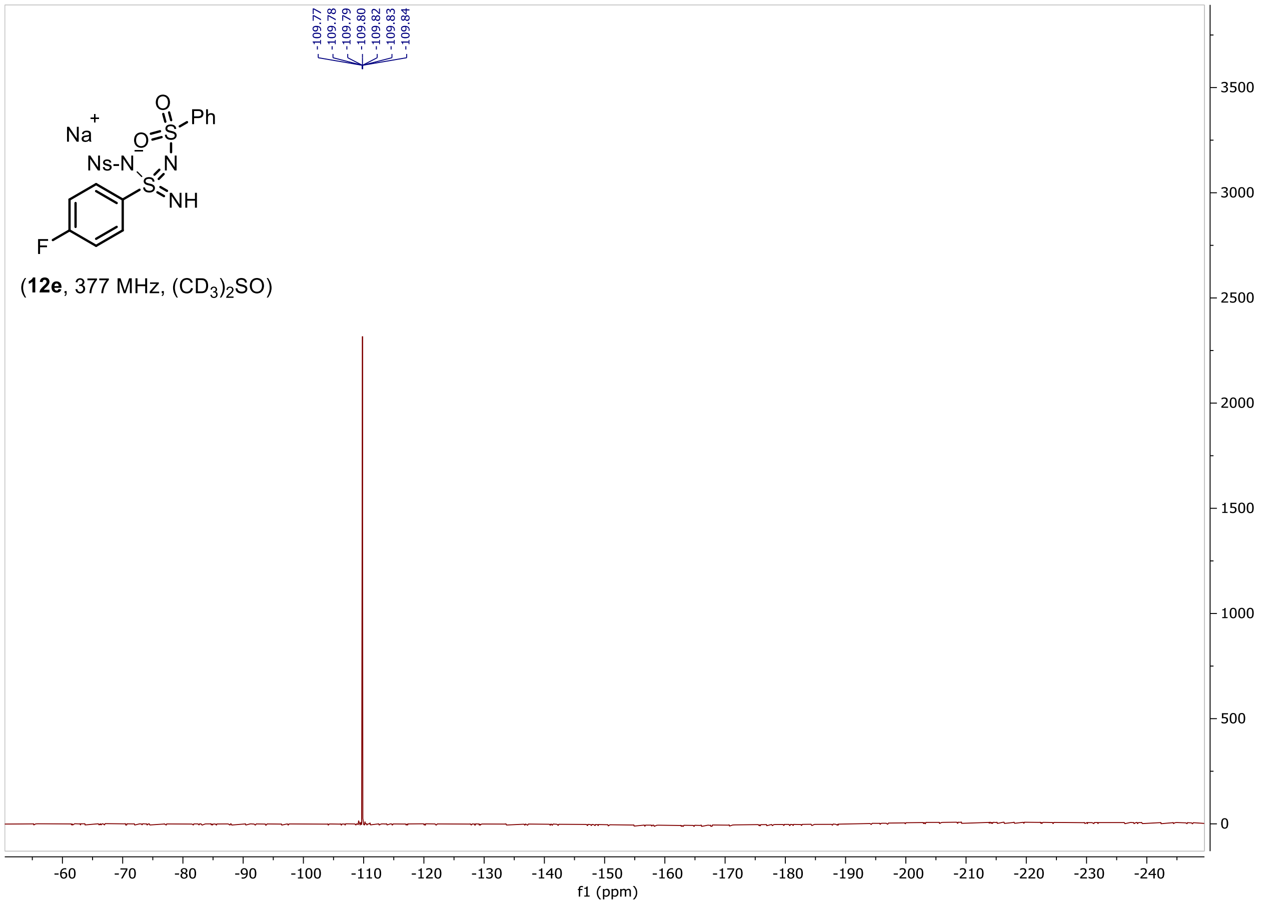

-109.77
-109.78
-109.79
-109.80
-109.82
-109.83
-109.84
Na+
O
Ph
S
O
N
Ns-N
S
NH
F
(12e, 377 MHz, (CD3)2SO)
f1 (ppm)

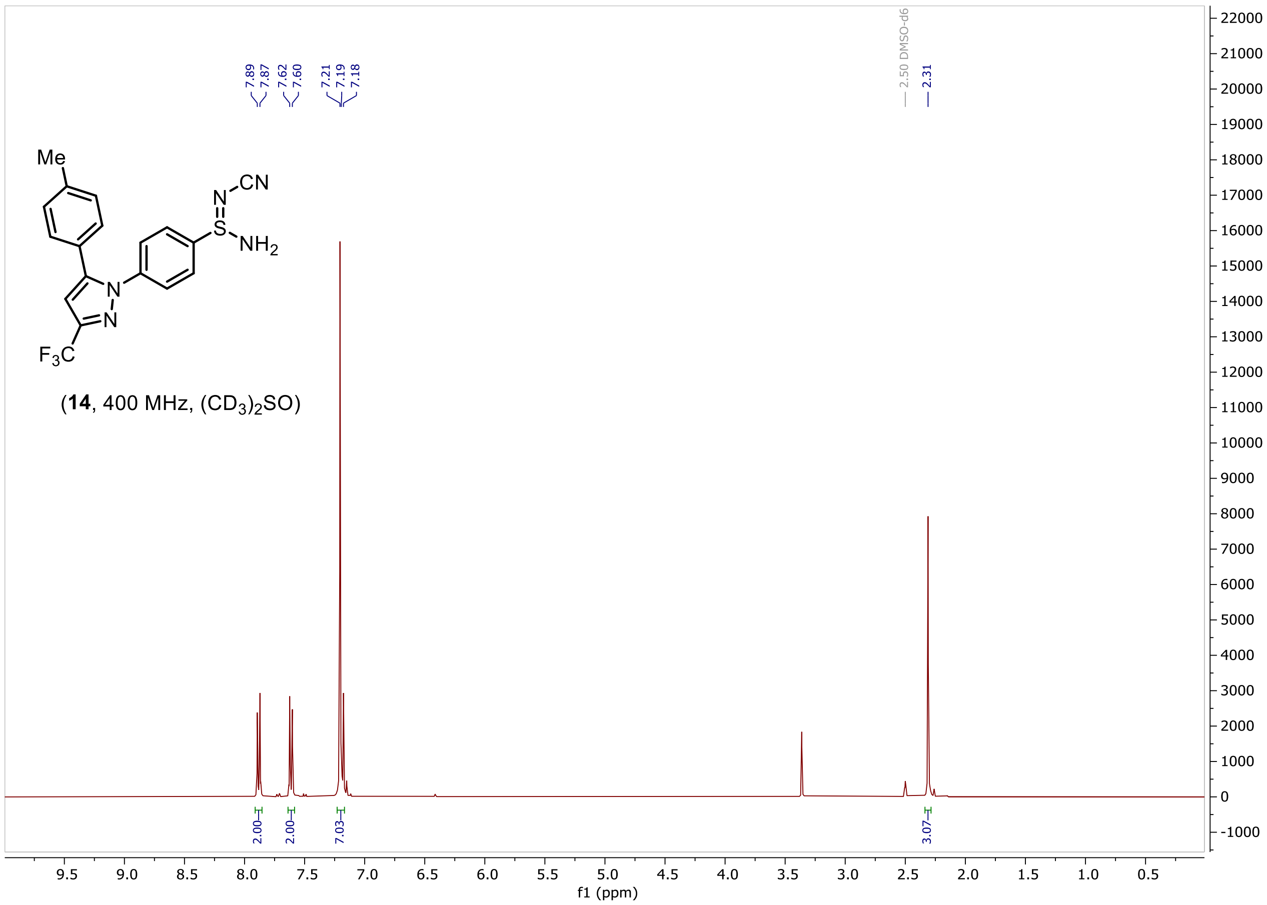

7.89
7.87
7.62
7.60
7.21
7.19
7.18
2.50 DMSO-d6
2.31
Me
N
CN
S
NH2
N
N
F3C
(14, 400 MHz, (CD3)2SO)
2.00
2.00
7.03
3.07
f1 (ppm)

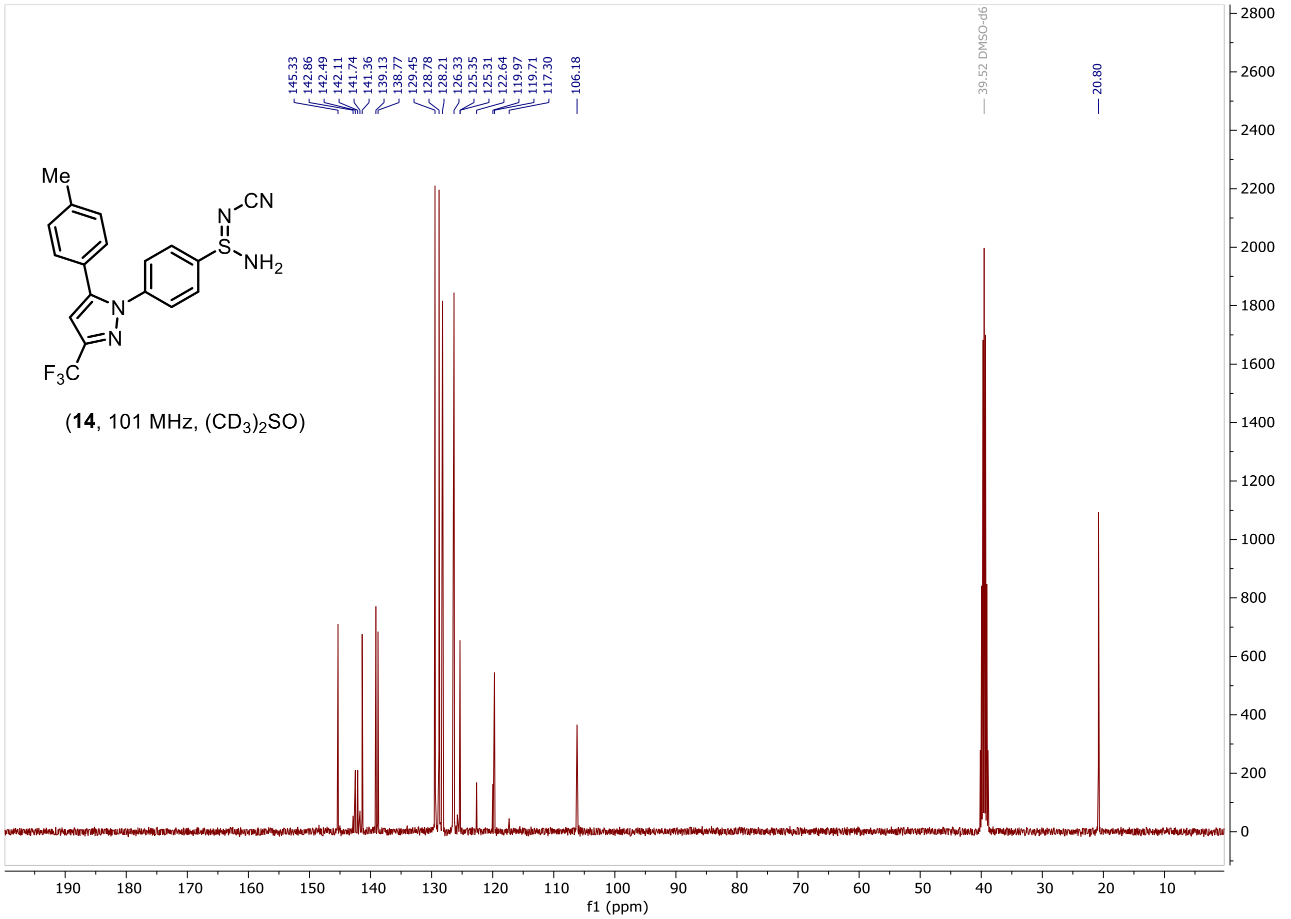
145.33
142.86
142.49
142.11
141.74
141.36
139.13
138.77
129.45
128.78
128.21
126.33
125.35
125.31
122.64
119.97
119.71
117.30
106.18
39.52 DMSO-d6
20.80
Me
N
CN
S
NH2
N
N
F3C
(14, 101 MHz, (CD3)2SO)
f1 (ppm)

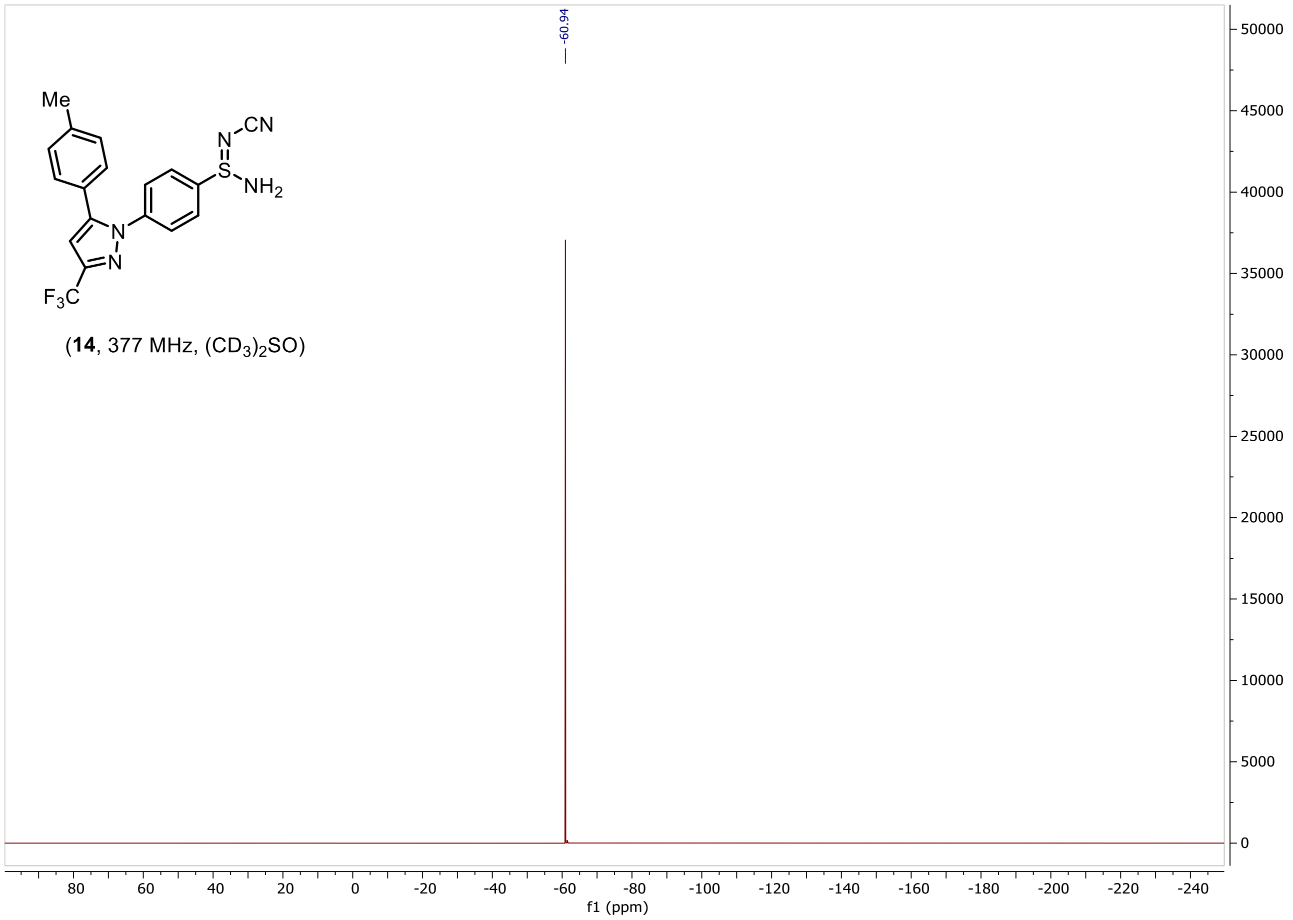
-60.94
Me
N
CN
S
NH2
N
N
F3C
(14, 377 MHz, (CD3)2SO)
f1 (ppm)

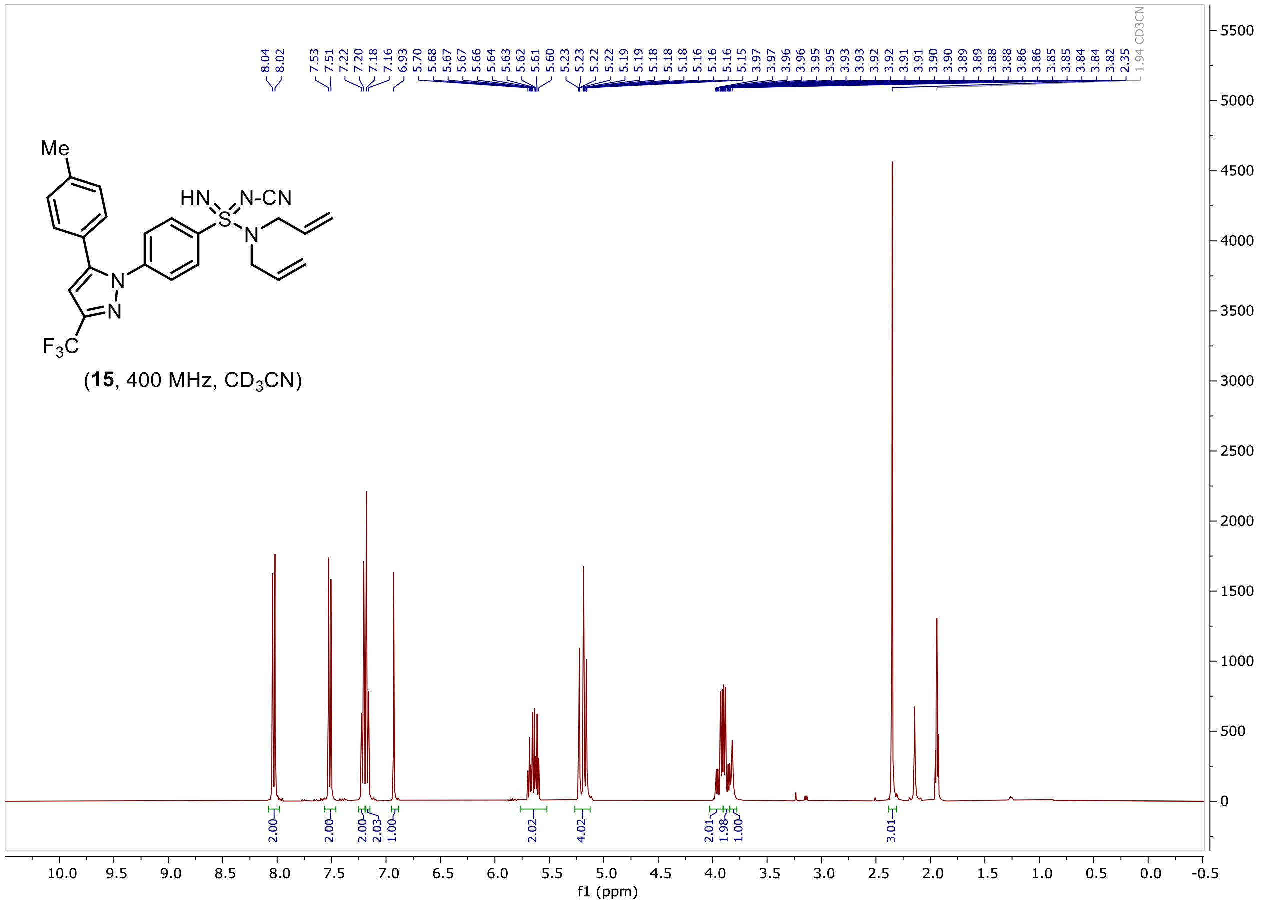
Me
HN
N-CN
S
N
N
N
F_3C
(**15**, 400 MHz, CD_3CN)
f1 (ppm)

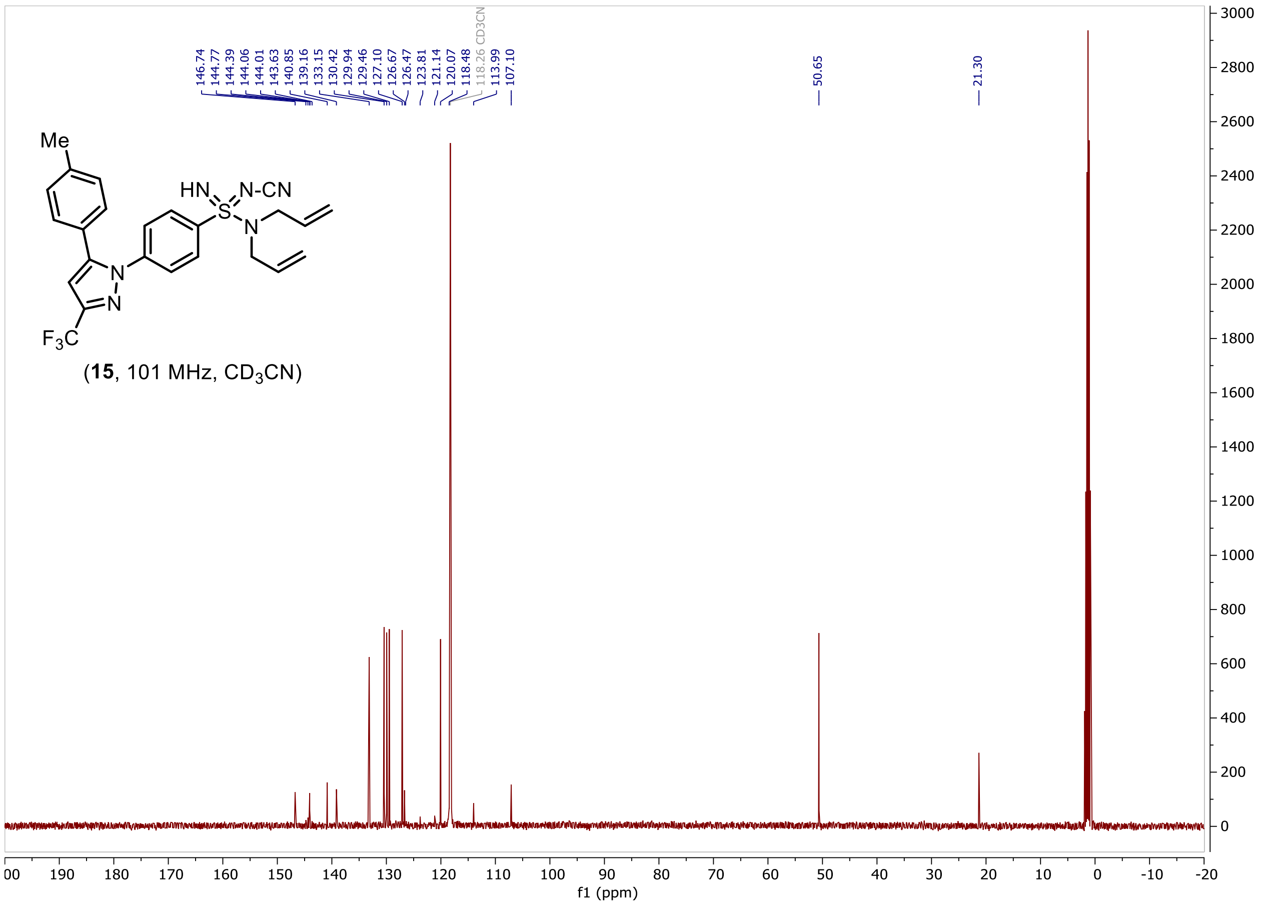
Me
HN
N-CN
S
N
N
N
F_3C
(**15**, 101 MHz, CD_3CN)
146.74 144.77 144.39 144.06 144.01 143.63 140.85 139.16 133.15 130.42 129.94 129.46 127.10 126.67 126.47 123.81 121.14 120.07 118.48 118.26 CD3CN 113.99 107.10
50.65
21.30
f1 (ppm)

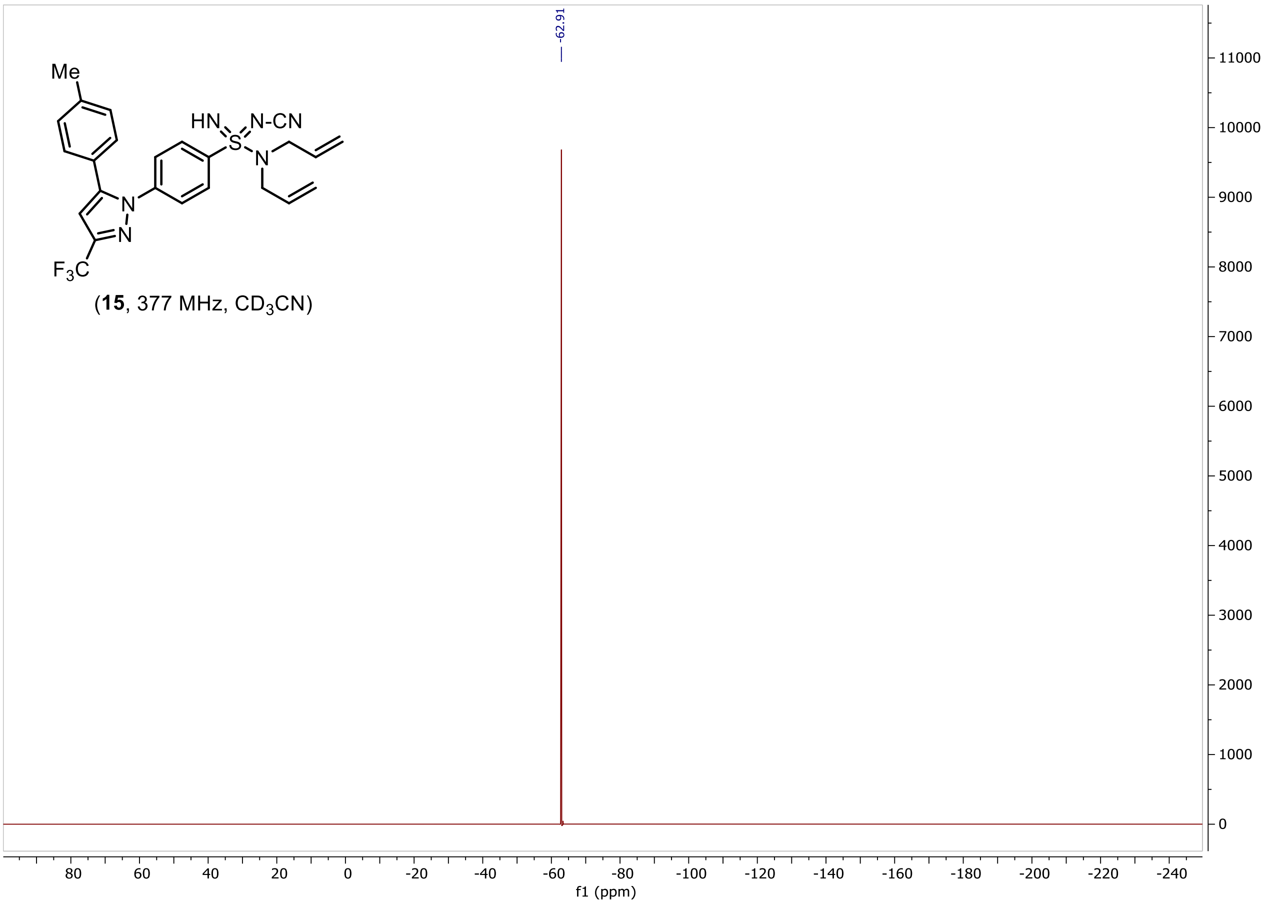

Me
HN
N-CN
S
N
N
N
F_3C
(15, 377 MHz, CD_3CN)
-62.91
f1 (ppm)

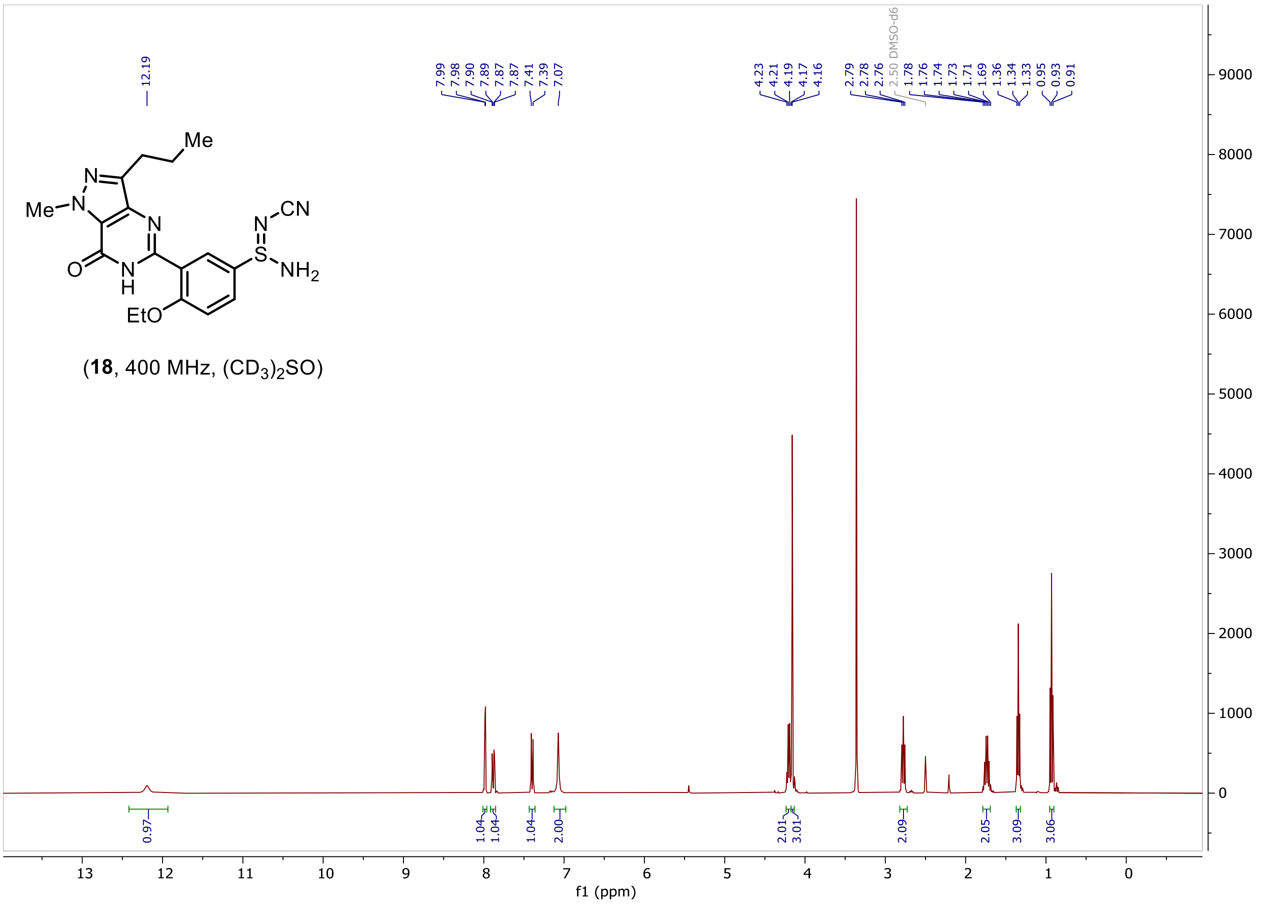

Me
N
Me
N
N
O
N
H
EtO
N
CN
S
NH_2
(18, 400 MHz, $(CD_3)_2SO$)
12.19
7.99 7.98 7.90 7.89 7.87 7.87 7.41 7.39 7.07
4.23 4.21 4.19 4.17 4.16
2.79 2.78 2.76 2.50 DMSO-d6 1.78 1.76 1.74 1.73 1.71 1.69 1.36 1.34 1.33 0.95 0.93 0.91
0.97 1.04 1.04 1.04 2.00 2.01 3.01 2.09 2.05 3.09 3.06
f1 (ppm)

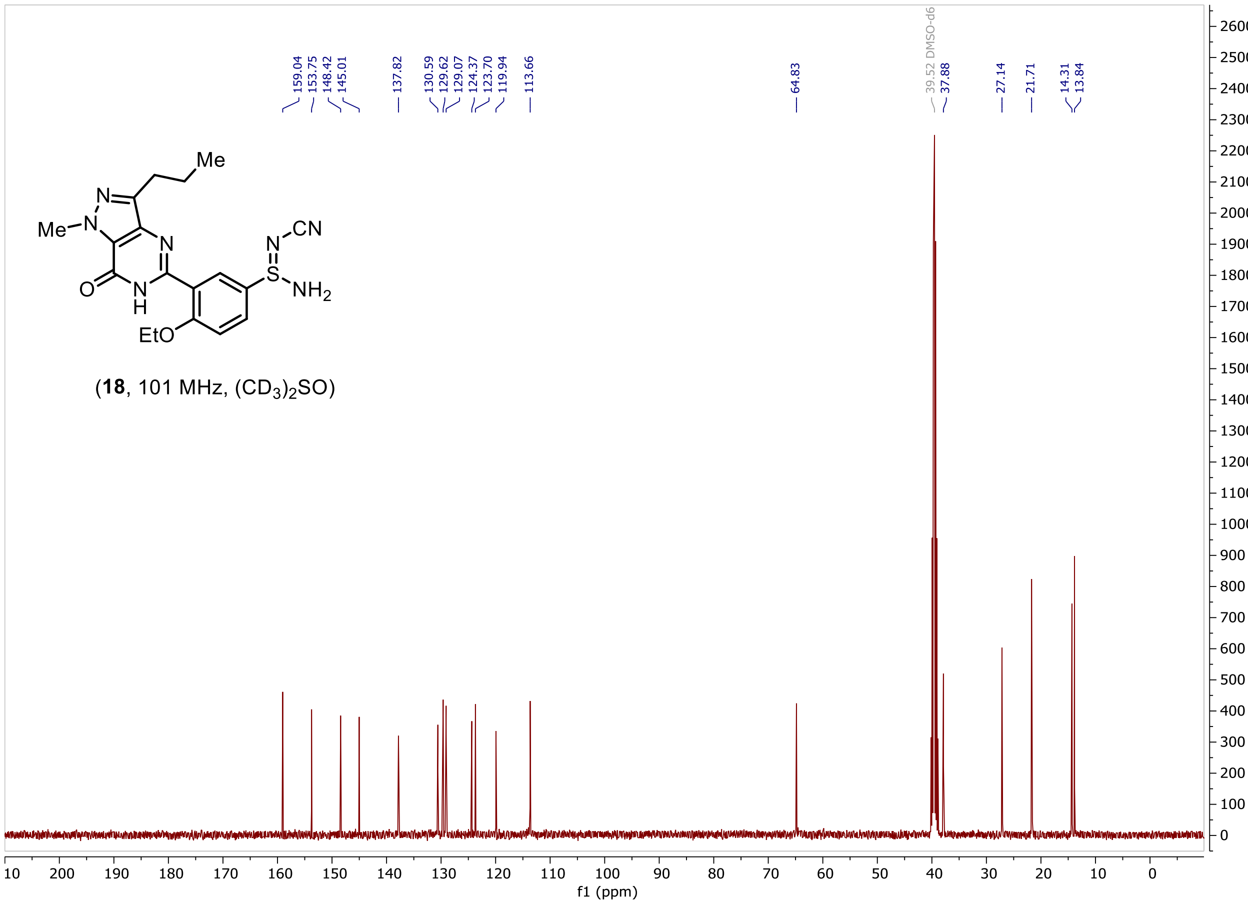

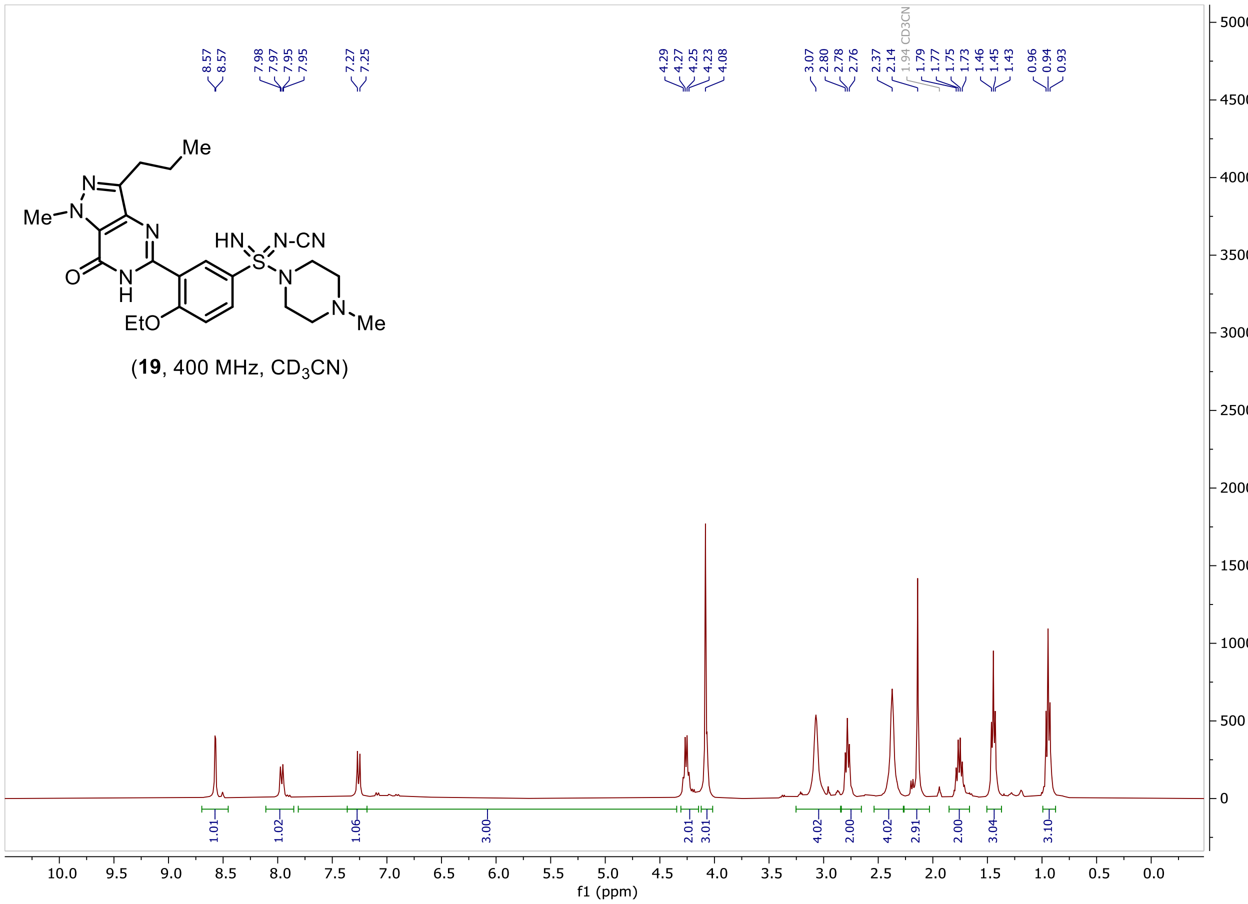

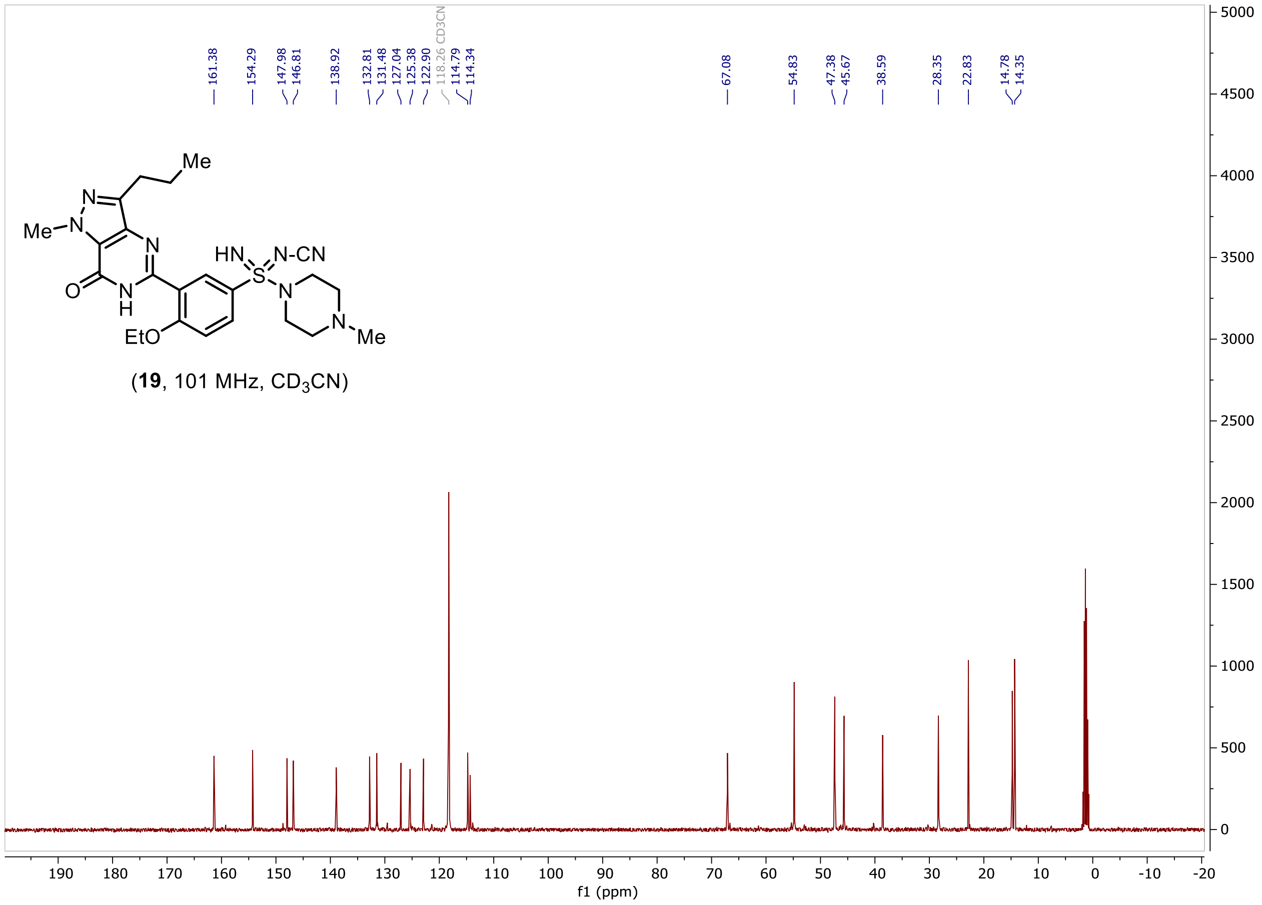
161.38
154.29
147.98
146.81
138.92
132.81
131.48
127.04
125.38
122.90
118.26 CD3CN
114.79
114.34
67.08
54.83
47.38
45.67
38.59
28.35
22.83
14.78
14.35
(19, 101 MHz, CD3CN)
f1 (ppm)

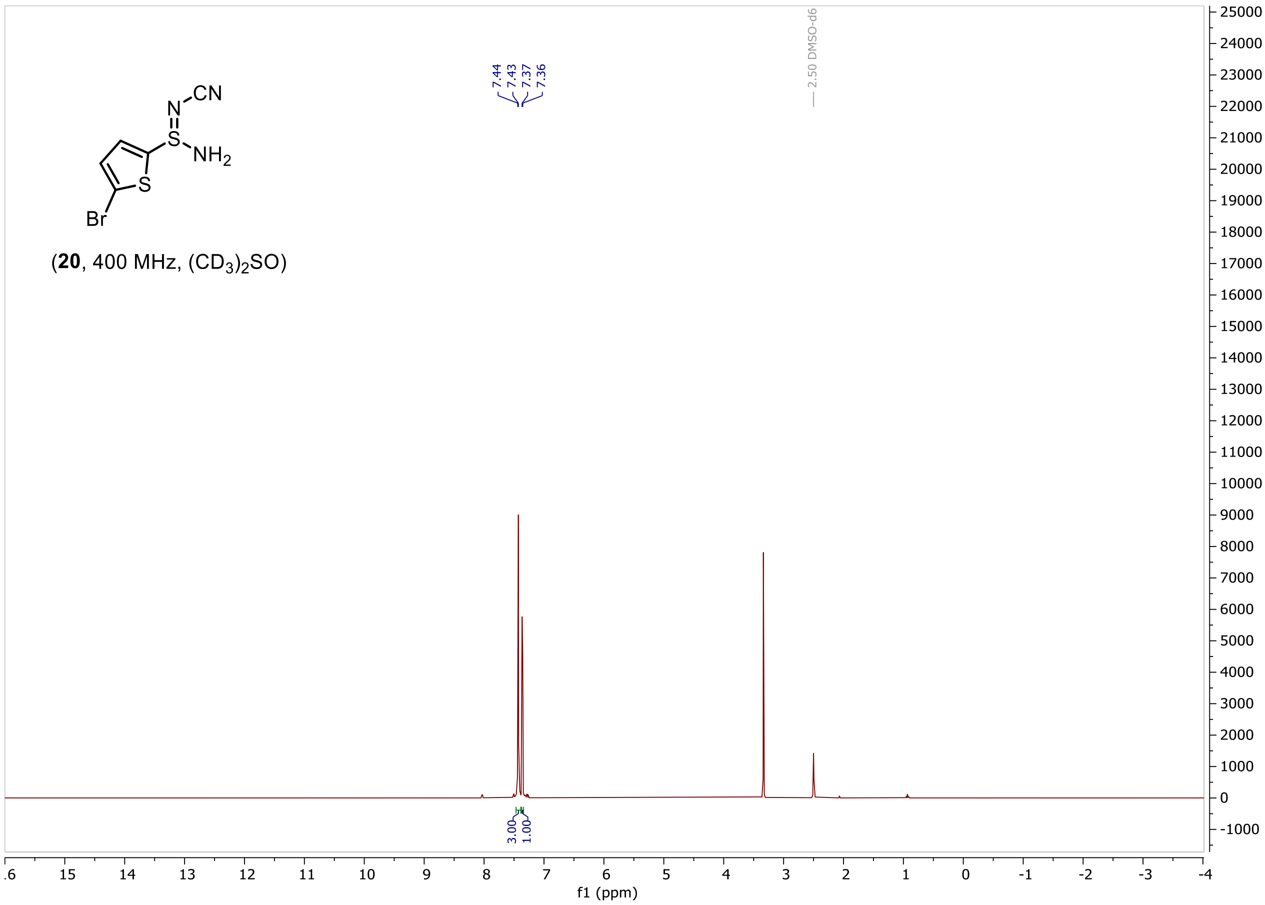
7.44
7.43
7.37
7.36
2.50 DMSO-d6
(20, 400 MHz, (CD3)2SO)
3.00
1.00
f1 (ppm)

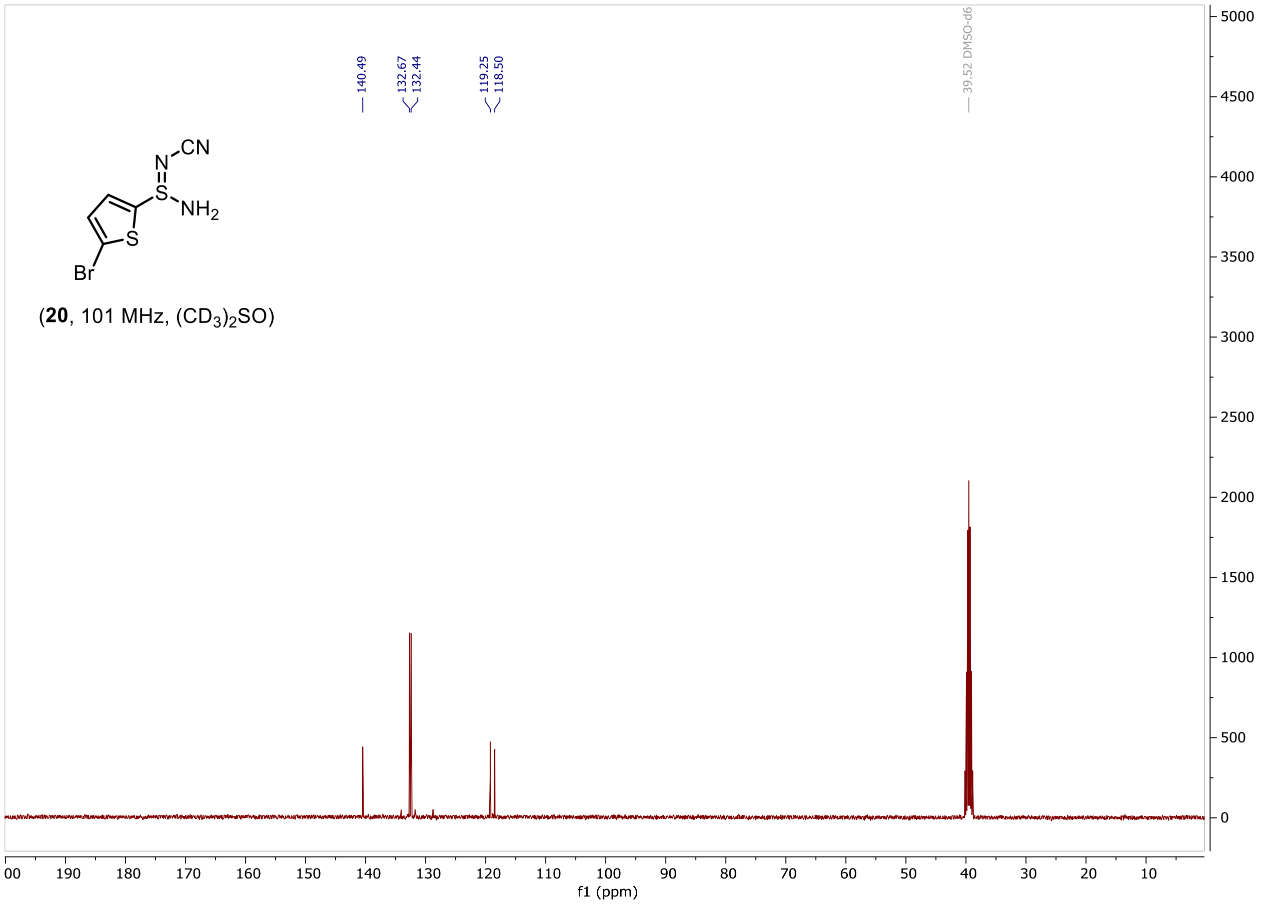

140.49
132.67
132.44
119.25
118.50
39.52 DMSO-d6
N-CN
S
NH2
S
Br
(20, 101 MHz, (CD3)2SO)
f1 (ppm)

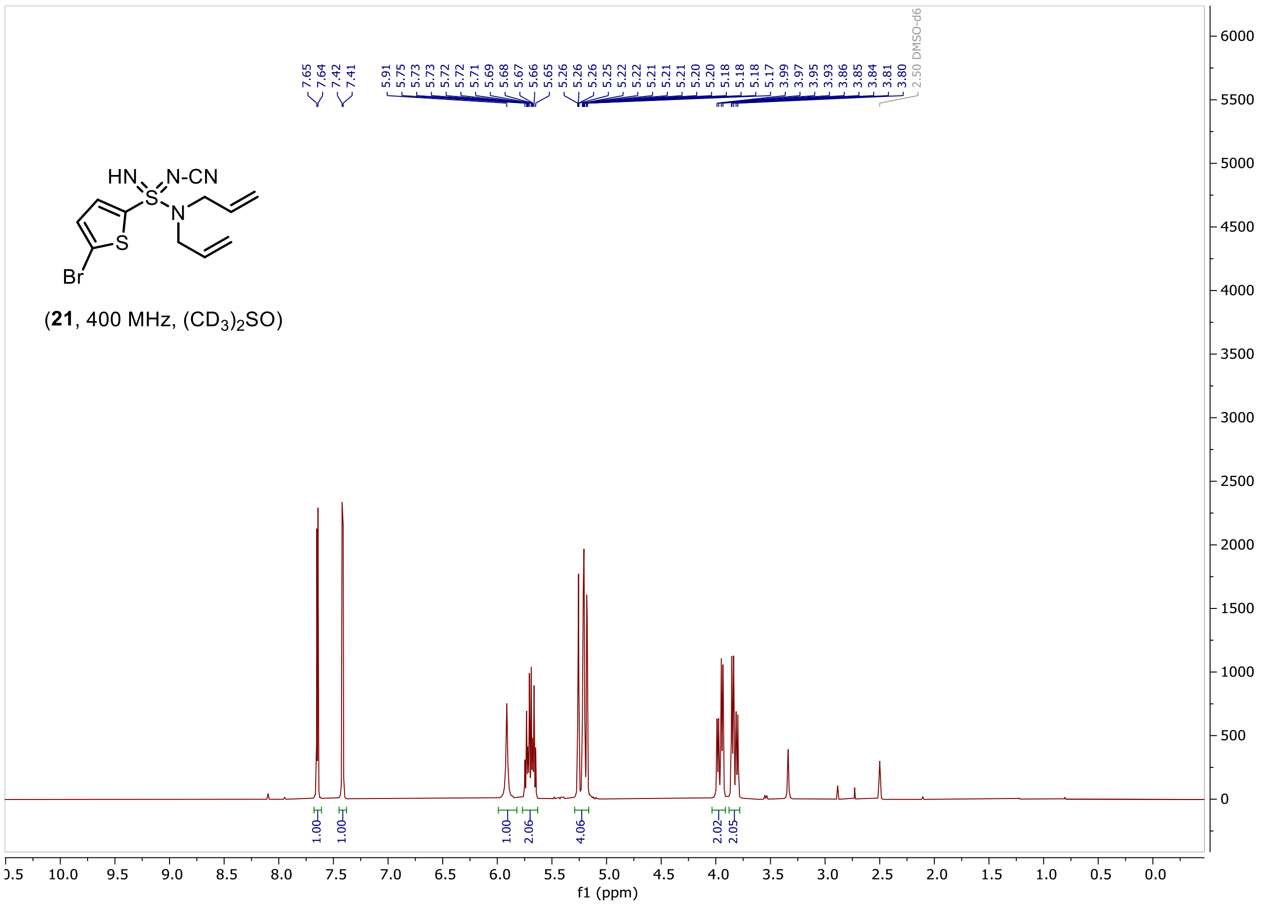

7.65
7.64
7.42
7.41
5.91
5.75
5.73
5.73
5.72
5.72
5.71
5.69
5.68
5.67
5.66
5.65
5.26
5.26
5.26
5.25
5.22
5.22
5.21
5.21
5.21
5.20
5.18
5.18
5.17
3.99
3.97
3.95
3.93
3.86
3.85
3.84
3.81
3.80
2.50 DMSO-d6
HN
N-CN
S
N
S
Br
(21, 400 MHz, (CD3)2SO)
1.00
1.00
1.00
2.06
4.06
2.02
2.05
f1 (ppm)

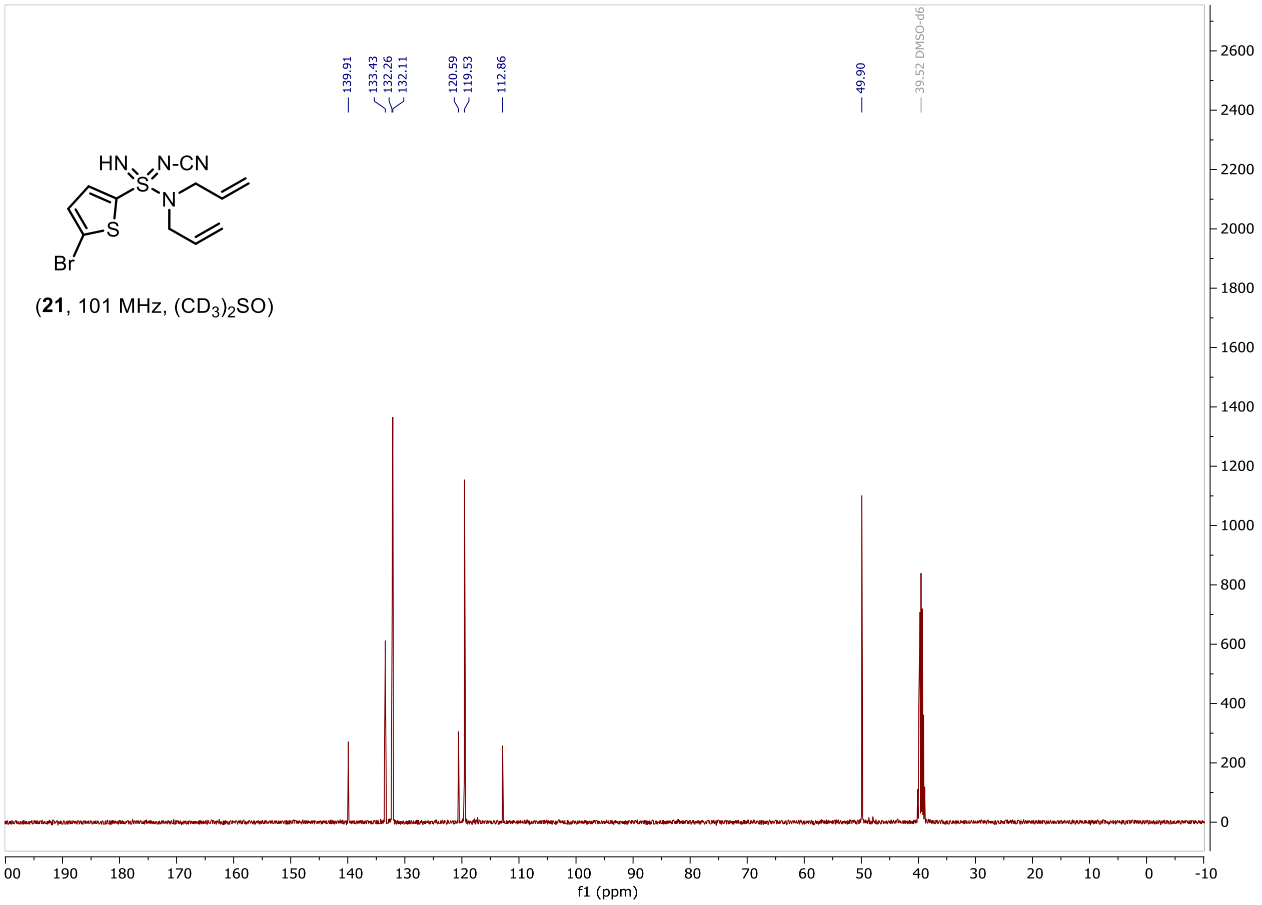

(21, 101 MHz, (CD3)2SO)
139.91
133.43
132.26
132.11
120.59
119.53
112.86
49.90
39.52 DMSO-d6
f1 (ppm)

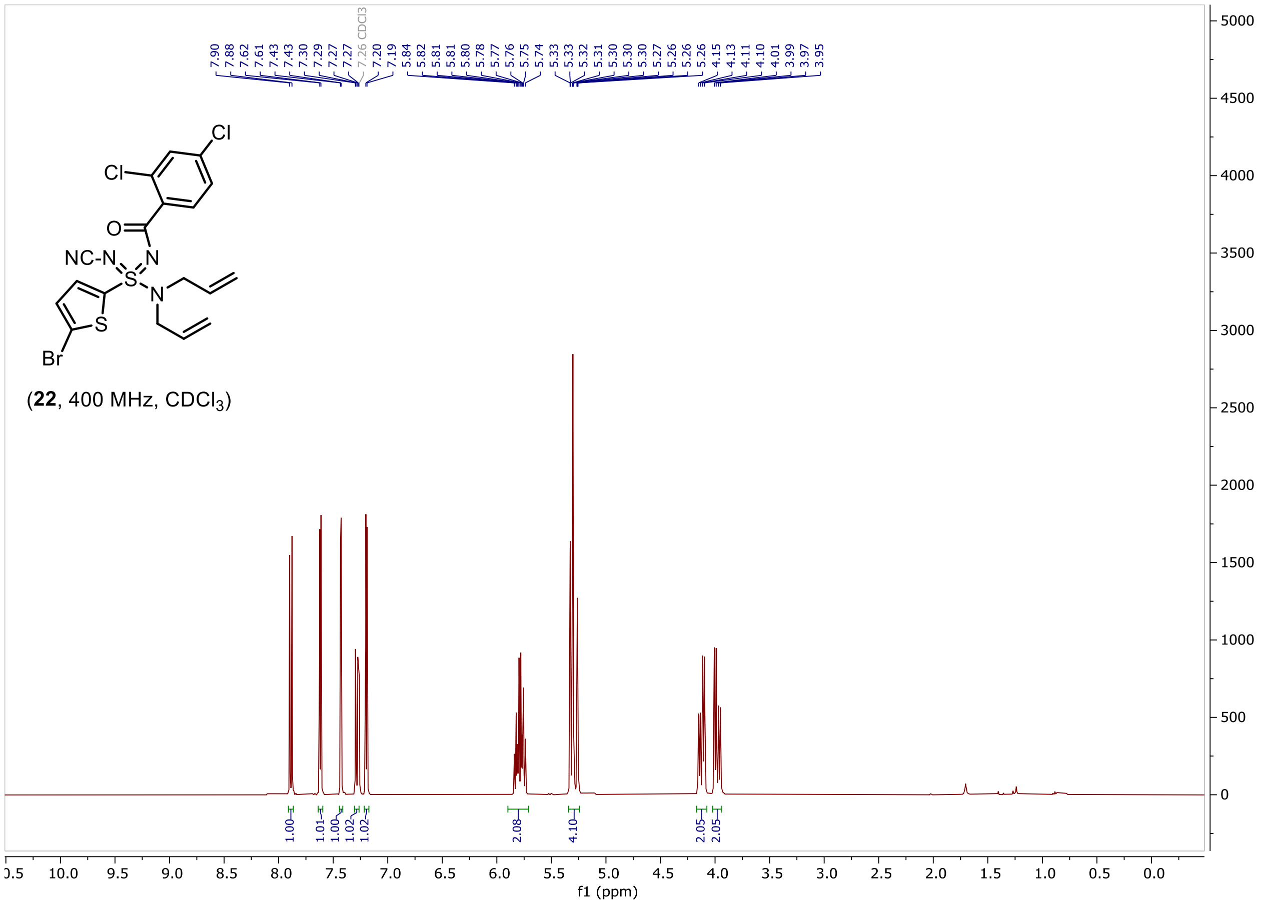

(22, 400 MHz, CDCl3)
f1 (ppm)

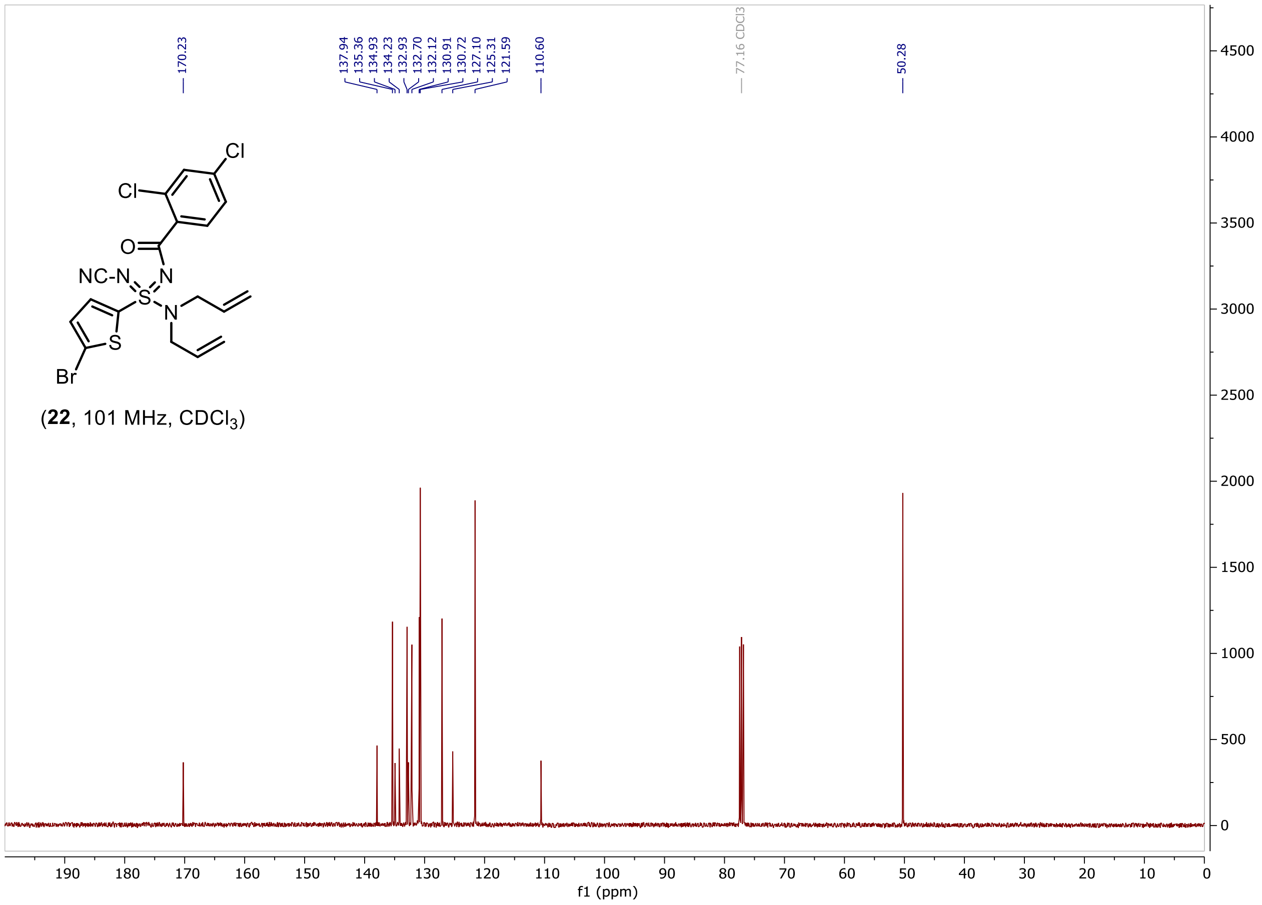

170.23
137.94
135.36
134.93
134.23
132.93
132.70
132.12
130.91
130.72
127.10
125.31
121.59
110.60
77.16 CDCl3
50.28
NC-N
N
O
Cl
Cl
S
N
S
Br
(22, 101 MHz, CDCl3)
f1 (ppm)

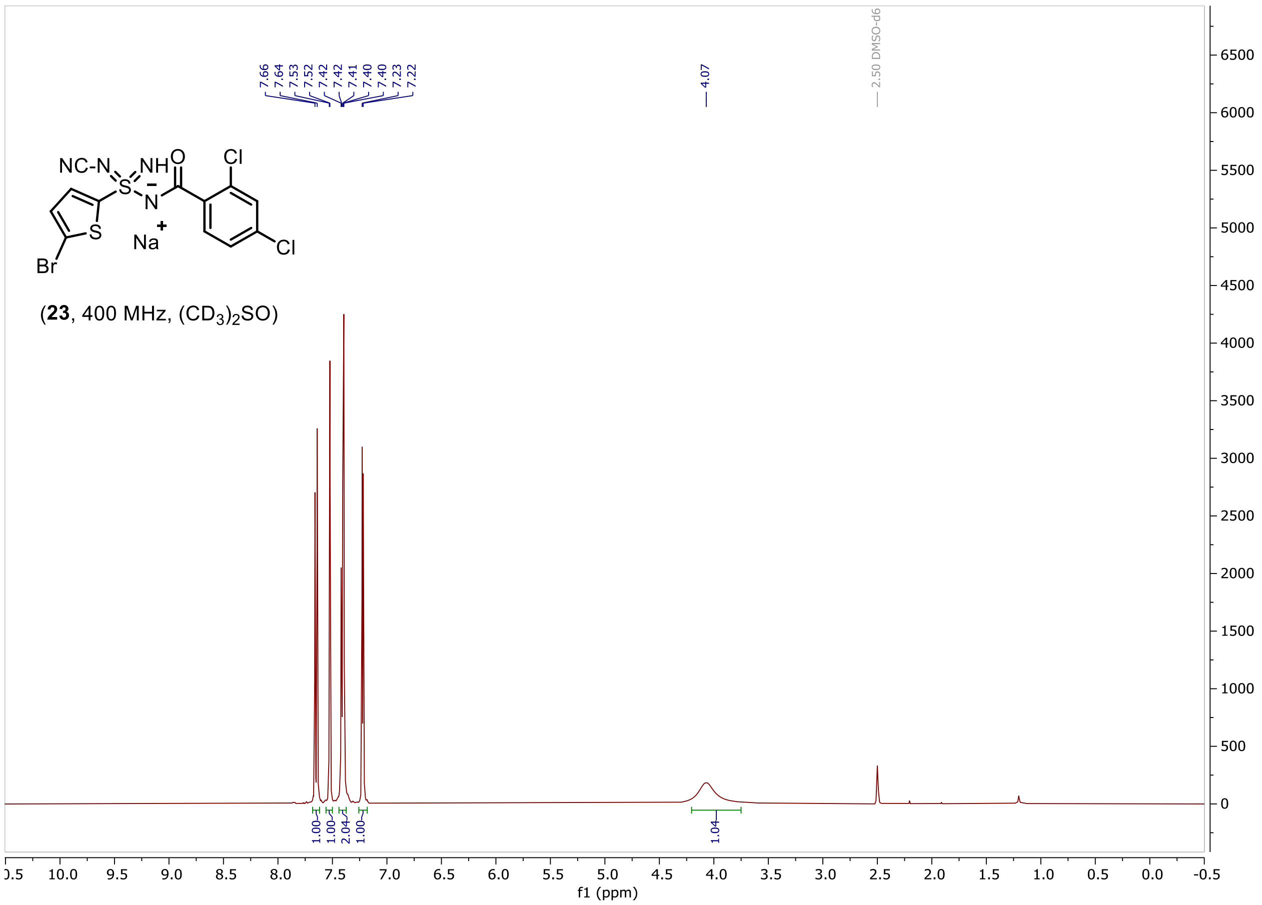

7.66
7.64
7.53
7.52
7.42
7.42
7.41
7.40
7.23
7.22
4.07
2.50 DMSO-d6
NC-N
NH
O
Cl
S
N
S
Na+
Cl
Br
(23, 400 MHz, (CD3)2SO)
1.00
1.00
2.04
1.00
1.04
f1 (ppm)

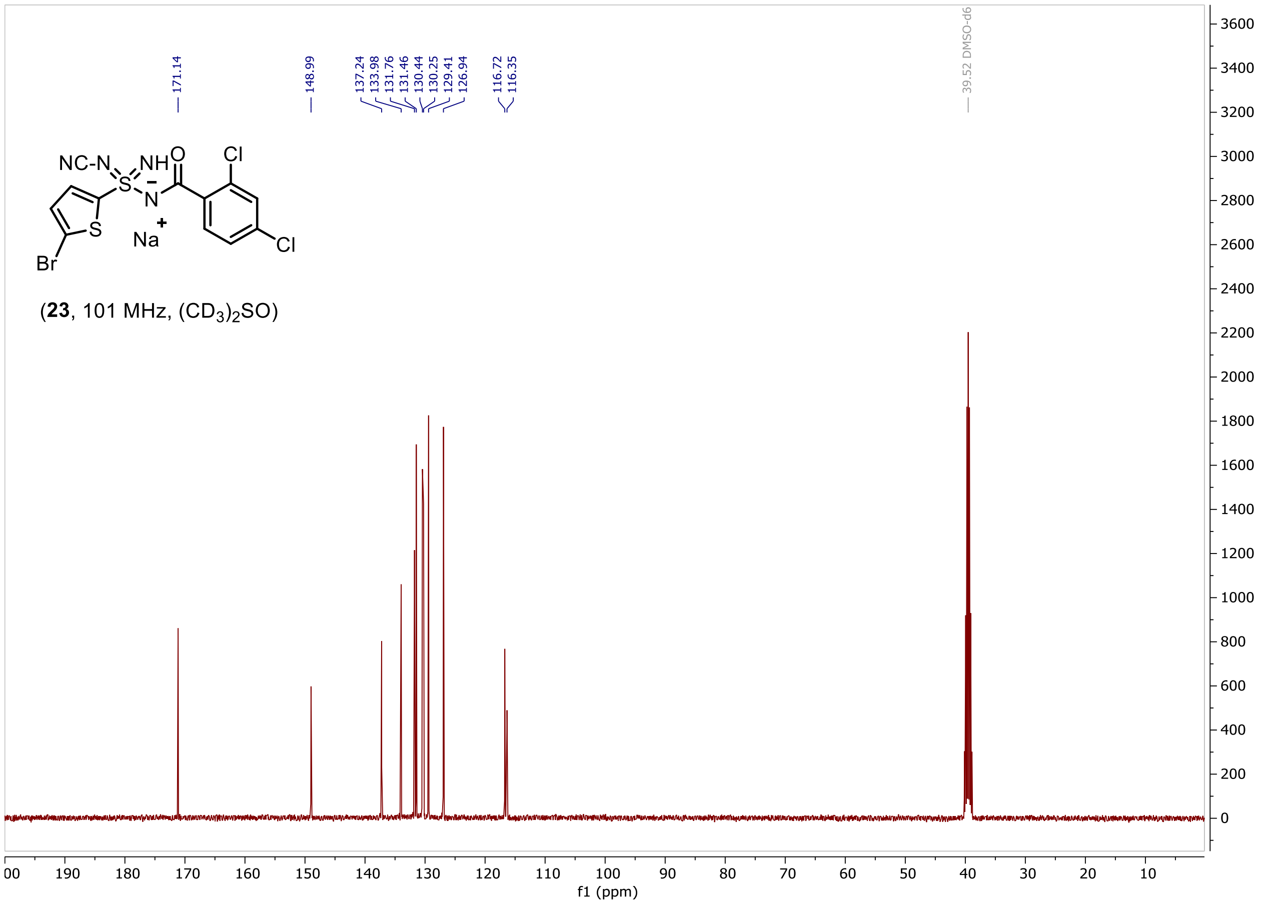
171.14
148.99
137.24
133.98
131.76
131.46
130.44
130.25
129.41
126.94
116.72
116.35
39.52 DMSO-d6
NC-N
NH
O
Cl
S
N
Na
S
Br
Cl
(23, 101 MHz, (CD3)2SO)
f1 (ppm)
